# Cyclic Bifunctional Reagents Enabling a Strain-Release-Driven Formal [3 + 2] Cycloaddition of 2*H*-Azirines by Cascade Energy Transfer

**DOI:** 10.1021/jacs.4c18080

**Published:** 2025-04-10

**Authors:** Alessia Petti, Mathis J. Karrasch, Preeti Chahar, Felix H. Wessels, Niklas Hölter, Florian Boser, Constantin G. Daniliuc, Frank Glorius

**Affiliations:** Organisch-Chemisches Institut, Universität Münster, Corrensstraße 36, Münster 48149, Germany

## Abstract

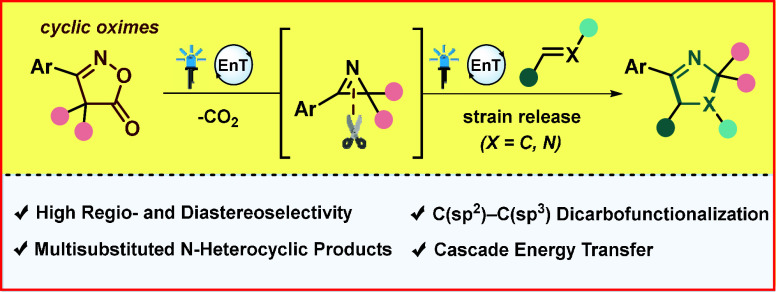

The energy transfer (EnT)-catalyzed ring opening and further decarboxylation of isoxazole-5(4*H*)-ones enables the in situ generation of strained 2*H*-azirines. Subsequent selective C(sp^2^)–C(sp^3^) bond cleavage of the azirine intermediate allows a formal [3 + 2] cycloaddition with a wide range of electrophiles, unlocking access to valuable pyrroline-type moieties. Mechanistic experiments in combination with density functional theory (DFT) calculations revealed the unique nature of the EnT-cascade process for the generation and ring opening of the three-membered aza-cycle while providing insight into the regio- and diastereoselectivity of the annulation. This mild and straightforward method ensures the rapid construction of highly substituted cyclic imines, which can be easily converted into pyrrolidines, fused oxaziridines, and biologically relevant β-amino acid precursors.

## Introduction

**Figure 1:**
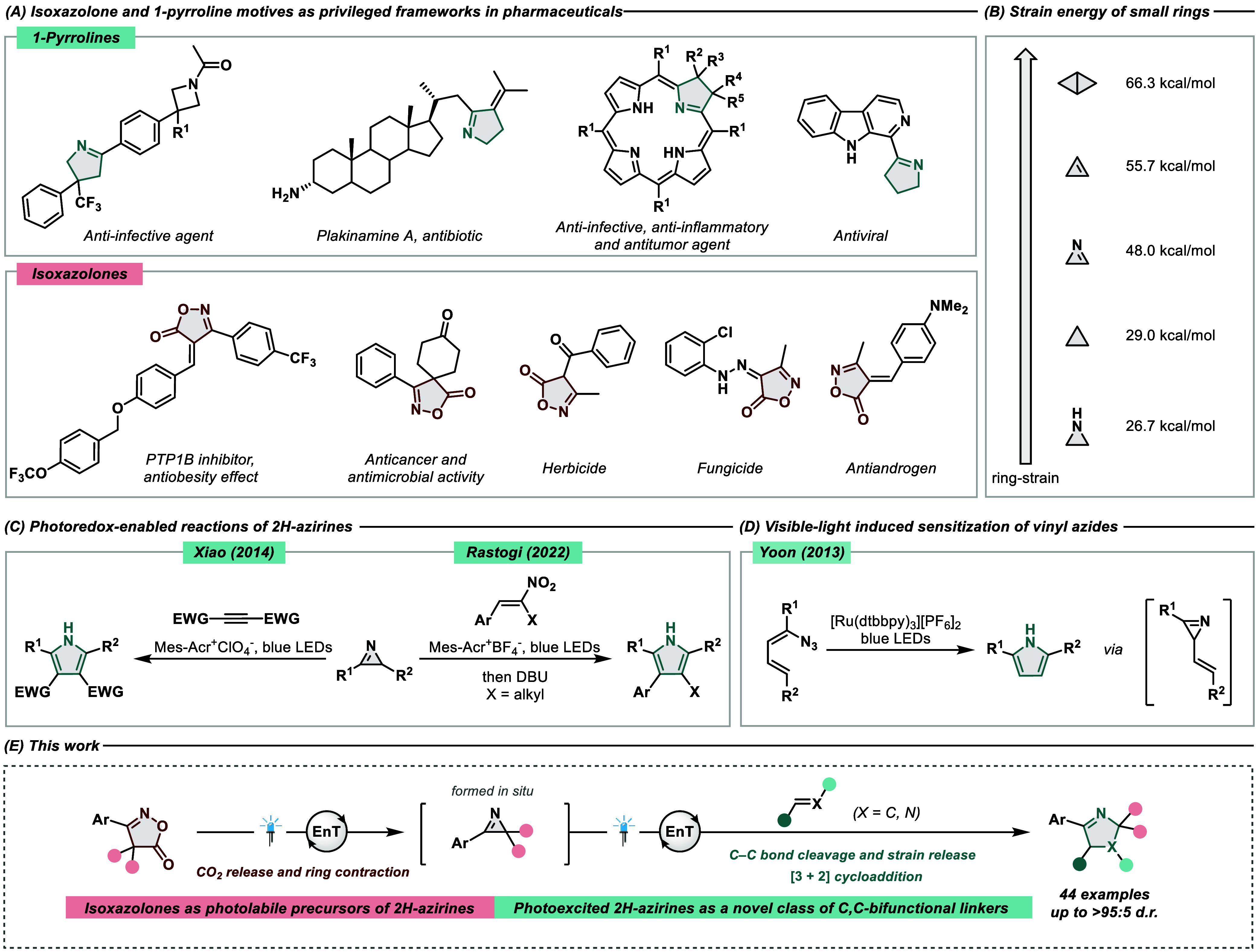
(A) 1-Pyrrolines and isoxazolones motifs in medicinal chemistry, (B) strain energy for three- and four-membered rings, (C) literature-known examples of photoredox catalysis with 2*H*-azirines, (D) literature-known example of visible-light-driven 2*H*-azirine formation and further intramolecular cyclization, and (E) this work: EnT-enabled formation and formal [3 + 2] cycloaddition of strained 2*H*-azirines.

Nitrogen-containing heterocycles represent highly sought-after motifs in drug design, with 82% of the FDA-approved small-molecule pharmaceuticals in the past decade containing at least one aza-cycle.^1^ Among them, endocyclic prochiral imines such as 1-pyrrolines and isoxazole-5(4*H*)-ones frequently appear as privileged scaffolds in medicinal chemistry (Figure 1A). Indeed, the 1-pyrroline motif can be located in several antibiotic,^2^ anti-inflammatory,^3^ and antiviral agents.^4,5^ Meanwhile, isoxazole-5(4*H*)-ones owe their popularity to their antidiabetic,^6^ fungicidal,^7^ antibiotic,^8^ and antineoplastic^9,10^ properties. Due to their inherent stability at ambient conditions, isoxazolones can also serve as bench-stable 2*H*-azirine hybrids, as recently reported by Ohe^11^ and Peters,^12^ who showcased a thermally induced and transition metal-mediated ring contraction of these scaffolds. Other synthetic approaches to access these highly strained heterocycles (Figure 1B)^13^ include, among others,^14^ the formation of unstable *N*-alkenyl nitrenes^15^ from potentially explosive vinyl azides^16^ or a base-promoted Neber^17^ rearrangement. In the latter case, strong bases are required in the absence of any anionic stabilizing group α to the iminyl carbon.^18^ Although azirine synthesis might represent a challenge due to its inherent instability, the release of strain energy associated with ring opening allows for intriguing reactivity.^19^ Some applications comprise peptide synthesis,^20^ thermal [4 + 2] cycloadditions,^21^ and the construction of various aza-cyclic compounds such as indoles, pyrazines, and aziridines.^22−24^ More recently, the photoredox activity of azirines has been investigated.^13,25^ In 2014, Xiao^26^ first reported the photoredox-enabled synthesis of polysubstituted pyrroles via single-electron oxidation of 2*H*-azirine motives in the presence of a strongly oxidizing acridinium photocatalyst. In a similar vein, Rastogi^27^ and co-workers developed a light-mediated dipolar cycloaddition between 2*H*-azirines and activated α-substituted nitroalkenes (Figure 1C). Despite the high regioselectivity of this protocol, cyclization was solely limited to nitrostyrene derivatives as olefinic electrophiles. The putative involvement of other coupling partners such as aldehydes or nitrosoarenes was also evaluated in following reports.^28−31^ Although the reactivity of 2*H*-azirines under photoredox conditions is well established, their activation via EnT is utterly underdeveloped. The latter has emerged as a complementary technique for the mild activation of organic molecules in redox-neutral processes.^32−36^ To the best of our knowledge, the only examples of 2*H*-azirine formation via EnT in the literature rely on the sensitization of alkenyl azides,^37−39^ which, due to the aforementioned instability, might be inconvenient to use (Figure 1D). On a parallel level, the homolytic cleavage of acyclic oxime esters has proven to be an ideal strategy for the difunctionalization of alkenes via the exploitation of the persistent radical effect,^40,41^ with successful examples from our group and others.^42−58^ However, in most cases, this approach relies on the formation of a transient C-centered radical and a long-lived N-centered radical, where the different polarities and lifetimes of these species dictate the order of addition. Employment of two carbon-centered radicals as bifunctional linkers remains elusive owing to intrinsic challenges in ensuring good product selectivity.^59^ Indeed, vicinal dicarbofunctionalizations can only be accomplished via transition metal catalysis, with the aid of palladium,^60^ nickel,^59^ or other metal-based catalysts.^61^ Additionally, no control of diastereoselectivity can usually be achieved when 1,2-disubstituted olefins are used as coupling partners. Willing to fill these gaps in the literature, while exploring new reactivity modes for 2*H*-azirines, we focused our efforts on designing cyclic oxime esters which could serve as bench-stable precursors of a novel class of EnT-enabled dicarbofunctionalization reagents (Figure 1E). The latter would arise from sensitization of isoxazole-5(4*H*)-ones and their subsequent decarboxylation to form the strained heterocyclic intermediate. Selective homolytic cleavage of the C(sp^2^)–C(sp^3^) bond of the 2*H*-azirine via a second energy transfer event promotes the formation of a highly reactive aza-allenyl diradical, which can react with a wide range of olefins and electrophiles, such as imines, isocyanates, and alkynes. This novel approach exploits the high reactivity of azirines while avoiding their handling by generating them in situ under mild conditions. The photoinduced formal [3 + 2] cycloaddition proceeds with a high degree of regio- and diastereoselectivity, unlocking access to partially saturated N-heterocycles such as the aforementioned 1-pyrrolines as well as 2*H*-pyrroles, phenyloxazol-5(2*H*)-imines, and 2,5-dihydro-1*H*-imidazoles with a complex substitution pattern. The partially saturated heterocycles herein obtained are particularly appealing from a synthetic and medicinal perspective, taking into account that the increase of Fsp^3^ character (escape from flatland) is known to significantly enhance properties such as solubility, potency, and selectivity to a given target for potential drug candidates.^62^

## Results and Discussion

### Method Development

Preliminary investigations started by determining the triplet energy and spin distribution for a series of cyclic oximes using the freely accessible EnTdecker^63^ machine learning-based (ML) platform. According to the model, compound **1a** was predicted to possess a triplet energy (^P^*E*_T_ = 54.9 kcal/mol) within an accessible range for commonly used photosensitizers.^32^ Additionally, the presence of a *gem*-dimethyl substitution pattern was believed to favor further ring closure to generate the desired product, in accordance with the Thorpe–Ingold effect. When **1a** was submitted to photosensitization using (Ir(dF(CF_3_)ppy)dtbbpy)[PF_6_] (**Ir–F**, *E*_T_ = 61.8 kcal/mol),^34^ a well-known sensitizer,^46^ 1-pyrroline **3a** was obtained in a 30% yield (Table 1, entry 1). The solvent effect was briefly studied, revealing that a less polar medium with a larger dipole moment such as PhCF_3_ was optimal for the reaction outcome (entries 2–4). Reaction monitoring via GC–MS analysis confirmed starting material consumption after only 3 h of irradiation (entry 5). The use of a photosensitizer with a higher triplet energy such as thioxanthone (**TXT**, *E*_T_ = 65.5 kcal/mol)^32^ led to a slight decrease in yield (entry 6), whereas no product formation was observed upon employing the highly oxidizing [Mes-Acr-Mes][ClO_4_] (**Acr-1**)^64^ photocatalyst (entry 7). Under these conditions, unreacted isoxazolone **1a** was mainly detected.

**Table 1:**
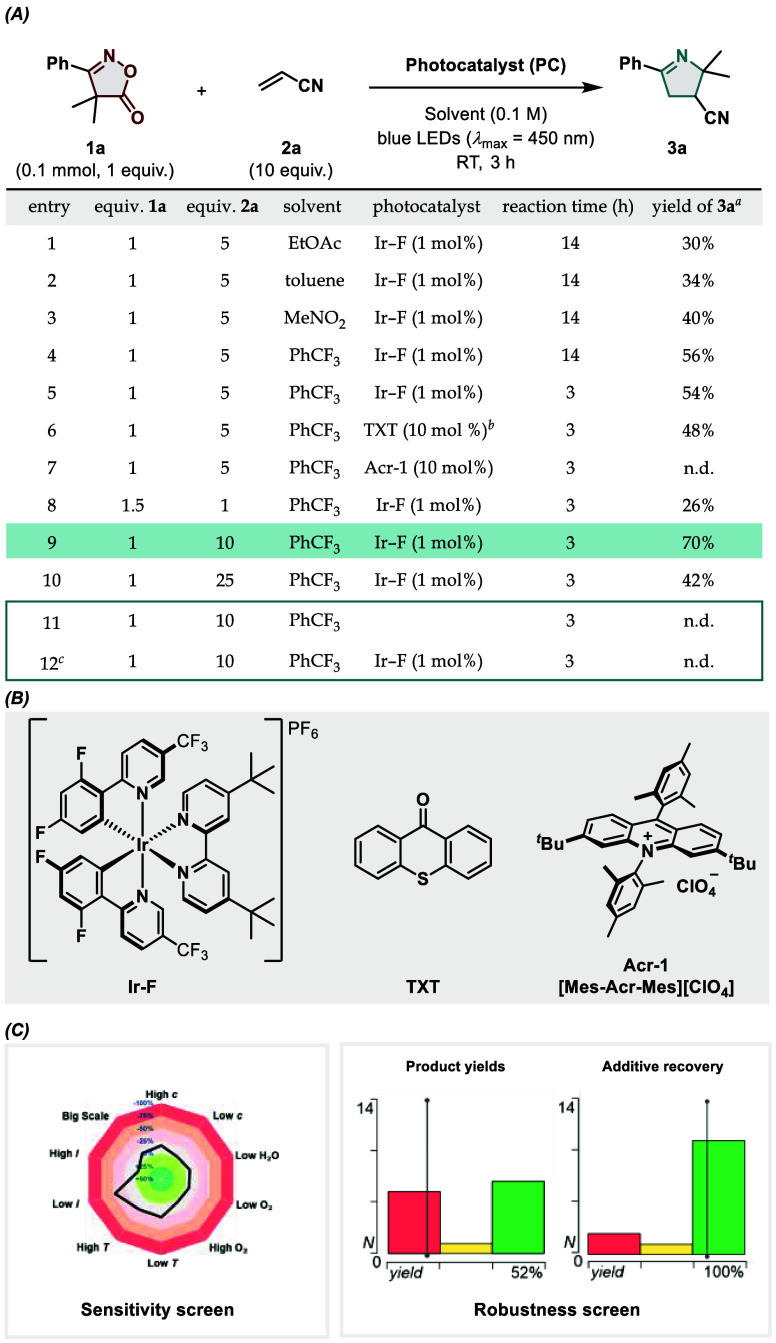
Optimization Studies and Control Reactions^a^

a Reactions were performed on a 0.1 mmol scale and analyzed by ^1^H NMR using CH_2_Br_2_ as an internal standard. n.d. = not detected.

b λ_max_ = 405 nm.

c No light. (A) Reaction optimization, (B) selected photocatalysts for the optimization studies, and (C) sensitivity and robustness assessment.

Notably, changing the oxime ester/alkene ratio (entries 8–10) showed that the optimal yield of 70% could be reached when 10 equiv of acrylonitrile was employed. The leftover mass balance was majorly represented by the CO_2_-azirine adduct (see the Supporting Information for additional information and full product characterization). Control experiments revealed that no desired product was formed in the absence of a photocatalyst or light. Tolerance toward other system perturbations was tested through a condition-based sensitivity assessment.^65^ This showcased a quite robust reaction, although the reproducibility seemed to be modestly affected by changes in light intensity. An additive-based screening^66^ was subsequently performed to assess the functional group tolerance, displaying a good tolerance for unprotected alcohols and diverse carbonyl compounds. Conversely, basic amine functionalities and aldehydes seemed to inhibit product formation (see the Supporting Information for detailed data).

### Mechanistic Analysis

**Figure 2:**
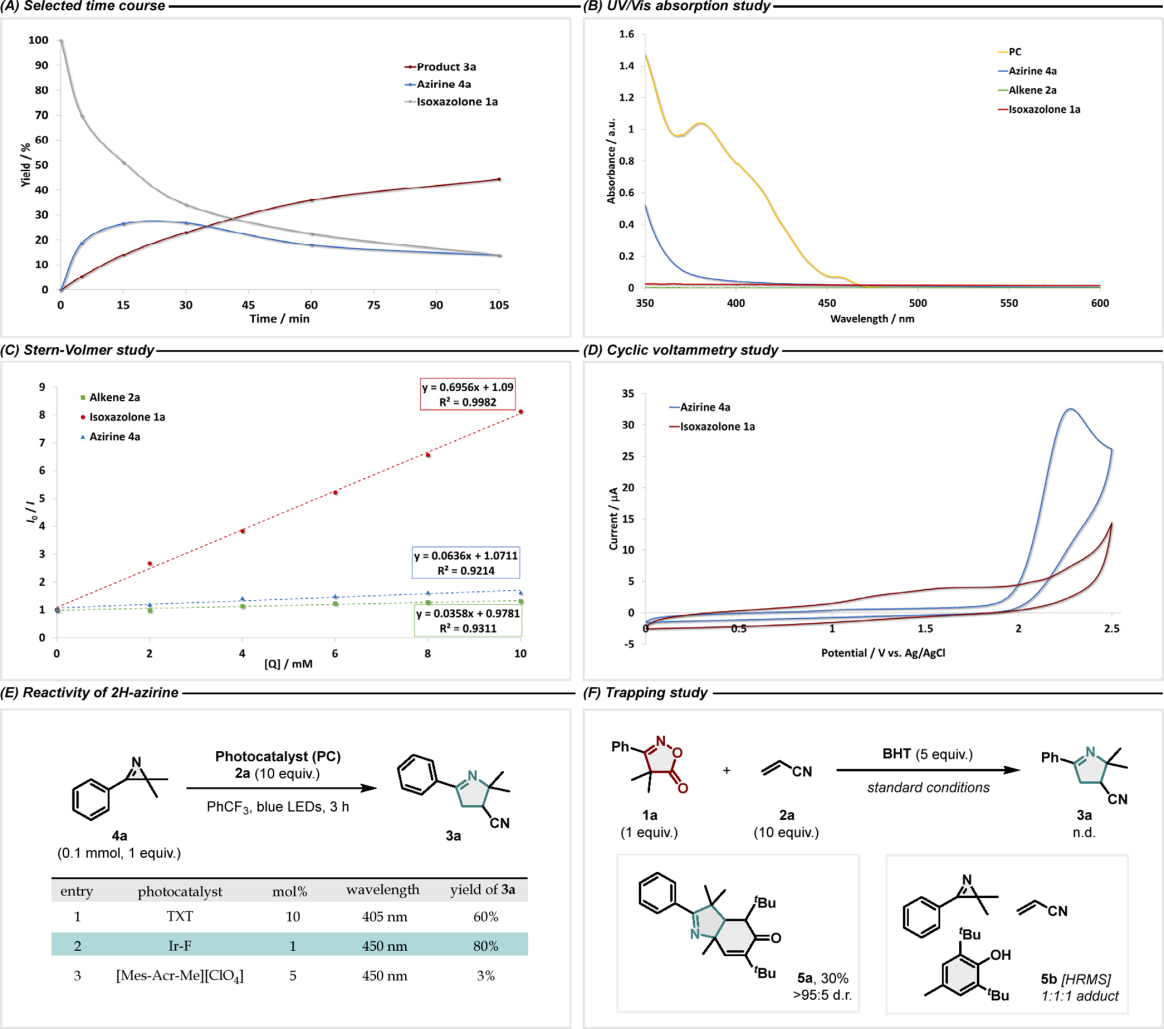
Mechanistic investigations. (A) Time-course experiment, yields are calculated by NMR using CH_2_Br_2_ as an internal standard, (B) UV–visible absorption spectrometry analysis, (C) Stern–Volmer luminescence quenching, (D) cyclic voltammetry studies conducted with degassed MeCN using Ag/AgCl (sat. KCl) as the reference electrode and then converted to the SCE scale, (E) reactivity of **4a** in the presence of photocatalysts with increasing oxidation potential, and (F) BHT trapping experiment.

**Figure 3:**
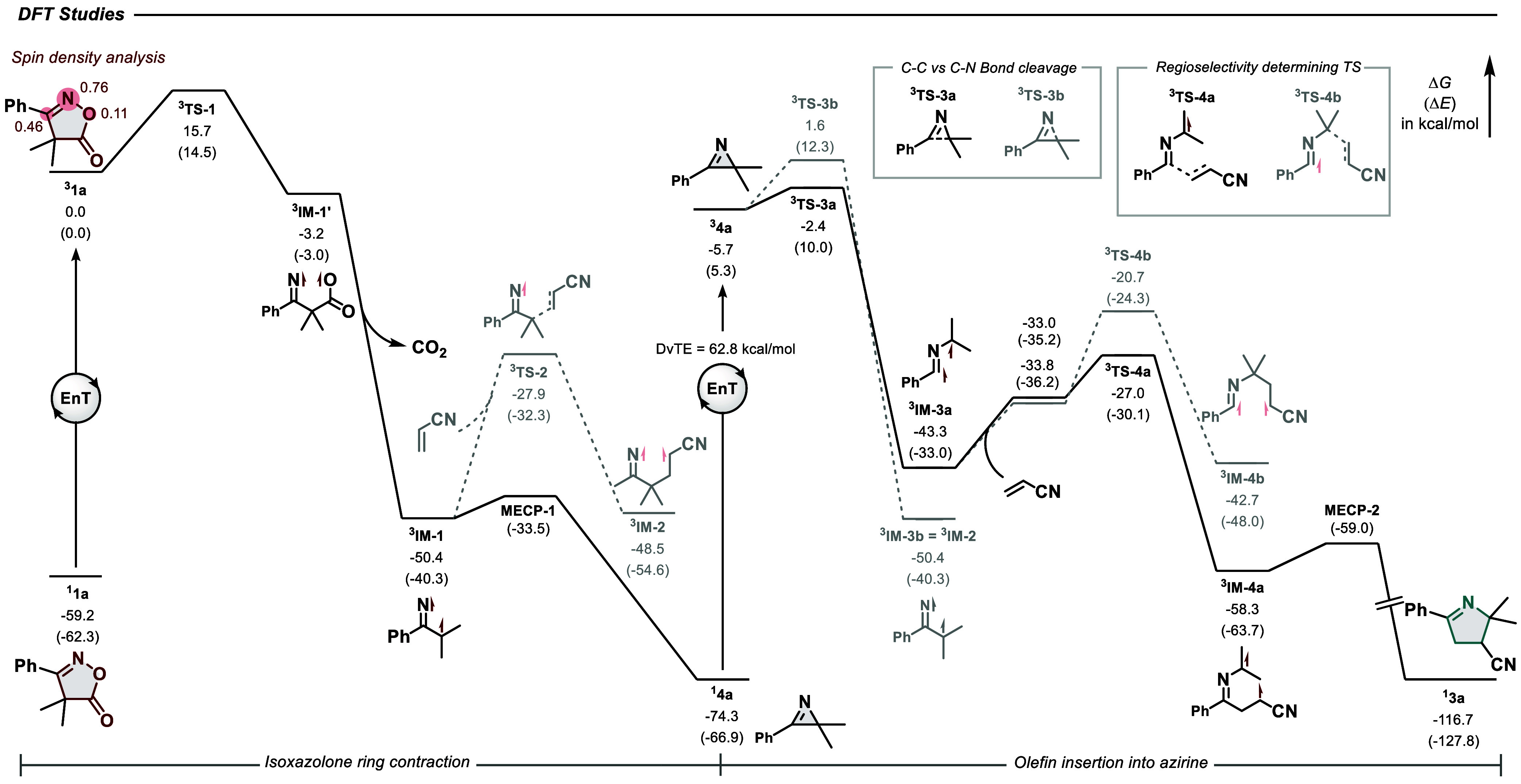
DFT-computed energy profile of the reaction coordinate, including the EnT-initiated ring contraction of isoxazolone **1a** to form 2*H*-azirine **4a**, followed by EnT-induced selective C–C bond cleavage of **4a** and addition to olefin **2a**. Calculations carried out at the B3LYP(D3)/def2-SVP//ωB97X-D3/def2-TZVPP level of theory; all energies are given in kcal/mol. DvTE = Dynamic vertical triplet energy.

After establishing the optimum reaction conditions, our efforts were directed toward defining the mechanistic blueprint as well as rationalizing the product chemo- and regioselectivity. First, we executed a time-course experiment in order to get insights into the 2*H*-azirine formation and subsequent cycloaddition (Figure 2A). When monitoring the reaction progression via ^1^H NMR, we could observe the depletion of **1a**, accompanied by the rapid formation and successive consumption of **4a**, allowing us to reach 40% of the desired product in less than 2 h of reaction time. From UV–visible absorption analysis (Figure 2B), **Ir–F** was identified as the sole absorbing species within the operating wavelength of the reaction (450 nm), thereby discounting the possibility of direct excitation of either **1a**, **2a**, or the proposed 2*H*-azirine intermediate **4a**. Stern–Volmer luminescence quenching identified isoxazole-5(4*H*)-one **1a** as the main quencher of the excited photocatalyst, while **4a** appeared approximately twice as effective as a quencher than the olefin **2a** (Figure 2C). Next, cyclic voltammetry (CV) studies were conducted for both the starting material and intermediate (**4a**) to rule out a potential photoredox pathway (Figure 2D). In this regard, cyclic oxime **1a** did not display any appreciable redox activity, while one single irreversible oxidation event was visible at 2.24 V vs SCE in MeCN for **4a**. However, this value falls significantly outside the range of the excited **Ir–F** (*E*_1/2_ (PC*/PC^·–^) = 1.21 V vs SCE),^64^ rendering a putative oxidation of **4a** by **Ir–F** unlikely. As additional proof suggesting that **4a** would undergo energy transfer rather than photoinduced oxidation, we isolated **4a** and irradiated it in the presence of three photocatalysts with increasing oxidizing power and decreasing triplet energy, respectively (Figure 2E). The results appear in accordance with the CV studies and discredit a possible photoinduced oxidation of the 2*H*-azirine. These findings emphasize the unique reactivity mode for the alkyl-disubstituted 2*H*-azirines used herein, which, until now, were less studied compared to the monosubstituted counterparts, commonly used in photoredox methodologies.^25^ The involvement of radical species in the reaction mechanism was demonstrated via trapping experiments with an excess of 2,6-di-*tert*-butyl-4-methylphenol (BHT) (Figure 2F). Formation of **3a** was not observed under these circumstances, but the 2*H*-azirine-BHT-acrylonitrile adduct **5b** could be detected by HRMS, whereas compound **5a** was isolated in a 30% yield and high d.r., further confirming the presence of **4a** as a reaction intermediate. To complete our mechanistic investigation, the energy profile along the reaction coordinate was analyzed by DFT (Figure 3). Aligning with quenching experiment results, triplet energy transfer between singlet ^1^**1a** (*E*_T_ = 59.2 kcal/mol) and excited state ^3^**Ir–F** is exergonic, resulting in ^3^**1a**, which contains most of the spin density in the isoxazolone ring. Subsequent cleavage of the N–O bond exhibits a moderate barrier of 15.7 kcal/mol (^**3**^**TS-1**), but it is followed by a barrierless and irreversible (Δ*G* = −47.2 kcal/mol) decarboxylation yielding C,N-triplet diradical ^**3**^**IM-1**. The direct coordination and addition of the transient C-centered radical of ^**3**^**IM-1** to acrylonitrile was found to exhibit a high total free energy barrier of 22.5 kcal/mol (^**3**^**TS-2**).

Instead, this diradical undergoes rapid intersystem crossing (ISC) and radical–radical recombination through a minimum energy crossing point (**MECP-1**, Δ*E*^⧧^ = 6.8 kcal/mol) to form the observed azirine intermediate ^1^**4a**. This intermediate is capable of quenching the excited state of **Ir–F** in a second, slightly endergonic, EnT-event, with a dynamic vertical triplet energy (DvTE)^67^ of 62.8 kcal/mol, yielding triplet state ^3^**4a**. From here, breaking of the azirine’s C–C bond is kinetically favored over breaking of the C–N bond (Δ*G*^⧧^ = 3.3 kcal/mol for **TS-3a** vs Δ*G*^⧧^ = 7.3 kcal/mol for **TS-3b**), thus explaining the selectivity for olefin insertion at this position. The subsequent attack of the internal sp^2^-type radical to olefin **2a** (**TS-4a**, Δ*G*^⧧^ = 6.0 kcal/mol) is kinetically and thermodynamically favored over the addition of the persistent tertiary radical (**TS-4b**, Δ*G*^⧧^ = 13.1 kcal/mol), rationalizing the observed regioselectivity of the reaction. The resulting intermediate ^**3**^**IM-4a** then undergoes facile ISC and radical–radical recombination (via **MECP-2**, Δ*E*^⧧^ = 4.7 kcal/mol) to yield the observed product **3a**.

### Reaction Scope

**Figure 4:**
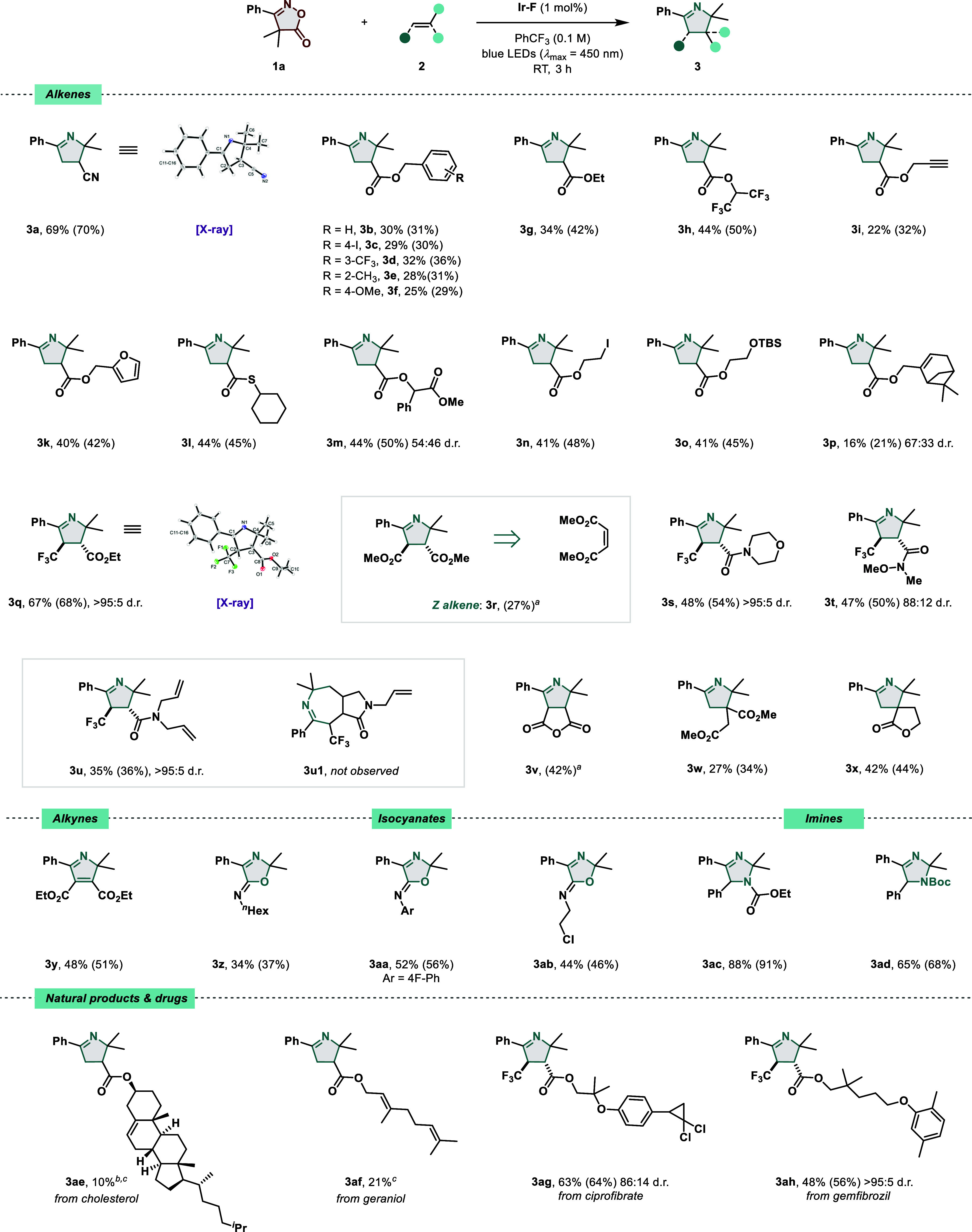
EnT-catalyzed decarboxylation and 2*H*-azirine [3 + 2] cycloaddition reaction. Standard reaction conditions: **1a** (0.2 mmol), **2** (2.0 mmol), **Ir–F** (1 mol %), PhCF_3_ (0.1 M), blue LEDs (λ_max_ = 450 nm), RT, 3 h. Isolated yields are given. NMR yields are given in brackets and calculated using CH_2_Br_2_ as an internal standard. The d.r. values were determined by ^1^H NMR and ^19^F analysis (if applicable) of the crude reaction mixture. Relative configuration is assigned by analogy to the X-ray structure of **3q**. ^a^NMR yield is solely reported due to unsuccessful isolation. ^b^Experiment performed in CH_2_Cl_2_ (0.1 M) due to poor solubility in PhCF_3._^c^Only isolated yields are given due to signal overlap in the crude.

With a thorough mechanistic analysis in hand, we tested the generality of the newly discovered methodology. To do so in a rapid and efficient manner, we resorted to high-throughput experimentation (HTE). Thus, we designed a 96-well plate with the aim of exploring the reactivity between 12 commercially available coupling partners with different stereoelectronic properties and 8 cyclic oxime esters (see the Supporting Information for detailed data). As a result of our investigations, electron-deficient mono- and disubstituted alkenes emerged as preferred reaction partners for the formal [3 + 2] cycloaddition. Disubstituted alkynes and isocyanates exhibited good to modest reactivity, while the desired product could not be detected in the case of electron-rich or nonactivated olefins, as well as sterically hindered Michael-type acceptors. Styrene derivatives, also known to be prone to photoexcitation, gave the [2 + 2] homocycloadduct as the major product. Imines, aldehydes, or nitrosoarenes, which were previously reported to react with 2*H*-azirines under photoredox conditions,^19,28,30,68^ did not afford the expected synthetic target. Once the limits of reactivity were defined, we next tested our reaction on a larger scale and in a more complex and diverse electrophile pool. First, the scope for monosubstituted alkenes was investigated using **1a** as a model substrate (Figure 4). Besides nitriles (**3a**), various benzylic esters were tolerated, although with an attenuated yield (**3b**–**3f**). Changing the substitution pattern of the aromatic ring, shifting from a deactivating trifluoromethyl group (**3d**) to a mildly activating methoxy group (**3f**), did not noticeably affect the product yield, thus demonstrating tolerance toward electronic variations. In accordance with the HTE results, augmenting the electron-withdrawing character of the Michael acceptor had a positive effect on the reaction outcome (**3g**–**3h**). Pleasingly, alkynes (**3i**), furans (**3k**), and thioesters (**3l**) were all tolerated by our protocol. When the acrylate derivative **2m** was employed as an electrophile, **3m** was formed in a good yield. Highly versatile functional handles like halides (**3n**) or protected alcohols (**3o**) could also be successfully engaged in a [3 + 2] cycloaddition reaction with **4a**. To conclude the series of monosubstituted olefins, a sterically biased 6,6-dimethylbicyclo[3.1.1]hept-2-en-2-yl acrylate was allowed to react with **1a** to afford **3p**.

Despite the modest yield, this example displays the ability of the C,C-diradical species to distinguish between the electron-poor exocyclic and the nonactivated endocyclic alkene. Next, the reactivity of *gem*-dimethyl isoxazole-5(4*H*)-one (**1a**) with 1,2-disubstituted olefins was studied. When employing ethyl (*E*)-4,4,4-trifluorobut-2-enoate **2q**, the corresponding 1-pyrroline **3q** was obtained in a 67% isolated yield and with a high degree of diastereoselectivity. Indeed, X-ray analysis confirmed the formation of the thermodynamically more stable *trans*-diastereomer. Interestingly, the cyclic iminyl product could also be formed when starting from the *cis*-alkene, as exemplified by product **3r**. Additionally, 1-pyrrolines featuring various amide groups such as morpholine (**3s**) and Weinreb amide (**3t**) were successfully obtained in good yield. In an attempt to trap the in situ generated 2*H*-azirine with the vinyl amide **2u**, the 5-membered imine **3u** was exclusively detected without any trace of the 7-membered hexahydropyrrolo[3,4-*d*]azepin-1(2*H*)-one, suggesting that the 1-pyrroline ring closure is a kinetically more favorable process than the expected 5-*exo*-trig intramolecular cyclization. Reactivity toward unsaturated anhydrides was also tested, leading to the formation of **3v**, whose isolation was, however, unsuccessful, due to product instability. At this stage, the involvement of 1,1-disubstituted alkenes was briefly evaluated, allowing for the synthesis of highly substituted N-heterocycles bearing a quaternary center at the C–3 position as in the case of diester 1-pyrroline **3w** or the aza-spiro-cycle **3x**. In an attempt to extend the substrate scope even further, we explored the usage of more diverse coupling partners beyond the alkenes. Utilization of alkynes and isocyanates, which showed promising results in the HTE screen, was therefore considered. Accordingly, 2*H*-pyrrole (**3y**) was synthesized as well as 2,2-dimethyl-4-phenyloxazol-5(2*H*)-imines (**3z**–**3ab**) bearing alkyl, aryl, or 2-chloro-ethyl moieties. It is worth noting that isocyanates chemoselectively reacted with the carbonyl rather than the iminyl fragment. This finding was confirmed by X-ray analysis (see the Supporting Information for more information) and appears in line with what has been observed for monosubstituted 2*H*-azirines.^69^ Considering the negative results shown by free electron-rich imines in the HTE, we evaluated their involvement in the reaction in a carbamate form with the aim of making the iminyl bond more electron-poor. Excellent results were obtained, which led to the regioselective synthesis of **3ac** and **3ad**. Since 1-pyrrolines can be seen as sp^3^-rich isosteres of pyrroles, with aforementioned benefits in drug design, the tolerance toward natural products and approved pharmaceuticals was studied.

**Figure 5:**
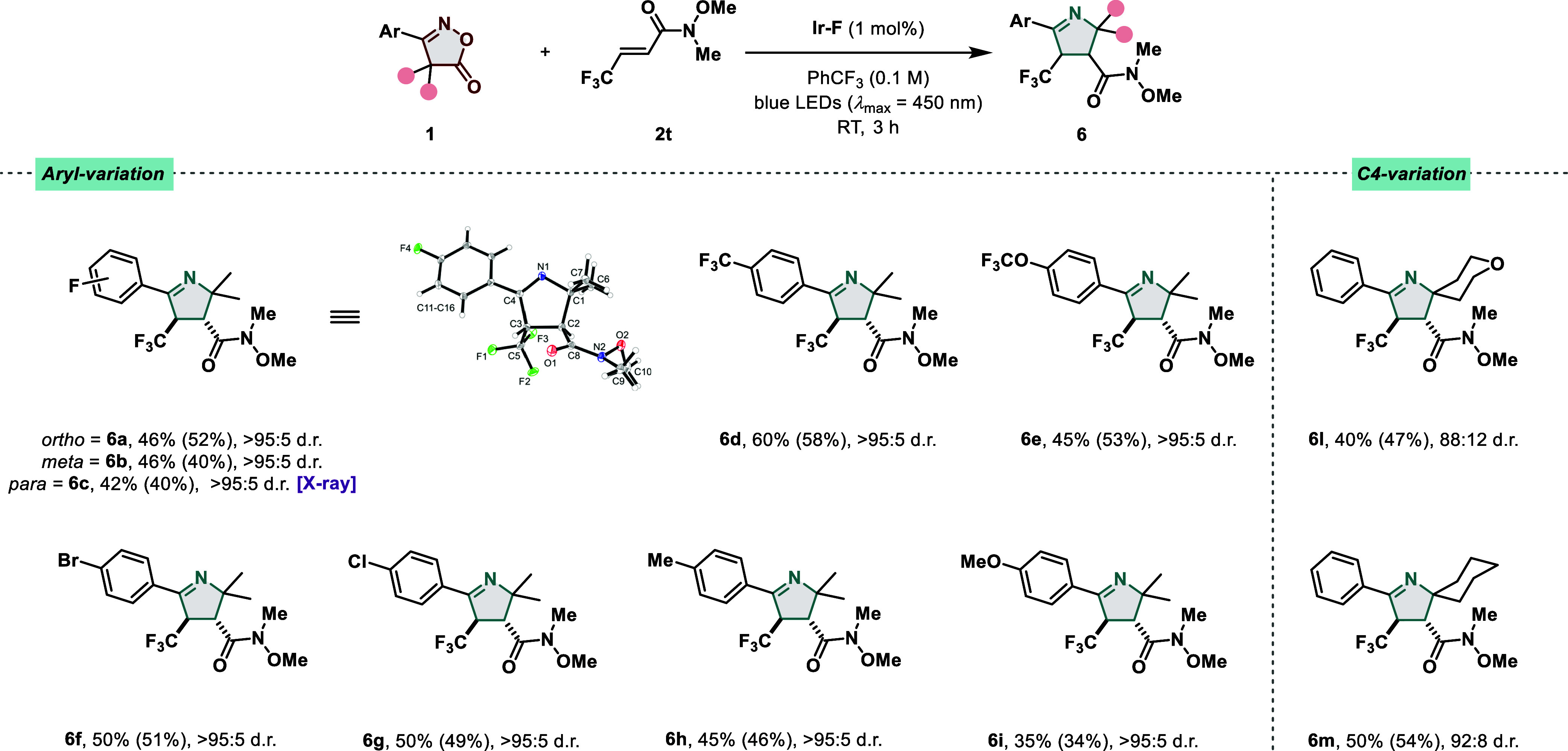
EnT-catalyzed decarboxylation and 2*H*-azirine [3 + 2] cycloaddition reaction with substituted oxime esters. Standard reaction conditions: **1** (0.2 mmol), **2t** (1.0 mmol), **Ir–F** (1 mol %), PhCF_3_ (0.1 M), blue LEDs (λ_max_ = 450 nm), RT, 3 h. Isolated yields are given. NMR yields are given in brackets and calculated with CH_2_Br_2_ as the internal standard. The d.r. values were determined by ^1^H NMR and ^19^F analysis (if applicable) of the crude reaction mixture. Relative configuration is assigned by analogy to the X-ray structure of **6c**.

Pleasingly, cholesterol- and geraniol-bearing 1-pyrrolines (**3ae**–**3af**) could be synthesized, although in low yield. In the same vein, the lipid-lowering agents ciprofibrate and gemfibrozil derivatives successfully delivered the corresponding products (**3ag**–**3ah**). Having examined electrophilic coupling partners and their substitution patterns, we next focused on the variations of the isoxazolone motif (Figure 5). Alkene **2t** was chosen as the coupling partner for this purpose due to the possibility to generate the corresponding N-heterocycles in a diastereoselective fashion. First, the effect of substituents that would decrease the electron density of the aromatic system was studied, leading to the corresponding products (**6a**–**c**) in good yields, with no substantial difference in terms of reactivity of the oxime fragment and d.r. among the *ortho*, *meta*, and *para* constitutional isomers. Compounds bearing medicinally relevant fragments such as **6d** and **6e** were also obtained in good yields with excellent diastereoselectivity. Pleasingly, *p*-Br (**6f**) and *p*-Cl (**6g**) substituents were also found to be compatible with our method, paving the way for further downstream transformations. Mildly electron-donating groups such as *p*-CH_3_ were tolerated (**6h**), while lower yields were observed when switching to more electron-rich oxime starting materials (**6i**). Next, the variation of the fragments in C–4 for compound **1** was evaluated. From our findings, these variations significantly affected the azirine formation (see the Supporting Information for substrate limitations). Nevertheless, cyclic oximes bearing cyclohexyl and 2-tetrahydropyranyl units gave rise to the synthetically interesting spirocyclic 1-pyrrolines **6l** and **6m**, respectively.

### Product Diversification

**Figure 6:**
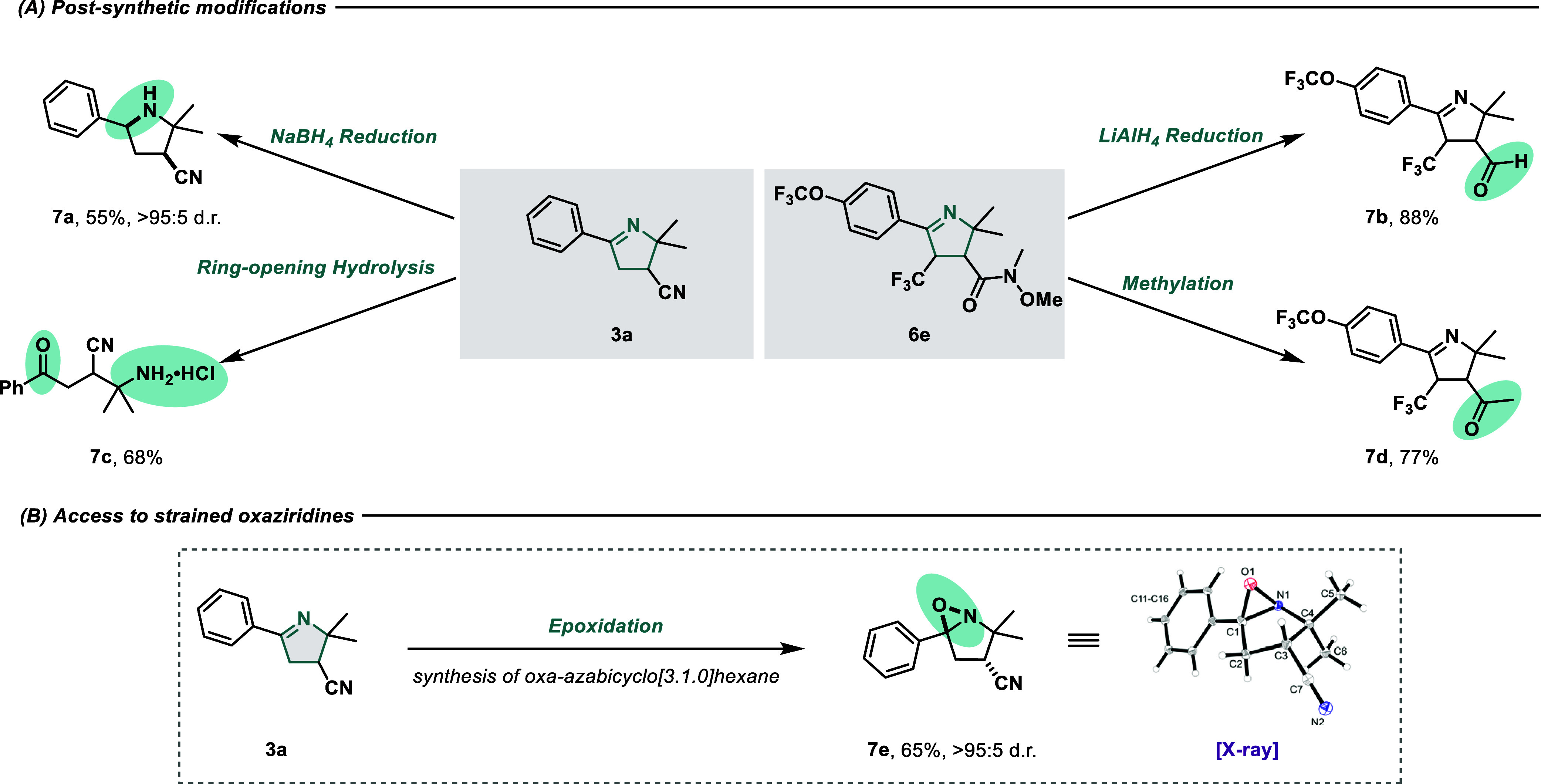
(A) Postsynthetic modifications. Reaction conditions: **3a** (0.2 mmol), NaBH_4_ (5 equiv), MeOH, 0 °C to RT, overnight; **6e** (0.2 mmol), LiAlH_4_ (5 equiv), THF, 0 °C, 3 h; **3a** (0.2 mmol), HCl 6 M, MeOH, 90 °C, 3 h; **6e** (0.2 mmol), MeMgBr (1.5 equiv), (THF, 0 °C to RT, 3 h, and B) Access to strained oxaziridines. Reaction conditions: **3a** (0.3 mmol), *m*-CPBA (1.3 equiv), Et_3_N (1.5 equiv), CH_2_Cl_2_, RT, 12 h.

To further substantiate the synthetic utility of the vicinal dicarbofunctionalization protocol, a series of derivatization experiments were carried out, exploiting the multiple potential modification sites that our product motifs offer (Figure 6A). First, a chemo- and diastereoselective reduction of the iminyl function was achieved with NaBH_4_, affording the tetra-substituted pyrrolidine **7a** as a mixture of (3*S*, 5*S*) and (3*R*, 5*R*) enantiomers (see the Supporting Information for detailed characterization). When a stronger reducing agent such as LiAlH_4_ was allowed to react with **6e**, partial reduction of the Weinreb amide to the corresponding aldehyde was observed (**7b**), leaving the 1-pyrroline backbone untouched. The hydrolysis under acidic conditions of **3a** led instead to the ring opening of the iminyl cycle and the formation of the δ-keto-iminium chloride derivative **7c**. In a similar vein, we exploited the good leaving group ability of the Weinreb amide in **6f** to synthesize the keto-pyrroline **7d** in a 77% yield. As a last postmodification effort, we performed an epoxidation of imine **3a**, unlocking access to fused oxaziridine **7e** in high d.r (Figure 6B).

## Conclusions

To summarize, we have developed a novel cascade EnT-mechanism for the generation of partially saturated N-heterocycles with a high degree of regio- and diastereoselectivity, starting from easily accessible cyclic oxime esters. Modern machine learning and high-throughput tools have been used to accelerate the creative process and chemical space discovery. Extensive mechanistic elucidation revealed the unique and unprecedented reactivity mode for the *gem*-dimethyl-2*H*-azirine intermediates, which were involved in a formal [3 + 2] cycloaddition with a wide array of electrophiles. Further derivatizations of the product motifs additionally demonstrated their extensive synthetic applicability. We foresee that this project, at a conceptual interphase between bifunctional reagents and strain-release catalysis, can represent a starting point for further investigations in the domain of N-heterocycle synthesis.

## Supporting Information Available

Materials and methods, mechanistic studies, high-throughput experiments and method limitations, computational studies, crystal structures, and spectroscopic data (PDF)
