# Supplementary material for: Cyclic Bifunctional Reagents Enabling a Strain-Release-Driven Formal [3 + 2] Cycloaddition of 2H-Azirines by Cascade Energy Transfer
Source: J Am Chem Soc. 2025 Apr 10;147(16):13276–85. doi: 10.1021/jacs.4c18080 (PMC12022991; doi:10.1021/jacs.4c18080)
Supplement: Supplementary file 1 — ja4c18080_si_001.pdf [file ja4c18080_si_001.pdf]

Supplementary Information

Cyclic Bifunctional Reagents Enabling a Strain-Release Driven Formal [3+2] Cycloaddition of 2*H*-Azirines by Cascade Energy Transfer

Alessia Petti[1,2], Mathis J. Karrasch[1,2], Preeti Chahar[1,2], Felix H. Wessels[1], Niklas Hölter[1], Florian Boser, Constantin G. Daniliuc and Frank Glorius[1,*]

[1]Organisch-Chemisches Institut, Universität Münster, Münster, Germany.

[2]These authors contributed equally.

*Correspondence to: glorius@uni-muenster.de

TABLE OF CONTENTS

1. GENERAL S3
1.1. Glassware, Solvents and Reagents S3
1.1.1. Photochemical Set-up and Light Sources S3
1.2. Chromatography and Data Analysis S4
2. EXPERIMENTAL DATA S4
2.1. General Procedures S4
2.1.1. General Procedure A: Synthesis of Cyclic Oxime Esters Starting Materials S5
2.1.2. General Procedure B: Synthesis of Cyclic Oxime Esters Starting Materials S5
2.1.3. General Procedure C: Synthesis of Cyclic Oxime Esters Starting Materials S6
2.2. Synthesis of 2,2-Dimethyl-3-phenyl-2*H*-azirine (**4a**) S14
2.3. Synthesis of Electrophilic Coupling Partners S15
2.4. Synthesis of Acryl Esters S17
2.4.1. General Procedure D: Ester Synthesis Starting from Carboxylic Acids S17
2.4.2 General Procedure E: Ester Synthesis Starting from Acyl Chlorides S18
2.5. General Procedure F: Photochemical Synthesis of Cyclic Imine Products (Alkene Scope) S26
2.6. General Procedure G: Photochemical Synthesis of Cyclic Imine Products (Oxime Scope) S27
2.7. Product Diversifications S51
2.7.1. Reduction of Imine to Substituted Pyrrolidine S51
2.7.2. Reduction of Weinreb Amide S53
2.7.3. Hydrolysis of Cyclic Imine to 1,4-Amino Ketones S53
2.7.4. Methylation of Weinreb Amide S54
2.7.5. Epoxidation of Imine to Access Fused Oxaziridine S55
2.8. Condition-Based Sensitivity Screen S56
2.9. Additive-Based Robustness Screen S57
2.10. Time-Course Study S59
2.11. Synthesis of 2,2-Dimethyl-4-phenyloxazol-5(2*H*)-one (**S6**) S60
3. MECHANISTIC STUDIES S61
3.1. UV/Vis Absorption Spectroscopy S61
3.2. Stern-Volmer Analysis S61
3.3. Cyclic Voltammetry Studies S62
3.4. Radical Trapping Studies S63
3.4.1. TEMPO Trapping Study S63
3.4.2. BHT Trapping Study S64
3.5. Activation of Azirine S66
4. HIGH-THROUGHPUT EXPERIMENTS AND METHOD LIMITATIONS S67
4.1. General Experimental S67
4.1.2. Formal [3+2] Cycloaddition of Strained Azirine Intermediates via Cascade EnT of Alkenes with

Cyclic Oxime Esters in 96 Well-plate ... S69
4.1.3. Automated Reaction Workup and Sample Preparation ... S71
4.1.4. Gas Chromatographic Analysis ... S73
4.1.5. GC-MS Evaluation and Qualitative Yield Estimation Using PyGecko Library ... S73

5. COMPUTATIONAL STUDIES ... S76
5.1. DFT Calculations ... S76
5.1.1. Calculated Energies ... S77
5.1.2. Optimized Geometries ... S78
5.2. Dynamic Vertical Triplet Energies ... S85
5.2.1. Optimized Geometry for MD Simulation ... S85
5.2.2. Vertical Triplet Energy Distribution and Dynamic Vertical Triplet Energy ... S86
5.2.3. Discussion on Energy Transfer between **Ir-F** and **4a** ... S87

6. ACKNOWLEDGEMENTS ... S87

7. CRYSTAL STRUCTURES ... S87

8. SPECTROSCOPIC DATA ... S94

9. REFERENCES ... S208

1. GENERAL

1.1. Glassware, Solvents and Reagents

All reactions were conducted under an inert atmosphere of argon using Schlenk manifold techniques unless stated otherwise. All glassware and Teflon-coated magnetic stir bars were dried in an oven at 120°C prior to use. All anhydrous solvents were commercially supplied and stored over 3 Å mol sieves or dried using an activated alumina column drying system. Reagents were purchased from commercial sources and used as received.

1.1.1. Photochemical Set-up and Light Sources

Photochemical reactions were performed in a Hepatochem EvoluChemTM PhotoRedOx Box Duo device and irradiated with two EvoluChem™ HCK1012-02-012 LEDs (18 W, λ_{max} = 450 nm). When the internal fan was used, the reaction temperature was determined to be between 30°C and 33°C.

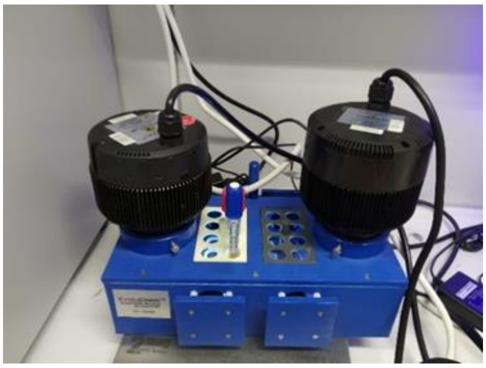

Figure S1: Experimental set-up for photochemical reactions.

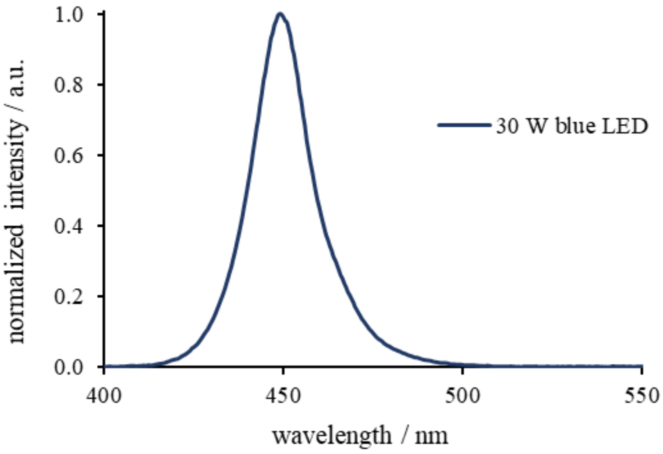

Figure S2: Emission spectrum of LEDs used (λ_{max} = 450 nm).

1.2. Chromatography and Data Analysis

Thin layer chromatography (TLC) was performed to monitor reactions when practical using Merck silica gel 60 F_{254} aluminum plates and visualized under UV light, or by staining with aqueous basic potassium permanganate followed by heating. **Flash column chromatography** (FCC) was carried out using Acros Organics silica gel (35–70 mesh) or a Biotage Isolera™ flash purification system. **NMR spectra** were recorded on a Bruker Avance II 400, Agilent DD2 500 or DD2 600 spectrometers. All spectral data was acquired at 295 K. Deuterated solvents were purchased from Eurisotop ($CDCl_3$, deuteration > 99.8%). Chemical shifts (δ) are reported in parts per million (ppm) and referenced to $CDCl_3$ (^{1}H: 7.26 ppm; ^{13}C: 77.16 ppm). Coupling constants (*J*) are given in Hertz (Hz) and refer to corresponding multiplicities (s = singlet, d = doublet, t = triplet, q = quartet, quin = quintet, hex = hextet, h = heptet, m = multiplet, app = apparent, br. = broad signal, dd = doublet of doublets, etc.). The ^{1}H NMR spectra are reported as follows: chemical shift (multiplicity, coupling constants, number of protons). NMR assignments were made according to spin systems, using two-dimensional NMR.

2. EXPERIMENTAL DATA

2.1. General Procedures

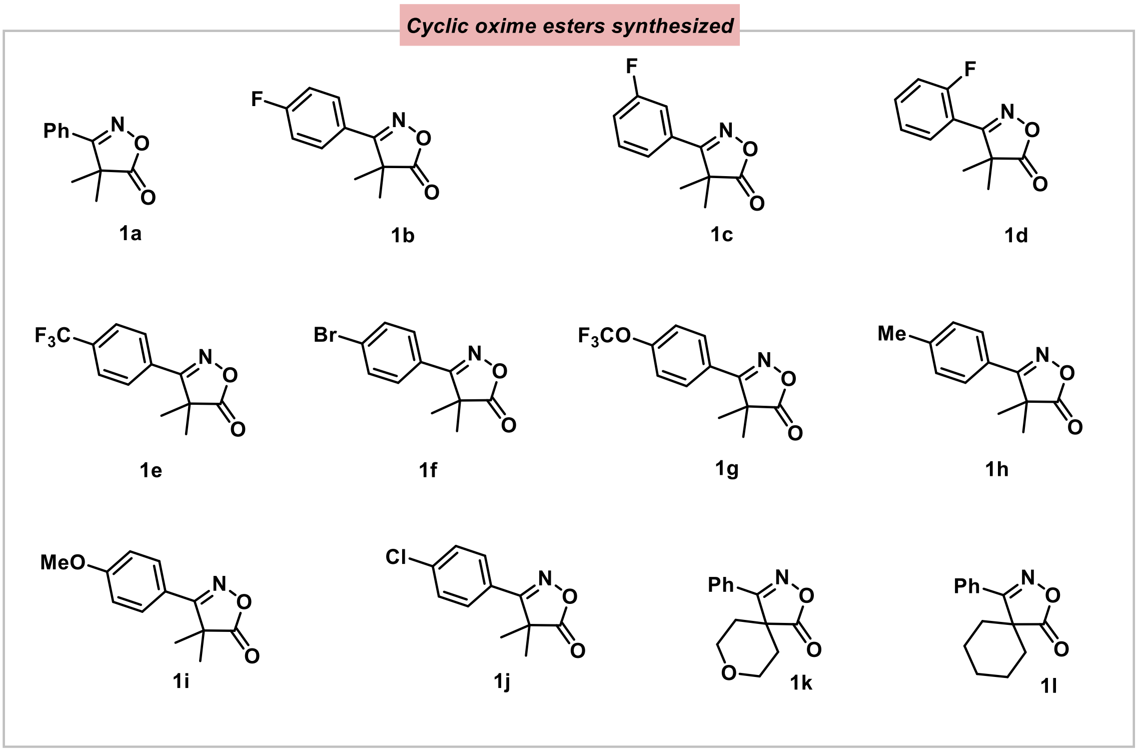

2.1.1. General Procedure A: Synthesis of Cyclic Oxime Esters Starting Materials

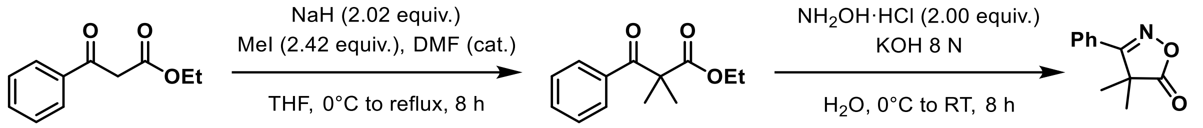

1a

Step 1:

MeI (1.38 mL, 24.20 mmol, 2.42 equiv.) was added dropwise at 0°C to a suspension of ethyl 3-oxo-3-phenylpropanoate (1.73 mL, 10.00 mmol, 1.00 equiv.), DMF (1 mL) and NaH (60% suspension in mineral oil, 0.88 g, 20.04 mmol, 2.02 equiv.) in dry THF (35 mL). The resulting mixture was refluxed for 8 h under inert atmosphere. H_2O (10 mL) was added, the mixture was extracted with EtOAc (3 x 20 mL), and the reunited organic phases were dried over Na_2SO_4 and evaporated. The resulting crude was used for the next step without further purification.[1]

Step 2:

To a stirred solution of NH_2OH•HCl (0.77 g, 11.00 mmol, 1.10 equiv.) in minimum amount of H_2O at 0 °C was added 8 N of KOH (1.12 g, 20.00 mmol, 2.00 equiv.) in H_2O and ethyl 2,2-dimethyl-3-oxo-3-phenylpropanoate (10.00 mmol, 1.00 equiv.) dropwise. The reaction mixture was allowed to stir at 0°C for 8 h. The reaction was quenched by 1 N aqueous HCl and the organic materials were extracted three times with CH_2Cl_2. The combined extracts were washed with sat. $NaHCO_3$ and dried over $MgSO_4$. The crude material was purified by flash column chromatography (SiO_2, pentane/EtOAc = 50:1 to 9:1) to give isoxazolone **1a** (1.13 g, 5.97 mmol, 60%) as a white solid.[1]

TLC: R_f = 0.63 (9:1 pentane/EtOAc).

NMR Spectroscopy (*see spectra*):

^{1}H NMR (400 MHz, $CDCl_3$): δ_H 7.79 – 7.73 (m, 2H), 7.58 – 7.43 (m, 3H), 1.61 (s, 6H) ppm;

^{13}C NMR (101 MHz, $CDCl_3$): δ_C 181.9, 169.8, 131.8, 129.3, 127.8, 127.1, 45.9, 23.2 ppm.

2.1.2. General Procedure B: Synthesis of Cyclic Oxime Esters Starting Materials

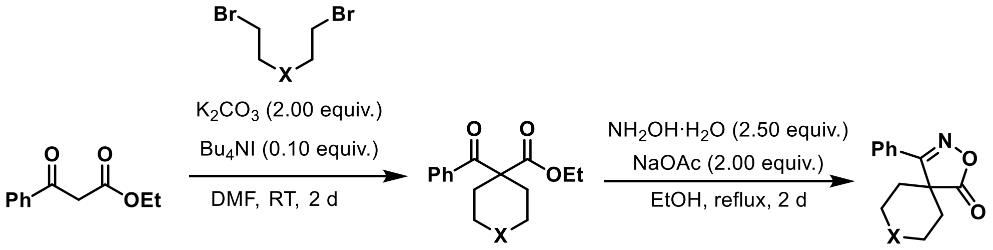

Step 1:

Dibromide (1.10 equiv.) was added dropwise to a suspension of ethyl 3-oxo-3-phenylpropanoate (1.00 equiv.), tetrabutylammonium iodide (0.10 equiv.) and K_2CO_3 (2.00 equiv.) in dry DMF (0.50 M). The resulting mixture was stirred at room temperature for 2 d under inert atmosphere. H_2O (20 mL) was added, the mixture was extracted with EtOAc (3x 20 mL) and the combined organic phases were washed with water (3x 20 mL) and brine (1x 20 mL), dried over $MgSO_4$ and evaporated. The crude material was purified by flash column chromatography.[2]

Step 2:

The product from Step 1 was dissolved in EtOH (0.50 M) and NH_2OH•HCl (2.50 equiv.), NaOAc (2.00 equiv.) and H_2O (5.00 M) was added. The reaction mixture was refluxed for 2 d. H_2O (20 mL) was added, the mixture was extracted with EtOAc (3x 20 mL) and the combined organic phases were washed with brine (1x 20 mL), dried over $MgSO_4$ and evaporated. The crude material was purified by flash column chromatography.[2]

2.1.3. General Procedure C: Synthesis of Cyclic Oxime Esters Starting Materials

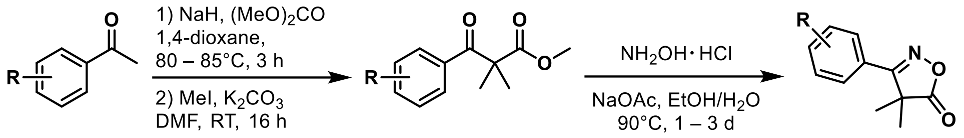

Step 1:

An oven-dried 100 mL two-neck round bottom flask was equipped with a reflux condenser and rubber septum and evacuated and backfilled with argon three times. Then, sodium hydride (60% in mineral oil, 2.80 equiv.) was suspended in 1,4-dioxane (1.00 M). Furthermore, dimethyl carbonate (2.80 equiv.) was added via syringe and the reaction was heated to 80°C. Then, the aryl ketone was added dropwise via syringe or in portions as a solid dissolved a minimum amount of dry 1,4-dioxane. After hydrogen evolution was observed, the reaction was heated to 80°C for two more hours, after which it was cooled to room temperature and then to 0°C to quench with saturated aq. NH_4Cl-solution. Then, the crude reaction mixture was extracted with EtOAc (3x 20 mL). The combined organic phases were dried over $MgSO_4$ and after filtration the solvent was evaporated *in vacuo*. The crude β-ketoester was directly used for the next step without further purification.[3]

Step 2:

The crude product from the first step was dissolved in DMF (0.36 M) in a 150 mL oven-dried Schlenk tube, which was evacuated and backfilled with argon three times beforehand. Then, K_2CO_3 (4.00 equiv.) was added and the reaction was stirred for 30 min at room temperature. Then, MeI (4.00 equiv.) was added and the reaction mixture was stirred at room temperature overnight. The next day, water (0.36 M) was added and then extracted with EtOAc (3x 20 mL). The combined organic phases were extracted with water (3x 20 mL) and brine (1x 20 mL) and dried over $MgSO_4$. After filtration, the solvent was evaporated the crude product filtered over a small silica plug to yield methylated β-ketoester, which was then directly used for the next step, whereas the amount

of substance of the intermediate was estimated by the crude mass.[4]

Step 3:

A 100 mL pressure tube was evaporated and backfilled with argon three times. Then, NH_2OH·HCl (2.50 equiv.) was suspended in EtOH (0.66 M) and H_2O (3.33 M) and NaOAc (2.00 equiv.) was added. Then, methylated β-ketoester from the previous step was added and the reaction mixture stirred under reflux for one to three days, whereas the reaction progress was monitored by TLC. Upon completion, the reaction was cooled down to room temperature and water and EtOAc were added. The aqueous phase was extracted with EtOAc (3x 20 mL) and the organic phases were combined, dried over $MgSO_4$ and filtrated. The crude product was purified via silica-gel flash column chromatography to yield the corresponding cyclic oxime ester (in case of non-sufficient purity recrystallize from EtOAc/pentane mixtures).[4,5]

3-(4-Fluorophenyl)-4,4-dimethylisoxazol-5(4*H*)-one (1b)

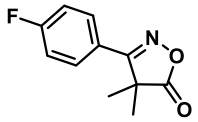

1b

Synthesized following **General Procedure C** using: 1-(4-fluorophenyl)ethan-1-one (1.20 mL, 10.00 mmol, 1.00 equiv.), sodium hydride (1.12 g, 28.00 mmol, 2.80 equiv.), dimethyl carbonate (1.60 mL, 20.00 mmol, 2.00 equiv.), and 1,4-dioxane (10 mL, 1.00 M) for step one, potassium carbonate (5.53 g, 40.00 mmol, 4.00 equiv.), methyl iodide (2.50 mL, 40.00 mmol, 4.00 equiv.), and DMF (28 mL, 0.36 M) for step two yield the methylated β-ketoester intermediate as a yellow oil. For the third step, NH_2OH·HCl (1.65 g, 23.80 mmol, 2.50 equiv.), NaOAc (1.56 g, 19.00 mmol, 2.00 equiv.), EtOH (14.40 mL, 0.66 M) and H_2O (2.90 mL, 3.33 M) were used. Purified by flash column chromatography (SiO_2; 90:10 pentane/EtOAc) to afford **1b** (1.09 g, 5.26 mmol, 53%) as a white solid over three steps.

NMR Spectroscopy (*see spectra*):

^{1}H NMR (400 MHz, $CDCl_3$): δ_H 7.81 – 7.74 (m, 1H), 7.23 – 7.13 (m, 1H), 1.60 (s, 3H) ppm;

^{19}F NMR (377 MHz, $CDCl_3$): δ_F -106.84 ppm;

^{13}C NMR (126 MHz, $CDCl_3$): δ_C 181.7, 168.9, 164.8 (d, *J* = 253.7 Hz), 129.3 (d, *J* = 8.6 Hz), 124.0 (d, *J* = 3.3 Hz), 116.7 (d, *J* = 21.9 Hz), 45.7, 23.2 ppm.

HRMS (ESI$^+$): m/z calc'd for $C_{11}H_{12}NO_2FNa$ $[M+Na]^+$: 230.0588, found: 230.0588.

3-(3-Fluorophenyl)-4,4-dimethylisoxazol-5(4*H*)-one (1c)

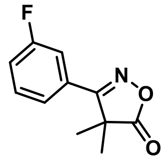

1c

Synthesized following **General Procedure C** using: 1-(3-fluorophenyl)ethan-1-one (0.61 mL, 5.00 mmol, 1.00 equiv.), sodium hydride (0.56 g, 14.00 mmol, 2.80 equiv.), dimethyl carbonate (0.85 mL, 10.00 mmol, 2.00 equiv.), and 1,4-dioxane (5 mL, 1.00 M) for step one, potassium carbonate (2.76 g, 20.00 mmol, 4.00 equiv.), methyl iodide (1.25 mL, 20.00 mmol, 4.00 equiv.), and DMF (14 mL, 0.36 M) for step two yield the methylated β-ketoester intermediate as a yellow oil. For the third step, $NH_2OH{\cdot}HCl$ (0.94 g, 10.50 mmol, 2.50 equiv.), NaOAc (0.69 g, 8.40 mmol, 2.00 equiv.), EtOH (6.40 mL, 0.66 M) and H_2O (1.30 mL, 3.33 M) were used. Purified by flash column chromatography (SiO_2; 90:10 pentane/EtOAc) to afford **1c** (0.45 g, 2.16 mmol, 22%) as a white solid over three steps.

NMR Spectroscopy (*see spectra*):

^{1}H NMR (400 MHz, $CDCl_3$): δ_H 7.58 – 7.43 (m, 1H), 7.27 – 7.20 (m, 1H), 1.61 (s, 2H) ppm;

^{19}F NMR (377 MHz, $CDCl_3$): δ_F -110.47 ppm;

^{13}C NMR (126 MHz, $CDCl_3$): δ_C 181.5, 168.9 (d, *J* = 2.9 Hz), 163.0 (d, *J* = 248.0 Hz), 131.1 (d, *J* = 8.1 Hz), 129.7 (d, *J* = 7.6 Hz), 122.8 (d, *J* = 2.9 Hz), 118.9 (d, *J* = 21.5 Hz), 114.1 d, *J* = 23.4 Hz), 45.7, 23.1 ppm.

HRMS (ESI$^+$): m/z calc'd for $C_{11}H_{12}NO_2FNa$ $[M+Na]^+$: 230.0588, found: 230.0588.

3-(2-Fluorophenyl)-4,4-dimethylisoxazol-5(4*H*)-one (1d)

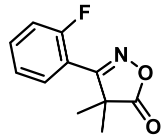

1d

Synthesized following **General Procedure C** using: 1-(2-fluorophenyl)ethan-1-one (1.20 mL, 10.00 mmol, 1.00 equiv.), sodium hydride (1.12 g, 28.00 mmol, 2.80 equiv.), dimethyl carbonate (1.60 mL, 20.00 mmol, 2.00 equiv.), and 1,4-dioxane (10 mL, 1.00 M) for step one, potassium carbonate (5.53 g, 40.00 mmol, 4.00 equiv.), methyl iodide (2.50 mL, 40.00 mmol, 4.00 equiv.), and DMF (28 mL, 0.36 M) for step two yield the methylated β-ketoester intermediate as a yellow oil. For the third step, $NH_2OH{\cdot}HCl$ (0.61 g, 8.75 mmol, 2.50 equiv.), NaOAc (0.56 g, 7.00 mmol, 2.00 equiv.), EtOH (5.30 mL, 0.66 M) and H_2O (1.00 mL, 3.33 M) were used. Purified by flash column chromatography (SiO_2; 95:5 pentane/EtOAc) to afford **1d** (0.14 g, 0.66 mmol, 7%) as a white solid over three steps.

NMR Spectroscopy (*see spectra*):

^{1}H NMR (400 MHz, $CDCl_3$): δ_H 7.62 – 7.48 (m, 2H), 7.34 – 7.16 (m, 2H), 1.49 (d, *J* = 1.7 Hz, 6H) ppm;

^{19}F NMR (377 MHz, $CDCl_3$): δ_F -109.38 ppm;

^{13}C NMR (126 MHz, $CDCl_3$): δ_C 181.4, 168.5 (d, J = 2.4 Hz), 160.1 (d, J = 252.7 Hz), 133.4 (d, J = 8.6 Hz), 130.3 (d, J = 3.3 Hz), 124.9 (d, J = 3.3 Hz), 116.9 (d, J = 21.9 Hz), 115.9 (d, J = 14.8 Hz), 46.8, 22.1 (d, J = 4.8 Hz) ppm.

HRMS (ESI^+): m/z calc'd for $C_{11}H_{12}NO_2FNa$ $[M+Na]^+$: 230.0588, found: 230.0587.

4,4-Dimethyl-3-(4-(trifluoromethyl)phenyl)isoxazol-5(4*H*)-one (1e)

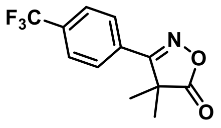

1e

Synthesized following **General Procedure C** using: 1-(4-(trifluoromethyl)phenyl)ethan-1-one (1.88 g, 10.00 mmol, 1.00 equiv., dissolved in 5 mL 1,4-dioxane), sodium hydride (1.12 g, 28.00 mmol, 2.80 equiv.), dimethyl carbonate (1.60 mL, 20.00 mmol, 2.00 equiv.), and 1,4-dioxane (10 mL, 0.67 M) for step one, potassium carbonate (5.53 g, 40.00 mmol, 4.00 equiv.), methyl iodide (2.50 mL, 40.00 mmol, 4.00 equiv.), and DMF (28 mL, 0.36 M) for step two yield the methylated β-ketoester intermediate as a yellow oil. For the third step, $NH_2OH{\cdot}HCl$ (1.04 g, 15.00 mmol, 2.50 equiv.), NaOAc (0.98 g, 12.00 mmol, 2.00 equiv.), EtOH (9.10 mL, 0.66 M) and H_2O (1.80 mL, 3.33 M) were used. Purified by flash column chromatography (SiO_2; 95:5 pentane/EtOAc) to afford **1e** (1.09 g, 5.26 mmol, 53%) as a white solid over three steps.

NMR Spectroscopy (*see spectra*)

^{1}H NMR (400 MHz, $CDCl_3$): δ_H 7.89 (dt, J = 8.0, 0.8 Hz, 1H), 7.76 (dt, J = 8.3, 0.8 Hz, 1H), 1.62 (s, 4H) ppm;

^{19}F NMR (377 MHz, $CDCl_3$): δ_F -63.23 ppm;

^{13}C NMR (126 MHz, $CDCl_3$): δ_C 181.3, 168.8, 133.5 (q, J = 33.1 Hz), 131.2, 127.5, 126.3 (q, J = 3.8 Hz), 123.6 (q, J = 272.5 Hz), 45.6, 23.3 ppm.

HRMS (ESI^+): m/z calc'd for $C_{12}H_{10}NO_2F_3Na$ $[M+Na]^+$: 280.0556, found: 280.0556.

3-(4-Bromophenyl)-4,4-dimethylisoxazol-5(4*H*)-one (1f)

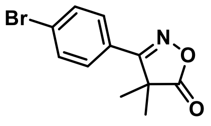

1f

Synthesized following **General Procedure C** using: 1-(4-bromophenyl)ethan-1-one (2.00 g, 10.00 mmol, 1.00 equiv., transferred with 2 mL 1,4-dioxane), sodium hydride (1.12 g, 28.00 mmol, 2.80 equiv.), dimethyl carbonate (1.60 mL, 20.00 mmol, 2.00 equiv.), and 1,4-dioxane (10 mL, 0.67 M) for step one, potassium carbonate (5.53 g, 40.00 mmol, 4.00 equiv.), methyl iodide (2.50 mL, 40.00 mmol, 4.00 equiv.), and DMF

(28 mL, 0.36 M) for step two yield the methylated β-ketoester intermediate as a yellow oil. For the third step, $NH_2OH\cdot HCl$ (1.48 g, 21.30 mmol, 2.50 equiv.), NaOAc (1.39 g, 17.00 mmol, 2.00 equiv.), EtOH (12.9 mL, 0.66 M) and H_2O (2.6 mL, 3.33 M) were used. Purified by flash column chromatography (SiO_2; 90:10 pentane/EtOAc) to afford **1f** (1.23 g, 4.59 mmol, 46%) as a white solid over three steps.

NMR Spectroscopy (*see spectra*)

^{1}H NMR (400 MHz, $CDCl_3$): δ_H 7.63 (s, 4H), 1.60 (s, 6H) ppm;

^{13}C NMR (101 MHz, $CDCl_3$): δ_C 181.6, 169.0, 132.7, 128.4, 126.6, 126.6, 45.6, 23.1 ppm.

HRMS (ESI^+): m/z calc'd for $C_{11}H_{10}NO_2BrNa$ $[M+Na]^+$: 289.9787, found: 289.9786.

4,4-Dimethyl-3-(4-(trifluoromethoxy)phenyl)isoxazol-5(4*H*)-one (1g)

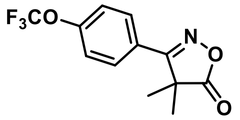

1g

Synthesized following **General Procedure C** using: 1-(4-(trifluoromethoxy)phenyl)ethan-1-one (1.60 mL, 10.00 mmol, 1.00 equiv.), sodium hydride (1.12 g, 28.00 mmol, 2.80 equiv.), dimethyl carbonate (1.60 mL, 20.00 mmol, 2.00 equiv.), and 1,4-dioxane (10 mL, 0.67 M) for step one, potassium carbonate (5.53 g, 40.00 mmol, 4.00 equiv.), methyl iodide (2.50 mL, 40.00 mmol, 4.00 equiv.), and DMF (28 mL, 0.36 M) for step two yield the methylated β-ketoester intermediate as a yellow oil. For the third step, $NH_2OH\cdot HCl$ (1.36 g, 19.50 mmol, 2.50 equiv.), NaOAc (1.25 g, 15.60 mmol, 2.00 equiv.), EtOH (11.80 mL, 0.66 M) and H_2O (2.30 mL, 3.33 M) were used. Purified by flash column chromatography (SiO_2; 95:5 pentane/EtOAc) to afford **1g** (0.87 g, 3.07 mmol, 31%) as a white solid over three steps.

NMR Spectroscopy (*see spectra*)

^{1}H NMR (400 MHz, $CDCl_3$): δ_H 7.87 – 7.78 (m, 1H), 7.38 – 7.29 (m, 1H), 1.61 (s, 3H) ppm;

^{19}F NMR (377 MHz, $CDCl_3$): δ_F -57.71 ppm;

^{13}C NMR (101 MHz, $CDCl_3$): δ_C 181.5, 168.6, 151.6 (d, J = 1.9 Hz), 128.8, 126.2, 121.5 (q, J = 1.2 Hz), 120.5 (q, J = 258.9 Hz), 45.7, 23.1 ppm.

HRMS (ESI^+): m/z calc'd for $C_{12}H_{10}F_3NO_3Na$ $[M+Na]^+$: 296.0505, found: 296.0504.

4,4-Dimethyl-3-(p-tolyl)isoxazol-5(4*H*)-one (1h)

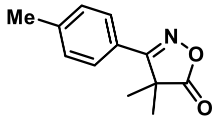

1h

Synthesized following **General Procedure C** using: 1-(p-tolyl)ethan-1-one (1.34 mL, 10.00 mmol, 1.00 equiv.), sodium hydride (1.12 g, 28.00 mmol, 2.80 equiv.), dimethyl carbonate (1.60 mL, 20.00 mmol, 2.00 equiv.), and 1,4-dioxane (10 mL, 0.67 M) for step one, potassium carbonate (5.53 g, 40.00 mmol, 4.00 equiv.), methyl iodide (2.50 mL, 40.00 mmol, 4.00 equiv.), and DMF (28 mL, 0.36 M) for step two yield the methylated β-ketoester intermediate as a yellow oil. For the third step, $NH_2OH{\cdot}HCl$ (1.31 g, 19.50 mmol, 2.50 equiv.), NaOAc (1.20 g, 15.60 mmol, 2.00 equiv.), EtOH (11.4 mL, 0.66 M) and H_2O (2.3 mL, 3.33 M) were used. Purified by flash column chromatography (SiO_2; 90:10 pentane/EtOAc) to afford **1h** (0.87 g, 4.09 mmol, 41%) as a white solid over three steps.

NMR Spectroscopy (*see spectra*)

^{1}H NMR (400 MHz, $CDCl_3$): δ_H 7.69 – 7.62 (m, 2H), 7.32 – 7.26 (m, 2H), 2.42 (s, 3H), 1.60 (s, 6H) ppm;

^{13}C NMR (101 MHz, $CDCl_3$): δ_C 182.1, 169.7, 142.4, 130.0, 127.0, 124.9, 45.9, 23.2, 21.7 ppm.

HRMS (ESI^+): m/z calc'd for $C_{12}H_{13}NO_2Na$ $[M+Na]^+$: 226.0839, found: 226.0838.

3-(4-Methoxyphenyl)-4,4-dimethylisoxazol-5(4*H*)-one (1i)

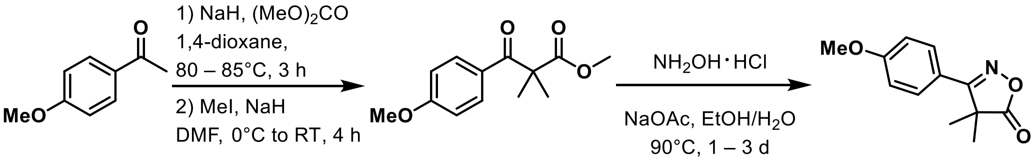

1i

Synthesized following **General Procedure C**, but employing a slightly modified procedure for step two. For the first step, 1-(4-methoxyphenyl)ethan-1-one (1.34 mL, 10.00 mmol, 1.00 equiv.), sodium hydride (1.12 g, 28.00 mmol, 2.80 equiv.), dimethyl carbonate (1.60 mL, 20.00 mmol, 2.00 equiv.), and 1,4-dioxane (10 mL, 0.67 M) were used. For the next step, the crude material was dissolved in dry DMF (28 mL, 0.36 M) in a 100 mL Schlenk tube, which was evacuated and refilled with argon three times beforehand. Then, NaH (60% dispersion in mineral oil, 0.52 g, 13.00 mmol, 1.30 equiv.) was added at 0°C and the reaction was stirred at room temperature for 30 min. Then, MeI (0.81 mL, 13.00 mmol, 1.30 equiv.) was added at 0°C and the reaction was stirred for 1 h at room temperature. Then, another porthion of NaH (60% dispersion in mineral oil, 0.52 g, 13.00 mmol, 1.30 equiv.) was added at 0°C and it was stirred at room temperature for 30 min, until a second portion of MeI (0.81 mL, 13.00 mmol, 1.30 equiv.) was added at 0°C. Then, it was stirred at room temperature for 1.5 h upon completion and the reaction was quenched with sat. aq. NH_4Cl-solution (20 mL). The aqueous layer was extracted with EtOAc (3x 20 mL), and the combined organic layers with water (3x 20 mL) and brine (1x 10 mL). The organic phase was dried over $MgSO_4$, filtered and the solvent was evaporated under reduced

pressure. The crude material was filtered over a small silica plug and directly used for the next step. According to the general procedure, for step 3 NH_2OH· HCl (0.87 g, 12.50 mmol, 2.50 equiv.), NaOAc (0.80 g, 10.00 mmol, 2.00 equiv.), EtOH (7.60 mL, 0.66 M) and H_2O (1.50 mL, 3.33 M) were used. Purified by flash column chromatography (SiO_2; 90:10 pentane/EtOAc) to afford **1i** (0.66 g, 2.99 mmol, 30%) as a white solid over three steps.

NMR Spectroscopy (*see spectra*)

^{1}H NMR (400 MHz, $CDCl_3$): δ_H 7.77 – 7.69 (m, 2H), 7.02 – 6.94 (m, 2H), 3.87 (s, 2H), 1.60 (s, 6H) ppm;

^{13}C NMR (101 MHz, $CDCl_3$): δ_C 182.2, 169.3, 162.4, 128.7, 120.0, 114.7, 55.6, 45.8, 23.3 ppm.

HRMS (ESI$^+$): m/z calc'd for $C_{12}H_{13}NO_3Na$ $[M+Na]^+$: 242.0788, found: 242.0786.

3-(4-Chlorophenyl)-4,4-dimethylisoxazol-5(4*H*)-one (1j)

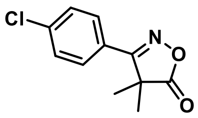

1j

Synthesized following General Procedure C using: 1-(4-chlorophenyl)ethan-1-one (1.90 mL, 10.00 mmol, 1.00 equiv.), sodium hydride (1.12 g, 28.00 mmol, 2.80 equiv.), dimethyl carbonate (1.60 mL, 20.00 mmol, 2.00 equiv.), and 1,4-dioxane (10 mL, 0.67 M) for step one, potassium carbonate (5.53 g, 40.00 mmol, 4.00 equiv.), methyl iodide (2.50 mL, 40.00 mmol, 4.00 equiv.), and DMF (28 mL, 0.36 M) for step two yield the methylated β-ketoester intermediate as a yellow oil. For the third step, NH_2OH·HCl (1.42 g, 20.50 mmol, 2.50 equiv.), NaOAc (1.34 g, 16.40 mmol, 2.00 equiv.), EtOH (12.40 mL, 0.66 M) and H_2O (2.50 mL, 3.33 M) were used. Purified by flash column chromatography (SiO_2; 95:5 pentane/EtOAc) to afford **1j** (0.83 g, 4.09 mmol, 41%) as a white solid over three steps.

NMR Spectroscopy (*see spectra*):

^{1}H NMR (400 MHz, $CDCl_3$): δ_H 7.75 – 7.67 (m, 2H), 7.51 – 7.43 (m, 2H), 1.60 (s, 6H) ppm;

^{13}C NMR (101 MHz, $CDCl_3$): δ_C 181.6, 168.9, 138.2, 129.7, 128.3, 126.2, 45.7, 23.1 ppm.

HRMS (ESI$^+$): m/z calc'd for $C_{11}H_{10}NO_2ClNa$ $[M+Na]^+$: 246.0292, found: 246.0292.

Ethyl 4-benzoyltetrahydro-2*H*-pyran-4-carboxylate (S1)

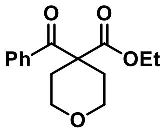

S1

Synthesized following General Procedure B Step 1 using ethyl 3-oxo-3-phenylpropanoate (870 µL, 5.00 mmol, 1.00 equiv.) and bis(2-bromoethyl) ether (690 µL, 5.50 mmol, 1.10 equiv.). The crude reaction mixture was

purified by flash column chromatography (SiO_2; 95:5 pentane/EtOAc) to afford **S1** as a colorless oil (0.80 g, 3.00 mmol, 61%).

TLC: R_f = 0.34 (95:5 pentane/EtOAc).

NMR Spectroscopy (*see spectra*):

^{1}H NMR (599 MHz, $CDCl_3$): δ_H 7.87 – 7.79 (m, 2H), 7.57 – 7.48 (m, 1H), 7.42 (ddt, *J* = 8.2, 6.8, 1.1 Hz, 2H), 4.16 (q, *J* = 7.1 Hz, 2H), 3.85 – 3.75 (m, 2H), 3.75 – 3.65 (m, 2H), 2.25 (t, *J* = 5.5 Hz, 4H), 1.10 (t, *J* = 7.1 Hz, 3H) ppm;

^{13}C NMR (151 MHz, $CDCl_3$): δ_C 196.9, 173.1, 135.7, 132.9, 128.7, 128.7, 64.4, 61.8, 55.7, 32.1, 14.0 ppm.

HRMS (ESI$^+$): m/z calc'd for $C_{15}H_{18}O_4Na$ $[M+Na]^+$: 285.1103, found: 285.1097.

4-Phenyl-2,8-dioxa-3-azaspiro[4.5]dec-3-en-1-one (1k)

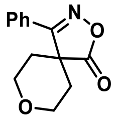

1k

Synthesized following **General Procedure B** Step 2 **S1** (0.08 g, 3.00 mmol, 1.00 equiv.). The crude reaction mixture was purified by flash column chromatography (SiO_2; 88:12 pentane/EtOAc) to afford **1k** as a white solid (0.33 g, 1.40 mmol, 47%).

TLC: R_f = 0.27 (85:15 pentane/EtOAc).

NMR Spectroscopy (*see spectra*):

^{1}H NMR (599 MHz, $CDCl_3$): δ_H 7.80 – 7.73 (m, 2H), 7.59 – 7.44 (m, 3H), 4.25 (td, *J* = 12.2, 2.3 Hz, 2H), 3.88 (ddt, *J* = 12.3, 5.4, 1.1 Hz, 2H), 2.42 (ddd, *J* = 14.2, 12.3, 5.2 Hz, 2H), 1.80 (ddt, *J* = 14.3, 2.5, 1.5 Hz, 2H) ppm;

^{13}C NMR (151 MHz, $CDCl_3$): δ_C 179.7, 168.3, 131.7, 129.3, 127.6, 127.3, 61.1, 45.8, 29.3 ppm:

HRMS (ESI$^+$): m/z calc'd for $C_{13}H_{13}NO_3Na$ $[M+Na]^+$: 254.0793, found: 254.0787.

Ethyl 1-benzoylcyclohexane-1-carboxylate (S2)

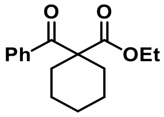

S2

Synthesized following **General Procedure B** Step 1 using ethyl 3-oxo-3-phenylpropanoate (870 µL, 5.00 mmol, 1.00 equiv.) and 1,5-dibromopentane (750 µL, 5.50 mmol, 1.10 equiv.). The crude reaction mixture was purified by flash column chromatography (SiO_2; 9:1 pentane/EtOAc) to afford **S2** as a colorless oil (0.65 g, 2.50 mmol, 50%).

TLC: R_f = 0.78 (9:1 pentane/EtOAc).

NMR Spectroscopy (*see spectra*):

^{1}H NMR (599 MHz, $CDCl_3$): δ_H 7.83 – 7.75 (m, 2H), 7.54 – 7.45 (m, 1H), 7.44 – 7.35 (m, 2H), 4.12 (q, *J* = 7.1 Hz, 2H), 2.10 (t, *J* = 6.0 Hz, 4H), 1.70 – 1.37 (m, 6H), 1.08 (t, *J* = 7.1 Hz, 3H) ppm;

^{13}C NMR (151 MHz, $CDCl_3$): δ_C 198.7, 174.0, 136.5, 132.5, 128.5, 128.5, 61.4, 58.3, 32.5, 25.6, 22.5, 14.0 ppm.

HRMS (ESI$^+$): m/z calc'd for $C_{16}H_{20}O_3Na$ $[M+Na]^+$: 283.1310, found: 283.1303.

4-Phenyl-2-oxa-3-azaspiro[4.5]dec-3-en-1-one (1l)

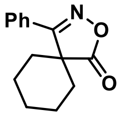

1l

Synthesized following **General Procedure B** Step 2 **S2** (0.06 g, 2.30 mmol, 1.00 equiv.). The crude reaction mixture was purified by flash column chromatography (SiO_2; 98:2 pentane/EtOAc) to afford **1l** as a white solid (0.24 g, 1.10 mmol, 46%).

TLC: R_f = 0.33 (98:2 pentane/EtOAc).

NMR Spectroscopy (*see spectra*):

^{1}H NMR (599 MHz, $CDCl_3$): δ_H 7.71 – 7.64 (m, 2H), 7.57 – 7.43 (m, 3H), 2.13 (qt, *J* = 12.8, 4.0 Hz, 2H), 2.04 – 1.80 (m, 5H), 1.69 – 1.58 (m, 2H), 1.28 (qt, *J* = 13.5, 3.8 Hz, 1H) ppm;

^{13}C NMR (151 MHz, $CDCl_3$): δ_C 179.9, 170.2, 131.2, 129.1, 128.1, 127.8, 48.5, 30.3, 24.7, 19.0 ppm.

HRMS (ESI$^+$): m/z calc'd for $C_{14}H_{15}NO_2Na$ $[M+Na]^+$: 252.1000, found: 252.0994.

2.2. Synthesis of 2,2-Dimethyl-3-phenyl-2*H*-azirine (4a)

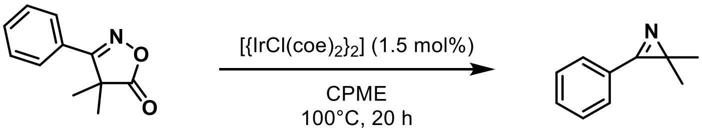

1a **4a**

Following a modified literature procedure, an oven-dried 50 mL Schlenk tube equipped with a stirring bar was transferred to a glovebox, where $[\{IrCl(coe)_2\}_2]$ (26.90 mg, 0.03 mmol, 0.015 equiv.) was added. The tube was sealed and removed from the glovebox again. Then, **1a** (0.38 g, 2.00 mmol, 1.00 equiv.) and cyclopentyl methyl ether (CPME) (20.00 mL, 0.10 M) were added under a positive argon pressure and the tube was sealed again. The reaction was stirred at 100°C for 20 h. Then, the solvent was removed under reduced pressure and the crude product purified using flash column chromatography (SiO_2; 95:5 pentane/EtOAc) to yield the product **4a**

as a pale brown oil (0.25 g, 1.74 mmol, 87%). The spectral data matches those reported in the literature.[6,7]

NMR spectroscopy (*see spectra*):

^{1}H NMR (400 MHz, $CDCl_3$) δ_H 7.85 – 7.76 (m, 1H), 7.61 – 7.47 (m, 2H), 1.42 (s, 3H) ppm;
^{13}C NMR (101 MHz, $CDCl_3$) δ_C 177.9, 132.7, 129.2, 129.1, 126.1, 34.0, 24.8 ppm.

2.3. Synthesis of Electrophilic Coupling Partners

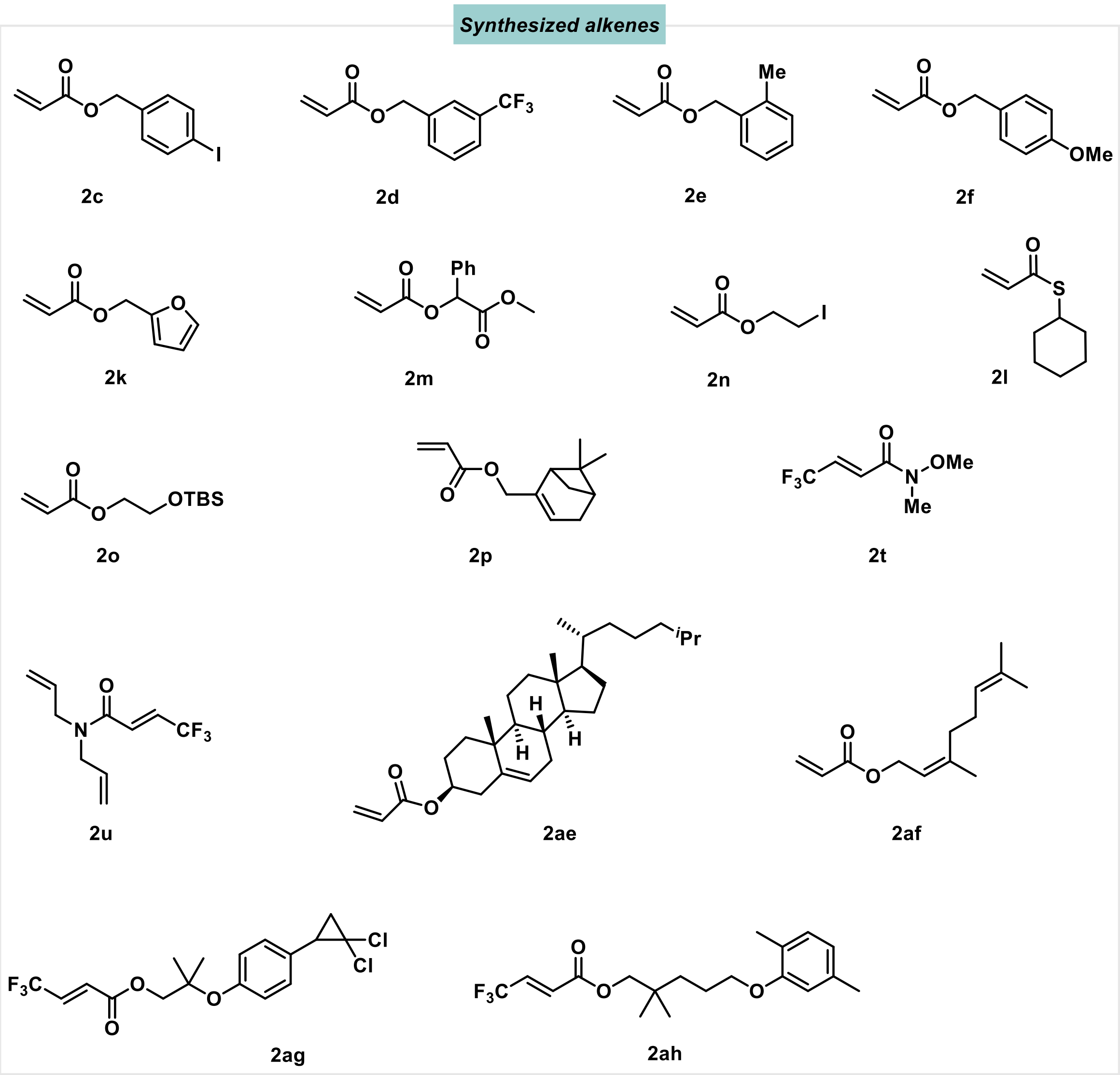

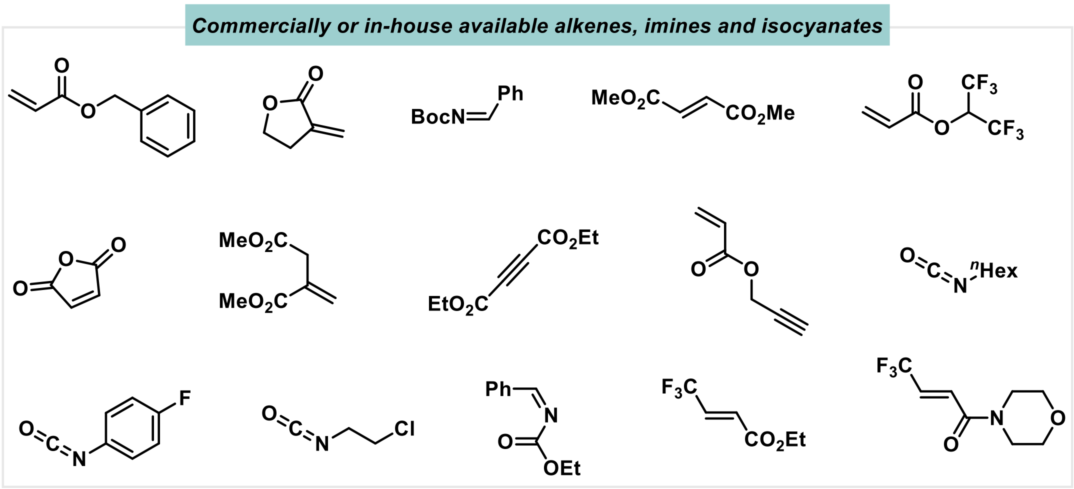

5-(2,5-Dimethylphenoxy)-2,2-dimethylpentan-1-ol (S3)

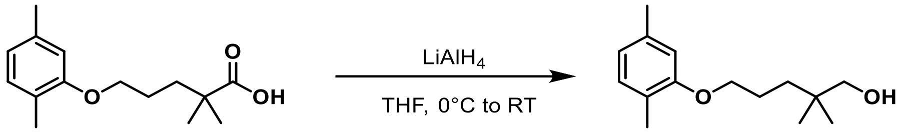

S3

The compound was synthesized following the literature procedure, the solution of gemfibrozil (1.25 g, 5.00 mmol,1.00 equiv.) in 20 mL dry THF was added dropwise to the suspension of $LiAlH_4$ (0.95 g, 25.00 mmol, 5.00 equiv.) in 40 mL dry THF at 0°C. The mixture was stirred at room temperature for 4 hours. Then the mixture was quenched by slow addition of 1 M HCl solution and extracted with ethyl acetate. The organic layer was concentrated under reduced pressure and the residue was purified by column chromatography (5:1 pentane/EtOAc) to afford the alcohol product **S3** as a pale yellow liquid (1.06 g, 2.02 mmol, 90%).[8]

NMR Spectroscopy (*see spectra*):

^{1}H NMR (400 MHz, $CDCl_3$) δ_H 7.05 – 7.00 (m, 1H), 6.72 – 6.63 (m, 2H), 3.95 (t, *J* = 6.4 Hz, 2H), 3.38 (s, 2H), 2.33 (s, 3H), 2.21 (s, 3H), 1.86 – 1.73 (m, 2H), 1.49 – 1.38 (m, 2H), 0.94 (s, 6H) ppm;

^{13}C NMR (101 MHz, $CDCl_3$) δ_C 157.1, 136.5, 130.3, 123.6, 120.7, 112.1, 71.8, 68.6, 34.9, 34.9, 24.2, 23.9, 21.4, 15.8 ppm.

HRMS (ESI$^+$): m/z calc'd for $C_{15}H_{24}O_2Na$ $[M+Na]^+$: 259.1669, found: 259.1667.

2-((*tert*-Butyldimethylsilyl)oxy)ethan-1-ol (S4)

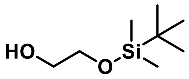
S4

The compound was synthesized following a literature procedure using ethylene glycol (11 mL, 186.00 mmol, 9.30 equiv.), *tert*-butyldimethylsilylchloride (3.00 g, 20.00 mmol, 1.00 equiv.) and pyridine (11 mL). The crude reaction mixture was purified by flash column chromatography (SiO_2; 95:5 pentane/EtOAc) to afford the product **S4** as a colorless oil (1.18 g, 6.70 mmol, 34%). Spectral data are in agreement with reported values.[9]

NMR Spectroscopy (<u>see spectra</u>):

^{1}H NMR (400 MHz, $CDCl_3$): δ_H 3.74 – 3.69 (m, 2H), 3.68 – 3.60 (m, 2H), 2.05 (td, *J* = 6.3, 1.4 Hz, 1H), 0.91 (s, 9H), 0.08 (s, 6H) ppm

2-(4-(2,2-Dichlorocyclopropyl)phenoxy)-2-methylpropan-1-ol (S5)

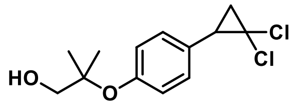

S5

Under argon atmosphere, ciprofibrate (2.90 g, 10.00 mmol, 1.00 equiv.) was dissolved in dry THF (60 mL) and cooled to 0°C. BH_3·THF solution (1 M in THF, 30 mL, 3.00 equiv.) was added dropwise. The reaction mixture was stirred at 50°C for 3 h. The reaction mixture was cooled to 0°C and carefully quenched with water and sat. aq. K_2CO_3-solution. The phases were separated and the aqueous layer was extracted with EtOAc (3x). The combined organic layers were dried over $MgSO_4$ and concentrated. The crude reaction mixture was purified by flash column chromatography (SiO_2; 95:5 to 9:1 pentane/EtOAc) to afford **S5** as a colorless oil (2.20 g, 8.10 mmol, 81%).

TLC: R_f = 0.13 (95:5 pentane/EtOAc).

NMR Spectroscopy (<u>see spectra</u>):

^{1}H NMR (400 MHz, $CDCl_3$): δ_H 7.20 – 7.12 (m, 2H), 7.01 – 6.93 (m, 2H), 3.59 (d, *J* = 6.5 Hz, 2H), 2.86 (dd, *J* = 10.7, 8.4 Hz, 1H), 2.20 (t, *J* = 6.5 Hz, 1H), 1.96 (dd, *J* = 10.7, 7.4 Hz, 1H), 1.81 (dd, *J* = 8.4, 7.4 Hz 1H), 1.28 (s, 6H) ppm;

^{13}C NMR (101 MHz, $CDCl_3$): δ_C 154.2, 130.1, 129.7, 123.7, 81.0, 70.5, 60.9, 35.1, 26.0, 23.2 ppm.

HRMS (ESI$^+$): m/z calc'd for $C_{13}H_{16}Cl_2O_2Na$ $[M+Na]^+$: 297.0425, found: 297.0418.

2.4. Synthesis of Acryl Esters

2.4.1. General Procedure D: Ester Synthesis Starting from Carboxylic Acids

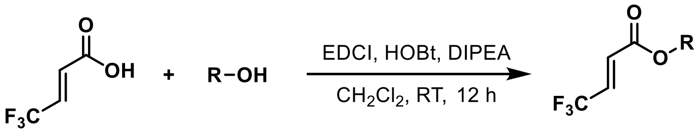

Following a reported procedure, a mixture of enoic acid (2.00 g, 8.46 mmol, 1.00 equiv.), alcohol (1.30 g, 9.30 mmol, 1.10 equiv.), EDCI (2.40 g, 12.69 mmol, 1.50 equiv.), HOBt (1.70 g, 12.69 mmol, 1.50 equiv.), and DIPEA (2.95 mL, 16.92 mmol, 2.00 equiv.) in CH_2Cl_2 (0.15 M) was stirred for 12 h at room temperature. After completion, H_2O (60 mL) was added to the reaction mixture, then the mixture was extracted with CH_2Cl_2 (3x 40 mL). This

was dried over $MgSO_4$, and the solvent was removed under reduced pressure. The residue was purified by flash chromatography with the given ratio of EtOAc/pentane to give the corresponding products.[10]

2.4.2 General Procedure E: Ester Synthesis Starting from Acyl Chlorides

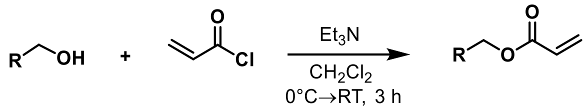

Following a reported procedure,[11] to a stirred solution of alcohol (25.00 mmol, 1.00 equiv.) and triethyl amine (5.2 mL, 37.50 mmol, 1.50 equiv.) in dry dichloromethane (30 mL) was added dropwise acryloyl chloride (2.1 mL, 26.25 mmol, 1.05 equiv.) at 0°C under argon atmosphere. The mixture was kept at 0°C for 30 min and stirred at room temperature for 2 h. Once the starting material was consumed (monitored by TLC), the reaction mixture was diluted with distilled water, CH_2Cl_2 and brine. The organic layer was dried over Na_2SO_4, and concentrated under reduced pressure. The residue was purified by flash chromatography with given ratio of EtOAc/pentane to give the corresponding products.

4-Iodobenzyl acrylate (2c)

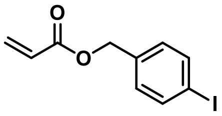

2c

The title compound was synthesized following **General Procedure E** using (4-iodophenyl)methanol (5.85 g, 25.00 mmol, 1.00 equiv.) and acryloyl chloride (2.1 mL, 26.25 mmol, 1.05 equiv.). The crude reaction mixture was purified by flash column chromatography (SiO_2; 98:2 pentane/EtOAc) to afford **2c** as colorless oil (4.90 g, 17.00 mmol, 68%). The obtained data is consistent with the values reported in the literature.[11]

TLC: R_f = 0.80 (90:10 pentane/EtOAc).

NMR Spectroscopy (*see spectra*):

^{1}H NMR (400 MHz, $CDCl_3$): δ_H 7.73 – 7.62 (m, 2H), 7.11 (d, *J* = 6.5 Hz, 2H), 6.43 (dd, *J* = 17.3, 1.4 Hz, 1H), 6.14 (dd, *J* = 17.3, 10.4 Hz, 1H), 5.85 (dd, *J* = 10.4, 1.4 Hz, 1H), 5.12 (s, 2H) ppm.

HRMS (ESI$^+$): m/z calc'd for $C_{10}H_9IO_2Na$ $[M+Na]^+$: 310.9540, found: 310.9539.

4-(Trifluoromethyl)benzyl acrylate (2d)

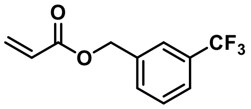

2d

The title compound was synthesized as colorless oil following **General Procedure E** using (3-(trifluoromethyl)phenyl)methanol (4.40 g, 25.00 mmol, 1.00 equiv.) and acryloyl chloride (2.10 mL, 26.25 mmol,

1.05 equiv.). The crude reaction mixture was purified by flash column chromatography (SiO_2; 98:2 pentane/EtOAc) to afford **2d** as colorless oil (4.72 g, 20.50 mmol, 82%).

TLC: R_f = 0.85 (90:10 pentane/EtOAc).

NMR Spectroscopy (*see spectra*):

^{1}H NMR (400 MHz, $CDCl_3$): δ_H 7.67 – 7.53 (m, 3H), 7.53 – 7.47 (m, 1H), 6.47 (dd, *J* = 17.4, 1.4 Hz, 1H), 6.18 (dd, *J* = 17.3, 10.4 Hz, 1H), 5.88 (dd, *J* = 10.4, 1.4 Hz, 1H), 5.25 (s, 2H) ppm;
^{13}C NMR (101 MHz, $CDCl_3$): δ_C 165.9, 137.0, 131.7, 131.5 (d, *J* = 1.4 Hz), 129.2, 128.1, 125.2 (q, *J* = 3.8 Hz), 124.9 (q, *J* = 3.8 Hz), 65.5 ppm;
^{19}F NMR (376 MHz, $CDCl_3$): δ_F -62.72 (d, *J* = 1.0 Hz) ppm.

HRMS (ESI^+): m/z calc'd for $C_{11}H_9O_2F_3Na$ $[M+Na]^+$: 253.0447, found: 253.0446.

2-Methylbenzyl acrylate (2e)

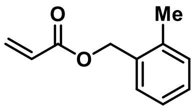

2e

The title compound was synthesized following **General Procedure E** using o-tolylmethanol (3.05 g, 25.00 mmol, 1.00 equiv.) and acryloyl chloride (2.10 mL, 26.25 mmol, 1.05 equiv.). The crude reaction mixture was purified by flash column chromatography (SiO_2; 98:2 pentane/EtOAc) to afford **2e** as colorless oil (3.30g, 18.75 mmol, 75%).

TLC: R_f = 0.85 (90:10 pentane/EtOAc).

NMR Spectroscopy (*see spectra*):

^{1}H NMR (400 MHz, $CDCl_3$): δ_H 7.42 (dd, *J* = 7.8, 1.7 Hz, 1H), 7.35 – 7.24 (m, 3H), 6.51 (dd, *J* = 17.4, 1.5 Hz, 1H), 6.23 (dd, *J* = 17.3, 10.4 Hz, 1H), 5.90 (dd, *J* = 10.4, 1.5 Hz, 1H), 5.29 (s, 2H), 2.43 (s, 3H) ppm;

^{13}C NMR (101 MHz, $CDCl_3$): δ_C 166.1, 137.1, 133.9, 131.1, 130.4, 129.3, 128.6, 128.4, 126.1, 64.8, 19.0 ppm.

HRMS (ESI^+): m/z calc'd for $C_{11}H_{12}O_2Na$ $[M+Na]^+$: 199.0730, found: 199.0729.

4-Methoxybenzyl acrylate (2f)

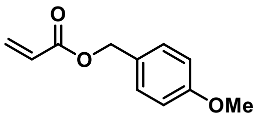

2f

The title compound was synthesized following **General Procedure E** using (4-methoxyphenyl)methanol (3.45 g, 25.00 mmol, 1.00 equiv.) and acryloyl chloride (2.10 mL, 26.25 mmol, 1.05 equiv.). The crude reaction mixture was purified by flash column chromatography (SiO_2; 98:2 pentane/EtOAc) to afford **2f** as colorless oil (4.08g, 21.25 mmol, 85%).

TLC: R_f = 0.80 (95:5 pentane/EtOAc).

NMR Spectroscopy (*see spectra*):

^{1}H NMR (400 MHz, $CDCl_3$): δ_H 7.37 – 7.28 (m, 2H), 6.94 – 6.86 (m, 2H), 6.43 (dd, *J* = 17.4, 1.5 Hz, 1H), 6.15 (dd, *J* = 17.3, 10.4 Hz, 1H), 5.82 (dd, *J* = 10.4, 1.5 Hz, 1H), 5.14 (s, 2H), 3.80 (s, 3H) ppm;

^{13}C NMR (101 MHz, $CDCl_3$): δ_C 166.2, 159.8, 131.0, 130.2, 128.5, 128.1, 114.0, 66.2, 55.3 ppm.

HRMS (ESI$^+$): m/z calc'd for $C_{11}H_{12}O_3Na$ $[M+Na]^+$: 215.0679, found: 215.0677.

Furan-2-ylmethyl acrylate (2k)

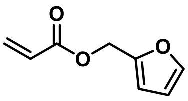

2k

The title compound was synthesized following General Procedure E using furan-2-ylmethanol (2.45 g, 25.00 mmol, 1.00 equiv.) and acryloyl chloride (2.10 mL, 26.25 mmol, 1.05 equiv.). The crude reaction mixture was purified by flash column chromatography (SiO_2; 98:2 pentane/EtOAc) to afford **2k** as colorless oil (3.31g, 21.75 mmol, 87%).

TLC: R_f = 0.85 (90:10 pentane/EtOAc).

NMR Spectroscopy (*see spectra*):

^{1}H NMR (400 MHz, $CDCl_3$): δ_H 7.42 (dd, *J* = 1.8, 0.9 Hz, 1H), 6.45 – 6.40 (m, 2H), 6.36 (dd, *J* = 3.3, 1.9 Hz, 1H), 6.17 – 6.08 (m, 1H), 5.83 (dd, *J* = 10.5, 1.4 Hz, 1H), 5.14 (s, 2H) ppm;

^{13}C NMR (101 MHz, $CDCl_3$): δ_C 165.8, 149.5, 143.4, 131.4, 128.2, 110.8, 110.7, 58.2 ppm.

HRMS (ESI$^+$): m/z calc'd for $C_8H_8O_3Na$ $[M+Na]^+$: 175.0366, found: 175.0364.

S-Cyclohexyl prop-2-enethioate (2l)

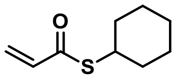

2l

The compound was synthesized following a literature procedure using acryloyl chloride (490 µL, 6.00 mmol, 1.00 equiv.), cyclohexanethiol (880 µL, 7.20 mmol, 1.20 equiv.), triethylamine (1.70 mL, 12.00 mmol, 2.00 equiv.) and dichloromethane (12 mL). The crude reaction mixture was purified by flash column chromatography (SiO_2; 9:1 pentane/CH_2Cl_2) to afford the product as a colorless oil (0.04 g, 2.60 mmol, 43%).

NMR Spectroscopy (*see spectra*):

^{1}H NMR (400 MHz, $CDCl_3$): δ_H 6.39 – 6.21 (m, 2H), 5.63 (dd, *J* = 9.5, 1.9 Hz, 1H), 3.67 – 3.55 (m, 1H), 2.02 – 1.87 (m, 2H), 1.77 – 1.65 (m, 2H), 1.65 – 1.52 (m, 1H), 1.52 – 1.37 (m, 4H), 1.29 (dddd, *J* = 14.6, 12.8, 6.7, 3.4

Hz, 1H) ppm;

^{13}C NMR (101 MHz, $CDCl_3$): δ_C 190.4, 135.6, 125.9, 42.4, 33.2, 26.1, 25.7 ppm.

HRMS (ESI$^+$): m/z calc'd for $C_9H_{14}OSNa$ $[M+Na]^+$: 193.0663, found: 193.0658.

2-Methoxy-2-oxo-1-phenylethyl acrylate (2m)

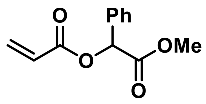

2m

The title compound was synthesized as a colorless oil following **General Procedure E** using methyl 2-hydroxy-2-phenylacetate (4.15 g, 25.00 mmol, 1.00 equiv.) and acryloyl chloride (2.10 mL, 26.25 mmol, 1.05 equiv.). The crude reaction mixture was purified by flash column chromatography (SiO_2; 98:2 pentane/EtOAc) to afford **2m** as pale yellow oil (3.85 g, 17.50 mmol, 70%).

TLC: R_f = 0.80 (90:10 pentane/EtOAc).

NMR Spectroscopy (*see spectra*):

^{1}H NMR (400 MHz, $CDCl_3$): δ_H 7.45 – 7.39 (m, 2H), 7.35 – 7.29 (m, 3H), 6.45 (dd, J = 17.3, 1.3 Hz, 1H), 6.17 (dd, J = 17.4, 10.5 Hz, 1H), 5.94 (s, 1H), 5.85 (dd, J = 10.4, 1.3 Hz, 1H), 3.65 (s, 3H) ppm;

^{13}C NMR (101 MHz, $CDCl_3$): δ_C 169.3, 165.4, 133.9, 132.4, 129.4, 128.9, 127.8, 127.6, 74.6, 52.7 pp

HRMS (ESI$^+$): m/z calc'd for $C_{12}H_{12}O_4Na$ $[M+Na]^+$: 243.0628, found: 243.0629.

2-Iodoethyl acrylate (2n)

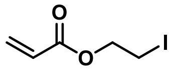

2n

The title compound was synthesized following **General Procedure E** using 2-iodoethan-1-ol (5.85 g, 25.00 mmol, 1.00 equiv.) and acryloyl chloride (2.1 mL, 26.25 mmol, 1.05 equiv.). The crude reaction mixture was purified by flash column chromatography (SiO_2; 98:2 pentane/EtOAc) to afford **2n** as colorless oil (3.84 g, 17.00 mmol, 68%).

TLC: R_f = 0.90 (90:10 pentane/EtOAc).

NMR Spectroscopy (*see spectra*):

^{1}H NMR (400 MHz, $CDCl_3$): δ_H 6.44 (dd, J = 17.3, 1.4 Hz, 1H), 6.12 (dd, J = 17.3, 10.4 Hz, 1H), 5.87 (dd, J = 10.5, 1.4 Hz, 1H), 4.40 (t, J = 6.9 Hz, 2H), 3.32 (t, J = 6.9 Hz, 2H) ppm;
^{13}C NMR (101 MHz, $CDCl_3$): δ_C 165.5, 131.7, 128.0, 64.7 ppm.

HRMS (ESI$^+$): m/z calc'd for $C_5H_7O_2INa$ $[M+Na]^+$: 248.9383, found: 248.9384.

2-((*tert*-Butyldimethylsilyl)oxy)ethan-1-ol (2o)

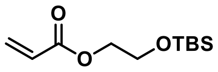

2o

The compound was synthesized following a literature procedure using acryloyl chloride (450 µL, 5.50 mmol, 1.00 equiv.), **S4** (1.20 g, 6.60 mmol, 1.20 equiv.), triethylamine (1.50 mL, 11.00 mmol, 2.00 equiv.) and dichloromethane (11 mL). The crude reaction mixture was purified by flash column chromatography (SiO_2; 95:5 pentane/EtOAc) to afford the product as a colorless oil (1.15 g, 5.00 mmol, 90%). Spectral data are in agreement with reported values.[12,13]

NMR Spectroscopy (*see spectra*):

^{1}H NMR (400 MHz, $CDCl_3$): δ_H 6.42 (dd, *J* = 17.3, 1.5 Hz, 1H), 6.14 (dd, *J* = 17.3, 10.4 Hz, 1H), 5.83 (dd, *J* = 10.4, 1.5 Hz, 1H), 4.27 – 4.20 (m, 2H), 3.89 – 3.82 (m, 2H), 0.89 (s, 9H), 0.07 (s, 6H) ppm;

^{13}C NMR (101 MHz, $CDCl_3$): δ_C 166.3, 130.9, 128.6, 65.9, 61.4, 26.0, 18.5, -5.2 ppm.

(6,6-Dimethylbicyclo[3.1.1]hept-2-en-2-yl)methyl acrylate (2p)

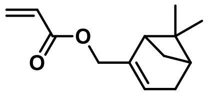

2p

The title compound was synthesized as a colorless oil following **General Procedure E** using (6,6-dimethylbicyclo[3.1.1]hept-2-en-2-yl)methanol (3.80 g, 25.00 mmol, 1.00 equiv.) and acryloyl chloride (2.10 mL, 26.25 mmol, 1.05 equiv.). The crude reaction mixture was purified by flash column chromatography (SiO_2; 98:2 pentane/EtOAc) to afford **2p** as colorless oil (3.10 g, 15.00 mmol, 60%). The obtained data is consistent with the values reported in the literature.[14]

TLC: R_f = 0.70 (90:10 pentane/EtOAc).

NMR Spectroscopy (*see spectra*):

^{1}H NMR (400 MHz, $CDCl_3$): δ_H 6.35 (dd, *J* = 17.3, 1.6 Hz, 1H), 6.08 (dd, *J* = 17.3, 10.4 Hz, 1H), 5.77 (dd, *J* = 10.4, 1.6 Hz, 1H), 5.54 (tt, *J* = 3.0, 1.5 Hz, 1H), 4.56 – 4.43 (m, 2H), 2.37 (dt, *J* = 8.7, 5.6 Hz, 1H), 2.33 – 2.16 (m, 2H), 2.13 – 2.03 (m, 2H), 1.25 (s, 3H), 1.15 (d, *J* = 8.7 Hz, 1H), 0.79 (s, 3H) ppm;

^{13}C NMR (101 MHz, $CDCl_3$): δ_C 166.4, 143.3, 130.8, 129.0, 121.9, 67.4, 44.0, 41.1, 38.4, 31.8, 31.6, 26.5, 21.4 ppm.

(*E*)-4,4,4-Trifluoro-*N*-methoxy-*N*-methylbut-2-enamide (2t)

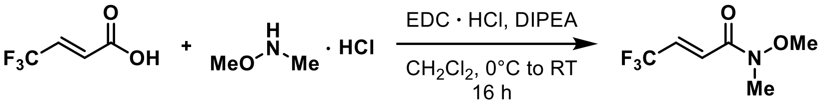

2t

Following a modified literature procedure, an oven-dried 250 mL roundbottom-Schlenk-flask equipped with a stir bar was evacuated and backfilled with argon three times. Then, (*E*)-4,4,4-trifluorobut-2-enoic acid (5.40 g, 38.55 mmol, 1.00 equiv.) and dry CH_2Cl_2 (100 mL, 0.39 M) were added and the mixture was cooled down to 0°C. Then, 1-ethyl(3-(3-dimethylamino)propyl)-carbodiimide hydrochloride (EDC • HCl) (9.01 g, 47.04 mmol, 1.64 equiv.), *N*,*O*-dimethylhydroxylamine hydrochloride (4.89 g, 50.13 mmol, 1.30 equiv.) and DIPEA (10.70 mL, 63.21 mmol, 1.64 equiv.) were added sequentially at 0 °C under stirring and the reaction mixture was stirred at that temperature for 5 min, before letting warm up to room temperature and stirring overnight. Then, brine (50 mL) was added and the aqueous phase separated again. The organic phase was dried over $MgSO_4$ and the solvent removed under reduced pressure. The crude product was purified using flash column chromatography (SiO_2, 80:20 pentante/EtOAc) to yield the product as a colorless oil (5.65 g, 30.83 mmol, 80%). The spectral data matches those reported in the literature.[15]

NMR Spectroscopy (*see spectra*)

^{1}H NMR (400 MHz, $CDCl_3$): δ_H 7.08 (dd, *J* = 15.6, 2.7 Hz, 1H), 6.82 (dq, *J* = 15.5, 6.7 Hz, 1H), 3.74 (s, 3H), 3.29 (s, 3H) ppm;

^{13}C NMR (151 MHz, $CDCl_3$): δ_C 163.6, 130.3 (q, *J* = 35.0 Hz), 126.5 (q, *J* = 5.8 Hz), 122.7 (q, *J* = 269.6 Hz), 62.3, 32.5;

^{19}F NMR (377 MHz, $CDCl_3$) δ_F -65.06 ppm.

(*E*)-*N,N*-Diallyl-4,4,4-trifluorobut-2-enamide (2u)

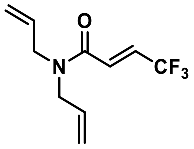

2u

The compound was prepared following a modified literature procedure. Under argon atmosphere, 4,4,4-trifluorobut-2-enoic acid (1.40 g, 10.00 mmol, 1.00 equiv.) and EDCI·HCl (2.30 g, 12.00 mmol, 1.20 equiv.) were dissolved in dry CH_2Cl_2 (35 mL) and cooled to 0°C. Diallylamine (1.60 mL, 13.00 mmol, 1.30 equiv.) and DIPEA (2.80 mL, 16.00 mmol, 1.60 equiv.) were added. The reaction mixture was stirred at room temperature for 16 h and quenched with brine. The phases were separated and the organic layer was dried over $MgSO_4$ and concentrated. The crude reaction mixture was purified by flash column chromatography (SiO_2; 9:1 pentane/EtOAc) to afford **2u** as a colorless oil (1.30 g, 5.90 mmol, 59%).[15]

TLC: R_f = 0.40 (9:1 pentane/EtOAc).

NMR Spectroscopy (*see spectra*):

^{1}H NMR (500 MHz, $CDCl_3$): δ_H 6.89 – 6.71 (m, 2H), 5.85 – 5.72 (m, 2H), 5.30 – 5.14 (m, 4H), 4.06 (dt, *J* = 6.1, 1.4 Hz, 2H), 3.95 (dt, *J* = 4.8, 1.8 Hz, 2H) ppm;

^{13}C NMR (126 MHz, $CDCl_3$): δ_C 163.7, 132.5, 132.4, 129.8 (q, *J* = 34.8 Hz), 128.3 (q, *J* = 5.9 Hz), 122.7 (q, *J* = 270.0 Hz), 118.4, 117.6, 49.4, 49.0 ppm;

^{19}F NMR (377 MHz, $CDCl_3$): δ_F -65.05 ppm.

HRMS (ESI$^+$): m/z calc'd for $C_{10}H_{12}F_3NONa$ $[M+Na]^+$: 242.0769, found: 242.0759.

(3*S*,8*S*,9*S*,10*R*,13*R*,14*S*,17*R*)-10,13-Dimethyl-17-((*R*)-6-methylheptan-2-yl)-2,3,4,7,8,9,10,11,12,13,14,15,16,17-tetradecahydro-1*H*-cyclopenta[a]phenanthren-3-yl acrylate (2ae)

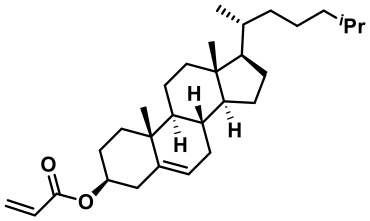

2ae

The title compound was synthesized as white solid following General Procedure E using (3*S*,8*S*,9*S*,10*R*,13*R*,14*S*,17*R*)-10,13-dimethyl-17-((*R*)-6-methylheptan-2-yl)-2,3,4,7,8,9,10,11,12,13,14,15,16,17-tetradecahydro-1*H*-cyclopenta[a]phenanthren-3-ol (9.32 g, 25.00 mmol, 1.00 equiv.) and acryloyl chloride (2.10 mL, 26.25 mmol, 1.05 equiv.). The crude reaction mixture was purified by flash column chromatography (SiO_2; 98:2 pentane/EtOAc) to afford **2ae** as white solid (7.05 g, 16.00 mmol, 64%).

TLC: R_f = 0.90 (90:10 pentane/EtOAc).

NMR Spectroscopy (*see spectra*):

^{1}H NMR (400 MHz, CD_2Cl_2): δ_H 6.35 (dd, *J* = 17.3, 1.6 Hz, 1H), 6.10 (dd, *J* = 17.3, 10.4 Hz, 1H), 5.79 (dd, *J* = 10.4, 1.6 Hz, 1H), 5.40 (d, *J* = 5.2 Hz, 1H), 4.63 (m, 1H), 2.41 – 2.30 (m, 2H), 2.07 – 1.94 (m, 2H), 1.94 – 1.80 (m, 3H), 1.67 – 0.99 (m, 24H), 0.93 (d, *J* = 6.5 Hz, 3H), 0.87 (dd, *J* = 6.6, 1.6 Hz, 6H), 0.70 (s, 3H) ppm;

^{13}C NMR (101 MHz, CD_2Cl_2): δ_C 161.0, 135.4, 125.5, 124.7, 118.1, 69.7, 52.3, 51.8, 45.7, 37.9, 35.4, 35.1, 33.7, 32.6, 32.2, 31.8, 31.4, 27.5, 27.5, 23.8, 23.6, 23.3, 19.8, 19.4, 18.1, 17.9, 16.6, 14.7, 14.1, 7.2 ppm.

HRMS (ESI$^+$): m/z calc'd for $C_{30}H_{48}O_2Na$ $[M+Na]^+$: 463.35465, found: 463.3548.

(*Z*)-3,7-Dimethylocta-2,6-dien-1-yl acrylate (2af)

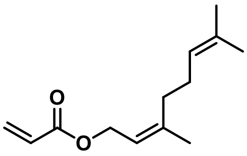

2af

The title compound was synthesized following **General Procedure E** using (*E*)-3,7-dimethylocta-2,6-dien-1-ol (3.86 g, 25.00 mmol, 1.00 equiv.) and acryloyl chloride (2.10 mL, 26.25 mmol, 1.05 equiv.). The crude reaction mixture was purified by flash column chromatography (SiO_2; 98:2 pentane/EtOAc) to afford **2af** as colorless oil (4.48 g, 21.50 mmol, 86%).

TLC: R_f = 0.90 (90:10 pentane/EtOAc).

NMR Spectroscopy (*see spectra*):

^{1}H NMR (400 MHz, $CDCl_3$): δ_H 6.39 (dd, *J* = 17.3, 1.5 Hz, 1H), 6.12 (dd, *J* = 17.3, 10.4 Hz, 1H), 5.80 (dd, *J* = 10.4, 1.5 Hz, 1H), 5.37 (tq, *J* = 7.2, 1.4 Hz, 1H), 5.07 (dddd, *J* = 8.3, 6.7, 2.9, 1.5 Hz, 1H), 4.67 (d, *J* = 7.1 Hz, 2H), 2.15 – 1.96 (m, 4H), 1.69 (dd, *J* = 17.1, 1.4 Hz, 6H), 1.59 (d, *J* = 1.4 Hz, 3H) ppm;

^{13}C NMR (101 MHz, $CDCl_3$): δ_C 166.7, 142.8, 132.3, 130.9, 129.1, 124.2, 118.6, 61.9, 40.0, 26.7, 26.1, 18.1, 16.9 ppm.

HRMS (ESI$^+$): m/z calc'd for $C_{13}H_{20}O_2Na$ $[M+Na]^+$: 231.1356, found: 231.1354.

2-(4-(2,2-Dichlorocyclopropyl)phenoxy)-2-methylpropyl (*E*)-4,4,4-trifluorobut-2-enoate (2ag)

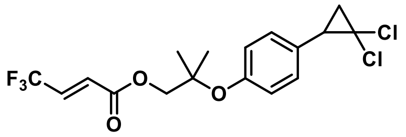

2ag

The compound was prepared following a modified literature procedure.[16] Under argon atmosphere, 4,4,4-Trifluorobut-2-enoic acid (0.56 g, 4.00 mmol, 1.00 equiv.) was dissolved in dry CH_2Cl_2 (40 mL) and EDCl·HCl (0.09 g, 4.80 mmol, 1.20 equiv.), DMAP (0.05 g, 0.40 mmol, 0.10 equiv.) and **S5** (1.30 g, 4.80 mmol, 1.20 equiv.) were added. The reaction mixture was stirred at room temperature for 13 h and quenched with brine. The phases were separated, and the organic layer was dried over $MgSO_4$ and concentrated. The crude reaction mixture was purified by flash column chromatography (SiO_2; 9:1 pentane/EtOAc) to afford **2ag** as a colorless oil (0.07 g, 1.70 mmol, 41%).

TLC: R_f = 0.76 (9:1 pentane/EtOAc).

NMR Spectroscopy (*see spectra*):

^{1}H NMR (599 MHz, $CDCl_3$): δ_H 7.18 – 7.11 (m, 2H), 6.99 – 6.93 (m, 2H), 6.80 (dq, *J* = 15.8, 6.5 Hz, 1H), 6.57 (dq, *J* = 15.8, 1.9 Hz, 1H), 4.25 (s, 2H), 2.86 (dd, *J* = 10.7, 8.3 Hz, 1H), 1.96 (dd, *J* = 10.7, 7.4 Hz, 1H), 1.80 (dd, *J* = 8.3, 7.4 Hz, 1H), 1.35 (s, 6H) ppm;

^{13}C NMR (151 MHz, $CDCl_3$): δ_C 163.8, 154.0, 132.0 (m), 130.5, 129.8, 128.6 (m), 124.0, 122.1 (d, *J* = 270.5 Hz), 78.6, 70.9, 60.9, 35.1, 26.1, 24.1 ppm;

^{19}F NMR (376 MHz, $CDCl_3$): δ_F -65.55 ppm.

HRMS (ESI$^+$): m/z calc'd for $C_{17}H_{17}Cl_2F_3O_3Na$ $[M+Na]^+$: 419.0405, found: 419.0399.

5-(2,5-Dimethylphenoxy)-2,2-dimethylpentyl (*E*)-4,4,4-trifluorobut-2-enoate (2ah)

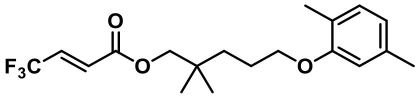

2ah

The title compound was synthesized following **General Procedure D** using **S3** (1.30 g, 9.30 mmol, 1.10 equiv.) and (*E*)-4,4,4-trifluorobut-2-enoic acid (2.00 g, 8.46 mmol, 1.00 equiv.) The crude reaction mixture was purified by flash column chromatography (SiO_2; 98:2 pentane/EtOAc) to afford **2ah** as colorless oil (2.09 g, 5.84 mmol, 69%).

TLC: R_f = 0.89 (90:10 pentane/EtOAc).

NMR Spectroscopy (*see spectra*):

^{1}H NMR (400 MHz, $CDCl_3$): δ_H 7.05 (d, *J* = 7.5 Hz, 1H), 6.84 (m, 1H), 6.71 (dd, *J* = 7.7, 1.6 Hz, 1H), 6.67 (d, *J* = 1.8 Hz, 1H), 6.58 (dq, *J* = 15.7, 1.9 Hz, 1H), 4.04 (s, 2H), 3.97 (t, *J* = 6.2 Hz, 2H), 2.36 (s, 3H), 2.23 (s, 3H), 1.89 – 1.76 (m, 2H), 1.60 – 1.49 (m, 2H), 1.05 (d, *J* = 1.6 Hz, 6H) ppm;

^{13}C NMR (101 MHz, $CDCl_3$): δ_C 164.3, 157.4, 136.8, 132.2, δ 131.7 (q, *J* = 35.4 Hz), 129.3 (q, *J* = 6.2 Hz), 123.9, 123.8, 121.1, 112.3, 73.7, 68.5, 35.7, 34.1, 24.5, 24.5, 21.7, 16.1 ppm;

^{19}F NMR (377 MHz, $CDCl_3$): δ_F -65.51 ppm.

HRMS (ESI$^+$): m/z calc'd for $C_{19}H_{25}O_3F_3Na$ $[M+Na]^+$: 381.16480, found: 381.1648.

2.5. General Procedure F: Photochemical Synthesis of Cyclic Imine Products (Alkene Scope)

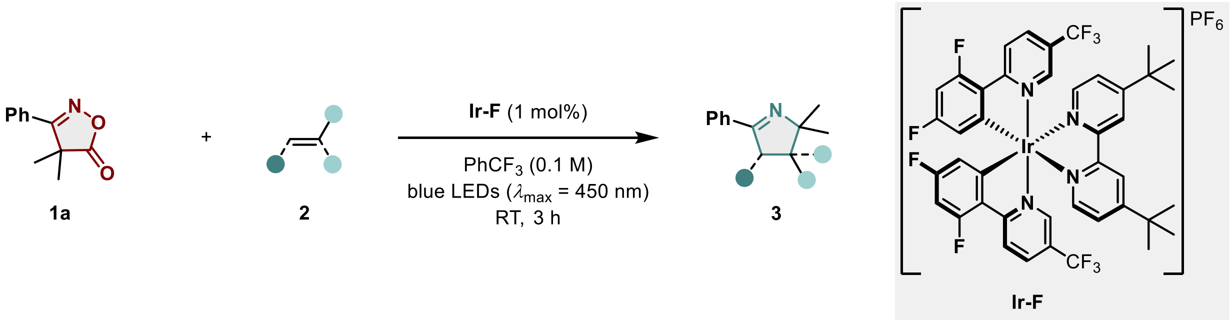

To an oven-dried 10 mL Schlenk tube equipped with a Teflon-coated magnetic stir bar was added $[Ir\{dF(CF_3)ppy\}_2(dtbbpy)]PF_6$ (**Ir-F**) (2.20 mg, 2.0 µmol, 1 mol%), 4,4-dimethyl-3-phenylisoxazol-5(4*H*)-one **1a** (37.80 mg, 0.20 mmol, 1.00 equiv.), and the respective olefin **2** (2.00 mmol, 10.00 equiv.). The Schlenk tube was evacuated and backfilled with argon three times before $PhCF_3$ (2.0 mL) was added under a positive argon pressure. The reaction mixture was stirred under irradiation with blue LEDs (18 W, λ_{max} = 450 nm) for 3 h.[A] After this time, the solvent was removed under reduced pressure and the crude product was purified by flash column chromatography on silica gel to yield the corresponding 5-phenyl-3,4-dihydro-2*H*-pyrrole (**3**).

Note: (**A**) Reaction times might vary according to the different bifunctional reagents used.

2.6. General Procedure G: Photochemical Synthesis of Cyclic Imine Products (Oxime Scope)

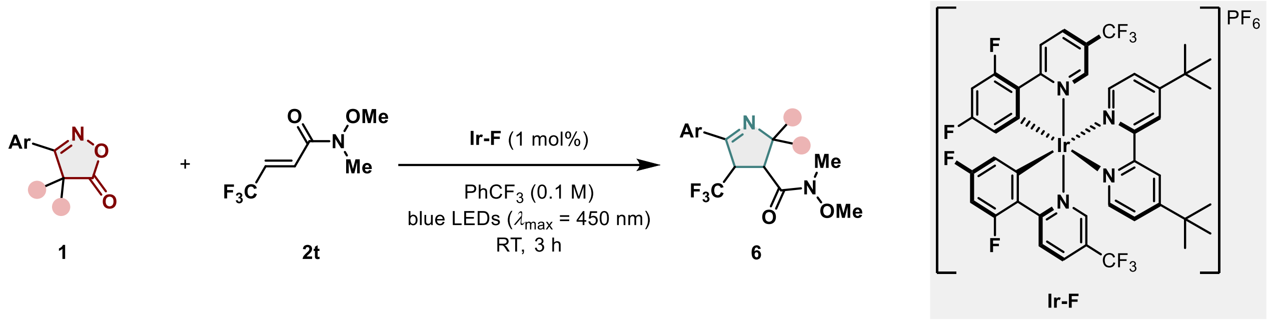

To an oven-dried 10 mL Schlenk tube equipped with a Teflon-coated magnetic stir bar was added $[Ir\{dF(CF_3)ppy\}_2(dtbbpy)]PF_6$ (**Ir-F**) (2.20 mg, 2.0 µmol, 1 mol%) and isoxazolone substrate **1** (0.20 mmol, 1.00 equiv.). The Schlenk tube was evacuated and backfilled with argon three times before $PhCF_3$ (2 mL, 0.1 M) and (*E*)-4,4,4-trifluoro-methoxy-methylbut-2-enamide **2t** (0.18 g, 1.00 mmol, 5.00 equiv.) were added under a positive argon pressure. The reaction mixture was stirred under irradiation with blue LEDs (18 W, λ_{max} = 450 nm) for the indicated reaction time. Afterwards, the crude reaction mixture was transferred into a scintillation vial with CH_2Cl_2 rinsing and the volatiles were removed under reduced pressure. Then, CH_2Br_2 (7.0 µL, 0.10 mmol, 0.50 equiv.) was added as an internal standard, and crude ^{1}H-NMR was measured. The NMR tube, pipettes, and Schlenk tube were carefully rinsed with CH_2Cl_2 into the previously used scintillation vial. After removal of the solvents under reduced pressure, the product was purified by silica-gel flash column chromatography.

2,2-Dimethyl-5-phenyl-3,4-dihydro-2*H*-pyrrole-3-carbonitrile (3a)

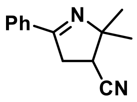

3a

Synthesized following **General Procedure F** using: **1a** (37.80 mg, 0.20 mmol, 1.00 equiv.), and acrylonitrile (2.00 mmol, 10.00 equiv.). Purified by flash column chromatography (SiO_2; 100:0 to 90:10 pentane/EtOAc) to afford **3a** (28.00 mg, 0.14 mmol, 69%) as a pale-yellow solid.

TLC: R_f = 0.33 (9:1 pentane/EtOAc).

NMR Spectroscopy (*see spectra*):

^{1}H NMR (400 MHz, $CDCl_3$): δ_H 7.82 – 7.75 (m, 2H), 7.50 – 7.39 (m, 3H), 3.48 (dd, *J* = 16.8, 9.5 Hz, 1H), 3.33 (dd, *J* = 16.8, 8.9 Hz, 1H), 2.97 (dd, *J* = 9.5, 8.8 Hz, 1H), 1.54 (s, 3H), 1.47 (s, 3H) ppm;

^{13}C NMR (126 MHz, $CDCl_3$): δ_C 167.2, 133.3, 131.3, 128.8, 127.8, 120.0, 75.0, 40.0, 37.9, 29.1, 25.1 ppm.

HRMS (ESI^+): m/z calc'd for $C_{13}H_{15}N_2$ $[M+H]^+$: 199.1230, found: 199.1230.

X-ray (*see data*)

Benzyl-2,2-dimethyl-5-phenyl-3,4-dihydro-2*H*-pyrrole-3-carboxylate (3b)

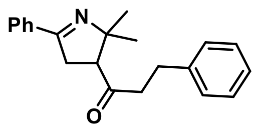

3b

Synthesized following General Procedure F using: **1a** (37.80 mg, 0.20 mmol, 1.00 equiv.), and benzyl acrylate (2.00 mmol, 10.00 equiv.). Purified by flash column chromatography (SiO_2; 100:0 to 90:10 pentane/EtOAc) to afford **3b** (18.00 mg, 0.06 mmol, 30%) as a colorless oil.

TLC: R_f = 0.44 (90:10 pentane/EtOAc).

NMR Spectroscopy (*see spectra*):

^{1}H NMR (400 MHz, $CDCl_3$): δ_H 7.98 – 7.69 (m, 2H), 7.60 – 7.31 (m, 8H), 5.35 – 5.04 (m, 2H), 3.55 (dd, *J* = 17.1, 8.9 Hz, 1H), 3.21 (dd, *J* = 17.1, 9.2 Hz, 1H), 3.09 (t, *J* = 9.0 Hz, 1H), 1.59 (s, 3H), 1.13 (s, 3H) ppm;

^{13}C NMR (101 MHz, $CDCl_3$): δ_C 172.6, 168.9, 135.8, 131.1, 128.8, 128.7, 128.6, 128.6, 128.0, 75.3, 66.9, 52.7, 38.0, 30.1, 23.7 ppm.

HRMS (ESI$^+$): m/z calc'd for $C_{20}H_{21}N_1O_2Na$ [M+Na]$^+$: 330.1465, found: 330.1465.

4-Iodobenzyl-2,2-dimethyl-5-phenyl-3,4-dihydro-2*H*-pyrrole-3-carboxylate (3c)

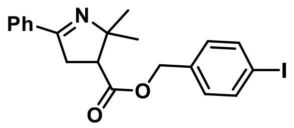

3c

Synthesized following General Procedure F using: **1a** (37.80 mg, 0.20 mmol, 1.00 equiv.), and 4-iodobenzyl acrylate (2.00 mmol, 10.00 equiv.). Purified by flash column chromatography (SiO_2; 100:0 to 90:10 pentane/EtOAc) to afford **3c** (25 mg, 0.06 mmol, 29%) as a colorless oil.

TLC: R_f = 0.50 (90:10 pentane/EtOAc).

NMR Spectroscopy (*see spectra*):

^{1}H NMR (400 MHz, $CDCl_3$): δ_H 7.84 – 7.77 (m, 2H), 7.75 – 7.65 (m, 2H), 7.46 – 7.34 (m, 3H), 7.19 – 7.09 (m, 2H), 5.19 – 5.03 (m, 2H), 3.51 (dd, *J* = 17.0, 8.9 Hz, 1H), 3.18 (dd, *J* = 17.0, 9.2 Hz, 1H), 3.06 (t, *J* = 9.0 Hz, 1H), 1.56 (s, 3H), 1.10 (s, 3H) ppm;

^{13}C NMR (101 MHz, $CDCl_3$): δ_C 173.0, 168.5, 134.3, 130.7, 128.6, 127.8, 75.3, 60.8, 52.9, 38.1, 30.2, 23.8, 14.5 ppm.

HRMS (ESI$^+$): m/z calc'd for $C_{20}H_{21}NO_2I$ [M+H]$^+$: 434.0612, found: 434.0611.

3-(Trifluoromethyl)benzyl-2,2-dimethyl-5-phenyl-3,4-dihydro-2*H*-pyrrole-3-carboxylate (3d)

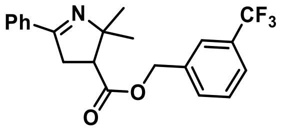

3d

Synthesized following **General Procedure F** using: **1a** (37.80 mg, 0.20 mmol, 1.00 equiv.), and 3-(trifluoromethyl)benzyl acrylate (2.00 mmol, 10.00 equiv.). Purified by flash column chromatography (SiO_2; 100:0 to 85:15 pentane/EtOAc) to afford **3d** (24.00 mg, 0.06 mmol, 32%) as a colorless oil.

TLC: R_f = 0.25 (95:5 pentane/EtOAc).

NMR Spectroscopy (*see spectra*):

^{1}H NMR (400 MHz, $CDCl_3$): δ_H 7.85 – 7.76 (m, 2H), 7.69 – 7.56 (m, 3H), 7.55 – 7.48 (m, 1H), 7.45 – 7.36 (m, 3H), 5.28 – 5.18 (m, 2H), 3.52 (dd, *J* = 16.9, 8.7 Hz, 1H), 3.20 (dd, *J* = 16.9, 9.2 Hz, 1H), 3.09 (t, *J* = 9.0 Hz, 1H), 1.57 (s, 3H), 1.11 (s, 3H) ppm;
^{13}C NMR (101 MHz, $CDCl_3$): δ_C 172.7, 168.4, 136.9, 134.2, 131.8 (d, *J* = 1.5 Hz), 131.2 (d, *J* = 30.3 Hz), 130.8, 129.3, 128.6, 127.8, 126.2 – 124.4 (m), 75.5, 65.8, 52.8, 38.2, 30.2, 23.8 ppm;

^{19}F NMR (376 MHz, $CDCl_3$): δ_F -62.73 ppm.

HRMS (ESI^+): m/z calc'd for $C_{21}H_{21}NO_2F_3$ $[M+H]^+$: 376.1519, found: 376.1520.

2-Methylbenzyl-2,2-dimethyl-5-phenyl-3,4-dihydro-2*H*-pyrrole-3-carboxylate (3e)

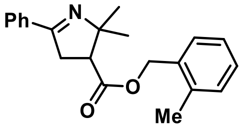

3e

Synthesized following **General Procedure F** using: **1a** (37.80 mg, 0.20 mmol, 1.00 equiv.), and 2-methylbenzyl acrylate (2.00 mmol, 10.00 equiv.). Purified by flash column chromatography (SiO_2; 100:0 to 90:10 pentane/EtOAc) to afford **3e** (18.00 mg, 0.06 mmol, 28%) as a colorless oil.

TLC: R_f = 0.60 (9:1 pentane/EtOAc).

NMR Spectroscopy (*see spectra*):

^{1}H NMR (400 MHz, $CDCl_3$): δ_H 7.98 – 7.74 (m, 2H), 7.48 – 7.34 (m, 4H), 7.29 – 7.17 (m, 3H), 5.25 – 5.17 (m, 2H), 3.54 (dd, *J* = 17.0, 8.9 Hz, 1H), 3.20 (dd, *J* = 17.0, 9.2 Hz, 1H), 3.08 (t, *J* = 9.1 Hz, 1H), 2.40 (s, 3H), 1.57 (s, 3H), 1.13 (s, 3H) ppm;

^{13}C NMR (101 MHz, $CDCl_3$): δ_C 172.8, 168.7, 137.1, 133.8, 130.9, 130.6, 129.7, 128.8, 128.6, 127.9, 126.2, 75.4, 65.1, 52.8, 38.1, 30.2, 23.8, 19.1 ppm.

HRMS (ESI^+): m/z calc'd for $C_{21}H_{23}NO_2Na$ $[M+Na]^+$: 344.1621, found: 344.1619.

4-Methoxybenzyl-2,2-dimethyl-5-phenyl-3,4-dihydro-2*H*-pyrrole-3-carboxylate (3f)

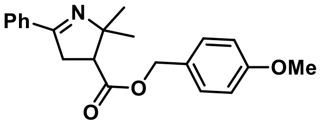

3f

Synthesized following **General Procedure F** using: **1a** (37.80 mg, 0.20 mmol, 1.00 equiv.), and 4-methoxybenzyl acrylate (2.00 mmol, 10.00 equiv.). Purified by flash column chromatography (SiO_2; 100:0 to 85:15 pentane/EtOAc) to afford **3f** (17.00 mg, 0.05 mmol, 25%) as a colorless oil.

TLC: R_f = 0.20 (90:10 pentane/EtOAc).

NMR Spectroscopy (*see spectra*):

^{1}H NMR (400 MHz, $CDCl_3$): δ_H 7.84 – 7.77 (m, 2H), 7.48 – 7.27 (m, 5H), 6.94 – 6.85 (m, 2H), 5.19 – 5.05 (m, 2H), 3.82 (s, 3H), 3.51 (dd, *J* = 16.9, 9.0 Hz, 1H), 3.17 (dd, *J* = 17.0, 9.2 Hz, 1H), 3.04 (t, *J* = 9.1 Hz, 1H), 1.55 (s, 3H), 1.09 (s, 3H) ppm;

^{13}C NMR (101 MHz, $CDCl_3$): δ_C 172.9, 168.5, 159.8, 134.3, 130.7, 130.5, 128.6, 128.1, 127.8, 114.1, 75.4, 66.6, 55.4, 52.8, 38.1, 30.2, 23.8 ppm.

HRMS (ESI^+): m/z calc'd for $C_{21}H_{24}NO_3$ $[M+H]^+$: 338.17507, found: 338.17536.

Ethyl-2,2-dimethyl-5-phenyl-3,4-dihydro-2*H*-pyrrole-3-carboxylate (3g)

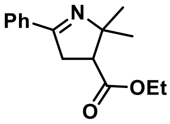

3g

Synthesized following **General Procedure F** using: **1a** (37.80 mg, 0.20 mmol, 1.00 equiv.), and ethyl acrylate (2.00 mmol, 10.00 equiv.). Purified by flash column chromatography (SiO_2; 100:0 to 90:10 pentane/EtOAc) to afford **3g** (17.00 mg, 0.07 mmol, 34%) as a colorless oil.

TLC: R_f = 0.71 (7:3 pentane/EtOAc).

NMR Spectroscopy (*see spectra*):

^{1}H NMR (400 MHz, $CDCl_3$): δ_H 7.84 – 7.77 (m, 2H), 7.45 – 7.38 (m, 3H), 4.21 (qd, *J* = 17.9, 10.8 Hz, 2H), 3.51 (dd, *J* = 17.0, 9.0 Hz, 1H), 3.16 (dd, *J* = 17.1, 9.2 Hz, 1H), 3.01 (t, *J* = 9.1 Hz, 1H), 1.58 (s, 3H), 1.31 (t, *J* = 7.1 Hz, 3H), 1.17 (s, 3H) ppm;

^{13}C NMR (101 MHz, $CDCl_3$): δ_C 173.0, 168.5, 134.3, 130.7, 128.6, 127.8, 75.3, 60.8, 52.9, 38.1, 30.2, 23.8, 14.5 ppm.

HRMS (ESI^+): m/z calc'd for $C_{15}H_{20}NO_2$ $[M+H]^+$: 246.1489, found: 246.1488.

1,1,1,3,3,3-Hexafluoropropan-2-yl-2,2-dimethyl-5-phenyl-3,4-dihydro-2*H*-pyrrole-3-carboxylate (3h)

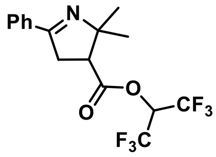

3h

Synthesized following **General Procedure F** using: **1a** (37.80 mg, 0.20 mmol, 1.00 equiv.), and 1,1,1,3,3,3-hexafluoropropan-2-yl acrylate (2.00 mmol, 10.00 equiv.). Purified by flash column chromatography (SiO_2; 100:0 to 90:10 pentane/EtOAc) to afford **3h** (32.00 mg, 0.09 mmol, 44%) as a colorless oil.

TLC: R_f = 0.88 (8:2 pentane/EtOAc).

NMR Spectroscopy (*see spectra*):

^{1}H NMR (400 MHz, $CDCl_3$): δ_H 7.84 – 7.79 (m, 2H), 7.47 – 7.39 (m, 3H), 5.84 (h, *J* = 6.1 Hz, 1H), 3.53 (dd, *J* = 16.8, 9.1 Hz, 1H), 3.30 (dd, *J* = 16.8, 9.1 Hz, 1H), 3.21 (t, *J* = 9.0 Hz, 1H), 1.77 (s, 3H), 1.18 (s, 3H) ppm;

^{13}C NMR (101 MHz, $CDCl_3$): δ_C 169.8, 167.7, 133.6, 130.9, 129.6, 128.6, 127.7, 126.6, 120.9, 75.7, 66.6 (h, *J* = 69.4 Hz), 52.26, 38.1, 29.8, 24.8 ppm;

^{19}F NMR (376 MHz, $CDCl_3$): δ_F -72.78 (q, *J* = 8.7 Hz), -73.16 (q, *J* = 8.6 Hz) ppm.

HRMS (ESI^+): m/z calc'd for $C_{16}H_{16}NO_2F_6$ $[M+H]^+$: 368.1090, 369.1112 found: 368.1076, 369.1111.

Prop-2-yn-1-yl-2,2-dimethyl-5-phenyl-3,4-dihydro-2*H*-pyrrole-3-carboxylate (3i)

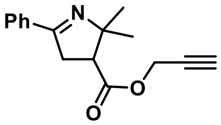

3i

Synthesized following **General Procedure F** using: **1a** (37.80 mg, 0.20 mmol, 1.00 equiv.), and prop-2-yn-1-yl acrylate (2.00 mmol, 10.00 equiv.). Purified by flash column chromatography (SiO_2; 100:0 to 90:10 pentane/EtOAc) to afford **3i** (11.00 mg, 0.04 mmol, 22%) as a colorless oil.

TLC: R_f = 0.45 (8:2 pentane/EtOAc).

NMR Spectroscopy (*see spectra*):

^{1}H NMR (400 MHz, $CDCl_3$): δ_H 7.88 – 7.78 (m, 2H), 7.45 – 7.38 (m, 3H), 4.89 – 4.63 (m, 2H), 3.53 (dd *J* = 17.0, 8.9 Hz, 1H), 3.20 (dd, *J* = 17.0, 9.2 Hz, 1H), 3.07 (t, *J* = 9.1 Hz, 1H), 2.49 (t, *J* = 2.4 Hz, 1H), 1.60 (s, 3H), 1.20 (s, 3H) ppm;

^{13}C NMR (101 MHz, $CDCl_3$): δ_C 172.2, 168.3, 134.2, 130.8, 128.6, 127.8, 75.7, 75.1, 52.6, 52.2, 38.1, 30.2, 23.8 ppm.

HRMS (ESI^+): m/z calc'd for $C_{16}H_{18}NO_2$ $[M+H]^+$: 256.1332, found: 256.1329.

Furan-2-ylmethyl-2,2-dimethyl-5-phenyl-3,4-dihydro-2*H*-pyrrole-3-carboxylate (3k)

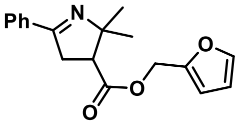

3k

Synthesized following General Procedure F using: **1a** (37.80 mg, 0.20 mmol, 1.00 equiv.), and 3-furan-2-ylmethyl acrylate (2.00 mmol, 10.00 equiv.). Purified by flash column chromatography (SiO_2; 100:0 to 85:15 pentane/EtOAc) to afford **3k** (24.00 mg, 0.08 mmol, 40%) as a colorless oil.

TLC: R_f = 0.50 (90:10 pentane/EtOAc).

NMR Spectroscopy (*see spectra*):

^{1}H NMR (400 MHz, $CDCl_3$): δ_H 7.80 (dd, *J* = 7.9, 1.7 Hz, 2H), 7.48 – 7.35 (m, 4H), 6.44 (dd, *J* = 3.3, 0.8 Hz, 1H), 6.37 (dd, *J* = 3.3, 1.9 Hz, 1H), 5.25 – 5.04 (m, 2H), 3.51 (dd, *J* = 17.0, 8.9 Hz, 1H), 3.17 (dd, *J* = 17.0, 9.2 Hz, 1H), 3.04 (t, *J* = 9.1 Hz, 1H), 1.55 (s, 3H), 1.09 (s, 3H) ppm;

^{13}C NMR (101 MHz, $CDCl_3$): δ_C 172.6, 168.4, 149.6, 143.4, 134.3, 130.7, 128.6, 127.8, 111.0, 110.7, 75.5, 58.3, 52.7, 38.1, 30.2, 23.6 ppm.

HRMS (ESI$^+$): m/z calc'd for $C_{18}H_{20}NO_3$ $[M+H]^+$: 298.1438, found: 298.1438.

***S*-Cyclohexyl 2,2-dimethyl-5-phenyl-3,4-dihydro-2*H*-pyrrole-3-carbothioate (3l)**

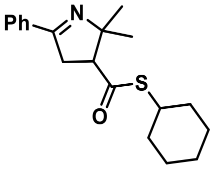

3l

Synthesized following General Procedure F using: **1a** (37.80 mg, 0.20 mmol, 1.00 equiv.), and *S*-cyclohexyl prop-2-enethioate (2.00 mmol, 10.00 equiv.). Reaction mixture was irradiated for 4 h. Purified by flash column chromatography (SiO_2; 99:1 to 9:1 pentane/EtOAc) to afford **3l** as a colorless oil (28.00 mg, 0.09 mmol, 44%).

TLC: R_f = 0.1 (99:1 pentane/EtOAc).

NMR Spectroscopy (*see spectra*):

^{1}H NMR (599 MHz, $CDCl_3$): δ_H 7.83 – 7.78 (m, 2H), 7.46 – 7.36 (m, 3H), 3.64 – 3.50 (m, 2H), 3.25 – 3.07 (m, 2H), 1.99 – 1.90 (m, 2H), 1.74 – 1.69 (m, 2H), 1.62 (s, 3H), 1.48 – 1.41 (m, 5H), 1.32 – 1.26 (m, 1H), 1.16 (s, 3H) ppm;

^{13}C NMR (151 MHz, $CDCl_3$): δ_C 198.4, 168.1, 134.2, 130.8, 128.6, 127.8, 75.6, 61.3, 42.8, 38.0, 33.31, 32.9, 30.3, 25.7, 23.6 ppm.

HRMS (ESI$^+$): m/z calc'd for $C_{19}H_{25}NNaOS$ $[M+Na]^+$: 338.1555, found: 338.1549.

2-Methoxy-2-oxo-1-phenylethyl-2,2-dimethyl-5-phenyl-3,4-dihydro-2*H*-pyrrole-3-carboxylate (3m)

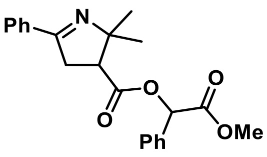

3m

Synthesized following **General Procedure F** using: **1a** (37.80 mg, 0.20 mmol, 1.00 equiv.), and 2-methylbenzyl acrylate (2.00 mmol, 10.00 equiv.). Purified by flash column chromatography (SiO_2; 100:0 to 95:5 pentane/EtOAc) to afford **3m** (32.00 mg, 0.09 mmol, 44%) as a colorless oil. (d.r. = 54:46)

TLC: R_f = 0.70 (90:10 pentane/EtOAc).

NMR Spectroscopy (*see spectra*):

^{1}H NMR (400 MHz, $CDCl_3$): δ_H 7.81 (m, 4H), 7.56 – 7.31 (m, 16H), 6.02 (s, 1H, d^1), 6.00 (s, 0.83H, d^2), 3.74 (d, J = 3.8 Hz, 6H), 3.58 – 3.48 (m, 2H), 3.34 – 3.05 (m, 4H), 1.71 (s, 2.67H, d^2), 1.55 (s, 3H, d^1), 1.31 (s, 2.64H, d^2), 1.10 (s, 3H, d^1) ppm; (d^1 = major diastereomer, d^2 = minor diastereomer)
^{13}C NMR (101 MHz, $CDCl_3$): δ_C 172.0, 171.9, 169.0, 168.9 167.9, 167.8, 133.9, 133.8, 133.6, 133.5, 130.4, 130.4, 129.1, 128.7, 127.5, 127.4, 127.4, 75.3, 75.1, 74.4, 52.5, 52.5, 52.4, 52.2, 37.8, 29.8, 29.6, 23.4, 23.1 ppm.
HRMS (ESI$^+$): m/z calc'd for $C_{22}H_{24}NO_4$ $[M+H]^+$: 366.1700, found: 366.1699.

2-Iodoethyl-2,2-dimethyl-5-phenyl-3,4-dihydro-2*H*-pyrrole-3-carboxylate (3n)

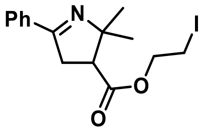

3n

Synthesized following **General Procedure F** using: **1a** (37.80 mg, 0.20 mmol, 1.00 equiv.), and 4-methoxybenzyl acrylate (2.00 mmol, 10.00 equiv.). Purified by flash column chromatography (SiO_2; 100:0 to 85:15 pentane/EtOAc) to afford **3n** (30.00 mg, 0.08 mmol, 41%) as a colorless oil.

TLC: R_f = 0.30 (90:10 pentane/EtOAc).

NMR Spectroscopy (*see spectra*):

^{1}H NMR (400 MHz, $CDCl_3$): δ_H 7.86 – 7.78 (m, 2H), 7.50 – 7.33 (m, 3H), 4.50 – 4.33 (m, 2H), 3.52 (dd, J = 16.9, 8.9 Hz, 1H), 3.35 (t, J = 6.6 Hz, 3H), 3.19 (dd, J = 17.0, 9.2 Hz, 1H), 3.06 (t, J = 9.1 Hz, 1H), 1.62 (s, 3H), 1.20 (s, 3H) ppm;

^{13}C NMR (101 MHz, $CDCl_3$): δ_C 172.4, 168.3, 134.2, 130.8, 128.6, 127.8, 75.4, 65.1, 52.7, 38.1, 30.2, 23.9 ppm.

HRMS (ESI$^+$): m/z calc'd for $C_{20}H_{21}NO_2I$ $[M+H]^+$: 434.0612, found: 434.0611.

2-((*tert*-Butyldimethylsilyl)oxy)ethyl-2,2-dimethyl-5-phenyl-3,4-dihydro-2*H*-pyrrole-3-carboxylate (3o)

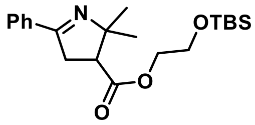

3o

Synthesized following General Procedure F using: **1a** (37.80 mg, 0.20 mmol, 1.00 equiv.), and 2-((tert-butyldimethylsilyl)oxy)ethyl acrylate (2.00 mmol, 10.00 equiv.). Purified by flash column chromatography (SiO_2; 95:5 to 9:1 pentane/EtOAc) to afford **3o** as a colorless oil (31.00 mg, 0.08 mmol, 41%).

TLC: R_f = 0.26 (95:5 pentane/EtOAc).

NMR Spectroscopy (*see spectra*):

^{1}H NMR (599 MHz, $CDCl_3$): δ_H 7.85 – 7.76 (m, 2H), 7.47 – 7.35 (m, 3H), 4.29 – 4.14 (m, 2H), 3.85 (t, *J* = 5.0 Hz, 2H), 3.52 (dd, *J* = 17.0, 8.9 Hz, 1H), 3.17 (dd, *J* = 17.0, 9.2 Hz, 1H), 3.05 (t, *J* = 9.1 Hz, 1H), 1.59 (s, 3H), 1.18 (s, 3H), 0.91 (s, 9H), 0.08 (d, *J* = 1.1 Hz, 6H) ppm;

^{13}C NMR (151 MHz, $CDCl_3$): δ_C 173.0, 168.4, 134.3, 130.7, 128.6, 127.8, 75.4, 66.2, 61.3, 52.9, 38.1, 30.2, 26.0, 23.8, 18.4, -5.2 ppm.

HRMS (ESI^+): m/z calc'd for $C_{21}H_{33}NO_3SiNa$ $[M+Na]^+$: 398.2127, found: 398.2117.

(6,6-Dimethylbicyclo[3.1.1]hept-2-en-3-yl)methyl-2,2-dimethyl-5-phenyl-3,4-dihydro-2*H*-pyrrole-3-carboxylate (3p)

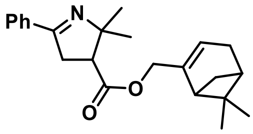

3p

Synthesized following General Procedure F using: **1a** (37.80 mg, 0.20 mmol, 1.00 equiv.), and (6,6-dimethylbicyclo[3.1.1]hept-2-en-2-yl)methyl acrylate (0.04 g, 2.00 mmol, 10.00 equiv.). Purified by flash column chromatography (SiO_2; 100:0 to 85:15 pentane/EtOAc) to afford **3p** (11.00 mg, 0.09 mmol, 16%) as a colorless oil. (d.r. 67:33)

TLC: R_f = 0.70 (90:10 pentane/EtOAc).

NMR Spectroscopy (*see spectra*):

^{1}H NMR (400 MHz, $CDCl_3$): δ_H 7.93 – 7.67 (m, 2H), 7.47 – 7.37 (m, 3H), 5.61 (tq, *J* = 2.9, 1.5 Hz, 1H), 4.61 – 4.43 (m, 2H), 3.51 (dd, *J* = 17.1, 9.0 Hz, 1H), 3.17 (ddd, *J* = 17.1, 9.2, 2.4 Hz, 1H), 3.03 (td, *J* = 9.1, 3.7 Hz, 1H), 2.46 – 2.34 (m, 2H), 2.34 – 2.22 (m, 3H), 2.19 – 2.14 (m, 1H), 1.58 (s, 3H), 1.30 (d, *J* = 2.9 Hz, 3H), 1.17 (d, *J* = 3.1 Hz, 3H), 0.85 (d, *J* = 1.2 Hz, 3H) ppm;

^{13}C NMR (101 MHz, $CDCl_3$): δ_C 172.5, 172.5, 168.4, 142.7, 142.6, 133.8, 130.6, 128.3, 127.6, 122.1, 122.0, 74.9, 74.9, 67.5, 67.4, 52.7, 52.7, 43.6, 43.6, 40.5, 37.9, 37.9, 37.7, 37.7, 31.3, 31.3, 31.1, 31.1, 29.8, 26.0, 23.5, 20.9, 20.9 ppm.

HRMS (ESI$^+$): m/z calc'd for $C_{23}H_{29}NO_2Na$ $[M+Na]^+$: 374.2091, found: 374.2087.

Ethyl-2,2-dimethyl-5-phenyl-4-(trifluoromethyl)-3,4-dihydro-2*H*-pyrrole-3-carboxylate (3q)

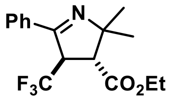

3q

Synthesized following **General Procedure F** using: **1a** (18.90 mg, 0.10 mmol, 1.00 equiv.), and ethyl-4,4,4-trifluorobut-2-enoate (1.00 mmol, 10.00 equiv.). Purified by flash column chromatography (SiO_2; 100:0 to 90:10 pentane/EtOAc) to afford **3q** (21.00 mg, 0.07 mmol, 67%) as a white solid. (d.r. >95:5)

TLC: R_f = 0.82 (7:3 pentane/EtOAc).

NMR Spectroscopy (*see spectra*):

^{1}H NMR (400 MHz, $CDCl_3$): δ_H 7.72 – 7.64 (m, 2H), 7.46 – 7.37 (m, 3H), 4.80 (qd, *J* = 9.1, 7.6 Hz, 1H), 4.35 – 4.17 (m, 2H), 3.21 (d, *J* = 7.6 Hz, 1H), 1.61 (s, 3H), 1.34 (t, *J* = 7.1 Hz, 3H), 1.25 (s, 3H) ppm;

^{13}C NMR (101 MHz, $CDCl_3$): δ_C 171.2, 163.5, 133.6, 130.7, 125.3 (q, *J* = 279.1 Hz), 128.4, 128.2, 74.8, 61.7, 56.7 (q, *J* = 28.1 Hz), 54.6 (d, *J* = 1.5 Hz), 30.4, 24.9, 14.4 ppm;

^{19}F NMR (376 MHz, $CDCl_3$): δ_F -65.59 ppm.

HRMS (ESI$^+$): m/z calc'd for $C_{16}H_{19}NO_2F_3$ $[M+H]^+$: 314.1362, 315.1395, found: 314.1357, 315.1392.

X-ray (*see data*)

Note: The relative stereochemistry was assigned according to the X-ray structure.

2,2-Dimethyl-5-phenyl-4-(trifluoromethyl)-3,4-dihydro-2*H*-pyrrol-3-yl)(morpholino)methanone (3s)

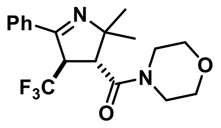

3s

Synthesized following **General Procedure F** using: **1a** (37.80 mg, 0.20 mmol, 1.00 equiv.), and (*Z*)-4,4,4-trifluoro-1-morpholinobut-2-en-1-one (2.00 mmol, 10.00 equiv.). Purified by flash column chromatography (SiO_2; 100:0 to 40:60 pentane/EtOAc) to afford **3s** (34.00 mg, 0.09 mmol, 48%) as a colorless oil. (d.r. >95:5)

TLC: R_f = 0.28 (6:4 pentane/EtOAc).

NMR Spectroscopy (*see spectra*):

^{1}H NMR (400 MHz, $CDCl_3$): δ_H 7.74 – 7.67 (m, 2H), 7.49 – 7.36 (m, 3H), 5.13 – 5.04 (m, 1H), 3.86 – 3.70 (m, 5H), 3.68 – 3.56 (m, 3H), 3.46 (d, *J* = 6.1 Hz, 1H), 1.57 (s, 3H), 1.28 (s, 3H) ppm;

^{13}C NMR (101 MHz, $CDCl_3$): δ_C 169.3, 163.8, 133.5, 130.8, 128.4, 125.6 (q, *J* = 279.3 Hz), 75.0, 67.1, 66.7, 58.7 (q, *J* = 27.5 Hz), 50.5, 46.9, 43.0, 30.7, 25.4 ppm;

^{19}F NMR (376 MHz, $CDCl_3$): δ_F -65.19 ppm.

HRMS (ESI$^+$): m/z calc'd for $C_{18}H_{21}N_2O_2F_3Na$ $[M+Na]^+$: 377.1447, found: 377.1450.

Note: The relative stereochemistry was assigned in analogy to **3q**.

N-Methoxy-*N*,2,2-trimethyl-5-phenyl-4-(trifluoromethyl)-3,4-dihydro-2*H*-pyrrole-3-carboxamide (3t)

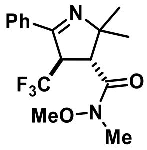

3t

Synthesized following **General Procedure G** using: **1a** (37.80 mg, 0.20 mmol, 1.00 equiv.), and (*E*)-4,4,4-trifluoro-*N*-methoxy-*N*-methylbut-2-enamide (2.00 mmol, 10.00 equiv.). Purified by flash column chromatography (SiO_2; 90:10 to 85:15 pentane/EtOAc) to afford **3t** as a white solid (31 mg, 0.09 mmol, 47%). (d.r. 88:12).

TLC: R_f = 0.34 (85:15 pentane/EtOAc).

NMR Spectroscopy (*see spectra*):

^{1}H NMR (599 MHz, $CDCl_3$): δ_H 7.77 – 7.69 (m, 2H), 7.48 – 7.33 (m, 3H), 4.98 (qd, *J* = 9.8, 5.7 Hz, 1H), 3.75 (s, 3H), 3.54 (d, *J* = 5.7 Hz, 1H), 3.26 (s, 3H), 1.57 (s, 3H), 1.25 (s, 3H). ppm;

^{13}C NMR (126 MHz, $CDCl_3$): δ_C 171.8, 163.2, 133.6, 130.7, 128.5, 128.4, 125.6 (d, *J* = 279.2 Hz), 76.2, 61.3, 57.1 (q, *J* = 27.4 Hz), 51.2, 32.6, 30.6, 25.0 ppm;

^{19}F NMR (377 MHz, $CDCl_3$): δ_F -65.43 ppm.

HRMS (ESI$^+$): m/z calc'd for $C_{16}H_{19}F_3N_2O_2Na$ $[M+Na]^+$: 351.1296, found: 351.1289.

Note: The relative stereochemistry was assigned in analogy to **3q**.

N,N-Diallyl-2,2-dimethyl-5-phenyl-4-(trifluoromethyl)-3,4-dihydro-2*H*-pyrrole-3-carboxamide (3u)

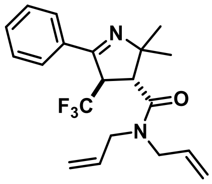

3u

Synthesized following **General Procedure F** using: **1a** (37.80 mg, 0.20 mmol, 1.00 equiv.), and (*E*)-*N,N*-diallyl-

4,4,4-trifluorobut-2-enamide (2.00 mmol, 10.00 equiv.). The reaction mixture was irradiated for 4 h. Purified by flash column chromatography (SiO_2; 9:1 pentane/EtOAc) to afford **3u** (25 mg, 0.07 mmol, 35%) as a colorless oil. (d.r. >95:5). (d^1 = major diastereomer, d^2 = minor diastereomer)

TLC: R_f = 0.3 (9:1 pentane/EtOAc).

NMR Spectroscopy (*see spectra*):

Major diastereomer (d^1):

^{1}H NMR (599 MHz, $CDCl_3$): δ_H 7.76 – 7.68 (m, 2H), 7.48 – 7.30 (m, 3H), 5.86 – 5.70 (m, 2H), 5.33 – 5.26 (m, 1H), 5.25 – 5.15 (m, 3H), 5.04 (qd, J = 9.9, 5.1 Hz, 1H), 4.50 (dd, J = 14.8, 4.7 Hz, 1H), 4.13 (m, 1H), 3.86 (dd, J = 17.3, 5.5 Hz, 1H), 3.59 (dd, J = 14.8, 7.4 Hz, 1H), 3.42 (d, J = 5.0 Hz, 1H), 1.56 (s, 3H), 1.32 (s, 3H) ppm;

^{13}C NMR (126 MHz, $CDCl_3$): δ_C 170.9, 163.7, 133.6, 132.8, 132.1, 130.7, 128.4, 128.3, 125.5 (d, J = 279.4 Hz), 118.4, 117.9, 75.4, 59.1 (q, J = 27.4 Hz), 50.9, 49.4, 48.8, 30. 8, 25.4 ppm;

^{19}F NMR (377 MHz, $CDCl_3$): δ_F -64.88 ppm.

Minor diastereomer (d^2):

^{1}H NMR (599 MHz, $CDCl_3$): δ_H 7.57 (m, 2H), 7.48 – 7.30 (m, 3H), 5.86 – 5.70 (m, 2H), 5.33 – 5.26 (m, 2H), 5.25 – 5.15 (m, 2H), 5.04 (m, 1H), 4.67 (d, J = 9.6 Hz, 1H), 4.13 (m, 1H), 4.05 – 3.99 (m, 2H), 3.31 (s, 1H), 1.63 (s, 3H), 1.32 (s, 3H) ppm;

^{13}C NMR {^{1}H, ^{19}F} (126 MHz, $CDCl_3$): δ_C 170.0, 165.7, 133.7, 132.9, 132.3, 130.6, 128.5, 127.6, 127.0, 118.9, 118.4, 72.9, 56.9, 52.4, 50.2, 48.4, 30.3, 23.7 ppm;

^{19}F NMR (377 MHz, $CDCl_3$): δ_F -64.06 ppm.

HRMS (ESI^+): m/z calc'd for $C_{20}H_{23}F_3N_2ONa$ $[M+Na]^+$: 387.1660, found: 387.1649.

Note: The relative stereochemistry of the major diastereomer was assigned in analogy to **3q**.

Methyl-3-(2-methoxy-2-oxoethyl)-2,2-dimethyl-5-phenyl-3,4-dihydro-2*H*-pyrrole-3-carboxylate (3w)

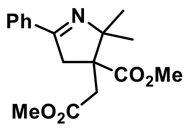

3w

Synthesized following **General Procedure F** using: **1a** (37.80 mg, 0.20 mmol, 1.00 equiv.), and dimethyl 2-methylenesuccinate (2.00 mmol, 10.00 equiv.). Purified by flash column chromatography (SiO_2; 100:0 to 90:10 pentane/EtOAc) to afford **3w** (17.00 mg, 0.05 mmol, 27%) as a colorless oil.

TLC: R_f = 0.21 (8:2 pentane/EtOAc).

NMR Spectroscopy (*see spectra*):

^{1}H NMR (400 MHz, $CDCl_3$): δ_H 7.85 – 7.77 (m, 2H), 7.47 – 7.38 (m, 3H), 3.97 (dd, J = 17.5, 1.3 Hz, 1H), 3.74

(s, 3H), 3.67 (s, 3H), 3.12 (dd, J = 16.8, 1.4 Hz, 1H), 2.95 (d, J = 17.5 Hz, 1H), 2.43 (d, J = 16.8 Hz, 1H), 1.42 (s, 3H), 1.16 (s, 3H) ppm;

^{13}C NMR (101 MHz, $CDCl_3$): δ_C 174.1, 172.5, 169.3, 134.3, 130.9, 128.6, 127.7, 75.7, 56.6, 52.3, 52.0, 43.8, 38.8, 25.2, 24.0 ppm.

HRMS (ESI$^+$): m/z calc'd for $C_{17}H_{22}NO_4$ [M+H]$^+$: 304.1543, found: 304.1541.

6,6-Dimethyl-8-phenyl-2-oxa-7-azaspiro[4.4]non-7-en-1-one (3x)

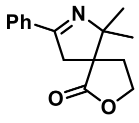

3x

Synthesized following **General Procedure F** using: **1a** (37.80 mg, 0.20 mmol, 1.00 equiv.), and 3-methylenedihydrofuran-2(3*H*)-one (2.00 mmol, 10.00 equiv.). Purified by flash column chromatography (SiO_2; 100:0 to 90:10 pentane/EtOAc) to afford **3x** (20.00 mg, 0.08 mmol, 42%) as a colorless oil.

TLC: R_f = 0.10 (7:3 pentane/EtOAc).

NMR Spectroscopy (*see spectra*):

^{1}H NMR (400 MHz, $CDCl_3$): δ_H 7.82 – 7.76 (m, 2H), 7.49 – 7.34 (m, 3H), 4.32 (td, J = 9.2, 8.4 Hz, 1H), 4.24 (td, J = 9.2, 6.6 Hz, 1H), 3.59 (d, J = 16.8 Hz, 1H), 3.06 (d, J = 16.8 Hz, 1H), 2.61 (ddd, J = 13.4, 6.6, 3.5 Hz, 1H), 2.30 (dt, J = 13.5, 8.9 Hz, 1H), 1.45 (s, 3H), 1.39 (s, 3H) ppm;

^{13}C NMR (101 MHz, $CDCl_3$): δ_C 179.1, 168.1, 133.9, 130.9, 128.6, 127.8, 75.6, 65.7, 54.0, 46.1, 32.3, 25.4, 25.4 ppm.

HRMS (ESI$^+$): m/z calc'd for $C_{15}H_{18}NO_2$ [M+H]$^+$: 244.1332, found: 244.1332.

Diethyl 2,2-dimethyl-5-phenyl-2*H*-pyrrole-3,4-dicarboxylate (3y)

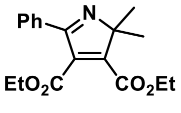

3y

Synthesized following **General Procedure F** using: **1a** (37.80 mg, 0.20 mmol, 1.00 equiv.), and diethyl but-2-ynedioate (1.00 mmol, 10.00 equiv.). The reaction mixture was irradiated for 8 h. Purified by flash column chromatography (SiO_2; 100:0 to 90:10 pentane/EtOAc) to afford **3y** (30.00 mg, 0.10 mmol, 48%) as a colorless oil.

TLC: R_f = 0.48 (8:2 pentane/EtOAc).

NMR Spectroscopy (*see spectra*):

^{1}H NMR (400 MHz, $CDCl_3$): δ_H 7.74 – 7.70 (m, 2H), 7.46 – 7.38 (m, 3H), 4.33 (dq, *J* = 12.7, 7.1 Hz, 4H), 1.33 (dt, *J* = 15.8, 7.2 Hz, 6H) ppm;

^{13}C NMR (101 MHz, $CDCl_3$): δ_C 165.7, 165.4, 161.5, 160.1, 137.6, 133.4, 130.6, 128.7, 127.8, 79.5, 62.1, 61.4, 23.1, 14.2, 14.1 ppm.

HRMS (ESI^+): m/z calc'd for $C_{18}H_{22}NO_4$ $[M+H]^+$: 316.1543, found: 316.1541.

3-Hexyl-5,5-dimethyl-2-phenyl-3,5-dihydro-4*H*-imidazol-4-one (3z)

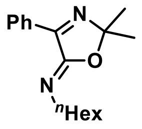

3z

Synthesized following **General Procedure F** using: **1a** (18.90 mg, 0.10 mmol, 1.00 equiv.), and 1-isocyanatohexane (1.00 mmol, 10.00 equiv.). The reaction mixture was irradiated for 8 h. Purified by flash column chromatography (SiO_2; 100:0 to 95:5 pentane/EtOAc) to afford **3z** (9.00 mg, 0.03 mmol, 34%) as a colorless oil.

TLC: R_f = 0.53 (50:1 pentane/EtOAc).

NMR Spectroscopy (*see spectra*):

^{1}H NMR (400 MHz, $CDCl_3$): δ_H 8.41 – 8.28 (m, 2H), 7.53 – 7.40 (m, 3H), 3.44 (t, *J* = 7.2 Hz, 1H), 1.69 – 1.64 (m, 2H), 1.62 (s, 6H), 1.44 – 1.37 (m, 2H), 1.35 – 1.29 (m, 4H), 0.90 (t, *J* = 6.8 Hz, 3H) ppm;

^{13}C NMR (101 MHz, $CDCl_3$): δ_C 158.5, 155.8, 134.5, 131.4, 129.3, 128.4, 106.0, 48.7, 31.8, 30.8, 27.4, 27.0, 22.8, 14.2 ppm.

HRMS (ESI^+): m/z calc'd for $C_{17}H_{24}N_2ONa$ $[M+Na]^+$: 295.1781, found: 295.1781.

3-(4-Fluorophenyl)-2,2-dimethyl-5-phenyl-2,3-dihydro-4*H*-imidazol-4-one (3aa)

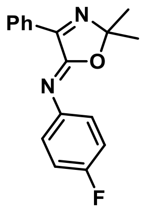

3aa

Synthesized following **General Procedure F** using: **1a** (18.90 mg, 0.10 mmol, 1.00 equiv.), and 1-fluoro-4-isocyanatobenzene (1.00 mmol, 10.00 equiv.). The reaction mixture was irradiated for 8 h. Purified by flash column chromatography (SiO_2; 100:0 to 95:5 pentane/EtOAc) to afford **3aa** (15.00 mg, 0.05 mmol, 52%) as a white solid.

TLC: R_f = 0.42 (50:1 pentane/EtOAc).

NMR Spectroscopy (see spectra):

^{1}H NMR (400 MHz, $CDCl_3$): δ_H 8.51 (dt, *J* = 6.9, 1.5 Hz, 2H), 7.57 – 7.46 (m, 3H), 7.45 – 7.34 (m, 2H), 7.09 – 7.00 (m, 2H), 1.69 (s, 6H) ppm;

^{13}C NMR (151 MHz, $CDCl_3$): δ_C 161.2 – 159.5 (d, *J* = 243.6 Hz), 159.1, 154.2, 154.2, 141.9 – 141.8 (d, *J* = 3.1 Hz), 131.7, 130.2, 129.5, 128.5, 125.8 (d, *J* = 8.2 Hz), 115.5 (d, *J* = 22.3 Hz), 108.2, 26.8 ppm;

^{19}F NMR (376 MHz, $CDCl_3$): δ_F -117.51 ppm.

HRMS (ESI$^+$): m/z calc'd for $C_{17}H_{16}N_2OF$ $[M+H]^+$: 283.1241, found: 283.1242.

X-ray (see data)

3-(2-Chloroethyl)-2,2-dimethyl-5-phenyl-2,3-dihydro-4*H*-imidazol-4-one (3ab)

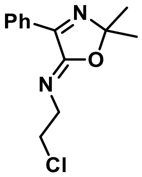

3ab

Synthesized following **General Procedure F** using: **1a** (18.90 mg, 0.10 mmol, 1.00 equiv.), and 1-chloro-2-isocyanatoethane (1.00 mmol, 10.00 equiv.). The reaction mixture was irradiated for 8 h. Purified by flash column chromatography (SiO_2; 100:0 to 95:5 pentane/EtOAc) to afford **3ab** (11.00 mg, 0.04 mmol, 44%) as a colorless oil.

TLC: R_f = 0.41 (50:1 pentane/EtOAc).

NMR Spectroscopy (see spectra):

^{1}H NMR (400 MHz, $CDCl_3$): δ_H 8.45 – 8.37 (m, 2H), 7.55 – 7.40 (m, 3H), 3.77 (bs, 4H), 1.63 (s, 6H) ppm;

^{13}C NMR (101 MHz, $CDCl_3$): δ_C 158.5, 157.6, 135.0, 132.0, 129.6, 128.8, 107.3, 50.7, 44.9, 27.2 ppm.

HRMS (ESI$^+$): m/z calc'd for $C_{13}H_{15}N_2OClNa$ $[M+Na]^+$: 273.0765, 275.0739, found: 273.0764, 275.0735.

3-(4-Fluorophenyl)-2,2-dimethyl-5-phenyl-2,3-dihydro-4*H*-imidazol-4-one (3ac)

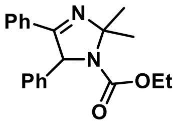

3ac

Synthesized following **General Procedure F** using: **1a** (18.90 mg, 0.10 mmol, 1.00 equiv.), and ethyl benzylidenecarbamate (1.00 mmol, 10.00 equiv.). The reaction mixture was irradiated for 8 h. Purified by flash column chromatography (SiO_2; 100:0 to 9:1 pentane/EtOAc) to afford **3ac** (28.00 mg, 0.09 mmol, 88%) as a colorless oil.

TLC: R_f = 0.40 (9:1 pentane/EtOAc).

NMR Spectroscopy (see spectra):

61:39 mixture of rotamers

^{1}H NMR (400 MHz, $CDCl_3$): δ_H 7.69 – 7.63 (m, 4H), 7.38 (d, J = 7.6 Hz, 1H), 7.35 – 7.19 (m, 15H), 6.10 (s, 1H), 5.99 (s, 1H), 4.21 – 4.05 (m, 2H), 3.98 (q, J = 7.1 Hz, 2H), 1.91 (s, 3H), 1.84 (s, 3H), 1.78 (s, 3H), 1.73 (s, 3H), 1.27 (t, J = 7.1 Hz, 3H), 1.07 (t, J = 7.1 Hz, 3H) ppm;

^{13}C NMR (151 MHz, $CDCl_3$): δ_C 165.0,164.9, 153.6, 152.9, 139.0, 138.4, 131.7, 131.6, 131.0, 130.9, 129.0, 128.9, 128.7, 128.6, 128.6, 128.5, 128.5, 128.2, 128.1, 90.7, 90.1, 70.9, 70.2, 61.3, 60.9, 28.1, 27.6, 27.3, 26.5, 14.6, 14.3 ppm.

HRMS (ESI$^+$): m/z calc'd for $C_{20}H_{22}N_2O_2Na$ $[M+Na]^+$: 345.1579, found: 345.1572.

3-(4-Fluorophenyl)-2,2-dimethyl-5-phenyl-2,3-dihydro-4*H*-imidazol-4-one (3ad)

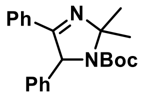

3ad

Synthesized following General Procedure F using: **1a** (37.80 mg, 0.20 mmol, 1.00 equiv.), and ethyl benzylidenecarbamate (2.00 mmol, 10.00 equiv.). The reaction mixture was irradiated for 8 h. Purified by flash column chromatography (SiO_2; 100:0 to 9:1 pentane/EtOAc) to afford **3ad** (46.00 mg, 0.13 mmol, 65%) as a colorless oil.

TLC: R_f = 0.37 (9:1 pentane/EtOAc).

NMR Spectroscopy (see spectra):

64:36 mixture of rotamers

^{1}H NMR (400 MHz, $CDCl_3$): δ_H 7.66 – 7.63 (m, 3H), 7.51 – 7.43 (m, 2H), 7.37 – 7.28 (m, 13H), 7.24 – 7.19 (m, 2H), 6.08 (s, 1H), 5.92 (s, 1H), 1.93 (s, 3H), 1.83 (s, 3H), 1.76 (s, 3H), 1.72 (s, 3H), 1.59 (s, 9H), 1.24 (s, 9H) ppm;

^{13}C NMR (151 MHz, $CDCl_3$): δ_C 165.2, 165.0, 152.7, 152.1, 139.6, 138.8, 132.1, 131.9, 130.7, 130.7, 128.8, 128.8, 128.7, 128.5, 128.5, 128.4, 128.3, 127.9, 90.4, 89.8, 80.4, 80.1, 77.2, 70.7, 70.6, 28.6, 28.4, 28.2, 27.7, 27.5, 26.4 ppm.

HRMS (ESI$^+$): m/z calc'd for $C_{22}H_{26}N_2O_2Na$ $[M+Na]^+$: 373.1887, found: 373.1883.

10,13-Dimethyl-17-(6-methylheptan-2-yl)-2,3,4,7,8,9,10,11,12,13,14,15,16,17-tetradecahydro-1*H*-cyclopenta[a]phenanthren-3-yl-2,2-dimethyl-5-phenyl-3,4-dihydro-2*H*-pyrrole-3-carboxylate (3ae)

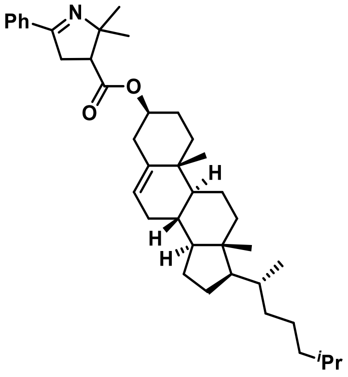

3ae

Synthesized following **General Procedure F** using: **1a** (37.80 mg, 0.20 mmol, 1.00 equiv.), and (3*S*,8*S*,9*S*,10*R*,13*R*,14*S*,17*R*)-10,13-dimethyl-17-((*R*)-6-methylheptan-2-yl)-2,3,4,7,8,9,10,11,12,13,14,15,16,17-tetradecahydro-1*H*-cyclopenta[a]phenanthren-3-yl acrylate (2.00 mmol, 5.00 equiv.) and dichloromethane as a solvent. Purified by flash column chromatography (SiO_2; 100:0 to 95:5 pentane/EtOAc) to afford **3ae** (12.00 mg, 0.02 mmol, 10%) as a colorless oil.

TLC: R_f = 0.70 (90:10 pentane/EtOAc).

NMR Spectroscopy (*see spectra*):

^{1}H NMR (400 MHz, CD_2Cl_2): δ_H 7.78 – 7.66 (m, 2H), 7.41 – 7.24 (m, 3H), 5.32 – 5.31 (m, 1H), 4.61 – 4.50 (m, 1H), 3.36 (dd, *J* = 17.0, 8.9 Hz, 1H), 3.06 (dd, *J* = 17.0, 9.2 Hz, 1H), 2.89 (t, *J* = 9.1 Hz, 1H), 2.26 (t, *J* = 3.7 Hz, 1H), 1.96 – 1.87 (m, 2H), 1.85 – 1.78 (m, 2H), 1.46 (d, *J* = 13.1 Hz, 10H), 1.18 (s, 10H), 1.05 (d, *J* = 2.8 Hz, 4H), 0.96 (s, 3H), 0.84 (d, *J* = 6.6 Hz, 3H), 0.78 (dd, *J* = 6.6, 1.6 Hz, 9H), 0.61 (s, 3H) ppm;

^{13}C NMR (101 MHz, CD_2Cl_2): δ_C 172.5, 168.4, 140.2, 140.2, 134.8, 130.8, 128.8, 128.0, 123.0, 123.0, 75.4, 74.7, 57.2, 56.6, 50.5, 42.7, 40.2, 39.9, 38.6, 38.2, 37.4, 37.0, 36.6, 36.2, 32.3, 32.3, 30.3, 30.1, 28.6, 28.4, 28.3, 24.7, 24.2, 23.9, 23.0, 22.7, 21.4, 19.5, 18.9, 12.0 ppm.

HRMS (ESI$^+$): m/z calc'd for $C_{40}H_{60}NO_2$ $[M+H]^+$: 586.4619, found: 586.4621.

(*Z*)-3,7-Dimethylocta-2,6-dien-1-yl-2,2-dimethyl-5-phenyl-3,4-dihydro-2*H*-pyrrole-3-carboxylate (3af)

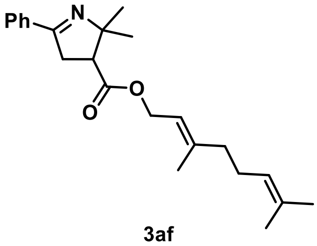

3af

Synthesized following **General Procedure F** using: **1a** (37.80 mg, 0.20 mmol, 1.00 equiv.), and (*Z*)-3,7-dimethylocta-2,6-dien-1-yl acrylate (2.00 mmol, 10.00 equiv.). Purified by flash column chromatography (SiO_2; 100:0 to 85:15 pentane/EtOAc) to afford **3af** (15.00 mg, 0.04 mmol, 21%) as a colorless oil.

TLC: R_f = 0.30 (90:10 pentane/EtOAc).

NMR Spectroscopy (*see spectra*):

^{1}H NMR (400 MHz, $CDCl_3$): δ_H 7.77 – 7.71 (m, 2H), 7.38 – 7.31 (m, 3H), 5.32 (m, 1H), 5.02 (m, 1H), 4.68 – 4.52 (m, 2H), 3.44 (dd, *J* = 17.0, 9.0 Hz, 1H), 3.10 (dd, *J* = 17.0, 9.2 Hz, 1H), 2.95 (t, *J* = 9.1 Hz, 1H), 2.04 – 1.97 (m, 4H), 1.66 (d, *J* = 1.3 Hz, 3H), 1.61 (d, *J* = 1.4 Hz, 3H), 1.54 – 1.49 (m, 6H), 1.09 (s, 3H) ppm;

^{13}C NMR (101 MHz, $CDCl_3$): δ_C 173.3, 168.9, 143.1, 134.7, 132.3, 131.0, 128.9, 128.1, 124.1, 118.7, 75.7, 62.0, 53.2, 40.0, 38.4, 30.5, 26.7, 26.1, 24.1, 18.2, 16.9 ppm.

.**HRMS** (ESI^+): m/z calc'd for $C_{23}H_{32}NO_2$ $[M+H]^+$: 354.2428, found: 354.2426.

2-(4-(2,2-Dichlorocyclopropyl)phenoxy)-2-methylpropyl 2,2-dimethyl-5-phenyl-4-(trifluoromethyl)-3,4-dihydro-2*H*-pyrrole-3-carboxylate (3ag)

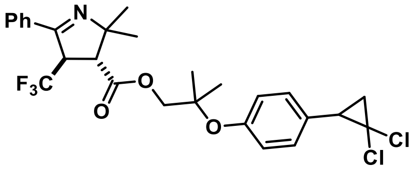

3ag

Synthesized following **General Procedure G** using: **1a** (37.80 mg, 0.20 mmol, 1.00 equiv.), and 2-(4-(2,2-dichlorocyclopropyl)phenoxy)-2-methylpropyl (*E*)-4,4,4-trifluorobut-2-enoate (1.00 mmol, 5.00 equiv.). Reaction mixture was irradiated for 4 h. Purified by flash column chromatography (SiO_2; 95:5 to 9:1 pentane/EtOAc) to afford **3ag** as a colorless oil (68.00 mg, 0.13 mmol, 63%). (d.r. 86:14).

TLC: R_f = 0.36 (9:11 pentane/EtOAc).

NMR Spectroscopy (*see spectra*):

^{1}H NMR (599 MHz, $CDCl_3$): δ_H 7.70 – 7.65 (m, 2H), 7.48 – 7.43 (m, 1H), 7.43 – 7.39 (m, 2H), 7.19 – 7.14 (m, 2H), 7.00 – 6.94 (m, 2H), 4.85 (d, *J* = 1.3 Hz, 1H), 4.29 – 4.18 (m, 2H), 3.30 (dd, *J* = 7.8, 1.5 Hz, 1H), 2.86 (dd, *J* = 10.7, 8.3 Hz, 1H), 1.96 (dd, *J* = 10.7, 7.4 Hz, 1H), 1.81 (dd, *J* = 8.3, 7.4 Hz, 1H), 1.65 (d, *J* = 1.7 Hz, 3H), 1.38 (s, 6H), 1.27 (s, 3H) ppm;

^{13}C NMR (126 MHz, $CDCl_3$): δ_C 171.0, 163.5, 154.1, 133.5, 130.8, 130.4, 129.8, 128.4, 128.2, 125.3 (d, *J* = 279.0 Hz), 123.9, 78.5, 74.8, 71.1, 60.9, 56.8 (q, *J* = 28.2 Hz), 54.8, 35.1, 30.5, 26.0, 25.0, 24.2 ppm;

^{19}F NMR (564 MHz, $CDCl_3$): δ_F -65.51 ppm.

HRMS (ESI^+): m/z calc'd for $C_{27}H_{28}Cl_2F_3NO_3Na$ $[M+Na]^+$: 564.1296, found: 564.1286.

Note: The relative stereochemistry was assigned in analogy to **3q**.

5-(2,5-Dimethylphenoxy)-2,2-dimethylpentyl-2,2-dimethyl-5-phenyl-4-(trifluoromethyl)-3,4-dihydro-2*H*-pyrrole-3-carboxylate (3ah)

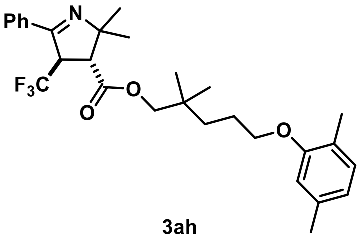

3ah

Synthesized following **General Procedure F** using: **1a** (37.80 mg, 0.20 mmol, 1.00 equiv.), and 5-(2,5-dimethylphenoxy)-2,2-dimethylpentyl acrylate (2.00 mmol, 10.00 equiv.). Purified by flash column chromatography (SiO_2; 100:0 to 85:15 pentane/EtOAc) to afford **3ah** (48.00 mg, 0.10 mmol, 48%) as a colorless oil. (d.r. >95:5)

TLC: R_f = 0.60 (90:10 pentane:EtOAc).

NMR Spectroscopy (*see spectra*):

^{1}H NMR (400 MHz, $CDCl_3$): δ_H 7.69 – 7.67 (m, 2H), 7.48 – 7.39 (m, 3H), 7.01 (d, *J* = 7.5 Hz, 1H), 6.68 – 6.63 (m, 2H), 4.82 (qd, *J* = 9.0, 7.7 Hz, 1H), 4.03 (d, *J* = 10.8 Hz, 1H), 3.95 (t, *J* = 6.3 Hz, 2H), 3.89 (d, *J* = 10.7 Hz, 1H), 3.25 (d, *J* = 7.8 Hz, 1H), 2.32 (s, 3H), 2.18 (s, 3H), 1.80 (m, 2H), 1.64 (s, 3H), 1.57 – 1.48 (m, 2H), 1.25 (s, 3H), 1.03 (d, *J* = 3.0 Hz, 6H) ppm;

^{13}C NMR (126 MHz, $CDCl_3$): δ_C 171.1, 163.6, 157.0, 136.5, 133.2, 130.7, 130.3, 128.3, 128.1, 125.2 (d, *J* = 279.7 Hz), 123.6, 120.8, 112.0, 74.5, 73.7, 68.2, 56.5 (d, *J* = 27.8 Hz), 54.7, 35.6, 33.6, 30.3, 29.7, 24.9, 24.3, 24.3, 24.1, 21.4, 15.7 ppm;

^{19}F NMR (376 MHz, $CDCl_3$): δ_F -65.55 ppm.

HRMS (ESI^+): m/z calc'd for $C_{29}H_{36}NO_3F_3Na$ $[M+Na]^+$: 526.2540, found: 526.2541.

Note: The relative stereochemistry was assigned in analogy to **3q**.

5-(2-Fluorophenyl)-*N*-methoxy-*N*,2,2-trimethyl-4-(trifluoromethyl)-3,4-dihydro-2*H*-pyrrole-3-carboxamide (6a)

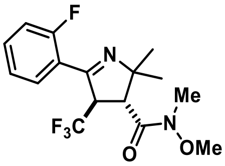

6a

Synthesized following **General Procedure G** using: **1d** (41.40 mg, 0.20 mmol, 1.00 equiv.) and stirring under blue light irradiation for 4 h. Purified by flash column chromatography (SiO_2; 85:15 pentane/EtOAc) to afford **6a** (31.40 mg, 0.09 mmol, 46%) as a yellow solid. (d.r. >95:5)

TLC: R_f = 0.20 (80:20 pentane/EtOAc).

NMR Spectroscopy (*see spectra*):

^{1}H NMR (400 MHz, $CDCl_3$): δ_H 7.63 (td, *J* = 7.4, 1.8 Hz, 1H), 7.42 (dddd, *J* = 8.3, 7.2, 5.2, 1.8 Hz, 1H), 7.18 (td, *J* = 7.6, 1.1 Hz, 1H), 7.10 (ddd, *J* = 10.7, 8.3, 1.1 Hz, 1H), 5.09 (qdd, *J* = 9.9, 6.4, 1.7 Hz, 1H), 3.75 (s, 3H), 3.51 (d, *J* = 6.4 Hz, 1H), 3.27 (s, 3H), 1.58 (s, 3H), 1.26 (s, 3H) ppm;

^{19}F NMR (470 MHz, $CDCl_3$): δ_F -67.49, -112.12 ppm;

^{13}C NMR (126 MHz, $CDCl_3$): δ_C 171.4, 160.9 (d, *J* = 251.7 Hz), 160.3, 132.3 (d, *J* = 7.2 Hz), 130.7 (d, *J* = 3.2 Hz), 125.6 (q, *J* = 279.2 Hz), 124.4 (d, *J* = 3.5 Hz), 122.1 (d, *J* = 14.4 Hz), 116.0 (d, *J* = 21.7 Hz), 76.1, 61.3, 58.8 (qd, *J* = 27.7, 4.4 Hz), 50.9, 32.6, 30.6, 24.9 ppm.

HRMS (ESI$^+$): m/z calc'd for $C_{16}H_{18}N_2$ $[M+Na]^+$: 369.1197, found: 369.1192

Note: The relative stereochemistry was assigned in analogy to **6c**.

5-(3-Fluorophenyl)-*N*-methoxy-*N*,2,2-trimethyl-4-(trifluoromethyl)-3,4-dihydro-2*H*-pyrrole-3-carboxamide (6b)

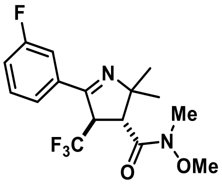

6b

Synthesized following **General Procedure G** using: **1c** (41.40 mg, 0.20 mmol, 1.00 equiv.) and stirring under blue light irradiation for 4 h. Purified by flash column chromatography (SiO_2; 80:20 pentane/EtOAc) to afford **6b** (32.00 mg, 0.09 mmol, 46%) as a white solid. (d.r. >95:5)

TLC: R_f = 0.18 (80:20 pentane:EtOAc).

NMR Spectroscopy (*see spectra*):

^{1}H NMR (599 MHz, $CDCl_3$): δ_H 7.51 – 7.43 (m, 2H), 7.36 (dddd, *J* = 8.2, 7.7, 5.7, 0.4 Hz, 1H), 7.13 (tdd, *J* = 8.4, 2.6, 1.0 Hz, 1H), 4.92 (qd, *J* = 9.8, 5.6 Hz, 1H), 3.75 (s, 3H), 3.52 (d, J = 5.5 Hz, 1H), 3.25 (s, 3H), 1.55 (s, 3H), 1.24 (s, 3H) ppm;

^{19}F NMR (564 MHz, $CDCl_3$): δ_F -65.47, -112.90 ppm;

^{13}C NMR (126 MHz, $CDCl_3$): δ_C 171.7, 162.6 (d, *J* = 246.7 Hz), 162.0, 135.7 (d, *J* = 7.5 Hz), 130.0 (d, *J* = 8.1 Hz), 125.6 (q, *J* = 279.5 Hz), 124.2, 117.7 (d, *J* = 21.1 Hz), 115.5 (d, *J* = 23.1 Hz), 76.4, 61.3, 57.3 (q, *J* = 27.6 Hz), 51.2, 32.7, 30.5, 25.0 ppm.

HRMS (ESI$^+$): m/z calc'd for $C_{16}H_{18}N_2$ $[M+Na]^+$: 369.1197, found: 369.1192.

Note: The relative stereochemistry was assigned in analogy to **6c**.

5-(4-Fluorophenyl)-*N*-methoxy-*N*,2,2-trimethyl-4-(trifluoromethyl)-3,4-dihydro-2*H*-pyrrole-3-carboxamide (6c)

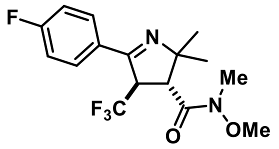

6c

Synthesized following **General Procedure G** using: **1b** (41.40 mg, 0.20 mmol, 1.00 equiv.) and stirring under blue light irradiation for 3 h. Purified by flash column chromatography (SiO_2; 80:20 pentane/EtOAc) to afford **6c** (29.20 mg, 0.08 mmol, 42%) as a white solid. (d.r. >95:5)

TLC: R_f = 0.18 (80:20 pentane/EtOAc).

NMR Spectroscopy (*see spectra*):

^{1}H NMR (599 MHz, $CDCl_3$): δ_H 7.79 – 7.68 (m, 2H), 7.13 – 7.00 (m, 2H), 4.94 (qd, *J* = 9.8, 5.6 Hz, 1H), 3.75 (s, 3H), 3.53 (d, *J* = 5.6 Hz, 1H), 3.26 (s, 3H), 1.55 (s, 3H), 1.25 (s, 3H) ppm;

^{19}F NMR (564 MHz, $CDCl_3$): δ_F -65.4%, -109.74 ppm;

^{13}C NMR (151 MHz, $CDCl_3$): δ_C 171.6, 164.2 (d, *J* = 250.6 Hz), 161.8, 130.4 (d, *J* = 8.7 Hz), 129.6 (d, *J* = 3.5 Hz), 125.5 (q, *J* = 279,2 Hz), 115.3 (d, *J* = 21.8 Hz), 76.0, 61.1, 57.0 (q, *J* = 27.5 Hz), 51.1, 32.5, 30.4, 24.8 ppm.

HRMS (ESI$^+$): m/z calc'd for $C_{16}H_{18}N_2$ $[M+Na]^+$: 369.1197, found: 369.1192.

X-ray (*see data*)

Note: The relative stereochemistry was assigned according to the X-ray structure.

***N*-Methoxy-*N*,2,2-trimethyl-4-(trifluoromethyl)-5-(4-(trifluoromethyl)phenyl)-3,4-dihydro-2*H*-pyrrole-3-carboxamide (6d)**

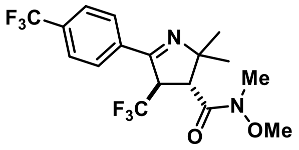

6d

Synthesized following **General Procedure G** using: **1e** (51.40 mg, 0.20 mmol, 1.00 equiv.) and stirring under blue light irradiation for 3 h. Purified by flash column chromatography (SiO_2; 85:15 pentane/EtOAc) to afford **6d** (45.50 mg, 0.12 mmol, 60%) as a white solid. (d.r. >95:5)

TLC: R_f = 0.20 (85:15 pentane:EtOAc).

NMR Spectroscopy (*see spectra*):

^{1}H NMR (500 MHz, $CDCl_3$): δ_H 7.85 (d, *J* = 8.1 Hz, 2H), 7.69 – 7.64 (m, 2H), 4.99 (qd, *J* = 9.7, 5.7 Hz, 1H), 3.76 (s, 3H), 3.55 (d, *J* = 5.7 Hz, 1H), 3.27 (s, 3H), 1.58 (s, 3H), 1.26 (s, 3H) ppm;

^{19}F NMR (470 MHz, $CDCl_3$): δ_F -62.92, -65.48 ppm;

^{13}C NMR (126 MHz, $CDCl_3$): δ_C 171.6, 162.0, 137.0, 132.5 (q, *J* = 32.7 Hz), 128.9, 125.9 (q, *J* = 232.8 Hz), 125.4 (q, *J* = 3.8 Hz), 124.0 (q, *J* = 226.0 Hz), 76.7, 61.3, 57.3 (q, *J* = 27.7 Hz), 51.3, 32.7, 30.5, 24.9 ppm.

HRMS (ESI$^+$): m/z calc'd for $C_{17}H_{18}N_2O_2F_6$ $[M+Na]^+$: 419.1165, found: 419.1164.

Note: The relative stereochemistry was assigned in analogy to **6c**.

_N_-Methoxy-_N_,2,2-trimethyl-5-(4-(trifluoromethoxzzy)phenyl)-4-(trifluoromethyl)-3,4-dihydro-2_H_-pyrrole-3-carboxamide (6e)

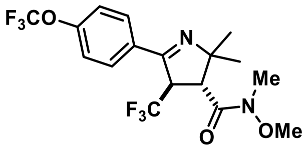

6e

Synthesized following **General Procedure G** using: **1g** (54.60 mg, 0.20 mmol, 1.00 equiv.) and stirring under blue light irradiation for 4 h. Purified by flash column chromatography (SiO_2; 85:15 pentane/EtOAc) to afford **6e** (36.70 mg, 0.09 mmol, 45%) as a white solid. (d.r. >95:5)

TLC: R_f = 0.20 (85:15 pentane/EtOAc).

NMR Spectroscopy (*see spectra*):

^{1}H NMR (500 MHz, $CDCl_3$): δ_H 7.81 – 7.76 (m, 2H), 7.26 – 7.22 (m, 2H), 4.95 (qd, *J* = 9.8, 5.6 Hz, 1H), 3.76 (s, 3H), 3.54 (d, *J* = 5.6 Hz, 1H), 3.27 (s, 3H), 1.56 (s, 4H), 1.25 (s, 3H) ppm;

^{19}F NMR (470 MHz, $CDCl_3$): δ_F -57.73, -65.42 ppm;

^{13}C NMR (126 MHz, $CDCl_3$): δ_C 171.7, 161.8, 151.0, 132.1, 130.2, 125.6 (q, *J* = 278.9 Hz), 120.6, 120.5 (q, *J* = 210.7 Hz), 76.41 61.3, 57.2 (q, *J* = 27.9 Hz), 51.3, 32.7, 30.6, 25.0 ppm.

HRMS (ESI$^+$): m/z calc'd for $C_{17}H_{18}N_2O_2F_6$ $[M+Na]^+$: 435.1114, found: 435.1110.

Note: The relative stereochemistry was assigned in analogy to **6c**.

5-(4-Bromophenyl)-_N_-methoxy-_N_,2,2-trimethyl-4-(trifluoromethyl)-3,4-dihydro-2_H_-pyrrole-3-carboxamide (6f)

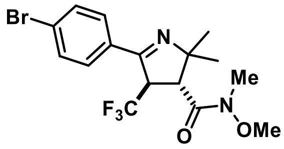

6f

Synthesized following **General Procedure G** using: **1f** (53.60 mg, 0.20 mmol, 1.00 equiv.) and stirring under blue light irradiation for 16 h. Purified by flash column chromatography (SiO_2; 85:15 pentane/EtOAc) to afford

6f (39.90 mg, 0.10 mmol, 50%) as a white solid. (d.r. >95:5)

TLC: R_f = 0.15 (85:15 pentane/EtOAc).

NMR Spectroscopy (*see spectra*):

^{1}H NMR (599 MHz, $CDCl_3$) δ_H 7.62 (d, *J* = 8.4 Hz, 2H), 7.57 – 7.52 (m, 2H), 4.94 (qd, *J* = 9.8, 5.6 Hz, 1H), 3.75 (s, 3H), 3.53 (d, *J* = 5.6 Hz, 1H), 3.26 (s, 3H), 1.56 (s, 3H), 1.25 (s, 3H) ppm;

^{19}F NMR (470 MHz, $CDCl_3$): δ_F -65.43 ppm;

^{13}C NMR (151 MHz, $CDCl_3$) δ_C 171.6, 162.3, 132.3, 131.7, 130.1, 125.6 (q, *J* = 279.5 Hz), 125.4, 76.4, 61.3, 57.1 (q, *J* = 27.6 Hz), 51.2, 32.7, 30.5, 24.9.

HRMS (ESI$^+$): m/z calc'd for $C_{16}H_{18}N_2O_2BrF_3$ $[M+Na]^+$: 429.0396, found: 429.0397.

Note: The relative stereochemistry was assigned in analogy to **6c**.

5-(4-Chlorophenyl)-*N*-methoxy-*N*,2,2-trimethyl-4-(trifluoromethyl)-3,4-dihydro-2*H*-pyrrole-3-carboxamide (6g)

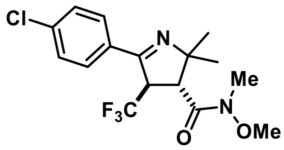

6g

Synthesized following **General Procedure G** using: **1j** (44.70 mg, 0.20 mmol, 1.00 equiv.) and stirring under blue light irradiation for 16 h. Purified by flash column chromatography (SiO_2; 85:15 pentane/EtOAc) to afford **6g** (36.60 mg, 0.10 mmol, 50%) as a white solid. (d.r. >95:5)

TLC: R_f = 0.16 (85:15 pentane/EtOAc).

NMR Spectroscopy (*see spectra*):

^{1}H NMR (500 MHz, $CDCl_3$): δ_H 7.71 – 7.64 (m, 2H), 7.41 – 7.35 (m, 2H), 4.94 (qd, *J* = 9.8, 5.6 Hz, 1H), 3.75 (s, 3H), 3.53 (d, *J* = 5.5 Hz, 1H), 3.26 (s, 3H), 1.56 (s, 3H), 1.25 (s, 3H) ppm;

^{19}F NMR (470 MHz, $CDCl_3$): δ_F -65.41 ppm;

^{13}C NMR (126 MHz, $CDCl_3$) δ_C 171.7, 162.0, 136.9, 132.0, 129.9, 128.7, 125.6 (q, *J* = 279.2 Hz), 76.4, 61.3, 57.2 (q, *J* = 27.5 Hz), 51.3, 32.7, 30.6, 25.0.

HRMS (ESI$^+$): m/z calc'd for $C_{16}H_{18}N_2O_2ClF_3$ $[M+Na]^+$: 385.0901, found: 385.0899.

Note: The relative stereochemistry was assigned in analogy to **6c**.

_N_-Methoxy-_N_,2,2-trimethyl-5-(_p_-tolyl)-4-(trifluoromethyl)-3,4-dihydro-2_H_-pyrrole-3-carboxamide (6h)

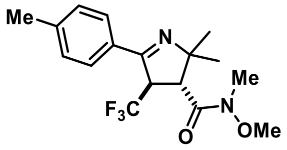

6h

Synthesized following **General Procedure G** using: **1h** (40.60 mg, 0.20 mmol, 1.00 equiv.) and stirring under blue light irradiation for 3 h. Purified by flash column chromatography (SiO_2; 85:15 pentane/EtOAc) to afford **6h** (30.40 mg, 0.09 mmol, 45%) as a white solid. (d.r. >95:5)

TLC: R_f = 0.20 (80:20 pentane/EtOAc).

NMR Spectroscopy (*see spectra*):

^{1}H NMR (500 MHz, $CDCl_3$): δ_H 7.63 (d, *J* = 8.0 Hz, 2H), 7.23 – 7.17 (m, 2H), 4.95 (qd, *J* = 9.8, 5.7 Hz, 1H), 3.75 (s, 3H), 3.52 (d, *J* = 5.7 Hz, 1H), 3.26 (s, 3H), 2.38 (s, 3H), 1.56 (s, 3H), 1.24 (s, 3H) ppm;

^{19}F NMR (470 MHz, $CDCl_3$): δ_F -65.4 ppm;

^{13}C NMR (126 MHz, $CDCl_3$): δ_C 172.0, 162.9, 140.9, 130.8, 129.1, 128.4, 125.8 (q, *J* = 279.2 Hz), 76.0, 61.3, 57.1 (q, *J* = 27.5 Hz), 51.2, 32.7, 30.7, 25.1, 21.6 ppm.

HRMS (ESI$^+$): m/z calc'd for $C_{17}H_{21}N_2O_2F_3$ $[M+Na]^+$: 365.1447, found: 365.1447.

Note: The relative stereochemistry was assigned in analogy to **6c**.

_N_-Methoxy-5-(4-methoxyphenyl)-_N_,2,2-trimethyl-4-(trifluoromethyl)-3,4-dihydro-2_H_-pyrrole-3-carboxamide (6i)

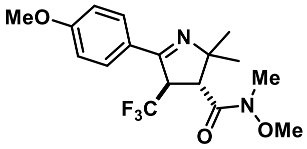

6i

Synthesized following **General Procedure G** using: **1i** (43.80 mg, 0.20 mmol, 1.00 equiv.) and stirring under blue light irradiation for 4 h. Purified by flash column chromatography (SiO_2; 80:20 pentane/EtOAc) to afford **6i** (30.40 mg, 0.09 mmol, 35%) as a white solid. (d.r. >95:5)

TLC: R_f = 0.20 (80:20 pentane/EtOAc).

NMR Spectroscopy (*see spectra*):

^{1}H NMR (500 MHz, $CDCl_3$): δ_H 7.72 – 7.67 (m, 2H), 6.94 – 6.86 (m, 2H), 4.93 (qd, *J* = 9.8, 5.6 Hz, 1H), 3.84 (s, 2H), 3.75 (s, 2H), 3.52 (d, *J* = 5.5 Hz, 1H), 3.26 (s, 3H), 1.55 (s, 3H), 1.24 (s, 3H) ppm;

^{19}F NMR (377 MHz, $CDCl_3$): δ_F -65.33 ppm;

^{13}C NMR (126 MHz, $CDCl_3$): δ_C 172.1, 162.3, 161.6, 130.2, 126.2, 125.8 (q, *J* = 279.2 Hz), 113.7, 75.9, 61.3,

57.0 (q, J = 27.2 Hz), 55.5, 51.3, 32.7, 30.7, 25.2 ppm.

HRMS (ESI$^+$): m/z calc'd for $C_{17}H_{21}N_2O_3F_3$ [M+Na]$^+$: 381.1396, found: 381.1396.

Note: The relative stereochemistry was assigned in analogy to **6c**.

N-Methoxy-*N*-methyl-2-phenyl-3-(trifluoromethyl)-8-oxa-1-azaspiro[4.5]dec-1-ene-4-carboxamide (6l)

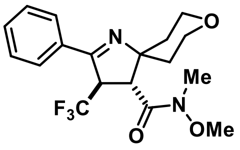

6l

Synthesized following **General Procedure G** using: **1k** (46.00 mg, 0.20 mmol, 1.00 equiv). The reaction mixture was irradiated for 4 h. Purified by flash column chrfomatography (SiO_2; 1:1 pentane/Et_2O) to afford **6l** as a white solid (28.00 mg, 0.08 mmol, 40%). (d.r.= 88:12).

TLC: R_f = 0.3 (1:1 pentane/Et_2O).

NMR Spectroscopy (*see spectra*):

^{1}H NMR (599 MHz, $CDCl_3$): 7.83 – 7.76 (m, 2H), 7.50 – 7.37 (m, 3H), 4.87 (qd, J = 9.8, 5.2 Hz, 1H), 4.15 – 3.99 (m, 2H), 3.87 (dddd, J = 22.4, 11.4, 5.0, 1.9 Hz, 2H), 3.76 (s, 3H), 3.48 (d, J = 5.2 Hz, 1H), 3.27 (s, 3H), 2.29 (td, J = 12.7, 4.9 Hz, 1H), 1.78 (ddd, J = 13.0, 11.8, 4.8 Hz, 1H), 1.67 (dt, J = 13.2, 2.3 Hz, 1H), 1.45 (dq, J = 13.1, 2.3 Hz, 1H) ppm;

^{13}C NMR (126 MHz, $CDCl_3$): δ_C 171.3, 163.4, 133.5, 130.9, 128.7, 128.3, 125.5 (q, J = 279.3 Hz), 76.2, 65.2, 65.1, 61.3, 56.5 (q, J = 27.5 Hz), 51.6, 40.0, 34.7, 32.5 ppm;

^{19}F NMR (377 MHz, $CDCl_3$): δ_F -65.12 ppm.

HRMS (ESI$^+$): m/z calc'd for $C_{18}H_{21}F_3N_2O_3Na$ [M+Na]$^+$: 393.1402, found: 393.1396.

Note: The relative stereochemistry was assigned in analogy to **6c**.

N-Methoxy-*N*-methyl-2-phenyl-3-(trifluoromethyl)-1-azaspiro[4.5]dec-1-ene-4-carboxamide (6m)

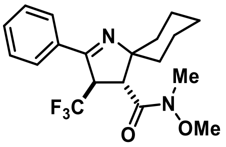

6m

Synthesized following **General Procedure G** using: **1l** (46.00 mg, 0.20 mmol, 1.00 equiv.). The reaction mixture was irradiated for 4 h. Purified by flash column chromatography (SiO_2; 95:5 to 9:1 pentane/EtOAc) to afford **6m** as a 92:8 mixture of diastereomers (36.00 mg, 0.10 mmol, 50%) as a white solid. (d.r. = 92:8).

TLC: R_f = 0.34 (9:1 pentane/EtOAc).

NMR Spectroscopy (*see spectra*):

^{1}H NMR (400 MHz, $CDCl_3$): δ_H 7.77 (dd, J = 7.4, 1.9 Hz, 2.2H), 7.48 – 7.31 (m, 3.3H), 5.00 (d, J = 9.7 Hz, 0.1H), 4.85 (qd, J = 9.9, 5.5 Hz, 1H), 3.81 (s, 0.3H), 3.75 (s, 3H), 3.45 (d, J = 5.5 Hz, 1H), 3.26 (s, 3H), 3.18 (s, 0.3H), 2.04 – 1.85 (m, 3.3H), 1.81 – 1.72 (m, 2.2H), 1.68 – 1.53 (m, 3.3H), 1.44 – 1.31 (m, 2.2H) ppm;

^{13}C NMR (151 MHz, $CDCl_3$): δ_C 172.1, 162.1, 134.2, 130.4, 128.6, 128.3, 125.8 (q, *J* = 279.4 Hz), 79.1, 61.3, 56.7 (q, *J* = 27.8 Hz), 51.8, 40.6, 34.4, 32.6, 26.1, 23.4, 23.1 ppm;

^{19}F NMR (377 MHz, $CDCl_3$): δ_F -63.22, -65.04 ppm.

HRMS (ESI$^+$): m/z calc'd for $C_{19}H_{23}F_3N_2O_2Na$ $[M+Na]^+$: 391.1609, found: 391.1599.

Note: The relative stereochemistry was assigned in analogy to **6c**.

2.7. Product Diversifications

2.7.1. Reduction of Imine to Substituted Pyrrolidine

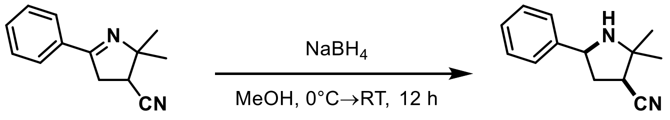

3a **7a**

Following the modified literature procedure, sodium borohydride ($NaBH_4$, 37.00 mg, 1.00 mmol, 5.00 equiv.) was gradually added to the reactant (38.00 mg, 0.20 mmol, 1.00 equiv.) in MeOH (2 mL) at 0°C. The mixture was stirred 12 h at room temperature. After the consumption of the reactant, water (10 mL) was added, and the mixture was extracted with EtOAc (2x 10 mL). The organic layer was separated, and the combined organic extracts were washed with brine (5 mL), and then dried over $MgSO_4$. The solvent was removed under reduced pressure, yielding the pure product **7a** (22.00 mg, 0.11 mmol, 55%) as a white solid.[17] (d.r. >95:5)

2,2-Dimethyl-5-phenylpyrrolidine-3-carbonitrile (7a)

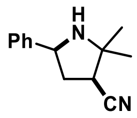

7a

NMR Spectroscopy (*see spectra*):

^{1}H NMR (400 MHz, MeOD): δ_H 7.45 – 7.40 (m, 2H), 7.37 – 7.31 (m, 2H), 7.29 – 7.24 (m, 1H), 4.37 (dd, *J* = 9.4, 7.0 Hz, 1H), 3.12 – 3.03 (m, 1H), 2.83 – 2.70 (m, 1H), 2.20 – 2.07 (m, 1H), 1.44 (s, 3H), 1.36 (s, 3H) ppm;

^{13}C NMR (101 MHz, MeOD): δ_C 143.0, 129.7, 128.6, 127.9, 121.7, 62.7, 61.9, 41.5, 40.5, 28.2, 27.1 ppm.

HRMS (ESI$^+$): m/z calc'd for $C_{13}H_{17}N_2$ $[M+H]^+$: 201.1386, found: 201.1383.

Note: Structure and diastereoselectivity of the product were confirmed by NOE correlations.

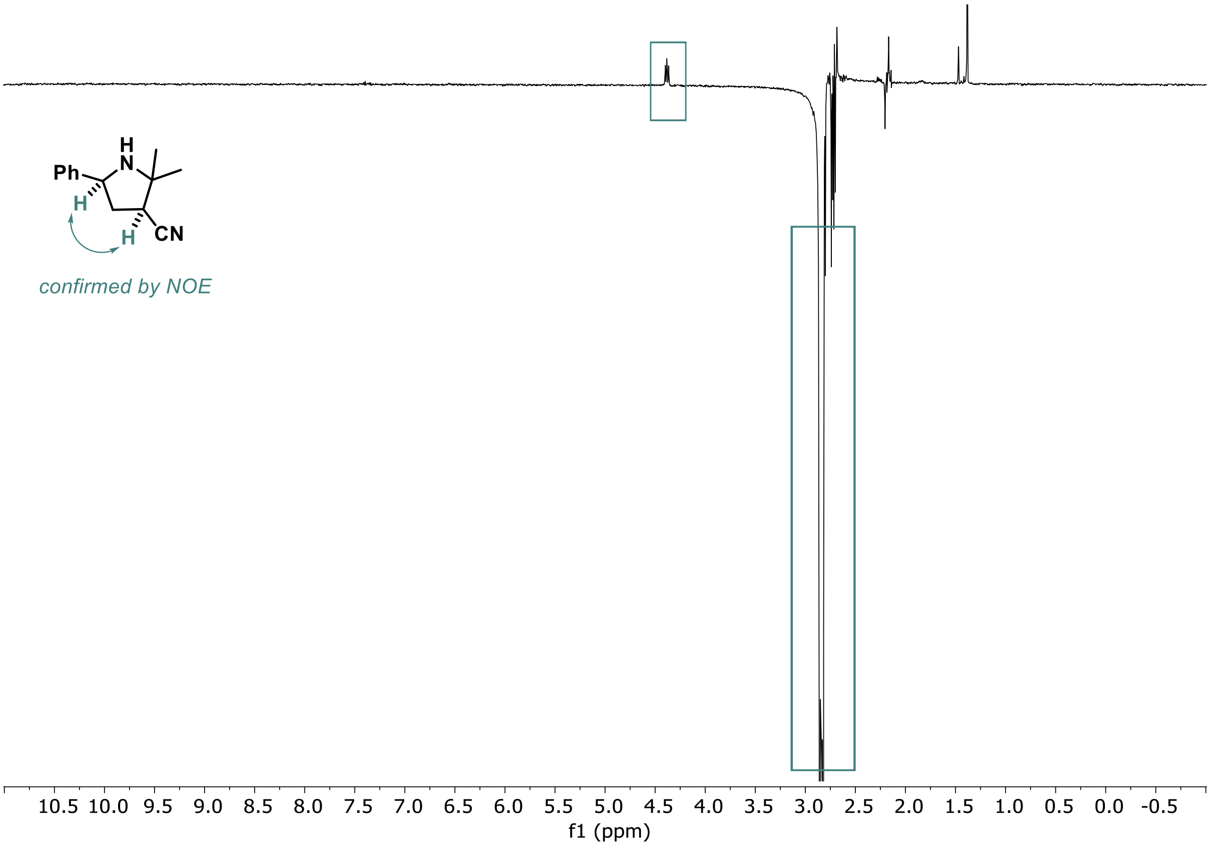

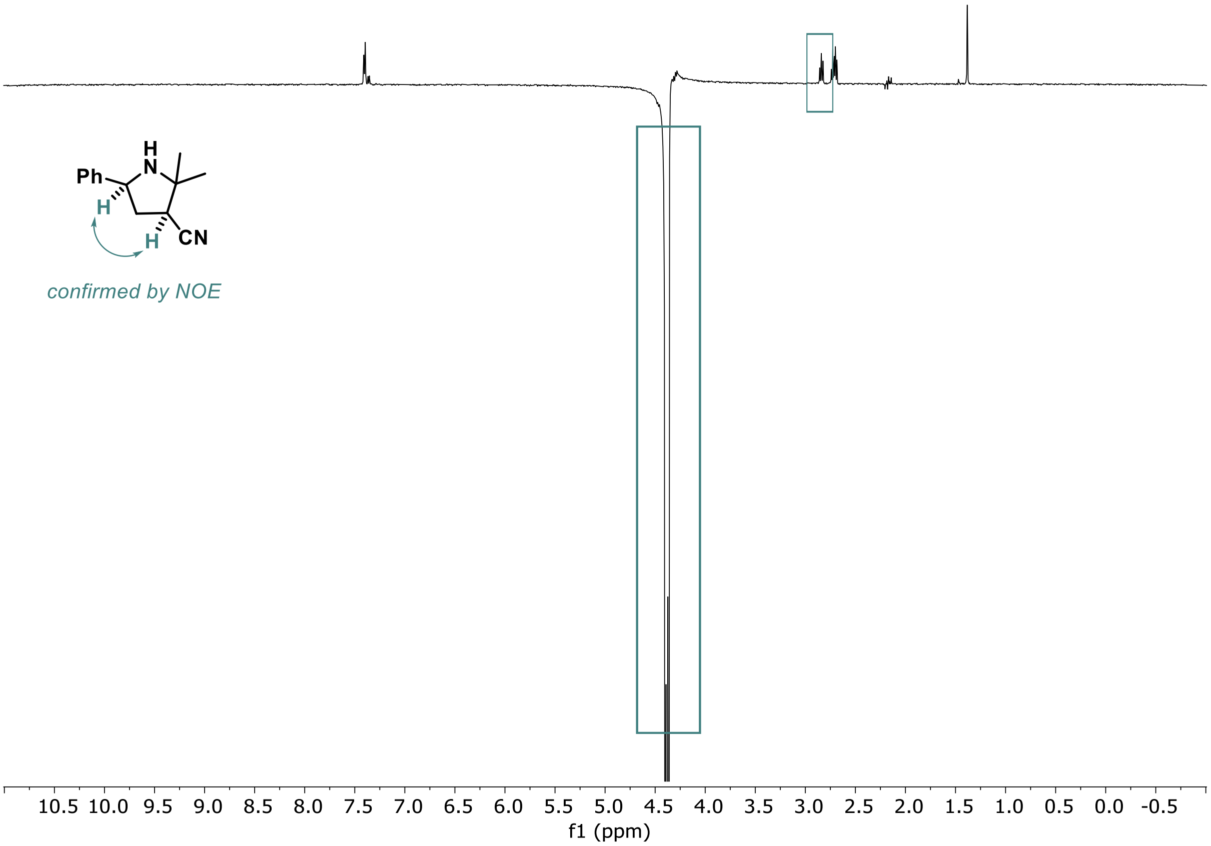

2.7.2. Reduction of Weinreb Amide

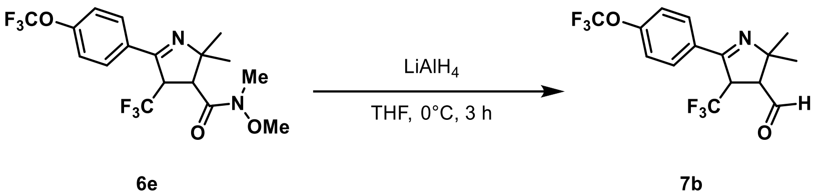

To an oven-dried 10 mL Schlenk tube, equipped with a PTFE-coated stirring bar, was added **6e** (41.20 mg, 0.10 mmol, 1.00 equiv.) and the tube was evacuated and backfilled with argon three times. Then, THF (2 mL, 0.05 M) was added and the solution was cooled to 0°C. Then, $LiAlH_4$ (20.00 mg, 0.50 mmol, 5.00 equiv.) was added at 0°C portion wise and it was stirred at the same temperature for 3 h. Then, the reaction was quenched with NH_4Cl aq. (2 mL), diluted with EtOAc (5 mL) and the phases were separated. The aqueous phase was extracted with EtOAc (3x 3 mL) and the combined organic phases dried over $MgSO_4$, filtered and concentrated under reduced pressure. The crude mixture was purified by flash column chromatography (SiO_2, 80:20 pentane/EtOAc) to yield the product **7b** as a yellow oil (31.10 mg, 0.09 mmol, 88%).

2,2-Dimethyl-5-(4-(trifluoromethoxy)phenyl)-4-(trifluoromethyl)-3,4-dihydro-2*H*-pyrrole-3-carbaldehyde (7b)

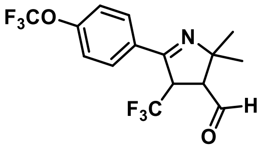

7b

NMR Spectroscopy (*see spectra*)

^{1}H NMR (599 MHz, $CDCl_3$)**:** δ_H 9.84 (s, 1H), 7.80 – 7.75 (m, 2H), 7.29 – 7.23 (m, 2H), 4.88 (qd, J = 9.3, 6.1 Hz, 1H), 3.33 (dd, J = 6.2, 1.1 Hz, 1H), 1.67 (s, 3H), 1.33 (s, 3H) ppm;

^{13}C NMR (151 MHz, $CDCl_3$): δ_C 196.8, 162.1, 151.2, 131.8, 130.1, 125.1 (q, *J* = 279.1 Hz), 120.7, 120.5 (q, *J* = 258.1 Hz), 74.8, 60.5, 60.5, 52.3 (q, *J* = 28.6 Hz), 31.1, 26.0 ppm.

^{19}F NMR (377 MHz, $CDCl_3$) δ_F -57.7, -65.2.

HRMS (ESI$^+$): m/z calc'd for $C_{15}H_{13}NO_2F_6$ [M+Na]$^+$: 354.0923, found: 354.0921.

2.7.3. Hydrolysis of Cyclic Imine to 1,4-Amino Ketones

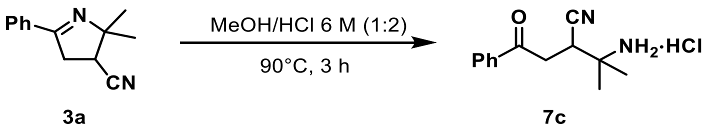

Following a modified literature procedure, a 50 mL tube was filled with the reactant (0.06 g, 0.30 mmol, 1.00

equiv.) dissolved in 4 mL of methanol. To this solution, 7 mL of 6 M HCl was added, and the mixture was stirred at 90°C. Completion of reaction was monitered by TLC till 3 h. After this period, the reaction was allowed to cool to room temperature. The reaction mixture was diluted with water. After multiple round of washing with ethyl acetate to remove the impurities, aqueous phase was collected. Evaporation of water using the freeze dryer, yielded the desired product **7c** as white solid (52.00 mg, 0.20 mmol, 68%). There are some minor impurities in the aliphatic region that remain, even after multiple rounds of washing.[18]

3-Cyano-2-methyl-5-oxo-5-phenylpentan-2-aminium chloride (7c)

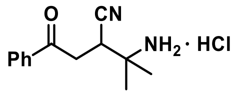

7c

NMR Spectroscopy (*see spectra*):

^{1}H NMR (400 MHz, MeOD**):** δ_H 8.22 – 8.14 (m, 2H), 7.90 (t, *J* = 7.5 Hz, 1H), 7.72 (t, *J* = 7.7 Hz, 2H), 4.31 (dd, *J* = 19.3, 9.3 Hz, 1H), 4.16 (dd, *J* = 19.3, 7.9 Hz, 1H), 3.98 – 3.91 (m, 1H), 1.77 (s, 6H) ppm;

^{13}C NMR (101 MHz, MeOD): δ_C 182.8, 138.7, 132.4, 131.2, 126.7, 118.2, 73.2 (d, *J* = 2.4 Hz), 39.8, 27.1, 24.7 (dd, *J* = 3.5, 1.3 Hz) ppm.

HRMS (ESI$^+$): m/z calc'd for $C_{13}H_{17}N_2O$ [M-Cl]$^+$: 217.1335, found: 217.1335.
Note: The NH_2 proton peak cannot be seen possibly due to proton exchange with the solvent.

2.7.4. Methylation of Weinreb Amide

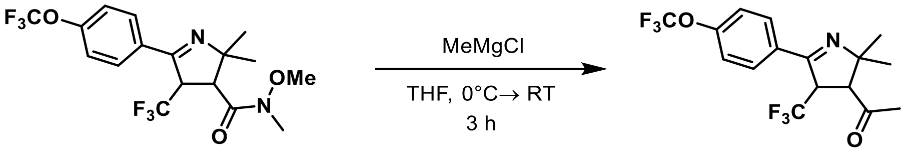

6f **7d**

Following the modified literature procedure, under an inert atmosphere, a round-bottom flask is charged with *N*-methoxy-*N*-methyl-2-phenylacetamide (0.08 g, 0.20 mmol, 1.00 equiv.) and dry THF (2 mL, 0.10 M). The mixture is cooled to 0°C, and a solution of methylmagnesium chloride (740 µL, 1.00 mmol, 5.00 equiv., 3 M in THF) is added dropwise. The reaction mixture is then allowed to warm to room temperature and stirred for 3 hours. After completion, the mixture is extracted with CH_2Cl_2 (3x 20mL), dried over magnesium sulfate, filtered, and concentrated under reduced pressure. The crude residue is purified by flash column chromatography (SiO_2; 100:0 to 90:10 pentane/EtOAc) to yield the ketone **7d** as a colorless oil (56.00 mg, 0.15 mmol, 77%).[19]

1-(2,2-Dimethyl-5-(4-(trifluoromethoxy)phenyl)-4-(trifluoromethyl)-3,4-dihydro-2*H*-pyrrol-3-yl)ethan-1-one (7d)

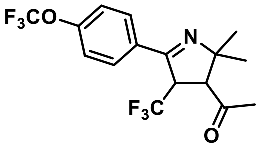

7d

NMR Spectroscopy (*see spectra*):

^{1}H NMR (500 MHz, $CDCl_3$): δ_H 7.75 – 7.69 (m, 2H), 7.24 (d, *J* = 8.0 Hz, 2H), 4.86 (qd, *J* = 9.2, 7.4 Hz, 1H), 3.44 (d, *J* = 7.5 Hz, 1H), 2.34 (s, 3H), 1.69 (s, 3H), 1.17 (s, 3H) ppm;

^{13}C NMR (126 MHz, $CDCl_3$): δ_C 204.1, 162.4, 151.0, 151.0, 132.0, 130.0, 125.3 (d, *J* = 279.7 Hz), 120.6, 120.6 120.5 (d, *J* = 259.6 Hz), 74.1, 61.8, 55.9 (q, *J* = 28.0 Hz), 31.3, 31.1, 25.0 ppm;

^{19}F NMR (470 MHz, $CDCl_3$): δ_F -57.74, -65.54 ppm.

HRMS (ESI$^+$): m/z calc'd for $C_{16}H_{16}NO_2F_6$ $[M+H]^+$: 368.1080, found: 368.1076.

2.7.5. Epoxidation of Imine to Access Fused Oxaziridine

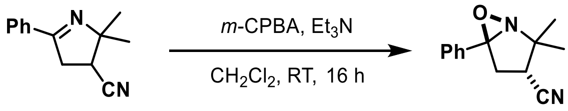

3a **7e**

Following the literature procedure, to a stirred solution of reactant (60 mg, 0.30 mmol, 1.00 equiv.) in dry CH_2Cl_2 (2 mL), *m*-CPBA (0.07 g, 0.39 mmol, 1.30 equiv.) was discharged at room temperature and the reaction was stirred overnight. Then, Et_3N (62 μL, 0.45 mmol, 1.50 equiv.) was added to the reaction mixture. Completion of the reaction was analysed by TLC. The resulting mixture was purified by flash chromatography (SiO_2; 100:0 to 95:5 pentane/EtOAc) to obtain the intended product **7e** (28.00 mg, 0.20 mmol, 65%) as a white solid.[20] (d.r. >95:5)

2,2-Dimethyl-5-phenyl-6-oxa-1-azabicyclo[3.1.0]hexane-3-carbonitrile (7e)

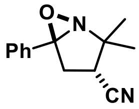

7e

NMR Spectroscopy (*see spectra*):

^{1}H NMR (400 MHz, $CDCl_3$): δ_H 7.48 – 7.36 (m, 5H), 3.10 (dd, *J* = 12.3, 5.6 Hz, 1H), 2.86 – 2.71 (m, 2H), 1.51 (s, 3H), 1.35 (s, 3H) ppm;

^{13}C NMR (101 MHz, $CDCl_3$): δ_C 133.1, 129.9, 128.5, 126.7, 118.5, 85.7, 68.2, 33.3, 32.6, 24.0, 20.8 ppm.

HRMS (ESI^+): m/z calc'd for $C_{13}H_{14}N_2O_1Na$ $[M+Na]^+$: 237.0998, found: 237.0997.
X-ray (*see data*)

2.8. Condition-Based Sensitivity Screen

In a 50 mL Schlenk tube under argon atmosphere, isoxazolone **1a** (230.00 mg, 1.20 mmol, 1.00 equiv.), acrylonitrile **2a** (640.00 mg, 12.00 mmol, 10.00 equiv.) and $[Ir\{dF(CF_3)ppy\}_2(dtbbpy)]PF_6$ (**Ir-F**) (13.00 mg, 0.01 mmol, 1 mol%) was dissolved in $PhCF_3$ (10.8 mL). 10 10 mL Schlenk tubes were charged with a magnetic stir bar and set under an argon atmosphere. 0.9 mL of the stock solution and 0.1 mL of $PhCF_3$ were added and the reaction mixtures were irradiated at room temperature with 450 nm blue LEDs for 3 h. Deviations from these conditions are shown. Another reaction was set up using isoxazolone **1a** (760.00 mg, 4.00 mmol, 1.0 equiv.), acrylonitrile **2a** (2.7 mL, 40.00 mmol, 10.00 equiv.) and **Ir-F** (45.00 mg, 0.04 mmol, 1 mol%) was dissolved in $PhCF_3$ (40 mL). The reaction mixtures were analysed by quantitative ^1H-NMR spectroscopy using dibromo methane (7 µL, 0.10 mmol, 1.00 equiv.) as an internal standard.[21]

Table S1: Results of the condition-based sensitivity screen.

Entry	Modification	Setup Deviation	Yield	Yield Deviation
**1**	control	none	64%	-
**2**	high concentration	without 0.1 mL $PhCF_3$	48%	-16%
**3**	low concentration	+0.1 mL $PhCF_3$	60%	-4%
**4**	high H_2O	+10 µL H_2O	54%	-10%
**5**	high O_2	+10 mL air	58%	-6%
**6**	low O_2	freeze pump thaw	56%	-8%
**7**	high intensity	LED distance of 3 cm	66%	+2%
**8**	low intensity	LED distance of 30 cm	18%	-46%
**9**	high temperature	fan off	40%	-24%
**10**	low temperature	water cooling	38%	-26%
**11**	big scale	4.0 mmol scale	51%	-13%

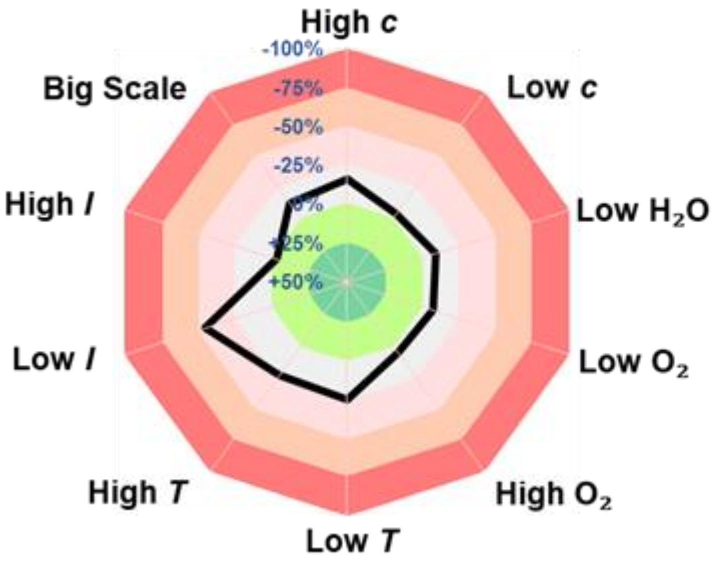

Figure S3: Radar graph representation of sensitivity screen.

2.9. Additive-Based Robustness Screen

The functional group tolerance and robustness screen was examined as reported by Glorius and coworkers[22], using conditions modified from **General Procedure F**. Different additives containing various functional groups and heterocycles were screened in additive-based robustness screen. Therefore, based on the product and additive recovery, the compatibility of functional groups and stability of additives in the showcased methodology is evaluated.

Stock solution preparation:

To an oven-dried Schlenk tube equipped with a Teflon-coated magnetic stir bar was discharged [Ir{dF(CF_3)ppy}$_2$(dtbbpy)]PF_6 (**Ir-F**) (20.00 mg, 0.10 mmol, 1.0 mol%). The Schlenk tube was evacuated and backfilled with argon three times before 4,4-dimethyl-3-phenylisoxazol-5(4*H*)-one **1a** (378.00 mg, 2.00 mmol, 1.00 equiv.), acrylonitrile **2a** (1.30 mL, 20.0 mmol, 1.00 equiv.) and $PhCF_3$ (20 mL, 0.10 M) were added.

Reaction preparation:

Fifteen oven-dried Schlenk tubes equipped with a Teflon-coated magnetic stir bar were evacuated and backfilled with argon three times before the addition of freshly prepared stock solution (1 mL) and the respective additive (0.10 mmol, 1.00 equiv.) to each reaction schlenk tube. A control reaction without an additive was also prepared simultaneously. The reaction mixtures were stirred under irradiation with blue LEDs (18 W, λ_{max} = 450 nm) for 3 h. After this time, mesitylene (14 µL, 0.10 mmol, 1.00 equiv.) was added as an internal standard for analysis of the product yield and the remaining additive by GC-FID. The results are shown in **Table S2**.

The color coding is defined as such:

Recovered additive: green (>66%), yellow (33-66%), red (<33%).

Product yield (with respect to the control reaction): green (>42%), yellow (21-42%), red (<21%).

Table S2: Results of the additive-based robustness screen.

Entry	Additive	Yield Additive	Yield Product	
1	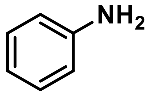	91	0	Aniline
2	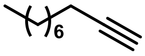	78	34	1-Decyne
3	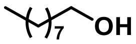	85	42	1-Nonanol
4	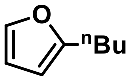	62	5	2-Butylfuran
5	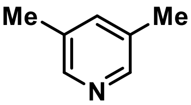	9	10	3,5-Dimethylpyridine
6	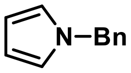	89	8	Benzylpyrrole
7	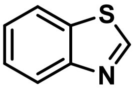	92	9	Benzothiazol
8	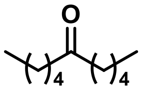	98	47	Alkylketone
9	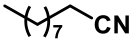	88	27	Decannitril
10	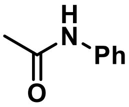	92	36	Acetanilide
11	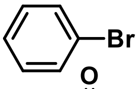	98	40	Bromobenzene
12	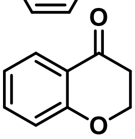	77	43	Chromanone
13	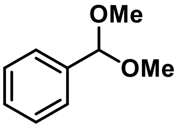	90	50	Dimethyl acetal
14	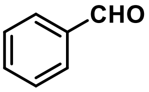	10	1	Benzaldehyde
15	none	none	50	Control

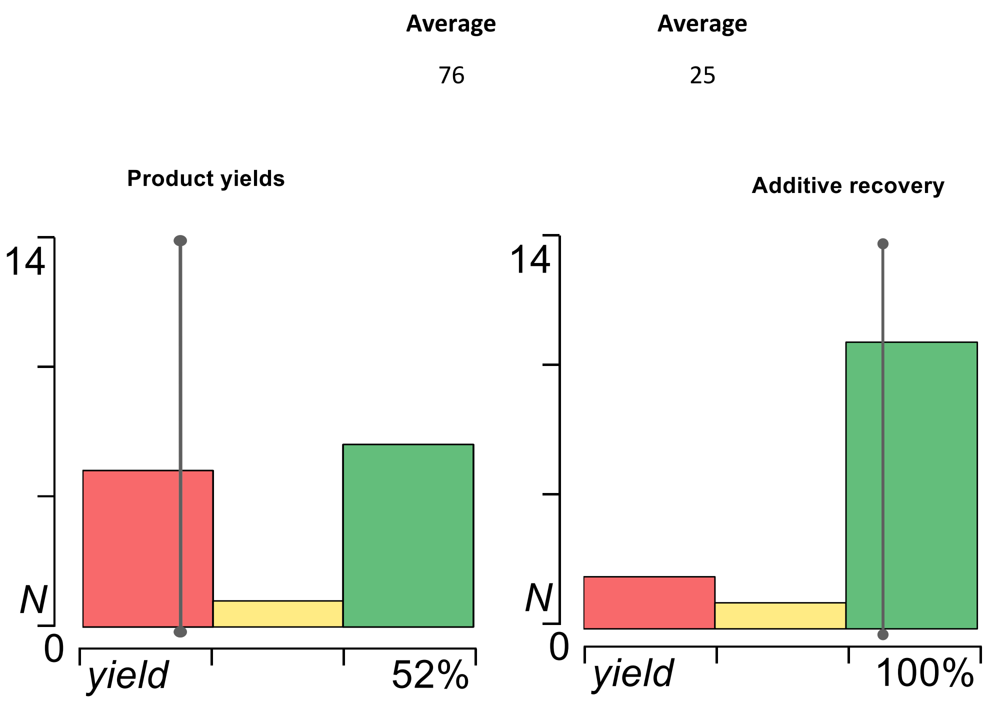

Figure S4: Bar graph representation of robustness screen. Left: Impact of additive normed on product yield. Right: Recovery of additive. Grey Line: average

2.10. Time-Course Study

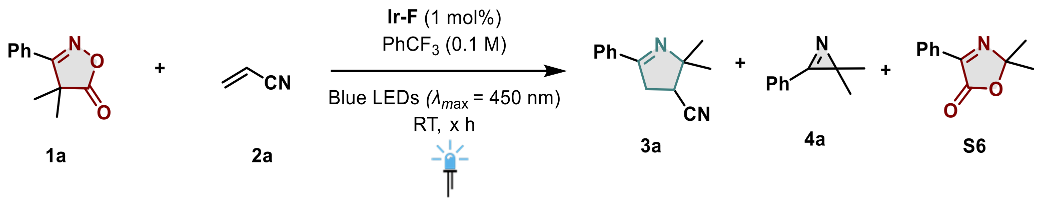

In oven-dried Schlenk tubes and under argon atmosphere, a 2 mM stock solution of [Ir{dF(CF_3)ppy}$_2$(dtbbpy)]PF_6 **(Ir-F)** (11.20 mg) in $PhCF_3$ (5 mL) and a 0.20 M stock solution of **1a** (190.00 mg) in $PhCF_3$ (5.0 mL) were prepared. 7 oven-dried Schlenk tubes were charged with a magnetic stir bar and set under argon atmosphere. To each Schlenk tube, 0.5 mL of each stock solution and acetonitrile (66 µL, 1.00 mmol, 10.0 equiv.) were added resulting in a reaction mixture containing 0.10 mmol of **1a**, 1 µmol of **Ir-F** and 1.00 mmol of **2** in $PhCF_3$ (1 mL, 0.10 M). The reaction mixtures were irradiated with blue LEDs (18 W, λ_{max} = 450 nm) for the indicated time. Then, the solvent was removed under reduced pressure and the crude product was analyzed by ^{1}H-NMR (vs. 7 µL of CH_2Br_2 as the internal standard).

Table S3: Data from monitoring the reaction using ^{1}H-NMR (vs. CH_2Br_2).

Time / min	[1] / %	[3] / %	[4] / %	[6] / %
5	70	5	19	1
15	51	14	27	3
30	34	23	27	5
60	23	36	18	7
105	14	44	14	8
150	7	51	8,0	11
180	4	56	7	11

2.11. Synthesis of 2,2-Dimethyl-4-phenyloxazol-5(2*H*)-one (S6)

Compound **S6** was identified as major side product of the standard reaction, therefore justifying the low yields obtained. It was also the main product formed in the absence of alkene.

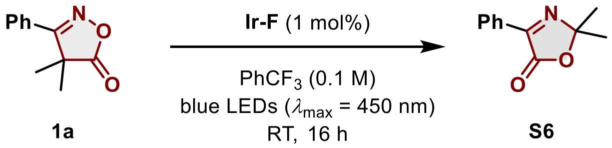

To an oven-dried 10 mL Schlenk tube equipped with a Teflon-coated magnetic stir bar was added [Ir{dF(CF_3)ppy}$_2$(dtbbpy)]PF_6 (**Ir-F**) (1.10 mg, 2.0 µmol, 1 mol%) and 4,4-dimethyl-3-phenylisoxazol-5(4*H*)-one **1a** (37.80 mg, 0.20 mmol, 1.00 equiv.). The Schlenk tube was evacuated and backfilled with argon three times before $PhCF_3$ (2.00 mL) was added under a positive argon pressure. The reaction mixture was stirred under irradiation with blue LEDs (18 W, λ_{max} = 450 nm) for 16 h. After this time, the solvent was removed under reduced pressure and the crude product was purified by preparative thin layer chromatography (SiO_2, 90:10 pentane/EtOAc) on silica gel to afford **S6** (11.00 mg, 0.06 mmol, 58%) as a white solid.

NMR Spectroscopy (*see spectra*):

^{1}H NMR (400 MHz, $CDCl_3$): δ_H 8.34 – 8.27 (m, 2H), 7.54 – 7.37 (m, 3H), 1.62 (s, 6H). ppm;

^{13}C NMR (101 MHz, $CDCl_3$): δ_C 164.4, 155.5, 132.39, 128.8, 128.6, 104.0, 26.2. ppm.

HRMS (ESI$^+$): m/z calc'd for $C_{11}H_{11}NO_2$ [M+Na]$^+$: 211.0941, found: 211.0939.

X-ray (*see data*)

3. MECHANISTIC STUDIES

3.1. UV/Vis Absorption Spectroscopy

UV/Vis absorption spectra were measured with a Jasco V-730 spectrophotometer, equipped with a temperature control unit at 25 °C. Recordings of the spectra were carried out using Starna® fluorescence quartz cuvettes (type: 29-F, chamber volume = 1.40 mL, H x W x D = 48 mm x 12.5 mm x 12.5 mm, path length = 10 mm). For the measurements, the following parameters were used: response time = 0.06 sec, data interval = 0.5 nm, scan speed = 1000 nm/min. All measurements were performed in $PhCF_3$, whereas the concentration of azirine **4a**, alkene **2a**, and isoxazolone **1a** were set to the standard concentration of c(**4**) = c(**2a**) = c(**1a**) = 0.1 M, respectively. For the photocatalyst [Ir{dF(CF_3)ppy}$_2$(dtbbpy)]PF_6 (**Ir-F)**, a concentration of c(**Ir-F**) = 10^{-4} M was used, corresponding to the concentration in the reaction mixture.

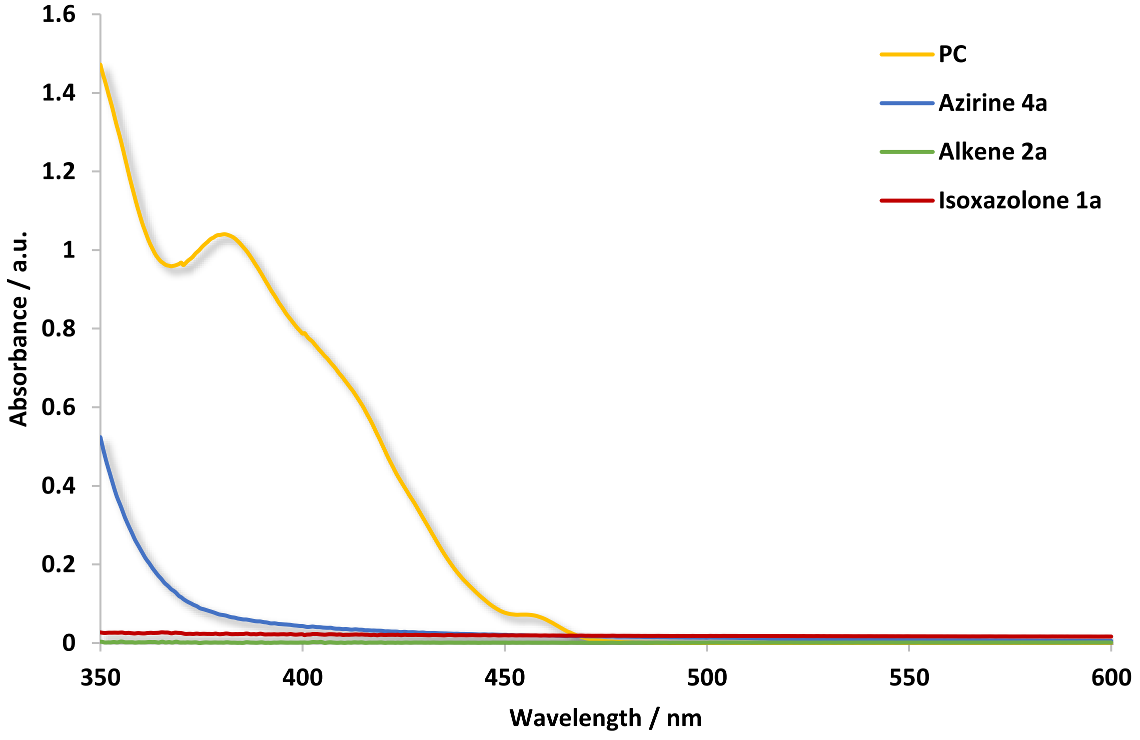

Figure S5: UV/Vis absorption spectroscopy.

3.2. Stern-Volmer Analysis

Stern–Volmer luminescence quenching studies were carried out to identify the relative quenching of the excited state photocatalyst of the reaction partners. In this context, the luminescence of the employed photocatalyst [Ir(dF(CF_3)ppy)$_2$dtbbpy][PF_6] (**Ir-F**) is measured in presence of varying quencher concentrations.

The quenching studies were carried out on a JASCO FP-8300 spectrofluorometer using Starna® fluorescence quartz cuvettes (type: 29-F, chamber volume = 1.40 mL, H x W x D = 48 mm x 12.5 mm x 12.5 mm, path length = 10 mm). The following parameters were set: data interval = 0.5 nm, scan-speed = 500 nm/min, excitation

wavelength λ_{ex} = 400 nm, measured luminescence λ = 475 nm. All samples were prepared in an argon-filled glovebox with dry $PhCF_3$. The studies were performed using a stock-solution of **Ir-F** with a concentration of *c*(**Ir-F**) = 25 μM, whereas the varying concentrations of the quencher were achieved by dilution of respective stock-solutions. The samples were prepared in cuvettes, which were then sealed with PTFE stoppers and removed from the glovebox and directly measured.

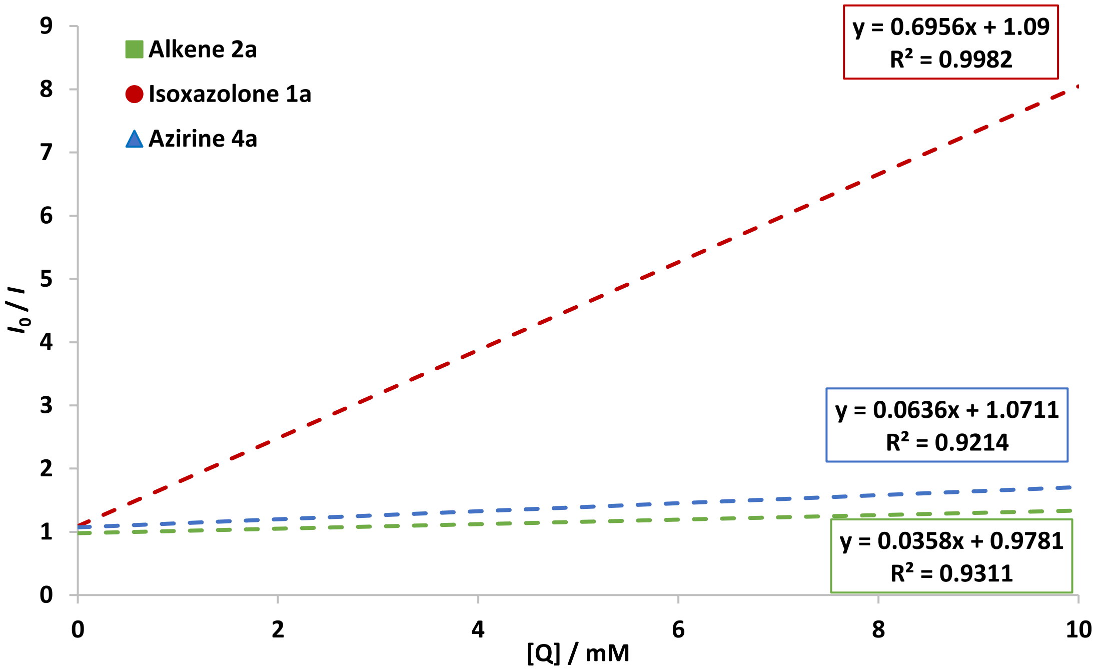

Figure S6: Stern-Volmer analysis.

3.3. Cyclic Voltammetry Studies

Cyclic voltammograms (CVs) were measured at room temperature with a Metrohm Dropsens μStat-i 400s potentiostat. A 2 mm glassy carbon disc electrode was used as the working electrode. A Ag/AgCl (2 M LiCl in ethanol) electrode was used as the reference electrode. A platinum sheet electrode was used as the counter electrode. All the electrodes were supplied by Metrohm. The electrolyte solution contained 0.05 M tetrabutylammonium hexafluorophosphate ($TBAPF_6$) and 10 mM of the respective substrate in MeCN. The solution was purged with nitrogen gas before the measurement to avoid the interference with atmospheric oxygen. The scan rate was set at 0.1 V/s (E_{step} = 0.002 V) and 10 scans were taken in a potential window of 0.0 V to +2.5 V.

The redox potential for the only redox active species within the potential window (**4a**) was converted to the saturated calomel electrode (SCE) scale by subtracting 45 mV (0.045 V).

E^{ox}(**4a**) = 1.28 V (vs Ag/AgCl) −0.045 V = 1.24 V (vs SCE)

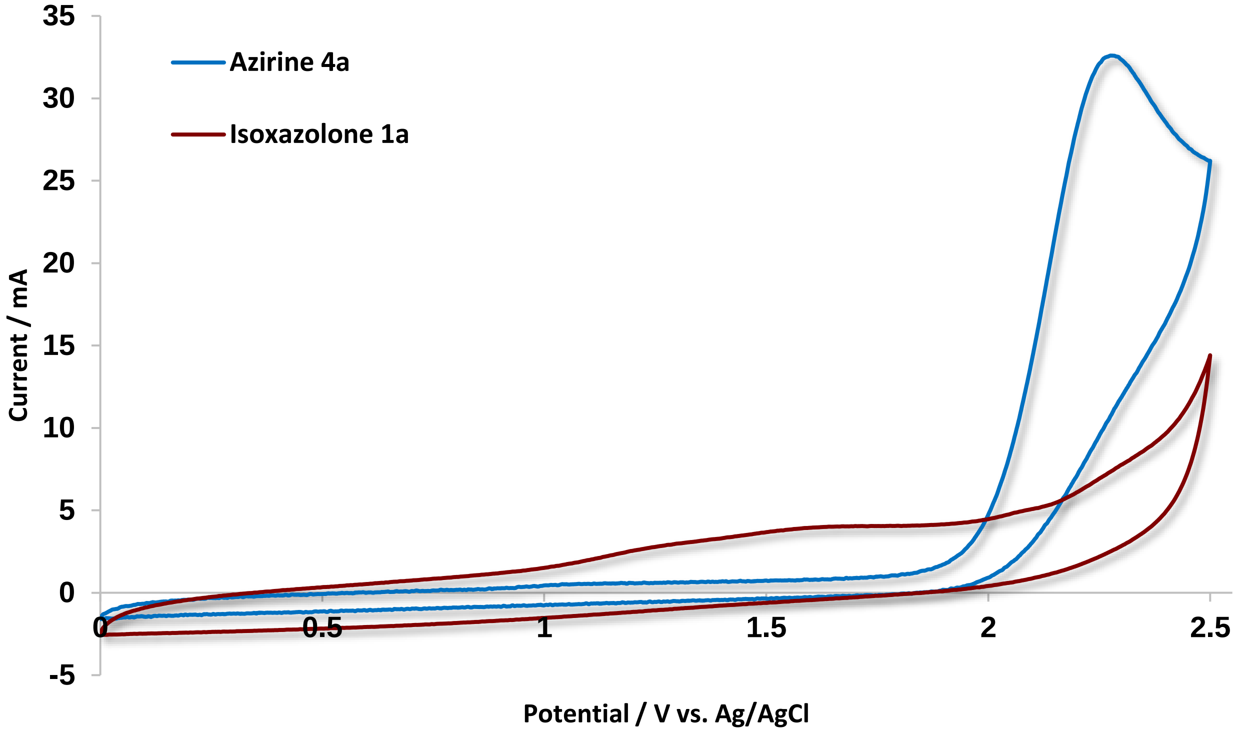

Figure S7: Cyclic voltammetry of **1a**, and **4a** in 0.1 M $TBAPF_6$ (MeCN), using a 2 mm glassy carbon disk working electrode, Pt sheet counter electrode and an Ag/AgCl (2 M LiCl in ethanol) reference electrode. Set at 0.1 V/s scan rate.

.

3.4. Radical Trapping Studies

3.4.1. TEMPO Trapping Study

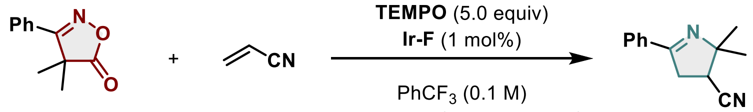

To an oven-dried 10 mL Schlenk tube equipped with a Teflon-coated magnetic stir bar was added [Ir{dF(CF_3)ppy}$_2$(dtbbpy)]PF_6 (**Ir-F**) (1.10 mg, 1.0 µmol, 1 mol%), and 4,4-dimethyl-3-phenylisoxazol-5(4*H*)-one **1a** (18.90 mg, 0.10 mmol, 1.00 equiv.). The Schlenk tube was evacuated and backfilled with argon three times before acrylonitrile (66 µL, 1.00 mmol, 10.00 equiv.), (2,2,6,6-tetramethylpiperidin-1-yl)oxyl (**TEMPO**) (78.20 mg, 0.50 mmol, 5.00 equiv.) and $PhCF_3$ (1.0 mL) were added under a positive argon pressure. The reaction mixture was stirred under irradiation with blue LEDs (18 W, λ_{max} = 450 nm) for 3 h. After this time, the solvent was removed under reduced pressure.

Analysis of the crude reaction mixture revealed that the product formation was completely suppressed, supported by GC-MS and HRMS. Furthermore, no TEMPO-trapping adduct of the proposed reaction-

intermediates could be detected by HRMS.

3.4.2. BHT Trapping Study

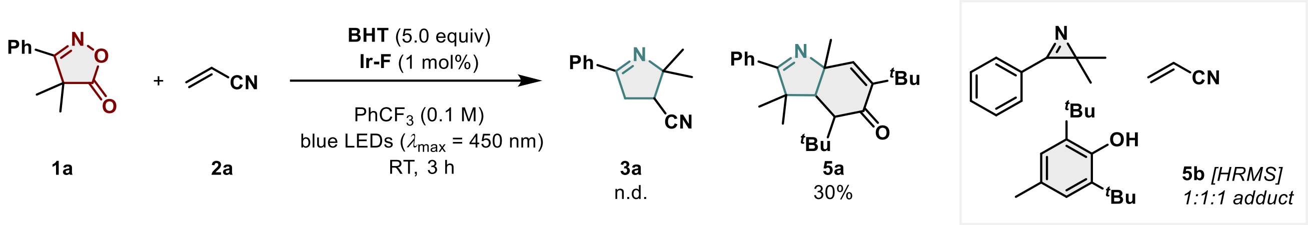

To an oven-dried 10 mL Schlenk tube equipped with a Teflon-coated magnetic stir bar was added [Ir{dF(CF_3)ppy}$_2$(dtbbpy)]PF_6 (**Ir-F**) (1.10 mg, 1.0 µmol, 1.0 mol%), and 4,4-dimethyl-3-phenylisoxazol-5(4*H*)-one **1a** (18.90 mg, 0.10 mmol, 1.00 equiv.). The Schlenk tube was evacuated and backfilled with argon three times before acrylonitrile (66 µL, 1.00 mmol, 10.00 equiv.), 2,6-di-*tert*-butyl-4-methylphenol (**BHT**) (110.00 mg, 0.50 mmol, 5.0 equiv.) and $PhCF_3$ (1.0 mL) were added under a positive argon pressure. The reaction mixture was stirred under irradiation with blue LEDs (18 W, λ_{max} = 450 nm) for 3 h. After this time, the solvent was removed under reduced pressure.

According to GC-MS analysis, formation of **3a** was completely suppressed. HRMS analysis of the crude reaction mixture indicated a trapping adduct consisting of alkene, azirine and BHT (**5b**): $[M+H]^+$ 419.3057, found 419.3059. Furthermore, a trapping adduct of the azirine and BHT was identified via HRMS: $[M+H]^+$ 366.2791, found 366.2791. Purification of the crude reaction mixture by preparative thin layer chromatography (SiO_2, two times: 97:3 pentane/EtOAc) yielded the respective trapping adduct **5a** as a white solid and single diastereomer (10.80 mg, 0.03 mmol, 30%).

NMR Spectroscopy (*see spectra*)

^{1}H NMR (500 MHz, $CDCl_3$): δ_H 7.50 – 7.45 (m, 2H), 7.41 – 7.30 (m, 3H), 6.14 (s, 1H), 4.11 (d, *J* = 3.6 Hz, 1H), 2.60 (d, *J* = 3.7 Hz, 1H), 1.27 (s, 3H), 1.23 (s, 3H), 1.20 (s, 9H), 1.08 (s, 9H), 0.98 (s, 3H) ppm;

^{13}C NMR (126 MHz, $CDCl_3$): δ_C 200.7, 179.4, 148.4, 142.1, 135.5, 129.4, 128.3, 127.9, 74.3, 62.3, 57.9, 46.7, 35.3, 34.4, 30.0, 29.3, 24.3, 23.5, 21.8 ppm.

HRMS (ESI$^+$): m/z calc'd for $C_{25}H_{36}NO$ $[M+H]^+$: 366.2791, found: 366.2791.

Note: Structure and diastereoselectivity of the product were confirmed by NOE correlations.

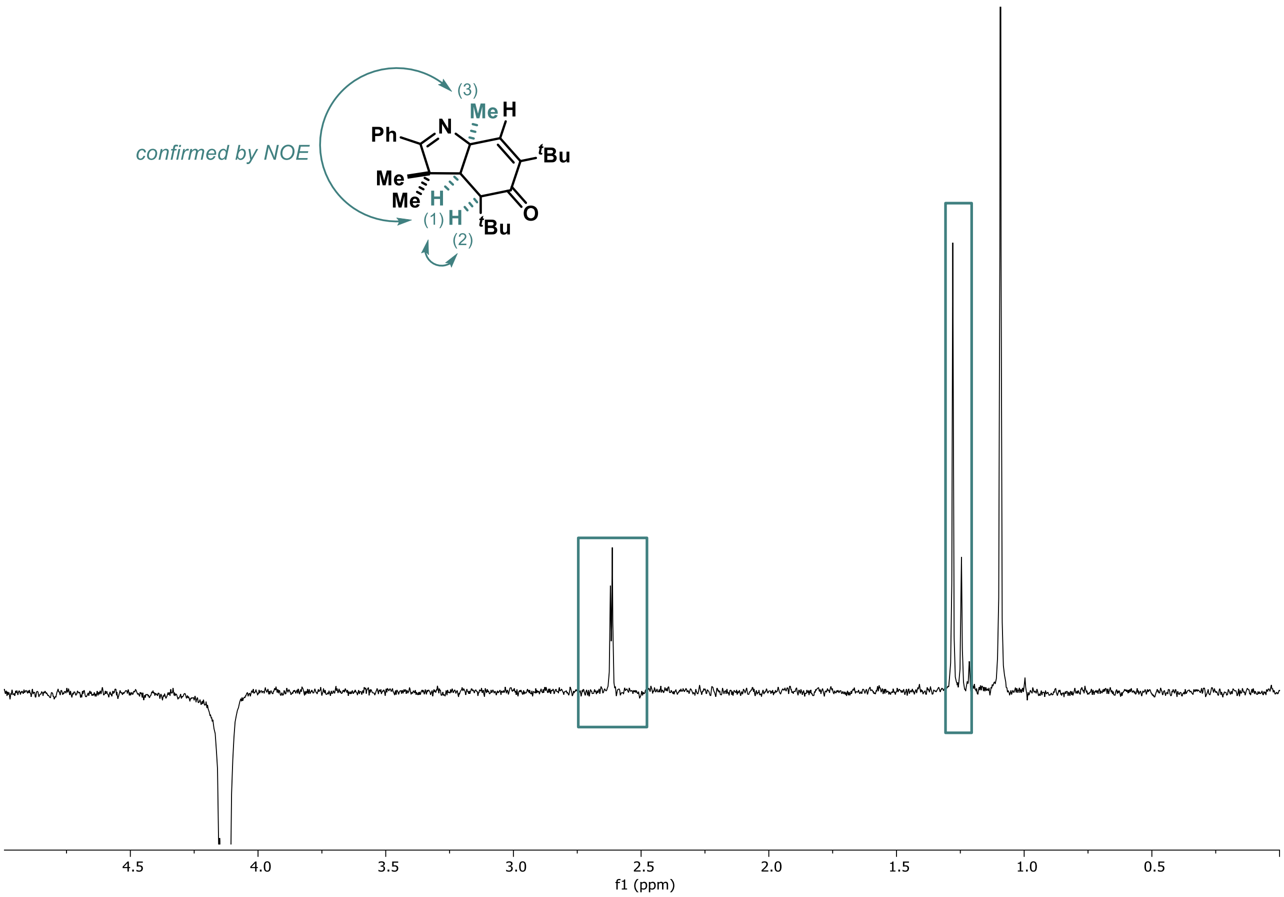
confirmed by NOE
(3)
Me H
Ph
N
tBu
Me
Me H
(1) H
tBu
O
(2)
f1 (ppm)

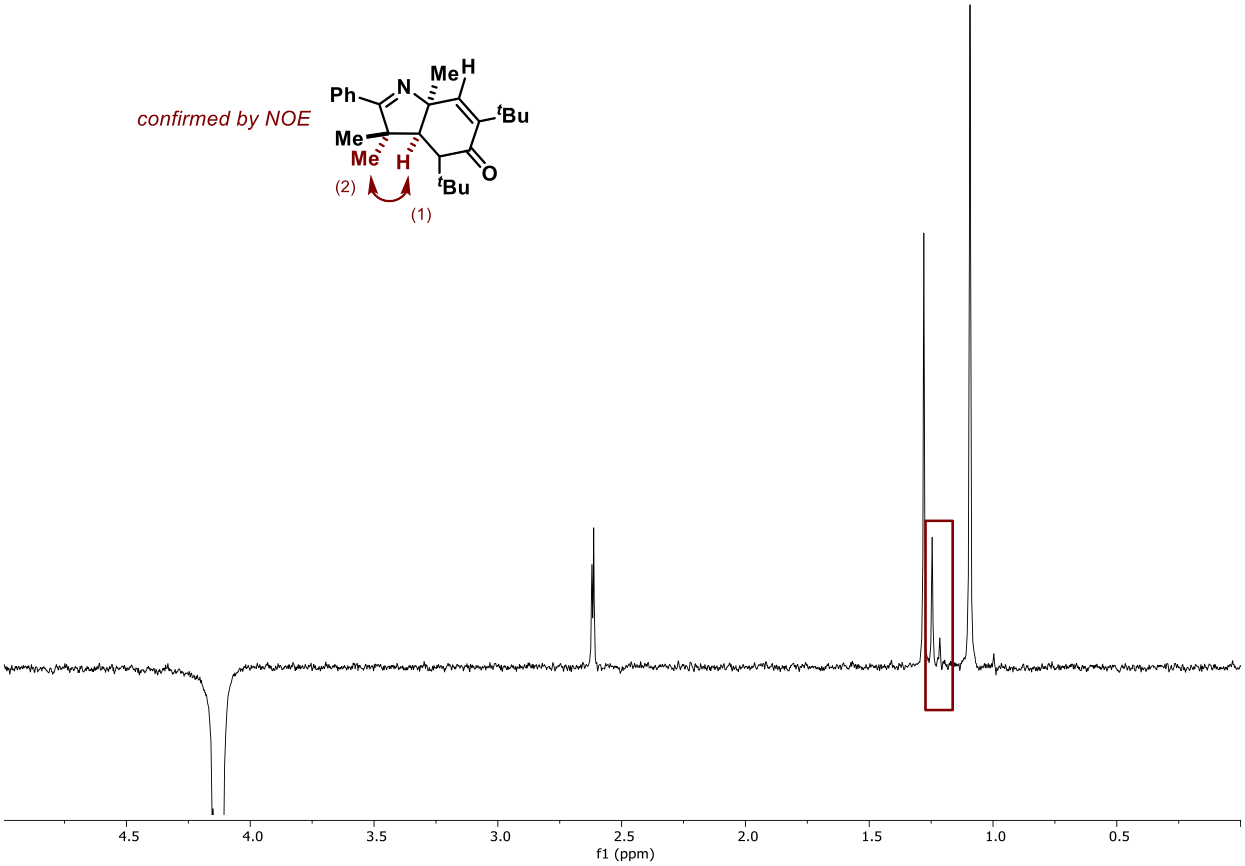
confirmed by NOE
Me H
Ph
N
tBu
Me
Me H
tBu
O
(2)
(1)
f1 (ppm)

The isolated trapping product supports the proposed mechanism with the ring-closed azirine as the intermediate, yielded after CO_2 extrusion of isoxazolone starting material and ring-closure of *C*-, *N*-diradical, which was herein trapped by BHT.

3.5. Activation of Azirine

Table S4: Variation of photocatalyst for azirine activation.

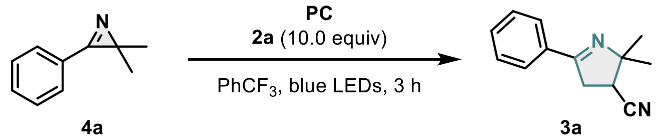

4a (0.1 mmol, 1.0 equiv) **3a**

Entry	PC	mol %	Wavelength	$E^{1/2}$(PC*/PC^{-})	E_T	Yield of 3a
1	TXT	10	405 nm	1.18 V	63.4 kcal/mol	60%
2	Ir-F	1	450 nm	1.21 V	61.8 kcal/mol	80%
3	[Mes-Acr-Me][ClO_4]	5	450 nm	2.18 V	44.7 kcal/mol	3%
4[a]	-	-	-	-	-	n.d.

[a]Reaction conducted at 60°C in the dark. Yield calculated via NMR analysis using CH_2Br_2 as internal standard.

To further understand the mode of reactivity, the proposed 2*H*-azirine intermediate was investigated under the standard reaction conditions employing different photocatalysts. With similar oxidation potential but slightly decreased oxidation potential, thioxanthone photocatalyst (**TXT**, **Table S4**, Entry 1) performs reasonably well in comparison to **Ir-F** (Entry 2). Notably, the ^{1}H-NMR yield of the reaction is slightly increased when using the 2*H*-azirine instead of **1a** with **Ir-F** as photocatalyst, likely due to lack of CO_2 for the formation of the azirine CO_2 side product observed in the standard reaction (***see 2.10.***). Based on recent literature reports for the photoredox-enabled formal [3+2] cycloaddition processes for 2*H*-azirines[23,24] **[Mes-Acr-Me][ClO_4]** was investigated as photocatalyst. However, reactivity was observed to be nearly shut down. Notably, the redox potential is strongly increased compared to the other photocatalysts, but triplet energy is significantly lower and underneath the calculated triplet energy of **4a** (Entry 3, also compare ***chapter 5.***). To rule out a thermal process, the reactivity was also investigated without photocatalyst at 60 °C in the dark (Entry 4). However, no product was formed and only starting material observed by GC-MS analysis of the crude reaction mixture.

Based on these results, the cyclic voltammetry study (***see 3.3.***), UV/Vis absorption spectroscopy (***see 3.1.***), and DFT-calculations, an energy-transfer pathway for the ring-opening and further formal [3+2] cycloaddition of **4a** is assumed, rendering the whole protocol an energy-transfer cascade.

Note: Excited-state potentials as well as triplet energies were taken from the literature.[25–27]

4. HIGH-THROUGHPUT EXPERIMENTS AND METHOD LIMITATIONS

4.1. General Experimental

High-throughput experimentation (HTE) was performed in a semi-automated fashion utilising an OT-2 liquid handler (Opentrons, SKU 999-00111) equipped with a P300 Single-Channel GEN2 and P300 8-Channel pipette. For all automated liquid transfers, 300 µL OT-2 Tips (Opentrons, SKU 999-00009) were used. Stock solutions were prepared manually in scintillation vials (20 mL, Thermo Fisher Scientific, FS7450420; 8 mL, Thermo Fisher Scientific, No. 10504463; 4 mL, Th. Geyer, No. 7613421). For manual reaction setups, custom-made polytetrafluorethylene (PTFE) reservoirs (**Figure S8**) were used in combination with piston pipettes (Thermo Fisher Scientific, 1 channel, No. 11885762; 8 channels, No. 11825772; 12 channels, No. 11865772). Reaction arrays were conducted in 96-Well Block Assemblies (Analytical Sales and Services, Photoredox, SKU 96973) equipped with glass shell vials (Analytical Sales and Services, SKU 84001-CASE). The reactions were set up in a custom glove box (**Figure S9**) to avoid any influence from O_2 and were irradiated with 445 nm Lumidox® II 96-Well LED Arrays (Analytical Sales and Services, SKU LUM296LS445) equipped with a custom water-cooled base. Additional cooling was provided by a cooling fan (Noctua NF-A14 industrialPPC-3000, No. 8590889) positioned in front of the reaction block. The assembly of LED array and 96-well block was placed on an orbital shaker (Grant-Bio, PMS-1000i) for mixing (**Figure S10**). Reported temperatures refer to the temperature of the 96-well block. Crude reaction mixtures were filtered through 96 PTFE membrane filter plates (Macherey-Nagel, No. 738660.M) filled with approximately 125 mg silica per well. The filtrate was collected in 96 PP deep well plates (Starlab, No. S1896-1110). Samples for GC analysis were prepared in GC vials (Thermo Fisher Scientific, No.16318367) with micro inserts (VWR, No. 548-0006A).

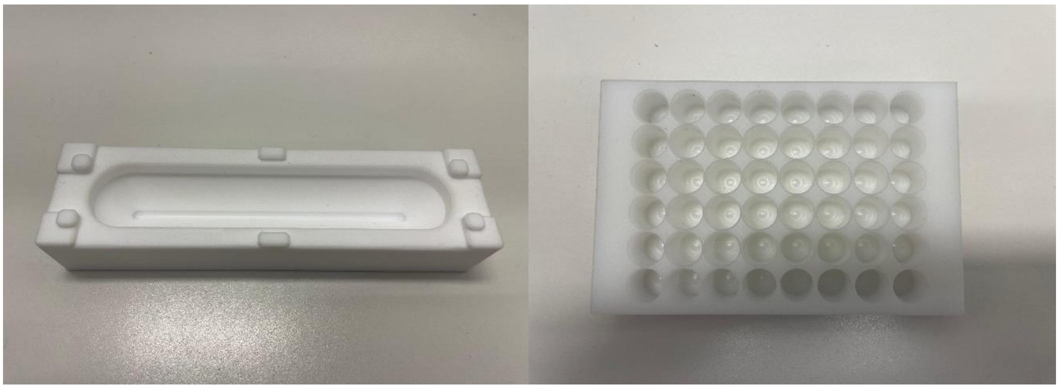

Figure S8: Left: Bar graph Custom-made polytetrafluorethylene (PTFE) reservoir. Right: Custom-made 48-GC vial holders.

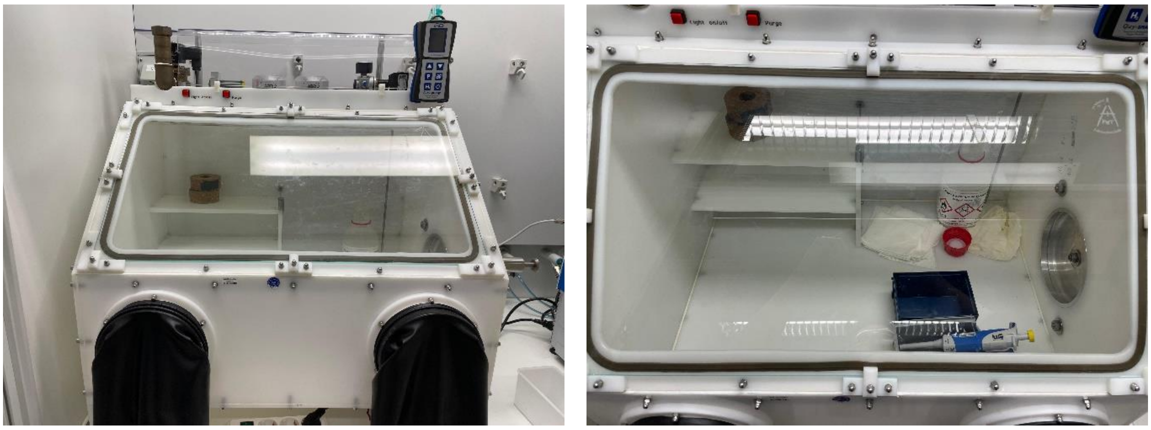

Figure S9: Custom glove box equipped with Orbitec OXY SMART Oxygen Analyser.

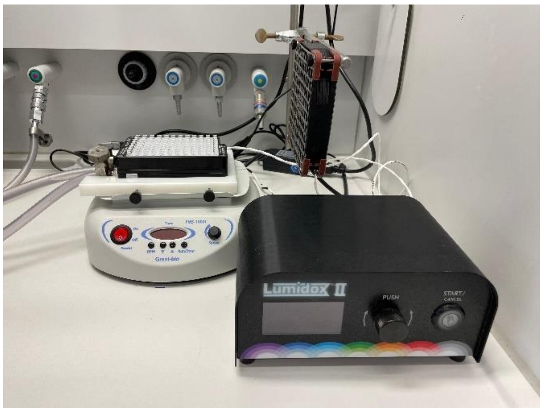

Figure S10: Photo setup for 96-well blocks with 445 nm Lumidox® II 96-Well LED Array equipped with a custom water-cooled base, a Noctua NF-A14 industrial PPC-3000 cooling fan and a Grant-Bio PMS-1000i orbital shaker.

4.1.2. Formal [3+2] Cycloaddition of Strained Azirine Intermediates via Cascade EnT of Alkenes with Cyclic Oxime Esters in 96 Well-plate

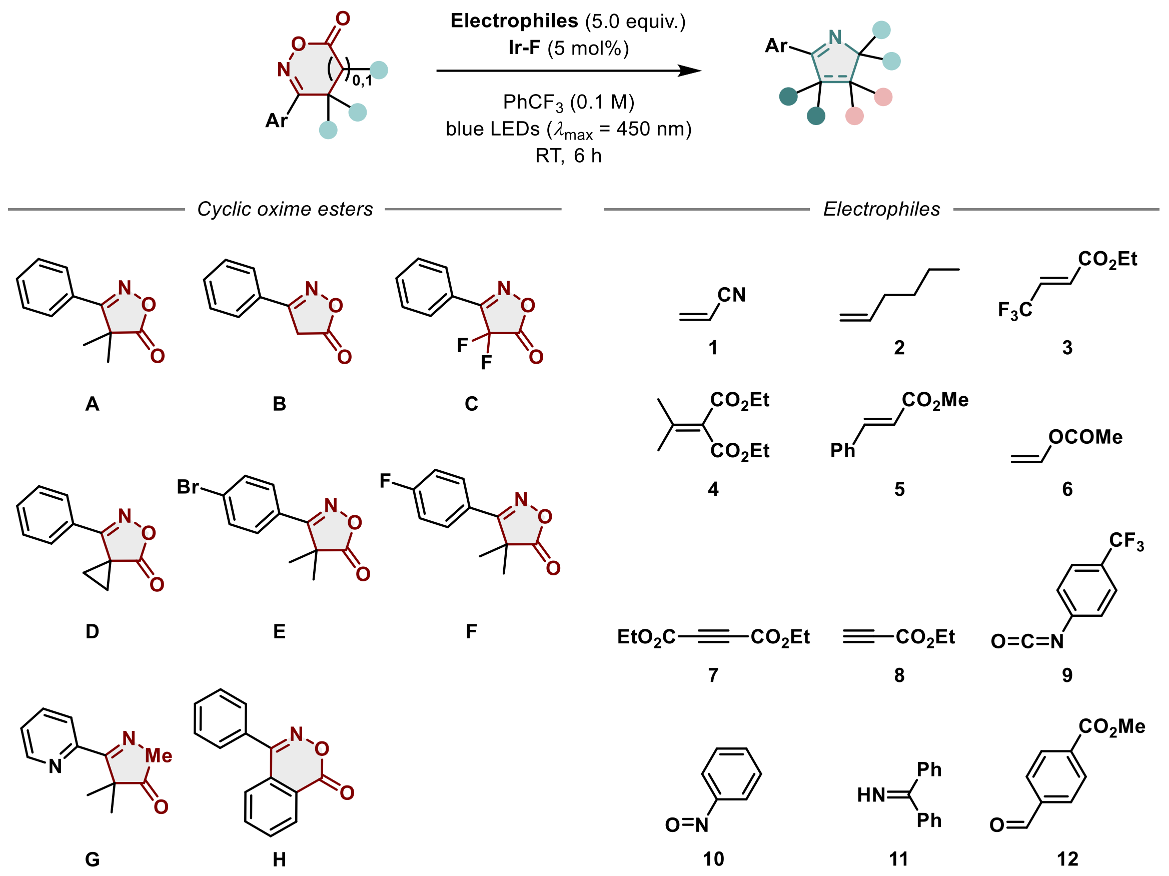

Figure S11: Evaluated scope for the cyclic oxime-enabled synthesis of partially saturated N-heterocycles via cascade energy transfer.

The oxime esters **A-H** (1.00 equiv.) and the electrophiles **1-12** (5.00 equiv.) were weighted into screw neck vials equipped with a rubber septum under air. All vials were purged with argon before the contents were dissolved in 0.80 mL and 1.10 mL trifluorotoluene ($PhCF_3$), respectively (**Table S5**). The catalyst Ir-F (140.20 mg, 0.125 mmol) was weighted into an 8 mL screw neck vial equipped with a rubber septum under air and dissolved in $PhCF_3$ as specified in **Table S5**. The obtained solutions were degassed by argon sparging for 1 minute. All stock solutions, the 96-Well Block Assemblies equipped with 96 stirring bars, custom-made polytetrafluorethylene (PTFE) reservoirs, 8- and 12-channel piston pipettes and a screwdriver were then transferred into a glove box under a positive pressure of argon with oxygen levels not exceeding 100 ppm. One stock solution at a time was then filled into a PTFE reservoir, starting with the oxime esters **A-H**. 50 µL of the corresponding stock solution (0.02 mmol, 1.0 equiv.) was dispensed into one row of the well plate. Next, 100 µL of the corresponding electrophiles **1-12** stock solution (0.026 mmol, 1.3 equiv.) was dispensed into one column **1-12** of the well plate. Then 50 µL of the catalyst **Ir-F** stock solution (0.125 mmol, 5.0 mol%) was dispensed into every well of the array, resulting in a total volume of 200 µL per well. The 96-well plate was then sealed, removed from the glovebox, and placed on the Lumidox® II 96-well LED arrays at 445 nm with a custom water cooling and cooling fan for 6 hours under irradiation at 36°C (details in section 2.11.1). Dodecane (852.00 mg,

5.00 mmol) was weighed into a 25 mL volumetric flask and dissolved in EtOAc. After 6 hours, the plate was unscrewed and 100 µL of the dodecane stock solution (0.02 mmol, 1.00 equiv.) was added to each well. Then the workup was conducted using the Opentrons OT2 pipetting robot according to the procedure specified in the following section.

Table S5: Measurements and solvent volumes for stock solutions.

Compound	IUPAC Name	CAS Number	MW (g/mol)	*m* (mg)	*V* (mL)
**1**	Acrylonitrile	107-13-1	53.06	58.4	1.10
**2**	Hex-1-ene	592-41-6	84.16	92.6	1.10
**3**	Ethyl (*E*)-4,4,4-trifluorobut-2-enoate	406-10-0	168.12	184.9	1.10
**4**	Diethyl 2-(propan-2-ylidene)malonate	6802-75-1	200.23	220.3	1.10
**5**	Methyl cinnamate	103-26-4	162.19	178.4	1.10
**6**	Vinyl acetate	108-05-4	86.09	94.7	1.10
**7**	Diethyl but-2-ynedioate	762-21-0	170.16	187.2	1.10
**8**	Ethyl propiolate	623-47-2	98.10	107.9	1.10
**9**	1-Isocyanato-4-(trifluoromethyl)benzene	1548-13-6	187.12	205.8	1.10
**10**	Nitrosobenzene	586-96-9	107.11	117.8	1.10
**11**	Diphenylmethanimine	1013-88-3	181.24	199.4	1.10
**12**	Methyl 4-formylbenzoate	1571-08-0	164.16	180.6	1.10
**A**	4,4-Dimethyl-3-phenylisoxazol-5(4*H*)-one	/	189.21	60.5	0.80
**B**	3-Phenylisoxazol-5(4*H*)-one	/	161.16	51.6	0.80
**C**	4,4-Difluoro-3-phenylisoxazol-5(4*H*)-one	/	197.14	63.1	0.80
**D**	7-Phenyl-5-oxa-6-azaspiro[2.4]hept-6-en-4-one	/	187.20	59.9	0.80
**E**	3-(4-Bromophenyl)-4,4-dimethylisoxazol-5(4*H*)-one	/	268.11	85.8	0.80
**F**	3-(4-Fluorophenyl)-4,4-dimethylisoxazol-5(4*H*)-one	/	207.20	66.3	0.80
**G**	4,4-Dimethyl-3-(pyridin-4-yl)isoxazol-5(4*H*)-one	/	190.20	60.9	0.80
**H**	4-Phenyl-1*H*-benzo[d][1,2]oxazin-1-one	/	223.23	71.4	0.80

**PC**	[4,4′-*Bis*(1,1-dimethylethyl)-2,2′-bipyridin-*N*1,*N*1′]*bis*[3,5-difluoro-2-[5-(trifluormethyl)-2-pyridinyl-*N*]phenyl-C] iridium(III) hexafluorophosphate	870987-63-6	1121.91	140.2	6.25
**Standard**	Dodecane	112-40-3	170.34	851.7	25.00

4.1.3. Automated Reaction Workup and Sample Preparation

Reaction workup and sample preparation were automated via Python scripts for the OT-2 liquid handler. In the workup protocol, a sample of each crude reaction mixture (60 µL) was aspirated from the 96-reaction block after mixing and transferred to a 96-filter plate on top of a deep well plate. Each well of the filter plate was filled beforehand with 125 mg of silica. Afterwards, the filter was flushed four times with 250 µL EtOAc at intervals of 5 minutes. Afterwards the robot sent a telegram message to the user for manual removal of the filter plate. After removing the filter plate, the GC sample preparation followed. Each filtrate in the deep well plate was mixed, and a sample of 140 µL was transferred into GC vials with micro inserts positioned in custom 48 GC vial holders (**Figure S12**).

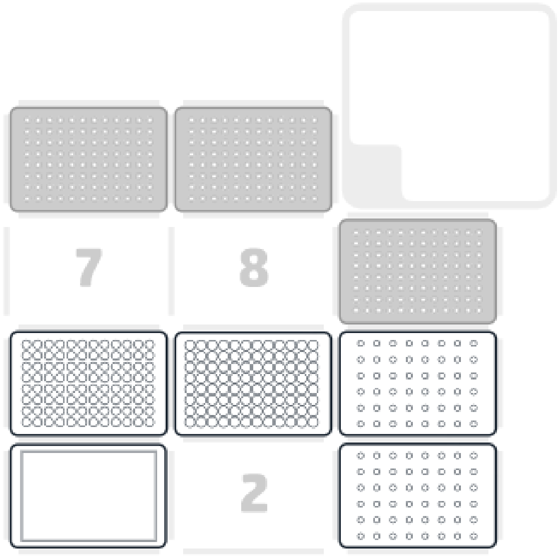

Figure S12: Single well reservoir (Pos. 1), 48 GC vial racks (Pos. 3, 6), Paradox 96-Well Block Assembly (Pos. 4), 96-deep well plate with 96-filter plate (Pos. 5), Labware: 300 µL tipracks (Pos. 9, 10, 11).

pyLurch Python script: https://github.com/FlorianBoser/pyLurch/tree/Double_EnT/examples/double_EnT

```
from opentrons import protocol_api
import time
import itertools
from itertools import product
# Telegram Integration
import os
import sys
# add path for opentrons builtroot linux
if os.name == 'posix':
    sys.path.append('/var/user-packages/usr/lib/python3.10/site-packages')
import telebot

# metadata
metadata = {
    'protocolName': 'Workup for Carboimination of Alkenes with Cyclic Oxime Esters',
    'author': 'Florian Boser <florian.boser@uni-muenster.de>, Dr. Alessia Petti <alessia.petti@uni-
muenster.de>',
    'description': 'Protocol to mix and filter crude reactions through silica and 96-well PTFE
filter. Preparation of samples for GC/MS for yield estimation.',
    'apiLevel': '2.15'}

def run(protocol: protocol_api.ProtocolContext):
    plate = protocol.load_labware('asparadox_96_wellplate_1000ul', location='4')
    filter_deck = protocol.load_labware('custom_small_96_filter_deck_1500ul', location='5')
    collection_plate = protocol.load_labware('custom_96_lowerdeck_2000ul', protocol_api.OFF_DECK)
    reservoir_1 = protocol.load_labware('starlab_1_reservoir_240000ul', location='1')
    tiprack_300_1 = protocol.load_labware('opentrons_96_tiprack_300ul', location='10')
    tiprack_300_2 = protocol.load_labware('opentrons_96_tiprack_300ul', location='11')
    tiprack_300_3 = protocol.load_labware('opentrons_96_tiprack_300ul', location='9')
    vials_holder_1 = protocol.load_labware('custom_48_gcvial_rack_inlays_100ul', location='3')
    vials_holder_2 = protocol.load_labware('custom_48_gcvial_rack_inlays_100ul', location='6')

    # Initialisation pipettes
    multi_pipette = protocol.load_instrument('p300_multi_gen2', mount='left',
tip_racks=[tiprack_300_1, tiprack_300_2, tiprack_300_3])
    multi_pipette.flow_rate.aspirate = 60
    multi_pipette.flow_rate.dispense = 20
    multi_pipette.well_bottom_clearance.aspirate = 4.5
    multi_pipette.well_bottom_clearance.dispense = 9
    single_pipette = protocol.load_instrument('p300_single_gen2', mount='right',
tip_racks=[tiprack_300_1, tiprack_300_2, tiprack_300_3])
    single_pipette.well_bottom_clearance.aspirate = 10

    # Telegram Messaging
    BOT_TOKEN = "INSERT_YOUR_PERSONAL_BOT_TOKEN"
    bot = telebot.TeleBot(BOT_TOKEN)

    # Mix and transfer mixtures to filter plate
    paired_columns = zip(plate.columns(), filter_deck.columns())
    for pair in paired_columns:
        origin = pair[0][0]
        target = pair[1][0]
        multi_pipette.pick_up_tip()
        multi_pipette.mix(3, 200, origin)
        multi_pipette.aspirate(60, origin)
        multi_pipette.touch_tip()
        multi_pipette.air_gap(10)
        multi_pipette.dispense(60, target)
        multi_pipette.blow_out()
        multi_pipette.drop_tip()

    # # # Flushing down the filter plate with EtOAc
    multi_pipette.pick_up_tip()
    for iteration in range(4):
        filter_deck_wells = [well.bottom(20) for well in filter_deck.wells()]
        multi_pipette.transfer(250, reservoir_1['A1'], filter_deck_wells, air_gap=5, new_tip='never',
blow_out=True,
                               blowout_location='destination well')
        if iteration != 3:
            multi_pipette.move_to(multi_pipette.trash_container['A1'].top())
            multi_pipette.blow_out()
            protocol.delay(minutes=5)
    multi_pipette.drop_tip()

    bot.send_message('ENTER_YOUR_PERSONAL_TELEGRAM_ID', 'Please remove the filter deck!')
    # Manually remove filter plate
    protocol.move_labware(labware=filter_deck, new_location=protocol_api.OFF_DECK)
```

4.1.4. Gas Chromatographic Analysis

Samples for GC were filtered over a pad of silica and eluted with EtOAc before analysis. GC-MS spectra were recorded on an Agilent Technologies 7890A GC-system (HP-5MS column: 0.25 mm × 30 m, film: 0.25 µm) with an Agilent 5977B Mass Selective Detector (MSD). All samples were measured in split mode (split ratio: 99:1, split flow 99 mL/min) with an injection volume of 1 µL at an inlet temperature of 300 °C and a septum purge flow of 3 mL/min. The column flow was set to 1 mL/min. The temperature program started at an initial oven temperature of 80 °C followed by a 20 °C/min ramp until 310 °C. The final temperature was held for 1 min. The MS detector was operated with a solvent delay of 1.75 min.

4.1.5. GC-MS Evaluation and Qualitative Yield Estimation Using PyGecko Library

pyGecko is an open-source Python3 library for the processing, analysis and visualization of GC-MS and GC-FID data (available on GitHub at https://github.com/FelixKatz77/pyGecko). pyGecko was developed by our group to perform calibration-free, accurate quantification of high-throughput experiments by automated processing and combination of GC-MS and GC-Polyarc-FID raw data.[28] pyGecko builds up on the program packages SciPy, RDKit, ProteoWizard, pymzML, Pyteomics and BRAIN.

Since pyGecko is implemented in Python3, the modular architecture and flexibility of pyGecko allows easy adaptation to different screening, automation and synthetic chemistry workflows. Therefore, pyGecko was modified accordingly to allow an estimation of product yields based on GC-MS spectra only. Based on the original pyGecko library (available on GitHub at https://github.com/FelixKatz77/pyGecko), a new function was developed (ms_only_quantify_plate), in which only the MS spectra is used for qualitative yield estimation (available on GitHub at https://github.com/FlorianBoser/pyLurch/tree/Double_EnT). The quantification of the product peak in the MS chromatogram, which is determined based on the known molecular mass of the product and the intensity of +1 and +2 isotope peak in the spectrum, is either relative to the area of all other peaks in the spectrum (which could indicate side reactions or remaining starting material) or relative to an internal standard. In our experiment, a hit identification of the products in the GC-MS relative to an internal standard dodecane was carried out. It is expressly pointed out that this does not lead to an exact quantification and is largely dependent on the ionizability and stability of the distinct products. However, repeats of a selection of the high-throughput reactions were performed on batch scale and were shown to give comparable results to the GC-MS quantification. Estimations of reaction yields were automatically calculated by pyGecko as the ratio of the peak area of an analyte and the standard dodecane in the MS chromatogram. The classification of yields was as follows: Major hit for ratios ≥25%, minor hit for ratios ≥ 5% and trace for ratios < 5% (**Figure S13**).

Due to the possible formation of different isomers of some products, the identification of the product peak in pyGecko was additionally modified. In pyGecko, the molecular mass of the compound is calculated and a signal with the corresponding m/z value is searched for in all peaks of an injection. To minimize false-positive assignments, the intensity of the +1 or +2 isotope peak is calculated (using the BRAIN algorithm[29,30]) and compared to the intensity of the signal in the chromatogram. The compound is assigned to the peak if the deviation between the calculated and the measured intensity is below a threshold value. If several peaks fulfill these criteria, the compound is assigned to the peak with the smallest deviation from the calculated intensity.

However, due to isomers, different peaks per chromatogram meet these criteria and may cause errors in the automated evaluation. To determine the yield of the main product isomer, the largest peak, which is below the defined threshold of 5% of the calculated intensity, was selected in this version of pyGecko (available on GitHub at https://github.com/FlorianBoser/pyLurch/tree/Double_EnT).

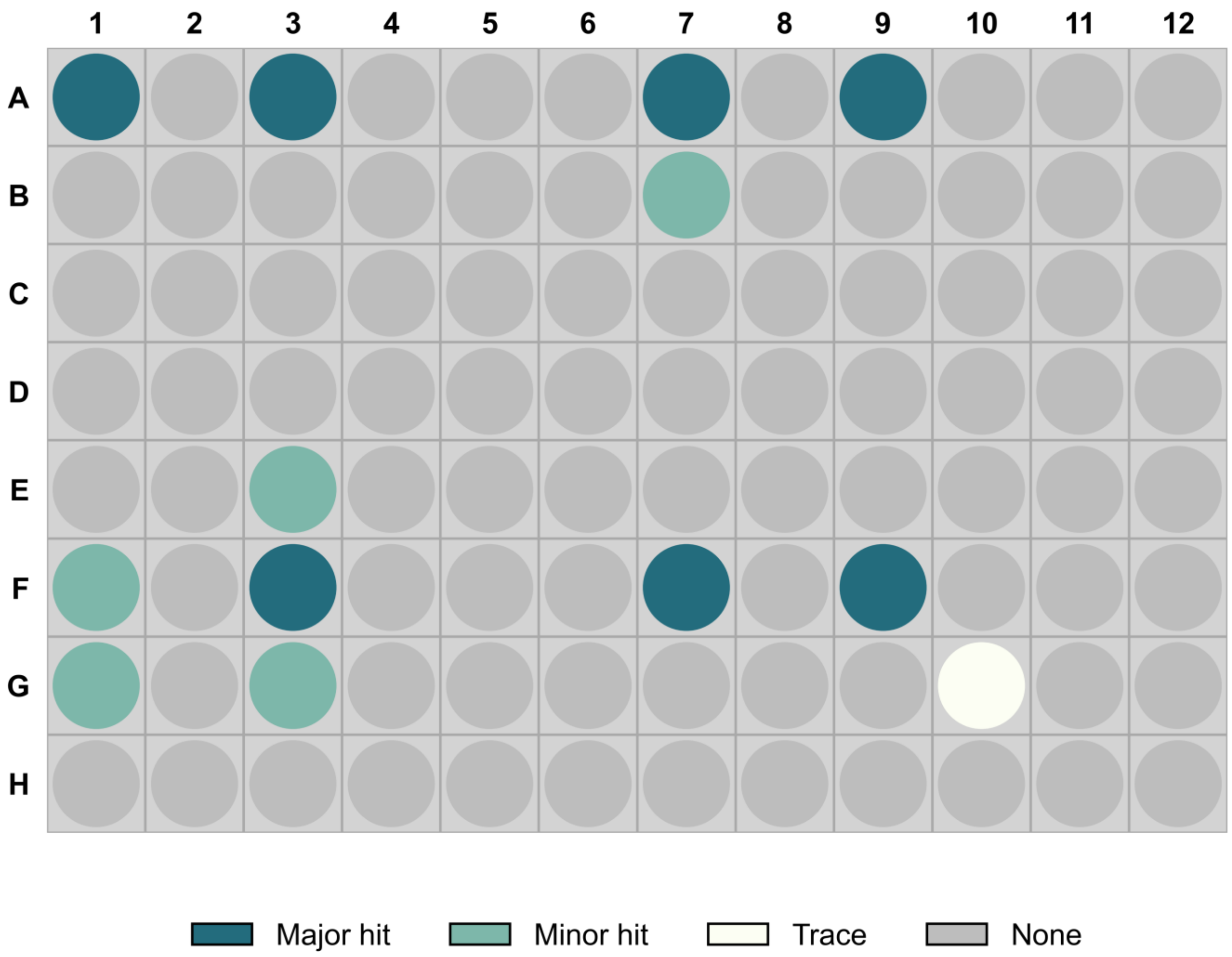

Figure S13: Heatmap illustrating the hit identification of the cyclic oximes enabled cycloaddition of strained azirines intermediates via cascade EnT.

pyLurch Python script: https://github.com/FlorianBoser/pyLurch/tree/Double_EnT/examples/double_EnT

```
from pygecko.parsers import MS_Base_Parser
from pygecko.parsers.msconvert_wraper import msconvert
from pygecko.reaction import Transformation, Product_Array
from pygecko.visualization.visuals import Visualization
from pygecko.analysis.analysis import Analysis
from pathlib import Path

def main():

    # Define input and output paths.
    ms_raw = '/raw_MS'
    mzml_path = '/mzML'

    layout_path = '/product_smiles.csv'
    layout = Product_Array(layout_path)

    convert_to_mzml(ms_raw, mzml_path)
    ms_sequence = MS_Base_Parser.load_sequence(mzml_path, pos=True)

    # Pick peaks in the GC-MS
    ms_sequence.pick_peaks()
    ms_sequence.set_internal_standard(3.32, name='Dodecane', smiles='CCCCCCCCCCCC')

    # Identify hits for the reactions.
    yield_array = Analysis.calc_plate_ms_only_yield(ms_sequence, layout)

    # Generate plate heatmap.
    Visualization.visualize_plate_qualitative(yield_array['quantity'], well_labels=True,
                                              row_labels=["A", "B", "C", "D", "E", "F", "G", "H"],
                                              col_labels=["1", "2", "3", "4", "5", "6", "7", "8", "9", "10",
"11",
                                                          "12"] , path='results/Heatmap_Double_EnT.png')

    print("Done! Best wishes from the Glorius Group!")

if __name__ == '__main__':
    def convert_to_mzml(ms_raw: str, output_dir: str):
        raw_directory = Path(ms_raw)
        supported_formats = ['.D']
        raw_files = []
        for file_format in supported_formats:
            raw_files.extend(raw_directory.glob(f'*{file_format}'))
        msconvert(input_files = raw_files, output_dir=output_dir, format='mzML')

    main()
```

5. COMPUTATIONAL STUDIES

5.1. DFT Calculations

DFT calculations were performed using the ORCA 5.0.3 software package[31] on a local high-performance computing cluster, equipped with Intel® Xeon® Gold 6240 CPUs. Resulting structures were visualized in *Avogadro*.[32] For the initial guess structure generation, an adapted workflow, originally described in the *EnTdecker* project, was utilized.[33] This workflow samples conformers using RDKit starting from SMILES notations and pre-optimizes the obtained lowest-energy conformer using GFN2-xTB.[34]

Geometry optimizations and frequency calculations were conducted using the B3LYP functional[35] combined with the def2-SVP basis set.[36] No constraints on internal coordinates or symmetry were imposed. In case of convergence issues, the *TightOpt* and *VerySlowConv* keywords were set. Additional single-point calculations using the ωB97X-D3 functional[37] and def2-TZVPP basis set[36] were performed on each optimized structure to achieve more accurate electronic energies. All calculations included GRIMME's D3 dispersion correction[38], the CPCM implicit solvation model[39] for α,α,α-trifluorotoluene (ε = 9.2)[40], and the RIJCOSX approximation.[41]

All intermediates were confirmed to represent local minima on the potential energy surface by verifying the absence of imaginary frequencies after optimization. Transition states (TS) were confirmed to represent saddle points on the potential energy surface by the presence of only one negative eigenvalue of the Hessian matrix, resulting in a single imaginary vibrational frequence. Intrinsic reaction coordinate (IRC) calculations were additionally performed to confirm that the TS is connecting the respective intermediates.

The reported free energies were calculated as the sum of the electronic energies at the ωB97X/def2-TZVPP level of theory and the free-energy corrections (zero-point energy, thermal correction, thermal enthalpy correction, entropy) at the level of theory of the geometry optimization and frequency calculations.

5.1.1. Calculated Energies

Table S6: DFT calculated energies, optimized structures and evaluated free energy corrections (ZPVE, thermal corrections, enthalpy corrections, entropic corrections) at B3LYP(D3)/def2-SVP level of theory. Electronic energy evaluated at ωB97X-D3/def2-TZVPP level of theory. All energy values are given in Hartee (E_h).

	**Electronic Energy (E)**	**Free Energy Corrections ($\Delta G_{corr.}$)**	**Free Energy ($G = E + \Delta G_{corr.}$)**
**^{1}1a**	-631.0372333	0.16151735	-630.8757160
**^{3}1a**	-630.9380045	0.15659579	-630.7814087
**^{3}TS-1**	-630.9148178	0.15862063	-630.7561972
**^{3}IM-1'**	-630.9427910	0.15630145	-630.7864896
**^{3}IM-1**	-442.3863555	0.14479034	-442.2415652
**^{3}IM-1 + 2a**	-613.2403825	0.19028903	-613.0500935
**^{3}TS-2**	-613.2229072	0.19318394	-613.0297232
**MECP-1**	-442.3755804	(0.14256633)	(-442.2330141)
**^{3}IM-2**	-613.2584095	0.19598602	-613.0624235
**^{1}4a**	-442.4287434	0.14898378	-442.2797596
**^{3}4a**	-442.3137081	0.14337840	-442.1703297
**^{3}TS-3a**	-442.3061536	0.14102295	-442.1651307
**^{3}TS-3b**	-442.3024944	0.14370627	-442.1587881
**^{3}IM-3a**	-442.3747028	0.14437241	-442.2303304
**^{3}IM-3b = ^{3}IM-2**	-442.3863555	0.14479034	-442.2415652
**^{3}IM-3a + 2a** (addition of internal radical)	-613.2275891	0.18980821	-613.0377809
**^{3}IM-3b + 2a** (addition of terminal radical)	-613.2291927	0.19007574	-613.0391169
**^{3}TS-4a**	-613.2195061	0.19124357	-613.0282625
**^{3}TS-4b**	-613.2102280	0.19197546	-613.0182525
**^{3}IM-4a**	-613.2729990	0.19491990	-613.0780791
**^{3}IM-4b**	-613.2480265	0.19482619	-613.0532003
**MECP-2**	-613.2655635	0.19374701	-613.0718165
**^{1}3a**	-613.3751544	0.20392445	-613.171230
**$^1CO_2$**	-188.6158577	-0.00424842	-188.6201061
**^{1}2a**	-170.8493138	0.02542911	-170.8238846

5.1.2. Optimized Geometries

[1]Isoxazolone

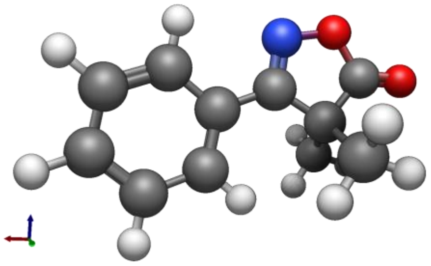

O	-3.884995973	-0.613424575	1.235204548
C	-2.685908894	-0.551416112	1.254268588
C	-1.716345840	-0.153615907	0.145605992
C	-2.016348815	1.301739097	-0.267378474
C	-1.897538113	-1.135306211	-1.029972184
C	-0.407669338	-0.313417079	0.894159932
C	0.954746038	-0.068329758	0.388713382
C	1.190994323	0.290978130	-0.950209442
C	2.492980070	0.515633789	-1.405800483
C	3.576321030	0.385897898	-0.533617394
C	3.352221652	0.029748549	0.803239449
C	2.056523645	-0.194807191	1.261048879
N	-0.577185405	-0.699537798	2.114660660
O	-1.962305233	-0.854697912	2.367240231
H	-1.369586134	1.618258559	-1.096689757
H	-3.064412531	1.365988042	-0.596378817
H	-1.869537612	1.991597095	0.577159858
H	-1.639315335	-2.162651201	-0.732343269
H	-2.951765357	-1.114944722	-1.344530945
H	-1.274089349	-0.848800636	-1.886712014
H	0.364714441	0.396489890	-1.651200665
H	2.656885133	0.793155131	-2.449760290
H	4.593881048	0.562329889	-0.891312069
H	4.194593559	-0.071967378	1.491965434
H	1.883142990	-0.468859588	2.302658851

[3]TS-1

(imaginary frequency: -754.60 cm^{-1})

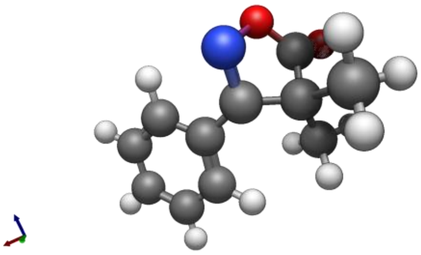

O	-2.883103785	-1.805639615	1.117268216
C	-2.091789770	-0.909254622	1.237372762
C	-1.702611503	0.082362546	0.095036201
C	-2.751295525	1.211242085	0.099998394
C	-1.693276787	-0.635830148	-1.246872264
C	-0.379994399	0.515125041	0.700796549
C	0.973893160	0.216642765	0.271108723
C	1.330555929	0.177252471	-1.092754134
C	2.650087482	-0.058865023	-1.462686347
C	3.628848274	-0.270905270	-0.488845481
C	3.288435898	-0.226795391	0.867377628
C	1.979100543	0.028162900	1.248005714
N	-0.626539256	0.805939403	1.980603769
O	-1.473431295	-0.637459264	2.374432493
H	-2.470515669	1.969030415	-0.643936908
H	-3.730184563	0.792449441	-0.170745033
H	-2.830554448	1.691436013	1.084388133
H	-0.924864153	-1.418787610	-1.293422415
H	-2.676092088	-1.109316796	-1.376126696
H	-1.544455824	0.074740300	-2.070555928
H	0.581152172	0.359861974	-1.860893423
H	2.918506783	-0.074699461	-2.519881563
H	4.661259168	-0.463317223	-0.785249542
H	4.052564958	-0.385969473	1.629623884
H	1.714304698	0.072594540	2.305957265

[3]Isoxazolone

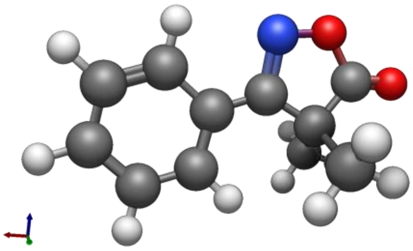

O	-3.825818833	-0.670083627	1.297938833
C	-2.636320585	-0.556264501	1.270660827
C	-1.707641520	-0.144426412	0.137675472
C	-2.082989378	1.294041479	-0.292385906
C	-1.905947953	-1.136337610	-1.032251739
C	-0.374065073	-0.252232372	0.828750740
C	0.915708886	-0.044361014	0.368716999
C	1.210282685	0.346861480	-0.994225607
C	2.510500243	0.536677976	-1.407927689
C	3.594714243	0.356297850	-0.514079030
C	3.339154160	-0.021021583	0.819534177
C	2.045709430	-0.218610218	1.264307039
N	-0.541716549	-0.644769684	2.217500926
O	-1.877431016	-0.815980778	2.406952787
H	-1.441565243	1.623848288	-1.121391872
H	-3.129907408	1.315670072	-0.631027486
H	-1.964736826	1.999286158	0.543761452
H	-1.642140316	-2.160618345	-0.729437106
H	-2.958509661	-1.120232833	-1.352999343
H	-1.278199757	-0.851209441	-1.887129830
H	0.395128921	0.491152888	-1.702235972
H	2.708260382	0.830466396	-2.442043912
H	4.620041526	0.509902076	-0.857678401
H	4.173558294	-0.159342939	1.512197437
H	1.853961350	-0.508683306	2.296847204

[3]IM-1’

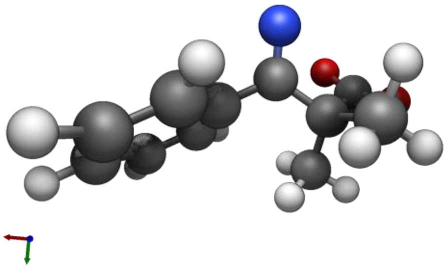

O	-3.801065047	-1.069150285	-1.174732619
C	-2.599415877	-1.163862404	-1.172179254
C	-1.654791623	-0.413189517	-0.236414058
C	-2.285656466	-0.346191846	1.158767269
C	-1.509629690	0.995967744	-0.833208892
C	-0.305087571	-1.159405649	-0.118874656
C	0.976455199	-0.391446515	0.052212031
C	1.587720438	0.238847224	-1.037772045
C	2.779845850	0.936268150	-0.851378306
C	3.361796704	1.005751818	0.416244416
C	2.756367175	0.369412510	1.499638525
C	1.566625863	-0.336854676	1.318707059
N	-0.277699780	-2.407867647	-0.056557006
O	-2.006356827	-1.894751993	-2.091389602
H	-1.634301334	0.240269003	1.822493021
H	-3.264326234	0.147567751	1.093615895
H	-2.414948854	-1.352295806	1.579087220
H	-1.063220739	0.969835183	-1.836338443
H	-2.506173063	1.453045494	-0.902024633
H	-0.881248552	1.614654517	-0.178798193
H	1.136385880	0.175446062	-2.029714919
H	3.259557602	1.424828463	-1.700870929
H	4.295605864	1.552488406	0.556824959
H	3.213304922	0.418079394	2.489229164
H	1.086846160	-0.843275380	2.158413994

^{3}IM-1

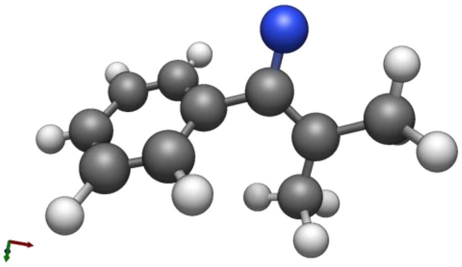

C	1.806939389	0.862033313	0.757280459
C	1.794162890	-0.126251642	-0.363007979
C	3.107204301	-0.442686258	-1.005238946
N	0.652692481	-1.678596909	-1.752578753
C	0.612728512	-0.748529418	-0.840288370
C	-0.756999404	-0.397137795	-0.331758395
C	-1.205193252	0.929675506	-0.335241790
C	-2.496477976	1.235035921	0.092280217
C	-3.351415276	0.218374269	0.522809897
C	-2.911714942	-1.105822720	0.520450377
C	-1.621866627	-1.414495626	0.088718046
H	2.681051924	0.677031436	1.400379186
H	0.898416634	0.825528093	1.370834305
H	1.914592987	1.889648202	0.367053560
H	3.010220789	-1.197362249	-1.794518374
H	3.539359281	0.473213084	-1.442980456
H	3.827629126	-0.798475398	-0.251610658
H	-0.541239767	1.724465423	-0.681872305
H	-2.837950353	2.271489563	0.084088754
H	-4.362571274	0.459141716	0.855521586
H	-3.576639318	-1.904227469	0.854101981
H	-1.275220127	-2.449531042	0.080837659

MECP-1

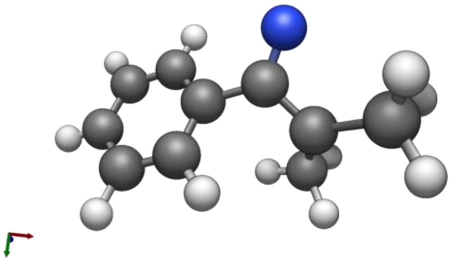

C	1.930651073	0.644591981	0.937350962
C	1.772701584	-0.027182194	-0.386938241
C	2.969018089	-0.118710201	-1.272729647
N	0.888293707	-2.060121715	-1.065621315
C	0.615307594	-0.914037946	-0.613400709
C	-0.772970605	-0.487076350	-0.251690315
C	-1.134626504	0.861329834	-0.341418066
C	-2.434269188	1.258391555	-0.028751257
C	-3.375380335	0.310198252	0.375747634
C	-3.017382820	-1.037066852	0.462409288
C	-1.720723838	-1.437023008	0.146093790
H	2.647522013	0.084988378	1.565633533
H	0.984143929	0.722708539	1.487434770
H	2.353667439	1.652989629	0.802434605
H	2.708208934	-0.501311808	-2.267862109
H	3.437802072	0.873347652	-1.366212917
H	3.735382534	-0.789016567	-0.843301748
H	-0.395280977	1.598053868	-0.665326656
H	-2.713406797	2.310853388	-0.101225055
H	-4.391235846	0.620950538	0.626075905
H	-3.751502254	-1.777772211	0.783569209
H	-1.428208806	-2.486564761	0.212989340

^{3}IM-1 + 2a

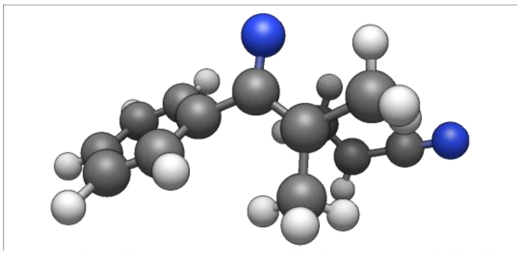

C	2.11686	1.742876	-0.00309
C	2.031254	0.828588	-1.18094
C	3.120178	0.933053	-2.19968
N	1.029609	-0.99019	-2.33214
C	0.988716	-0.11886	-1.36413
C	-0.20606	-0.20451	-0.45878
C	-0.96066	0.934564	-0.15134
C	-2.09261	0.82678	0.654891
C	-2.48297	-0.41762	1.153851
C	-1.74137	-1.55728	0.840469
C	-0.60903	-1.45228	0.033333
H	3.161014	1.787826	0.346022
H	1.47752	1.4282	0.830826
H	1.835076	2.772387	-0.28586
H	2.999283	0.206703	-3.01236
H	3.13258	1.948175	-2.63126
H	4.10588	0.78554	-1.7273
H	-0.02536	-2.33958	-0.22041
H	-0.662	1.905849	-0.55114
H	-2.67599	1.717862	0.89263
H	-2.04701	-2.53221	1.223697
H	-3.3692	-0.49622	1.785952
C	3.306669	-1.63721	0.25538
C	4.345498	-1.03061	0.83948
H	2.40338	-1.85561	0.829279
H	3.325596	-1.92703	-0.79782
N	6.501493	-0.4606	-0.48677
C	5.543172	-0.71934	0.105619
H	4.332165	-0.73405	1.890952

^{1}Azirine

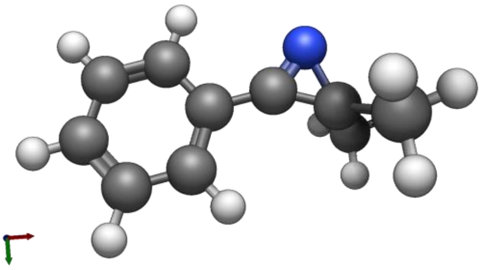

C	2.654240935	0.477313229	1.133159951
C	1.997650824	-0.184017661	-0.059051313
C	2.677148540	0.067715908	-1.387153047
N	1.421258420	-1.573113902	0.159632957
C	0.613993226	-0.621296684	0.001480240
C	-0.815118050	-0.350850370	-0.044905563
C	-1.267694220	0.960216801	-0.236754659
C	-2.636596897	1.220230406	-0.274445942
C	-3.547289597	0.173139407	-0.121907012
C	-3.096124636	-1.137168928	0.067887778
C	-1.730775267	-1.402522811	0.107038408
H	3.703158919	0.152465429	1.220608813
H	2.128831714	0.223128441	2.063462712
H	2.651622463	1.572046785	1.018519147
H	2.156687622	-0.452236242	-2.202658997
H	2.696286339	1.144508898	-1.614904706
H	3.720068852	-0.285463612	-1.354441338
H	-0.539101783	1.765132217	-0.355567607
H	-2.996271820	2.239600569	-0.422185809
H	-4.618929586	0.378394453	-0.150020043
H	-3.815222264	-1.949040846	0.187859331
H	-1.357843733	-2.418201488	0.254536698

^{3}TS-2

(imaginary frequency: -559.28 cm^{-1})

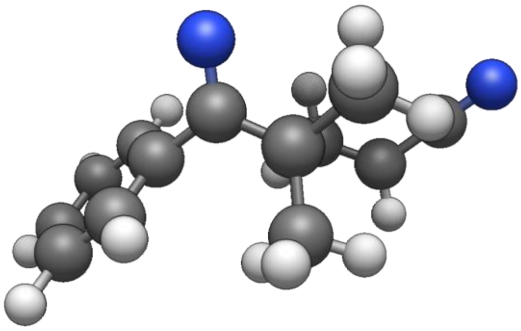

C	0.81408811	1.63105840	0.32103419
C	0.89500012	0.58236603	-0.74946412
C	1.93437265	0.79639671	-1.81131480
N	-0.34510314	-0.81379729	-2.24189335
C	-0.31645158	-0.13979182	-1.16081954
C	-1.53421980	-0.21519106	-0.28097274
C	-2.26914282	0.93310750	0.03867495
C	-3.40803796	0.82898267	0.83523745
C	-3.81863702	-0.41701776	1.31431016
C	-3.09466778	-1.56383053	0.98785552
C	-1.95759607	-1.46499182	0.18593848
H	1.82353257	1.84990758	0.69786648
H	0.17707473	1.32895538	1.16282660
H	0.40703534	2.56719334	-0.09897217
H	2.12165950	-0.11511269	-2.39556657
H	1.58538239	1.57658549	-2.50961493
H	2.87768524	1.14135798	-1.36578108
H	-1.38618507	-2.35696334	-0.07913455
H	-1.95186364	1.90601072	-0.34112984
H	-3.97965035	1.72463286	1.08382402
H	-3.41738362	-2.53924572	1.35520038
H	-4.70835730	-0.49072683	1.94207721
C	1.72764887	-1.09477804	0.36378879
C	2.95346243	-0.70147473	0.84692956
H	0.87964666	-1.11814299	1.05169603
H	1.67777937	-1.74649852	-0.51117883
N	5.11245531	-1.02143007	-0.53414389
C	4.14276564	-0.87328982	0.08438679
H	3.05770720	-0.19427165	1.80833979

^{3}IM-2

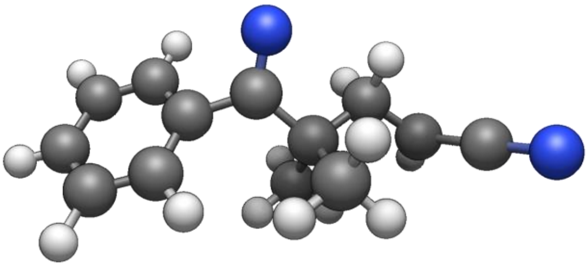

C	2.2008788548	0.5562270962	1.0057798342
C	2.4369305845	-0.0471425009	-0.3813398163
C	2.7647517805	1.0440599385	-1.4104873153
N	1.3023678844	-1.8717237884	-1.5256287827
C	1.1814285624	-0.8236175354	-0.8502728722
C	-0.2129781365	-0.3845782205	-0.4873856105
C	-0.6527407112	0.9361231268	-0.6462778681
C	-1.9624999651	1.2819698816	-0.3180518866
C	-2.8436707535	0.3169740186	0.1722771808
C	-2.4123021059	-1.0004178376	0.3282037170
C	-1.1047792269	-1.3515681710	-0.0042394493
H	3.1173136297	1.0553048857	1.3536661027
H	1.9284496988	-0.2221242221	1.7346111032
H	1.3994479673	1.3059405959	0.9845431022
H	2.9692184673	0.5982525084	-2.3949889325
H	1.9453304713	1.7658005385	-1.5196520366
H	3.6539588699	1.6066838945	-1.0876591455
H	-0.7608993440	-2.3798239360	0.1186600984
H	0.0200181511	1.7009336079	-1.0329064188
H	-2.2960869232	2.3126351797	-0.4485553819
H	-3.0963217241	-1.7591063056	0.7117480516
H	-3.8671664411	0.5918769663	0.4326195889
C	3.6073034694	-1.0737641153	-0.3249634869
C	4.9081014839	-0.4841665207	0.1142095398
C	5.8761508500	-0.0581322722	-0.8066087540
N	6.6687827094	0.2980156529	-1.5822883301
H	3.7234973477	-1.5258341920	-1.3200248805
H	3.3279823362	-1.8728307909	0.3786017977
H	5.1357422131	-0.3442274831	1.1723808513

^{3}Azirine

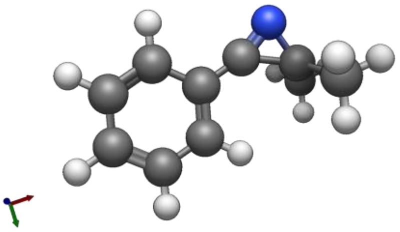

C	2.547691375	0.523495035	1.135202876
C	2.105128063	-0.285170542	-0.044374574
C	2.572787397	0.138792328	-1.401403774
N	1.655523741	-1.624534078	0.151972931
C	0.580272156	-0.929931878	0.041987483
C	-0.742297036	-0.531464781	-0.020825395
C	-1.104717409	0.839635221	-0.234063874
C	-2.436413986	1.208707816	-0.283818415
C	-3.464133807	0.262815234	-0.131673207
C	-3.120040799	-1.083894978	0.072582821
C	-1.800020539	-1.492190544	0.129445306
H	3.642594321	0.429509450	1.234463648
H	2.075766874	0.166390322	2.058464105
H	2.324290804	1.589356680	0.981578693
H	2.127301982	-0.487020559	-2.183939813
H	2.336261981	1.197788176	-1.581968489
H	3.670505199	0.036589730	-1.444049060
H	-0.317888555	1.586936557	-0.357212596
H	-2.686388421	2.259441717	-0.446973055
H	-4.509866524	0.568550678	-0.169872174
H	-3.906941352	-1.832213853	0.193438882
H	-1.549425463	-2.541587730	0.291257682

^{3}TS-3b

(imaginary frequency: -849.88 cm^{-1})

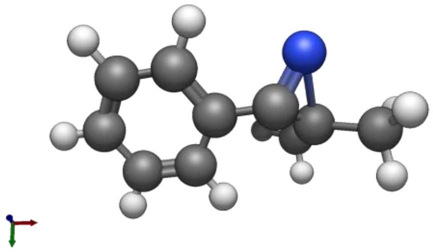

C	1.953457683	0.781642158	0.952208813
C	1.843838914	-0.075724380	-0.270146347
C	3.088405638	-0.201784095	-1.098927962
N	1.284562522	-1.875936816	-0.433984669
C	0.623877434	-0.663415969	-0.661961554
C	-0.718188479	-0.366427221	-0.258466913
C	-1.172280977	0.970265522	-0.153052333
C	-2.486949242	1.240042423	0.203329994
C	-3.384489553	0.195351661	0.457909787
C	-2.948569560	-1.129411362	0.350178888
C	-1.634580942	-1.413878950	-0.003112099
H	2.835313492	0.479322215	1.536434916
H	1.050476879	0.738447439	1.572253635
H	2.121233735	1.824306744	0.635912929
H	2.969490020	-0.916250860	-1.920999956
H	3.280265144	0.796323559	-1.530087473
H	3.955042519	-0.466708637	-0.477327067
H	-0.481813573	1.789227848	-0.368038532
H	-2.822794561	2.276196291	0.278066979
H	-4.417680456	0.414693452	0.731354451
H	-3.644265774	-1.948063700	0.544955276
H	-1.294350864	-2.448217325	-0.086500764

^{3}TS-3a

(imaginary frequency: -474.31 cm^{-1})

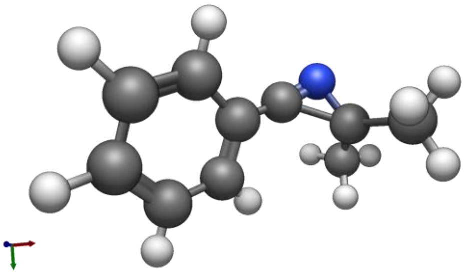

C	2.631434554	0.926688989	0.981138640
C	2.199965250	-0.198338443	0.102187181
C	2.616886380	-0.270622078	-1.324482000
N	1.651118192	-1.380459794	0.678977948
C	0.506737479	-0.867055564	0.580589835
C	-0.793123302	-0.506216816	0.331673442
C	-1.234294724	0.858408909	0.471297048
C	-2.549419240	1.190646070	0.212442972
C	-3.491068831	0.217697078	-0.167229252
C	-3.075029268	-1.121650310	-0.292411305
C	-1.767260680	-1.495994973	-0.060670794
H	3.733799642	0.921389095	1.061949766
H	2.207008487	0.831939266	1.987831220
H	2.349664171	1.898667592	0.547378147
H	1.765887864	-0.580534050	-1.952071595
H	3.003011946	0.690147245	-1.686007926
H	3.398786304	-1.041860391	-1.436862937
H	-0.515484881	1.621023481	0.774950283
H	-2.859390359	2.233322624	0.311330121
H	-4.527197895	0.494502937	-0.362473663
H	-3.796244931	-1.886123360	-0.589937213
H	-1.455786161	-2.535577508	-0.169599917

^{1}IM-3a

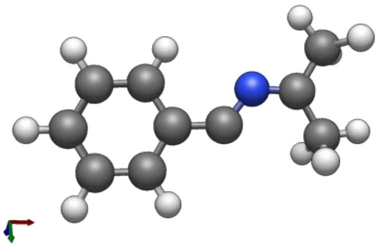

C	3.604943997	0.408742667	-0.447416067
C	2.855522613	-0.730390637	-1.070456036
C	3.604878194	-1.792425075	-1.805377129
N	1.548001305	-0.814549507	-0.982060172
C	0.765959599	0.031225803	-0.379945294
C	-0.663032853	0.002769774	-0.232506656
C	-1.323724936	1.035794782	0.464186158
C	-2.706709955	1.014137942	0.613610110
C	-3.457061697	-0.034583514	0.074217431
C	-2.813227482	-1.065227247	-0.619406740
C	-1.431987821	-1.052664309	-0.774567826
H	4.686550680	0.317358649	-0.603443697
H	3.395759568	0.449700312	0.633996907
H	3.257088108	1.364819082	-0.872212208
H	2.915596387	-2.542991110	-2.209174086
H	4.187218140	-1.348879860	-2.629500282
H	4.331332601	-2.281199148	-1.135547030
H	-0.732618827	1.851803560	0.884956798
H	-3.206482839	1.820589513	1.153221458
H	-4.541633121	-0.049526169	0.193489774
H	-3.396742726	-1.885002902	-1.042955336
H	-0.919798935	-1.852682607	-1.311710078

^{3}IM-3b

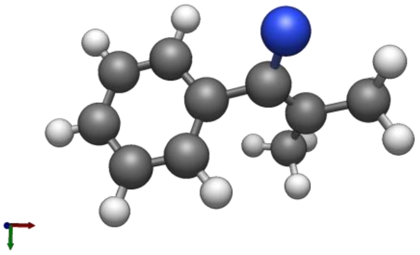

C	1.806939389	0.862033313	0.757280459
C	1.794162890	-0.126251642	-0.363007979
C	3.107204301	-0.442686258	-1.005238946
N	0.652692481	-1.678596909	-1.752578753
C	0.612728512	-0.748529418	-0.840288370
C	-0.756999404	-0.397137795	-0.331758395
C	-1.205193252	0.929675506	-0.335241790
C	-2.496477976	1.235035921	0.092280217
C	-3.351415276	0.218374269	0.522809897
C	-2.911714942	-1.105822720	0.520450377
C	-1.621866627	-1.414495626	0.088718046
H	2.681051924	0.677031436	1.400379186
H	0.898416634	0.825528093	1.370834305
H	1.914592987	1.889648202	0.367053560
H	3.010220789	-1.197362249	-1.794518374
H	3.539359281	0.473213084	-1.442980456
H	3.827629126	-0.798475398	-0.251610658
H	-0.541239767	1.724465423	-0.681872305
H	-2.837950353	2.271489563	0.084088754
H	-4.362571274	0.459141716	0.855521586
H	-3.576639318	-1.904227469	0.854101981
H	-1.275220127	-2.449531042	0.080837659

^{3}IM-3b + 2a

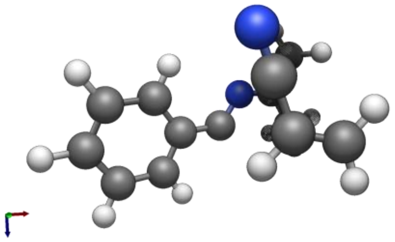

C	3.495760617	-1.367447908	-0.162786736
C	2.806896988	-1.060536326	-1.458997601
C	3.588160798	-1.112936957	-2.730583643
N	1.525157925	-0.778886587	-1.503986684
C	0.744581471	-0.682107520	-0.467596083
C	-0.645797297	-0.327445404	-0.398293154
C	-1.391811058	-0.065201281	-1.570262381
C	-2.733573646	0.286591669	-1.481173168
C	-3.358355363	0.384303754	-0.233043946
C	-2.628972340	0.128996995	0.931724460
C	-1.285848977	-0.224816139	0.854137392
H	4.581298820	-1.456999811	-0.291704022
H	3.107681917	-2.316139239	0.245625847
H	3.276054050	-0.591322447	0.587726847
H	4.032005698	-2.113196259	-2.866161427
H	2.950123856	-0.877419197	-3.590320156
H	4.429261968	-0.401665651	-2.689866915
H	-0.895372836	-0.146879509	-2.538687998
H	-3.301224276	0.489380949	-2.391261416
H	-4.412289023	0.660369496	-0.170704346
H	-3.112218207	0.209627026	1.906990785
H	-0.707675467	-0.422073139	1.758935339
C	3.123298583	2.272785239	-0.521671205
C	1.918540043	2.143410508	0.043377041
C	1.761002660	1.631102182	1.379354651
N	1.633753426	1.215802254	2.450900905
H	4.036657276	2.004916686	0.014300423
H	3.213361078	2.656684476	-1.539400947
H	0.996331317	2.402562139	-0.483091861

^{SssS3}IM-3a + 2a

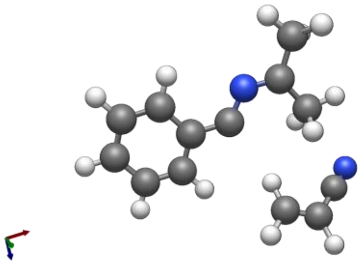

C	2.652758149	-0.304528283	0.305101121
C	1.900156392	-0.669702498	-0.938261356
C	2.648430672	-0.884308598	-2.211851999
N	0.595610704	-0.817385178	-0.929101655
C	-0.194825531	-0.676490865	0.091695240
C	-1.622014985	-0.837056928	0.159359077
C	-2.308540022	-0.629048021	1.372953241
C	-3.689302786	-0.784000841	1.439892590
C	-4.413117099	-1.148621363	0.301106915
C	-3.743288515	-1.359287341	-0.909161594
C	-2.364384657	-1.206200924	-0.986579228
H	3.730352490	-0.217903573	0.121045156
H	2.484717648	-1.065775449	1.085609552
H	2.278114423	0.651920040	0.707840321
H	1.964148042	-1.142408036	-3.028318467
H	3.215721411	0.021655193	-2.480962223
H	3.390264889	-1.689921343	-2.085594059
H	-1.740261310	-0.345893909	2.260917238
H	-4.206028369	-0.619464667	2.386913273
H	-5.496218991	-1.269185835	0.355931038
H	-4.306386232	-1.644824426	-1.799829218
H	-1.834292774	-1.367974949	-1.926416194
C	0.844857003	0.820752532	3.446292385
C	2.007783020	1.046551511	4.064635612
H	0.738575162	0.051464245	2.676034505
H	-0.034222647	1.414759707	3.703296503
C	3.190294970	0.286380208	3.755942820
H	2.120739906	1.816806342	4.831241575
N	4.141129037	-0.323896750	3.512577834

^{3}TS-4a

(imaginary frequency: -365.07 cm^{-1})

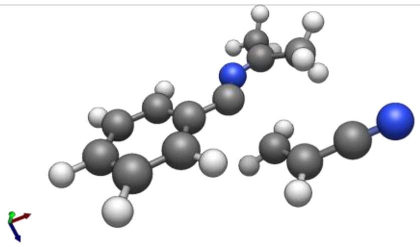

C	2.794495479	0.819806269	-0.551430595
C	1.968932236	-0.076250286	-1.425739236
C	2.633262916	-0.697017002	-2.612687845
N	0.707301956	-0.326040255	-1.211734845
C	-0.045030593	0.064177016	-0.211912917
C	-1.483149287	-0.044992135	-0.141250981
C	-2.197740410	0.492212297	0.948379892
C	-3.585056692	0.399014719	0.998553591
C	-4.287937243	-0.230886343	-0.032134406
C	-3.590465041	-0.765990052	-1.119738587
C	-2.204551079	-0.675614194	-1.179811058
H	3.434806403	0.223312105	0.120874898
H	2.160562347	1.474231231	0.061235669
H	3.467823893	1.430287504	-1.170807427
H	1.940240248	-1.359168057	-3.144038367
H	2.993047905	0.086488108	-3.298968605
H	3.519520622	-1.269879045	-2.296082246
H	-1.652294479	0.993046220	1.750344392
H	-4.123780542	0.825134915	1.846697010
H	-5.375766714	-0.303872670	0.011282216
H	-4.133354896	-1.260600160	-1.927492028
H	-1.652496452	-1.090525930	-2.024254598
C	0.809907306	-0.592326150	1.774628541
C	0.934420890	0.414136225	2.688813732
H	1.684419742	-0.959332260	1.234266596
H	-0.058350155	-1.250248049	1.832401398
C	2.129334821	1.182984129	2.811833810
H	0.109935729	0.694246327	3.348045268
N	3.101961092	1.803665524	2.920726729

^{3}TS-4b

(imaginary frequency: -545.19 cm^{-1})

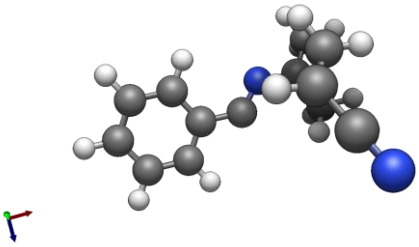

C	2.447594434	-1.140049228	0.672653477
C	1.851782499	-0.605698255	-0.599437157
C	2.590723749	-0.874214868	-1.878682940
N	0.483369198	-0.568233932	-0.717781479
C	-0.357126492	-0.648834221	0.212703618
C	-1.797665804	-0.444118569	0.185825219
C	-2.423277850	0.088836244	-0.957728702
C	-3.799462455	0.289274514	-0.967680072
C	-4.564509940	-0.038000799	0.156637335
C	-3.950094770	-0.566015798	1.293948343
C	-2.571286391	-0.765139054	1.312057635
H	3.530549857	-0.959740134	0.709475913
H	2.275934048	-2.229948757	0.714254541
H	1.966852379	-0.692480950	1.553262310
H	2.580583303	-1.960073433	-2.077729433
H	2.102888269	-0.368851129	-2.722324396
H	3.639969826	-0.556730283	-1.811197242
H	-1.811294146	0.339782358	-1.825995580
H	-4.281214510	0.703732811	-1.854906375
H	-5.644315235	0.120178779	0.144627557
H	-4.548206276	-0.822403051	2.169804802
H	-2.078122075	-1.173377463	2.196310120
C	2.273053456	1.500345101	-0.415138828
C	1.586581160	2.006702213	0.662949765
H	3.344234146	1.302266129	-0.331089399
H	1.879529028	1.691444635	-1.414553347
C	2.133682270	2.000963641	1.975749489
H	0.560815823	2.369470422	0.566615699
N	2.578432494	2.000913078	3.047369126

^{3}IM-4a

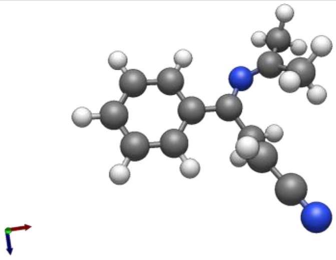

C	2.847224669	0.505551941	-0.048132848
C	2.081611805	-0.653906905	-0.632779181
C	2.755551348	-1.415159501	-1.735565760
N	0.889678134	-0.995077829	-0.280514090
C	0.205255747	-0.578019939	0.813898530
C	-1.237181069	-0.457792610	0.719878916
C	-2.030761342	-0.102510728	1.835987815
C	-3.411897897	0.022078075	1.719605168
C	-4.046152026	-0.205585744	0.496135136
C	-3.275974045	-0.559403848	-0.617579595
C	-1.896928511	-0.681088664	-0.511941371
H	3.449520329	0.189378858	0.819725965
H	2.166357868	1.302701571	0.281426223
H	3.542866658	0.912568793	-0.794058984
H	2.129032137	-2.251023120	-2.067509560
H	2.966742841	-0.748327193	-2.586561938
H	3.726356130	-1.800512921	-1.385049267
H	-5.129375597	-0.107797750	0.409474878
H	-1.299214174	-0.957408586	-1.380702197
H	-4.000532202	0.298235813	2.596440128
H	1.898311269	-0.843555734	2.107915709
H	-3.759601011	-0.740449489	-1.579486816
C	0.887543514	-0.409556214	2.147543574
C	0.963206512	1.009175613	2.636008455
N	2.335256293	1.605259183	4.722789636
H	0.348418372	-1.006211794	2.908715454
C	1.708191685	1.344722309	3.776887085
H	0.420820253	1.810395093	2.129585522
H	-1.569717689	0.081841320	2.807783415

^{3}IM-4b

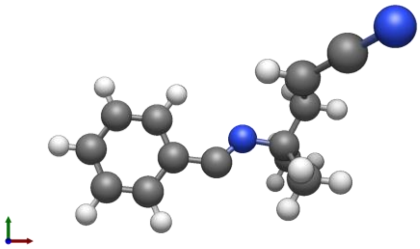

C	3.130384018	-1.158041275	0.548112098
C	2.744612320	-0.210632411	-0.587526467
C	3.150912974	-0.792351533	-1.943051779
N	1.294775295	0.027781717	-0.622454270
C	0.401888431	-0.760774510	-0.304942228
C	-1.056897103	-0.597588758	-0.307431782
C	-1.633612132	0.679943492	-0.408960382
C	-3.018178756	0.818845539	-0.403317199
C	-3.835104126	-0.311559871	-0.301260612
C	-3.266400510	-1.581994659	-0.200804644
C	-1.879499921	-1.725124844	-0.195140412
H	4.223412741	-1.273048902	0.586809470
H	2.672658802	-2.144260530	0.388267105
H	2.781340229	-0.767607890	1.515767778
H	2.677248932	-1.774428849	-2.084021254
H	2.834427250	-0.127356747	-2.758962829
H	4.242542228	-0.914639524	-1.988044697
H	-0.981639851	1.551642843	-0.491616451
H	-3.465531209	1.811135748	-0.483171446
H	-4.920775725	-0.199172136	-0.299297222
H	-3.904684580	-2.463579713	-0.123833425
H	-1.420131510	-2.711493927	-0.105687829
C	3.925145694	2.323994667	1.793263576
C	3.422892847	1.165181419	-0.408553928
C	3.007008455	1.855385297	0.846379253
N	4.698103374	2.712458791	2.574190176
H	3.127264572	1.792364606	-1.268311999
H	4.513852819	1.039661390	-0.442636380
H	1.945114443	2.012480572	1.049327779

MECP-2

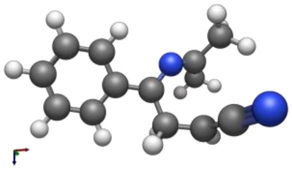

C	2.553340919	-1.273891785	0.277037581
C	1.968064792	-0.346802735	-0.751227947
C	2.853150297	0.064021721	-1.884831312
N	0.732162866	0.025018868	-0.751709418
C	-0.128441797	0.199142960	0.236589562
C	-1.535467249	-0.102492998	0.024627694
C	-2.479102285	0.020141964	1.066981492
C	-3.819509625	-0.285624366	0.847954327
C	-4.250891695	-0.714162480	-0.409094309
C	-3.325101322	-0.842917588	-1.450643613
C	-1.985091183	-0.546514887	-1.239206742
H	1.863040751	-1.454818535	1.109833521
H	3.503217840	-0.872489510	0.659224759
H	2.782541001	-2.234315026	-0.214331387
H	2.269856778	0.535280881	-2.684060990
H	3.585825138	0.795877698	-1.504958611
H	3.418821709	-0.794302067	-2.276237344
H	-2.165648795	0.344783613	2.059663949
H	-4.534907403	-0.186481884	1.666203777
H	-5.303394426	-0.946745285	-0.579038195
H	-3.656498187	-1.177467736	-2.435273507
H	-1.261514511	-0.643395075	-2.049669423
C	0.313018808	0.838186149	1.554523606
C	1.713329108	1.351195236	1.586073678
H	-0.410988285	1.647585997	1.761225361
H	0.181295204	0.104661140	2.368202107
C	2.155415517	2.357861638	0.722711670
H	2.429302183	0.962537346	2.310155136
N	2.534173849	3.176126747	-0.020725423

[1]Product

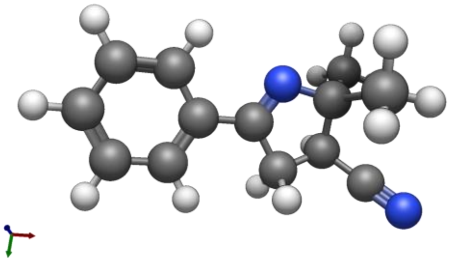

C	3.793125793	-1.631648505	0.350213051
C	2.890854080	-0.957223194	-0.675967262
C	3.118304815	-1.545336317	-2.070700193
N	1.480588076	-1.159765650	-0.326389100
C	0.818008874	-0.079954973	-0.462187531
C	-0.647119702	-0.011857728	-0.254560846
C	-1.349905110	1.172839063	-0.506031565
C	-2.733226357	1.223275038	-0.327925162
C	-3.422366117	0.091855662	0.106575190
C	-2.725848472	-1.093298677	0.362915752
C	-1.347553115	-1.146271236	0.183320387
H	4.848349634	-1.411017287	0.130495311
H	3.649747355	-2.720971589	0.320851964
H	3.560168091	-1.272430215	1.362958238
H	2.797261207	-2.595871350	-2.077517570
H	2.539534730	-0.996313402	-2.828992472
H	4.182784578	-1.499523529	-2.341178793
H	-0.819464314	2.063642197	-0.847329961
H	-3.272173065	2.149799824	-0.532022895
H	-4.504299082	0.130989457	0.244458346
H	-3.264528436	-1.979985478	0.701578324
H	-0.791409448	-2.064152002	0.377950137
C	1.631296881	1.122992121	-0.902512118
C	3.054665442	0.601688538	-0.651011773
H	1.437936455	1.337626832	-1.965771782
N	4.843766888	1.504206148	-2.331252640
C	4.057108918	1.111843533	-1.582977493
H	1.405516969	2.030200796	-0.327747990
H	3.370154432	0.897031923	0.361100445

[1]Acrylonitrile

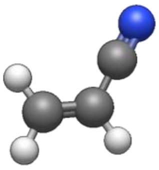

C	5.0241920212	-0.1941224712	0.0000361303
C	-3.6887315703	-0.1551011120	-0.0001647813
H	-5.5462062559	-1.1524852730	-0.0000166543
H	-5.6231164251	0.7190893104	0.0003680575
N	-2.3767591448	2.0835796382	-0.0000210370
C	2.9660601427	1.0896939260	-0.0000761710
H	3.0802044397	-1.0627240184	-0.0001955440

[1]CO2

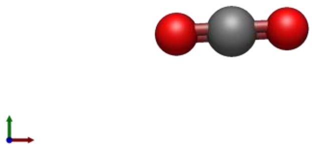

O	0.944682389	0.237078118	-0.000009365
C	2.099530188	0.293801060	-0.000011605
O	3.254367423	0.350730821	-0.000019030

5.2. Dynamic Vertical Triplet Energies

Dynamic vertical triplet energies were calculated using an adapted method originally developed by Paton[42] and colleagues.[42] Starting from structures obtained with the workflow described in 5.1, another geometry optimization and high precision frequency calculation (at M06-2X/6-31G(d) level of theory) was carried out using Gaussian 16[43] on the Palma II high performance computing cluster of the University of Münster. Based on this result, 25 MD trajectories were generated using the *milo* package developed by ESS and colleagues[44] interfering with Gaussian 16, also on the Palma II cluster. The milo MD simulations were run for 1000 fs at the M06-2X/MIDI! level of theory with a step size of 1 fs at a temperature of 298.15 K. Snapshots of the geometry were extracted every 5 fs from every of the 25 simulations, yielding 5000 geometries in total.

For each of the geometries, ORCA single point calculation job scripts (at M06-2X/6-31G(d)) level of theory, D3 dispersion correction, CPCM model for MeCN were automatically generated for the singlet and triplet state and executed in parallel on the local computing cluster of the GLORIUS group on Intel© Xeon® Gold 6240 CPUs.

Vertical triplet energies were extracted as the difference in electronic energies for the singlet and triplet state of every snapshot. The collection of vertical gaps was subsequently plotted in a histogram using *matplotlib*[45] and approximated with a normal distribution with *scikit-learn*.[46] The corresponding cumulative distribution function (CDF) was calculated, and dynamic vertical triplet energies were determined as the energy including 0.8% of the distribution.[42]

5.2.1. Optimized Geometry for MD Simulation

Starting geometry of ^{1}Azirine

Optimized using Gaussian16 at M06-2X/6-31G(d) level of theory

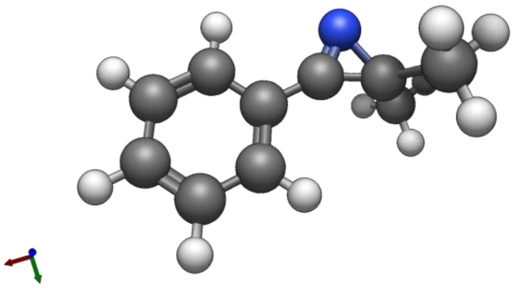

C	-2.894658	0.442848	-1.276242
C	-2.215790	-0.006774	0.000008
C	-2.894563	0.442697	1.276362
N	-1.634325	-1.414567	-0.000089
C	-0.831359	-0.442633	-0.000071
C	0.596869	-0.170352	-0.000043
C	1.057579	1.147670	-0.000021
C	2.424993	1.402331	0.000016
C	3.328273	0.341618	0.000032
C	2.868559	-0.976209	0.000011
C	1.504537	-1.235120	-0.000027
H	-3.927477	0.077130	-1.306282
H	-2.368981	0.060827	-2.154712
H	-2.926592	1.536504	-1.338954
H	-2.368689	0.060779	2.154758
H	-2.926709	1.536346	1.339083
H	-3.927304	0.076769	1.306543
H	0.337319	1.960950	-0.000033
H	2.786848	2.425500	0.000033
H	4.395455	0.541074	0.000063
H	3.577026	-1.798461	0.000024
H	1.122742	-2.251899	-0.000043

5.2.2. Vertical Triplet Energy Distribution and Dynamic Vertical Triplet Energy

Vertical triplet energy distribution of azirine **4a**.

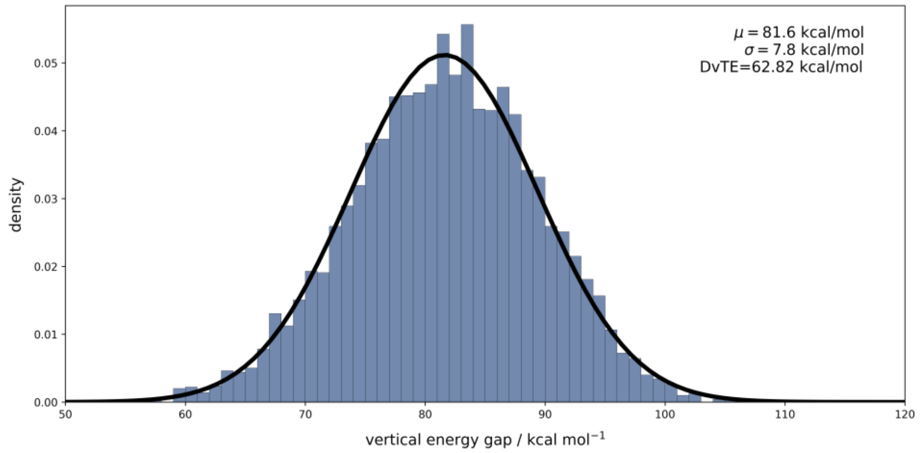

Figure S14: Histogram of vertical triplet energies obtained from different geometries in a molecular dynamics simulation. Distriubtion was approximated using a normal distribution and DvTE was determined to include 0.8% of the distribution.

Cumulative distribution function of the vertical triplet energy distribution

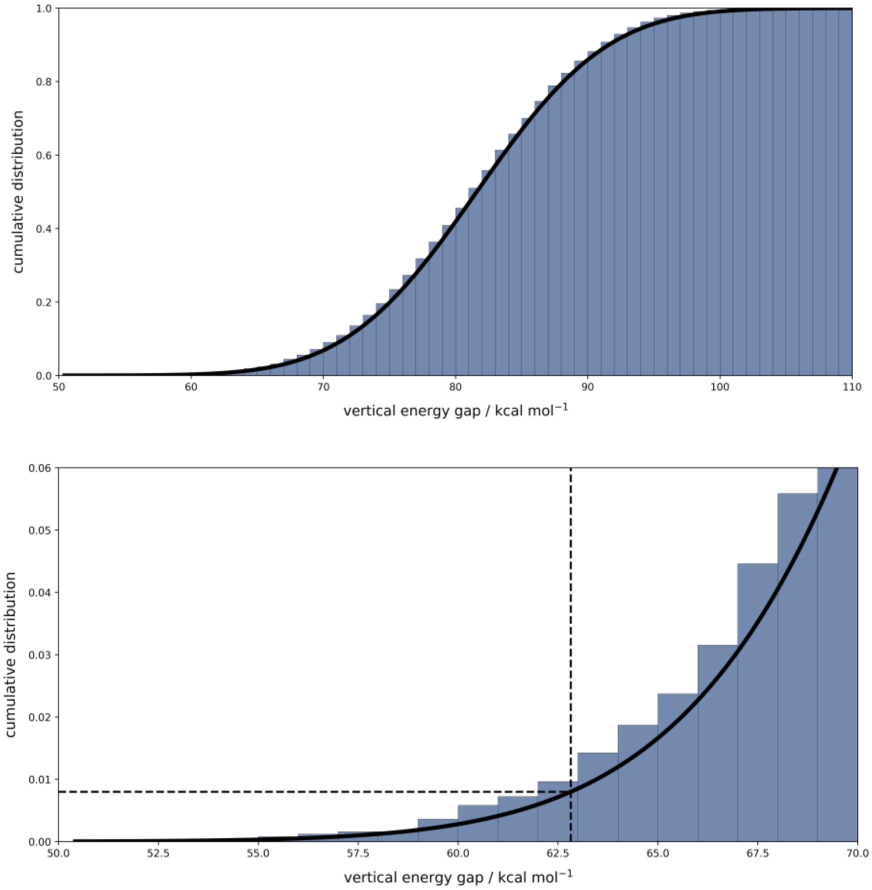

Figure S15: Cumulative distribution function (CDF) of the approximated normal distribution and determined DvTE at 0.8% of the distribution included.

5.2.3. Discussion on Energy Transfer between Ir-F and 4a

As mentioned in the main manuscript, direct excitation and photoredox pathways for the reactivity of azirine **4a** were ruled out by experiments. The adiabatic triplet energy was, however, calculated at 68.6 kcal/mol. However, a major change in geometry, alas nuclear reorganization, was observed. As discussed by Paton and coworkers[47], the adiabatic approximation often fails to make reliable predictions for triplet energy in these cases. Therefore, we investigated the dynamic vertical triplet energy of **4a** according to their protocol, which resulted in a value of 62.8 kcal/mol, which is very close to reported triplet energies of **Ir-F** (61.8 kcal/mol).[26] This slightly endergonic energy transfer can nevertheless proceed, as the vertical triplet energy distribution possesses values down to 55 kcal/mol. Furthermore, the endergonic character of this process explains the lower observed quenching rate of **4a** in comparison to **1a** and the resulting accumulation of **4a** during the reaction (ref. **Figure** 3 in the main manuscript).

6. ACKNOWLEDGEMENTS

All calculations in Gaussian16 that were made for this publication were performed on the HPC cluster PALMA II of the University of Münster, subsidized by the DFG (INST 211/667-1).

7. CRYSTAL STRUCTURES

X-Ray diffraction: Data sets for compounds **3a**, **3q**, **3aa**, **6c** and **S6** were collected with a Bruker D8 Venture Photon III Diffractometer. Programs used: data collection: *APEX4* Version 2021.4-0[48] (Bruker AXS Inc., **2021**); cell refinement: *SAINT* Version 8.40B (Bruker AXS Inc., **2021**); data reduction: *SAINT* Version 8.40B (Bruker AXS Inc., **2021**); absorption correction, *SADABS* Version 2016/2 (Bruker AXS Inc., **2021**); structure solution *SHELXT*-Version 2018-3[49] (Sheldrick, G. M. *Acta Cryst.,* **2015**, *A71*, 3-8); structure refinement *SHELXL*-Version 2018-3[50] (Sheldrick, G. M. *Acta Cryst.,* **2015**, *C71* (1), 3-8) and graphics, *XP*[51] (Version 5.1, Bruker AXS Inc., Madison, Wisconsin, USA, **1998**). *R*-values are given for observed reflections, and wR^2 values are given for all reflections.

X-ray crystal structure analysis of 3a (glo10598) (*see procedure***):** A colorless, needle-like specimen of $C_{13}H_{14}N_2$, approximate dimensions 0.044 mm x 0.057 mm x 0.205 mm, was used for the X-ray crystallographic analysis. The X-ray intensity data were measured on a single crystal diffractometer Bruker D8 Venture Photon III system equipped with a micro focus tube Cu ImS (CuKα, λ = 1.54178 Å) and a MX mirror monochromator. A total of 1846 frames were collected. The total exposure time was 22.95 hours. The frames were integrated with the Bruker SAINT software package using a wide-frame algorithm. The integration of the data using a monoclinic unit cell yielded a total of 16787 reflections to a maximum θ angle of 66.82° (0.84 Å resolution), of which 1987 were independent (average redundancy 8.448, completeness = 99.3%, R_{int} = 4.65%, R_{sig} = 2.42%) and 1728 (86.97%) were greater than 2σ(F^2). The final cell constants of a = 8.7772(2) Å, b = 14.0163(3) Å, c = 9.8278(2) Å, β = 111.2080(10)°, volume = 1127.17(4) Å^3, are based upon the refinement of the XYZ-centroids of 7635 reflections above 20 σ(I) with 11.53° < 2θ < 132.9°. Data were corrected for absorption effects using the multi-scan method (SADABS). The ratio of minimum to maximum

apparent transmission was 0.893. The calculated minimum and maximum transmission coefficients (based on crystal size) are 0.8970 and 0.9770. The structure was solved and refined using the Bruker SHELXTL Software Package, using the space group $P2_1/c$, with Z = 4 for the formula unit, $C_{13}H_{14}N_2$. The final anisotropic full-matrix least-squares refinement on F^2 with 138 variables converged at R1 = 3.48%, for the observed data and wR2 = 8.46% for all data. The goodness-of-fit was 1.031. The largest peak in the final difference electron density synthesis was 0.238 $e^-/Å^3$ and the largest hole was -0.175 $e^-/Å^3$ with an RMS deviation of 0.032 $e^-/Å^3$. On the basis of the final model, the calculated density was 1.168 g/cm^3 and F(000), 424 e^-. CCDC Nr.: 2407092.

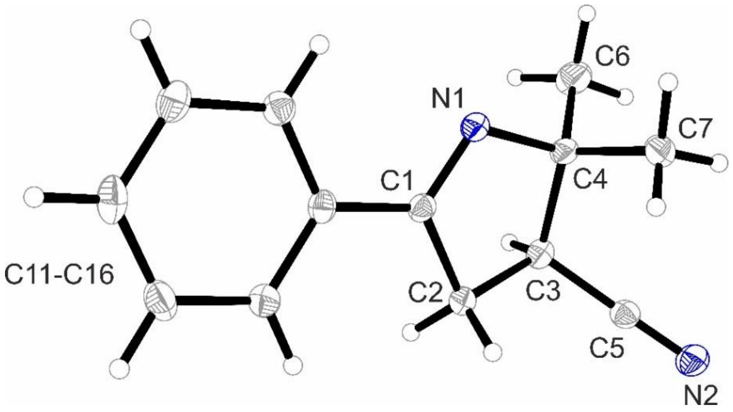

Figure S16: Crystal structure of compound **3a**.
Thermal ellipsoids are shown at 50% probability.

X-ray crystal structure analysis of 3q (glo10597) (see procedure)**:** A colorless, prism-like specimen of $C_{16}H_{18}F_3NO_2$, approximate dimensions 0.096 mm x 0.103 mm x 0.111 mm, was used for the X-ray crystallographic analysis. The X-ray intensity data were measured on a single crystal diffractometer Bruker D8 Venture Photon III system equipped with a micro focus tube Cu ImS (CuKα, λ = 1.54178 Å) and a MX mirror monochromator. A total of 1949 frames were collected. The total exposure time was 22.10 hours. The frames were integrated with the Bruker SAINT software package using a wide-frame algorithm. The integration of the data using a monoclinic unit cell yielded a total of 29082 reflections to a maximum θ angle of 67.12° (0.84 Å resolution), of which 2681 were independent (average redundancy 10.847, completeness = 98.9%, R_{int} = 3.89%, R_{sig} = 1.93%) and 2458 (91.68%) were greater than 2σ(F^2). The final cell constants of a = 9.9476(3) Å, b = 8.3413(2) Å, c = 18.4562(5) Å, β = 96.4070(10)°, volume = 1521.86(7) $Å^3$, are based upon the refinement of the XYZ-centroids of 9877 reflections above 20 σ(I) with 8.945° < 2θ < 134.1°. Data were corrected for absorption effects using the multi-scan method (SADABS). The ratio of minimum to maximum apparent transmission was 0.897. The calculated minimum and maximum transmission coefficients (based on crystal size) are 0.8990 and 0.9120. The structure was solved and refined using the Bruker SHELXTL Software Package, using the space group $P2_1/c$, with Z = 4 for the formula unit, $C_{16}H_{18}F_3NO_2$. The final anisotropic full-matrix least-squares refinement on F^2 with 202 variables converged at R1 = 3.06%, for the observed data and wR2 = 7.71% for all data. The goodness-of-fit was 1.041. The largest peak in the final difference electron density synthesis was 0.274 $e^-/Å^3$ and the largest hole was -0.187 $e^-/Å^3$ with an RMS deviation of 0.035 $e^-/Å^3$. On the basis of the final model, the calculated density was 1.367 g/cm^3 and F(000), 656 e^-. CCDC Nr.: 2407093.

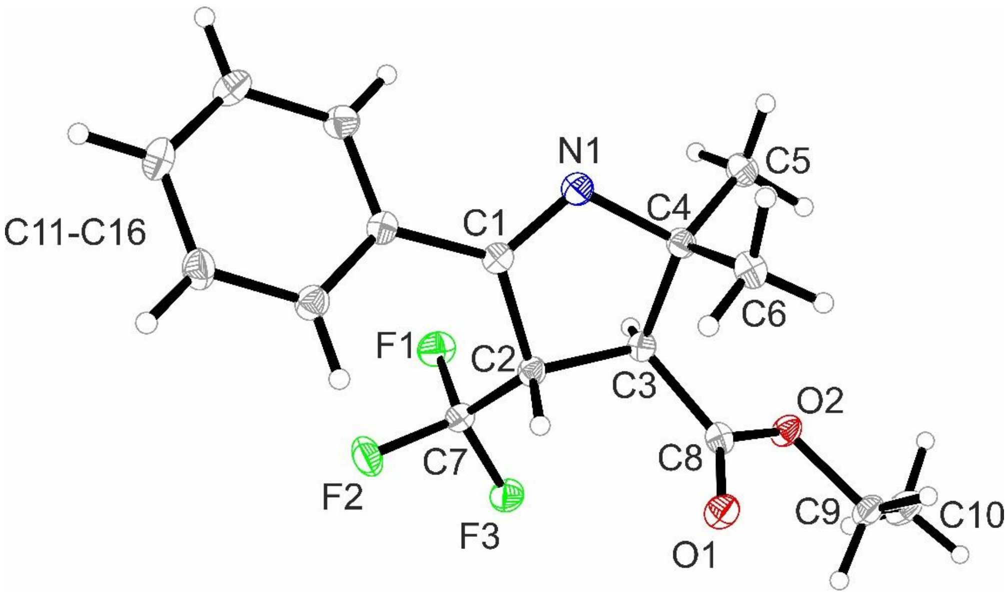

Figure S17: Crystal structure of compound **3q**.
Thermal ellipsoids are shown at 50% probability.

X-ray crystal structure analysis of 3aa (glo10679) (see procedure)**:** A colorless, prism-like specimen of $C_{17}H_{15}FN_2O$, approximate dimensions 0.070 mm x 0.076 mm x 0.092 mm, was used for the X-ray crystallographic analysis. The X-ray intensity data were measured on a single crystal diffractometer Bruker D8 Venture Photon III system equipped with a micro focus tube Cu ImS (CuKα, λ = 1.54178 Å) and a MX mirror monochromator. A total of 1855 frames were collected. The total exposure time was 21.82 hours. The frames were integrated with the Bruker SAINT software package using a wide-frame algorithm. The integration of the data using a monoclinic unit cell yielded a total of 22404 reflections to a maximum θ angle of 68.36° (0.83 Å resolution), of which 2580 were independent (average redundancy 8.684, completeness = 99.5%, R_{int} = 5.83%, R_{sig} = 3.02%) and 2229 (86.40%) were greater than 2σ(F^2). The final cell constants of a = 9.87380(10) Å, b = 8.99780(10) Å, c = 15.9311(2) Å, β = 91.3260(10)°, volume = 1414.98(3) Å^3, are based upon the refinement of the XYZ-centroids of 9976 reflections above 20 σ(I) with 11.11° < 2θ < 136.6°. Data were corrected for absorption effects using the multi-scan method (SADABS). The ratio of minimum to maximum apparent transmission was 0.797. The calculated minimum and maximum transmission coefficients (based on crystal size) are 0.9330 and 0.9490. The structure was solved and refined using the Bruker SHELXTL Software Package, using the space group $P2_1/n$, with Z = 4 for the formula unit, $C_{17}H_{15}FN_2O$. The final anisotropic full-matrix least-squares refinement on F^2 with 192 variables converged at R1 = 3.87%, for the observed data and wR2 = 9.96% for all data. The goodness-of-fit was 1.067. The largest peak in the final difference electron density synthesis was 0.255 e^-/Å^3 and the largest hole was -0.213 e^-/Å^3 with an RMS deviation of 0.045 e^-/Å^3. On the basis of the final model, the calculated density was 1.325 g/cm^3 and F(000), 592 e^-. CCDC Nr.: 2407094.

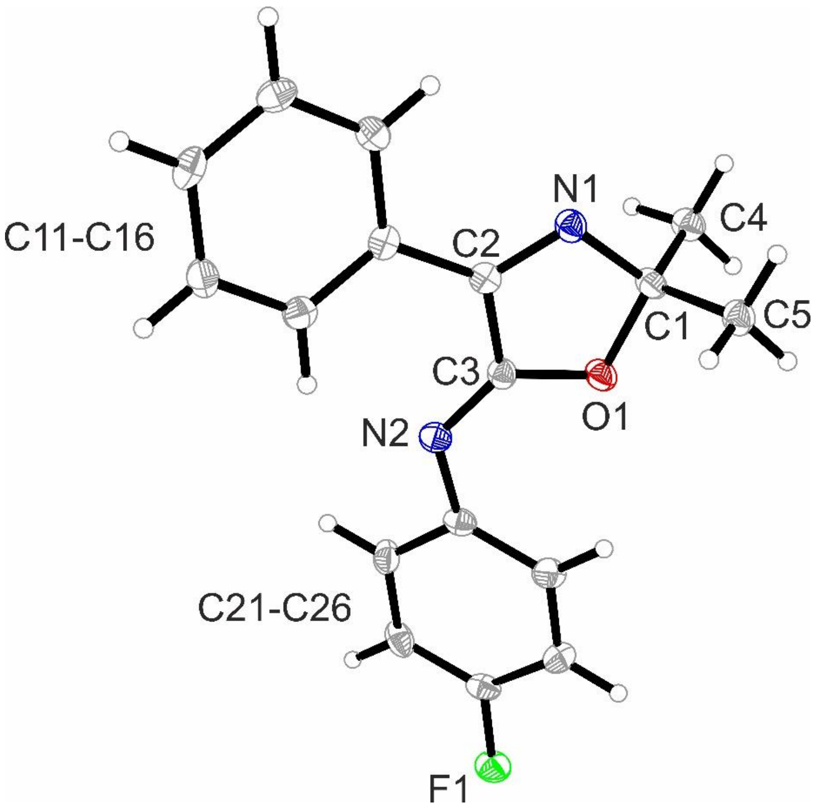

Figure S18: Crystal structure of compound **3aa**.

Thermal ellipsoids are shown at 50% probability.

X-ray crystal structure analysis of 6c (glo10637) (see procedure)**:** A colorless, prism-like specimen of $C_{16}H_{18}F_4N_2O_2$, approximate dimensions 0.079 mm x 0.080 mm x 0.095 mm, was used for the X-ray crystallographic analysis. The X-ray intensity data were measured on a single crystal diffractometer Bruker D8 Venture Photon III system equipped with a micro focus tube Cu ImS (CuKα, λ = 1.54178 Å) and a MX mirror monochromator. A total of 1730 frames were collected. The total exposure time was 22.39 hours. The frames were integrated with the Bruker SAINT software package using a wide-frame algorithm. The integration of the data using a triclinic unit cell yielded a total of 12714 reflections to a maximum θ angle of 68.19° (0.83 Å resolution), of which 2980 were independent (average redundancy 4.266, completeness = 99.6%, R_{int} = 4.59%, R_{sig} = 3.48%) and 2428 (81.48%) were greater than $2\sigma(F^2)$. The final cell constants of a = 8.02770(10) Å, b = 9.6539(2) Å, c = 11.6178(2) Å, α = 105.050(1)°, β = 94.9970(10)°, γ = 107.6980(10)°, volume = 814.73(2) Å^3, are based upon the refinement of the XYZ-centroids of 6179 reflections above 20 σ(I) with 8.012° < 2θ < 136.2°. Data were corrected for absorption effects using the multi-scan method (SADABS). The ratio of minimum to maximum apparent transmission was 0.885. The calculated minimum and maximum transmission coefficients (based on crystal size) are 0.9040 and 0.9200. The structure was solved and refined using the Bruker SHELXTL Software Package, using the space group *P*-1, with Z = 2 for the formula unit, $C_{16}H_{18}F_4N_2O_2$. The final anisotropic full-matrix least-squares refinement on F^2 with 221 variables converged at R1 = 3.55%, for the observed data and wR2 = 8.79% for all data. The goodness-of-fit was 1.030. The largest peak in the final difference electron density synthesis was 0.252 $e^-/Å^3$ and the largest hole was -0.206 $e^-/Å^3$ with an RMS deviation of 0.042 $e^-/Å^3$. On the basis of the final model, the calculated density

was 1.412 g/cm^3 and F(000), 360 e$^-$. CCDC Nr.: 2407095.

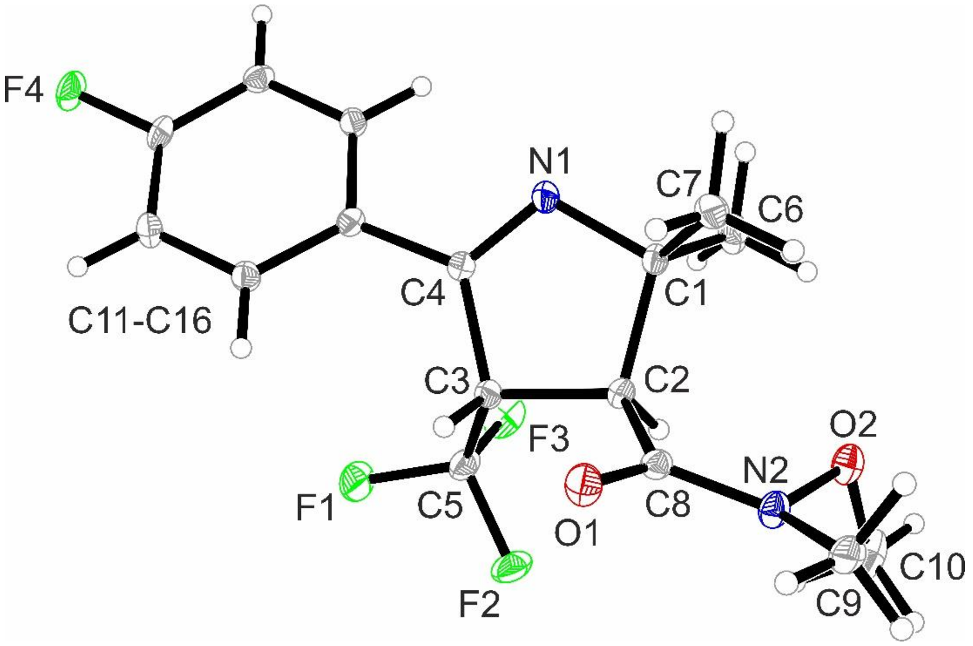

Figure S19: Crystal structure of compound **6c**.

Thermal ellipsoids are shown at 50% probability.

X-ray crystal structure analysis of S6 (glo10642) (<u>see procedure</u>): A colorless, prism-like specimen of $C_{11}H_{11}NO_2$, approximate dimensions 0.082 mm x 0.085 mm x 0.157 mm, was used for the X-ray crystallographic analysis. The X-ray intensity data were measured on a single crystal diffractometer Bruker D8 Venture Photon III system equipped with a micro focus tube Cu ImS (CuKα, λ = 1.54178 Å) and a MX mirror monochromator. A total of 1086 frames were collected. The total exposure time was 8.34 hours. The frames were integrated with the Bruker SAINT software package using a wide-frame algorithm. The integration of the data using a monoclinic unit cell yielded a total of 7189 reflections to a maximum θ angle of 66.65° (0.84 Å resolution), of which 867 were independent (average redundancy 8.292, completeness = 96.7%, R_{int} = 3.58%, R_{sig} = 2.26%) and 796 (91.81%) were greater than 2σ(F^2). The final cell constants of <u>a</u> = 8.1662(3) Å, <u>b</u> = 7.0095(3) Å, <u>c</u> = 8.4592(3) Å, β = 105.2330(10)°, volume = 467.20(3) Å^3, are based upon the refinement of the XYZ-centroids of 5070 reflections above 20 σ(I) with 10.83° < 2θ < 133.3°. Data were corrected for absorption effects using the multi-scan method (SADABS). The ratio of minimum to maximum apparent transmission was 0.921. The calculated minimum and maximum transmission coefficients (based on crystal size) are 0.8900 and 0.9400. The structure was solved and refined using the Bruker SHELXTL Software Package, using the space group $P2_1/m$, with Z = 2 for the formula unit, $C_{11}H_{11}NO_2$. The final anisotropic full-matrix least-squares refinement on F^2 with 83 variables converged at R1 = 3.23%, for the observed data and wR2 = 8.34% for all data. The goodness-of-fit was 1.102. The largest peak in the final difference electron density synthesis was 0.182 e$^-$/Å^3 and the largest hole was -0.193 e$^-$/Å^3 with an RMS deviation of 0.034 e$^-$/Å^3. On the basis of the final model, the calculated density was 1.345 g/cm^3 and F(000), 200 e$^-$. CCDC Nr.: 2407096.

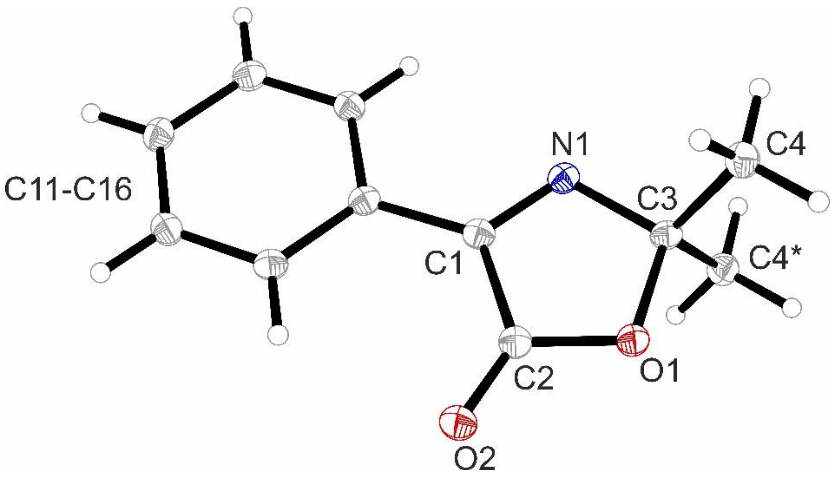

Figure S20: Crystal structure of compound **S6**.
Thermal ellipsoids are shown at 50% probability.

X-ray crystal structure analysis of 7e (glo10746) (see procedure)**:** A colorless, prism-shaped crystal was mounted on the goniometer. Data for glo10746 were collected from a single crystal in 14.75 hours at 100(2) K on a Bruker D8 VENTURE KAPPA diffractometer with a microfocus sealed tube using a multilayer mirror as monochromator and a Bruker PHOTON III CPAD detector. The diffractometer was equipped with an Oxford Cryostream 700 low temperature device and used Cu K_α radiation (λ = 1.54178 Å). All data were integrated with SAINT V8.41, yielding 45047 reflections of which 4055 where independent and 87.2% were greater than $2\sigma(F^2)$.[48] A Multi-Scan absorption correction using SADABS 2016/2 was applied.[1] The structure was solved by Intrinsic Phasing methods with SHELXT 2018/2 and refined by full-matrix least-squares methods against F^2 using SHELXL-2019/2.[49,50] All non-hydrogen atoms were refined with anisotropic displacement parameters. All hydrogen atoms were refined isotropic on calculated positions using a riding model with their U_{iso} values constrained to 1.5 times the U_{eq} of their pivot atoms for terminal sp^3 carbon atoms and 1.2 times for all other carbon atoms. Crystallographic data for the structures reported in this paper have been deposited with the Cambridge Crystallographic Data Centre.[51] CCDC Nr.: 2408024 contain the supplementary crystallographic data for this paper. These data can be obtained free of charge from The Cambridge Crystallographic Data Centre via www.ccdc.cam.ac.uk/structures. This report and the CIF file were generated using FinalCif,

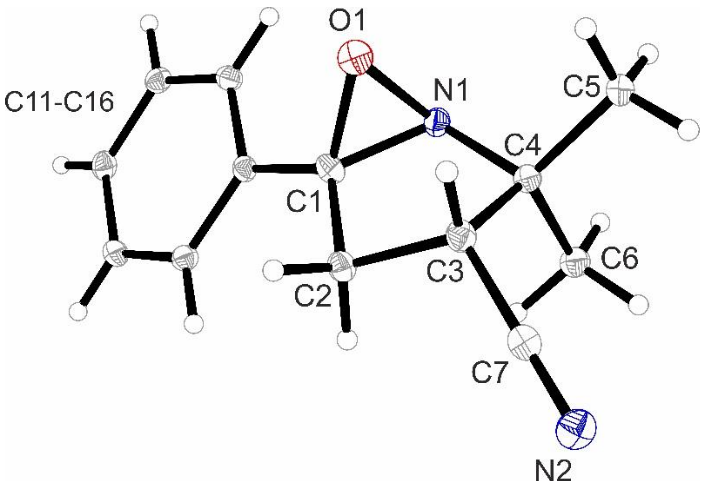

Figure S22: Crystal structure of compound **7e**.
Thermal ellipsoids are shown at 50% probability.

8. SPECTROSCOPIC DATA

^{1}H NMR (400 MHz, $CDCl_3$) of **1a** (*see procedure*)

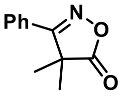

1a

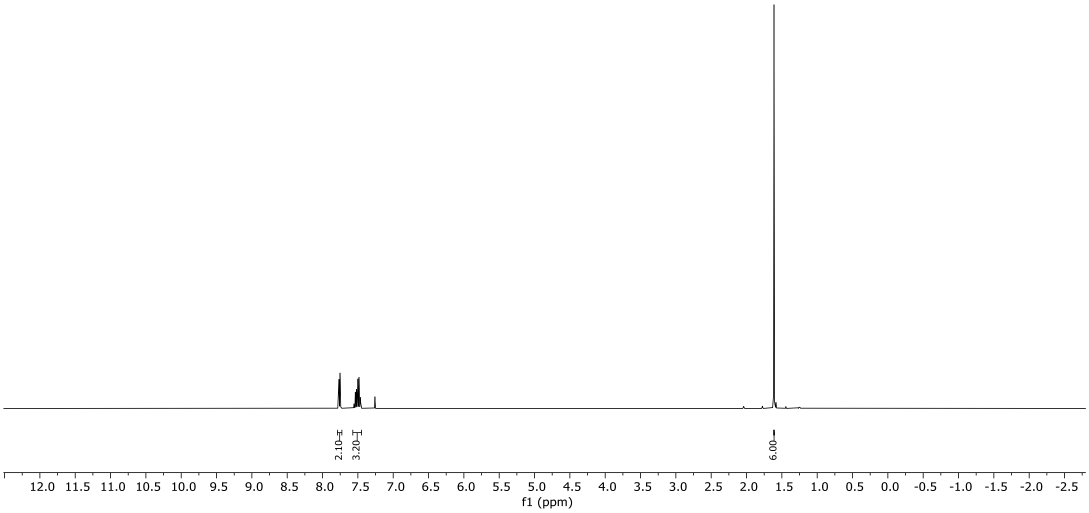

^{13}C NMR (101 MHz, $CDCl_3$) of **1a**

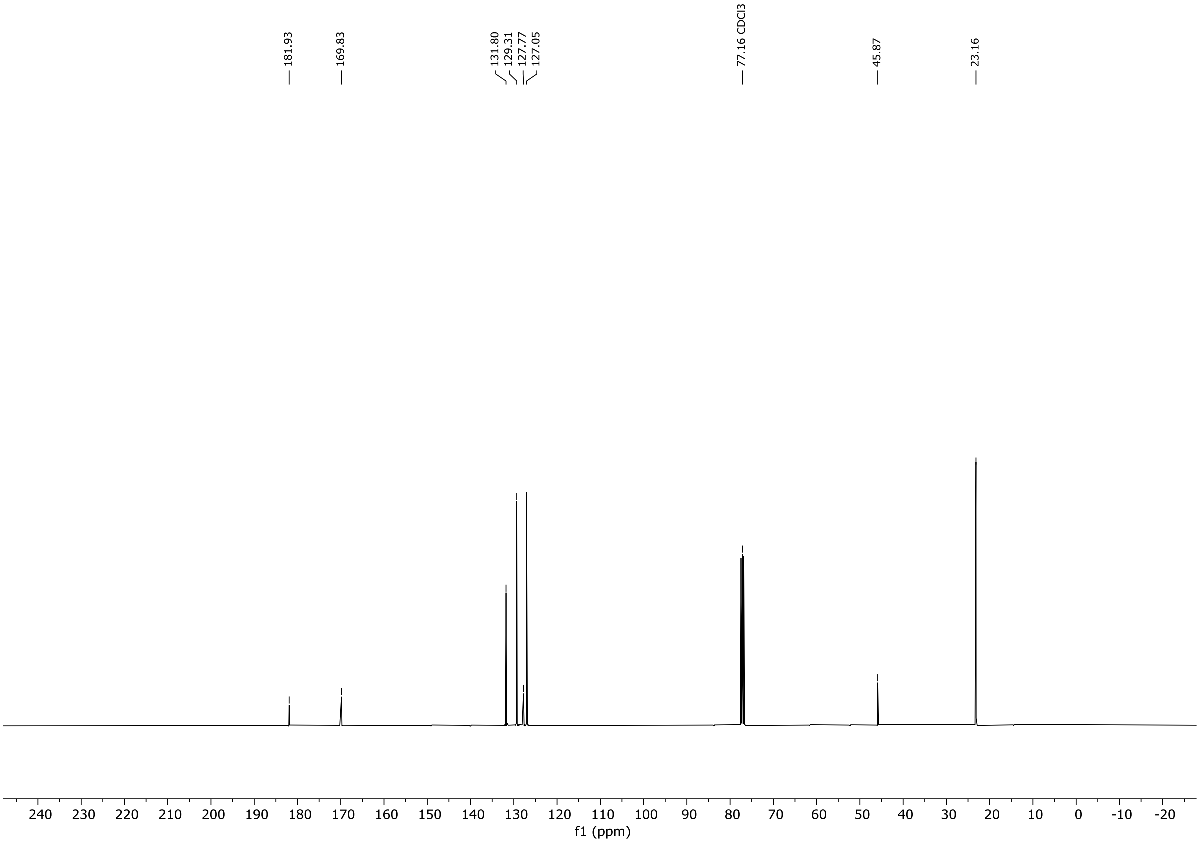

^{1}H NMR (400 MHz, $CDCl_3$) of **1b** (*see procedure*)

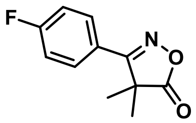

1b

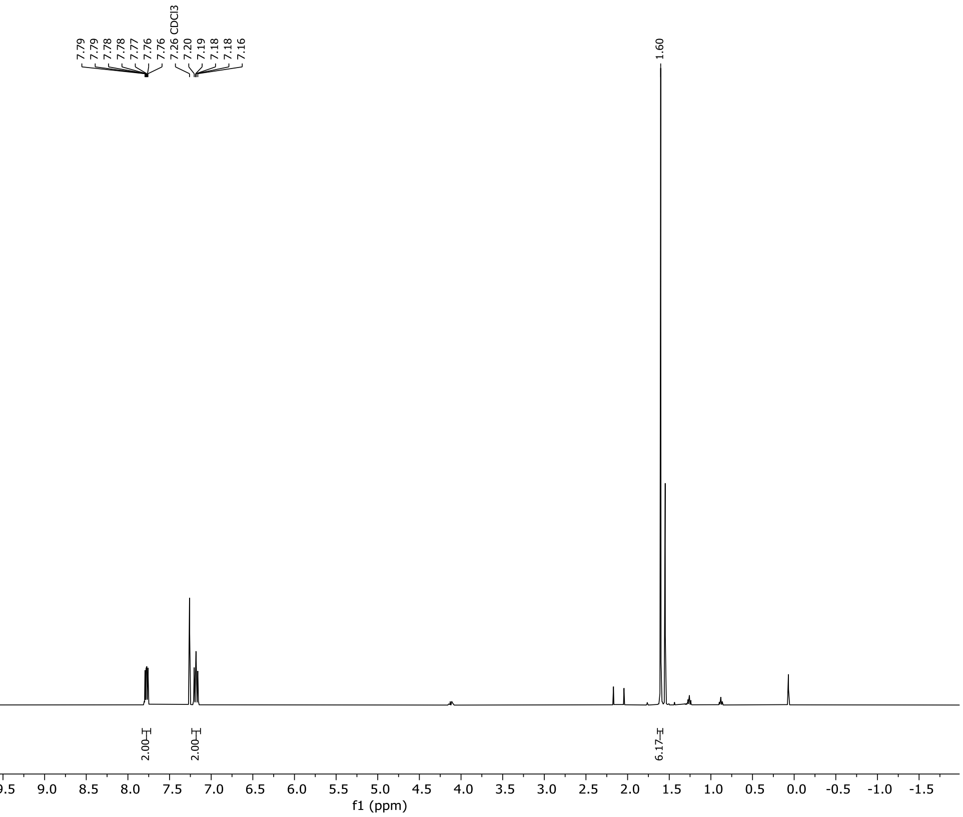

^{19}F NMR (377 MHz, $CDCl_3$) of **1b**

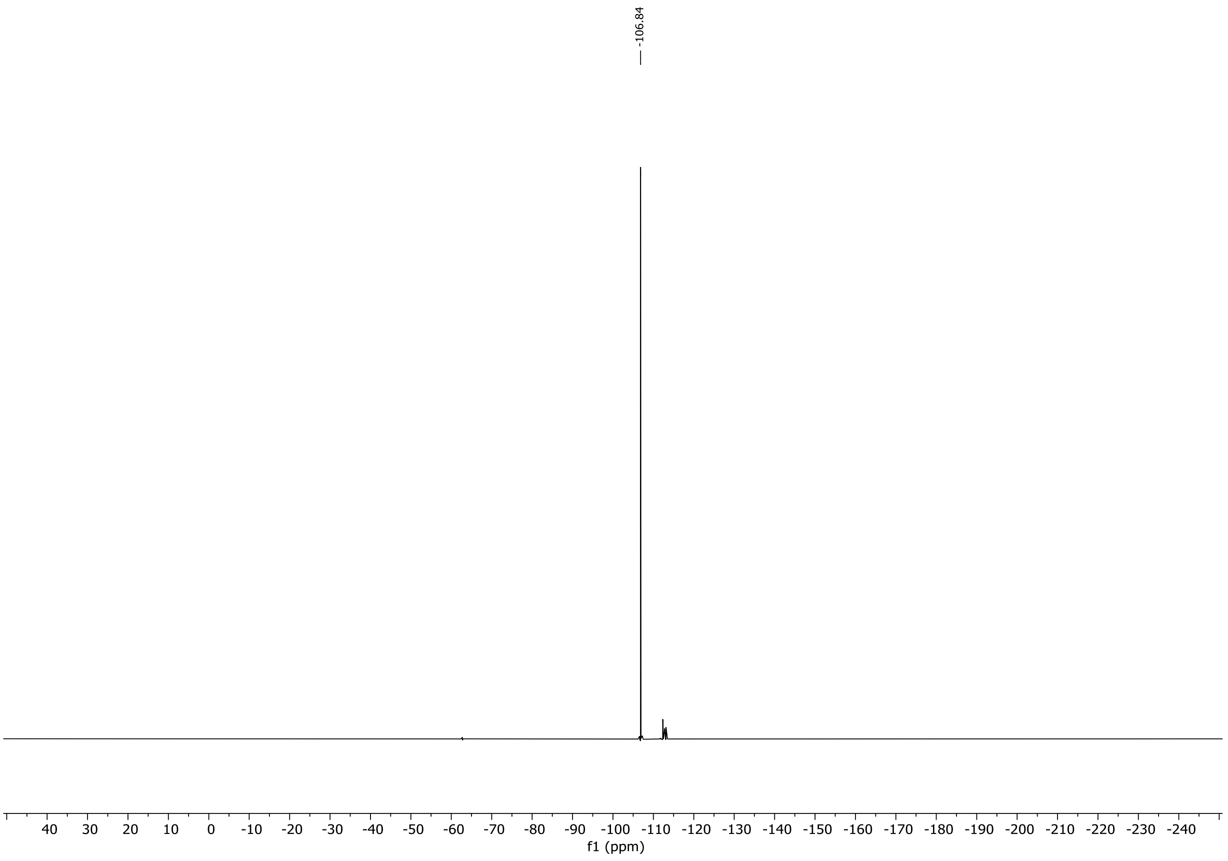

^{13}C NMR (126 MHz, $CDCl_3$) of **1b**

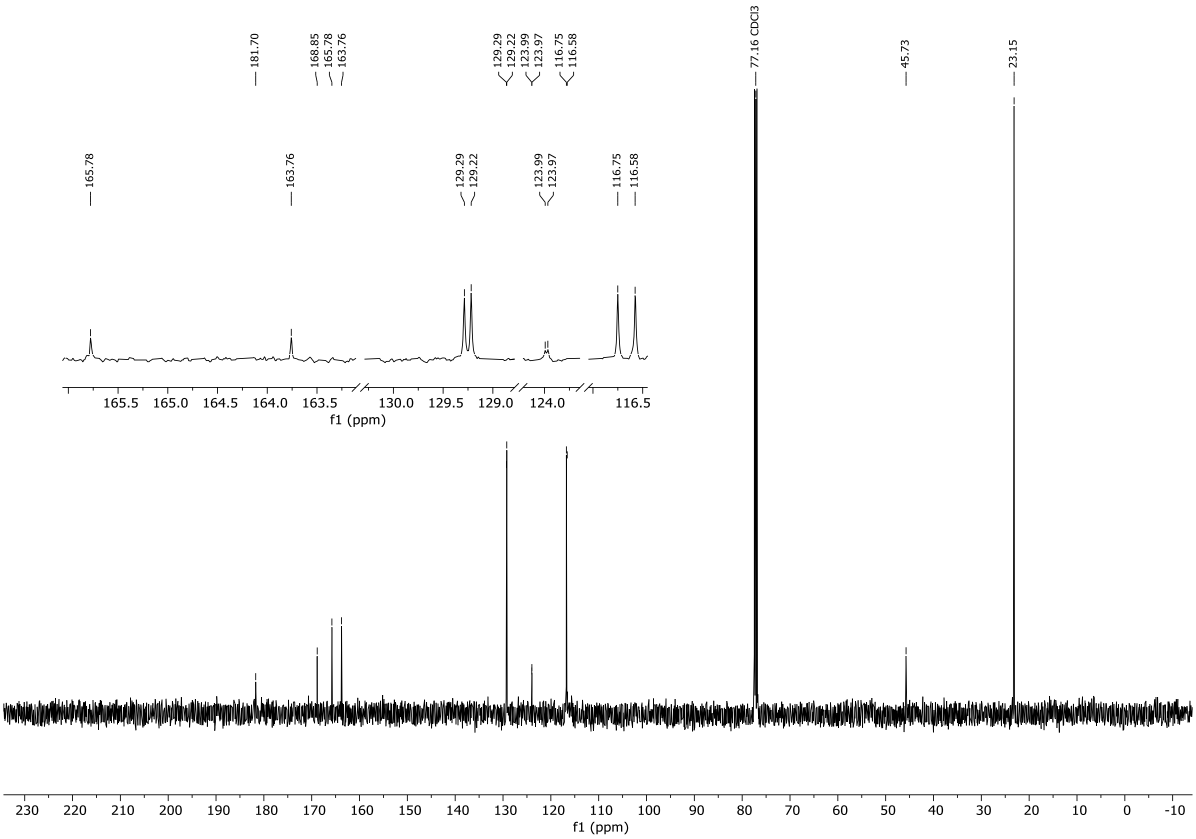

^{13}C NMR {^{1}H, ^{19}F} (126 MHz, $CDCl_3$) of **1b**

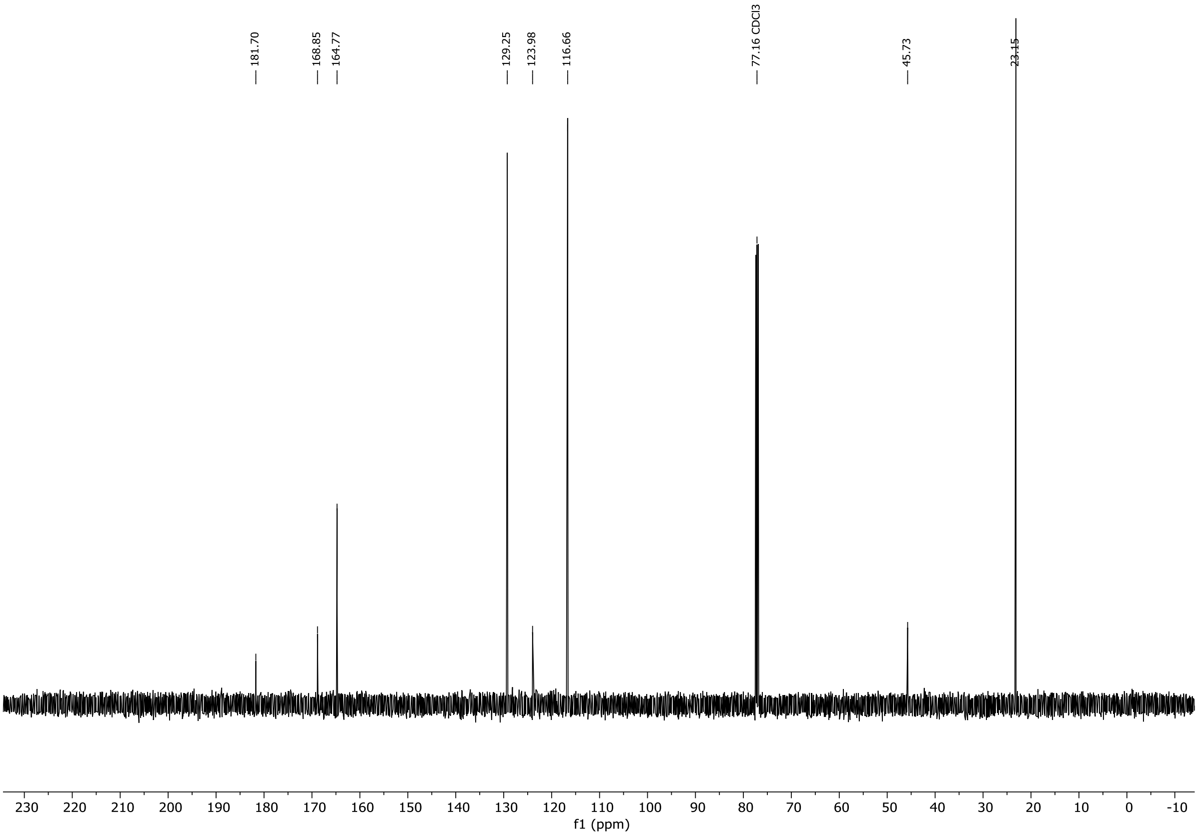

^{1}H NMR (400 MHz, $CDCl_3$) of **1c** (*see procedure*)

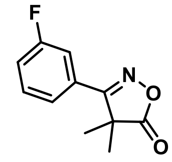

1c

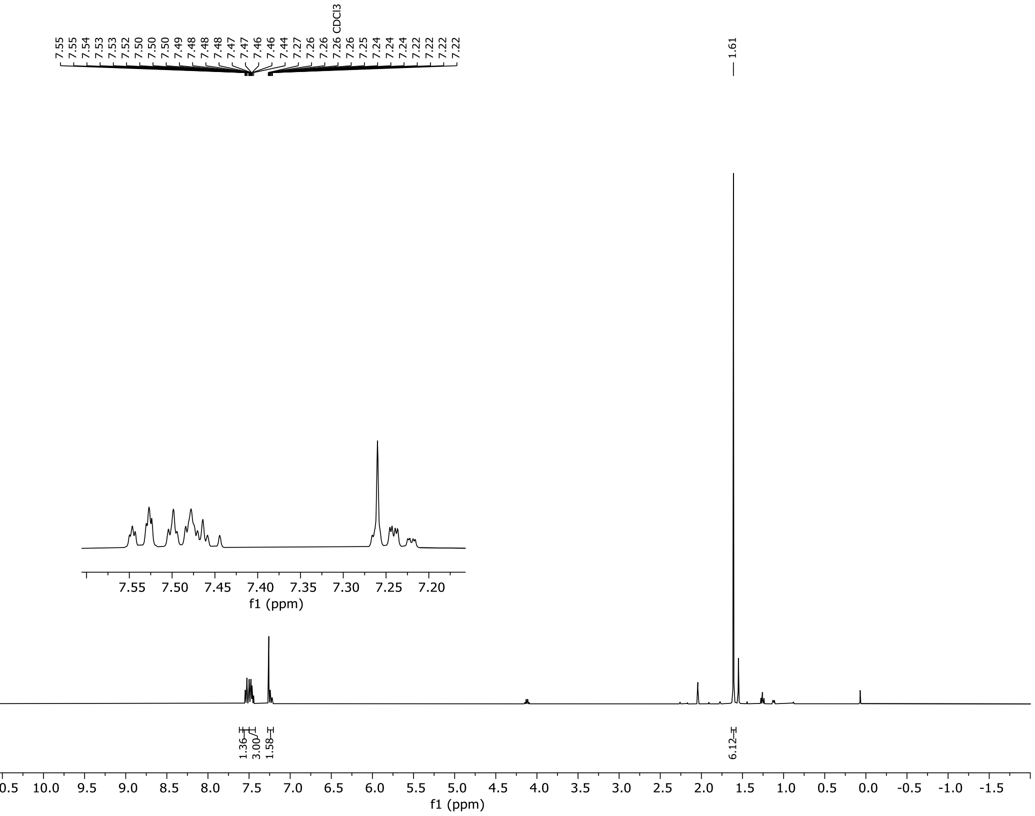

^{19}F NMR (377 MHz, $CDCl_3$) of **1c**

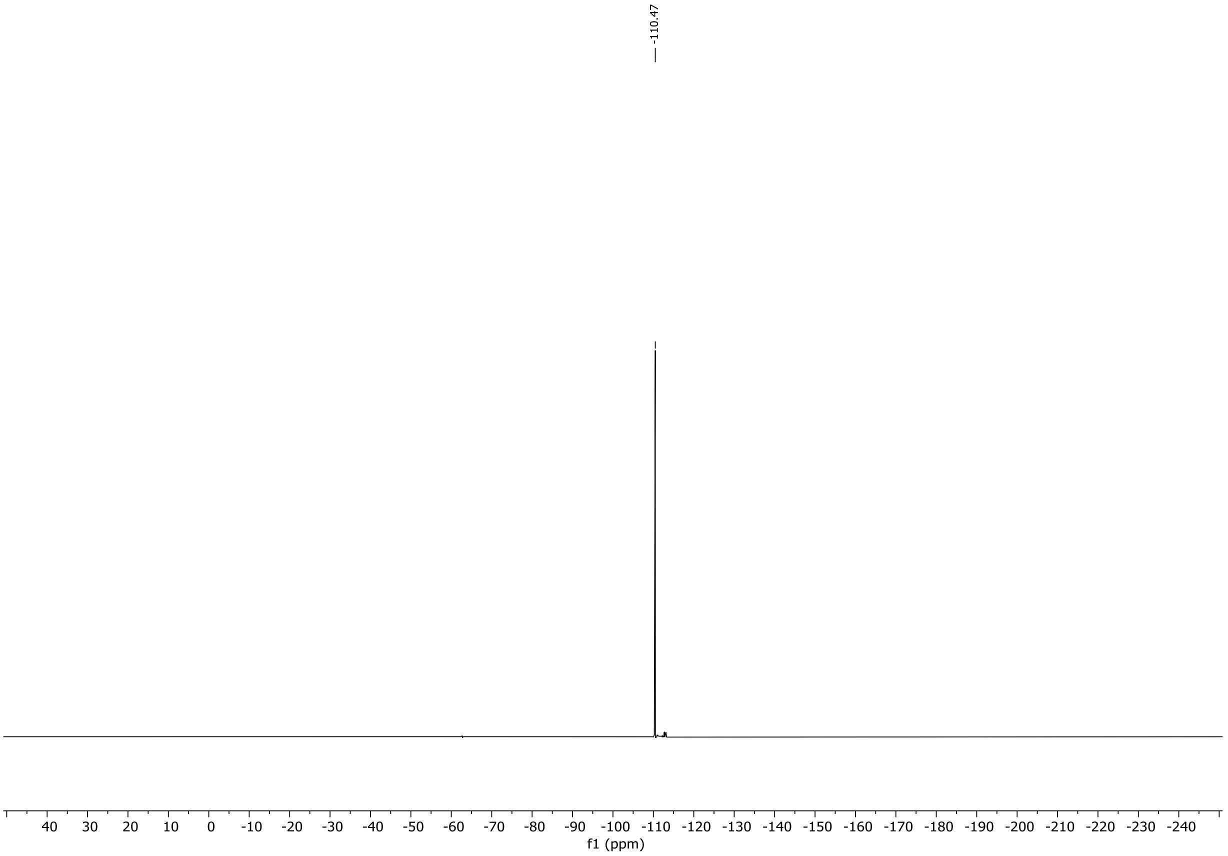

^{13}C NMR (126 MHz, $CDCl_3$) of **1c**

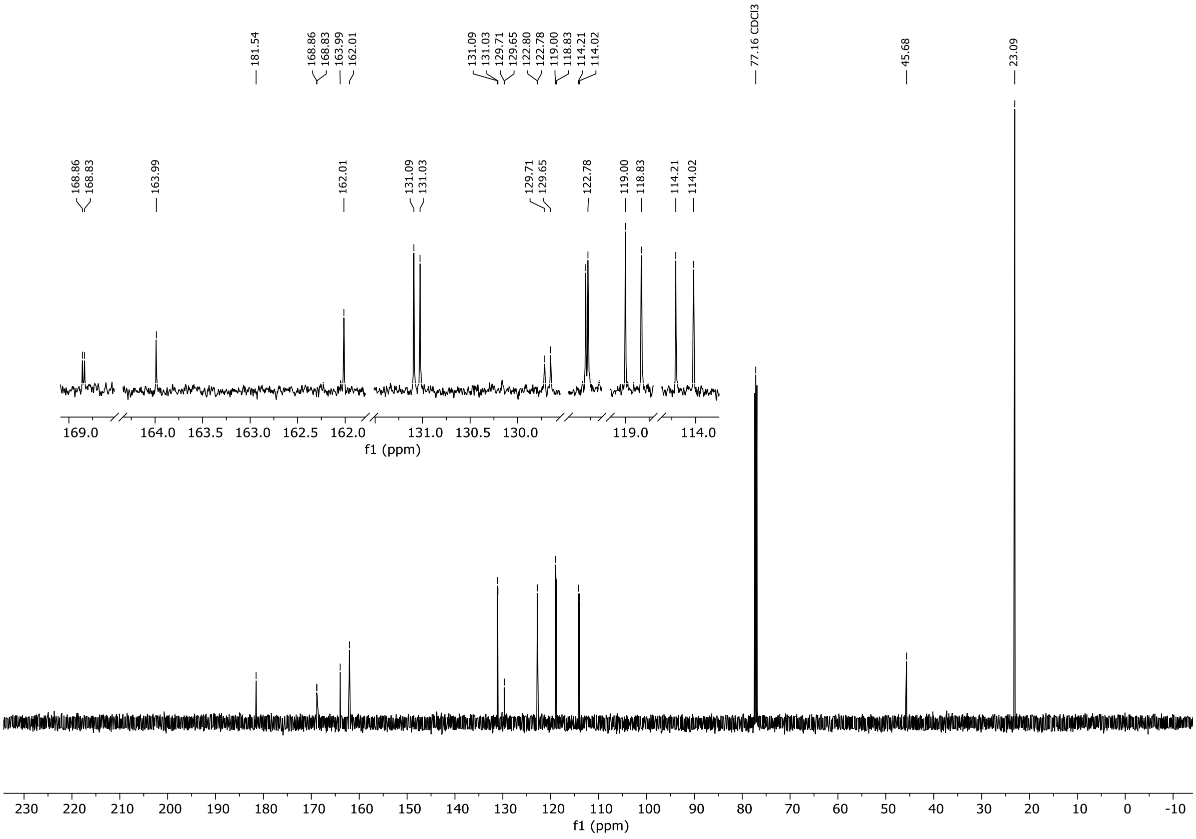

^{13}C NMR {^{1}H, ^{19}F} (126 MHz, $CDCl_3$) of **1c**

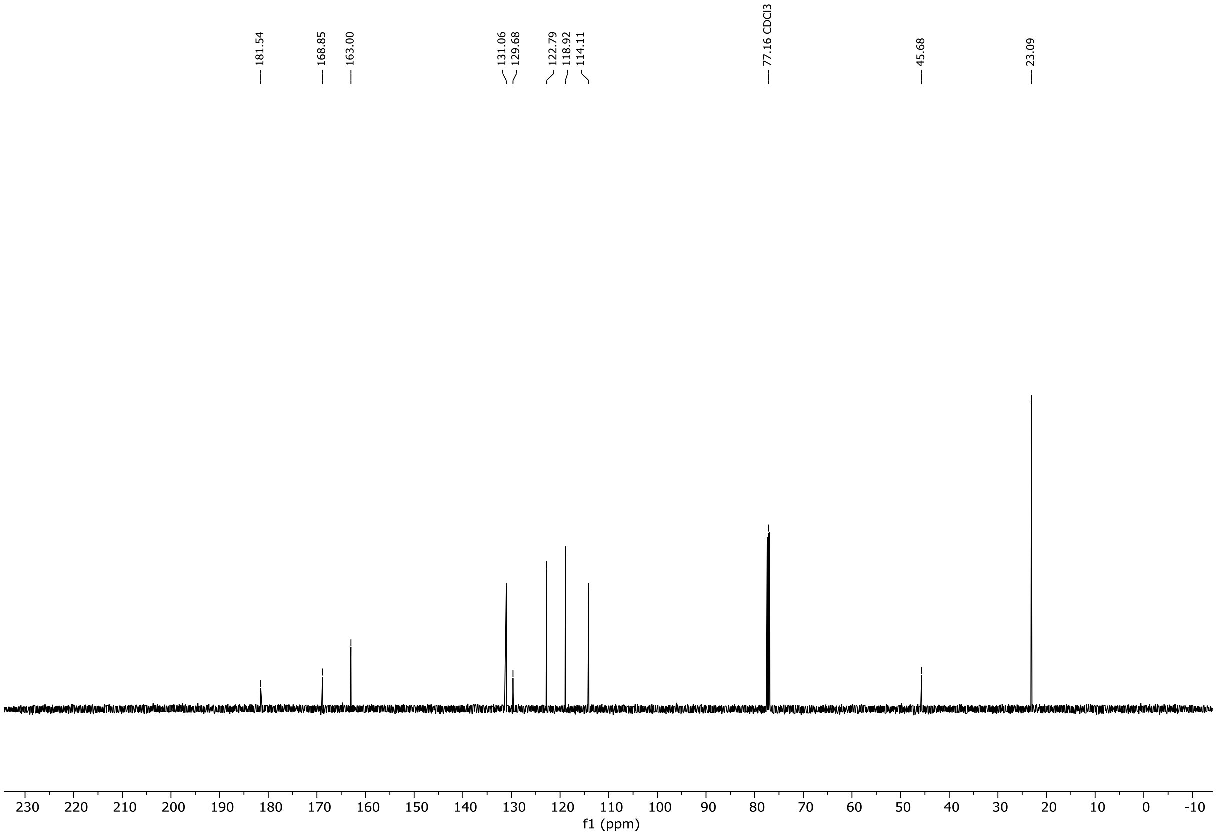

^{1}H NMR (400 MHz, $CDCl_3$) of **1d** (see procedure)

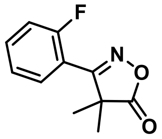

1d

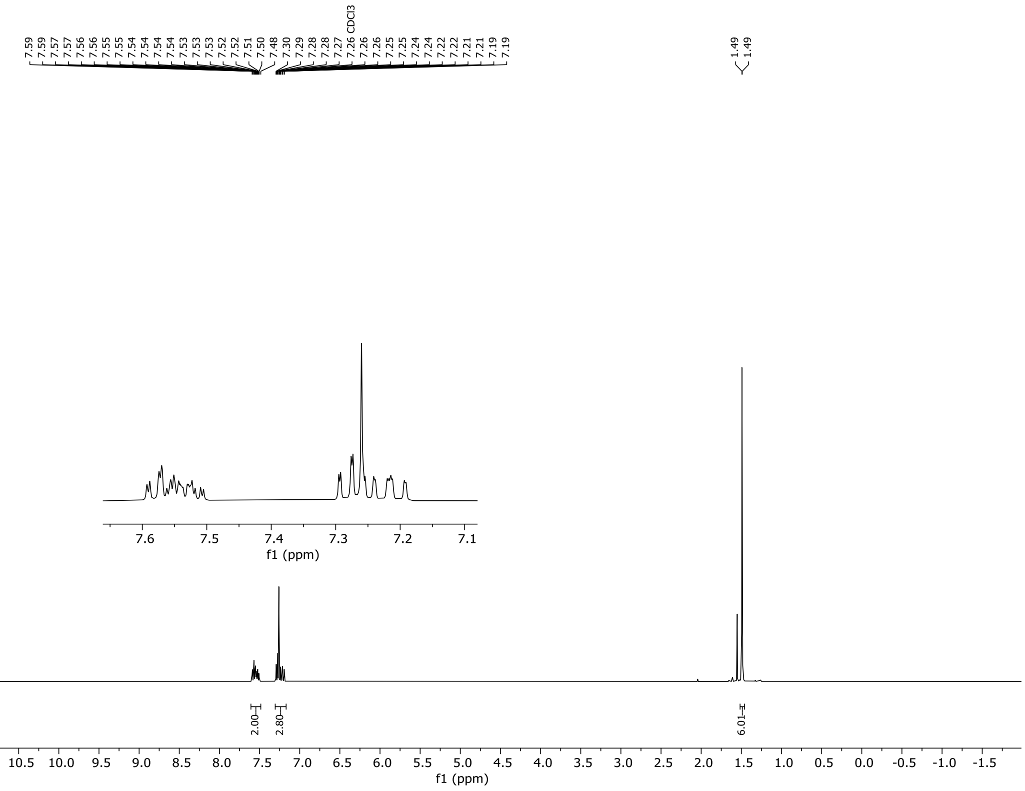

^{19}F NMR (377 MHz, $CDCl_3$) of **1d**

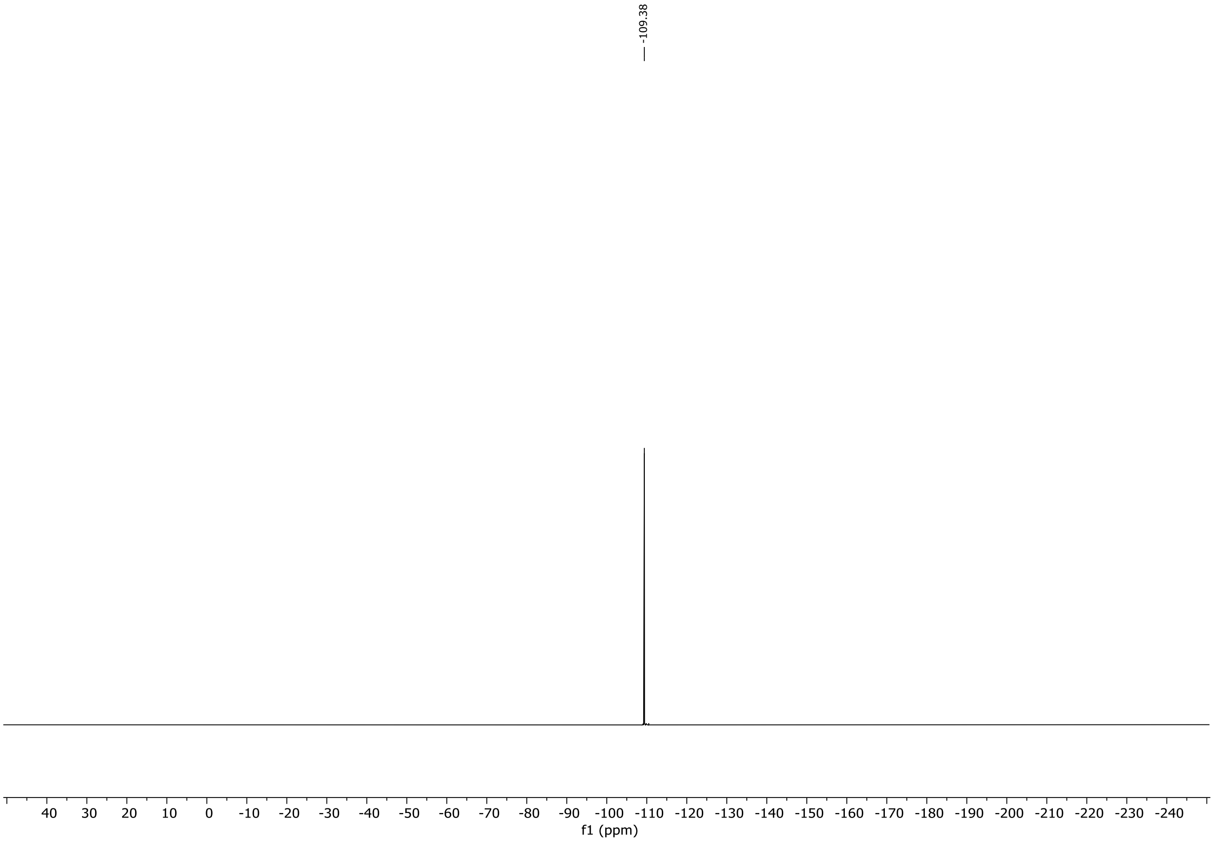

^{13}C NMR (126 MHz, $CDCl_3$) of **1d**

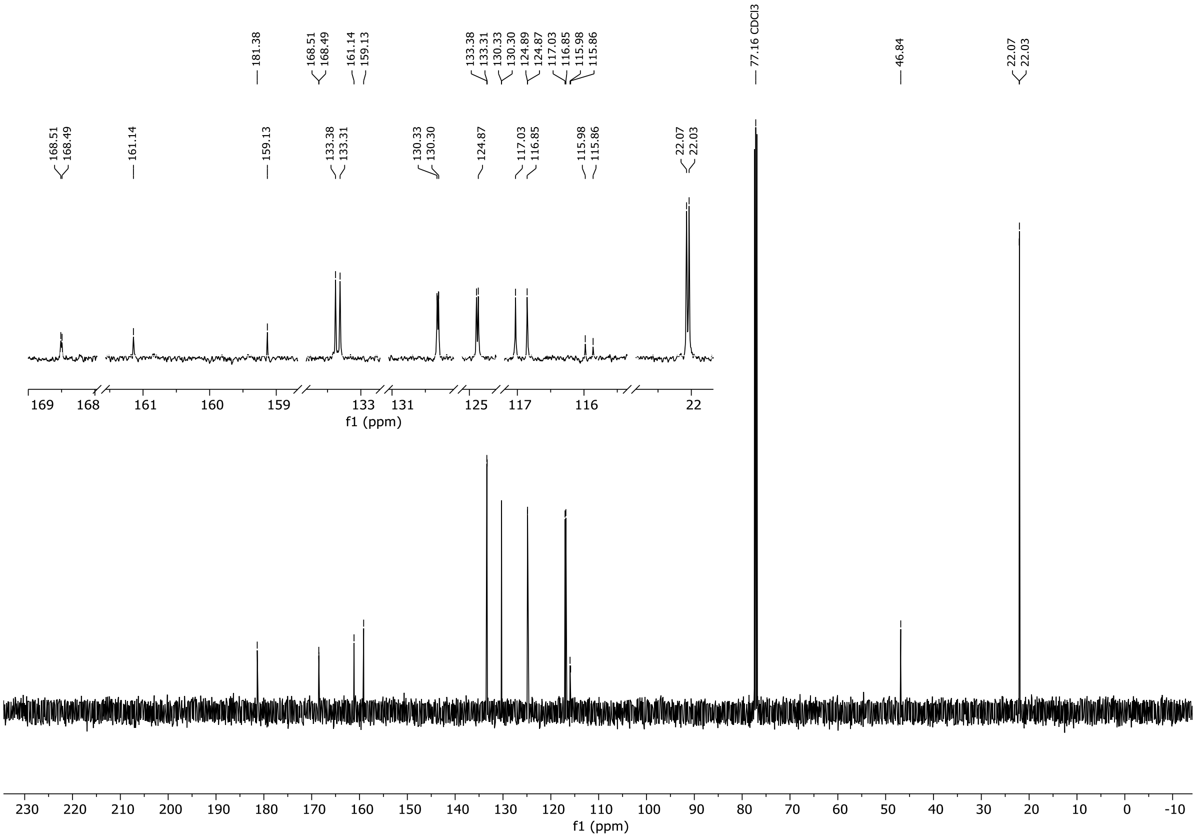

^{13}C NMR {^{1}H, ^{19}F} (126 MHz, $CDCl_3$) of **1d**

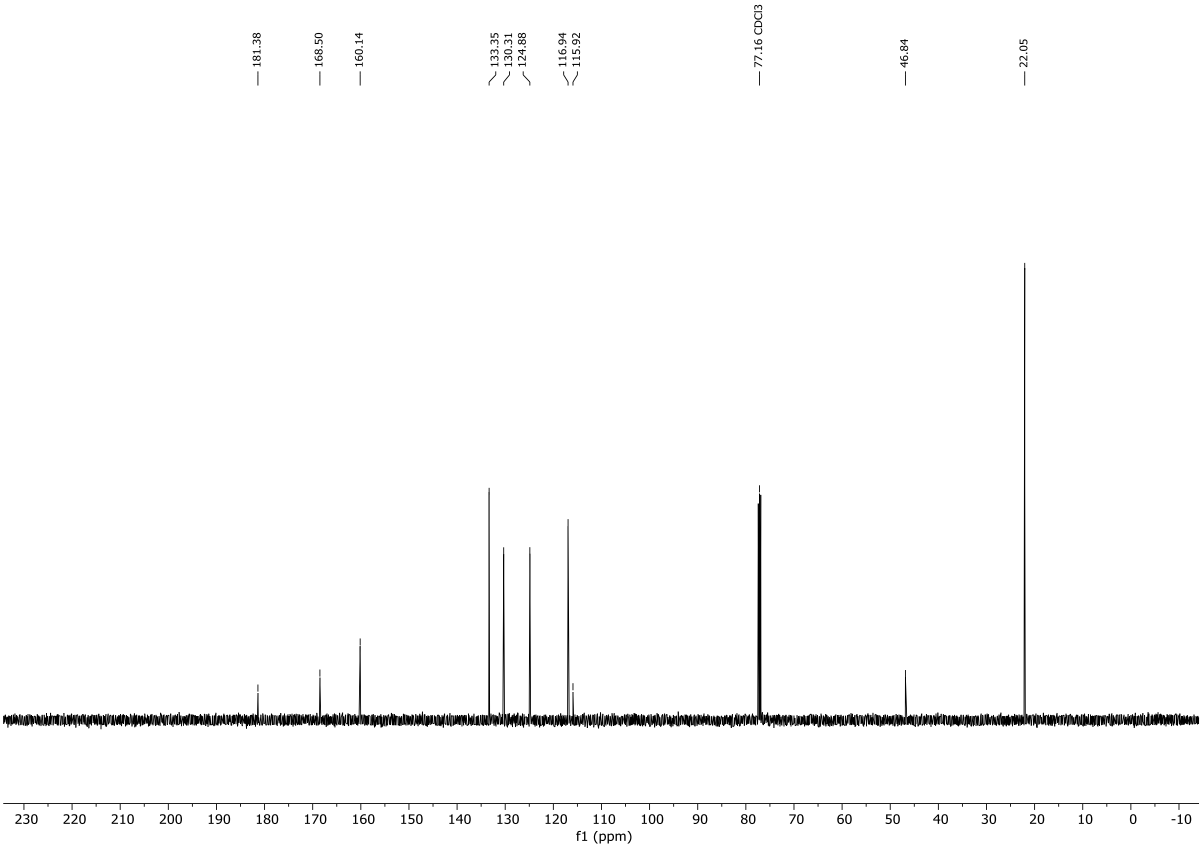

^{1}H NMR (400 MHz, $CDCl_3$) of **1e** (*see procedure*)

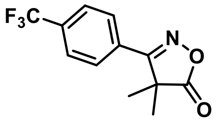

1e

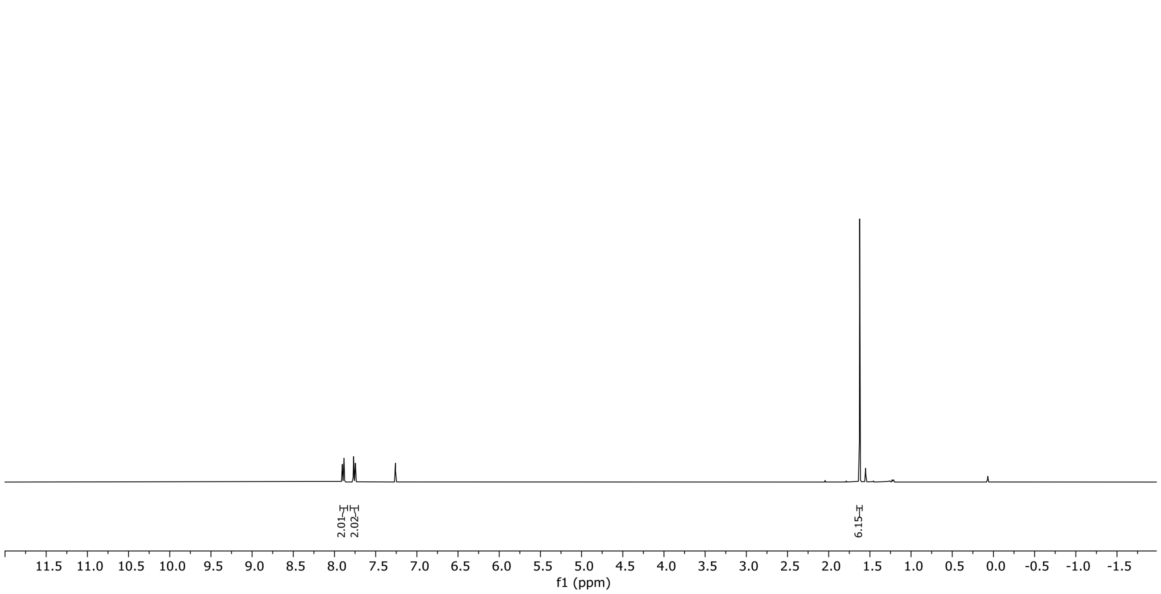

^{19}F NMR (377 MHz, $CDCl_3$) of **1e**

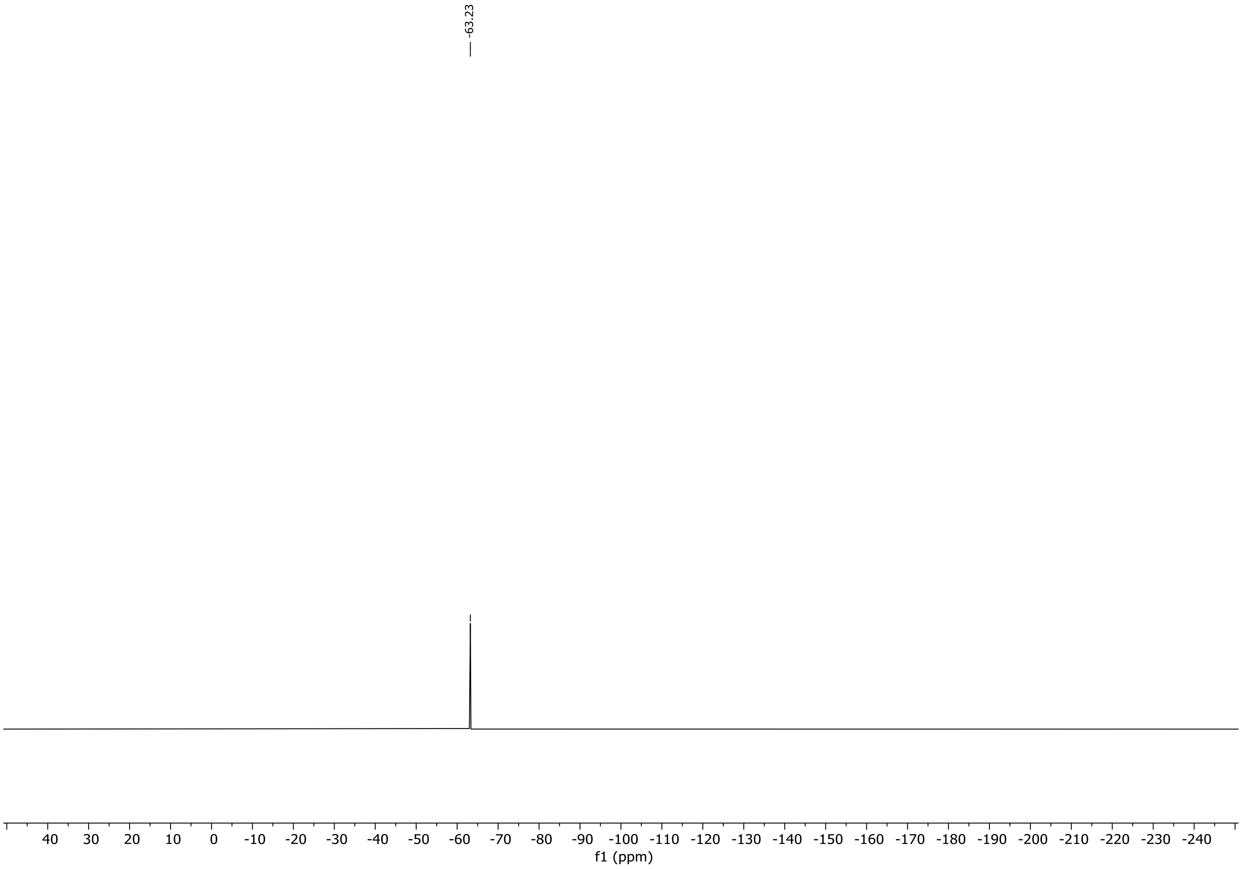

^{13}C NMR (151 MHz, $CDCl_3$) of **1e**

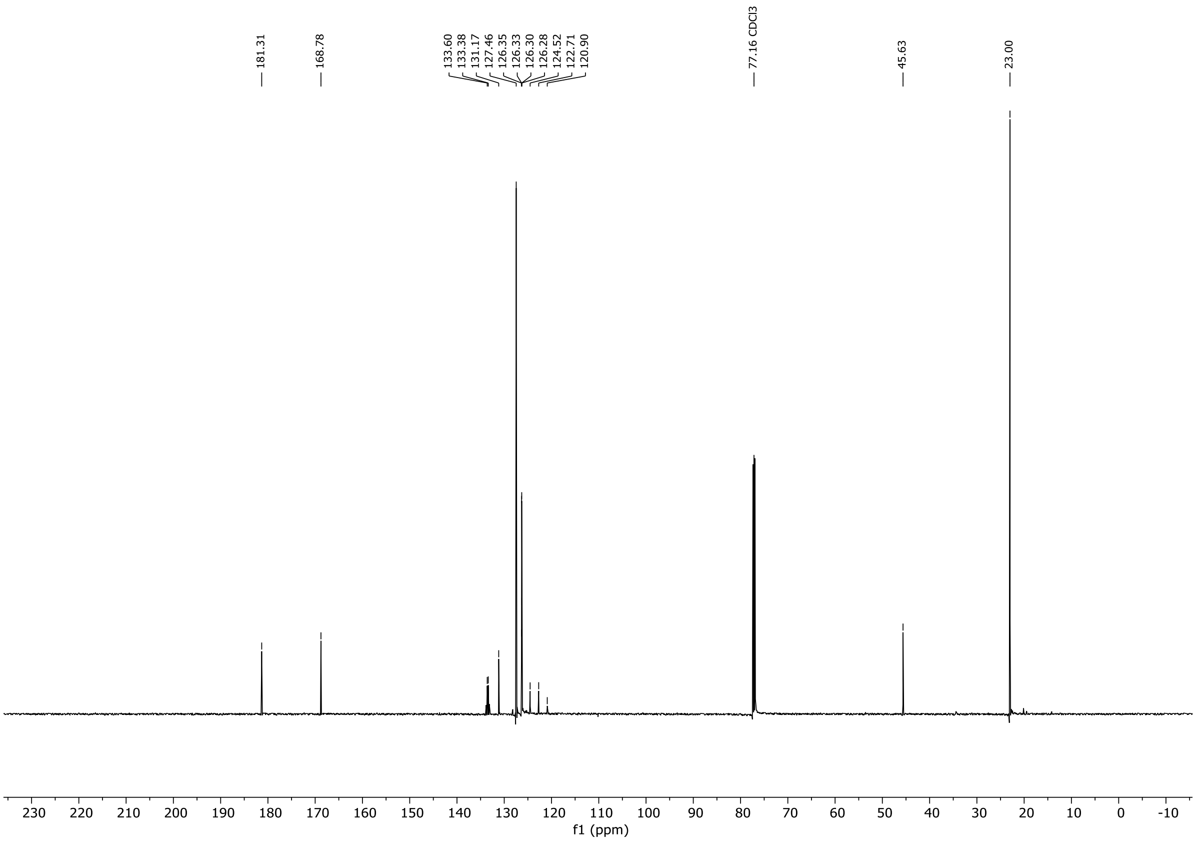

^{13}C NMR {^{1}H, ^{19}F} (126 MHz, $CDCl_3$) of **1e**

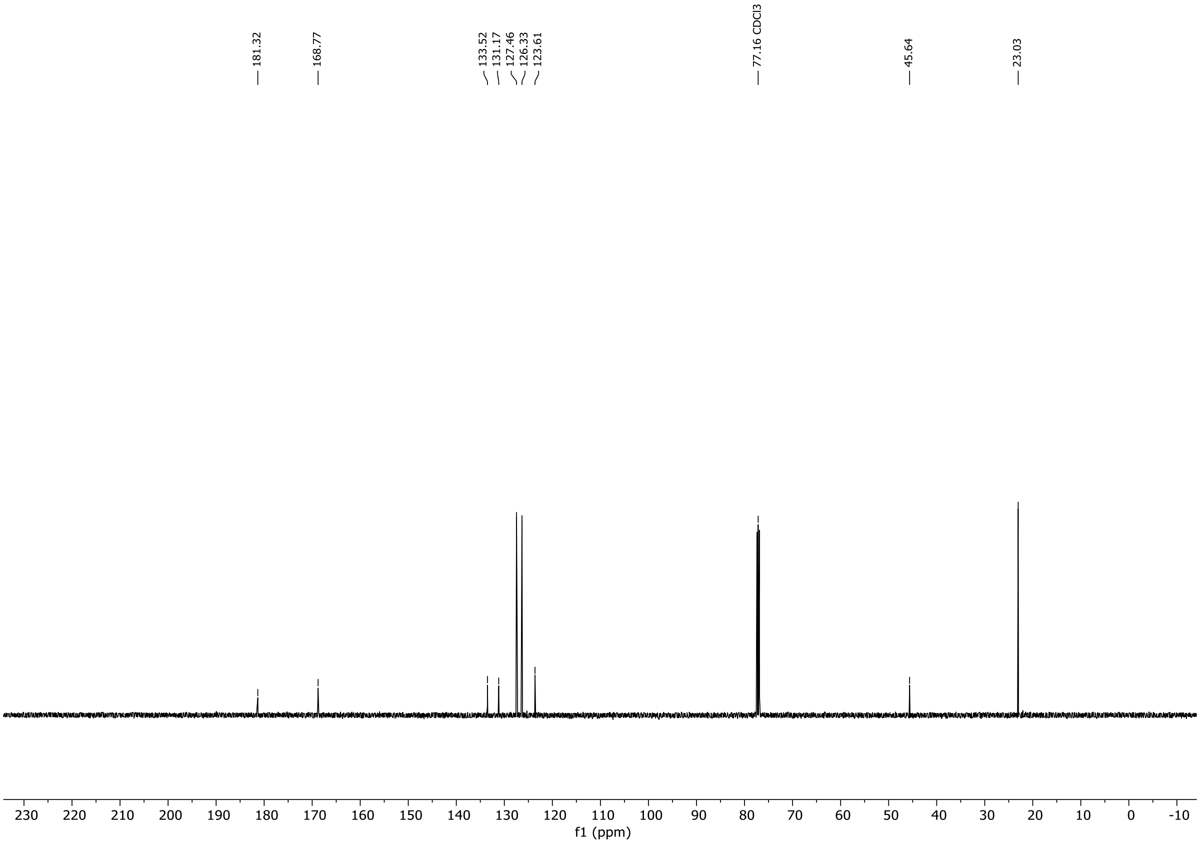

^{1}H NMR (400 MHz, $CDCl_3$) of **1f** (*see procedure*)

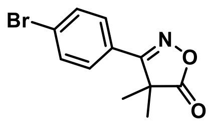

1f

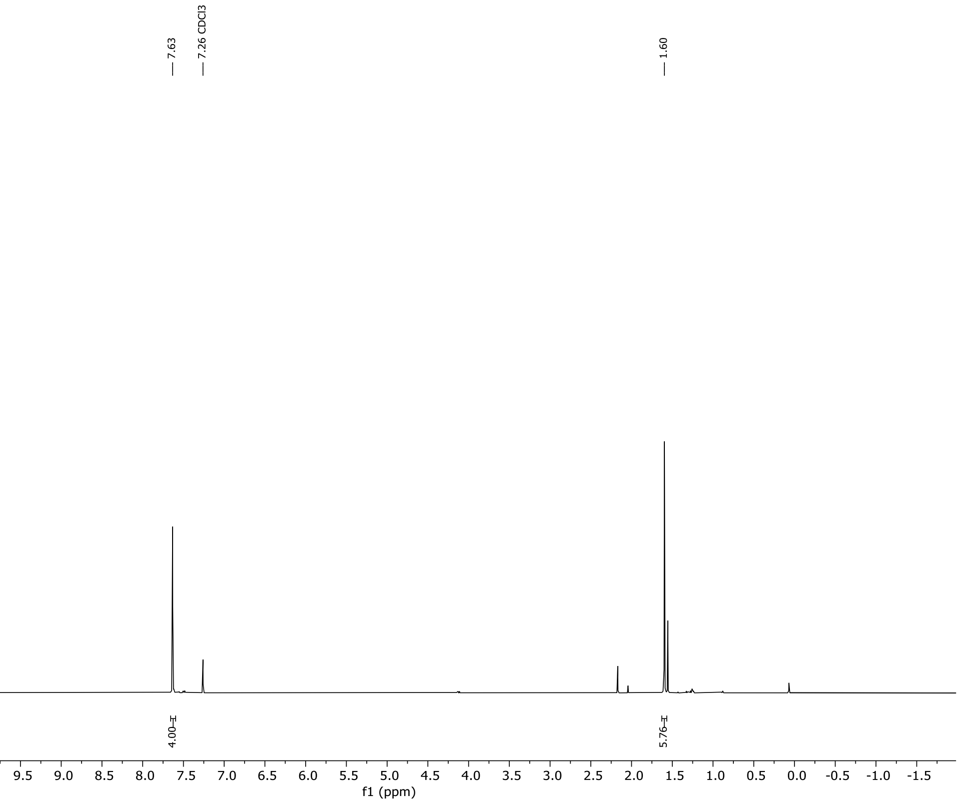

^{13}C NMR (101 MHz, $CDCl_3$) of **1f**

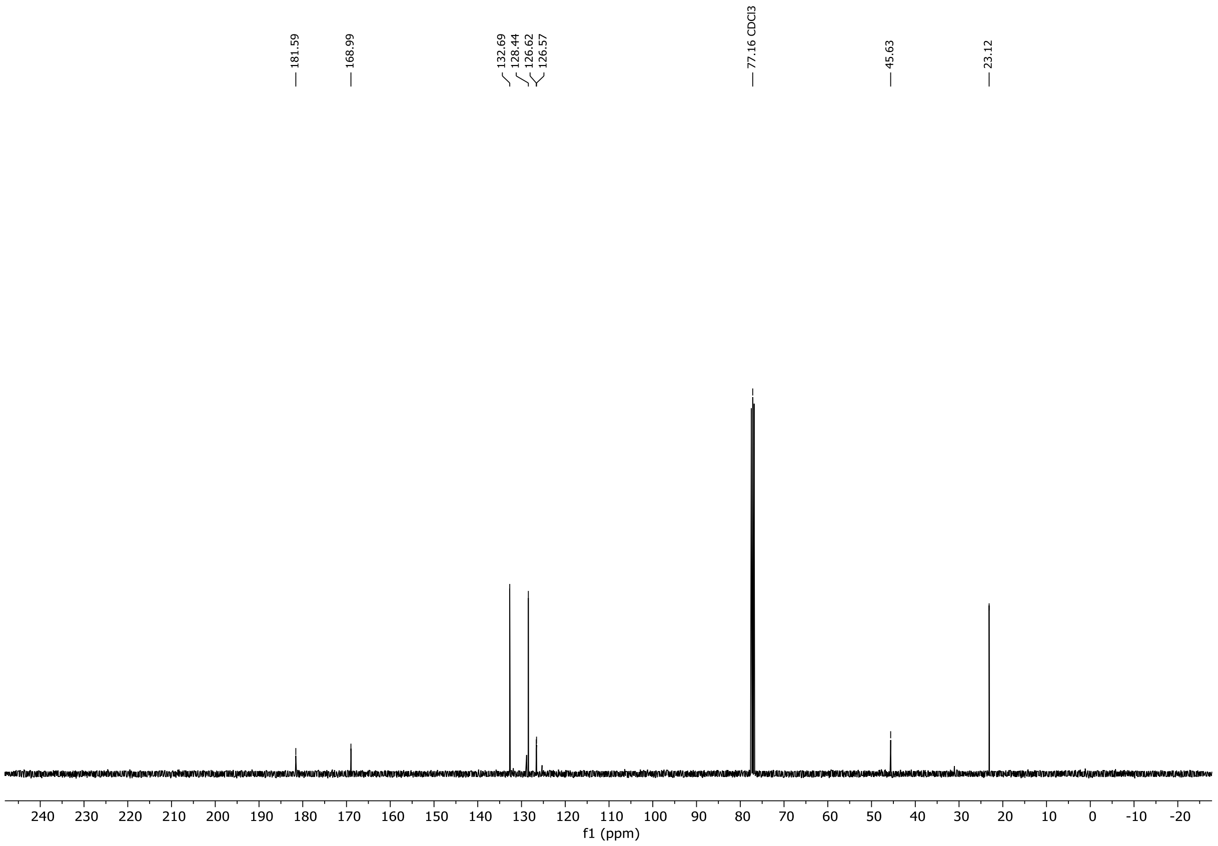

^{1}H NMR (400 MHz, $CDCl_3$) of **1g** (*see procedure*)

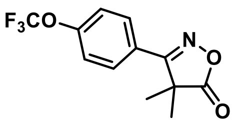

1g

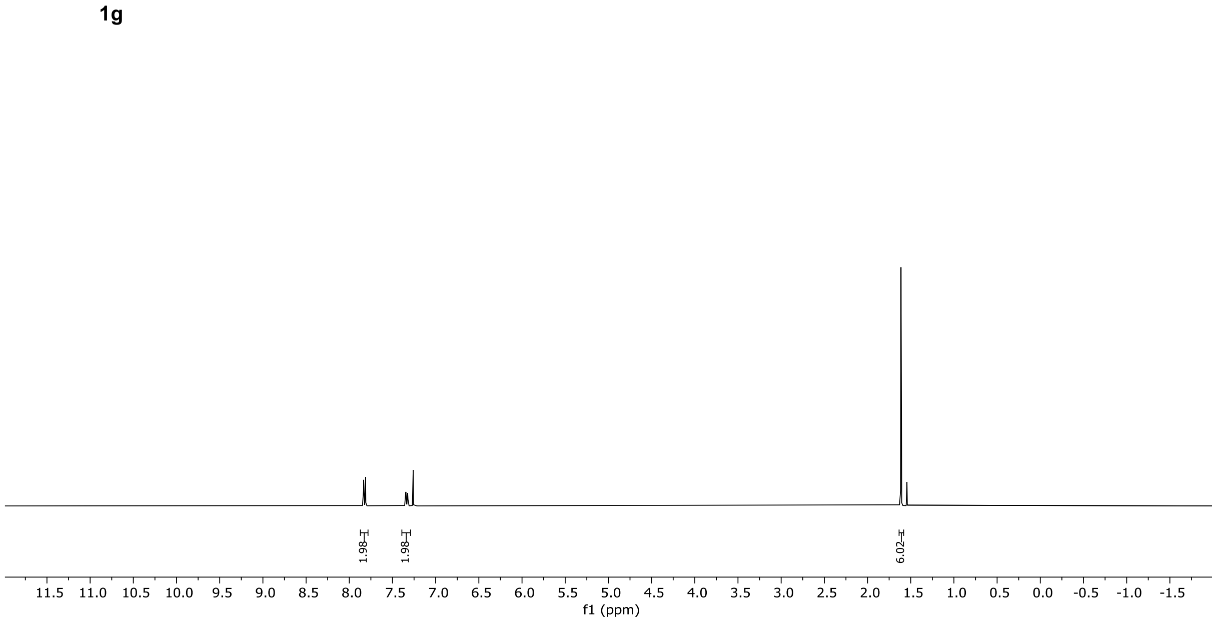

^{19}F NMR (377 MHz, $CDCl_3$) of **1g**

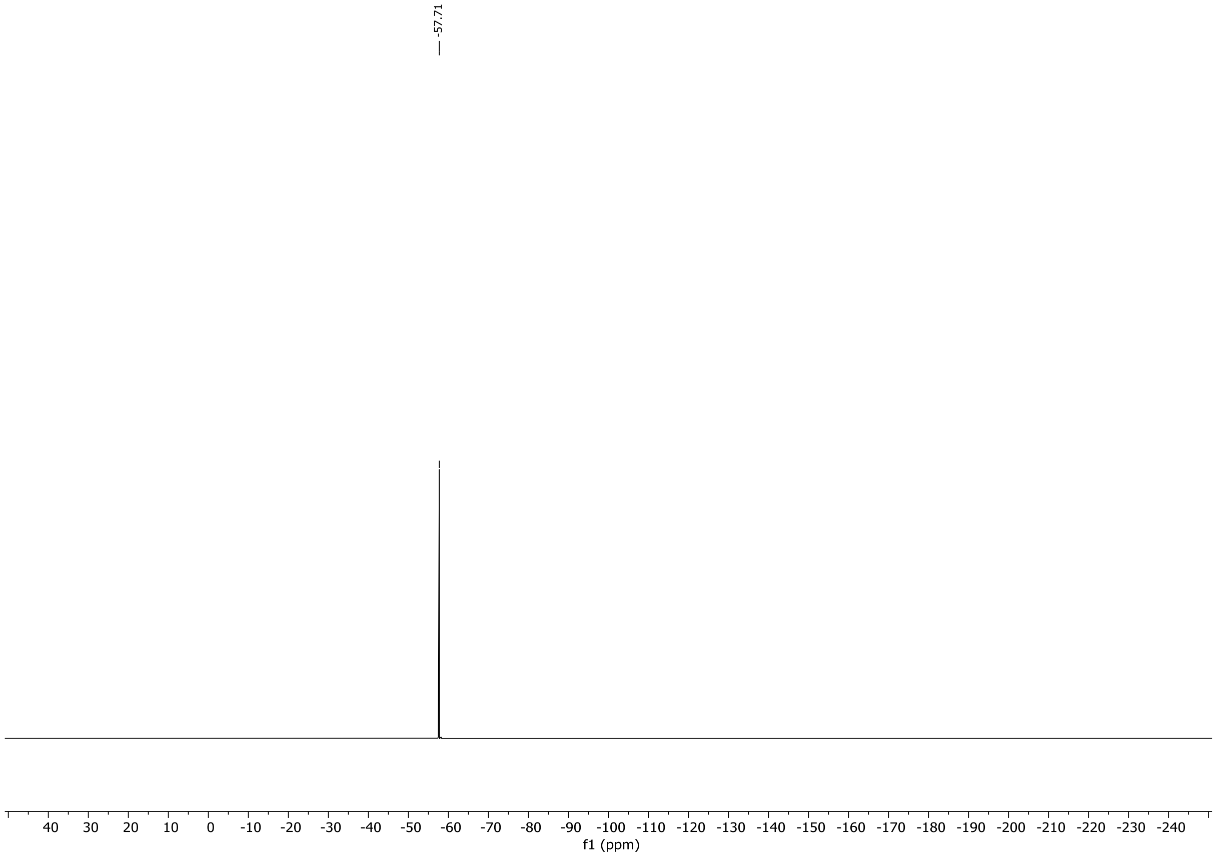

^{13}C NMR (126 MHz, $CDCl_3$) of **1g**

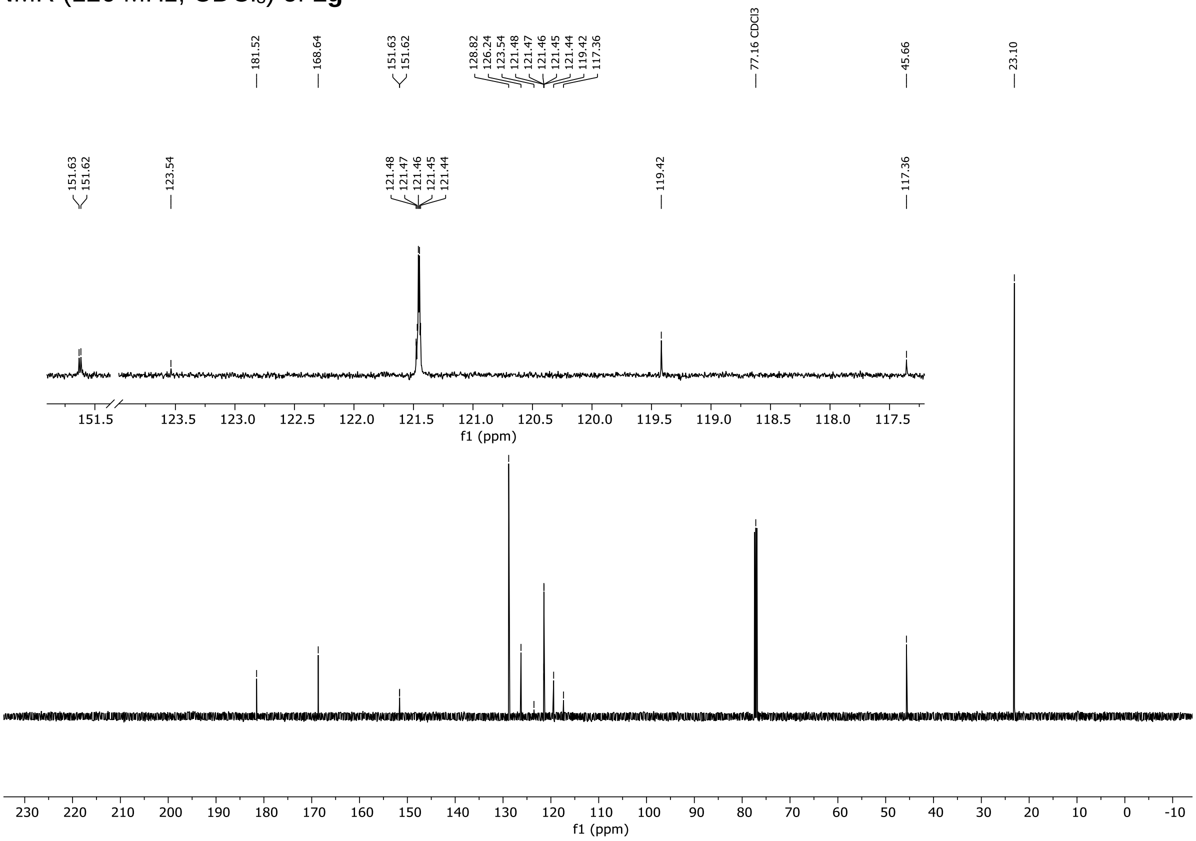

^{13}C NMR {^{1}H, ^{19}F} (101 MHz, $CDCl_3$) of **1g**

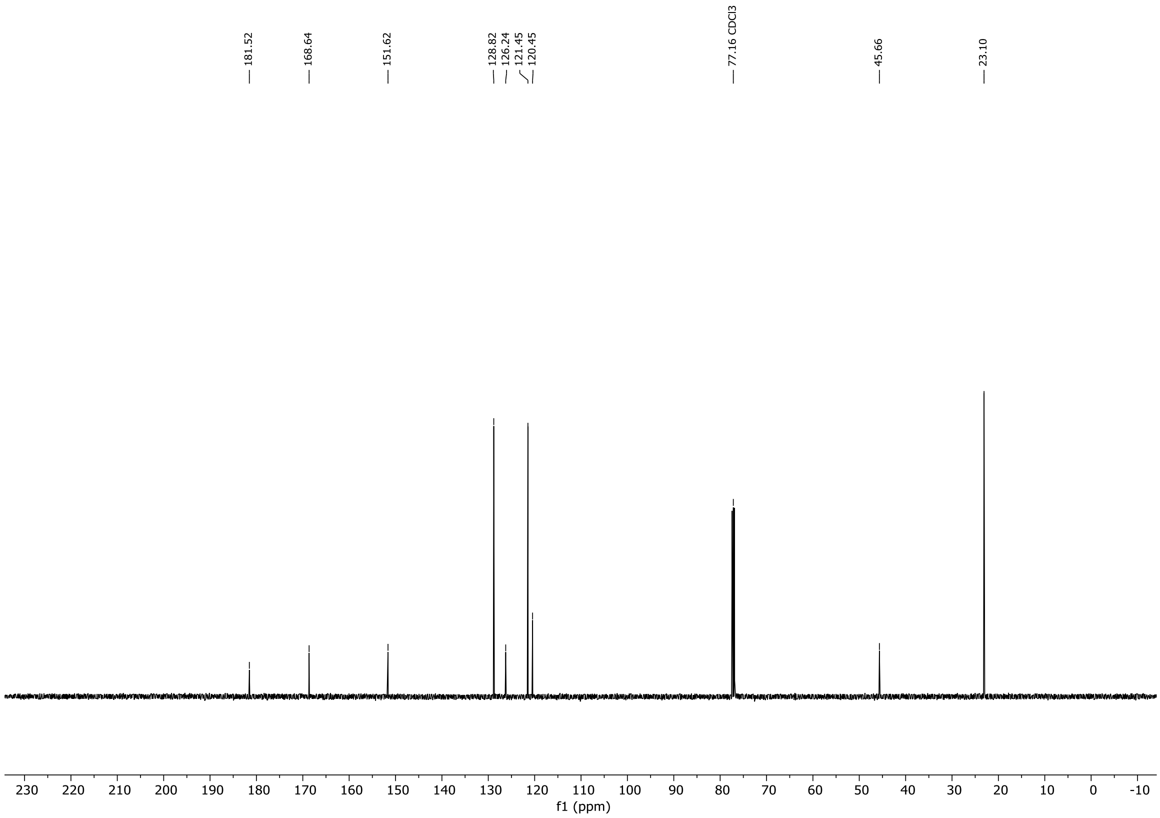

^{1}H NMR (400 MHz, $CDCl_3$) of **1h** (*see procedure*)

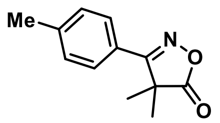

1h

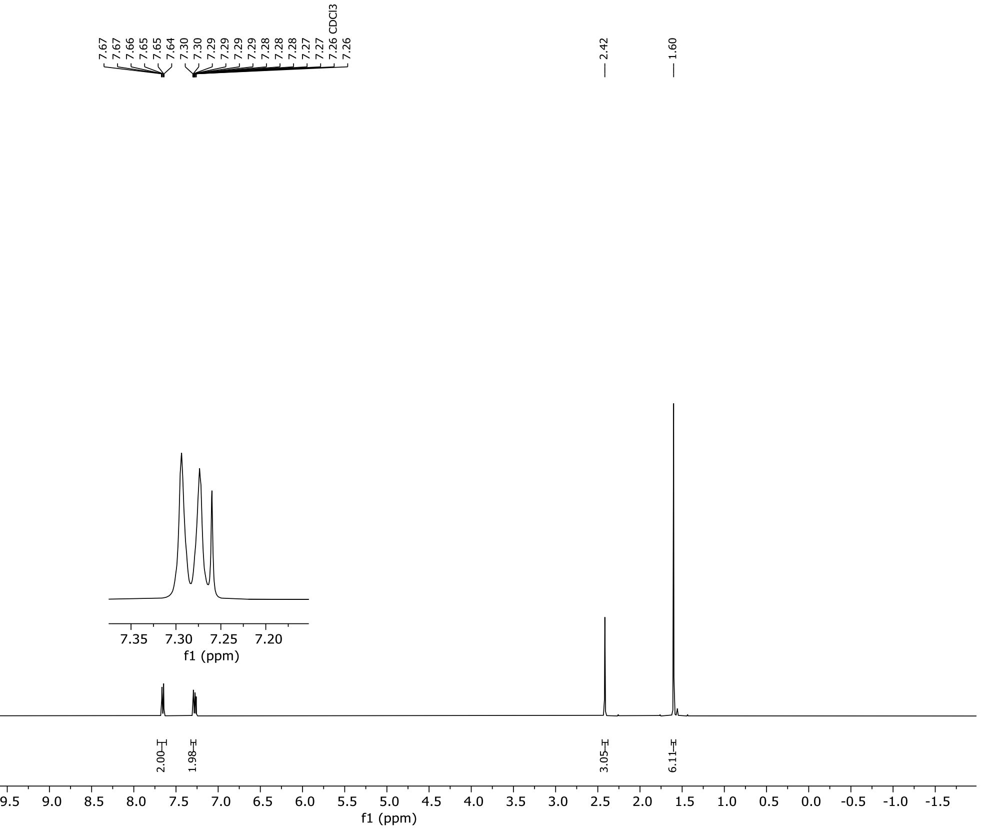

^{13}C NMR (101 MHz, $CDCl_3$) of **1h**

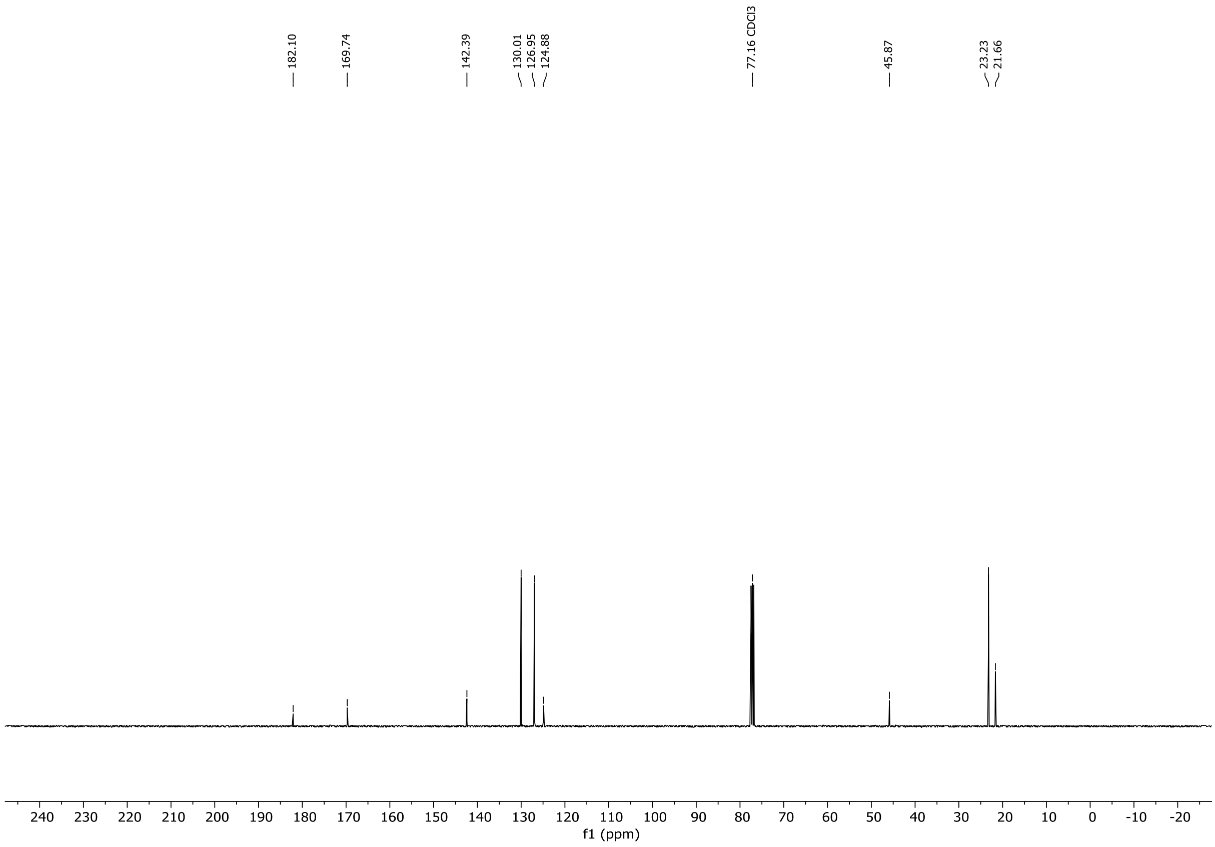

^{1}H NMR (400 MHz, $CDCl_3$) of **1i** (*see procedure*)

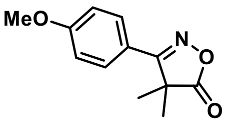

1i

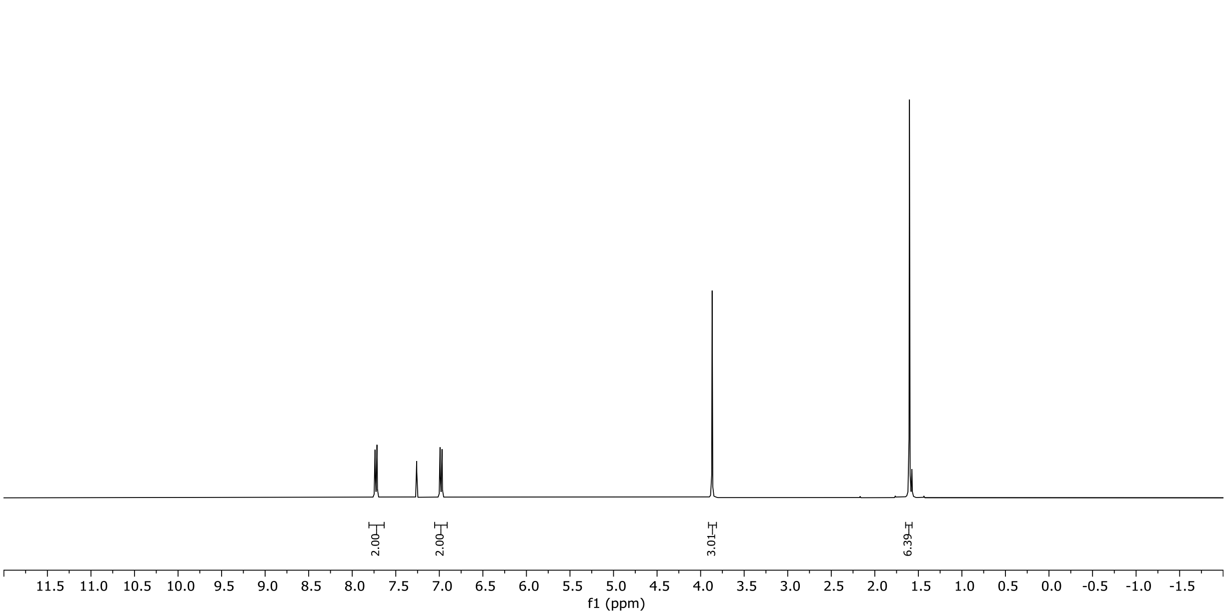

^{13}C NMR (101 MHz, $CDCl_3$) of **1i**

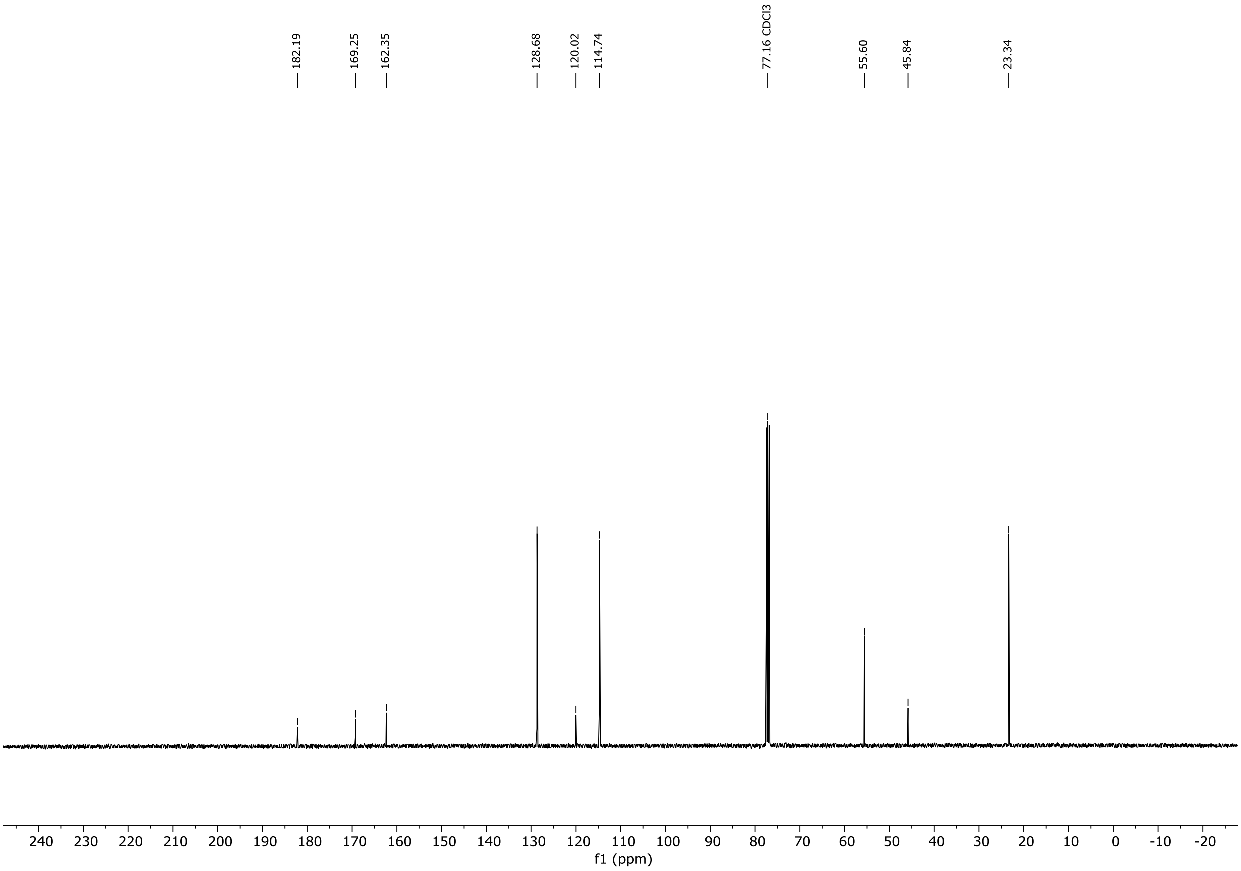

^{1}H NMR (400 MHz, $CDCl_3$) of **1j** (*see procedure*)

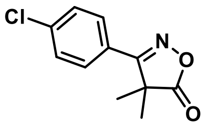

1j

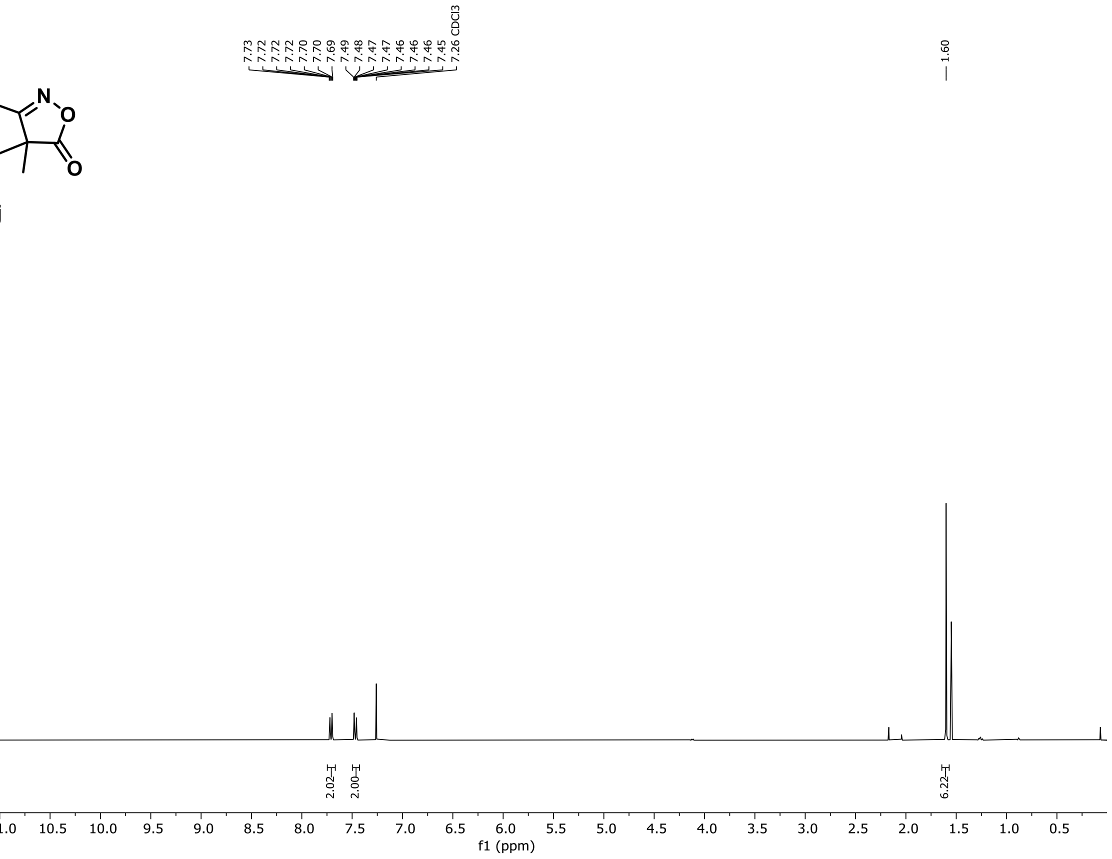

^{13}C NMR (101 MHz, $CDCl_3$) of **1j**

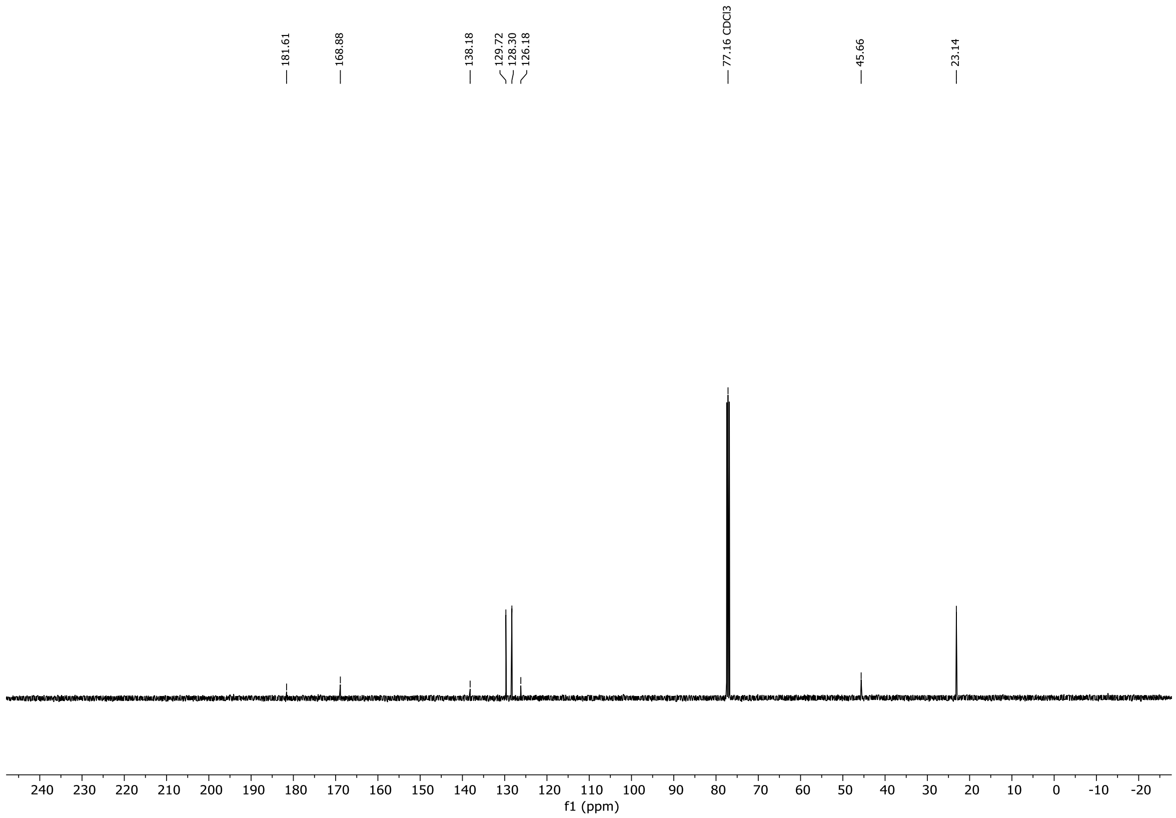

^{1}H NMR (400 MHz, $CDCl_3$) of **S1** (see procedure)

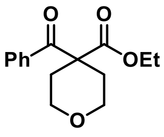

S1

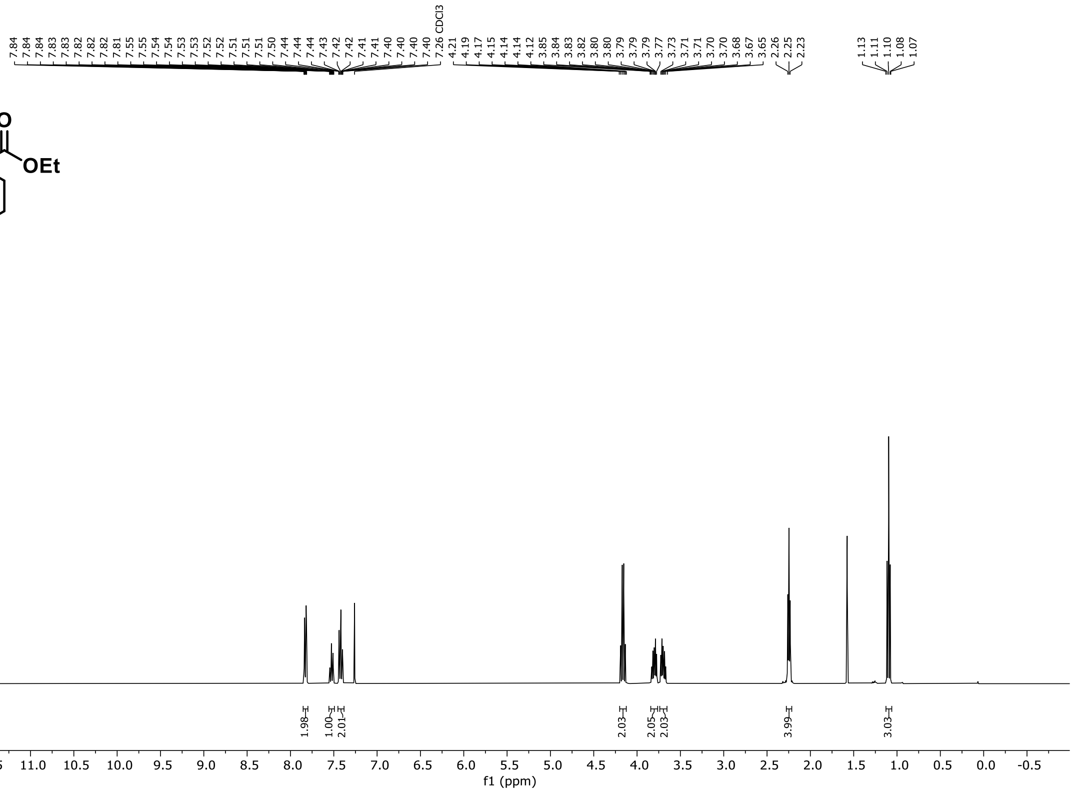

^{13}C NMR (101 MHz, $CDCl_3$) of **S1**

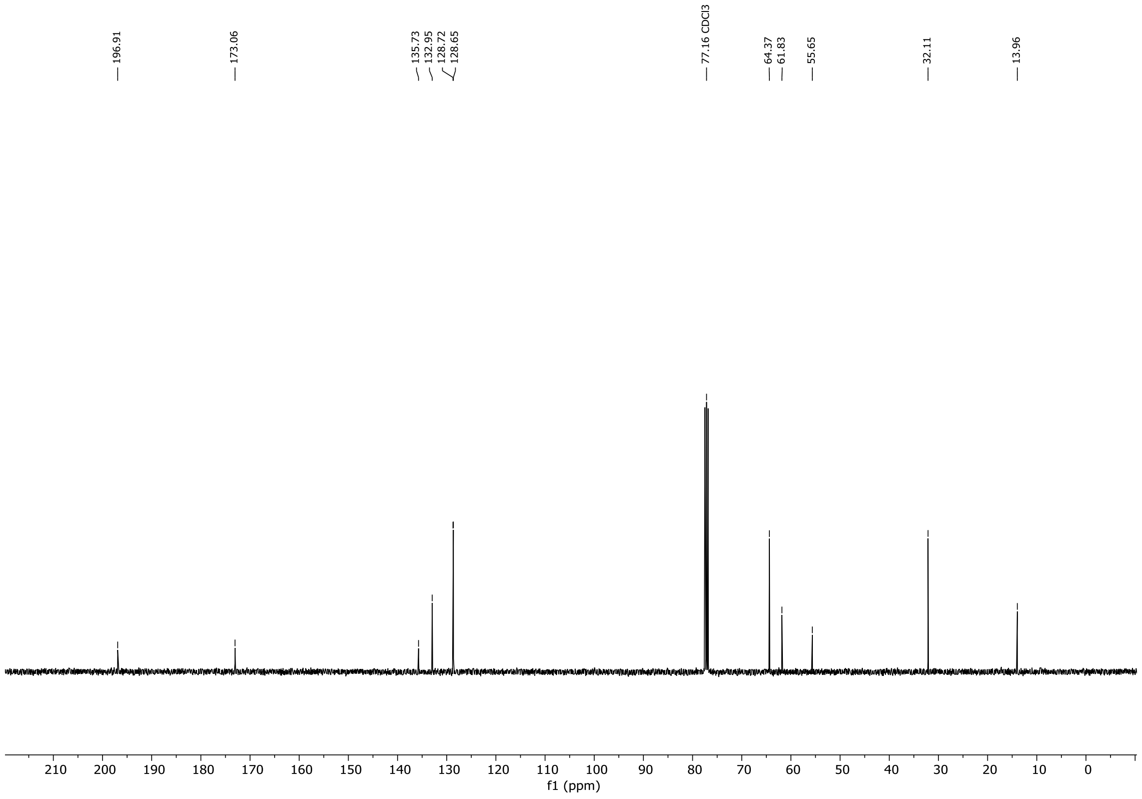

^{1}H NMR (400 MHz, $CDCl_3$) of **1k** (*see procedure*)

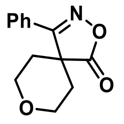

1k

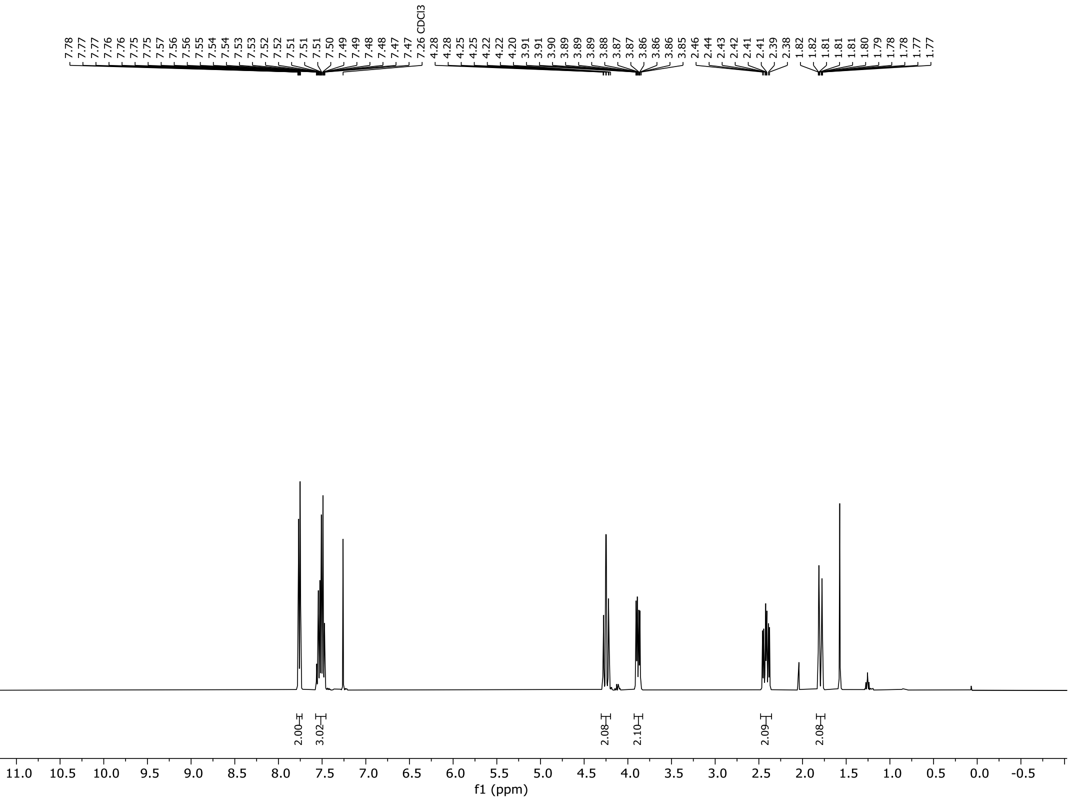

^{13}C NMR (101 MHz, $CDCl_3$) of **1k**

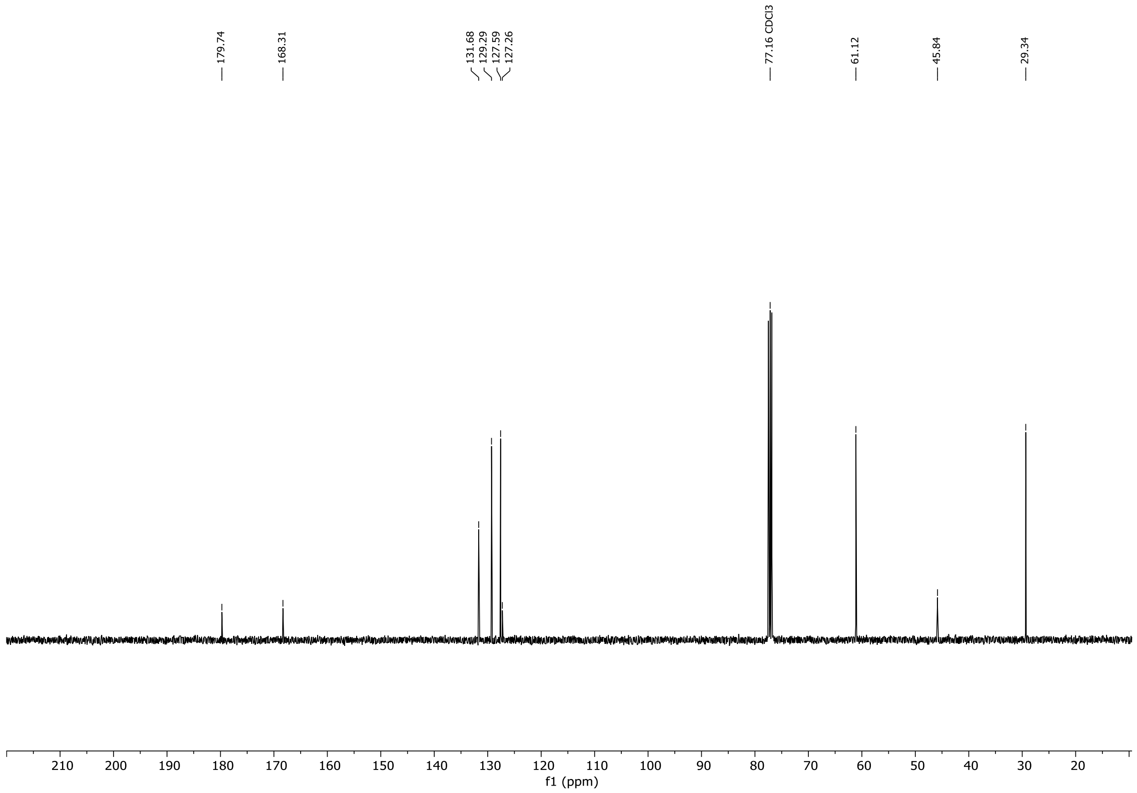

^{1}H NMR (400 MHz, $CDCl_3$) of **S2** (*see procedure*)

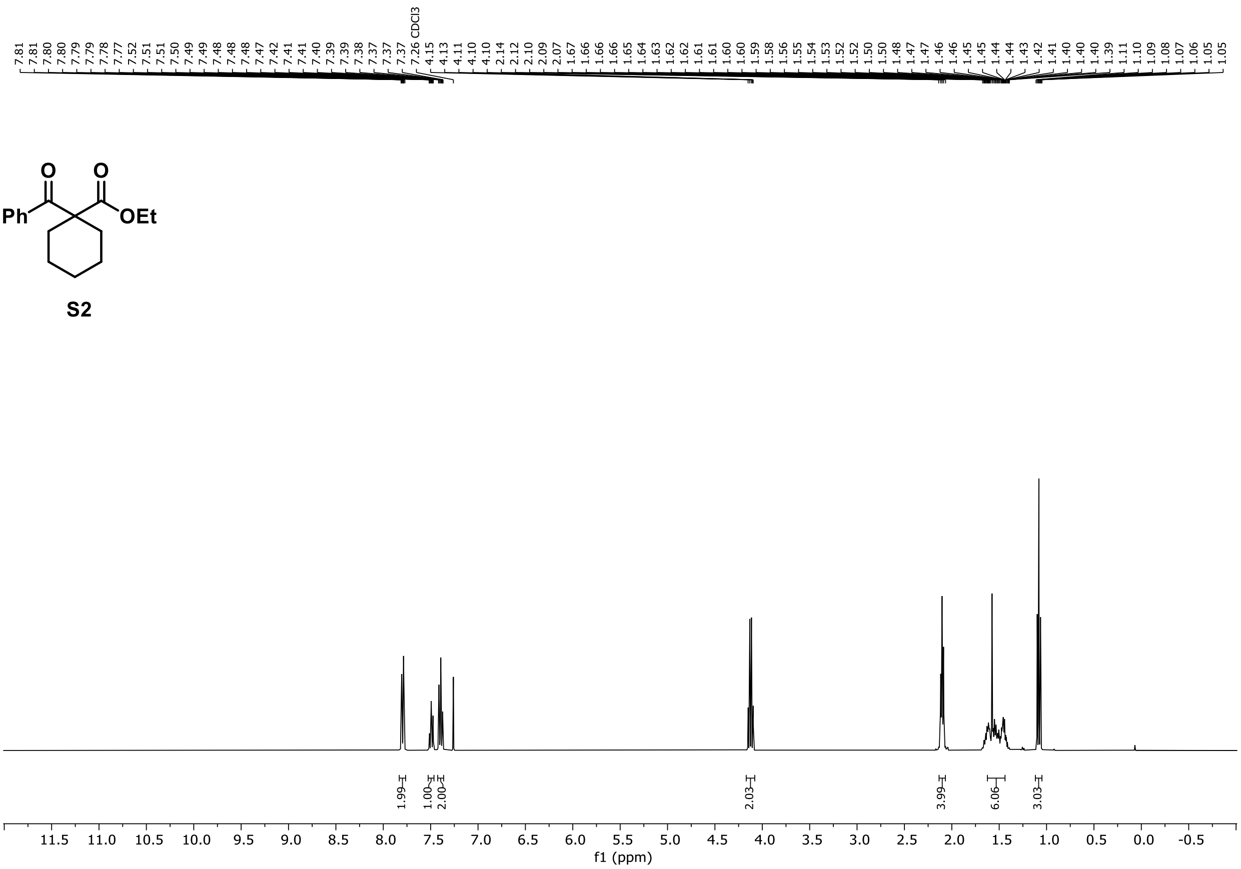

^{13}C NMR (101 MHz, $CDCl_3$) of **S2**

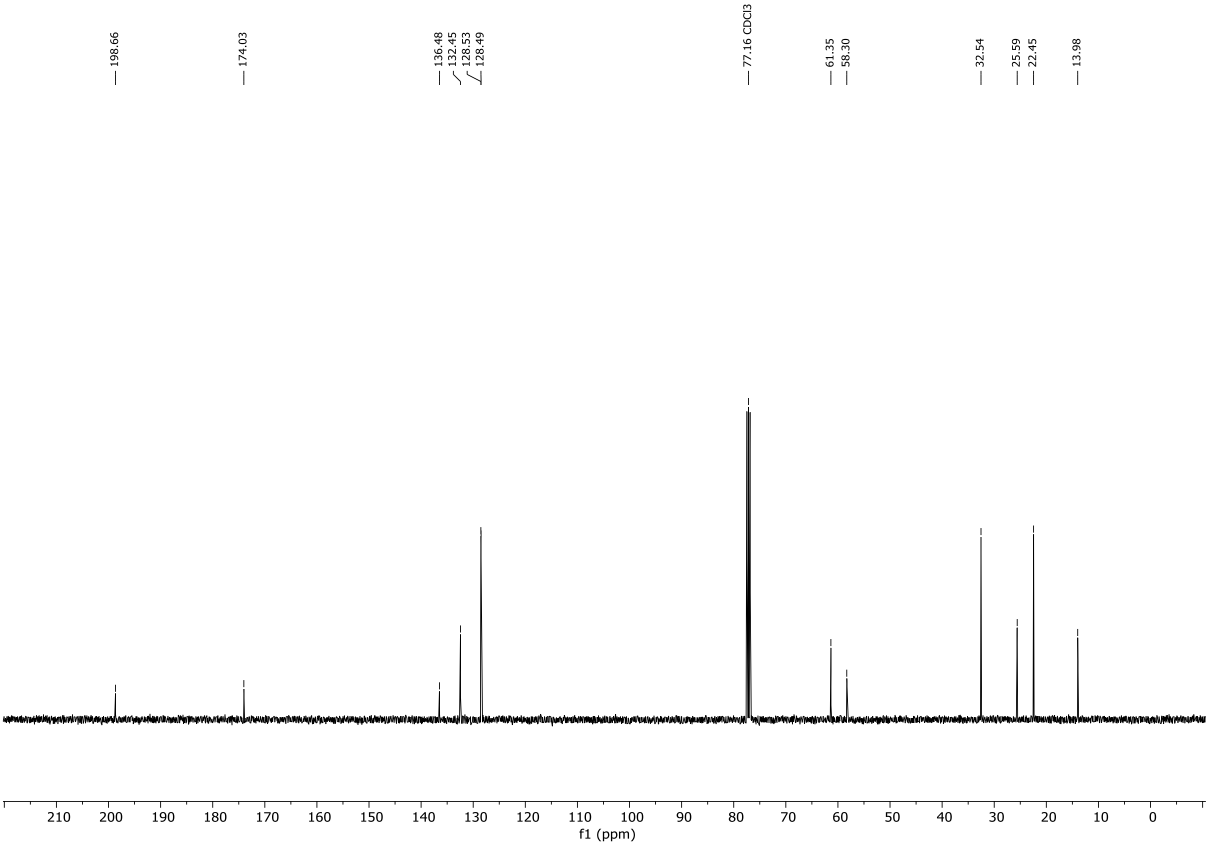

^{1}H NMR (400 MHz, $CDCl_3$) of **1l** (*see procedure*)

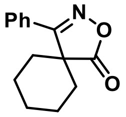

1l

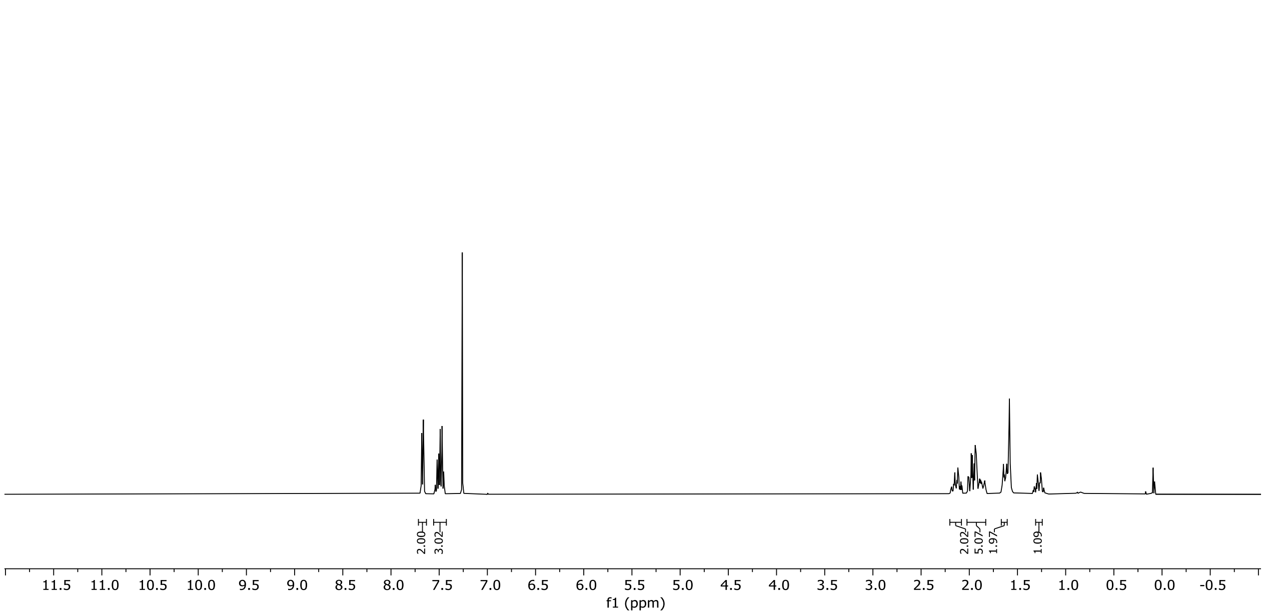

^{13}C NMR (101 MHz, $CDCl_3$) of **1l**

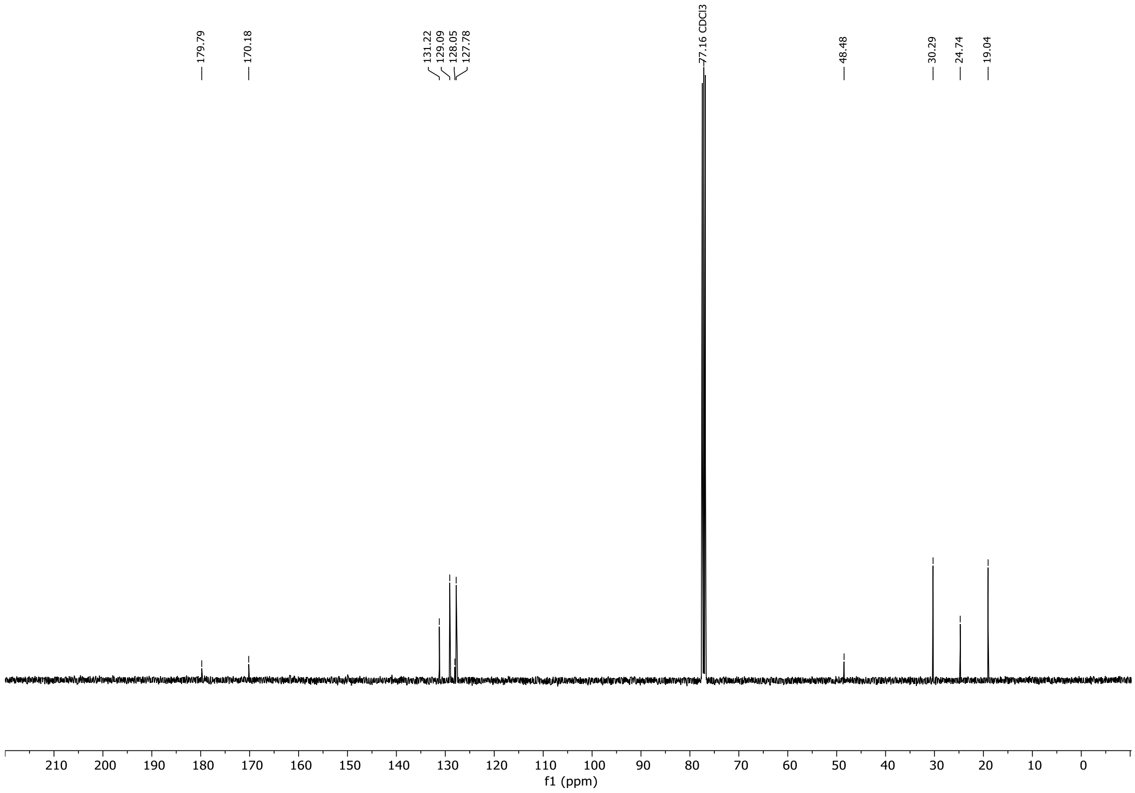

^{1}H NMR (400 MHz, $CDCl_3$) of **4a** (*see procedure*)

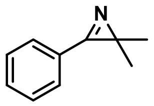

4a

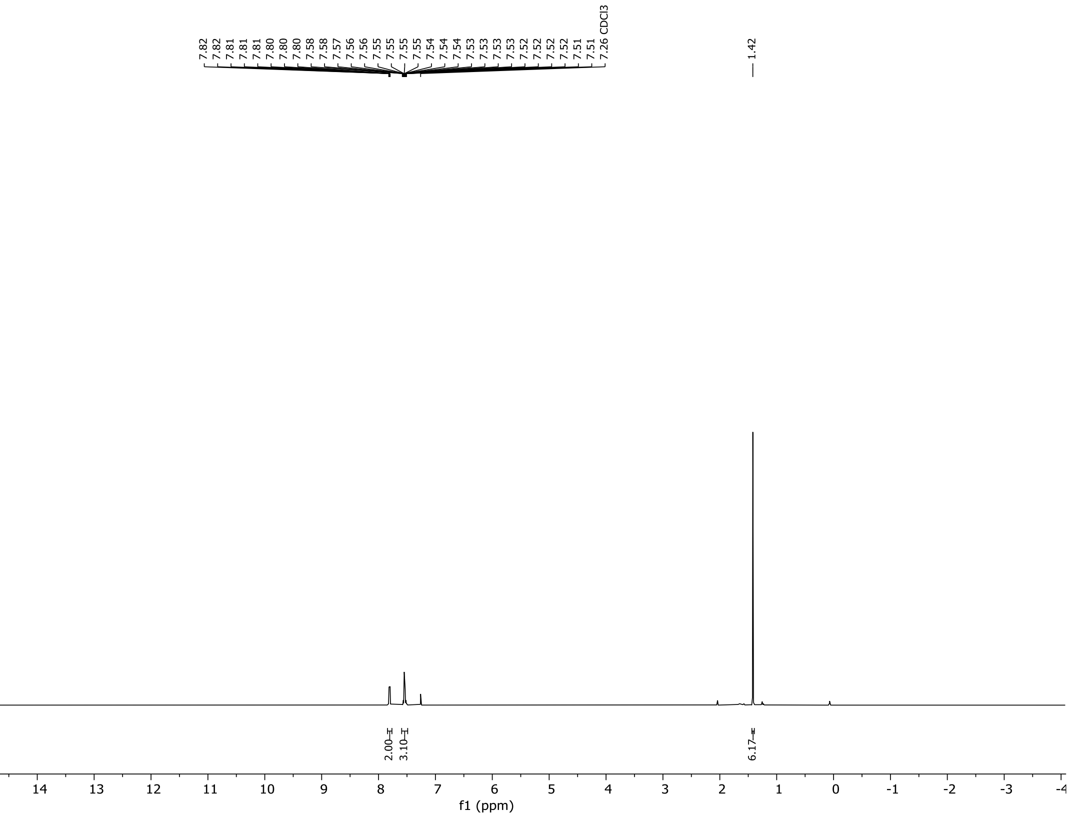

^{13}C NMR (101 MHz, $CDCl_3$) of **4a**

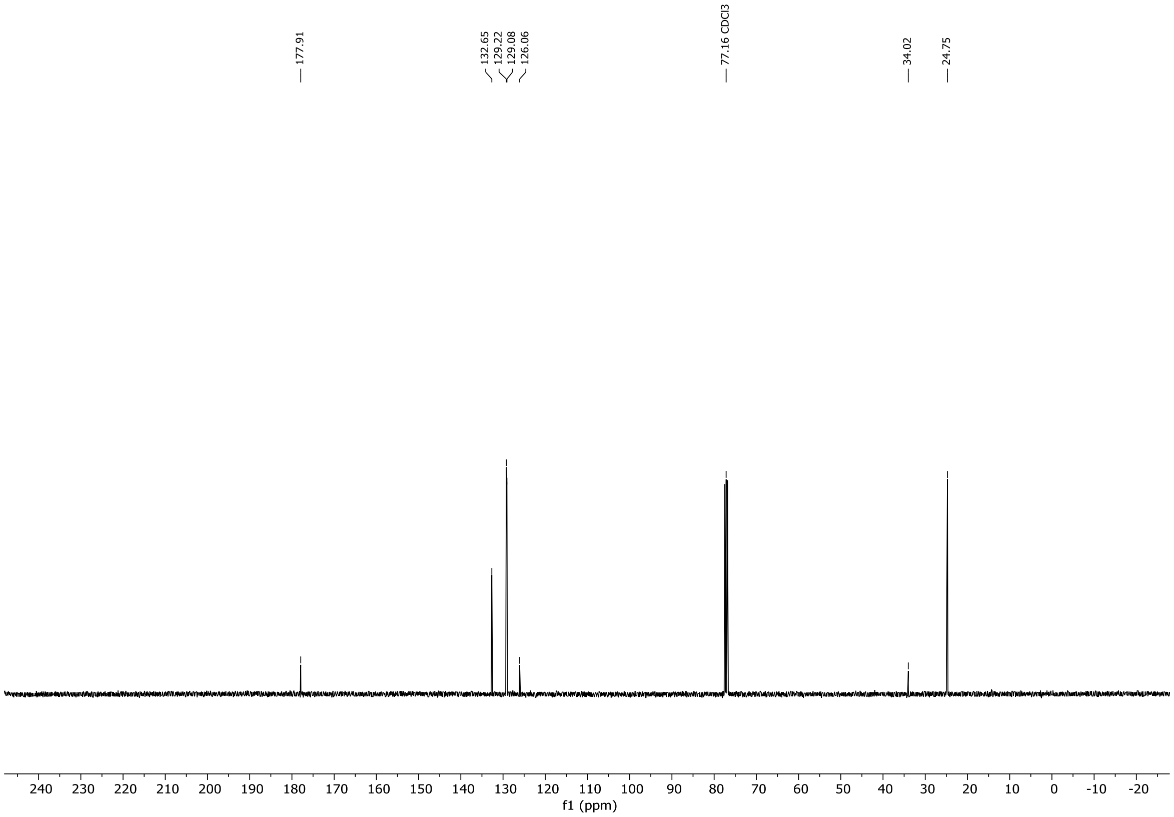

^{1}H NMR (400 MHz, $CDCl_3$) of **S3** (*see procedure*)

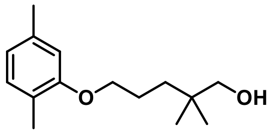

S3

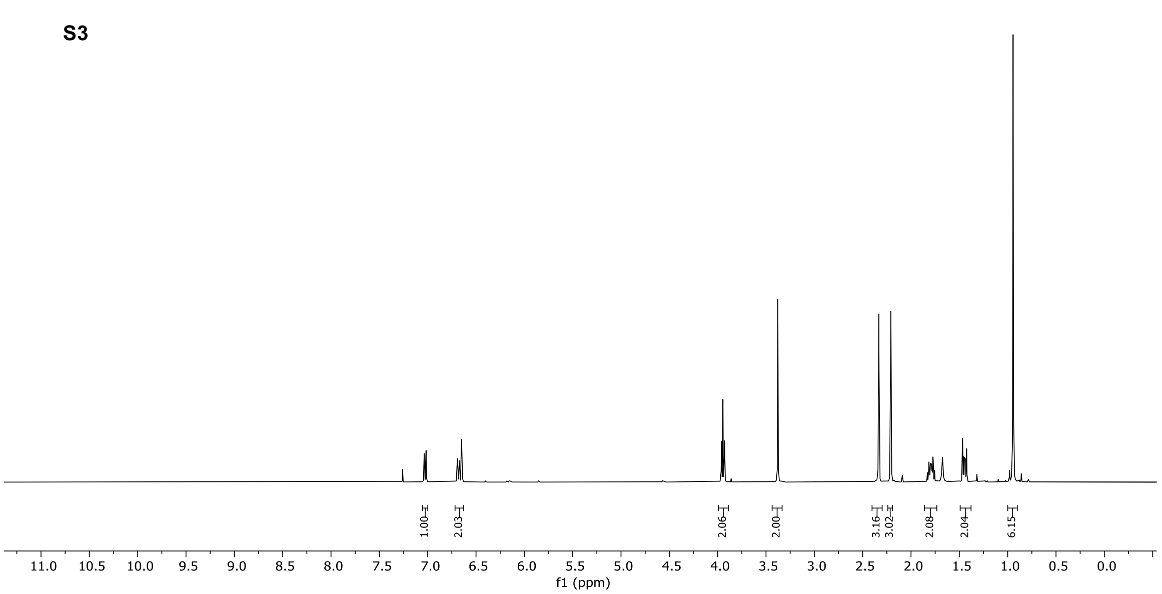

^{13}C NMR (126 MHz, $CDCl_3$) of **S3**

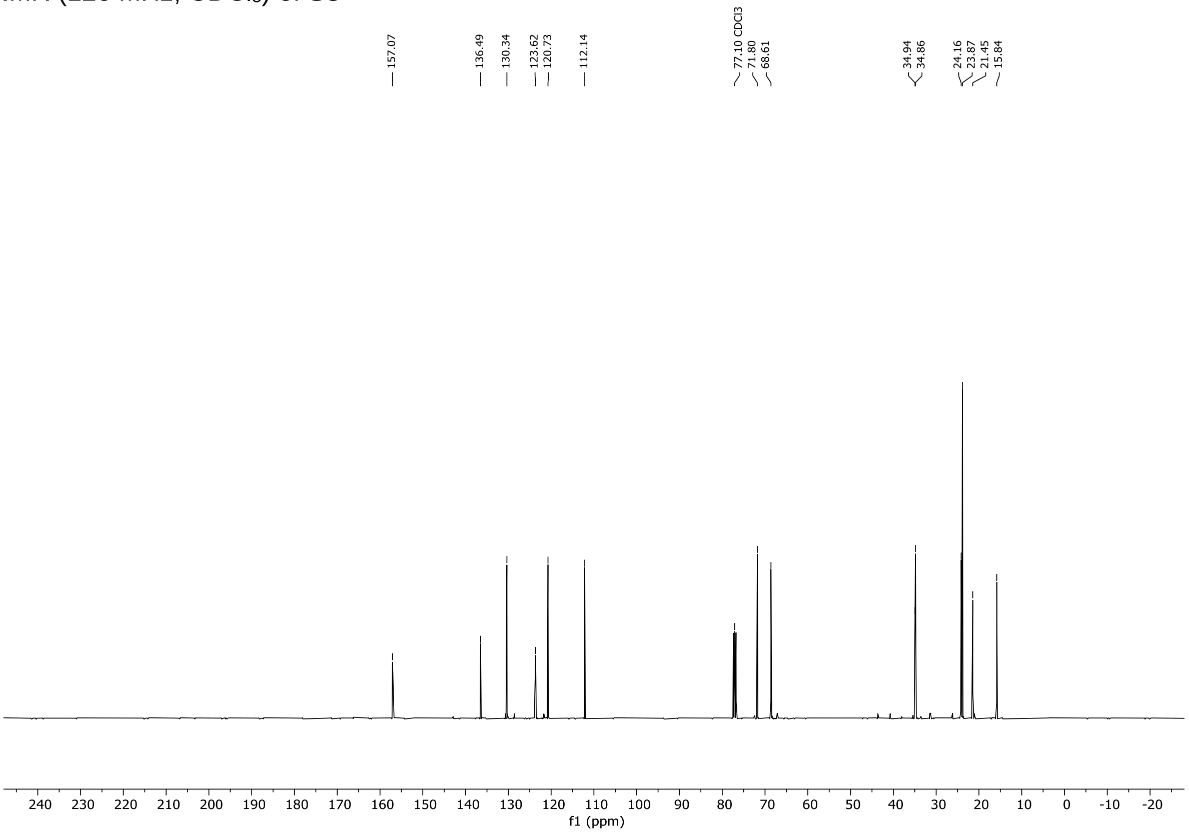

^{1}H NMR (400 MHz, $CDCl_3$) of **S4** (see procedure)

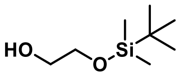

S4

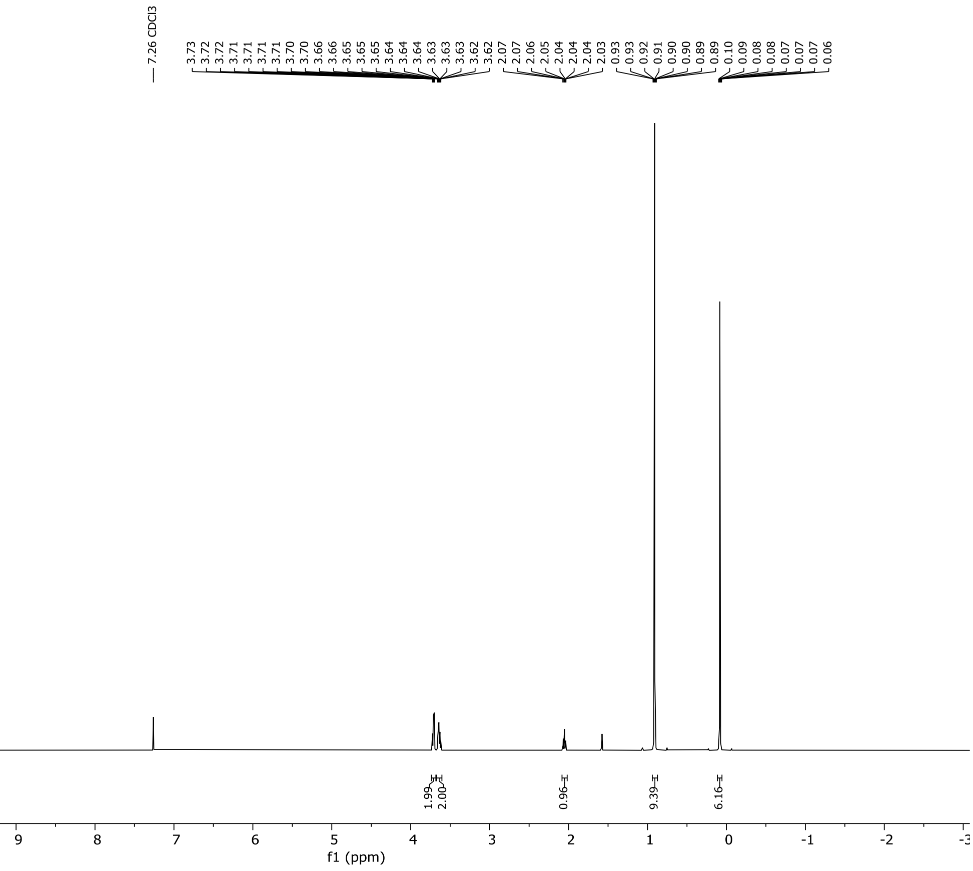

^{1}H NMR (400 MHz, $CDCl_3$) of **S5** (*see procedure*)

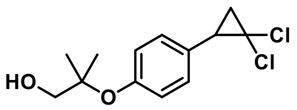

S5

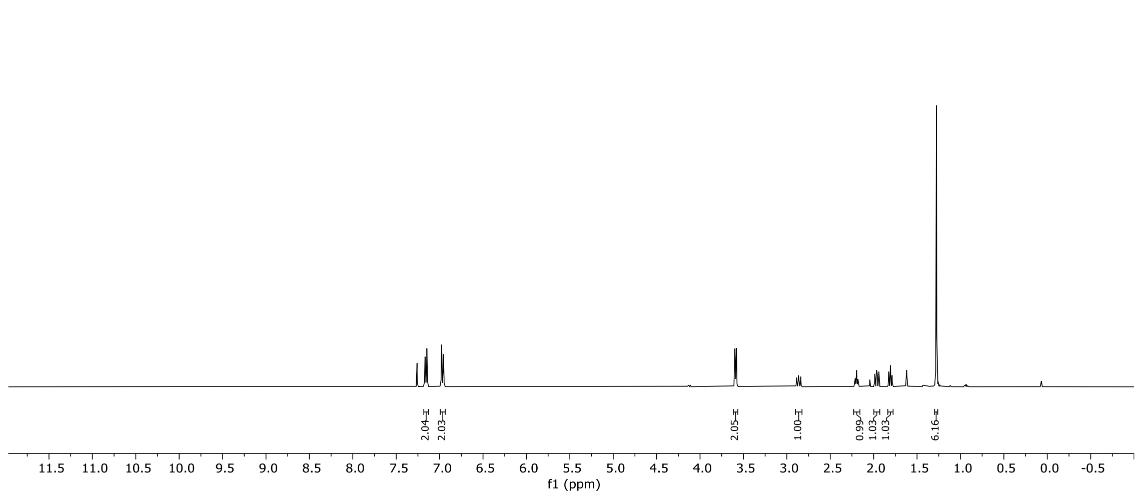

^{13}C NMR (101 MHz, $CDCl_3$) of **S5**

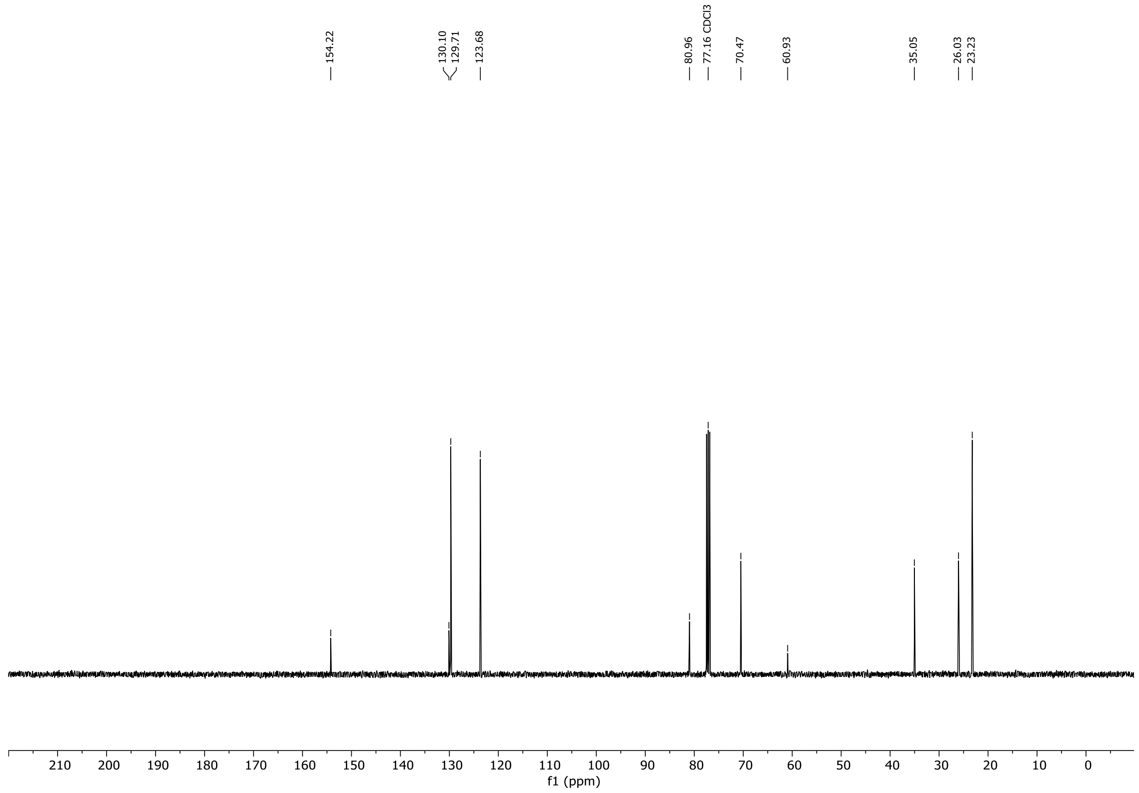

^{1}H NMR (400 MHz, $CDCl_3$) of **2c** (see procedure)

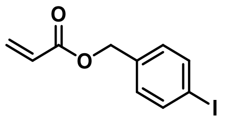

2c

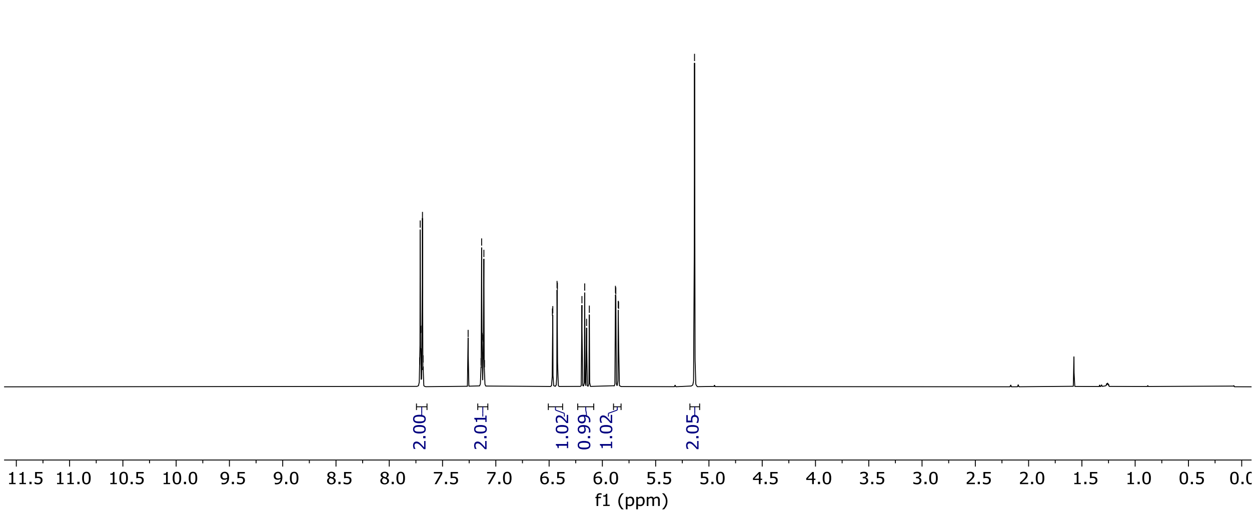

^{1}H NMR (400 MHz, $CDCl_3$) of **2d** (see procedure)

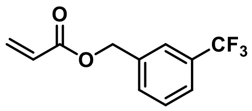

2d

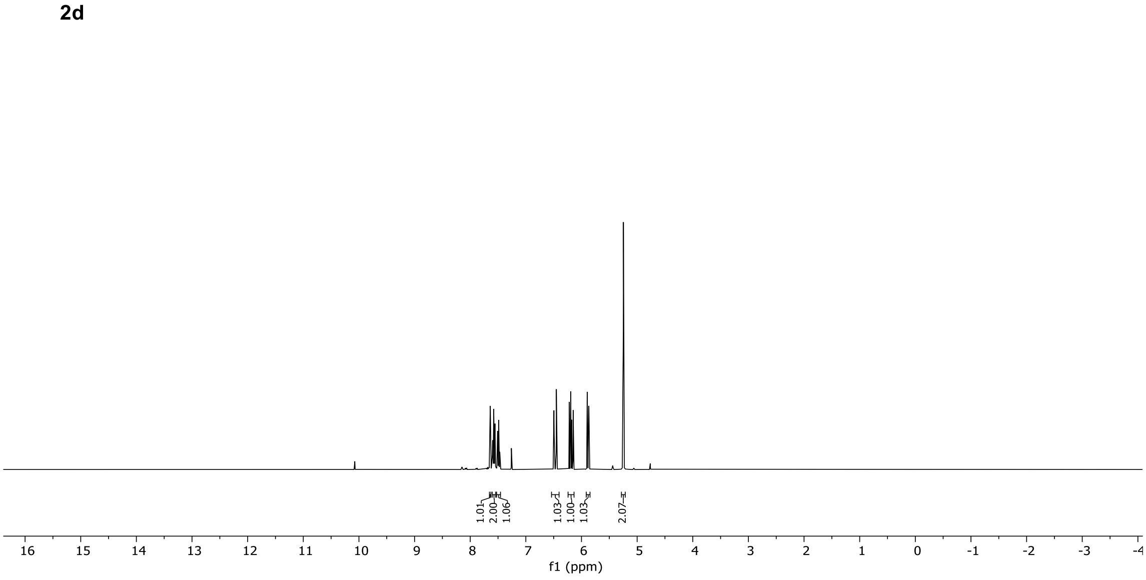

^{13}C NMR (126 MHz, $CDCl_3$) of **2d**

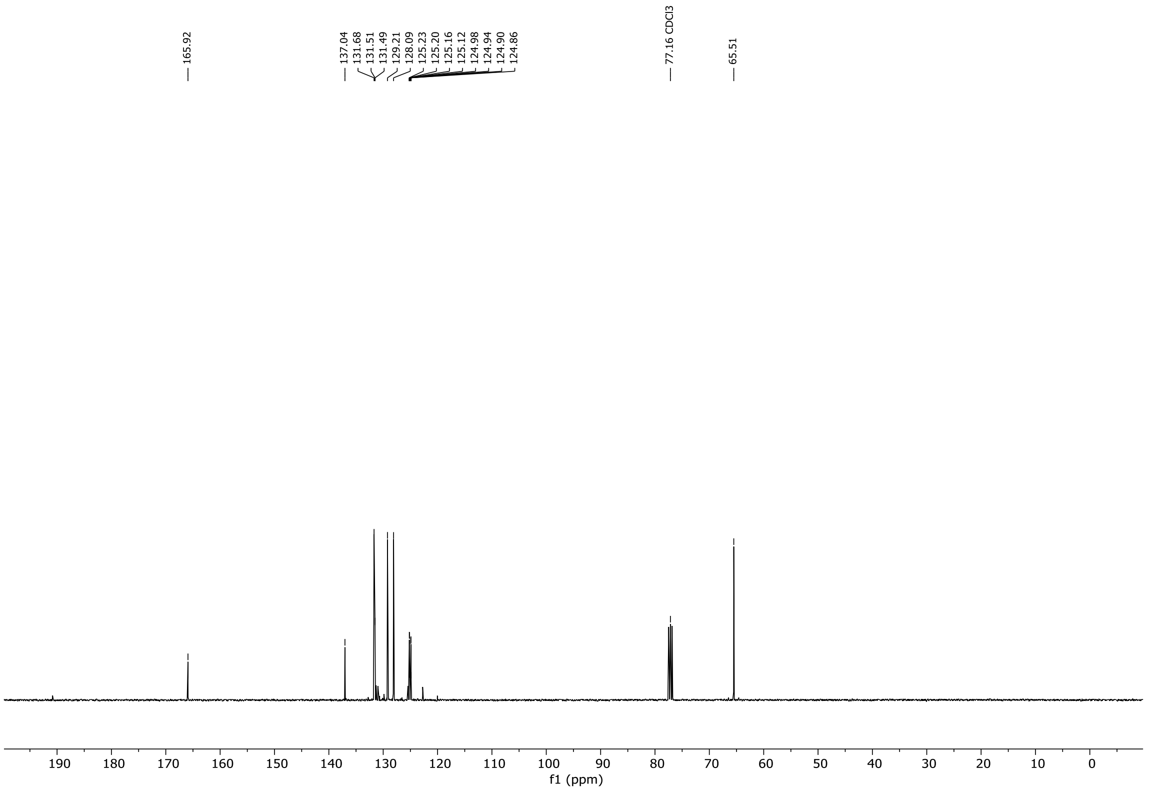

^{19}F NMR (376 MHz, $CDCl_3$) of **2d**

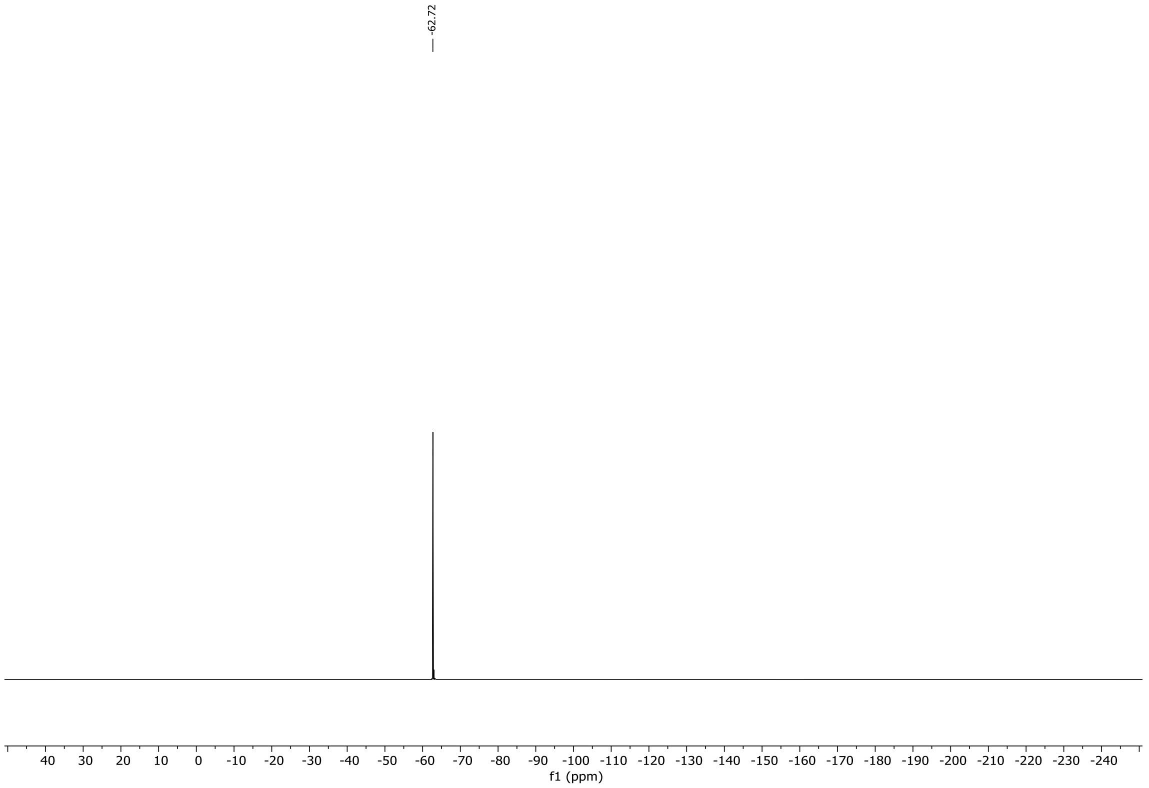

^{1}H NMR (400 MHz, $CDCl_3$) of **2e** (see procedure)

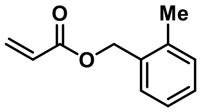

2e

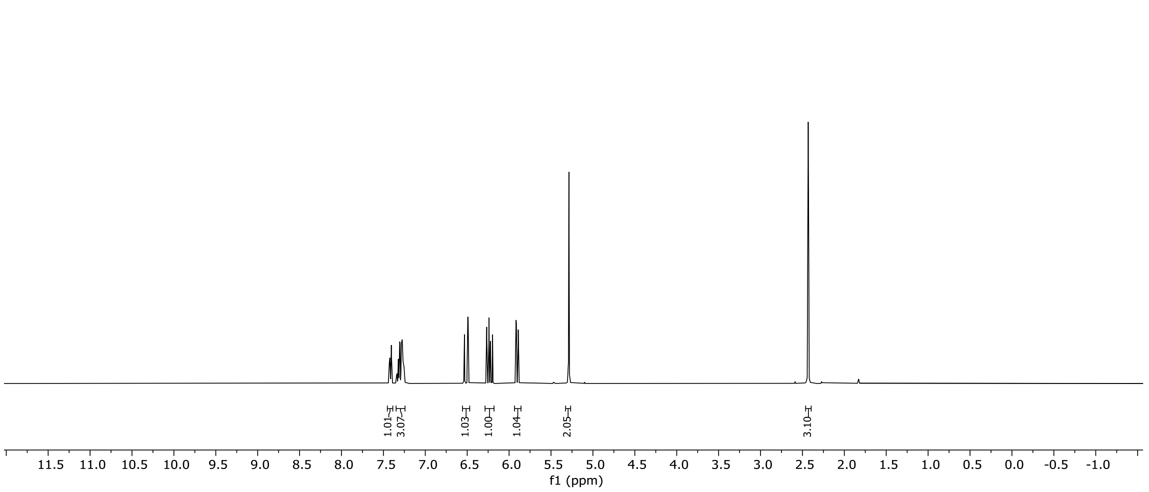

^{13}C NMR (126 MHz, $CDCl_3$) of **2e**

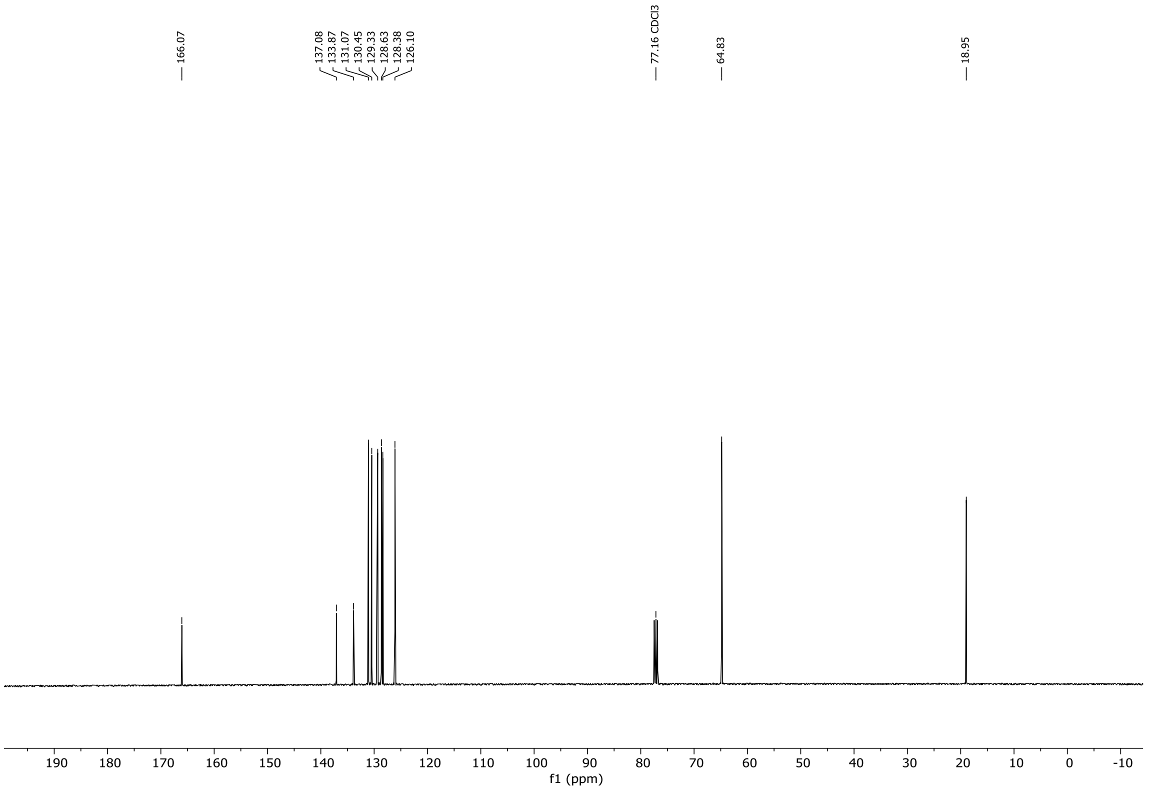

^{1}H NMR (400 MHz, $CDCl_3$) of **2f** (*see procedure*)

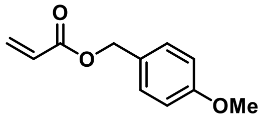

2f

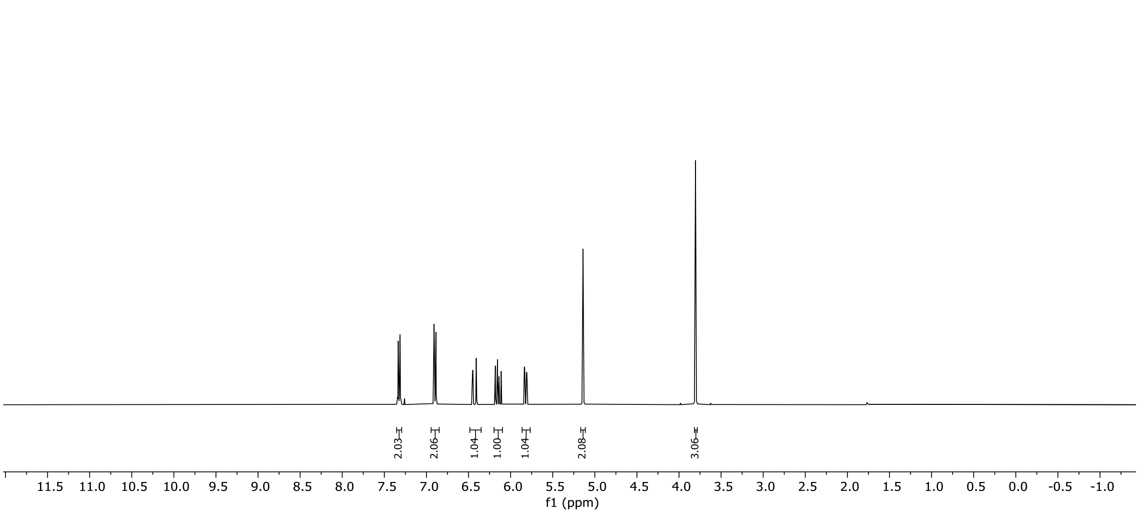

^{13}C NMR (126 MHz, $CDCl_3$) of **2f**

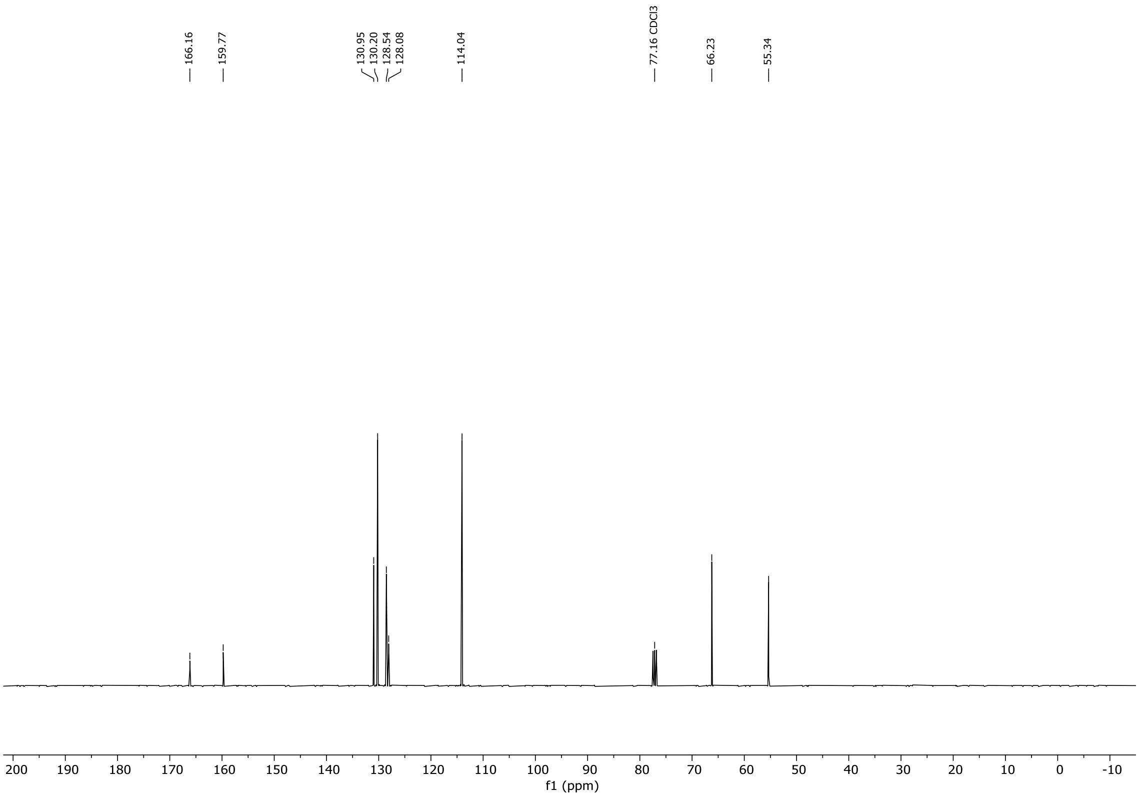

^{1}H NMR (400 MHz, $CDCl_3$) of **2k** (*see procedure*)

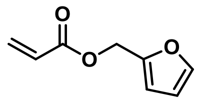

2k

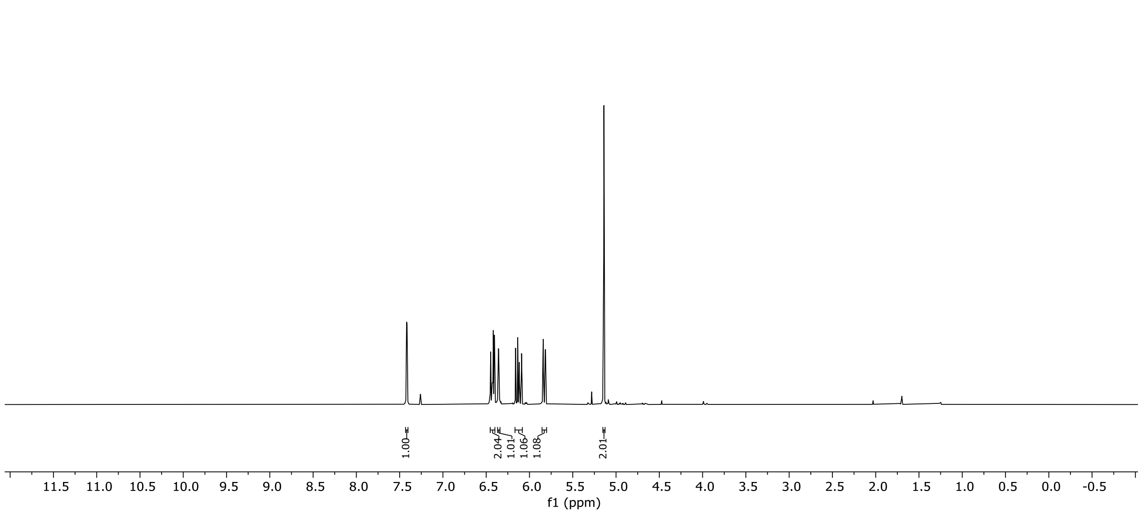

^{13}C NMR (126 MHz, $CDCl_3$) of **2k**

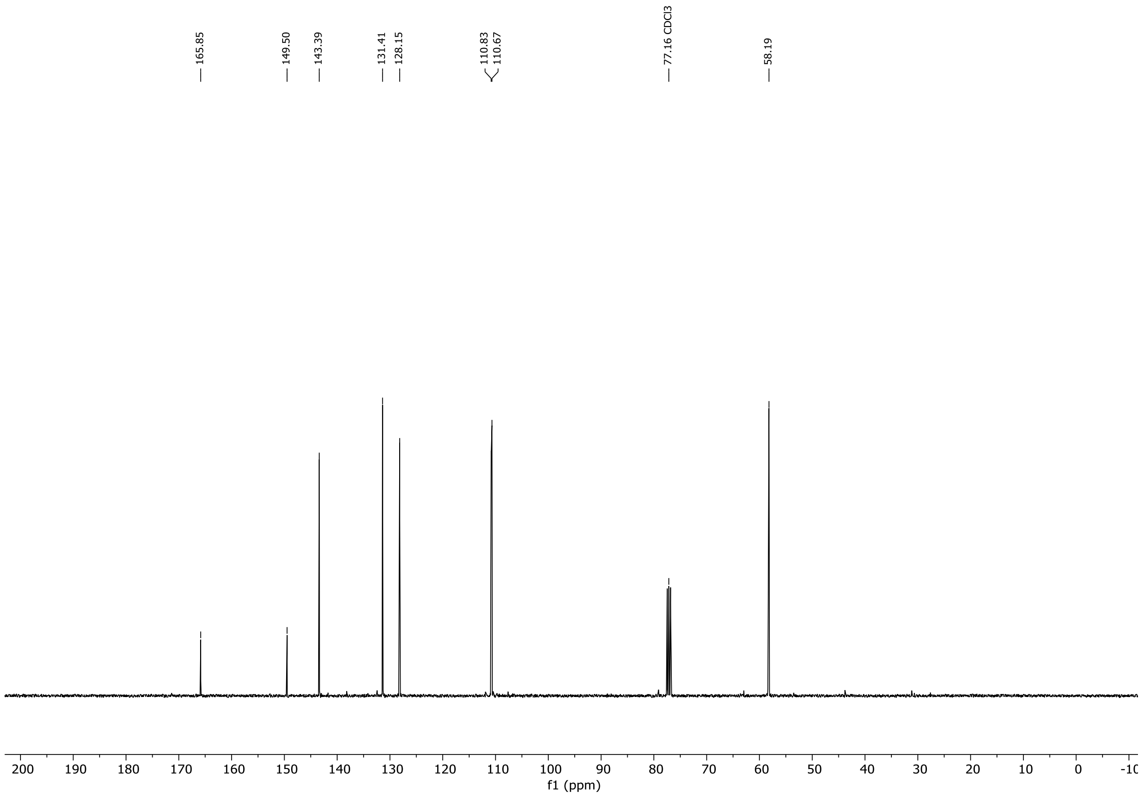

^{1}H NMR (400 MHz, $CDCl_3$) of **2l** (*see procedure*)

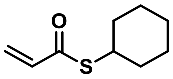

2l

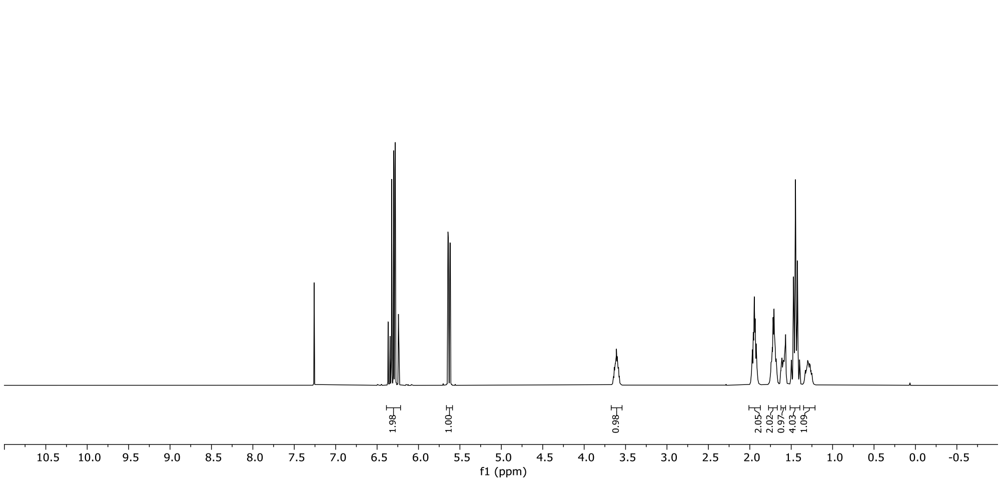

^{13}C NMR (126 MHz, $CDCl_3$) of **2l**

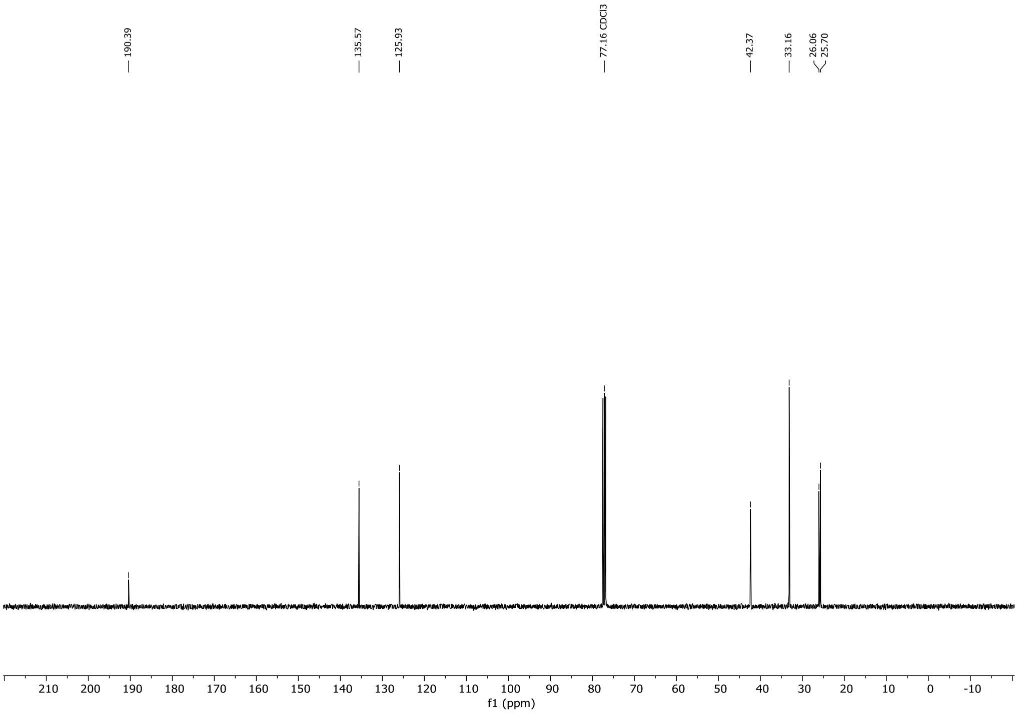

^{1}H NMR (400 MHz, $CDCl_3$) of **2m** (*see procedure*)

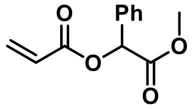

2m

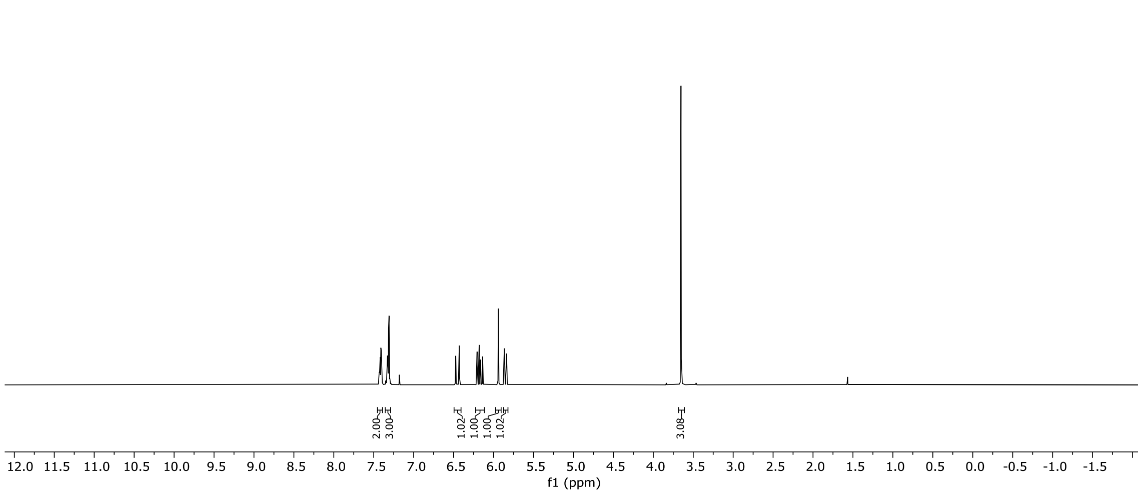

^{13}C NMR (126 MHz, $CDCl_3$) of **2m**

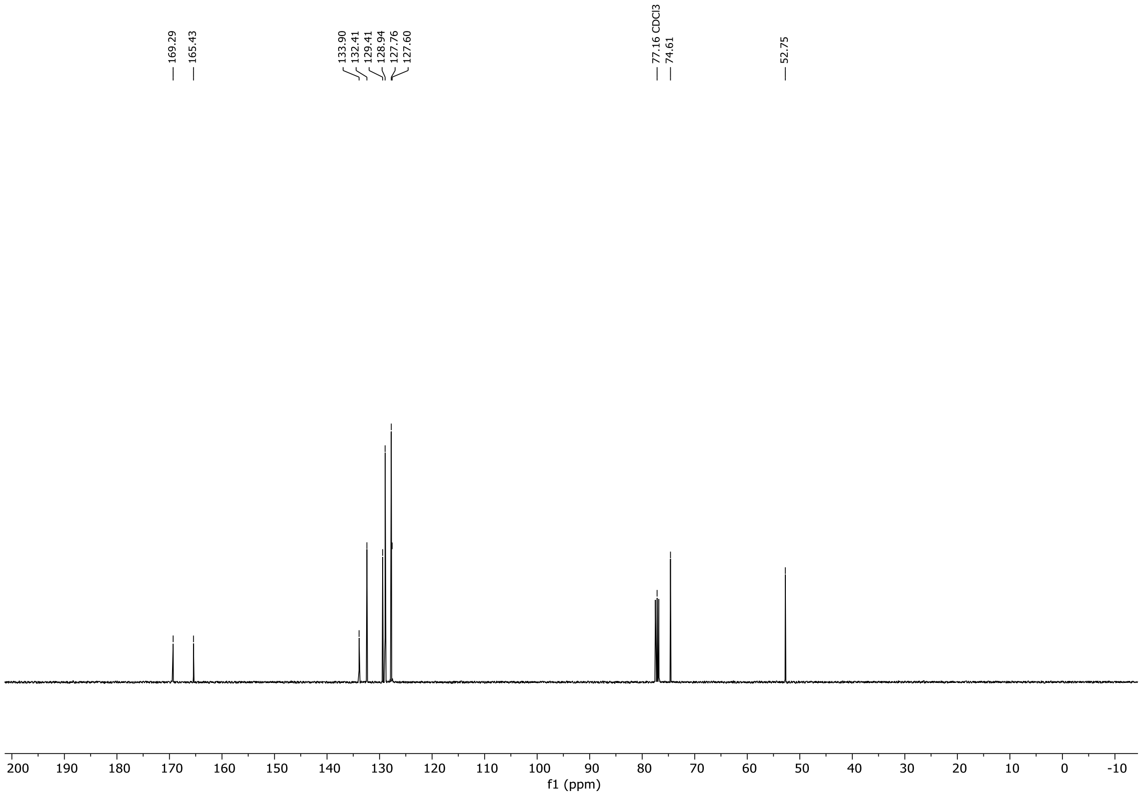

^{1}H NMR (400 MHz, $CDCl_3$) of **2n** (see procedure)

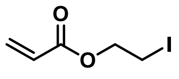

2n

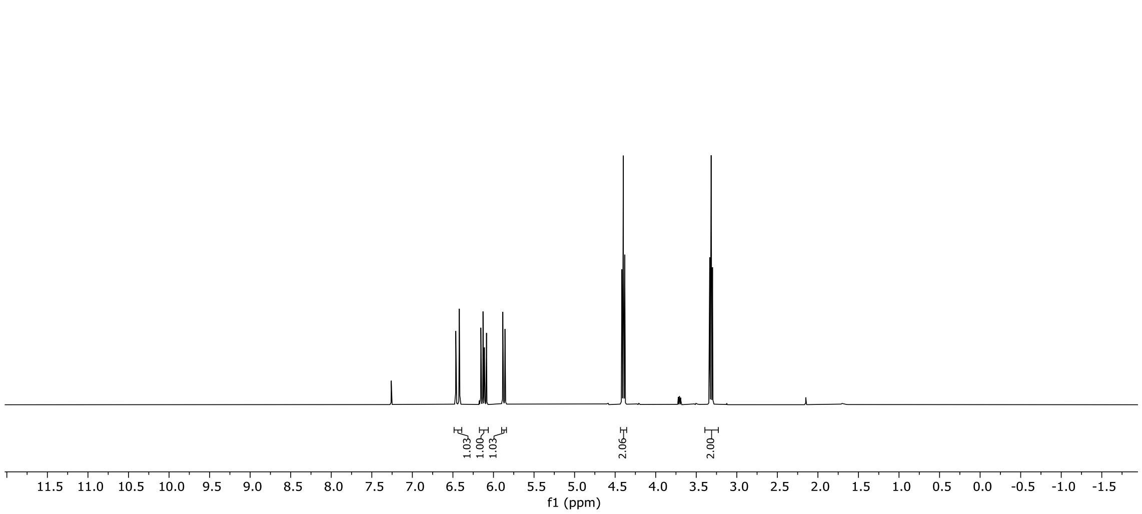

^{13}C NMR (126 MHz, $CDCl_3$) of **2n**

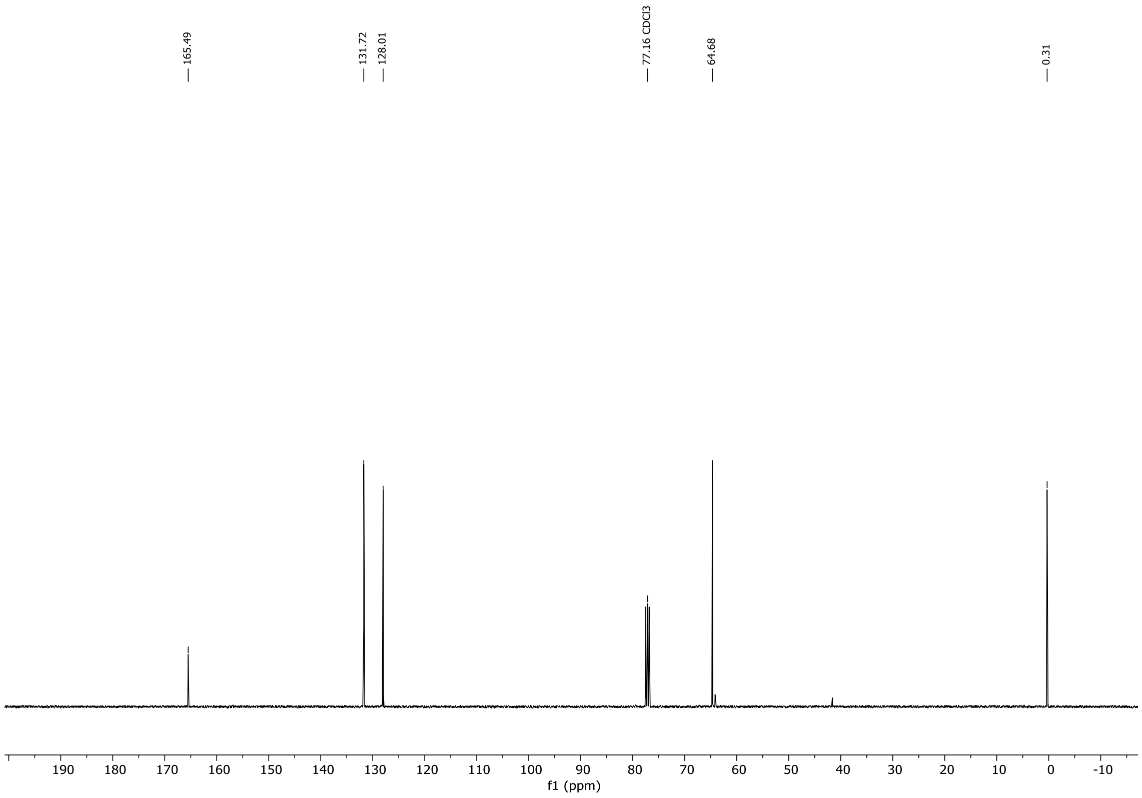

^{1}H NMR (400 MHz, $CDCl_3$) of **2o** (*see procedure*)

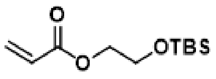

2o

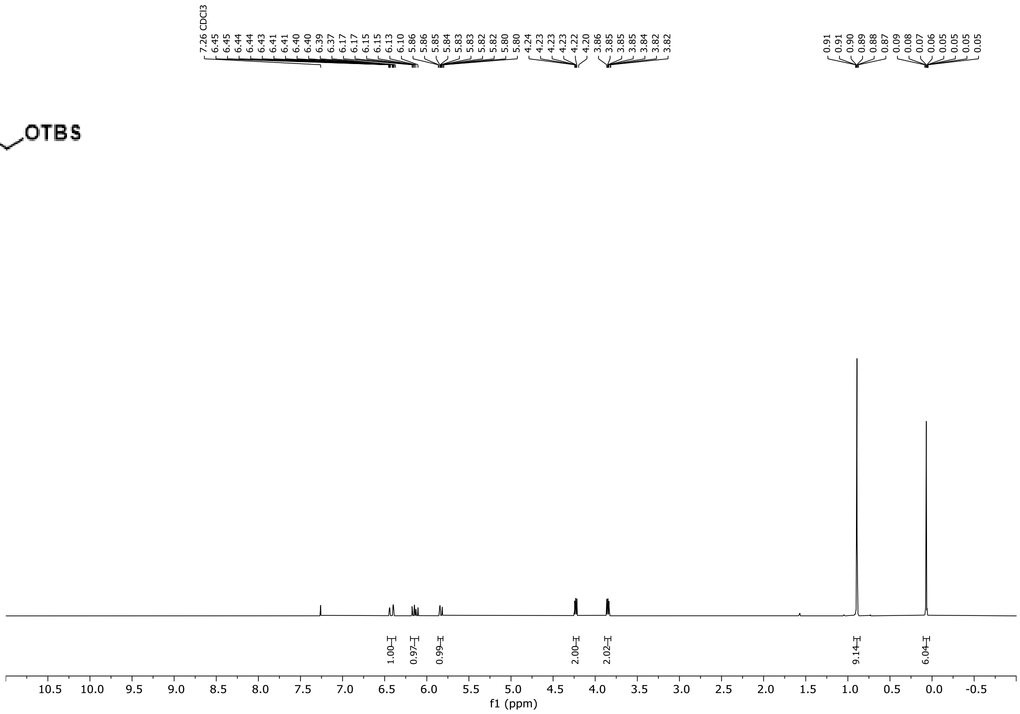

^{13}C NMR (126 MHz, $CDCl_3$) of **2o**

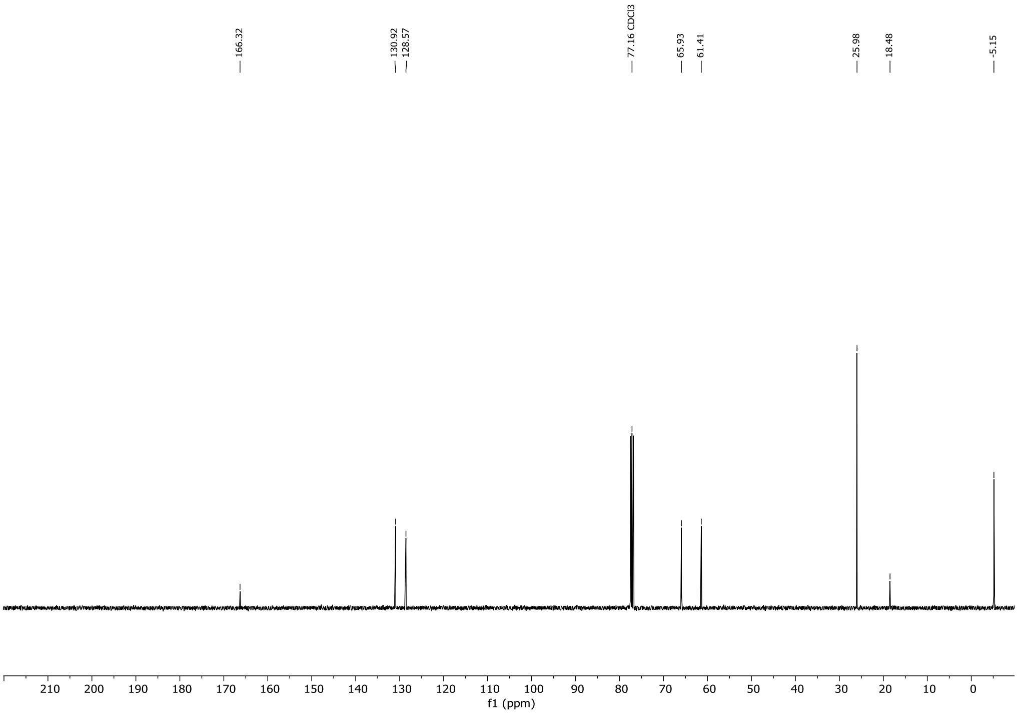

^{1}H NMR (400 MHz, $CDCl_3$) of **2p** (*see procedure*)

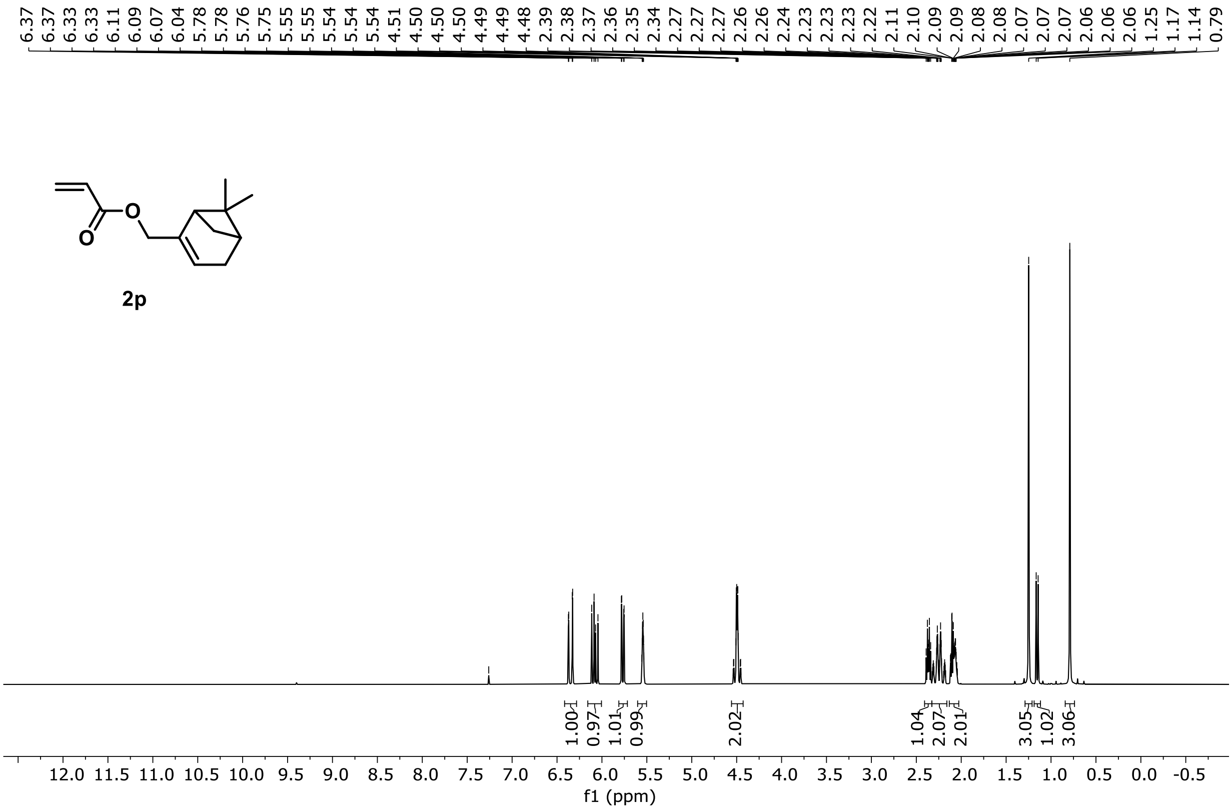

^{13}C NMR (101 MHz, $CDCl_3$) of **2p**

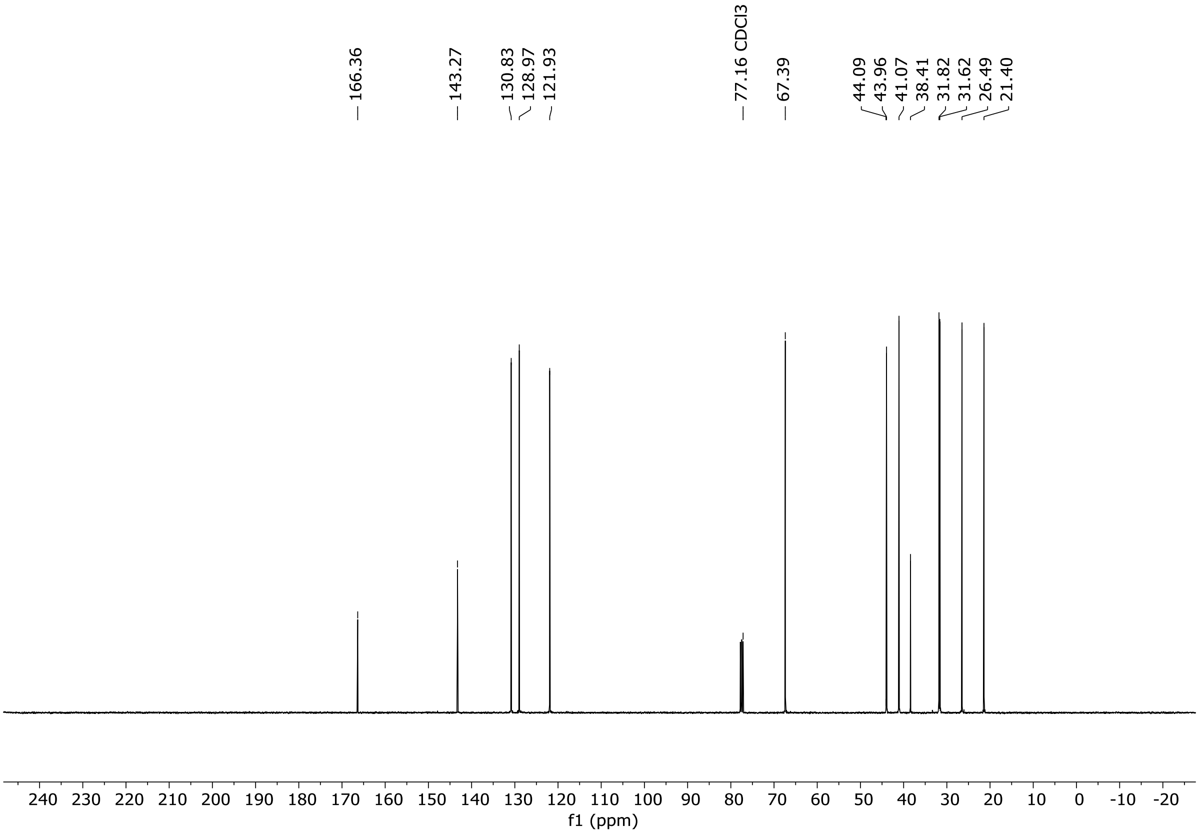

^{1}H NMR (400 MHz, $CDCl_3$) of **2t** (*see procedure*)

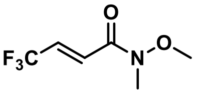

2t

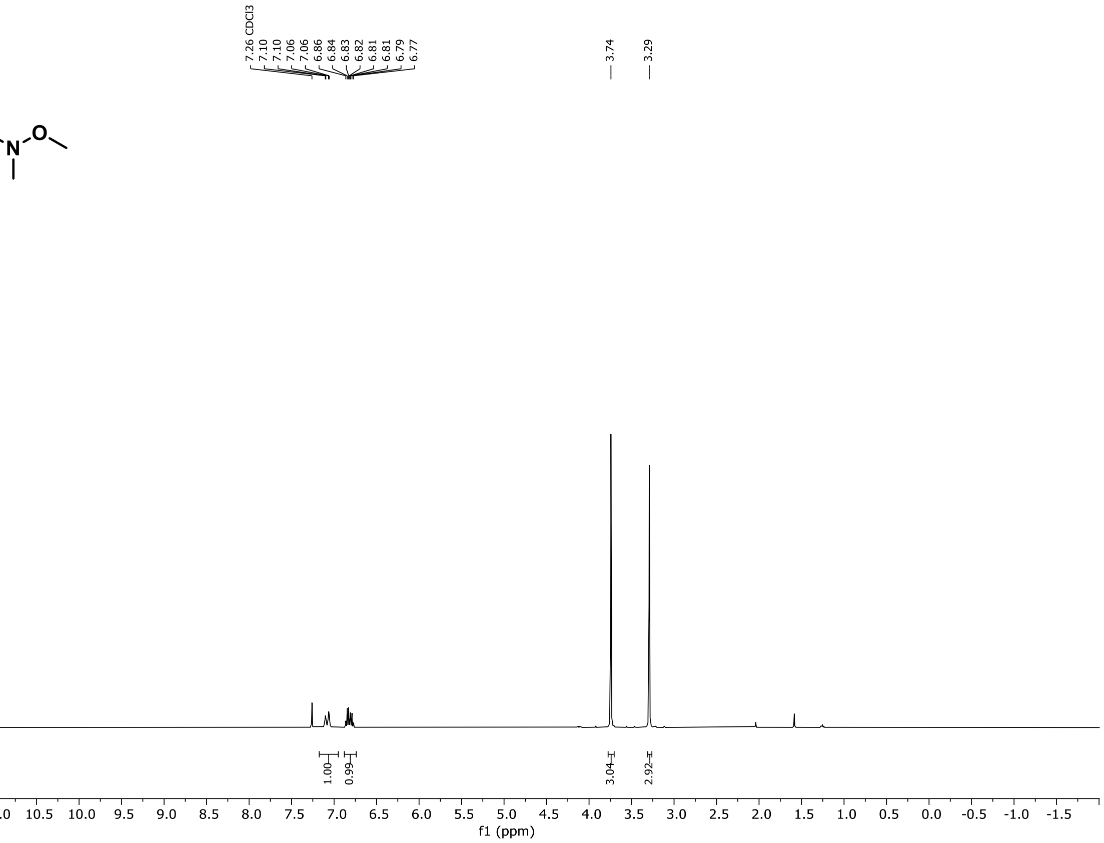

^{13}C NMR (151 MHz, $CDCl_3$) of **2t**

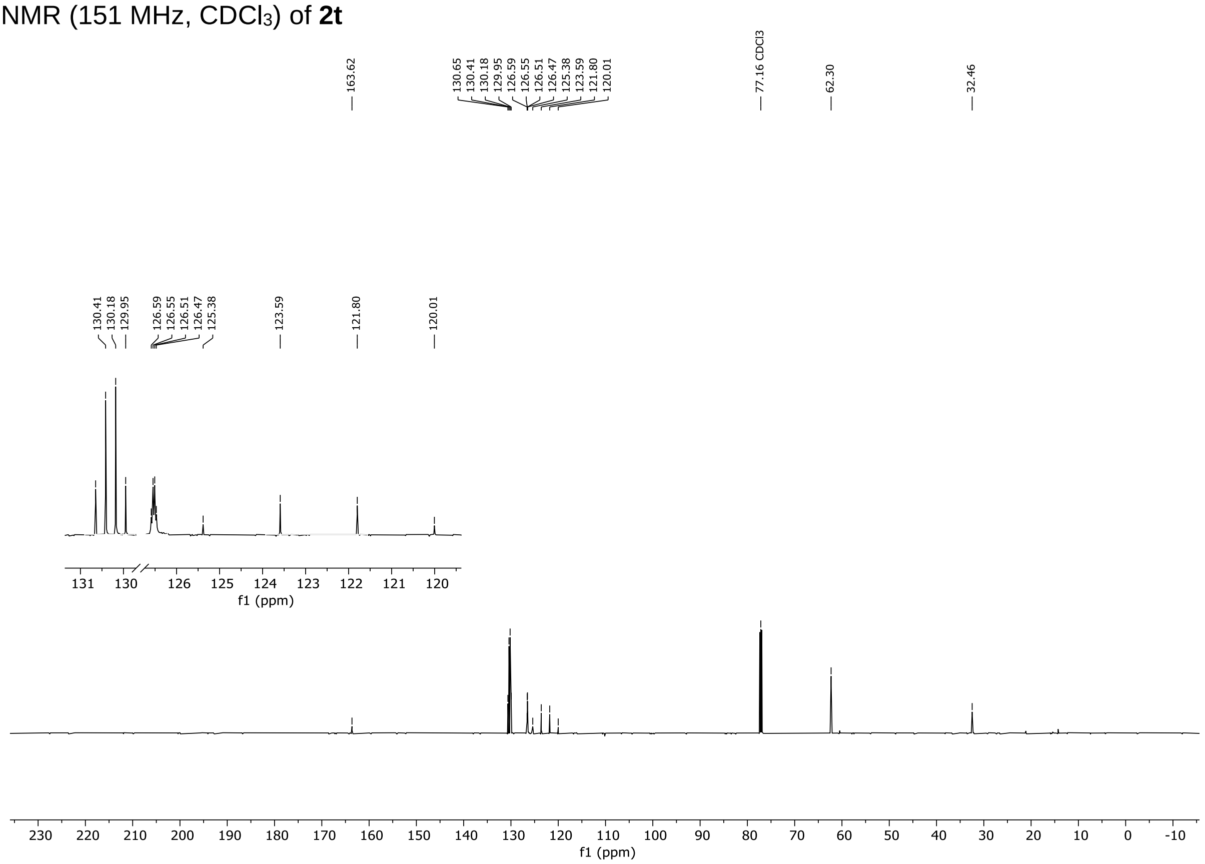

^{13}C NMR {^{1}H, ^{19}F} (126 MHz, $CDCl_3$) of **2t**

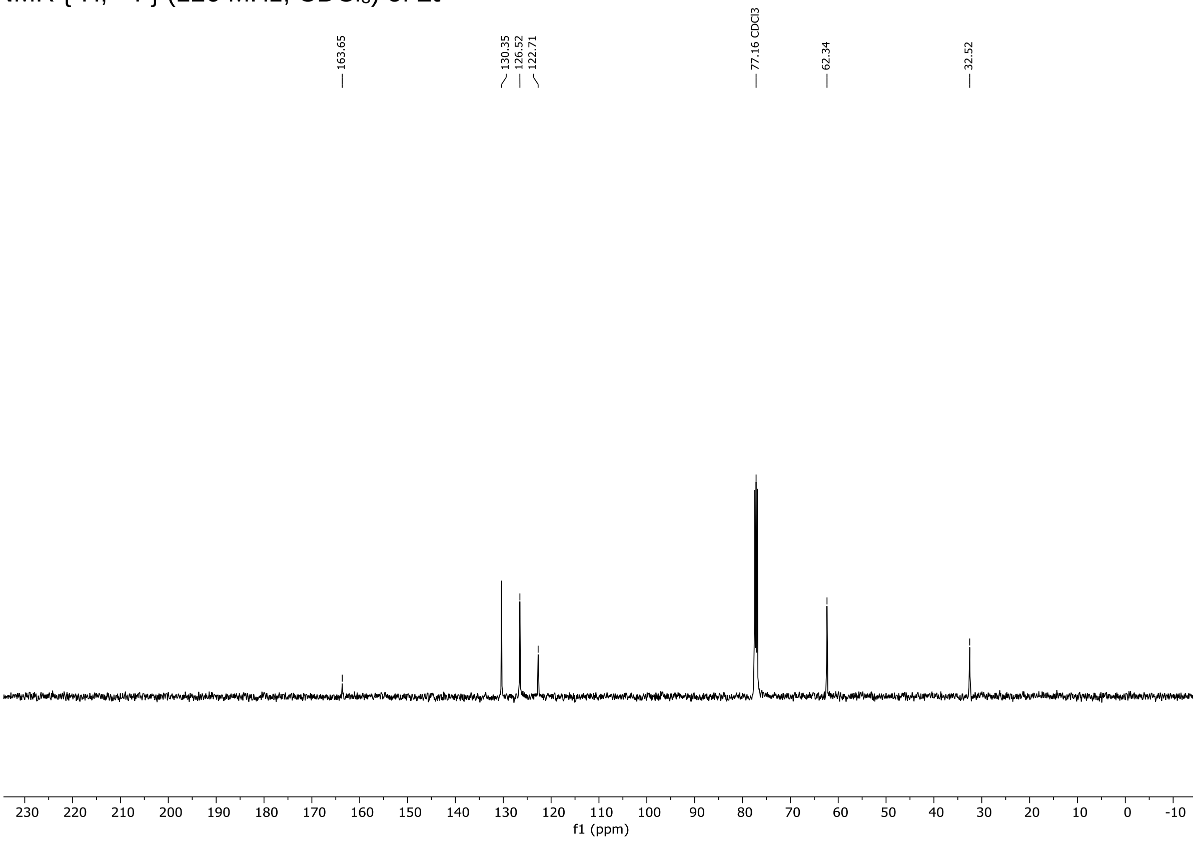

^{19}F NMR (377 MHz, $CDCl_3$) of **2t**

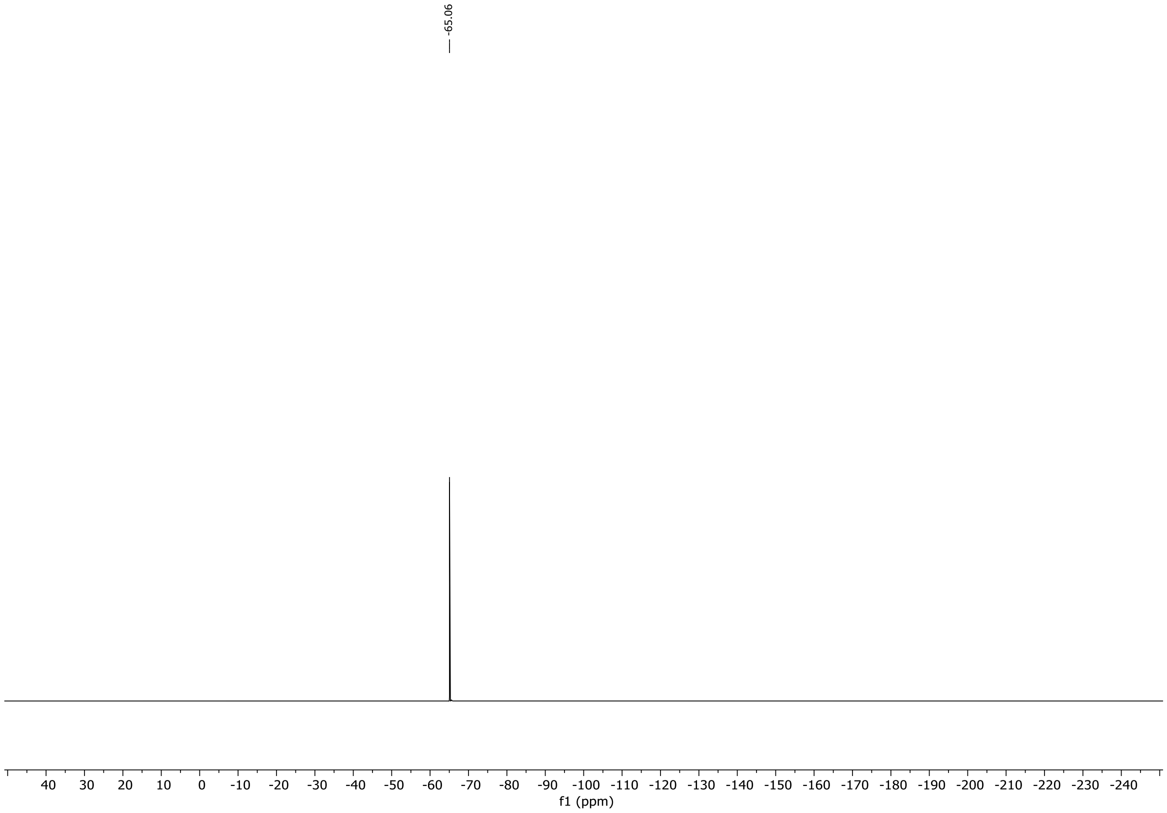

^{1}H NMR (500 MHz, $CDCl_3$) of **2u** (see procedure)

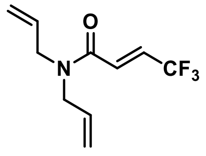

2u

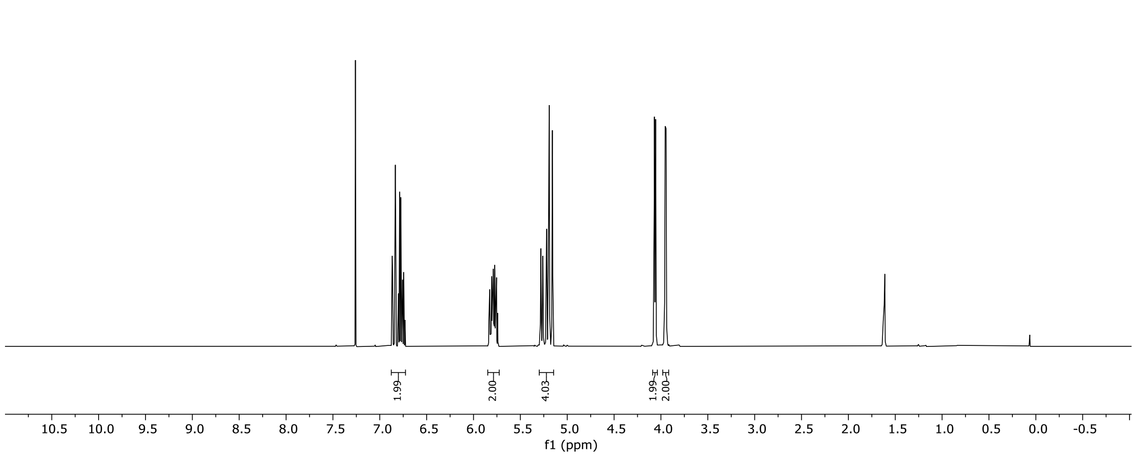

^{13}C NMR (126 MHz, $CDCl_3$) of **2u**

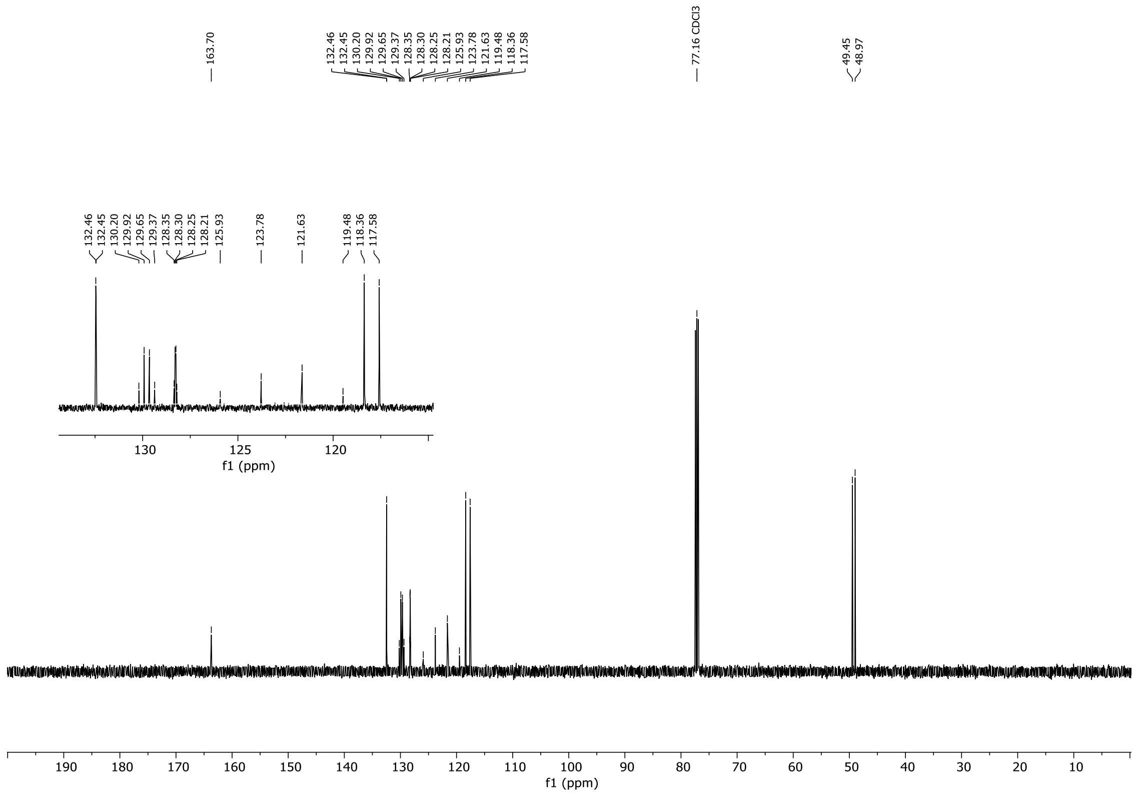

^{13}C NMR {^{1}H, ^{19}F} (126 MHz, $CDCl_3$) of **2u**

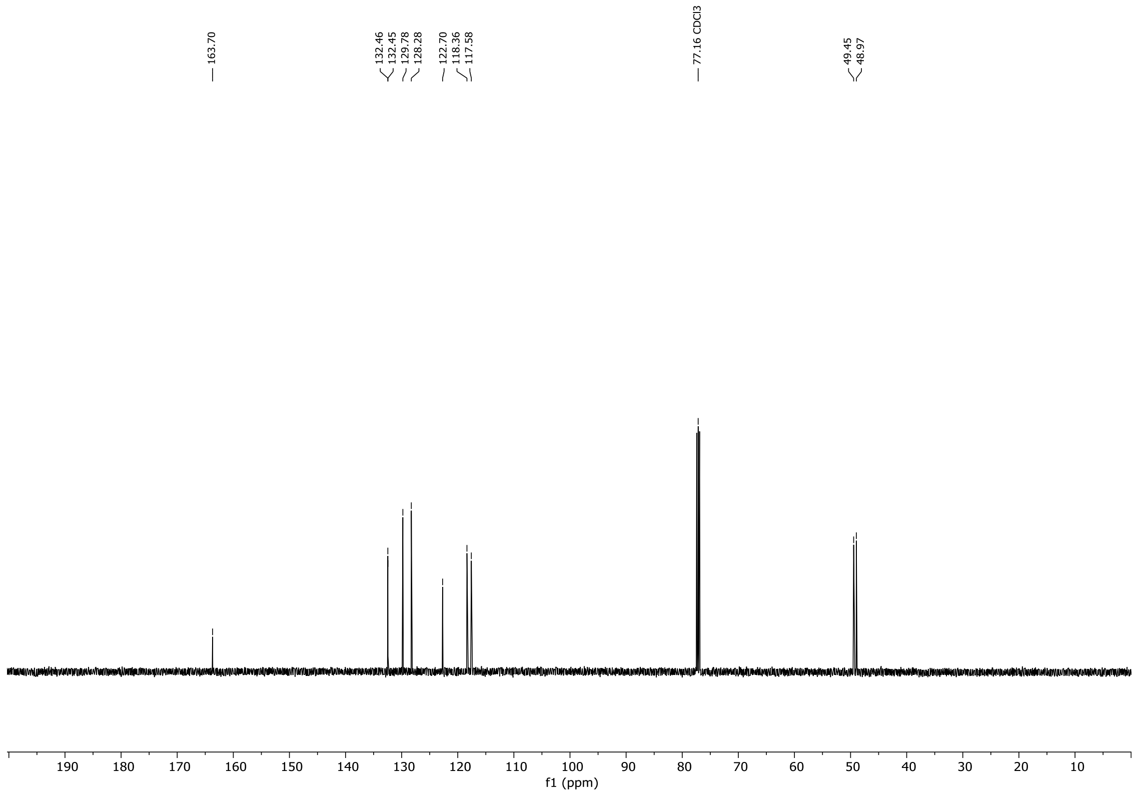

^{19}F NMR (377 MHz, $CDCl_3$) of **2u**

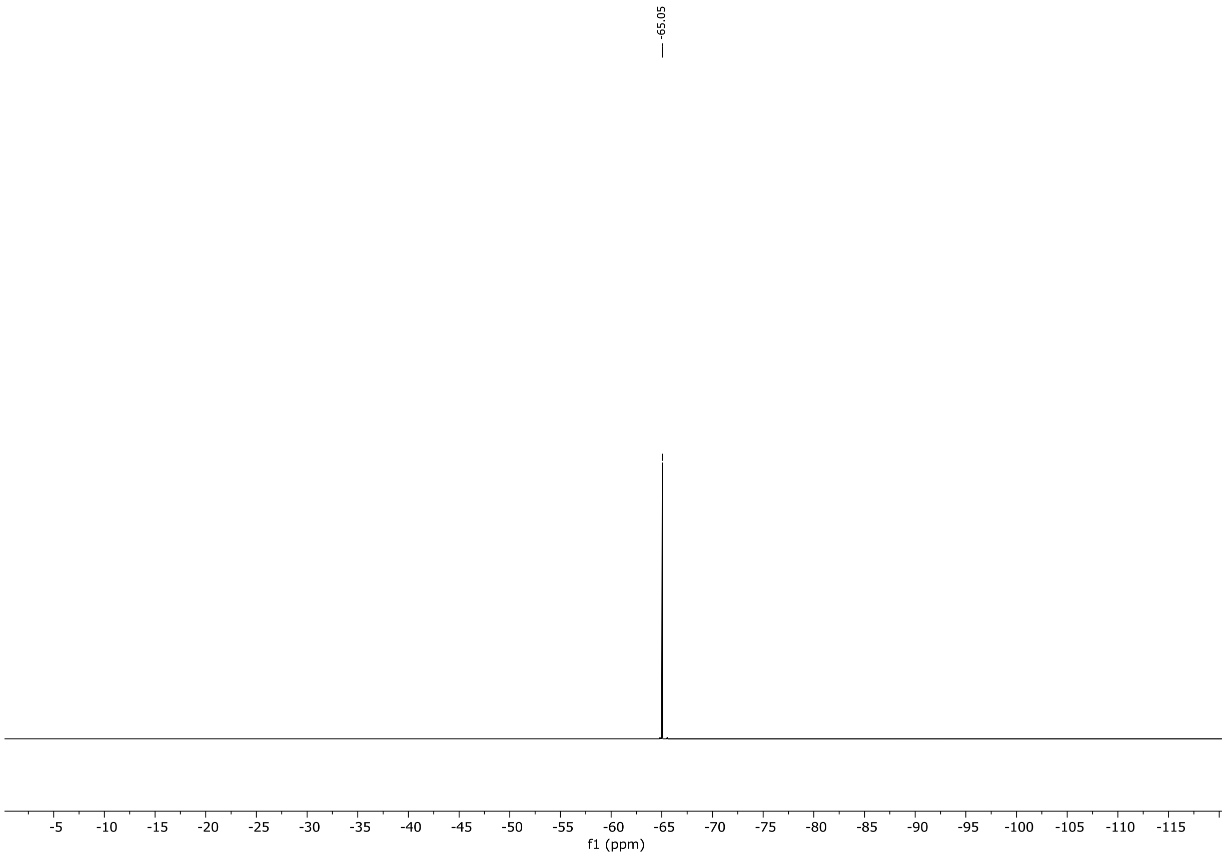

^{1}H NMR (400 MHz, $CDCl_3$) of **2ae** (*see procedure*)

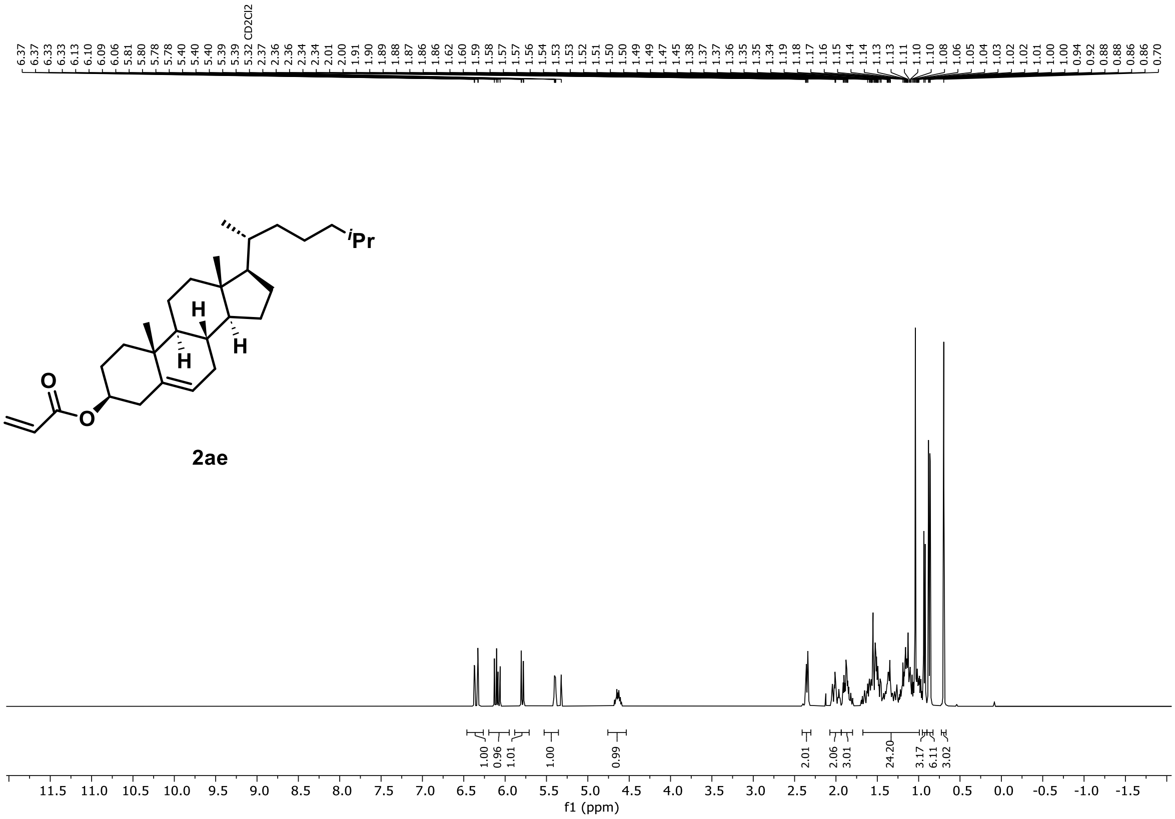

^{13}C NMR (126 MHz, $CDCl_3$) of **2ae**

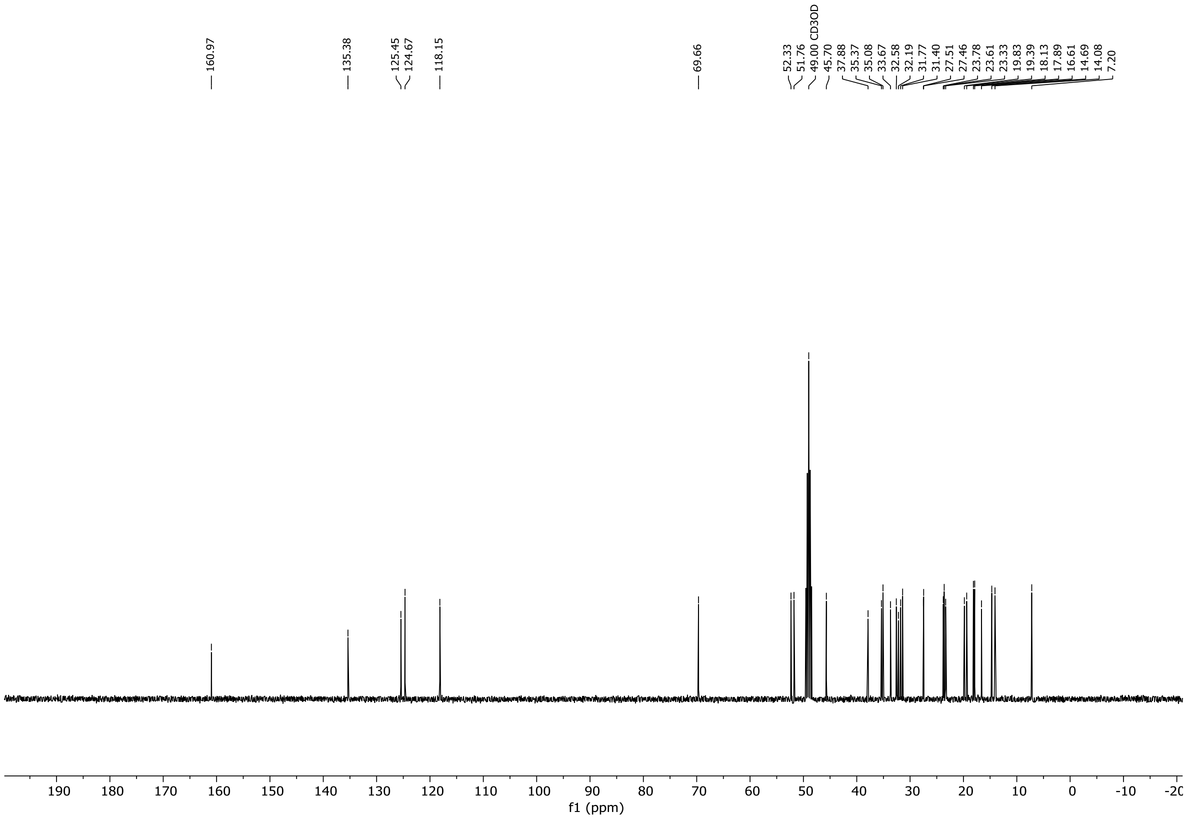

^{1}H NMR (400 MHz, $CDCl_3$) of **2af** (*see procedure*)

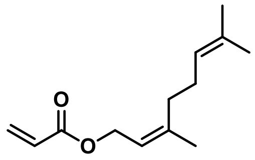

2af

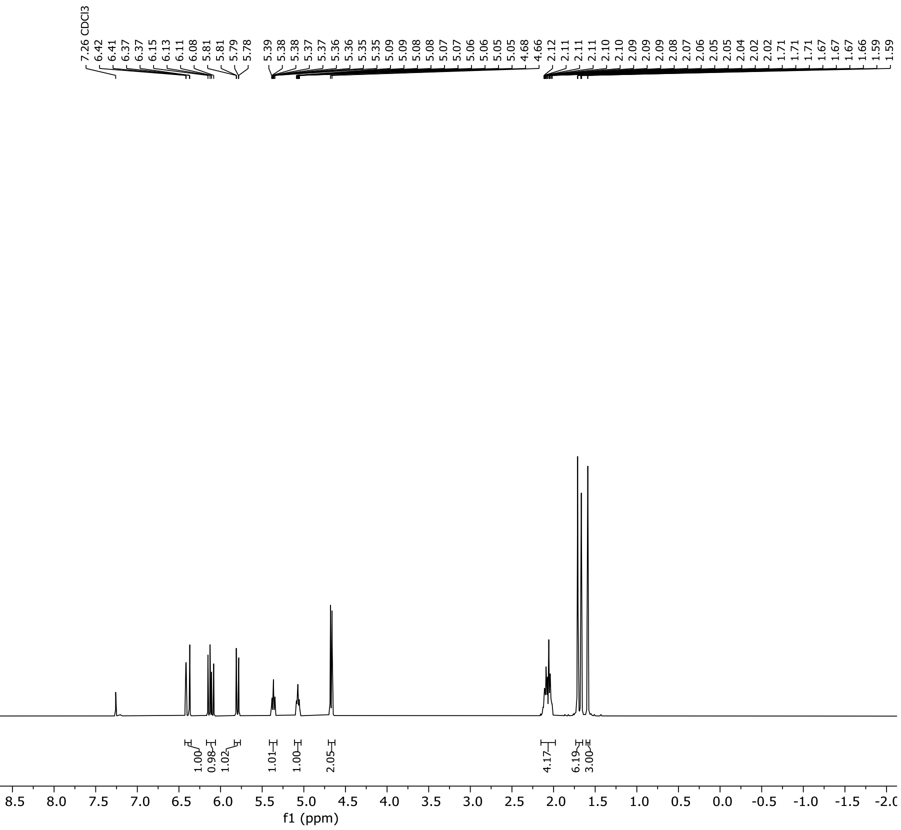

^{13}C NMR (126 MHz, $CDCl_3$) of **2af**

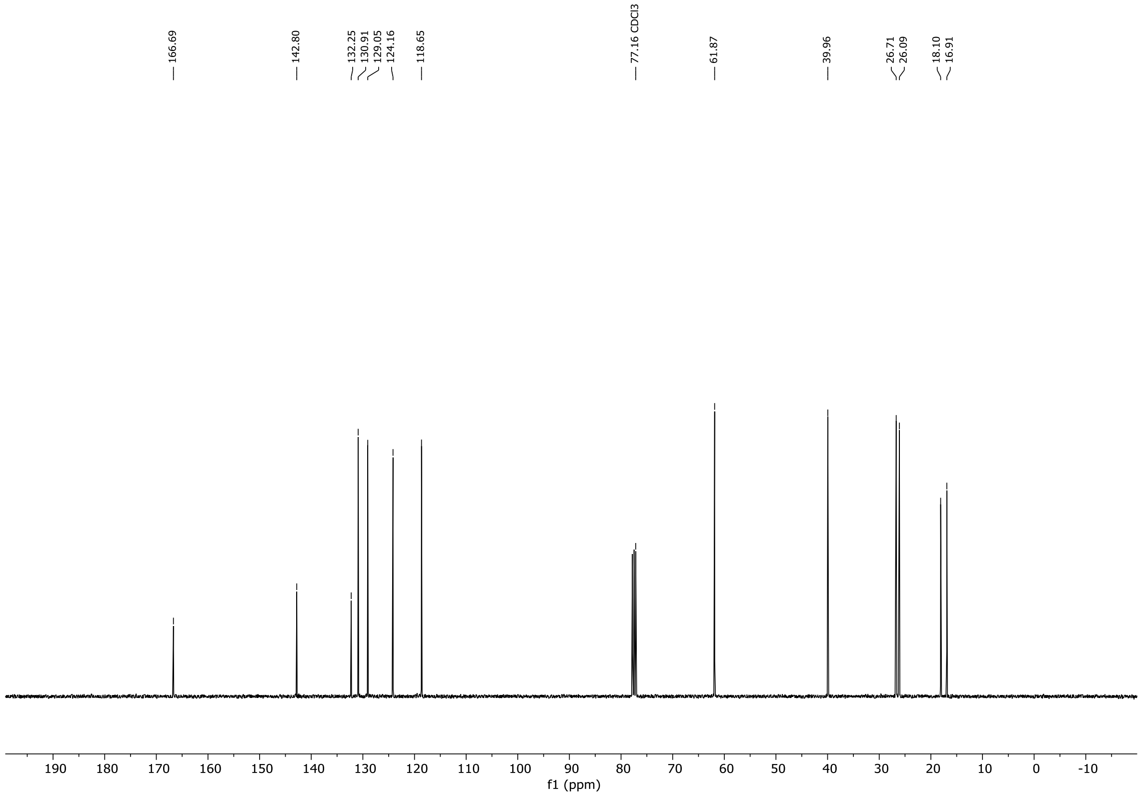

^{1}H NMR (599 MHz, $CDCl_3$) of **2ag** (see procedure)

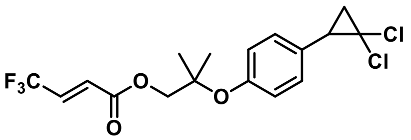

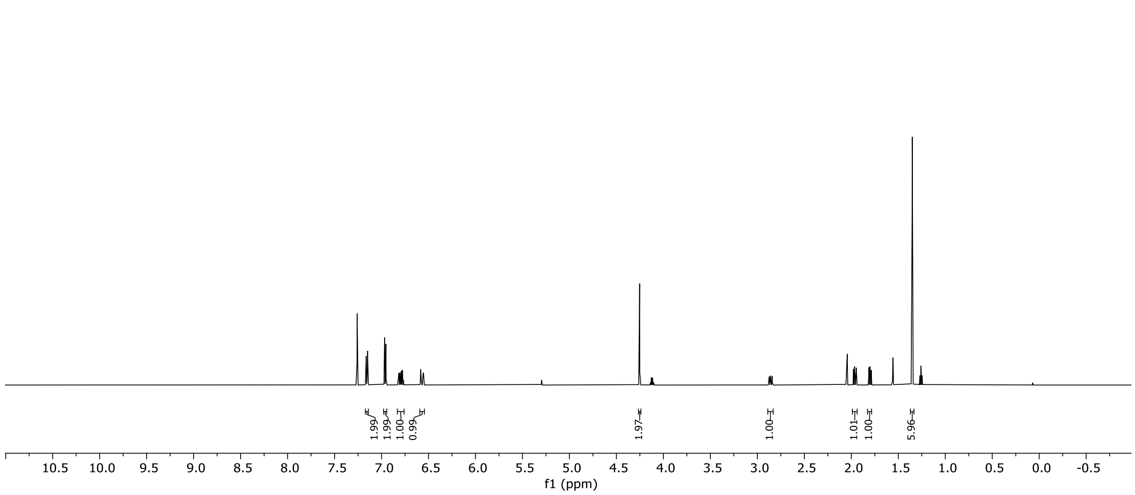

^{13}C NMR (151 MHz, $CDCl_3$) of **2ag**

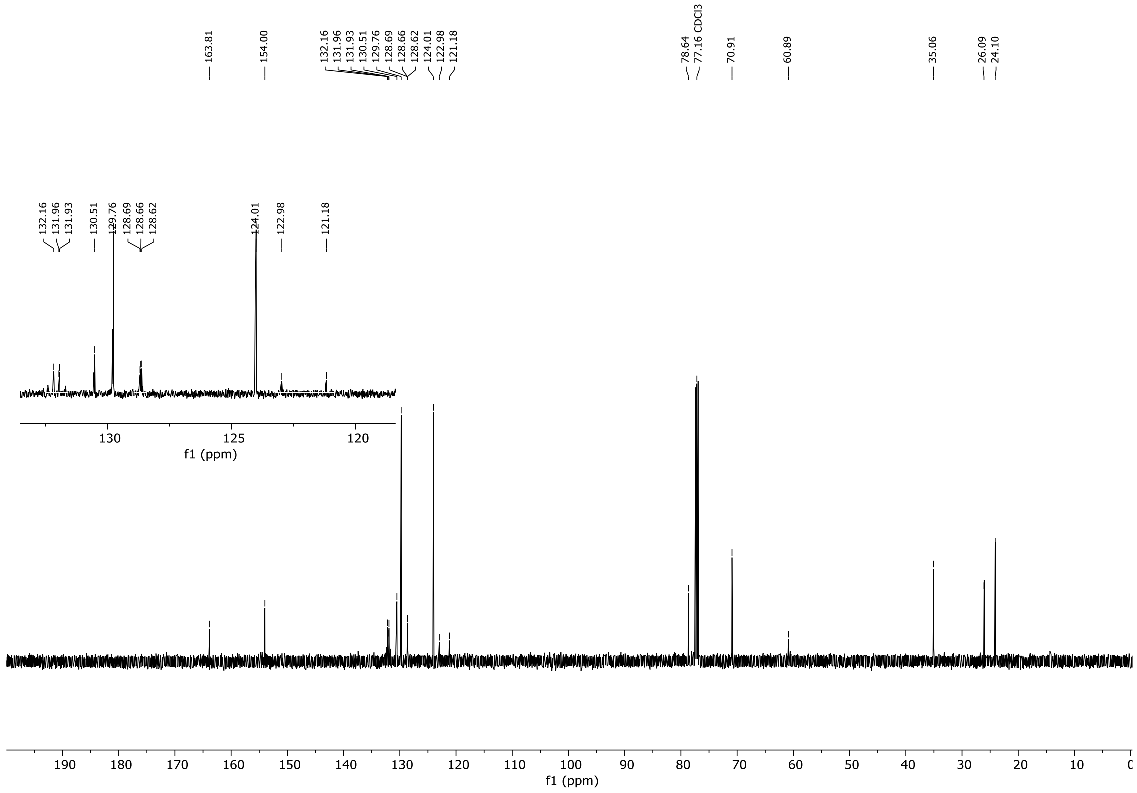

^{13}C NMR {^{1}H, ^{19}F} (151 MHz, $CDCl_3$) of **2ag**

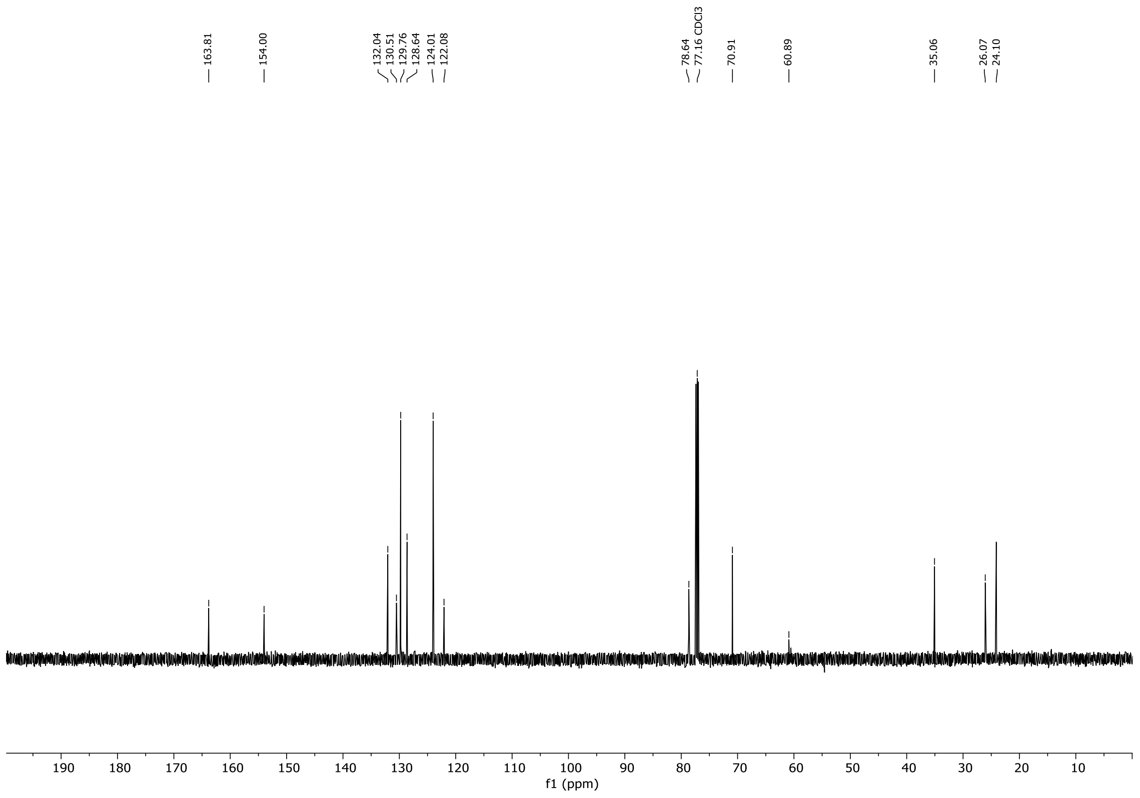

^{19}F NMR (377 MHz, $CDCl_3$) of **2ag**

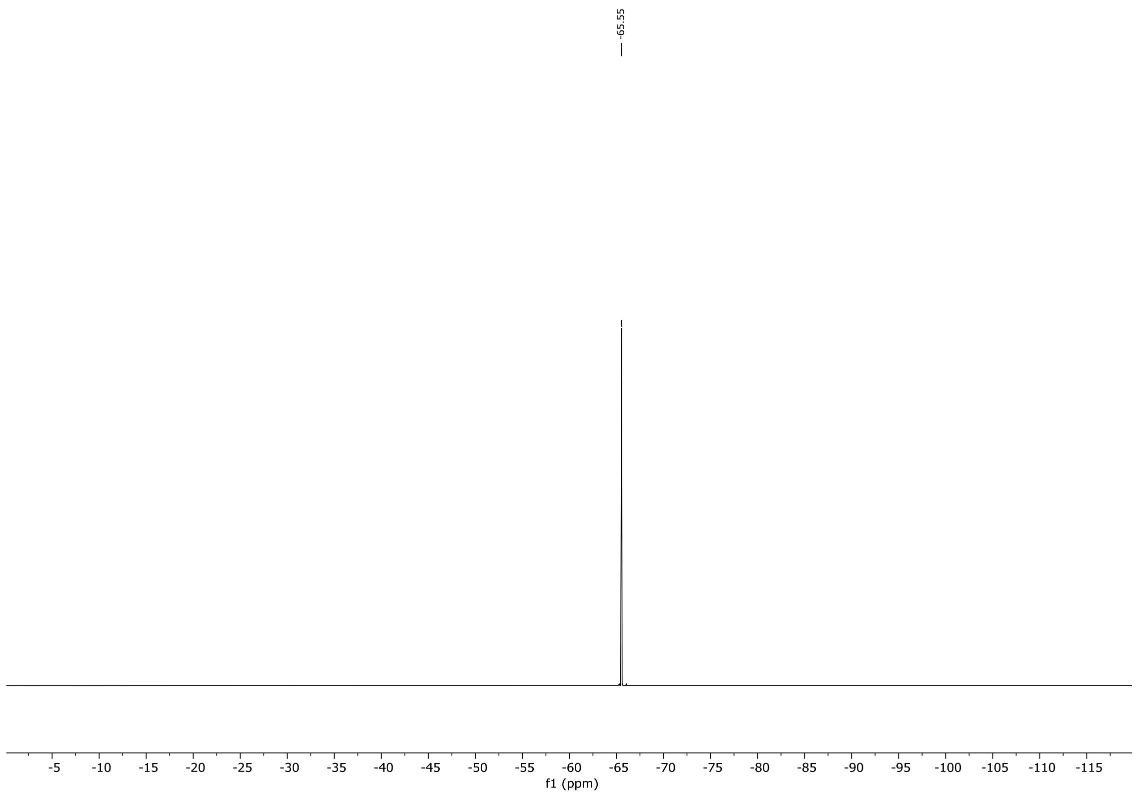

^{1}H NMR (400 MHz, CDCl$_3$) of **2ah** (see procedure)

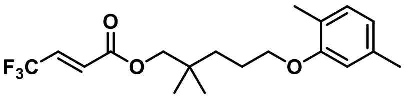

2ah

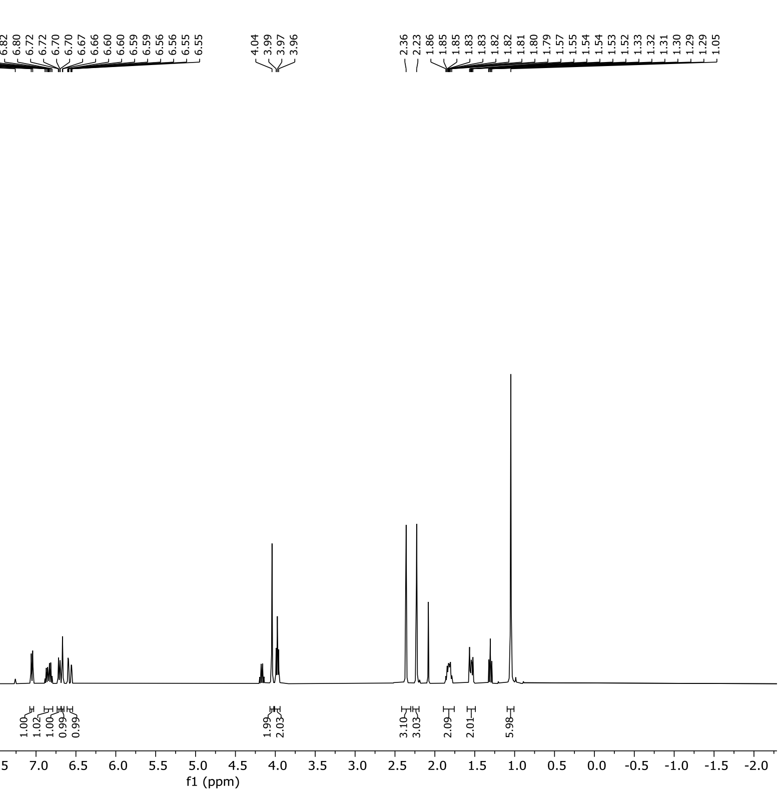

^{13}C NMR (126 MHz, CDCl$_3$) of **2ah**

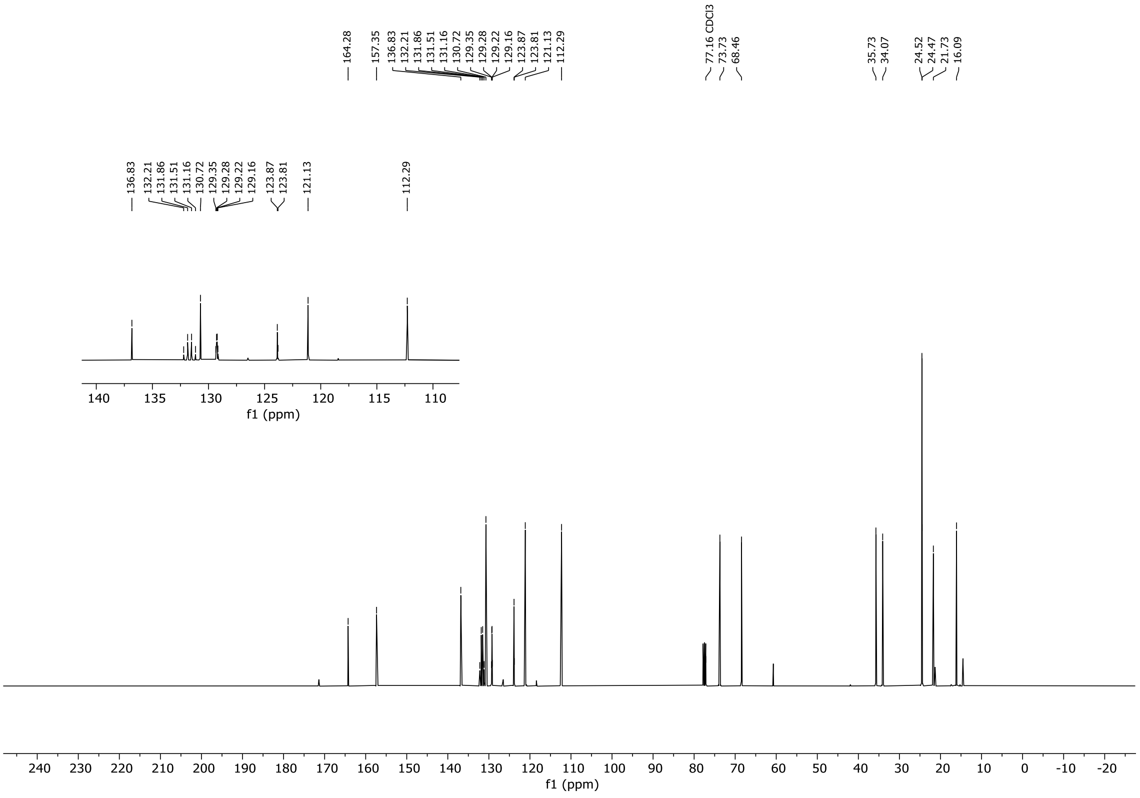

^{19}F NMR (376 MHz, $CDCl_3$) of **2ah**

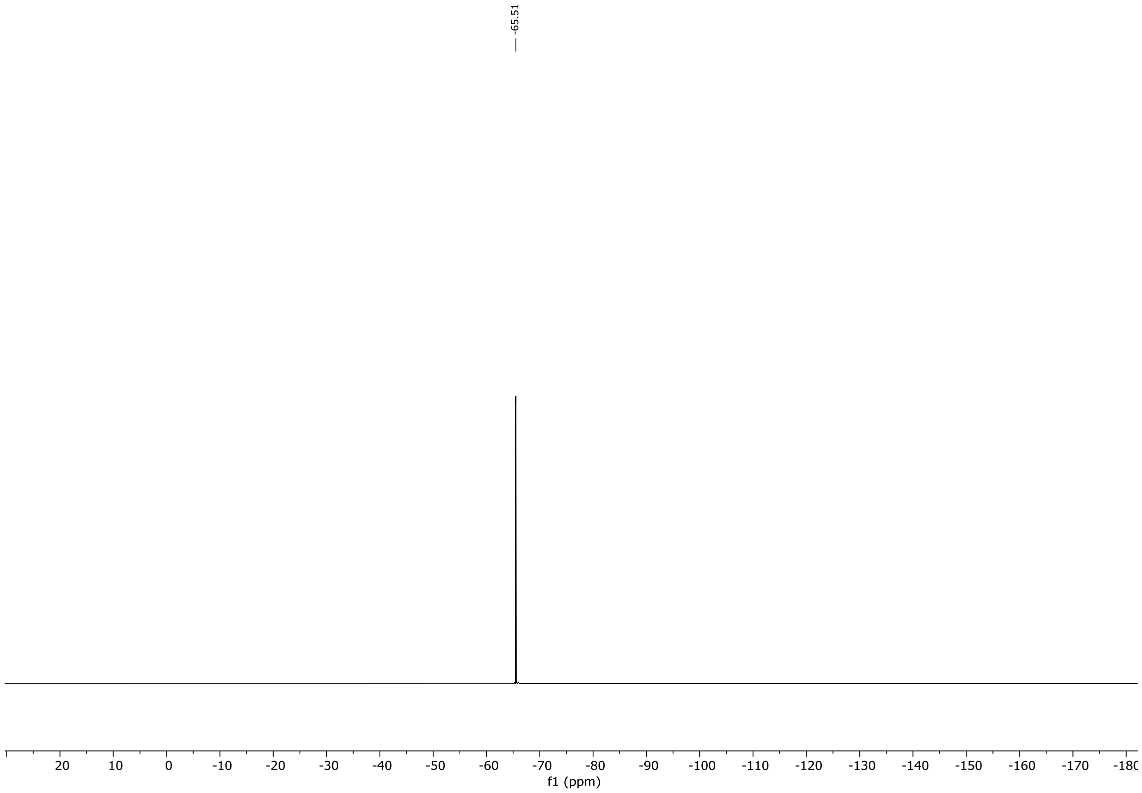

^{1}H NMR (400 MHz, $CDCl_3$) of **3a** (*see procedure*)

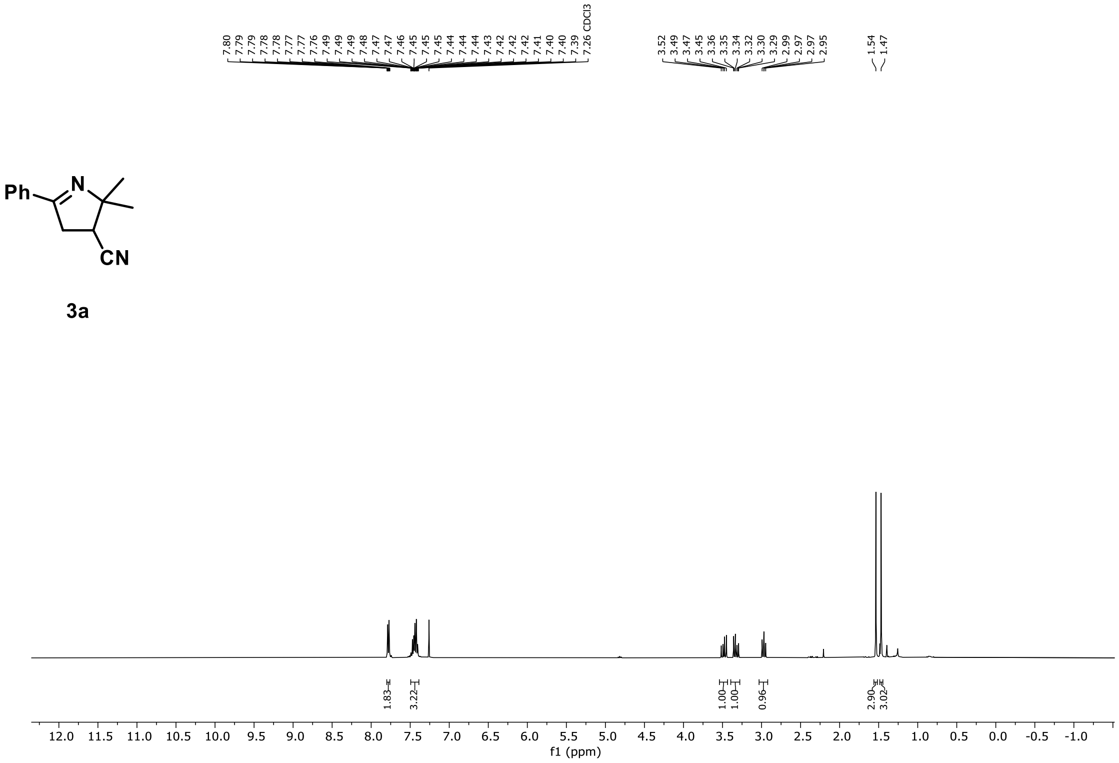

^{13}C NMR (126 MHz, $CDCl_3$) of **3a**

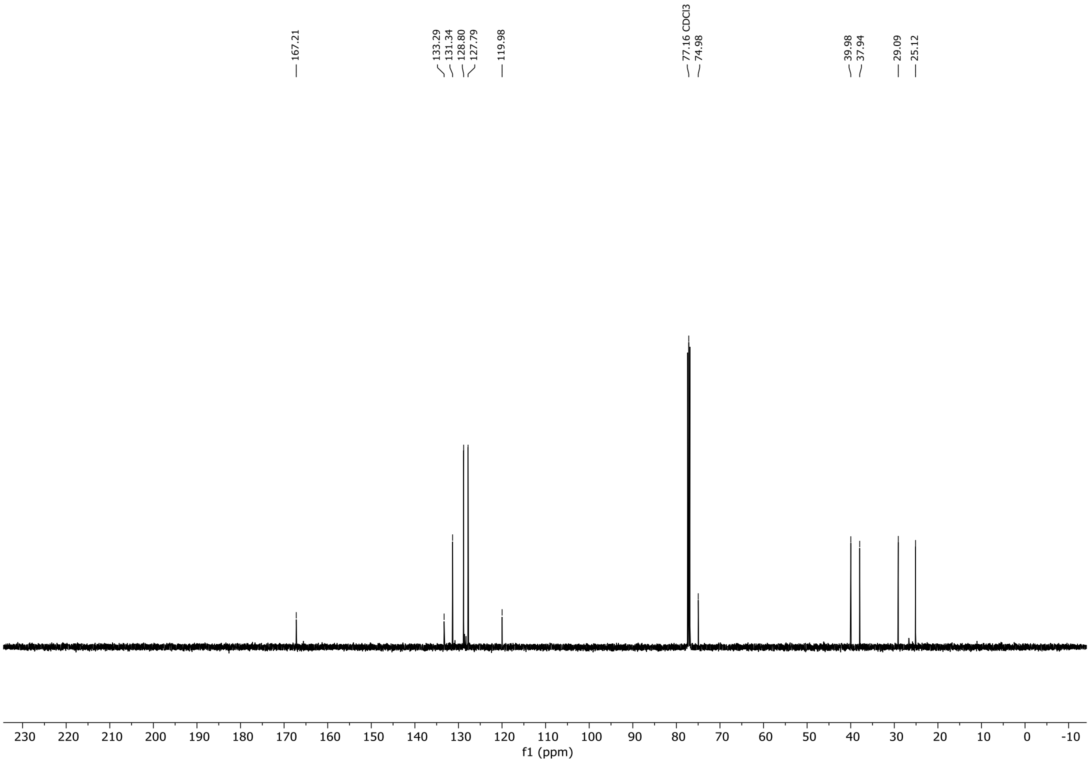

^{1}H NMR (400 MHz, $CDCl_3$) of **3b** (*see procedure*)

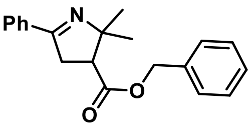

3b

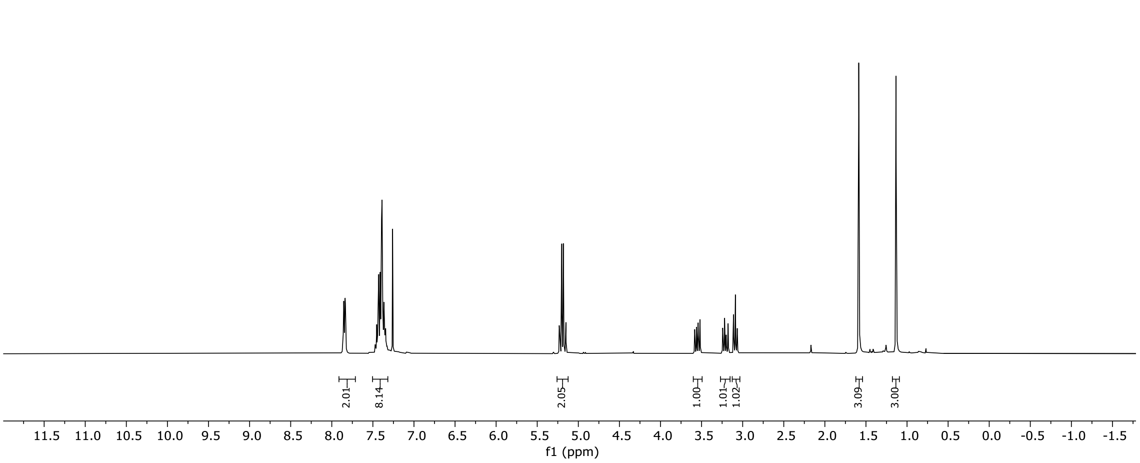

^{13}C NMR (126 MHz, $CDCl_3$) of **3b**

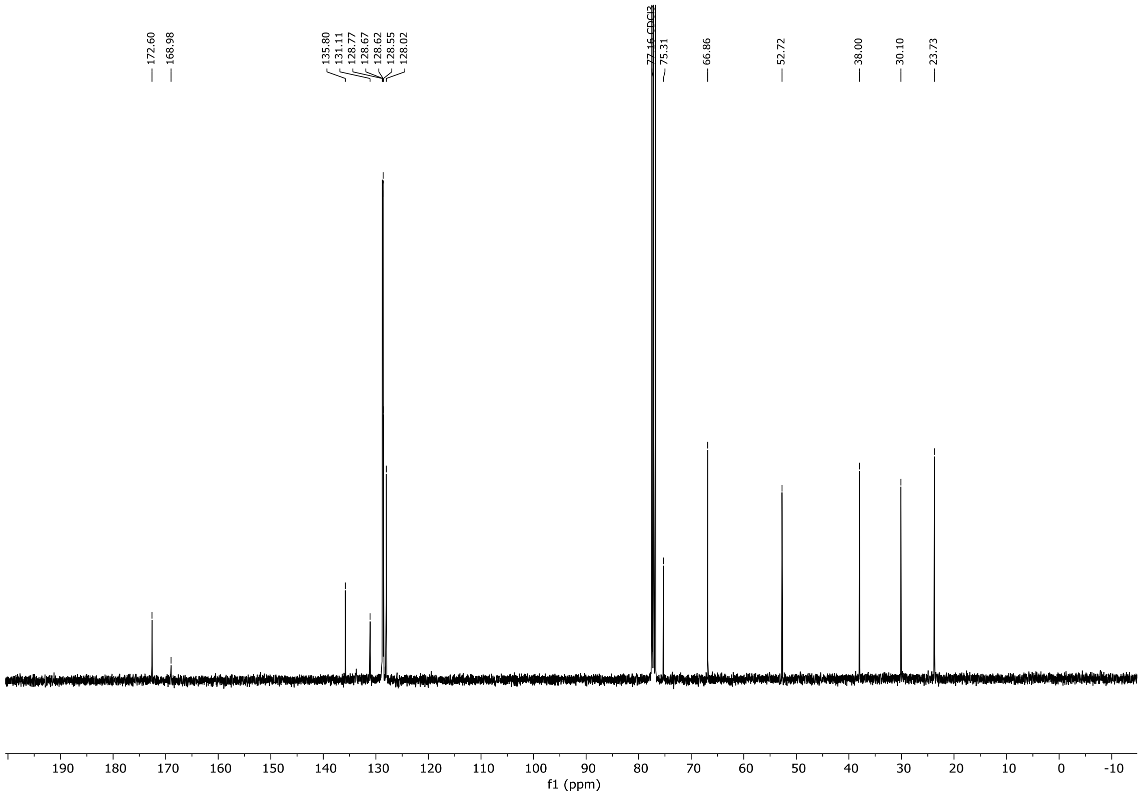

^{1}H NMR (400 MHz, $CDCl_3$) of **3c** (*see procedure*)

3c

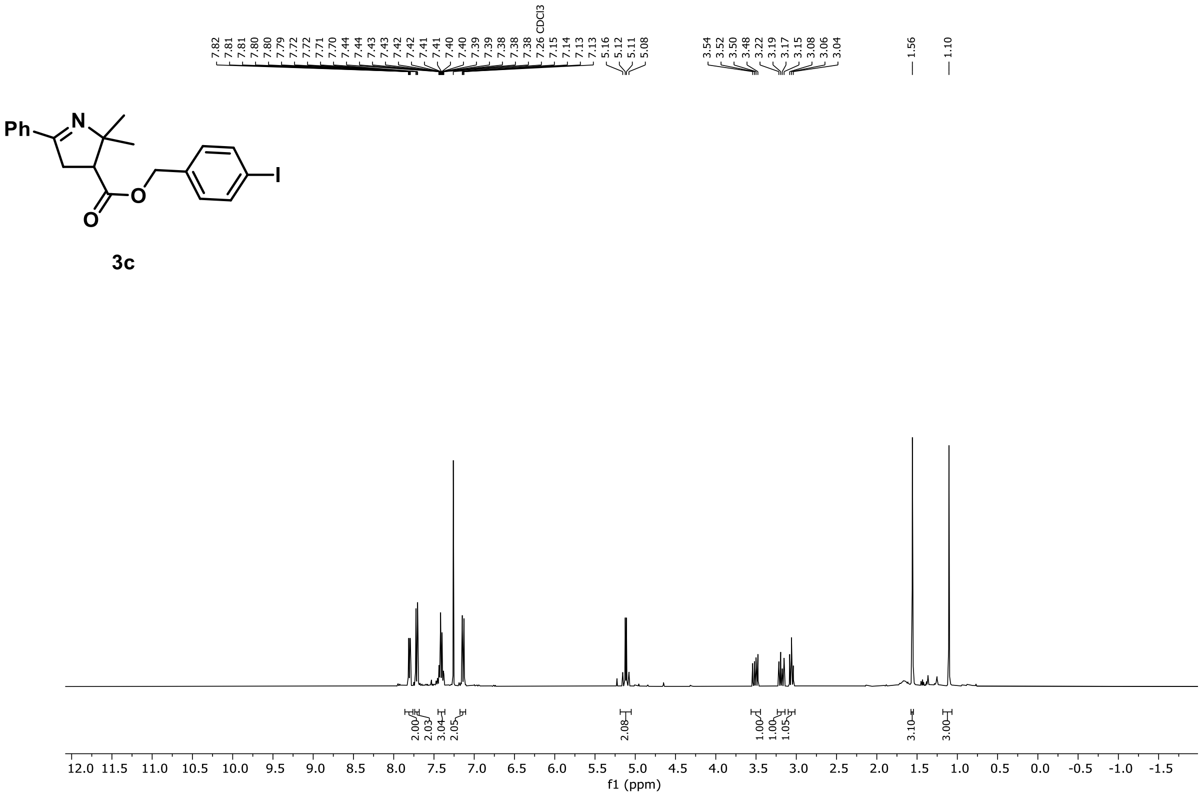

^{13}C NMR (126 MHz, $CDCl_3$) of **3c**

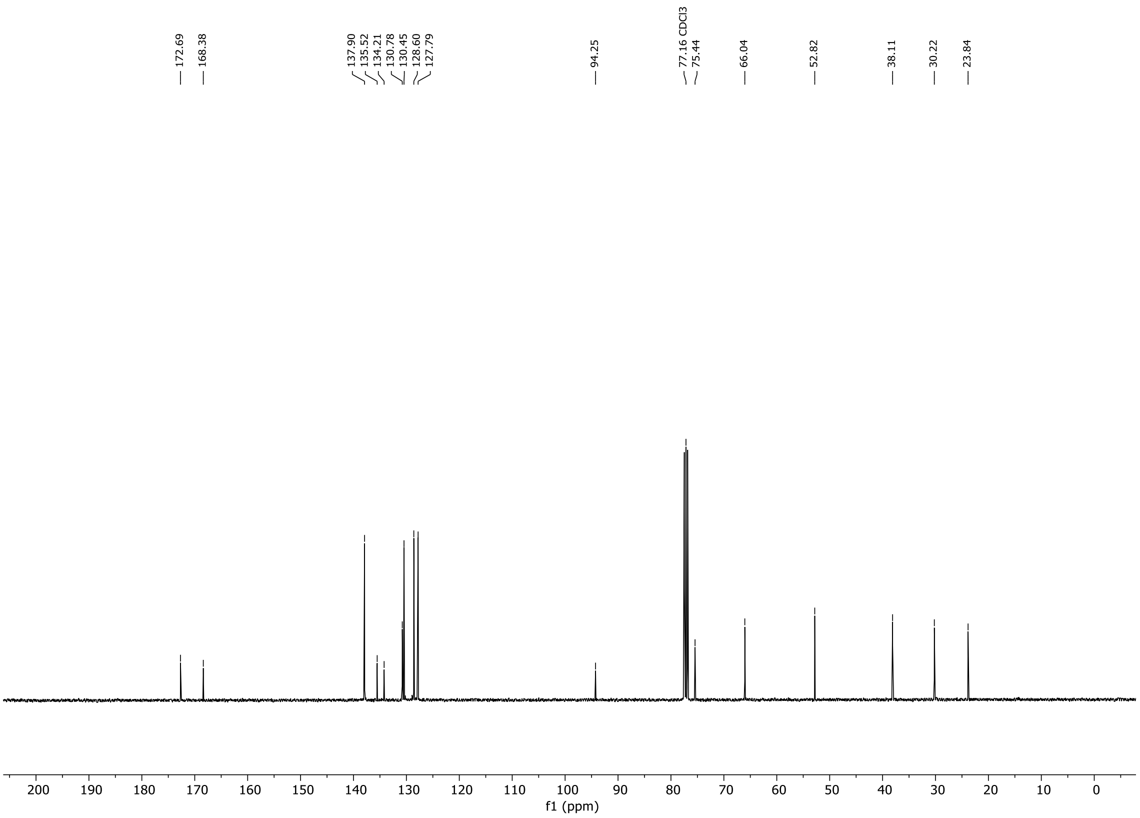

^{1}H NMR (400 MHz, $CDCl_3$) of **3d** (*see procedure*)

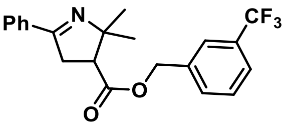

3d

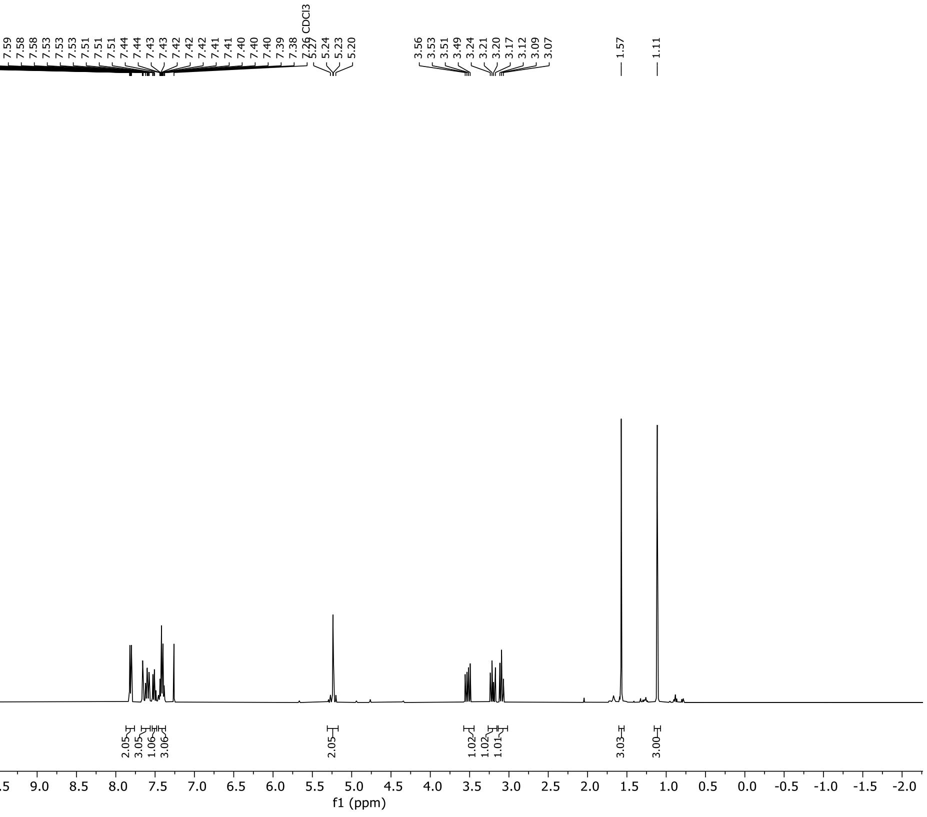

^{13}C NMR (101 MHz, $CDCl_3$) of **3d**

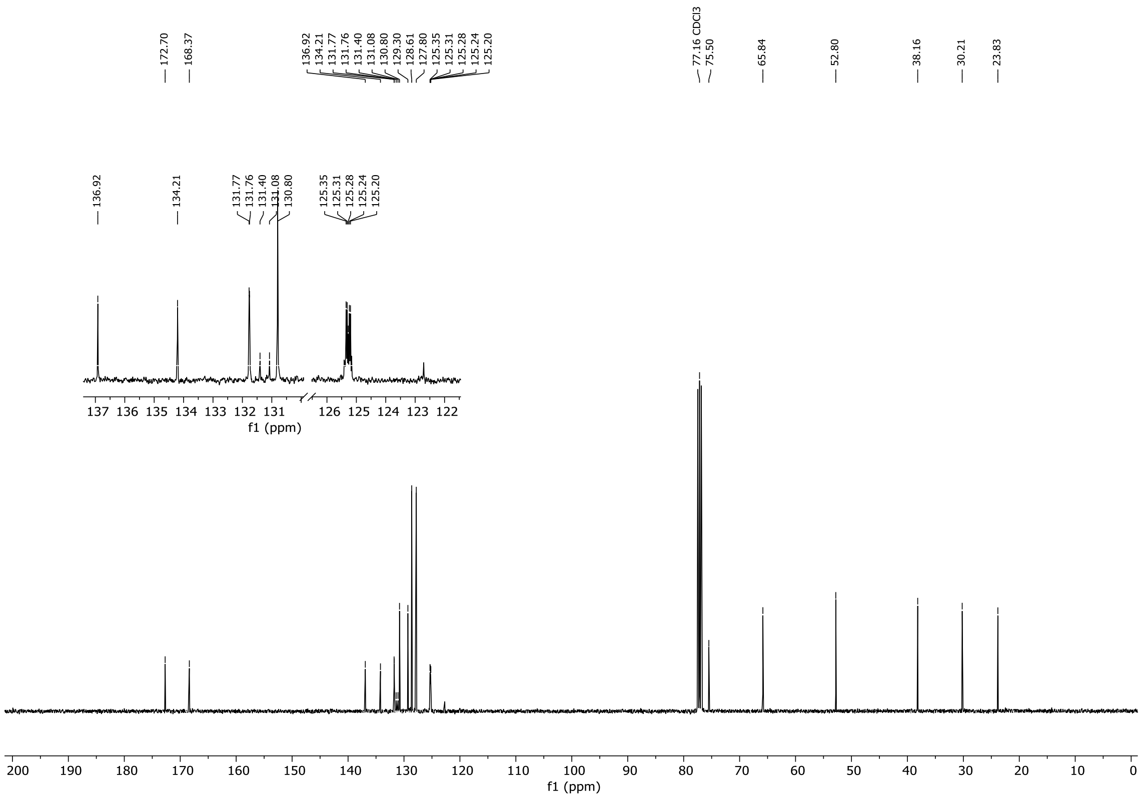

^{13}C NMR {^{1}H, ^{19}F} (126 MHz, $CDCl_3$) of **3d**

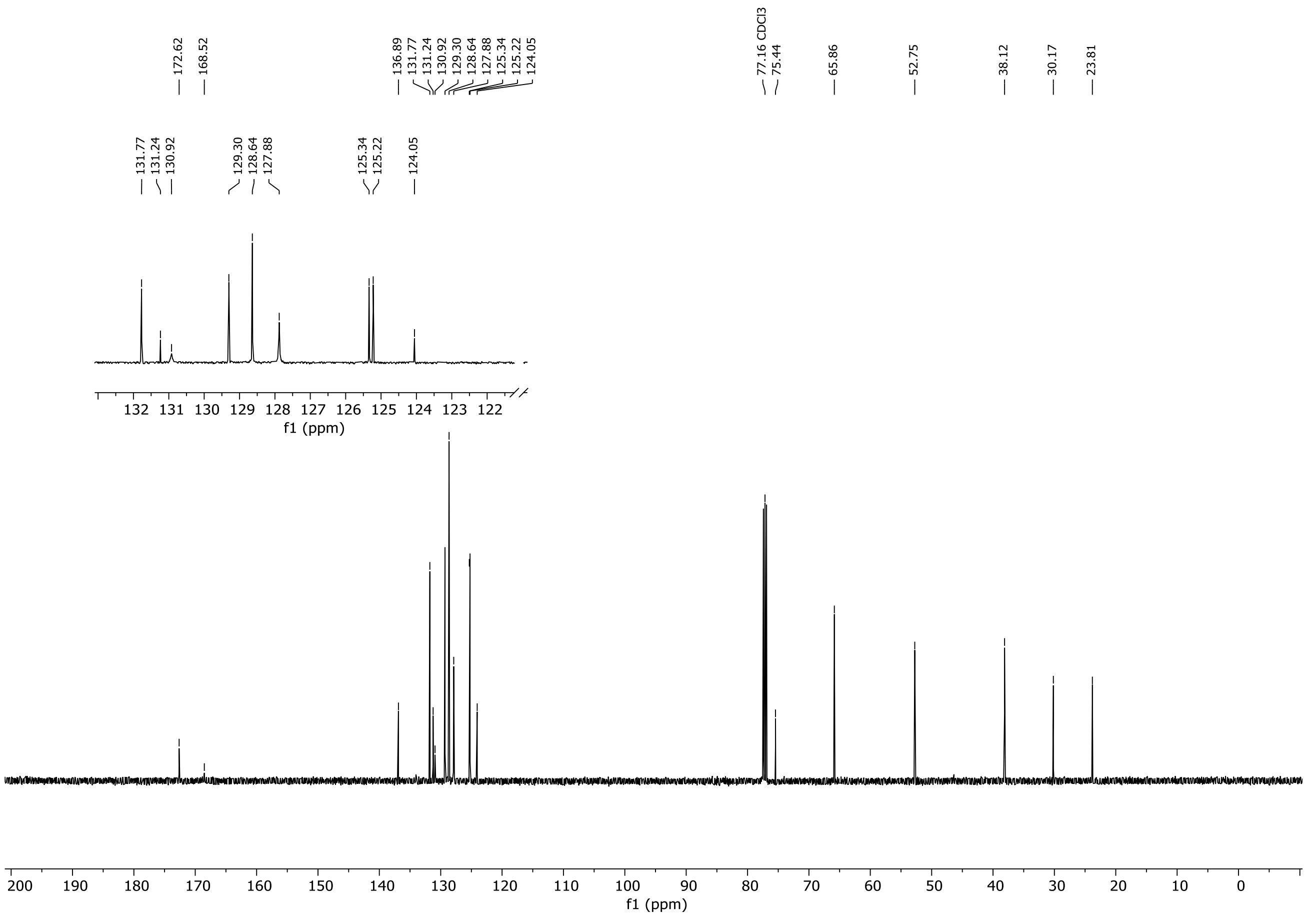

^{19}F NMR (376 MHz, $CDCl_3$) of **3d**

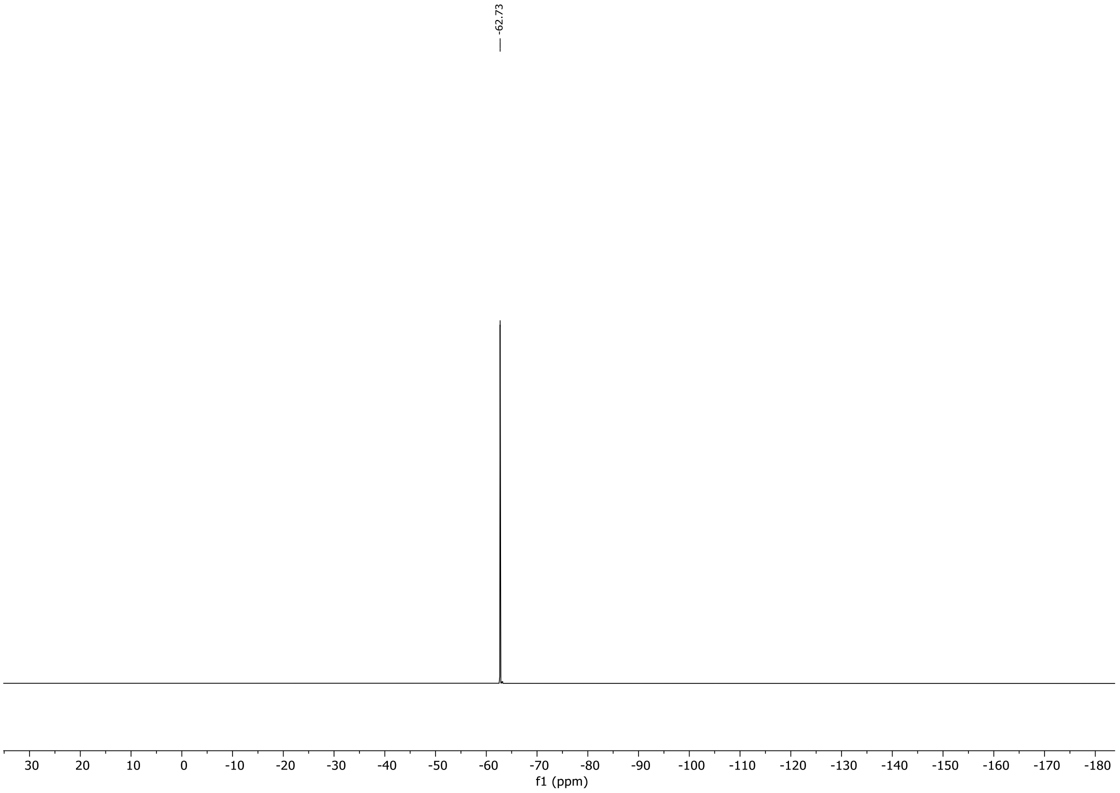

^{1}H NMR (400 MHz, $CDCl_3$) of **3e** (*see procedure*)

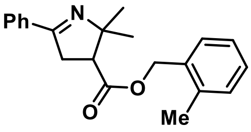

3e

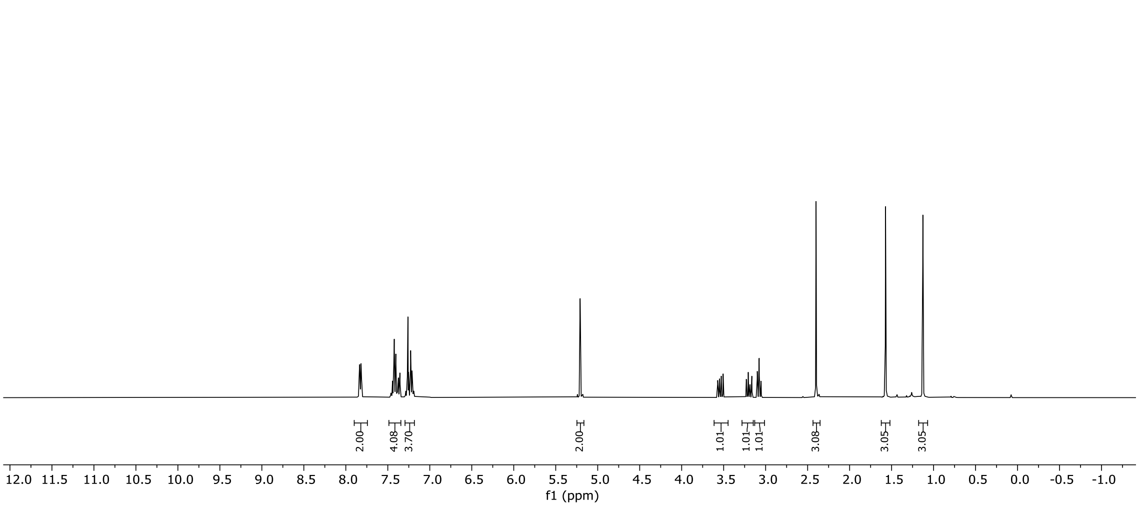

^{13}C NMR (126 MHz, $CDCl_3$) of **3e**

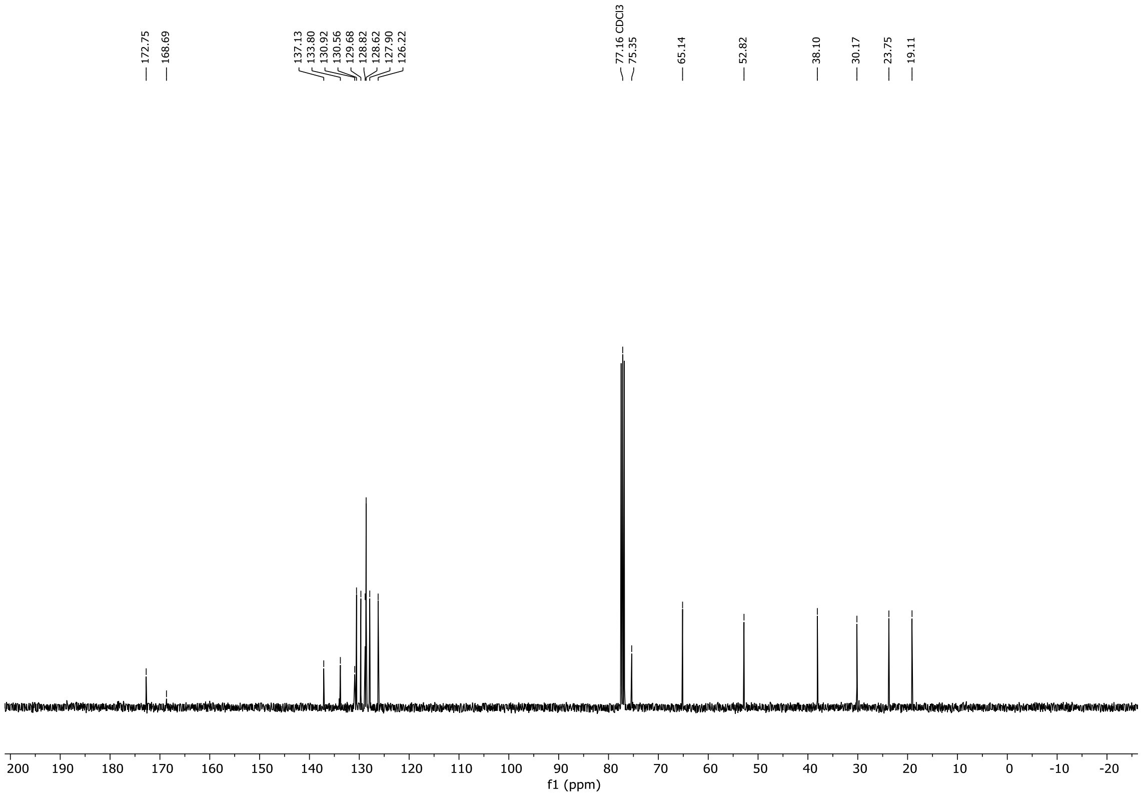

^{1}H NMR (400 MHz, $CDCl_3$) of **3f** (*see procedure*)

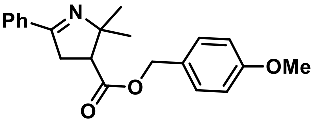

3f

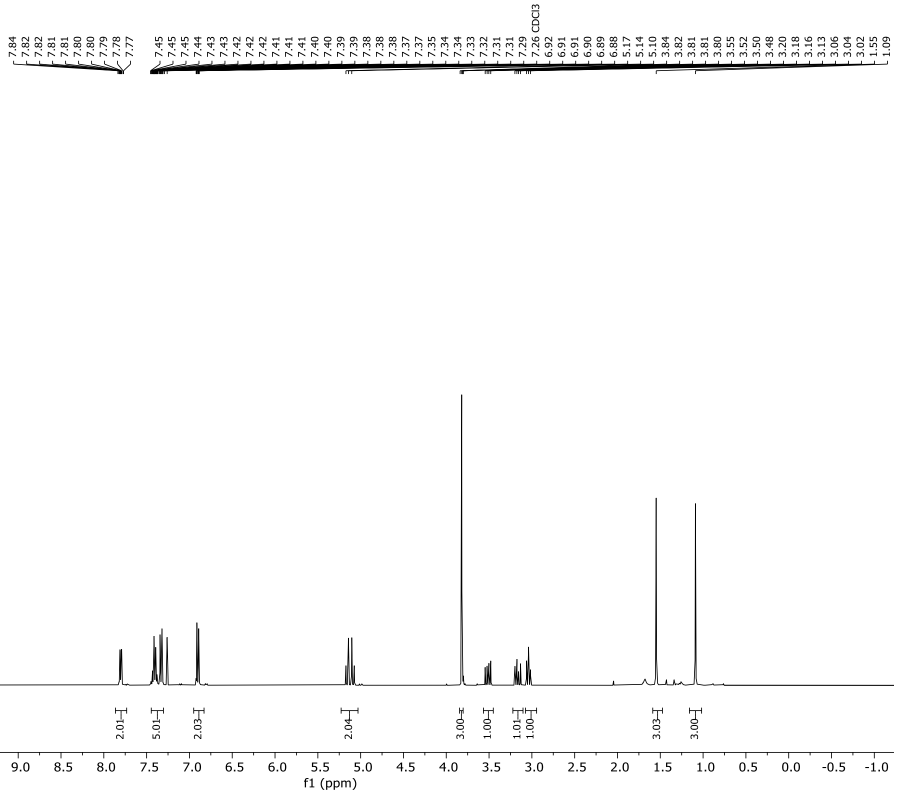

^{13}C NMR (126 MHz, $CDCl_3$) of **3f**

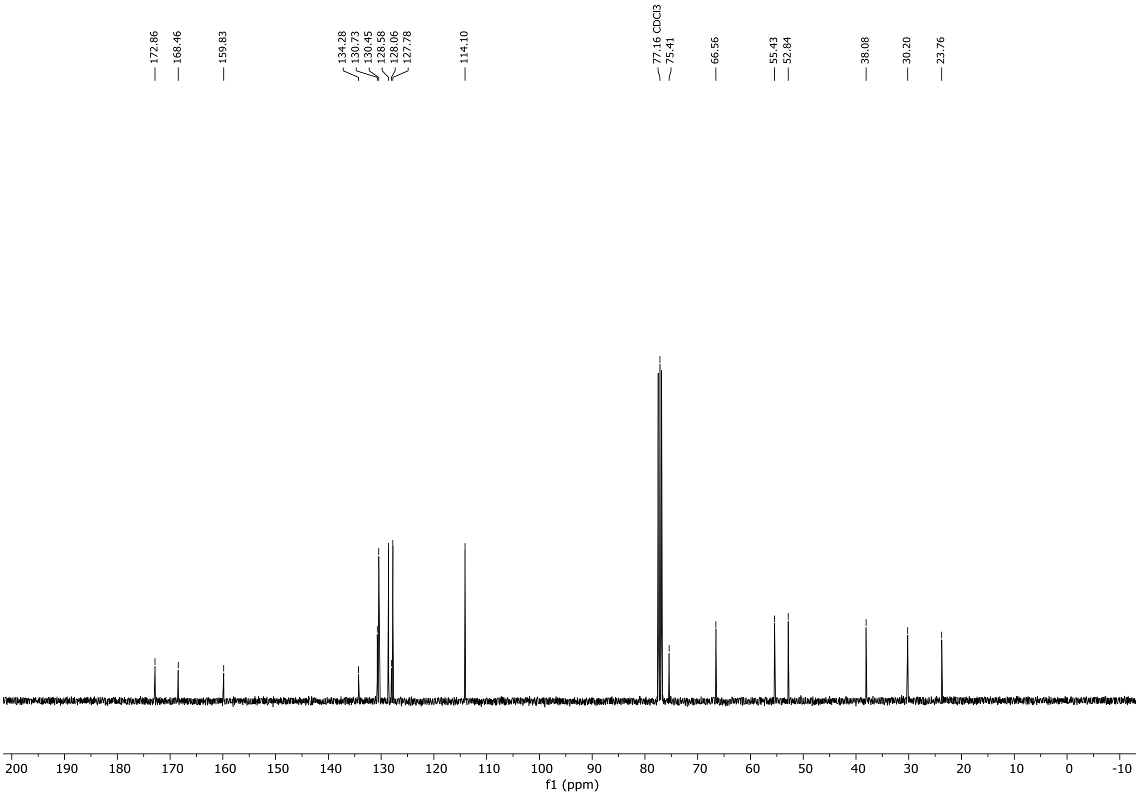

^{1}H NMR (400 MHz, $CDCl_3$) of **3g** (*see procedure*)

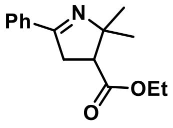

3g

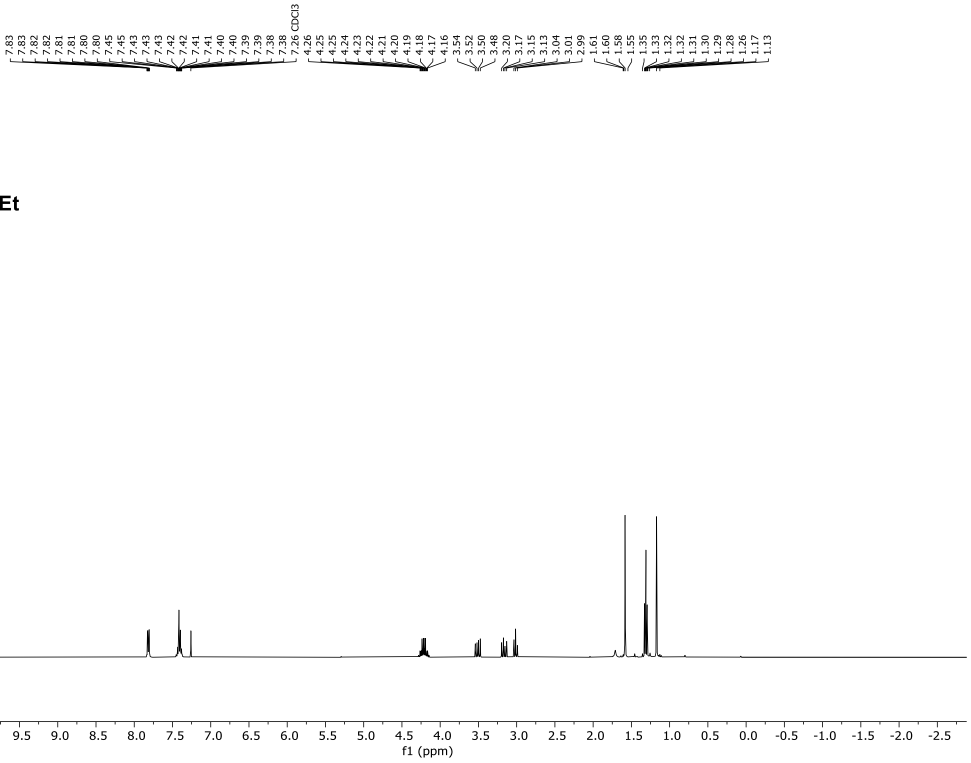

^{13}C NMR (101 MHz, $CDCl_3$) of **3g**

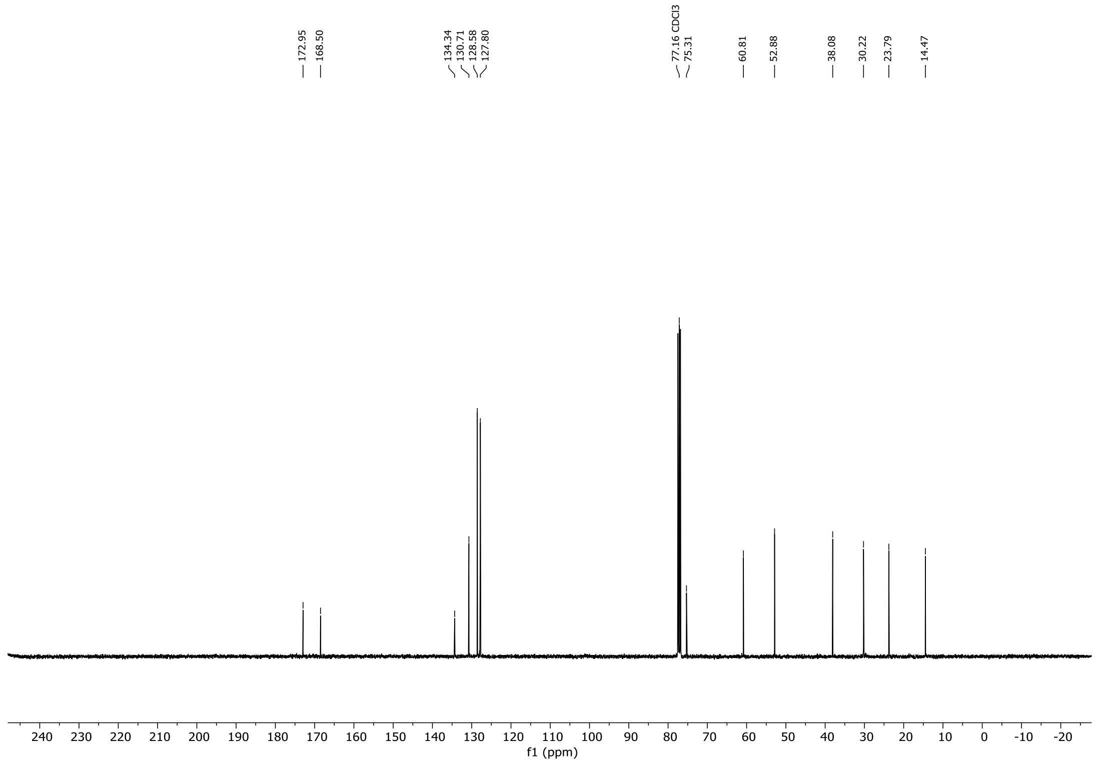

^{1}H NMR (400 MHz, $CDCl_3$) of **3h** (*see procedure*)

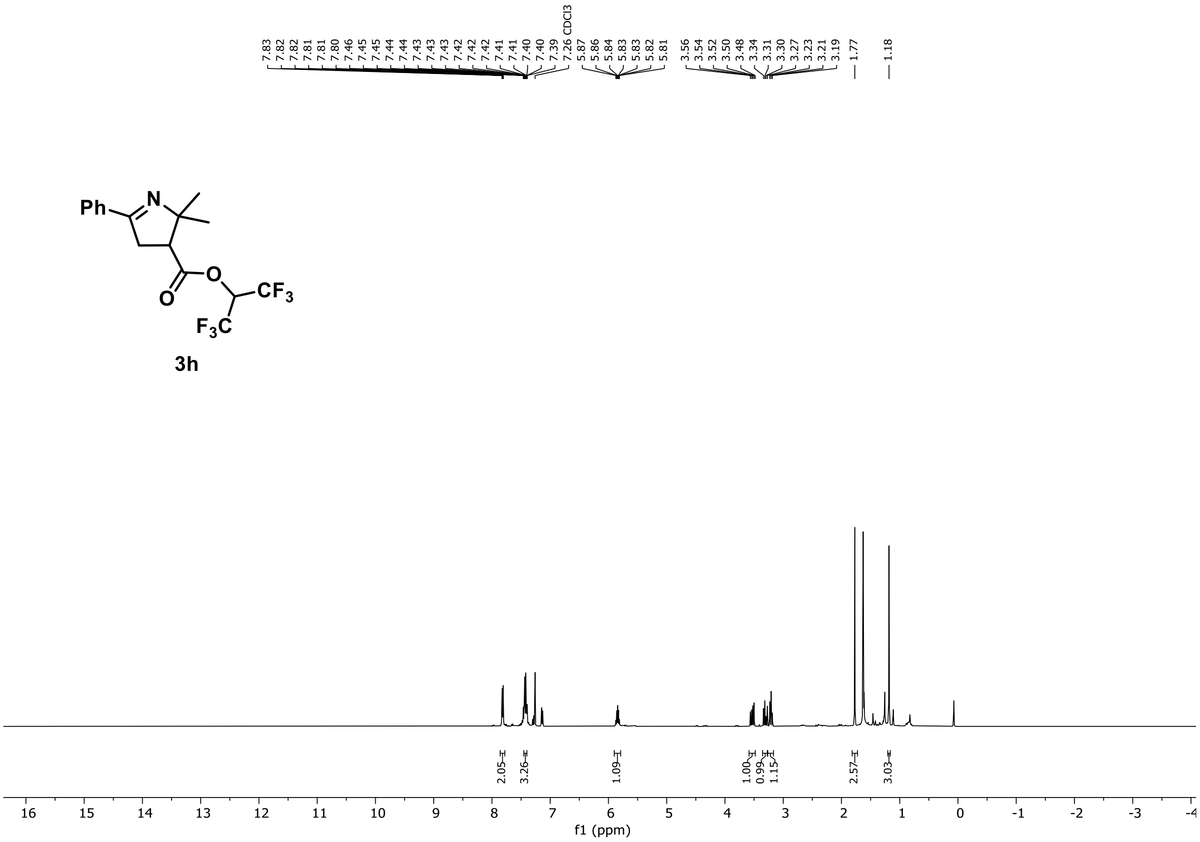

^{13}C NMR (101 MHz, $CDCl_3$) of **3h**

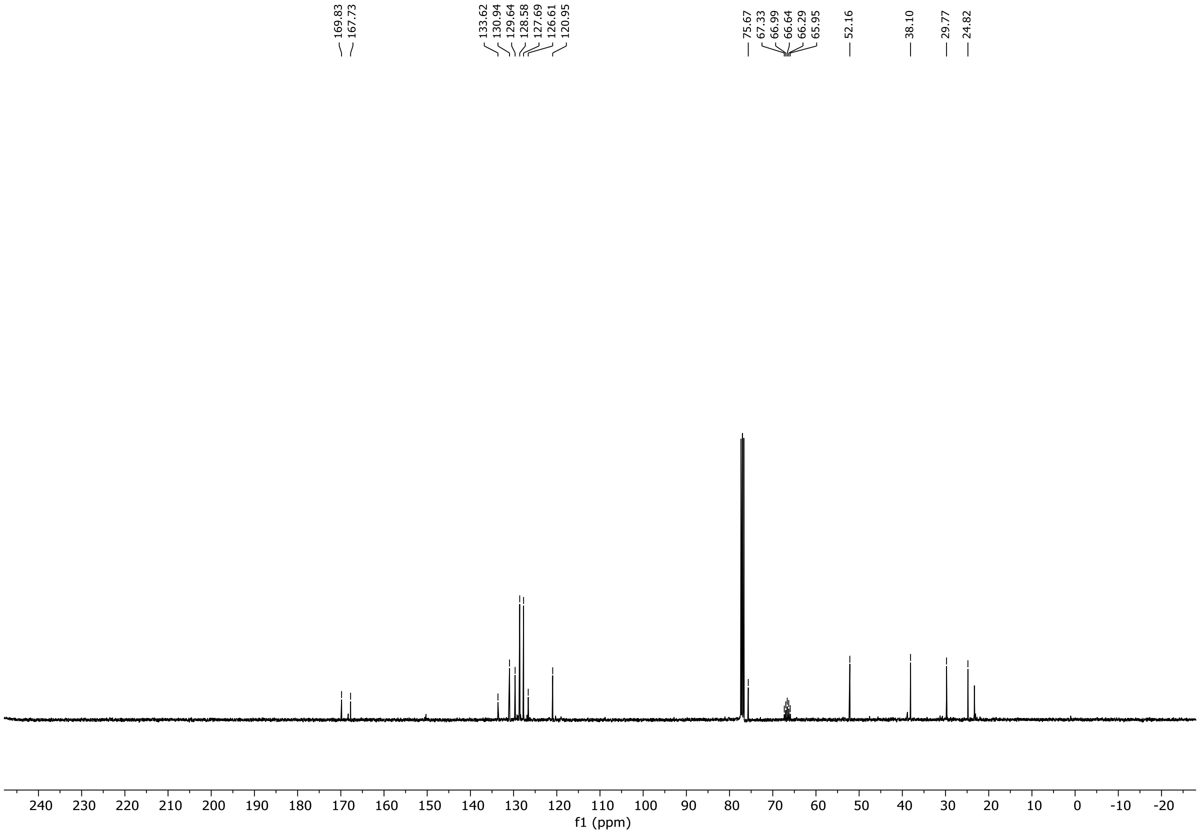

^{19}F NMR (376 MHz, $CDCl_3$) of **3h**

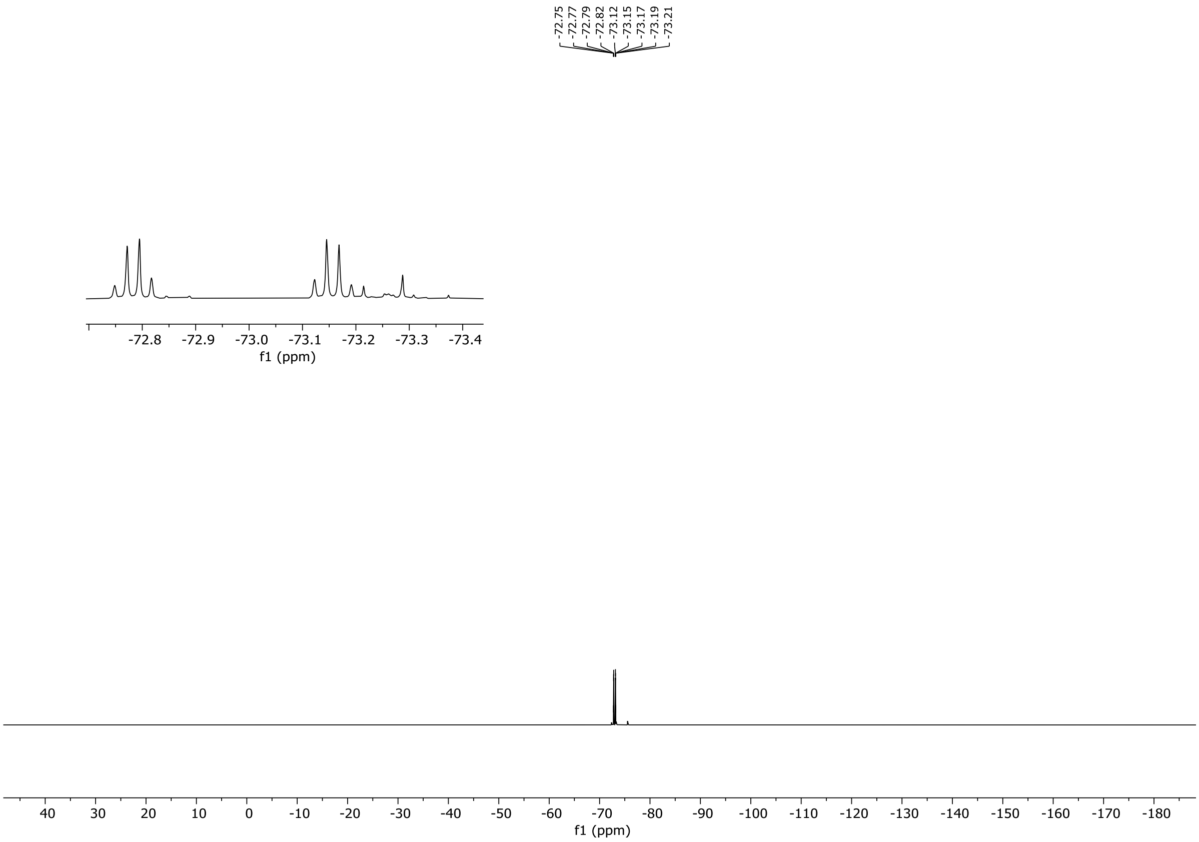

^{1}H NMR (400 MHz, $CDCl_3$) of **3i** (*see procedure*)

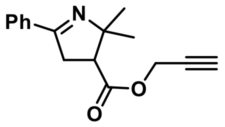

3i

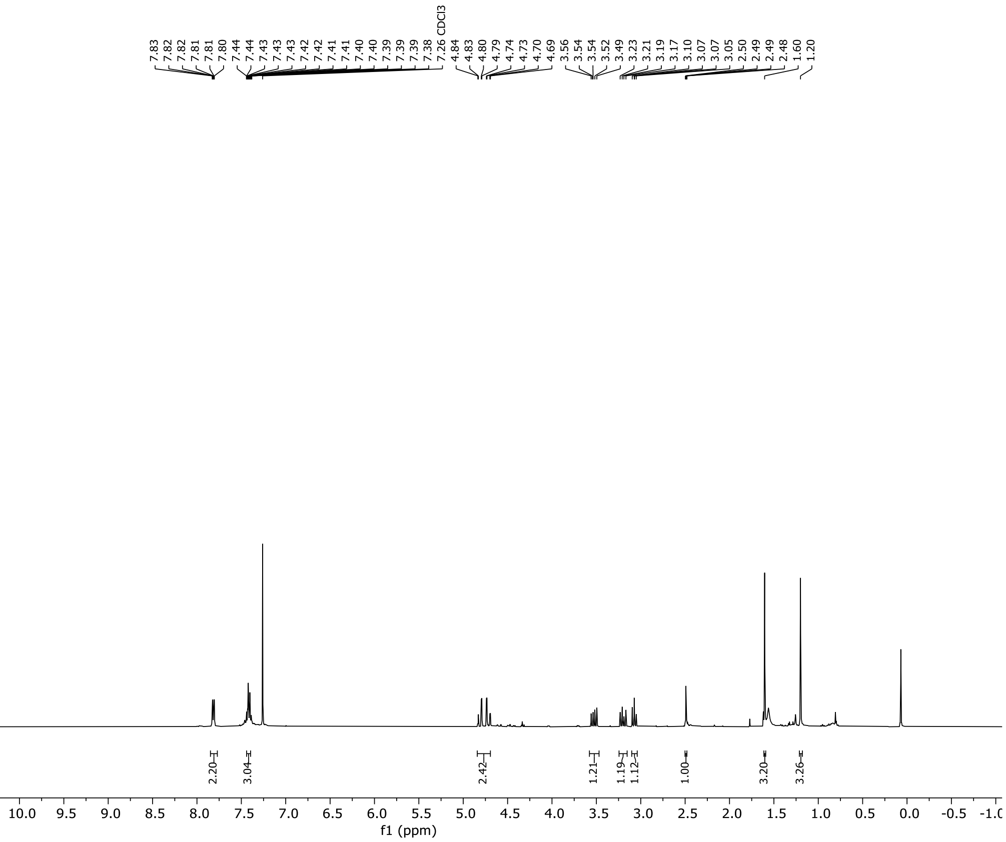

^{13}C NMR (101 MHz, $CDCl_3$) of **3i**

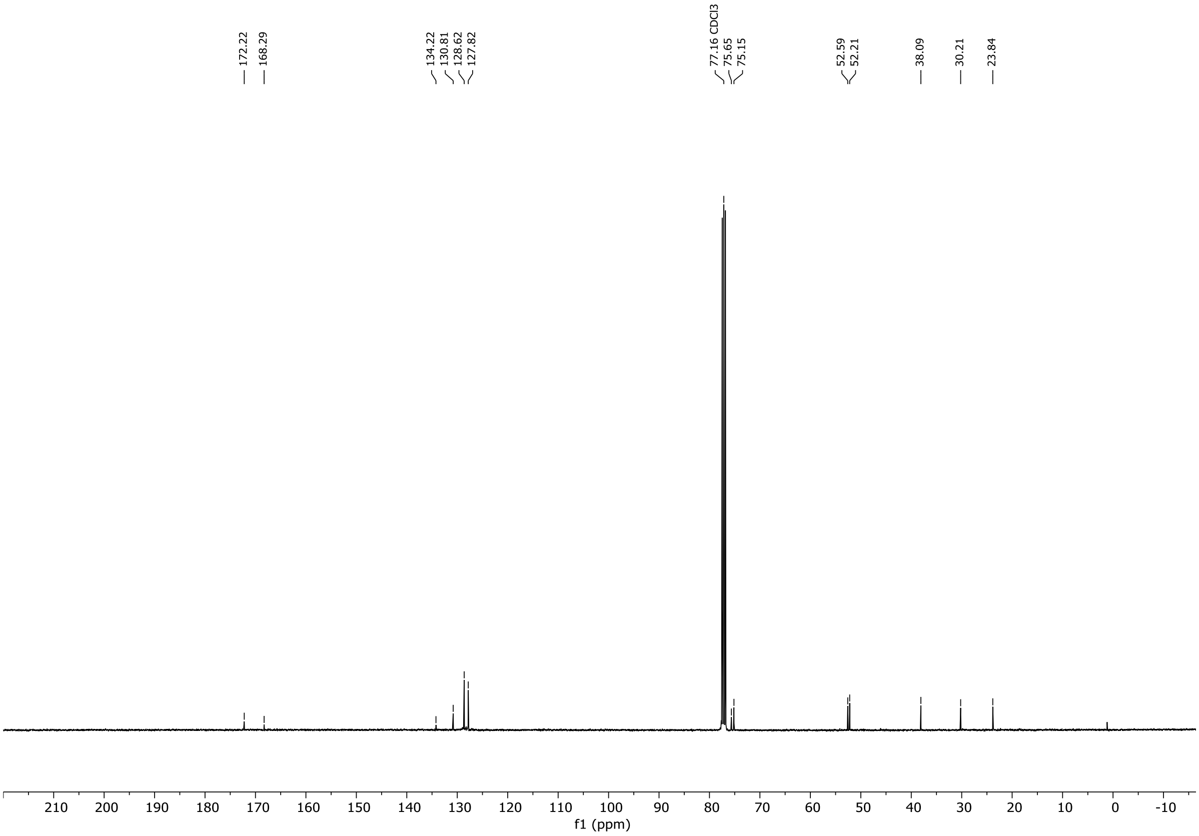

^{1}H NMR (400 MHz, $CDCl_3$) of **3k** (*see procedure*)

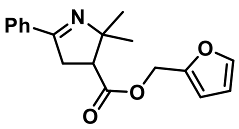

3k

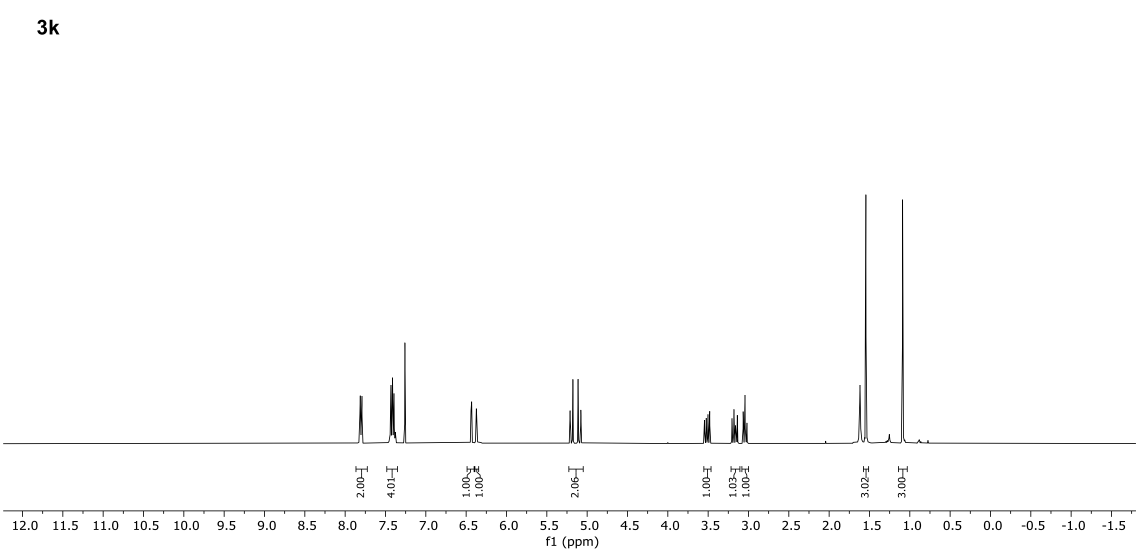

^{13}C NMR (126 MHz, $CDCl_3$) of **3k**

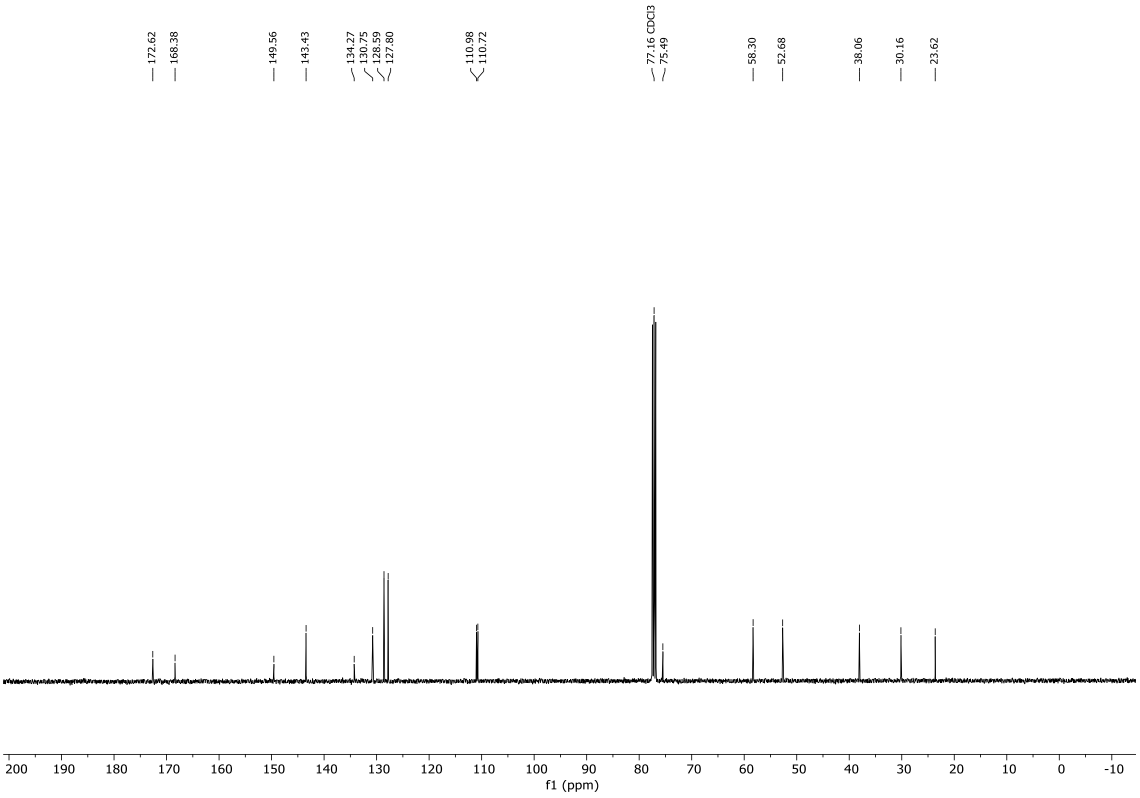

^{1}H NMR (400 MHz, $CDCl_3$) of **3l** (*see procedure*)

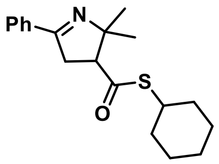

3l

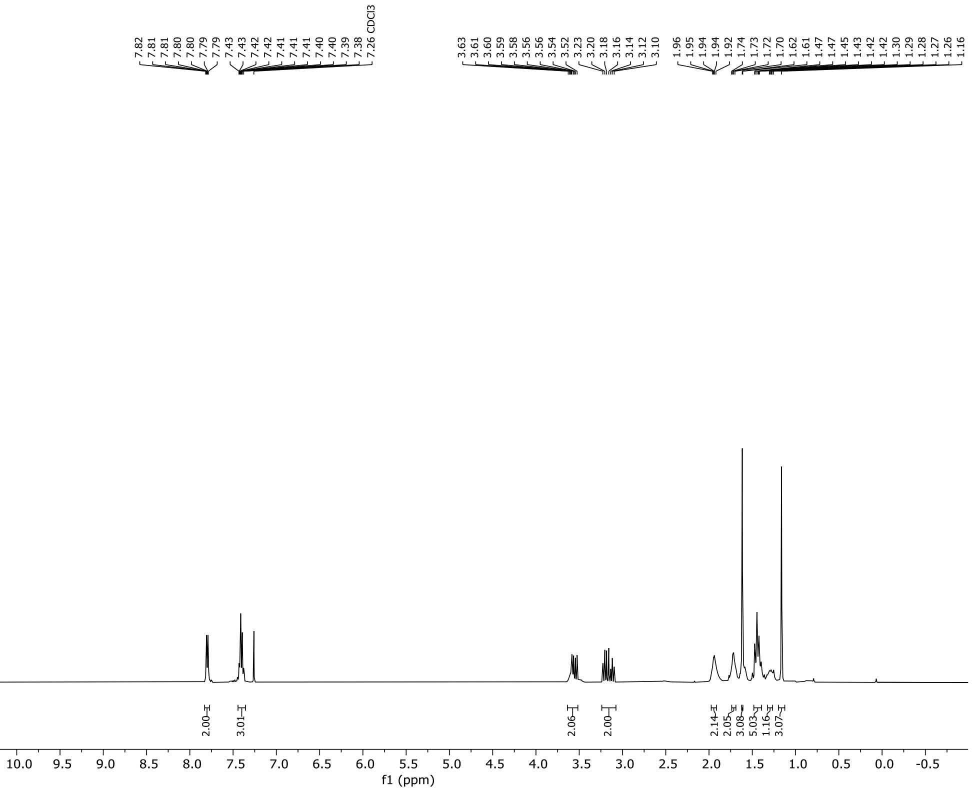

^{13}C NMR (101 MHz, $CDCl_3$) of **3l**

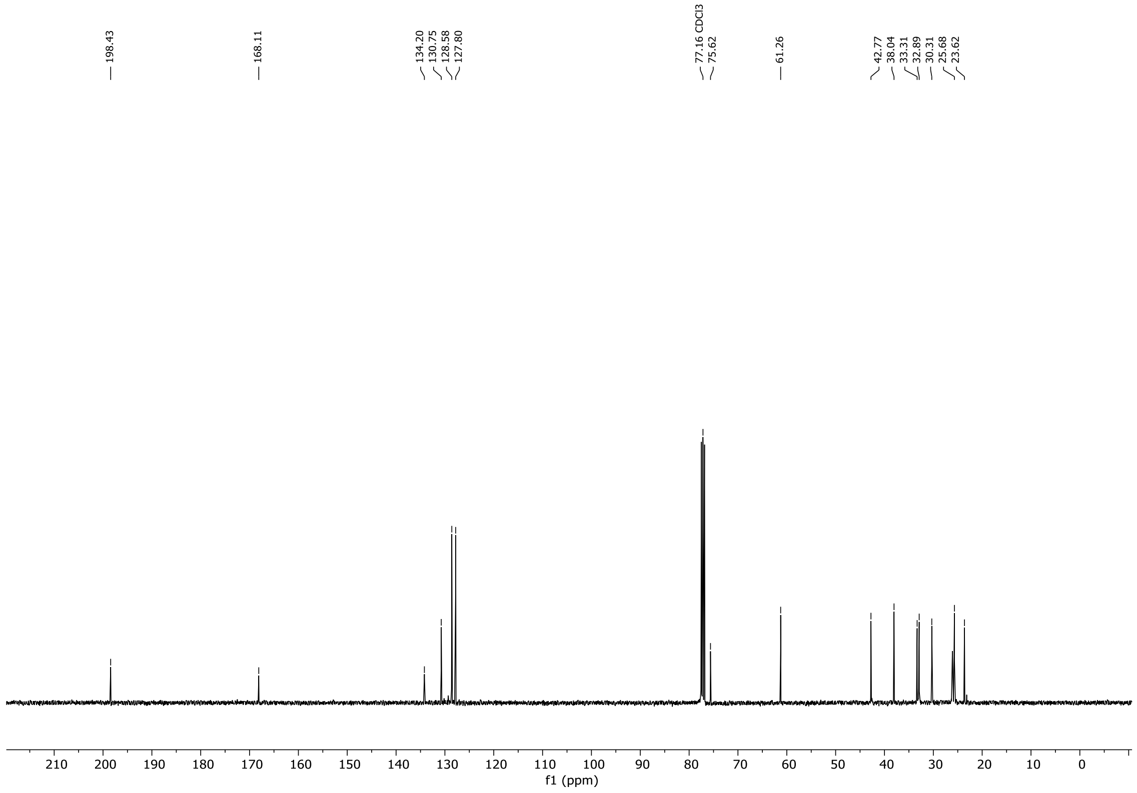

^{1}H NMR (400 MHz, $CDCl_3$) of **3m** (*see procedure*)

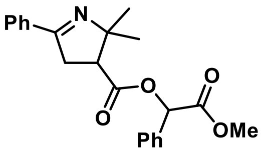

3m

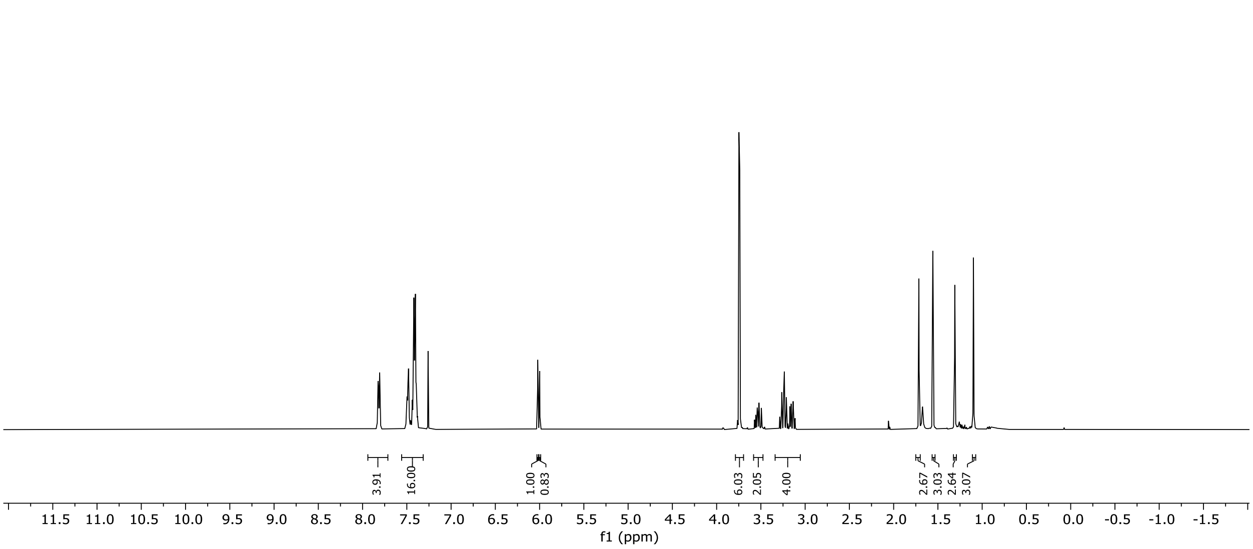

^{13}C NMR (126 MHz, $CDCl_3$) of **3m**

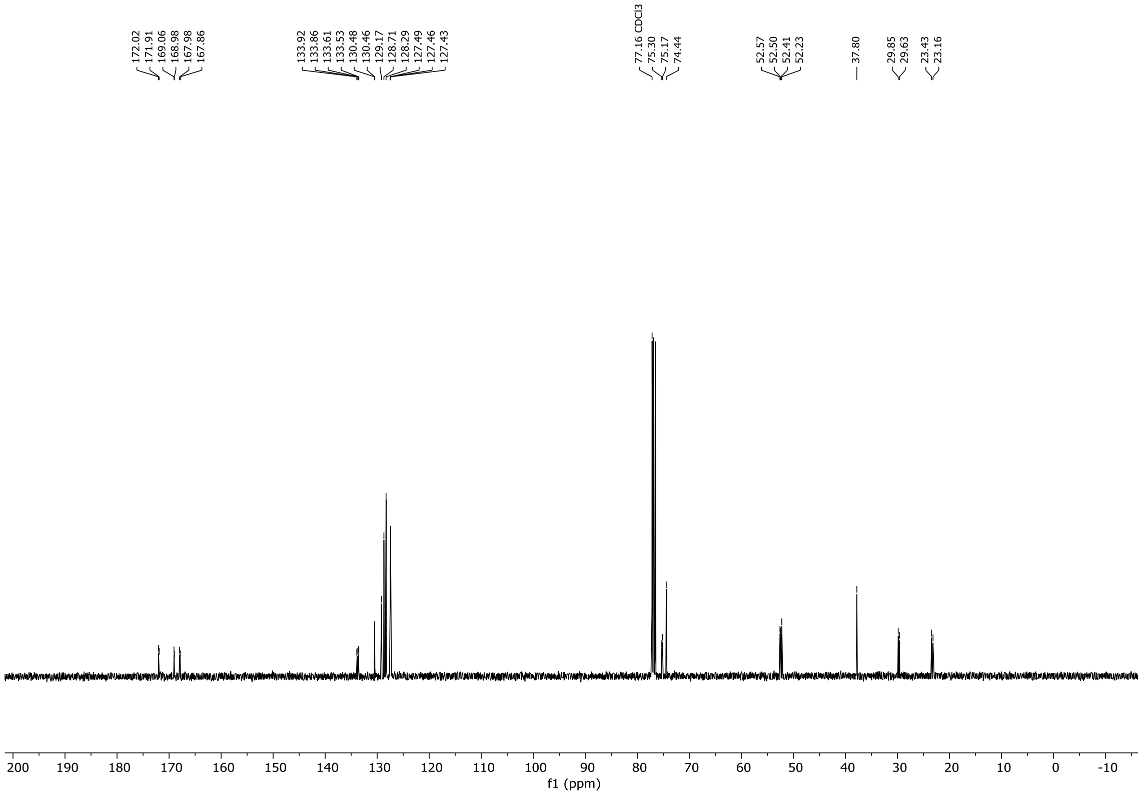

^{1}H NMR (400 MHz, $CDCl_3$) of **3n** (*see procedure*)

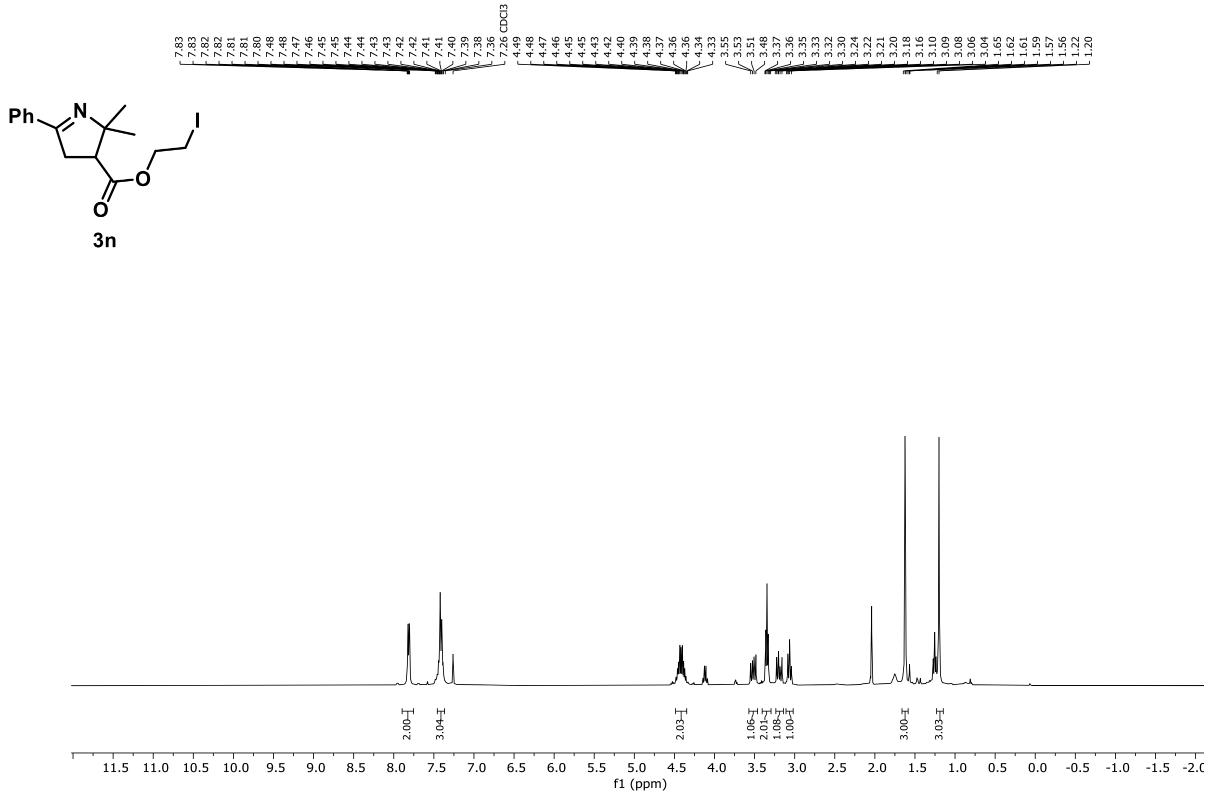

^{13}C NMR (126 MHz, $CDCl_3$) of **3n**

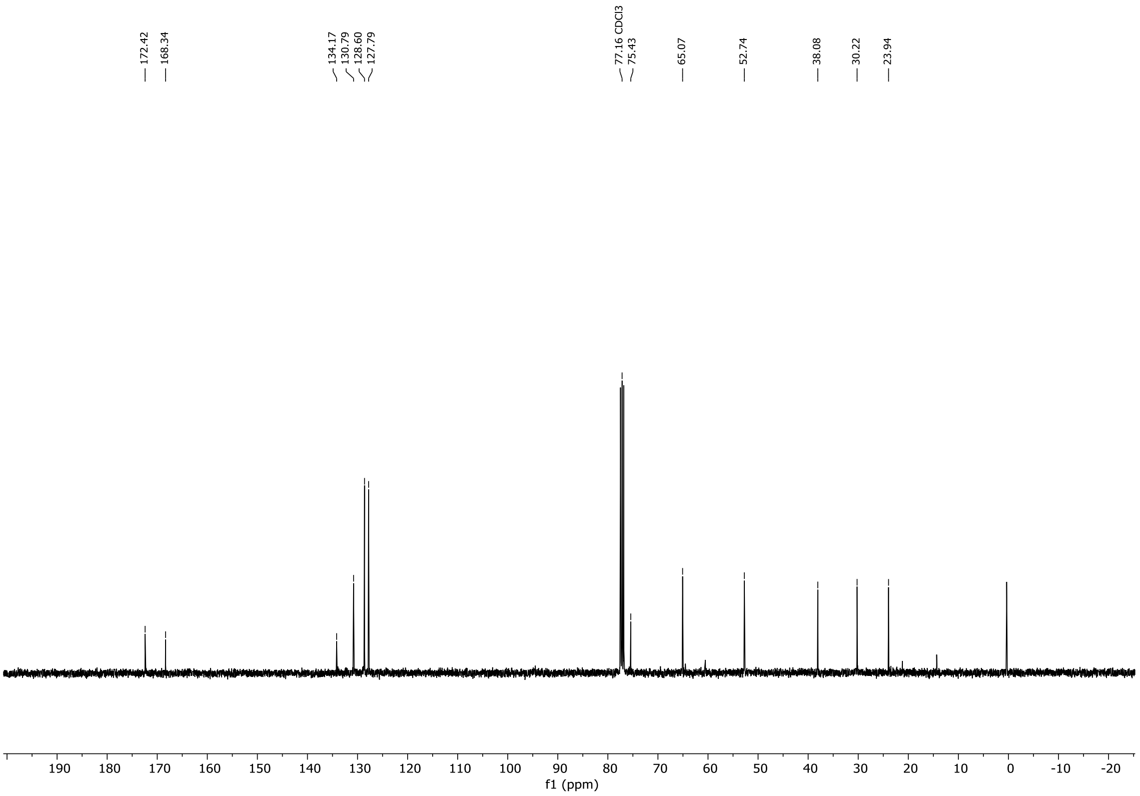

^{1}H NMR (400 MHz, $CDCl_3$) of **3o** (*see procedure*)

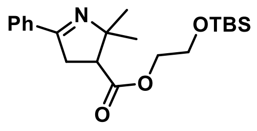

3o

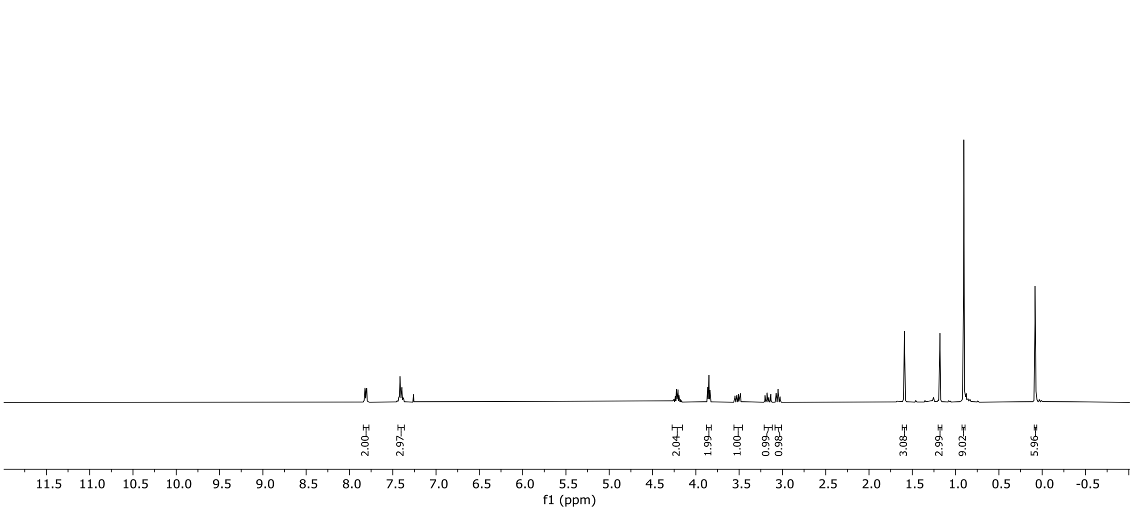

^{13}C NMR (101 MHz, $CDCl_3$) of **3o**

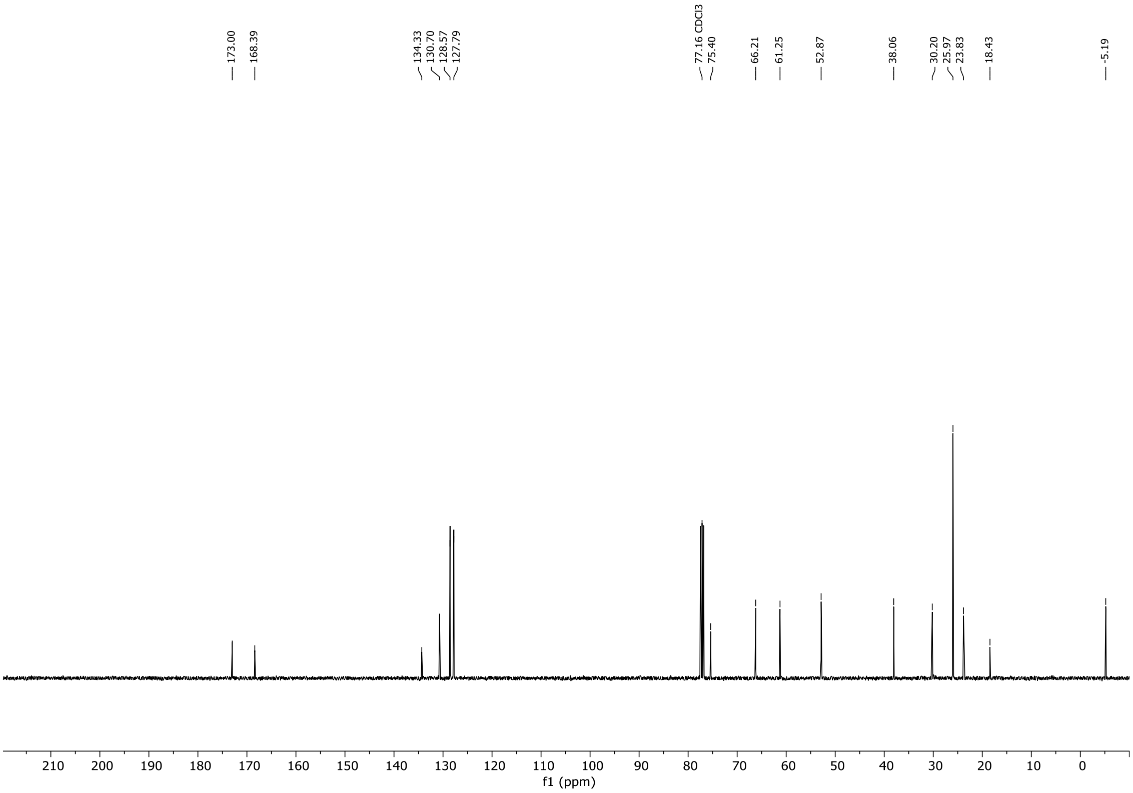

^{1}H NMR (400 MHz, $CDCl_3$) of **3p** (<u>*see procedure*</u>)

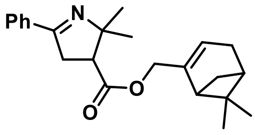

3p

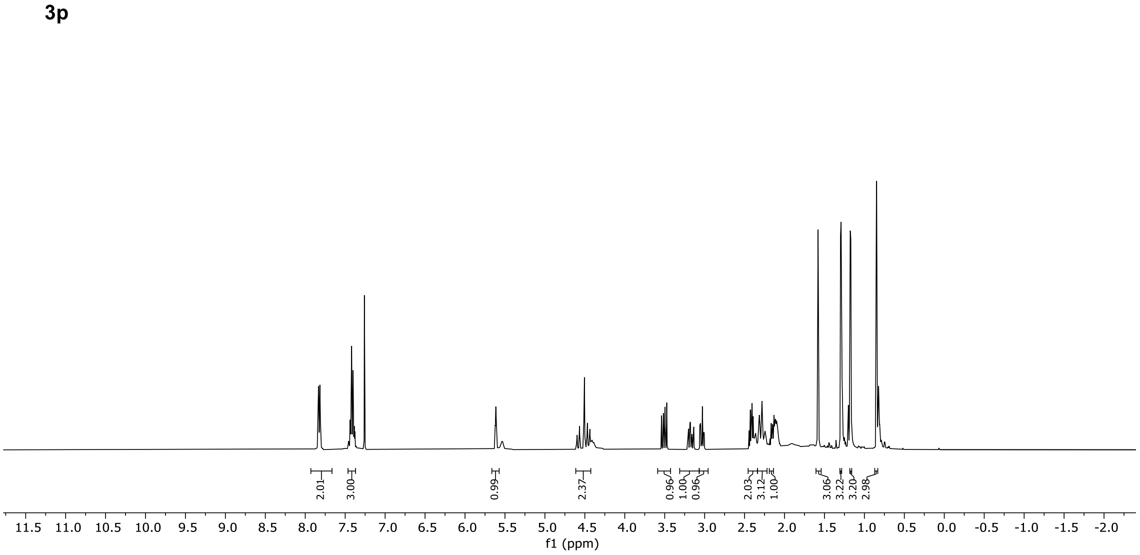

^{13}C NMR (126 MHz, $CDCl_3$) of **3p**

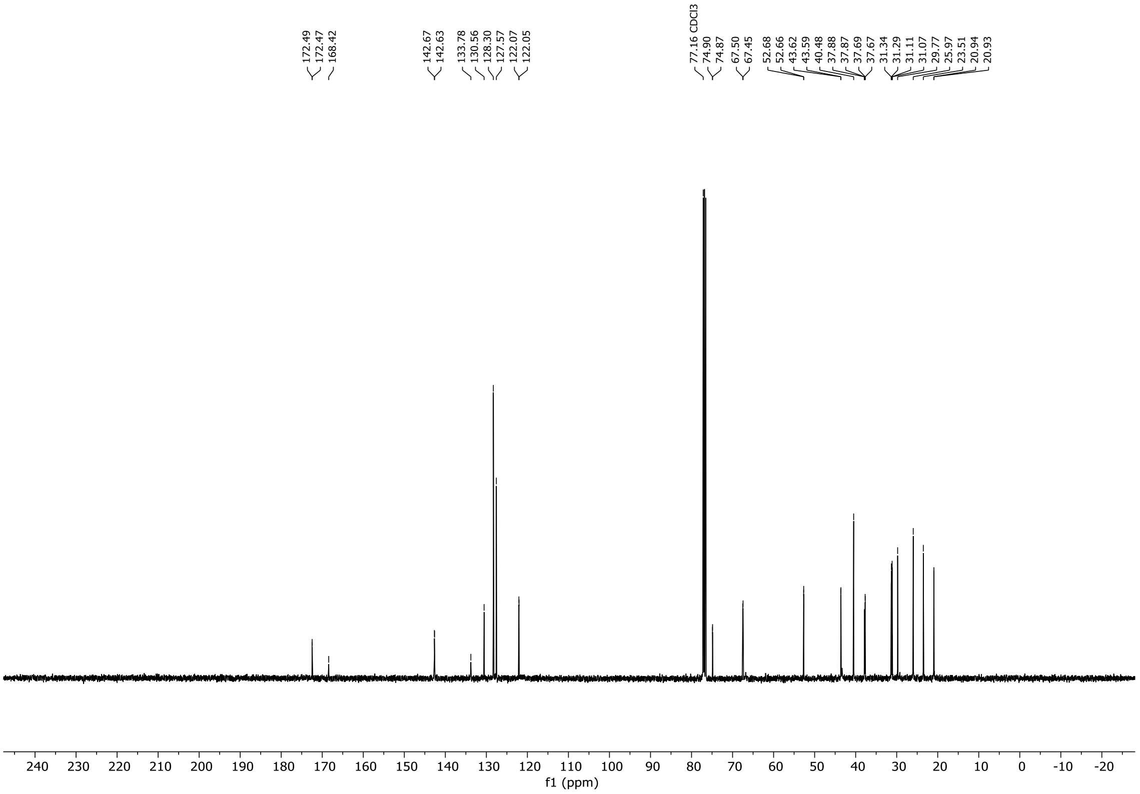

^{1}H NMR (400 MHz, $CDCl_3$) of **3q** (*see procedure*)

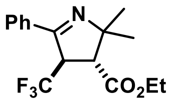

3q

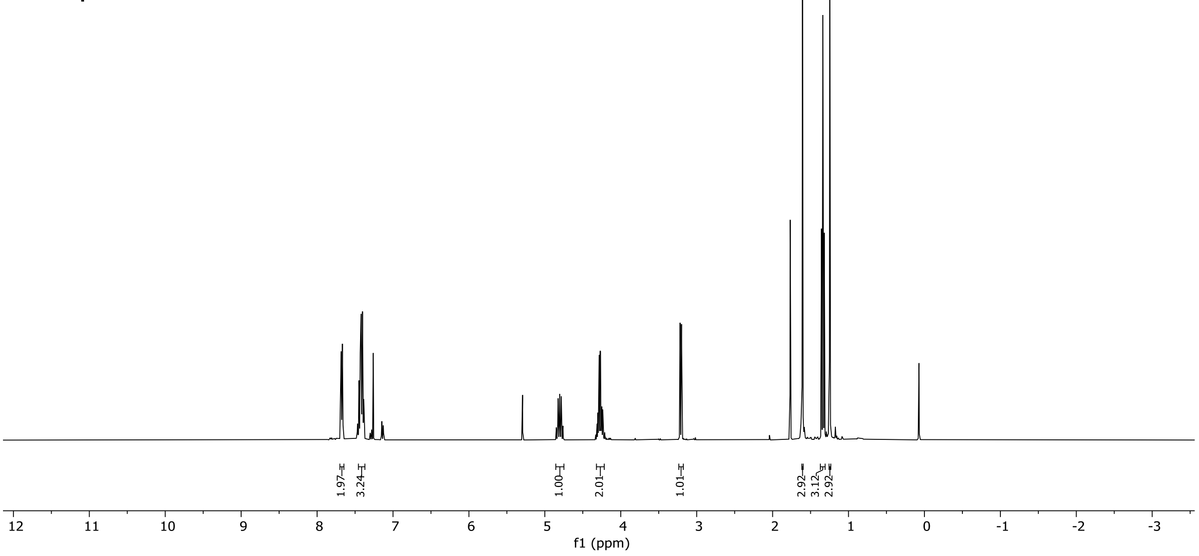

^{13}C NMR (101 MHz, $CDCl_3$) of **3q**

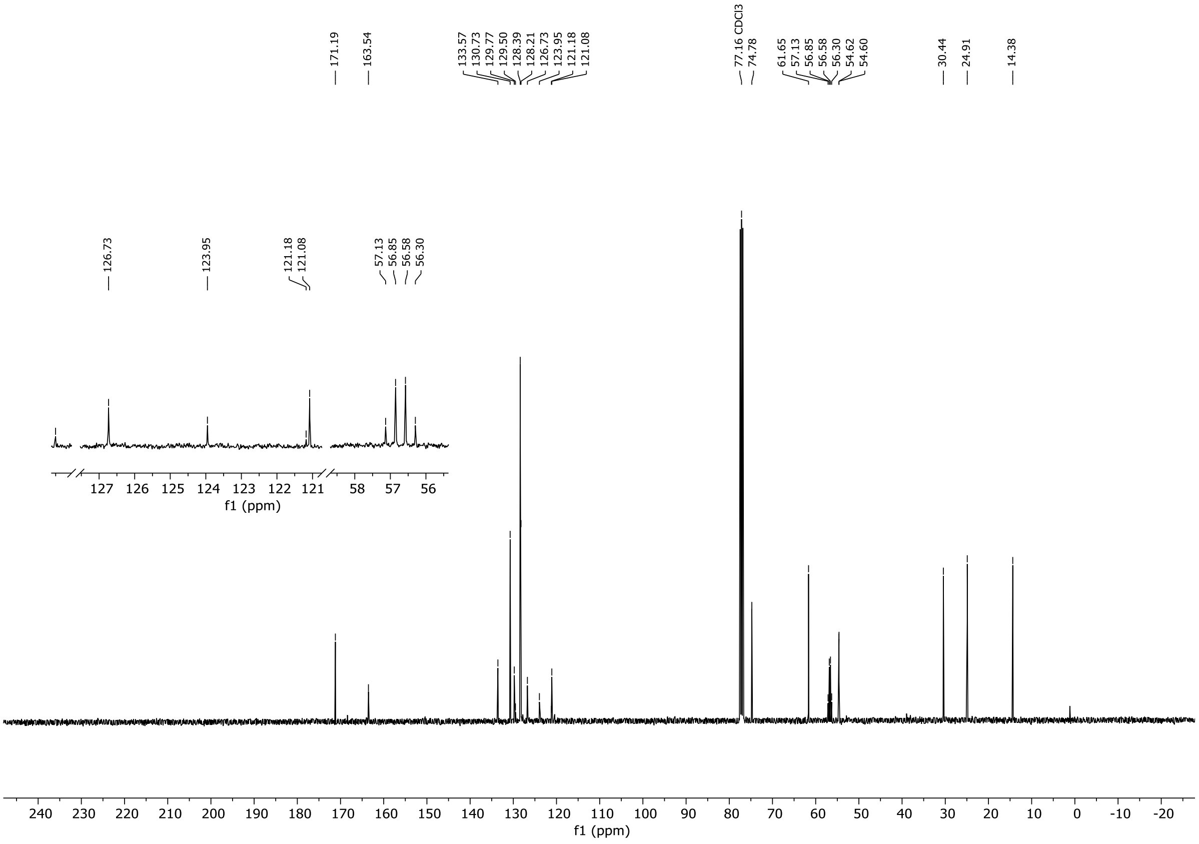

^{13}C NMR {^{1}H, ^{19}F} (126 MHz, $CDCl_3$) of **3q**

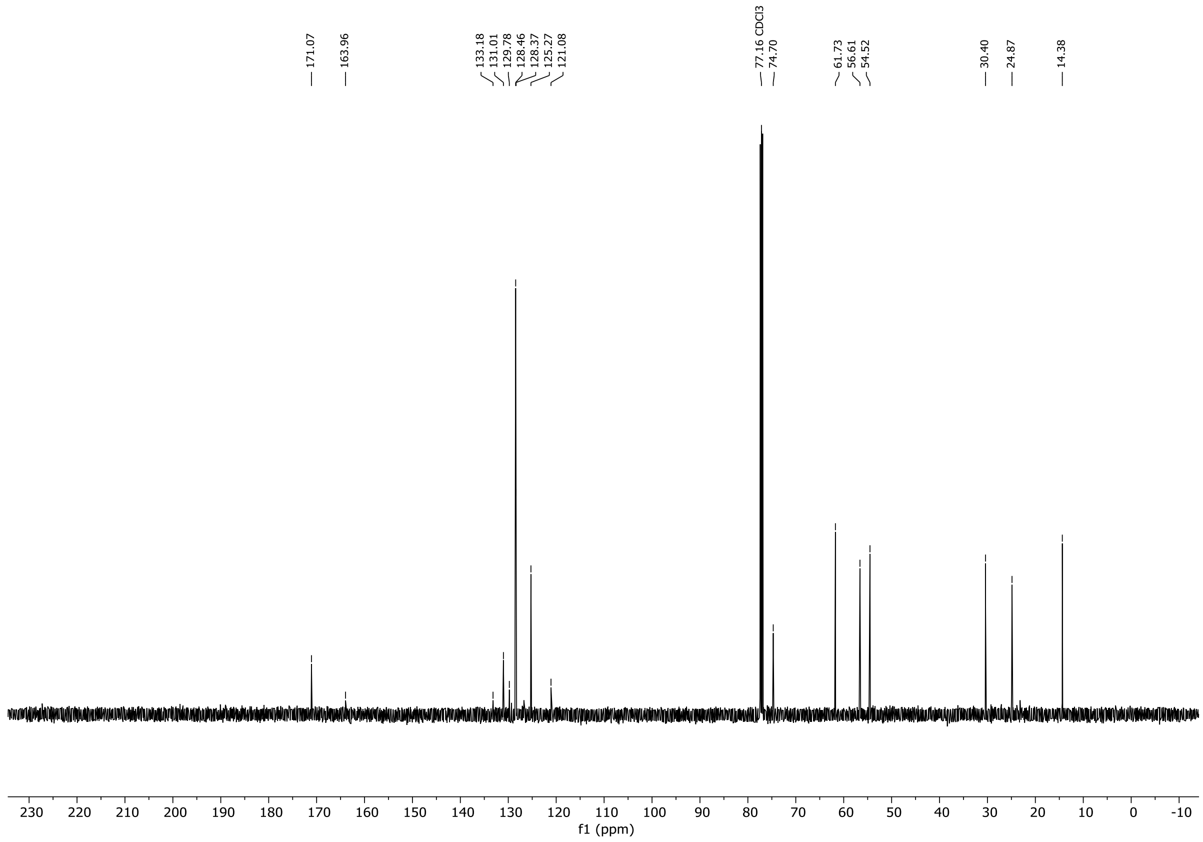

^{19}F NMR (376 MHz, $CDCl_3$) of **3q**

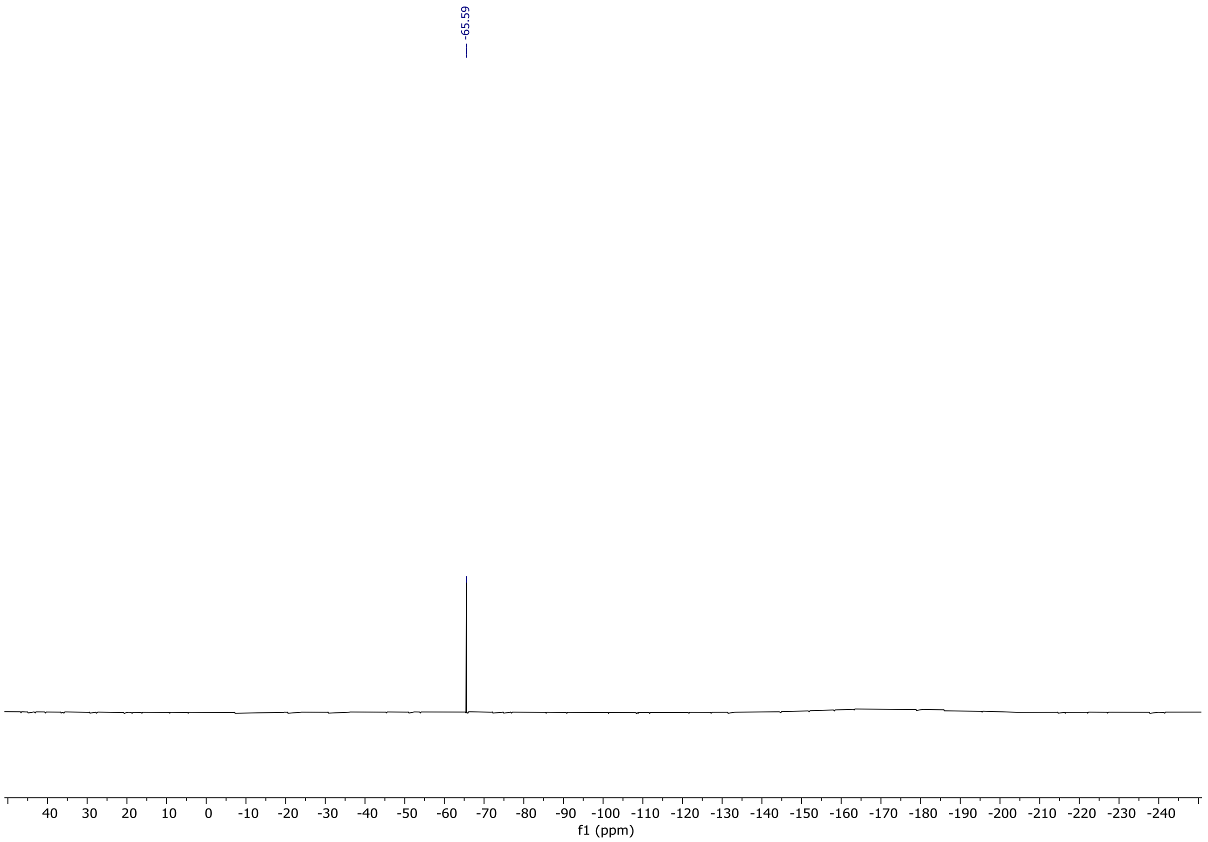

^{1}H NMR (400 MHz, $CDCl_3$) of **3s** (*see procedure*)

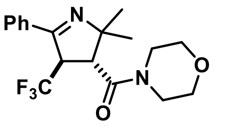

3s

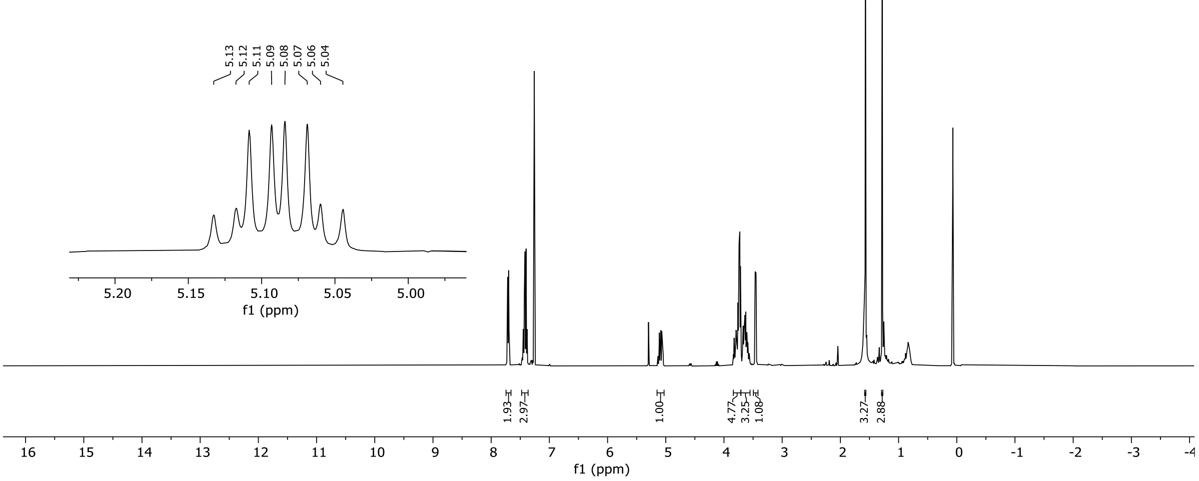

^{13}C NMR (101 MHz, $CDCl_3$) of **3s**

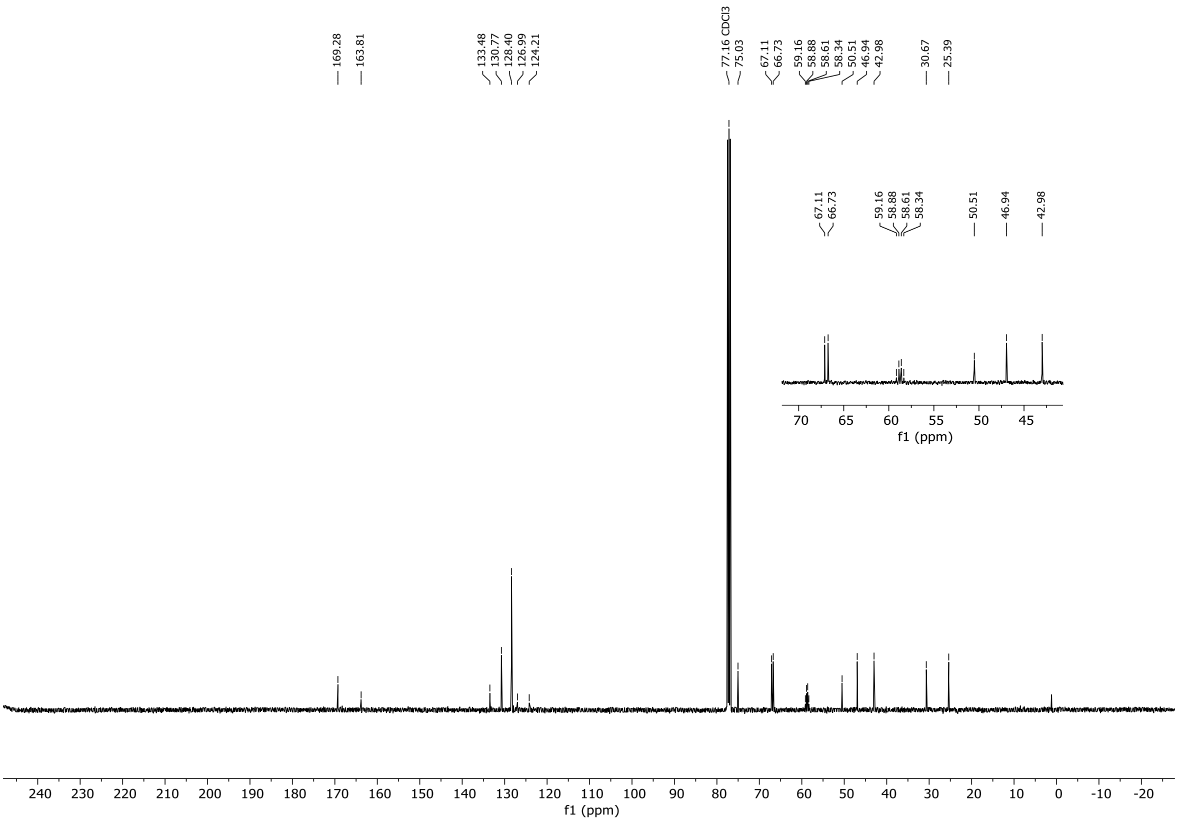

^{13}C NMR {^{1}H, ^{19}F} (126 MHz, $CDCl_3$) of **3s**

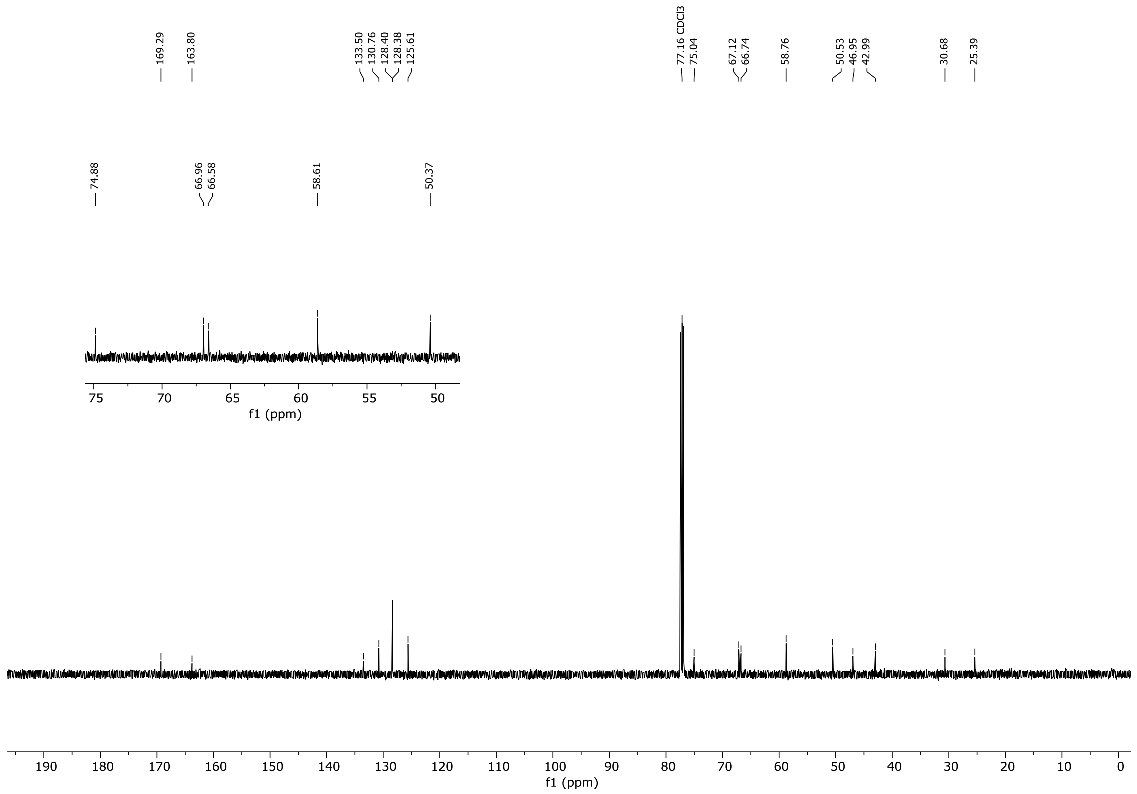

^{19}F NMR (376 MHz, $CDCl_3$) of **3s**

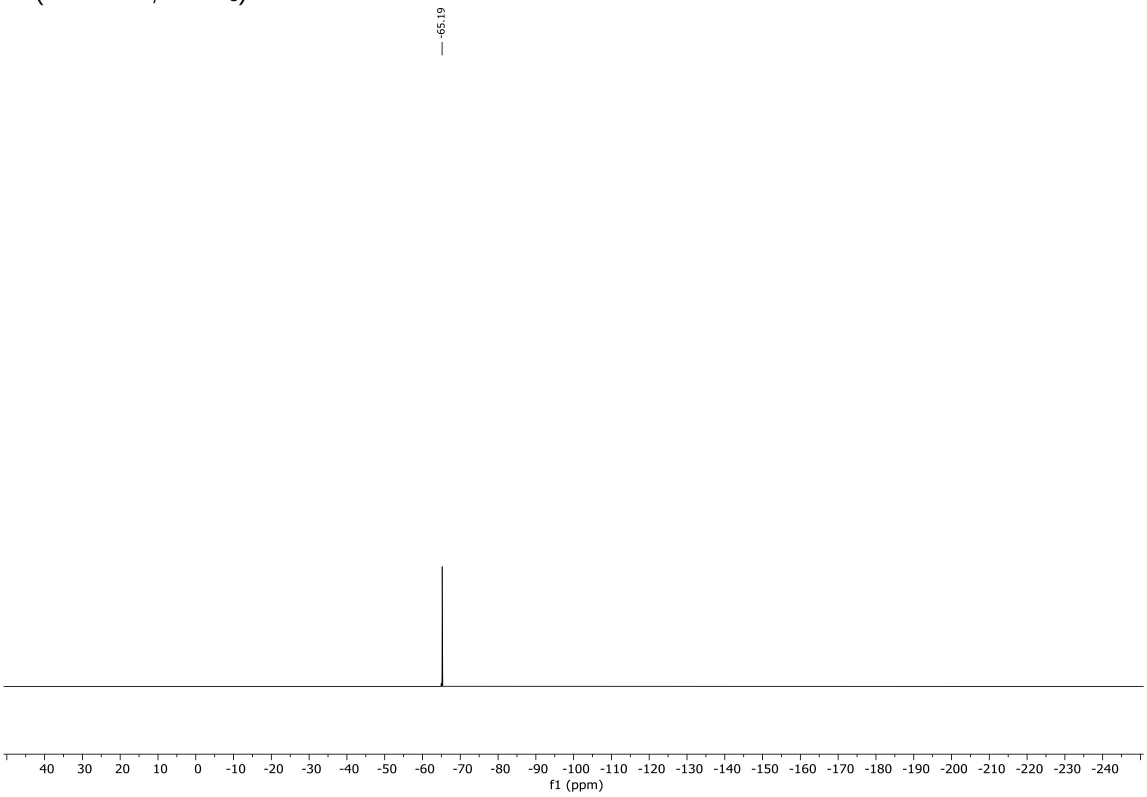

^{1}H NMR (400 MHz, $CDCl_3$) of **3t** (*see procedure*)

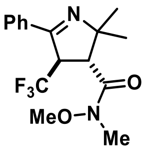

3t

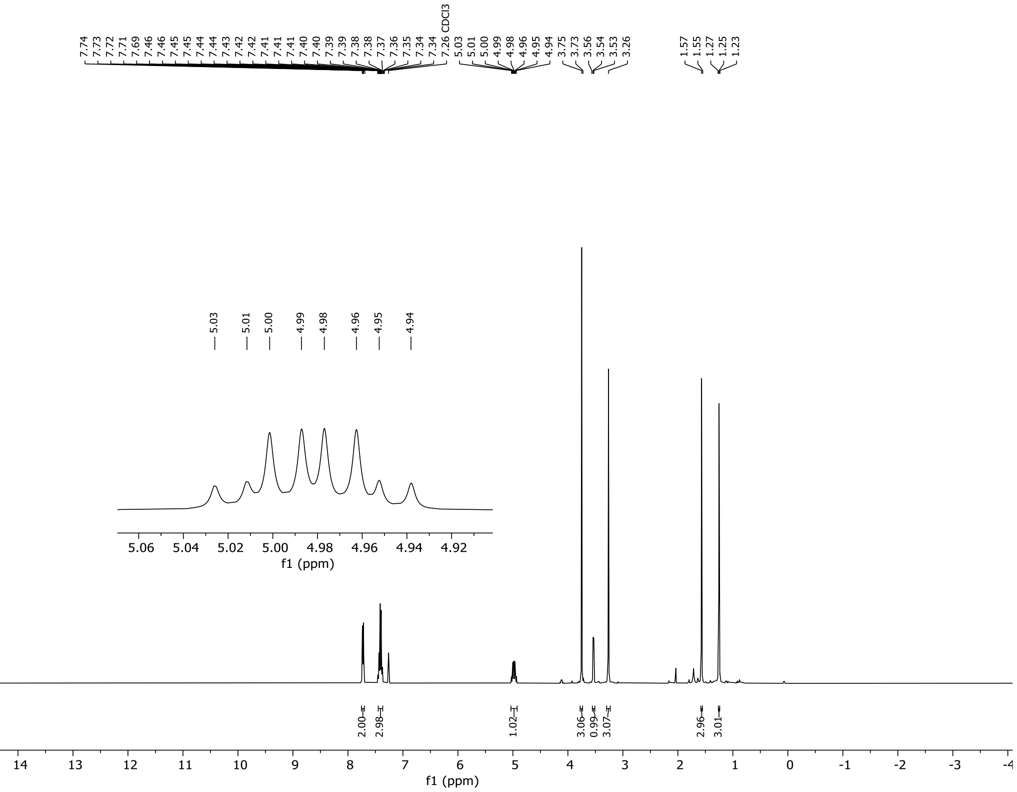

^{13}C NMR (126 MHz, $CDCl_3$) of **3t**

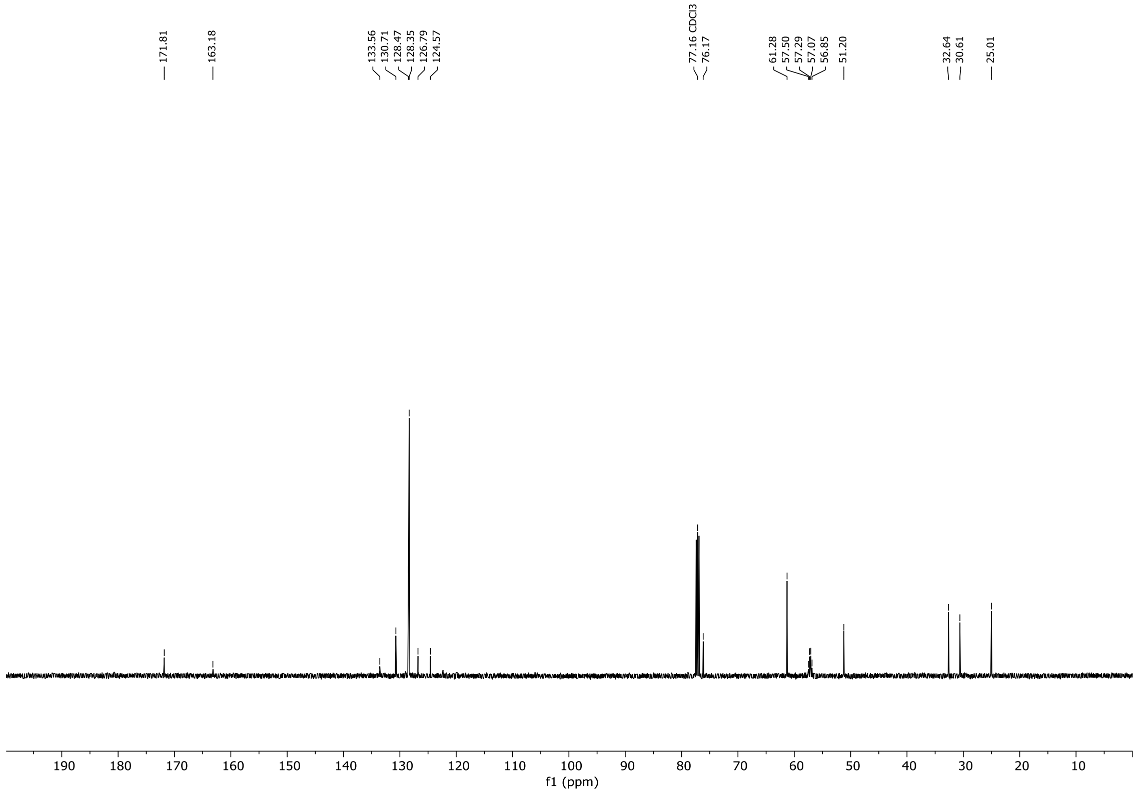

^{13}C NMR {^{1}H, ^{19}F} (126 MHz, $CDCl_3$) of **3t**

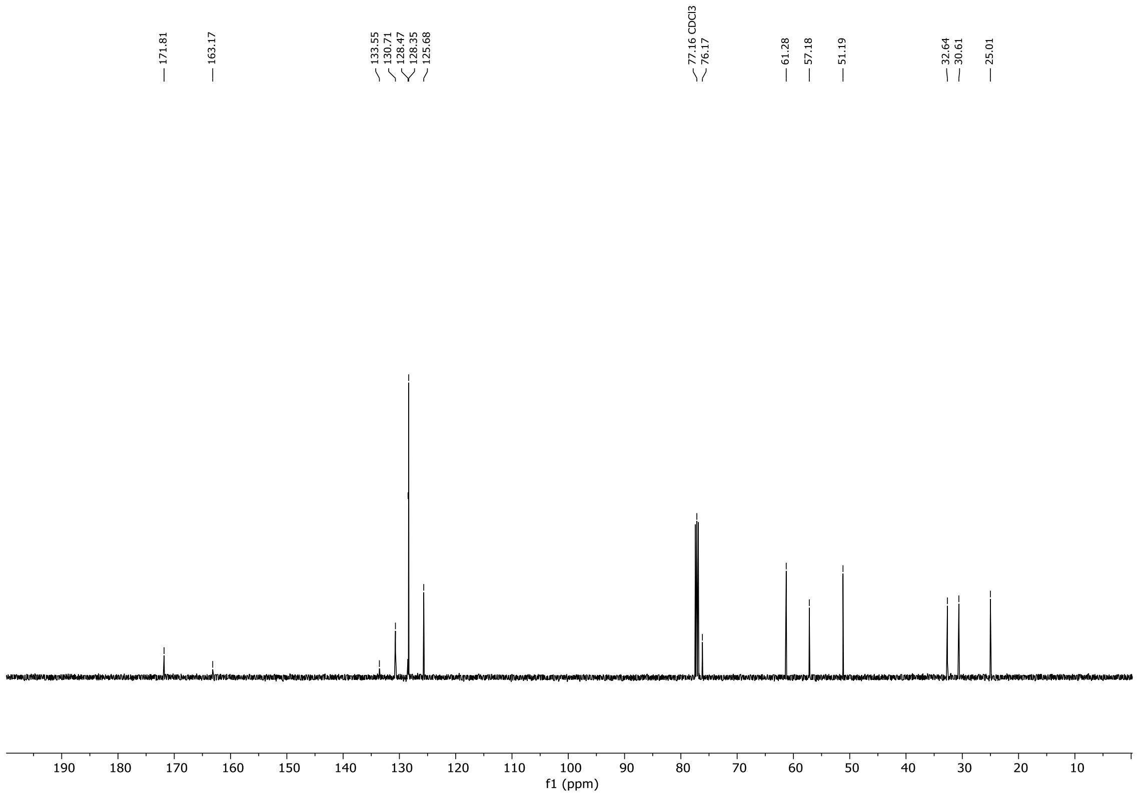

^{19}F NMR (377 MHz, $CDCl_3$) of **3t**

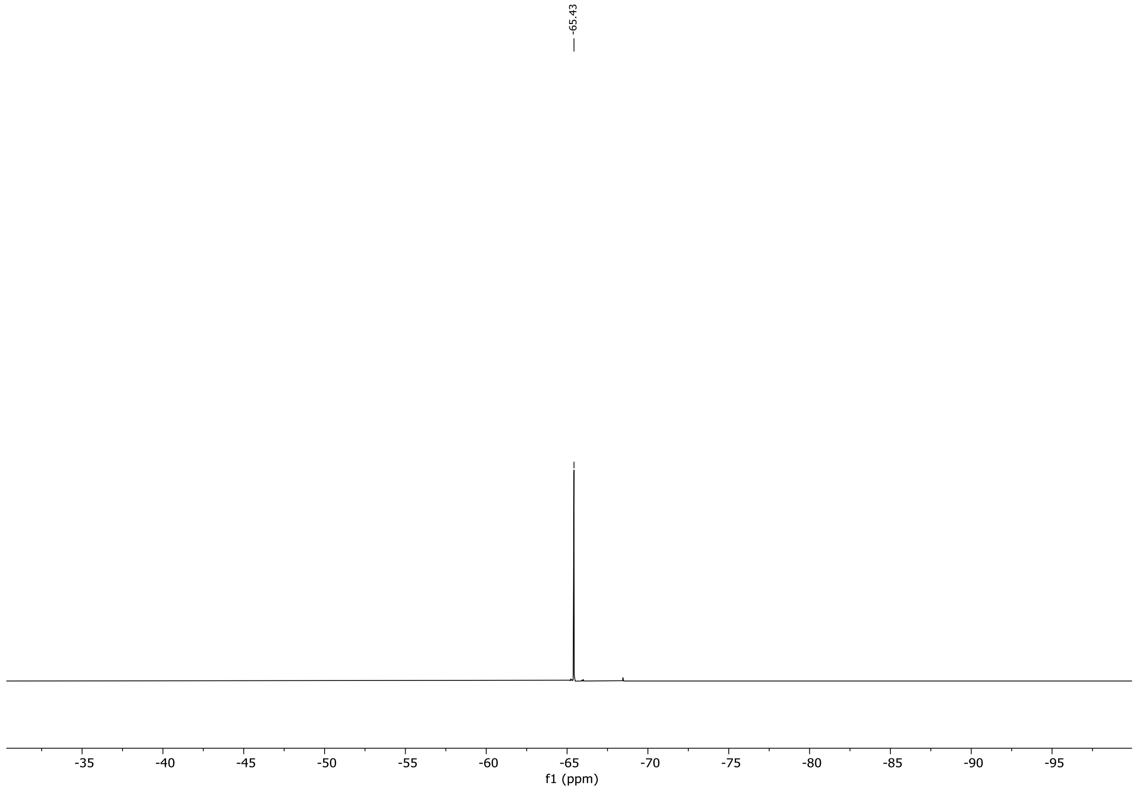

^{1}H NMR (400 MHz, $CDCl_3$) of **3u** (*see procedure*)

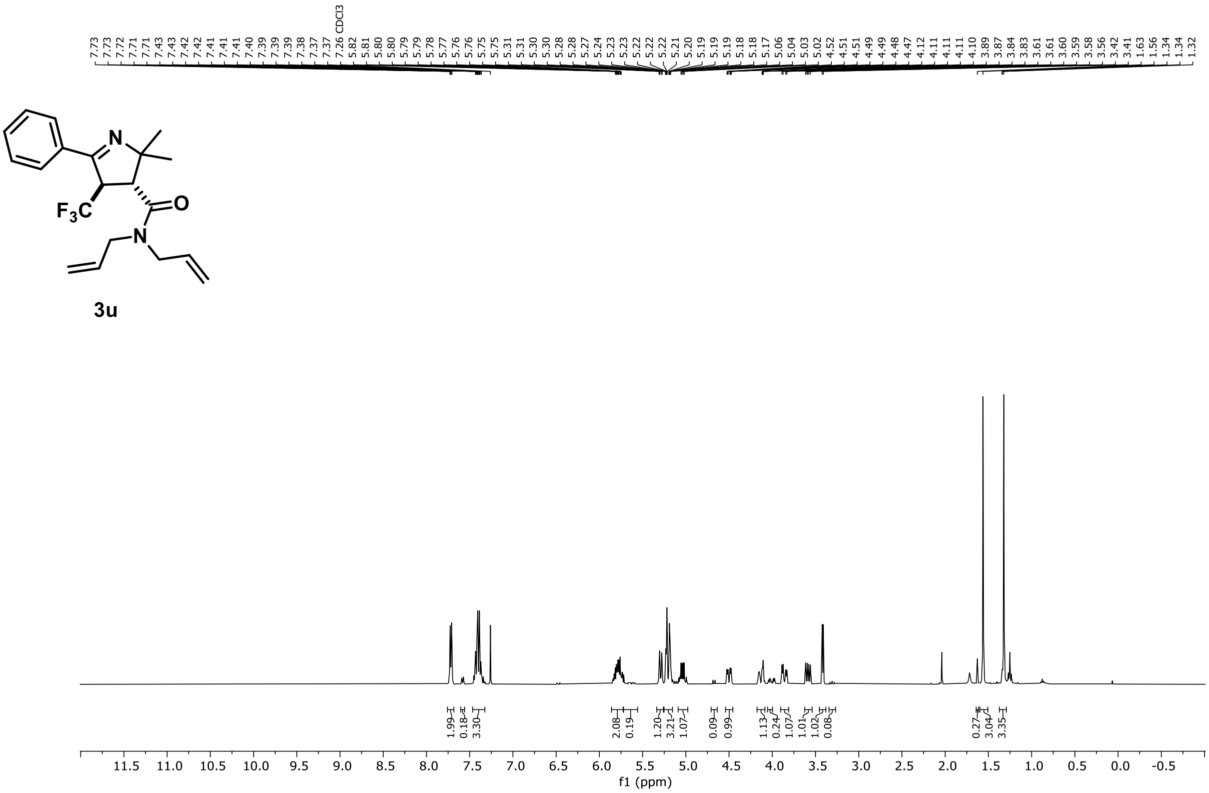

^{13}C NMR (126 MHz, $CDCl_3$) of **3u**

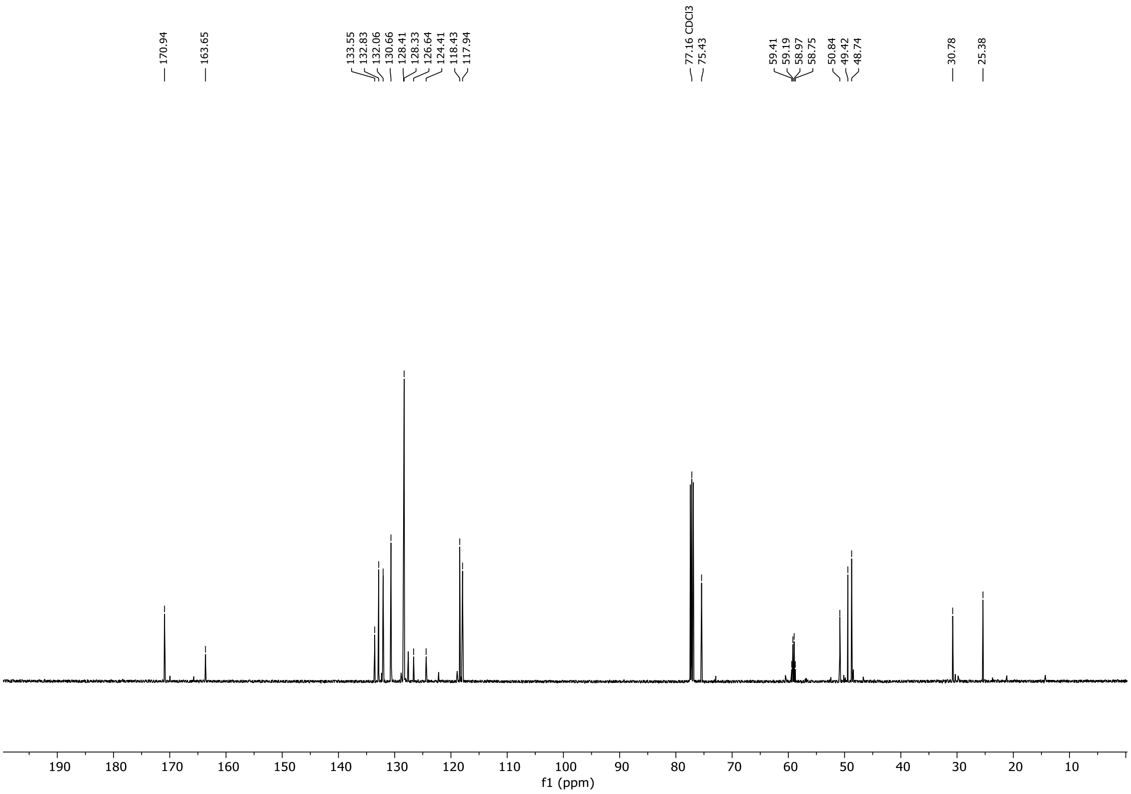

^{13}C NMR {^{1}H, ^{19}F} (126 MHz, $CDCl_3$) of **3u**

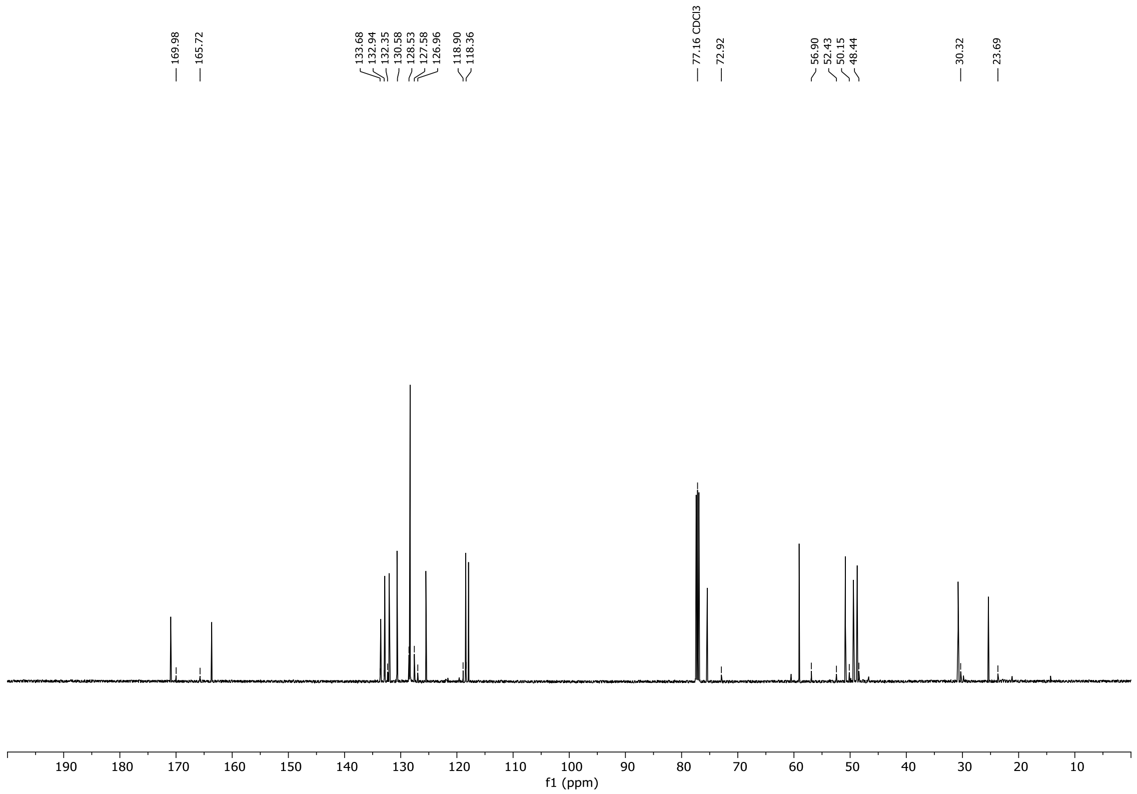

^{19}F NMR (377 MHz, $CDCl_3$) of **3u**

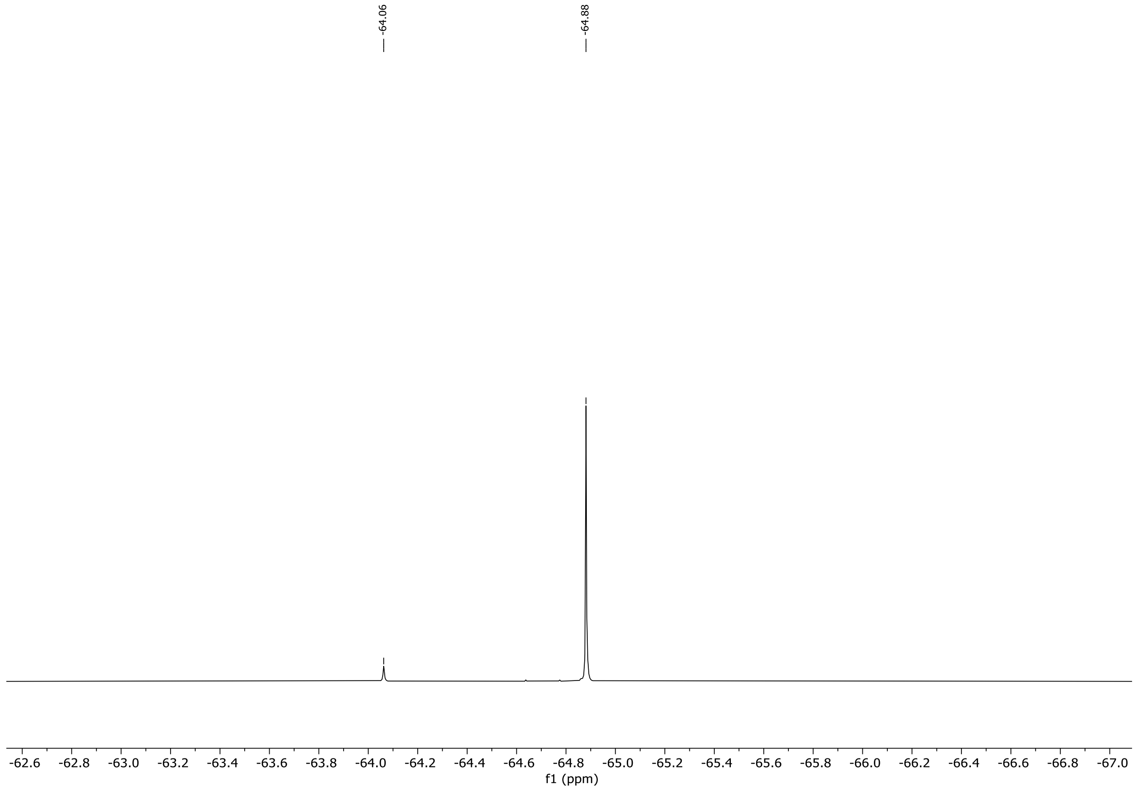

^{1}H NMR (400 MHz, $CDCl_3$) of **3w** (*see procedure*)

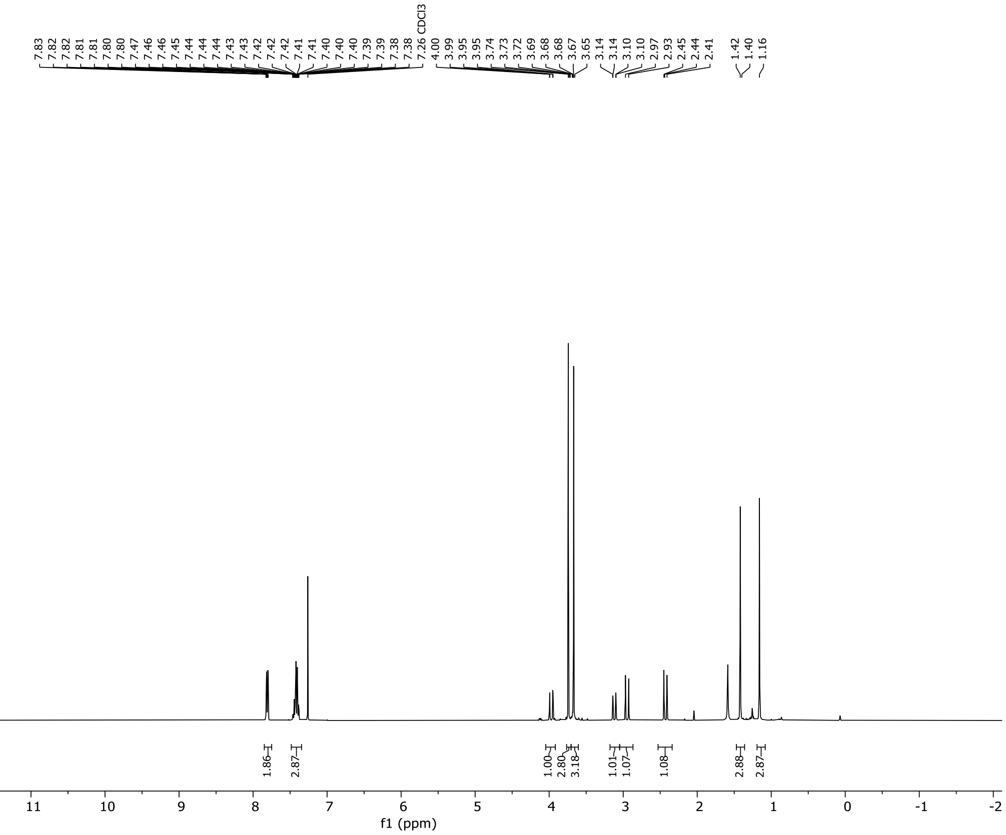

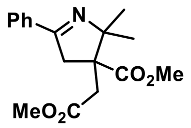

3w

^{13}C NMR (101 MHz, $CDCl_3$) of **3w**

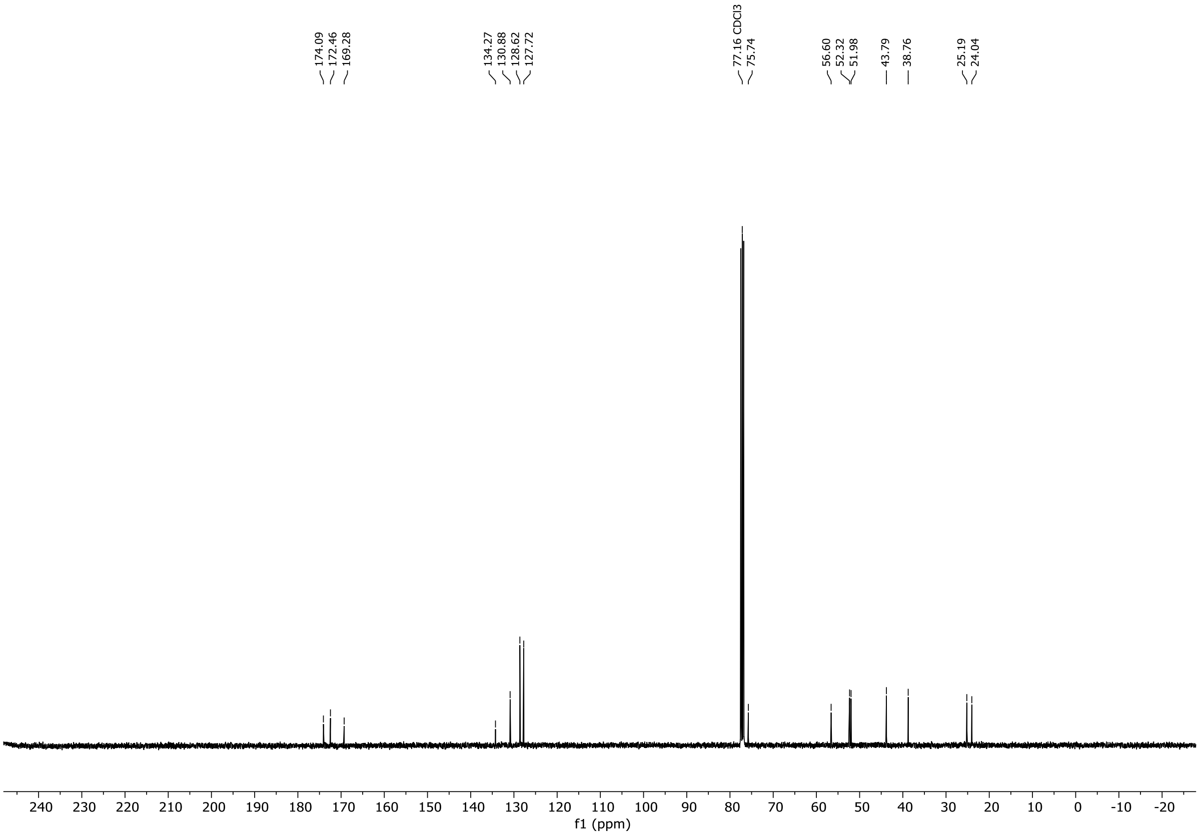

^{1}H NMR (400 MHz, $CDCl_3$) of **3x** (*see procedure*)

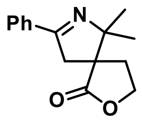

3x

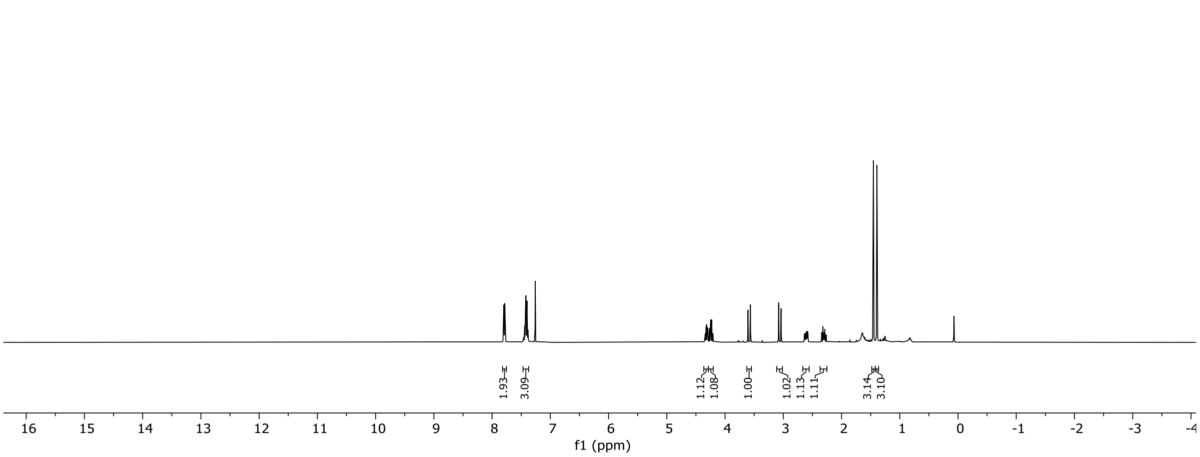

^{13}C NMR (101 MHz, $CDCl_3$) of **3x**

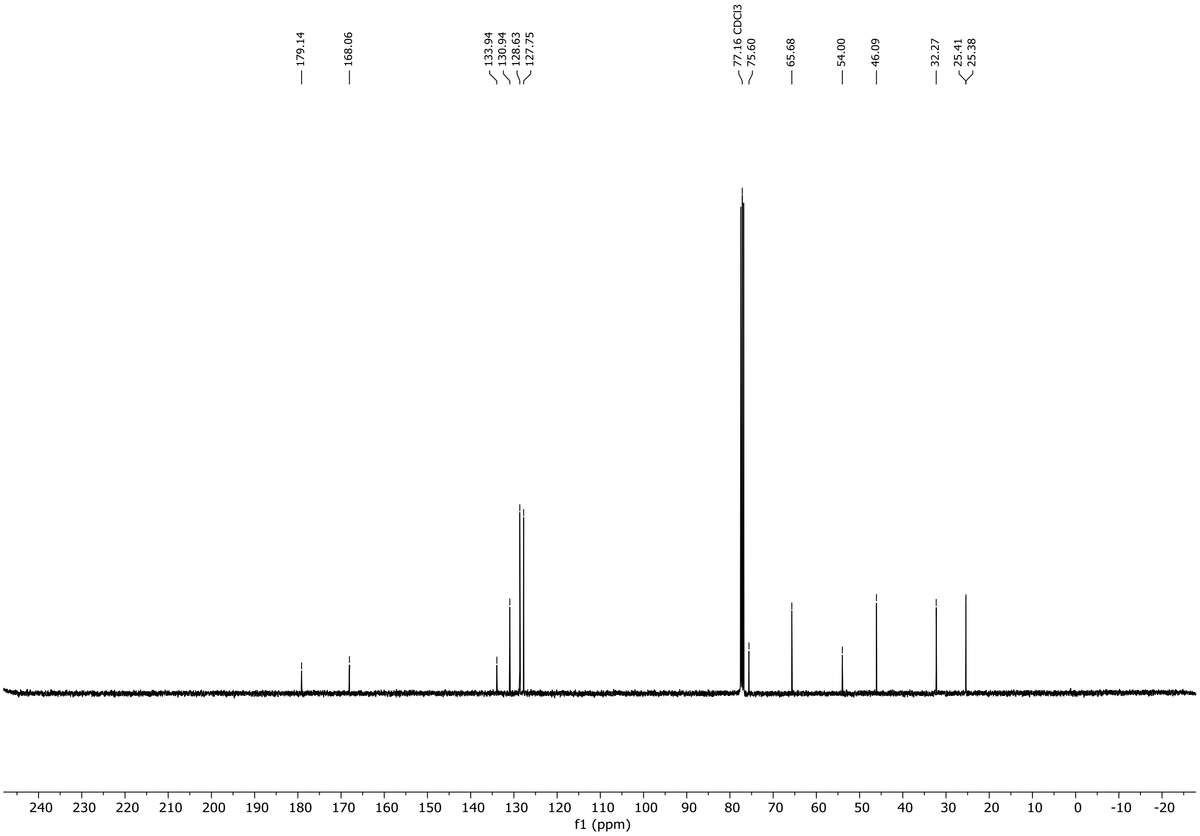

^{1}H NMR (400 MHz, $CDCl_3$) of **3y** (see procedure)

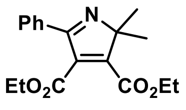

3y

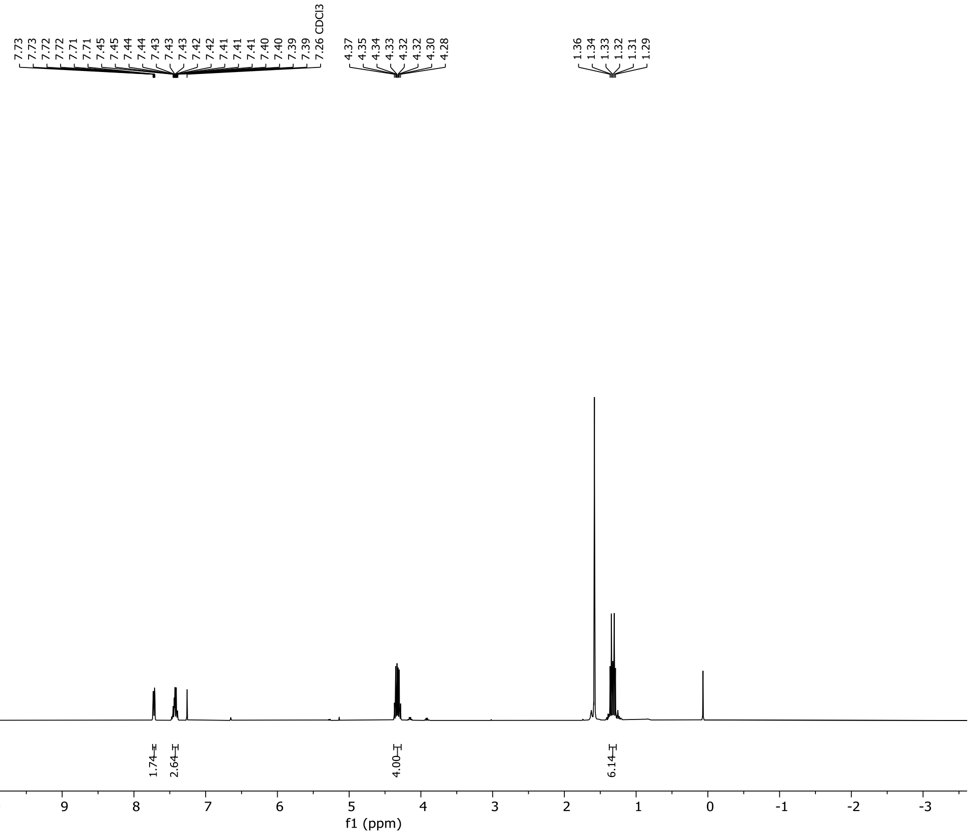

^{13}C NMR (101 MHz, $CDCl_3$) of **3y**

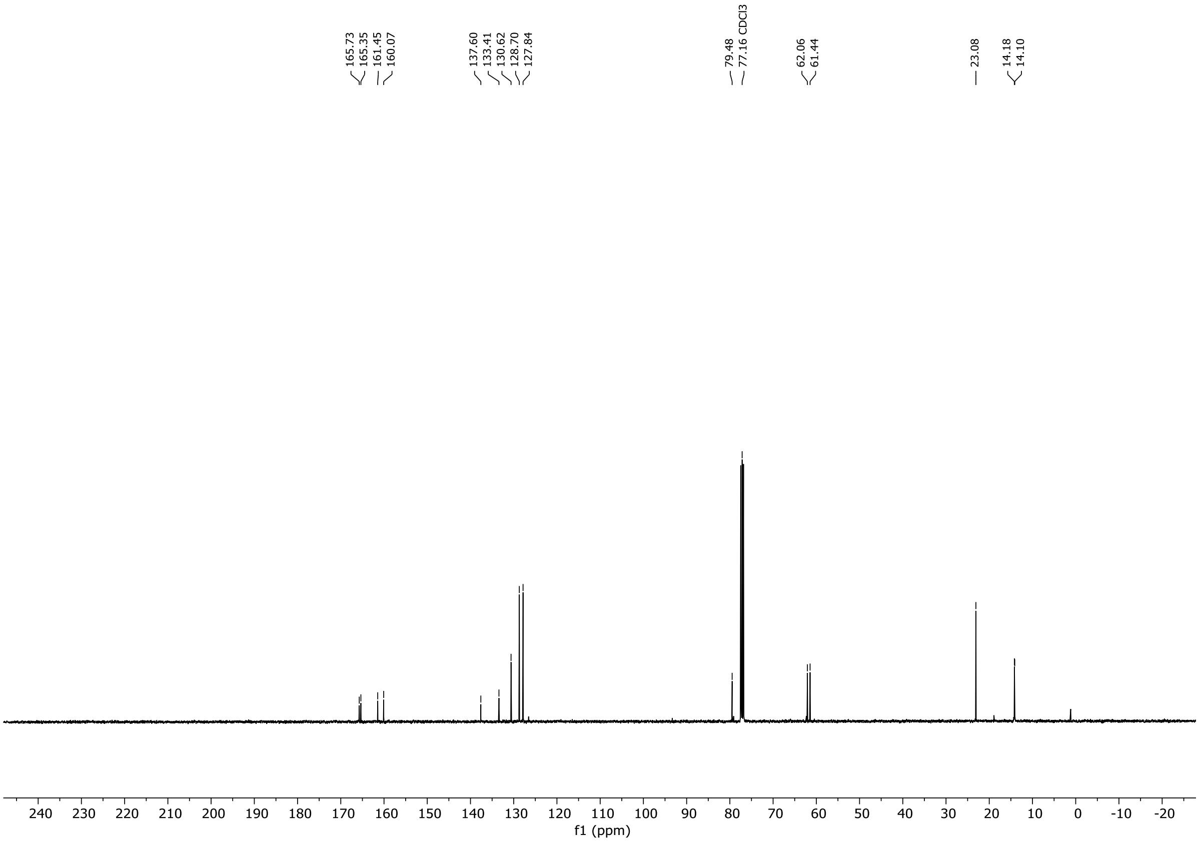

^{1}H NMR (400 MHz, CDCl$_3$) of **3z** (*see procedure*)

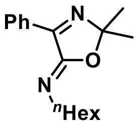

3z

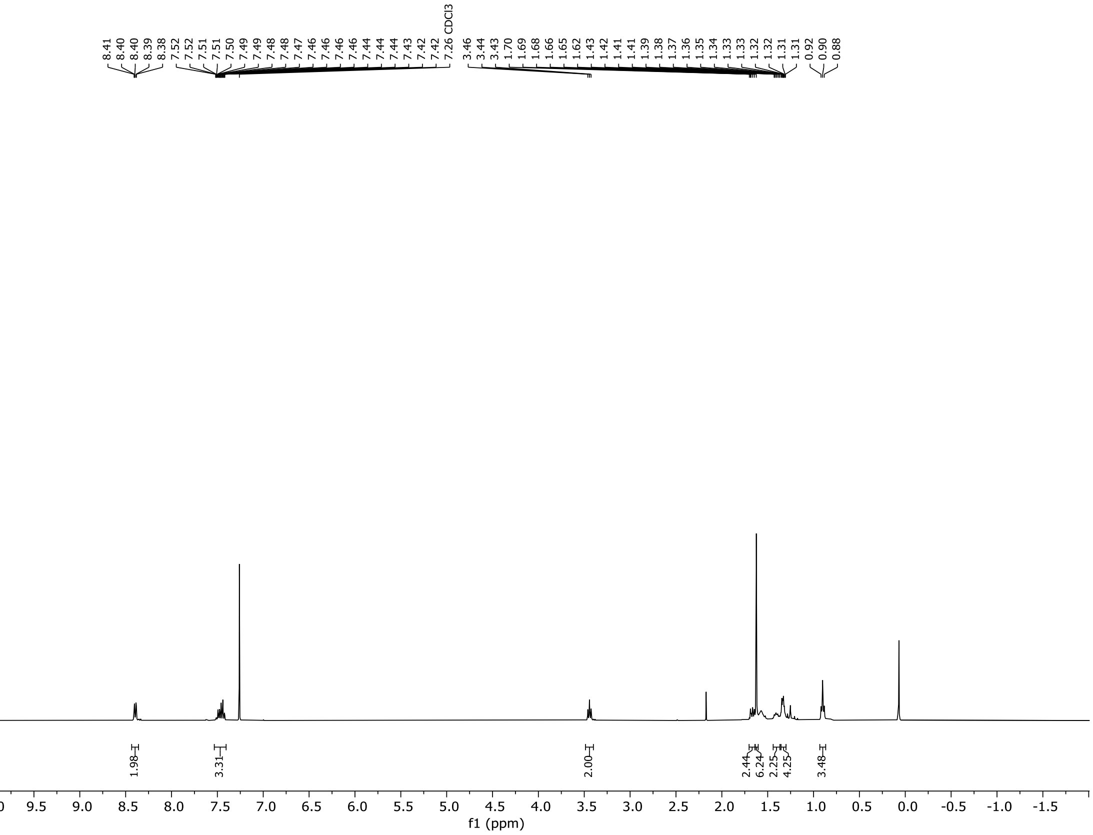

^{13}C NMR (101 MHz, CDCl$_3$) of **3z**

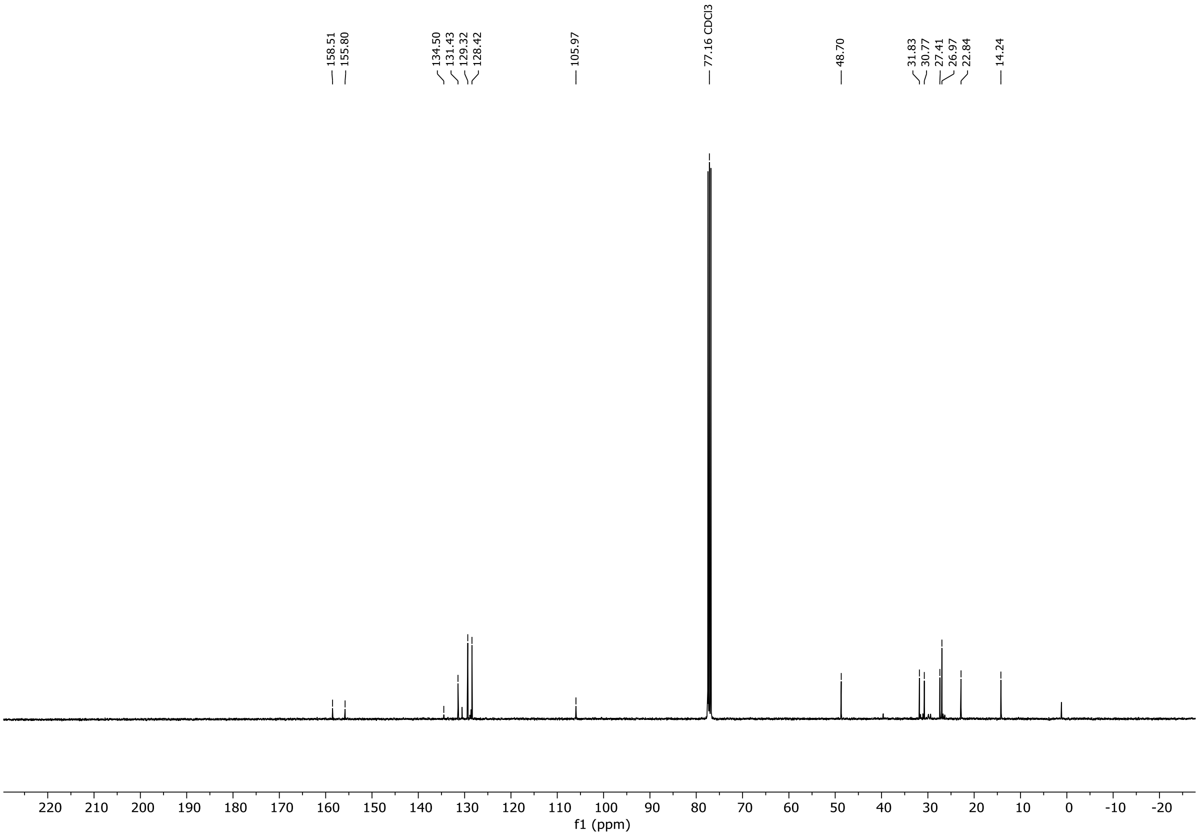

^{1}H NMR (400 MHz, $CDCl_3$) of **3aa** (see procedure)

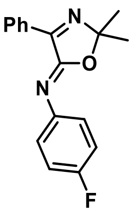

3aa

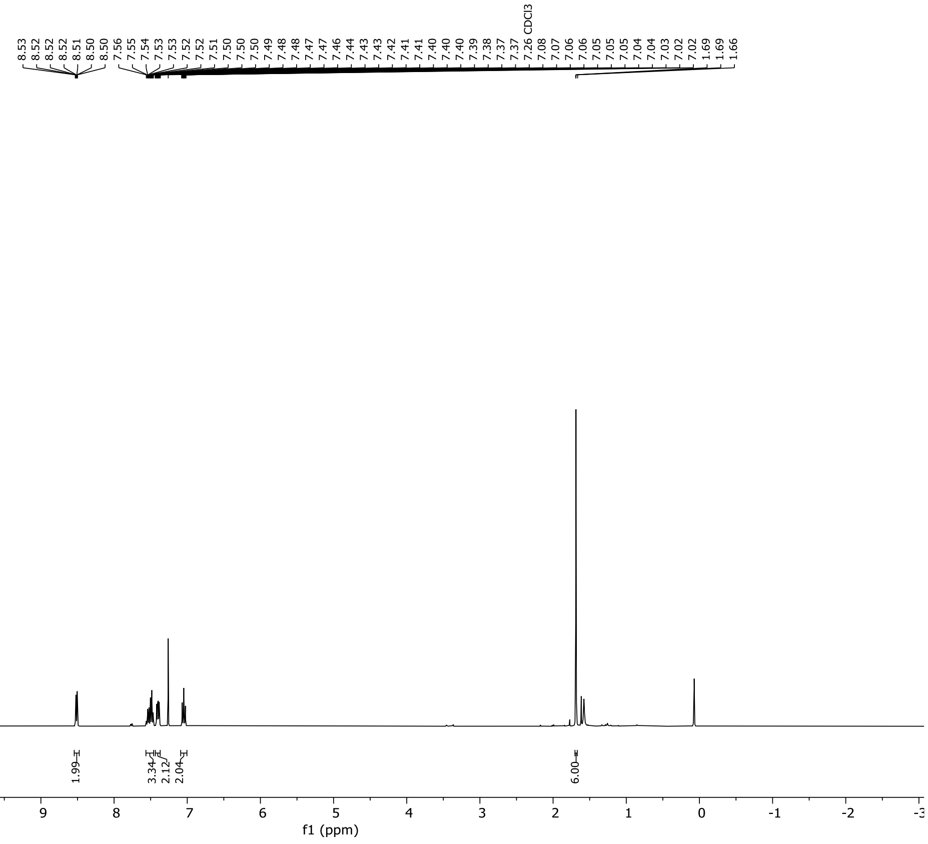

^{13}C NMR (101 MHz, $CDCl_3$) of **3aa**

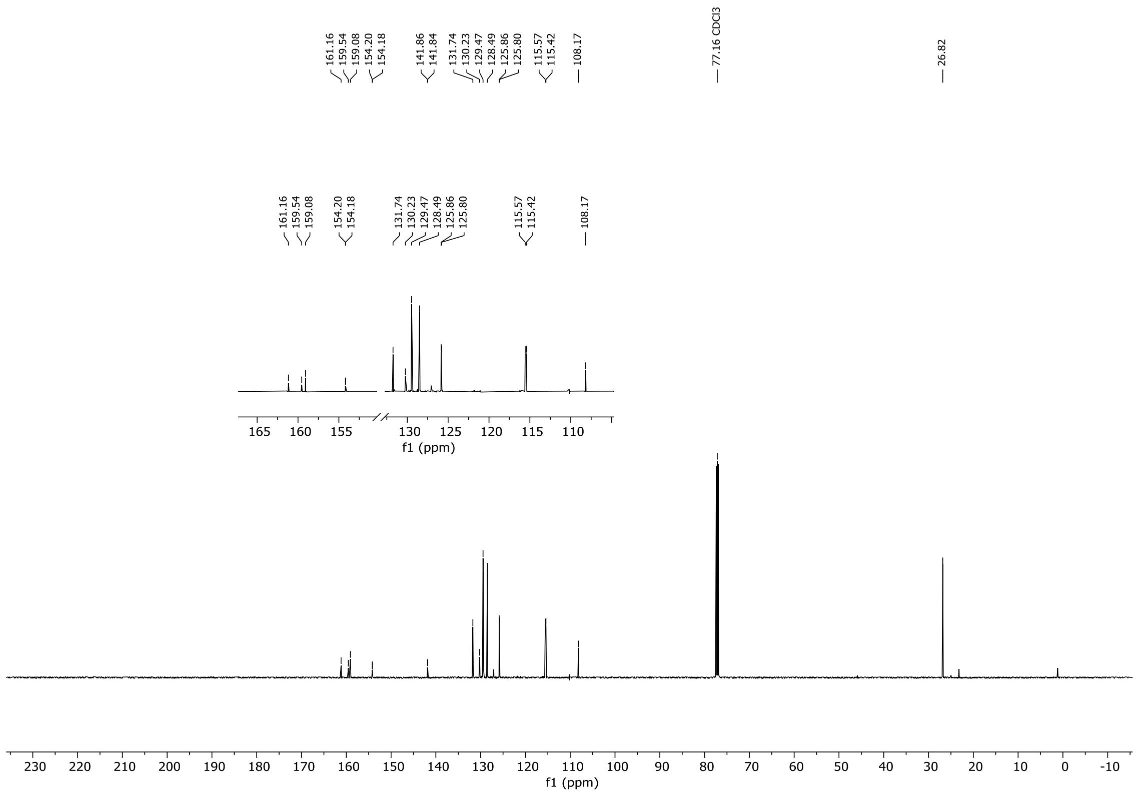

^{13}C NMR {^{1}H, ^{19}F} (126 MHz, $CDCl_3$) of **3aa**

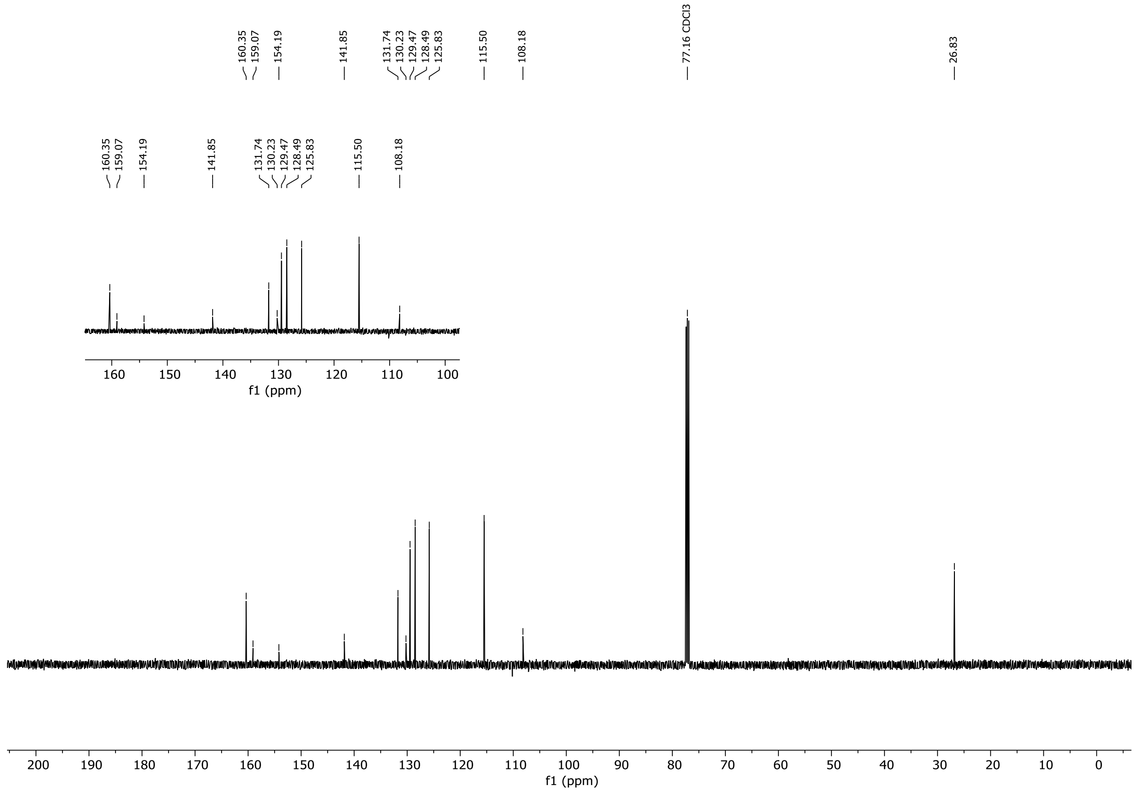

^{19}F NMR (376 MHz, $CDCl_3$) of **3aa**

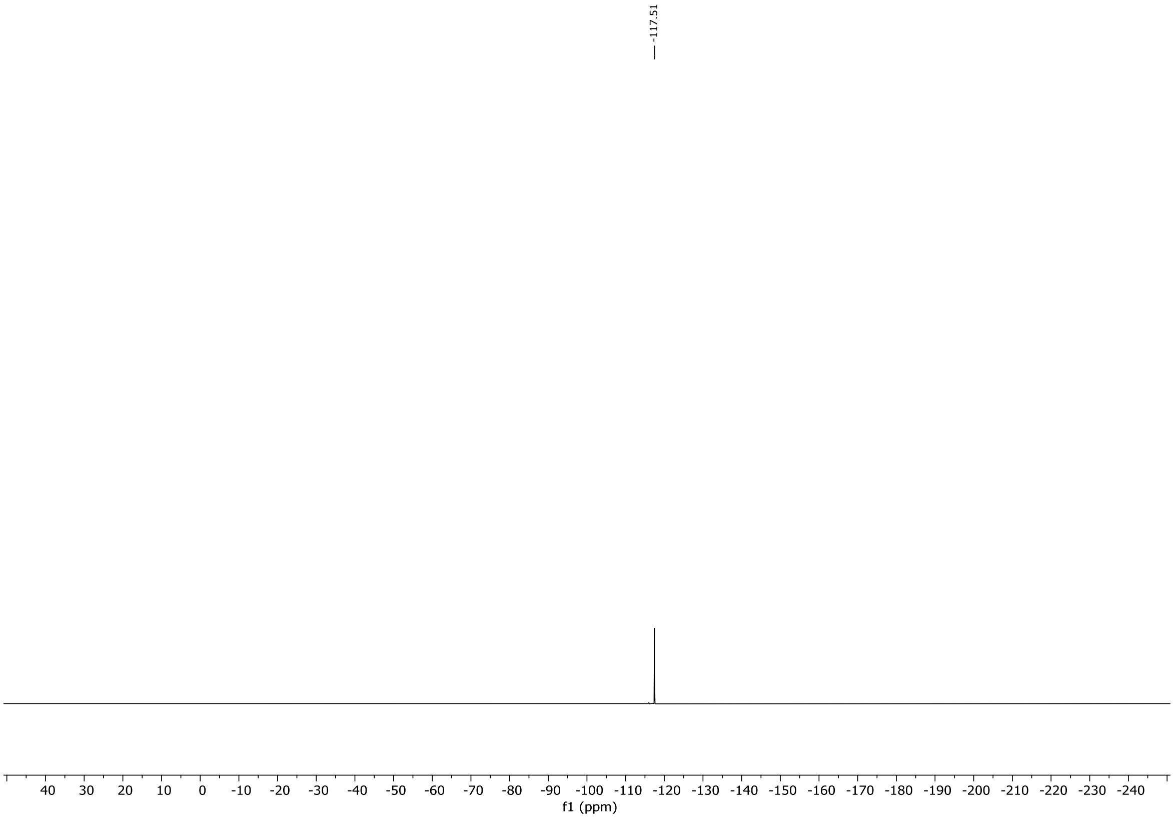

^{1}H NMR (400 MHz, $CDCl_3$) of **3ab** (*see procedure*)

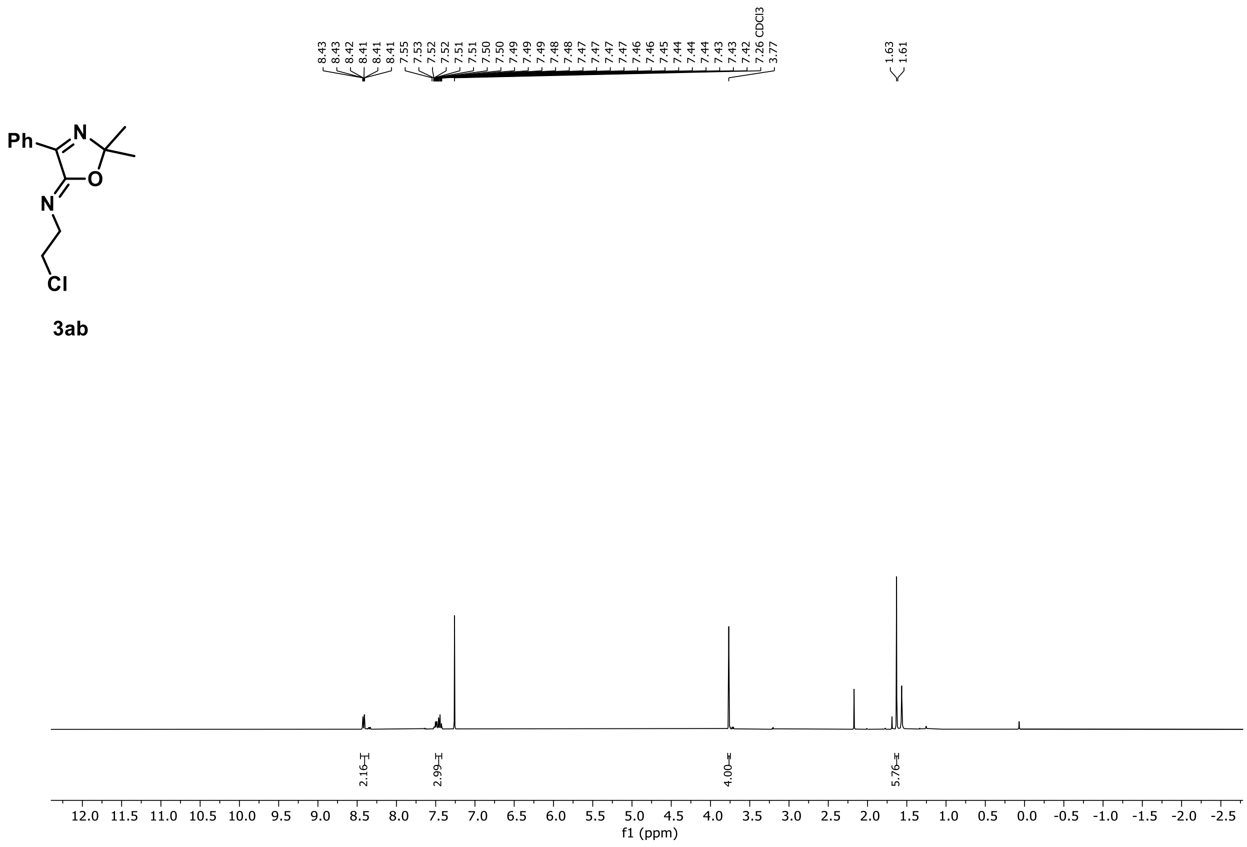

^{13}C NMR (101 MHz, $CDCl_3$) of **3ab**

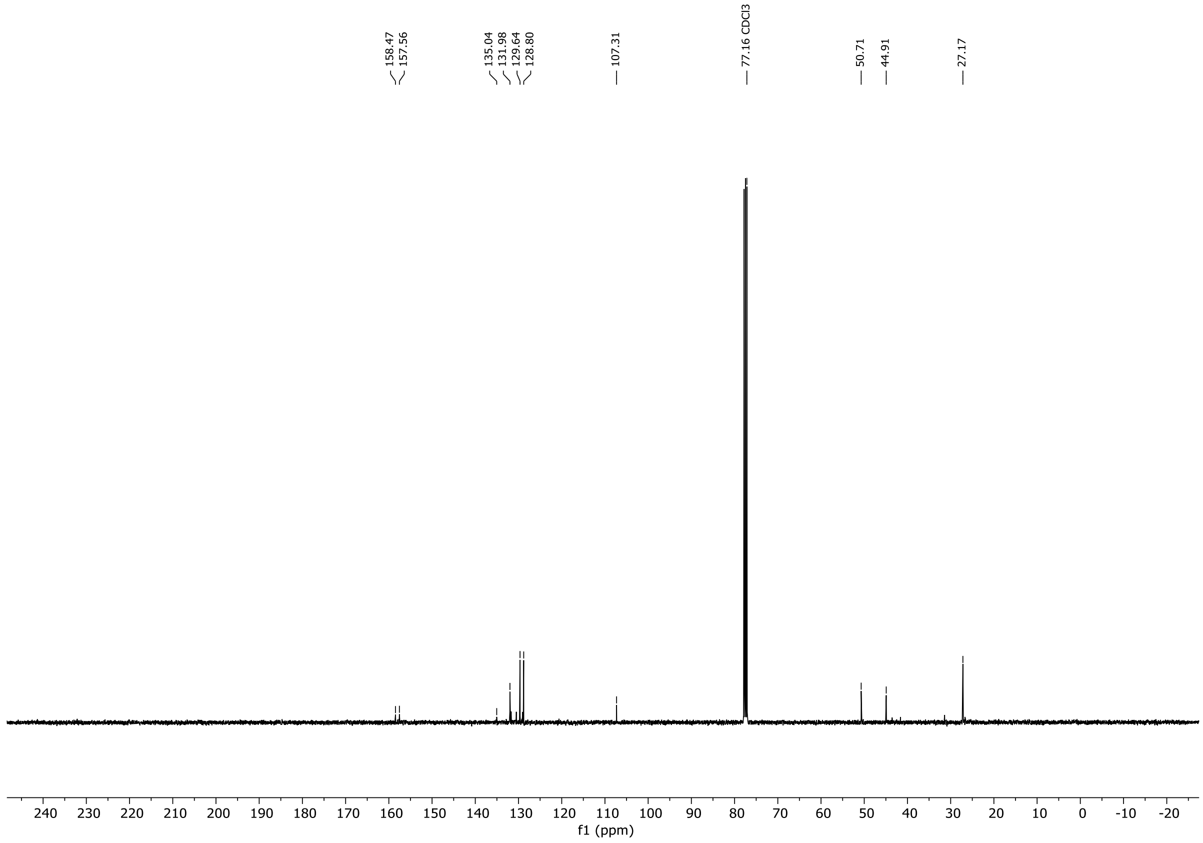

^{1}H NMR (400 MHz, $CDCl_3$) of **3ac** (<u>*see procedure*</u>)

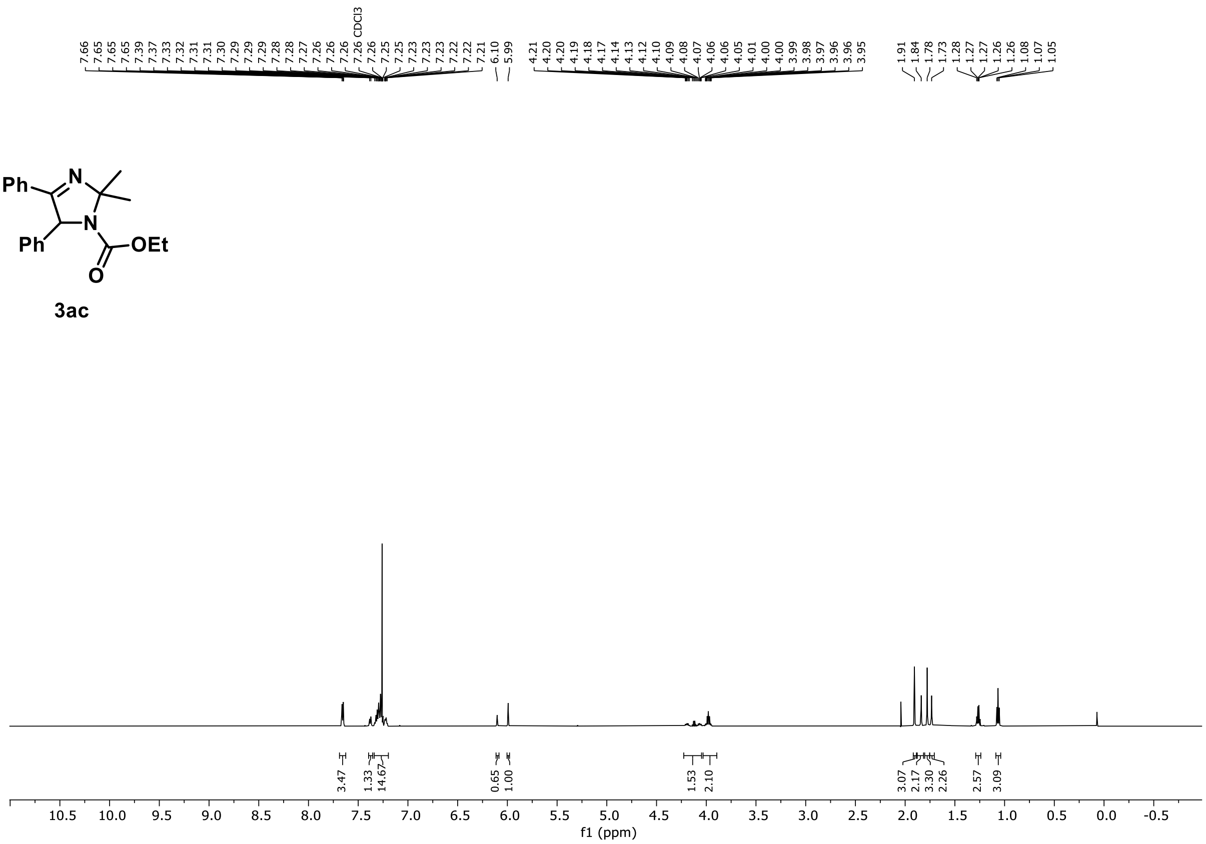

^{13}C NMR (101 MHz, $CDCl_3$) of **3ac**

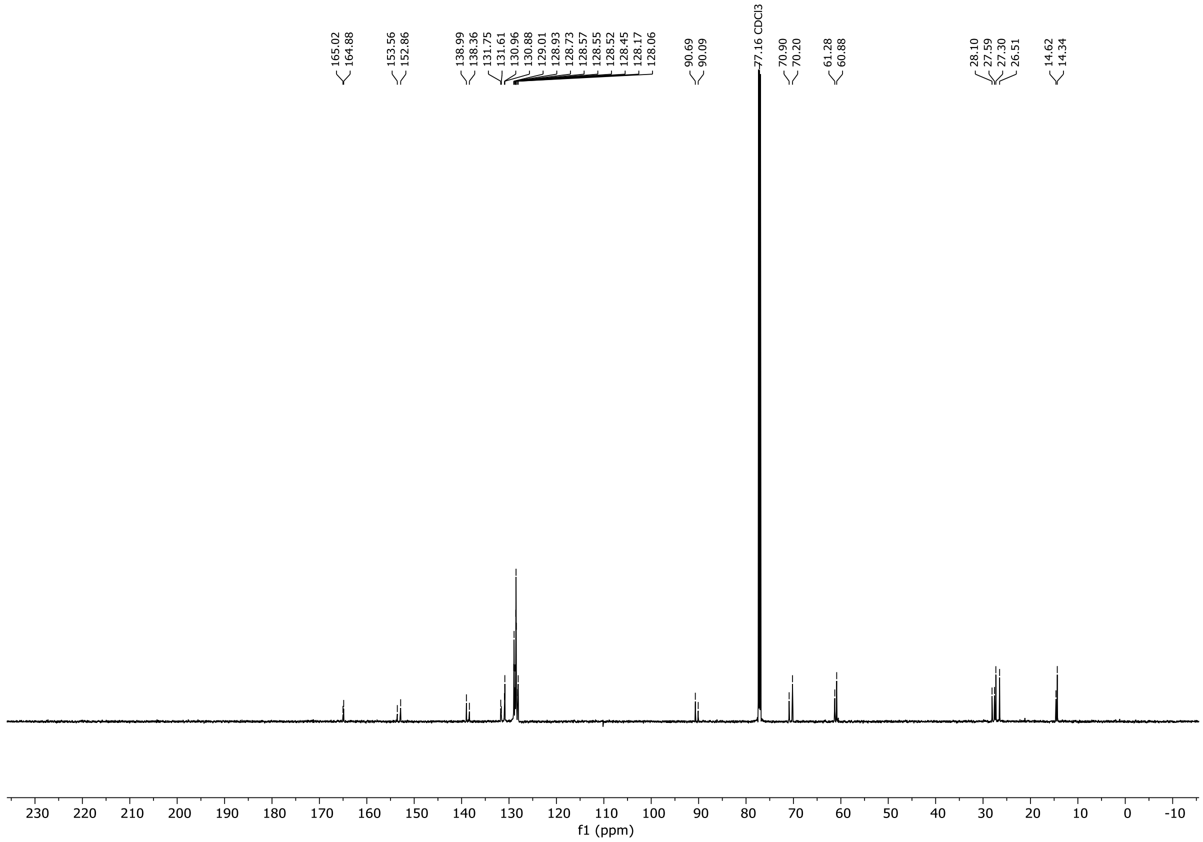

^{1}H NMR (400 MHz, $CDCl_3$) of **3ad** (*see procedure*)

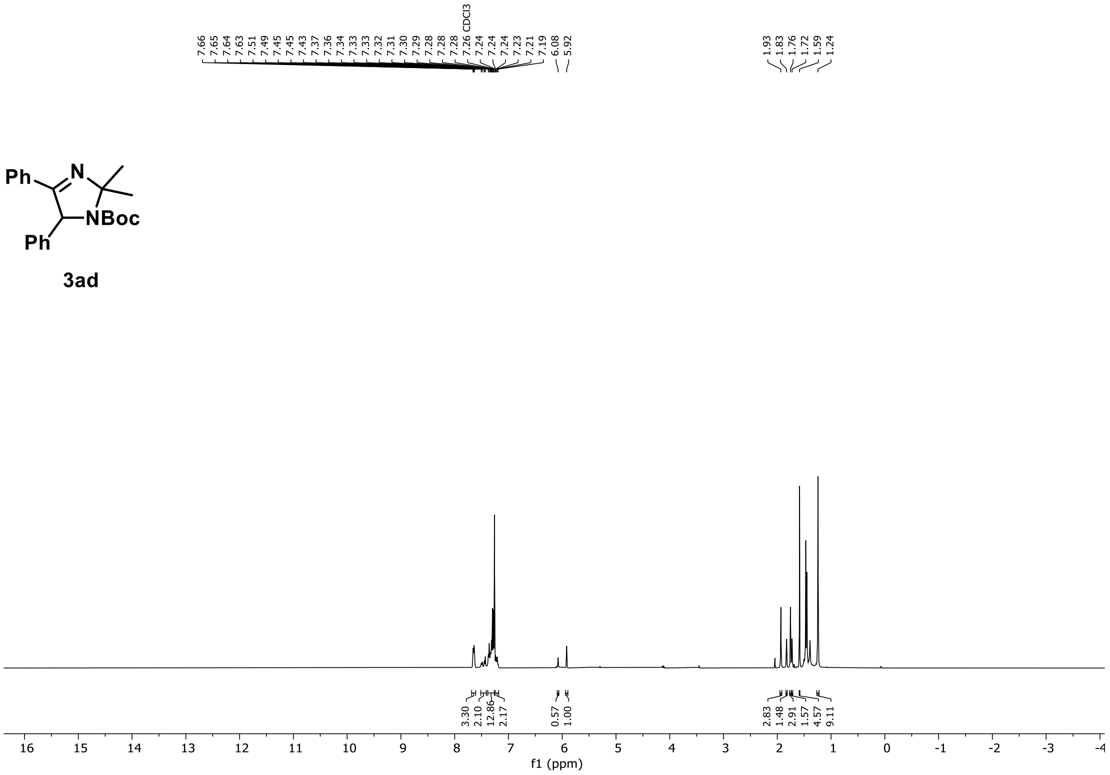

^{13}C NMR (151 MHz, $CDCl_3$) of **3ad** (*see procedure*)

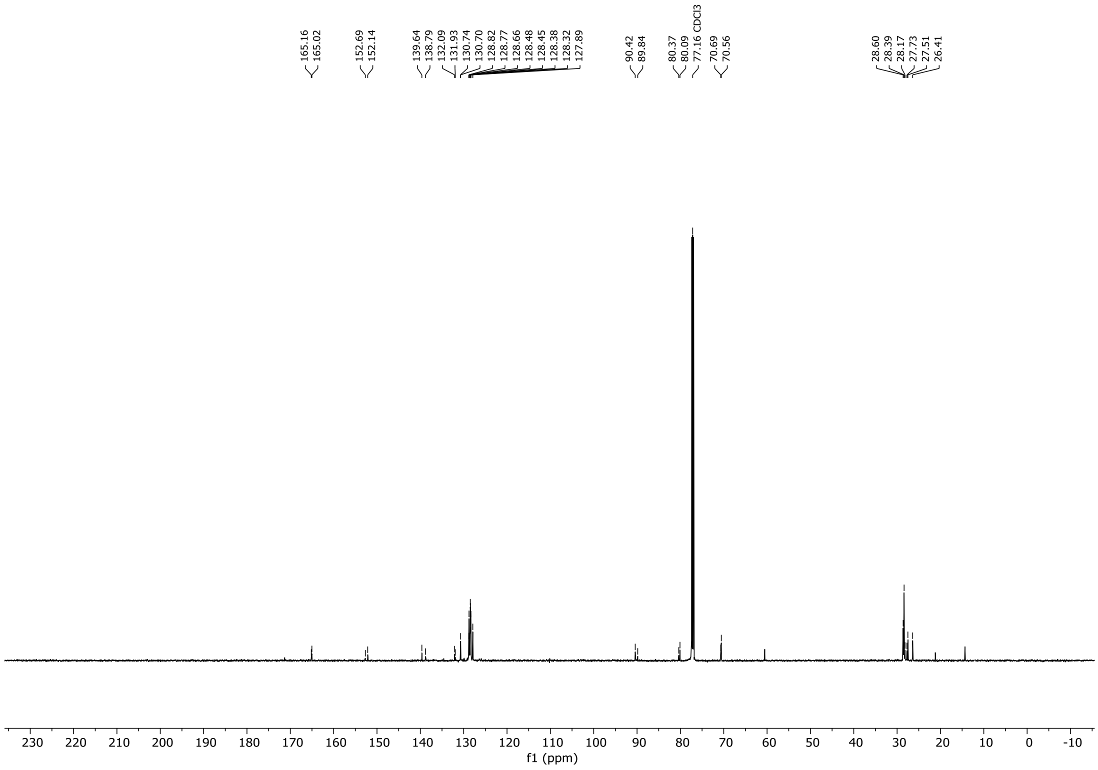

^{1}H NMR (400 MHz, $CDCl_3$) of **3ae** (*see procedure*)

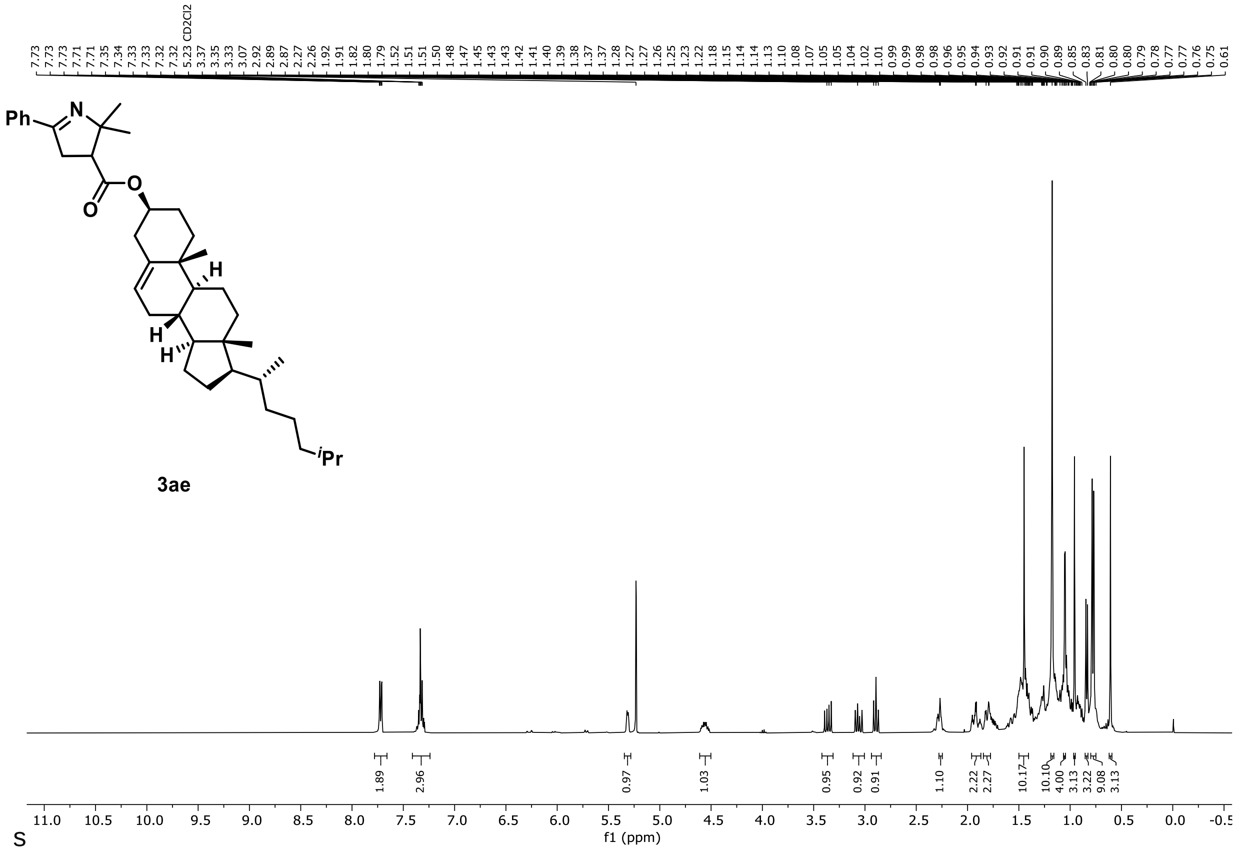

^{13}C NMR (126 MHz, $CDCl_3$) of **3ae**

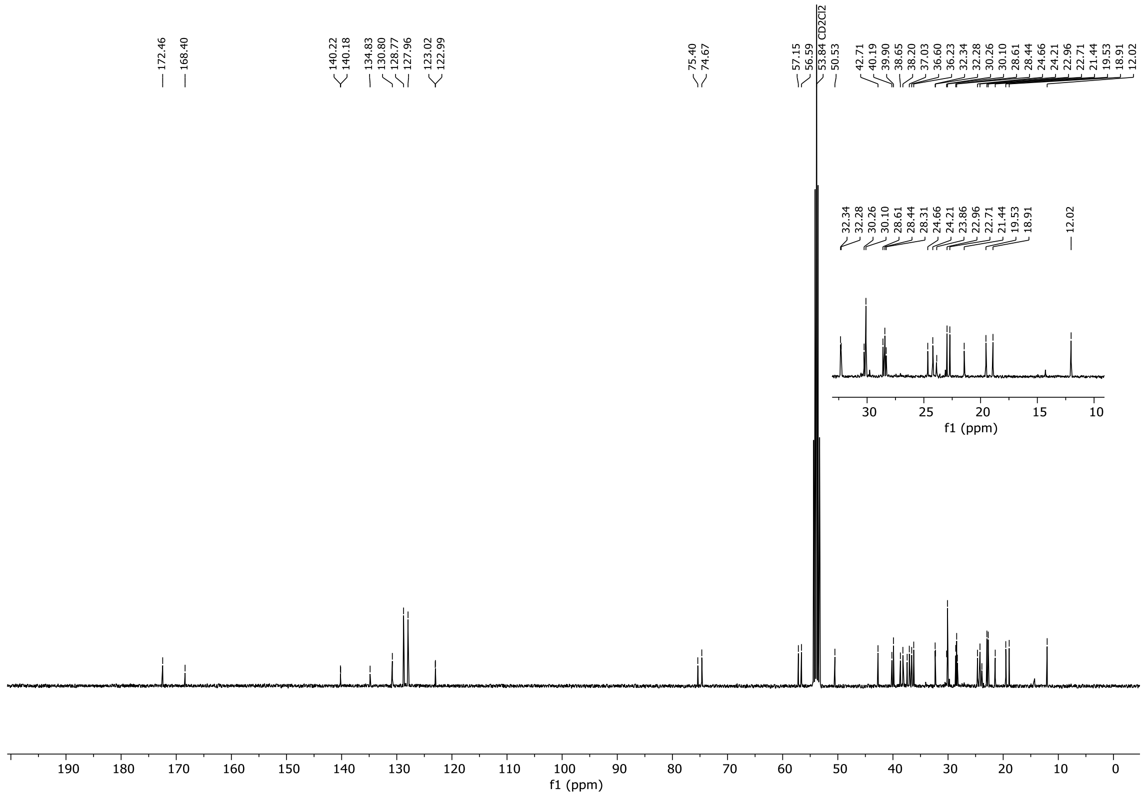

^{1}H NMR (400 MHz, $CDCl_3$) of **3af** (*see procedure*)

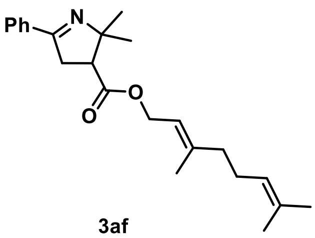

3af

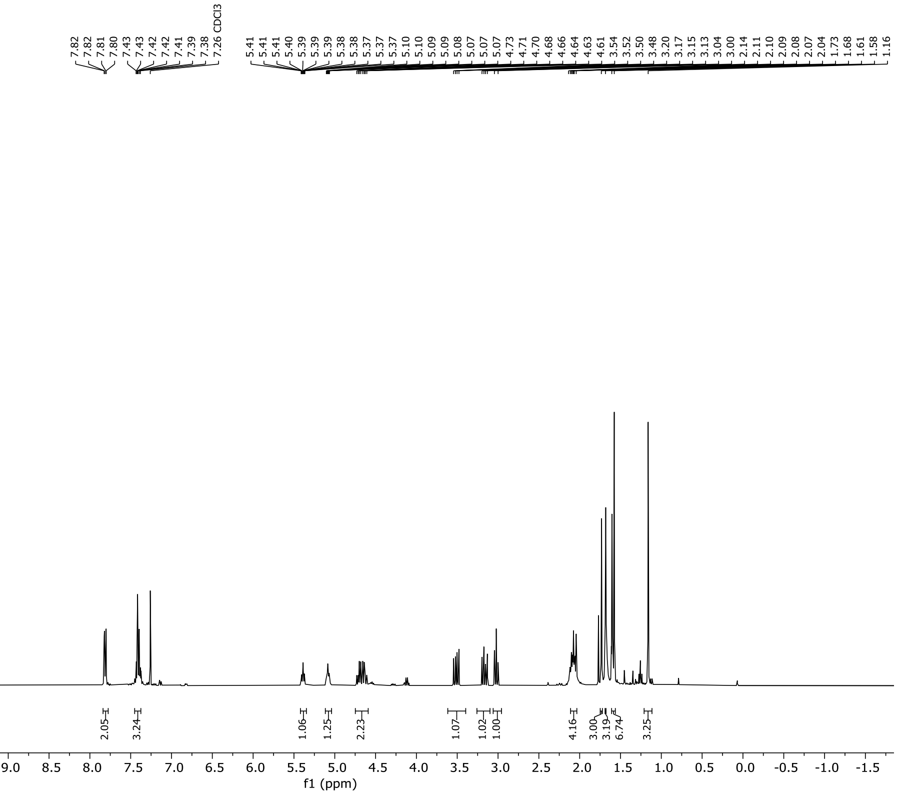

^{13}C NMR (126 MHz, $CDCl_3$) of **3af**

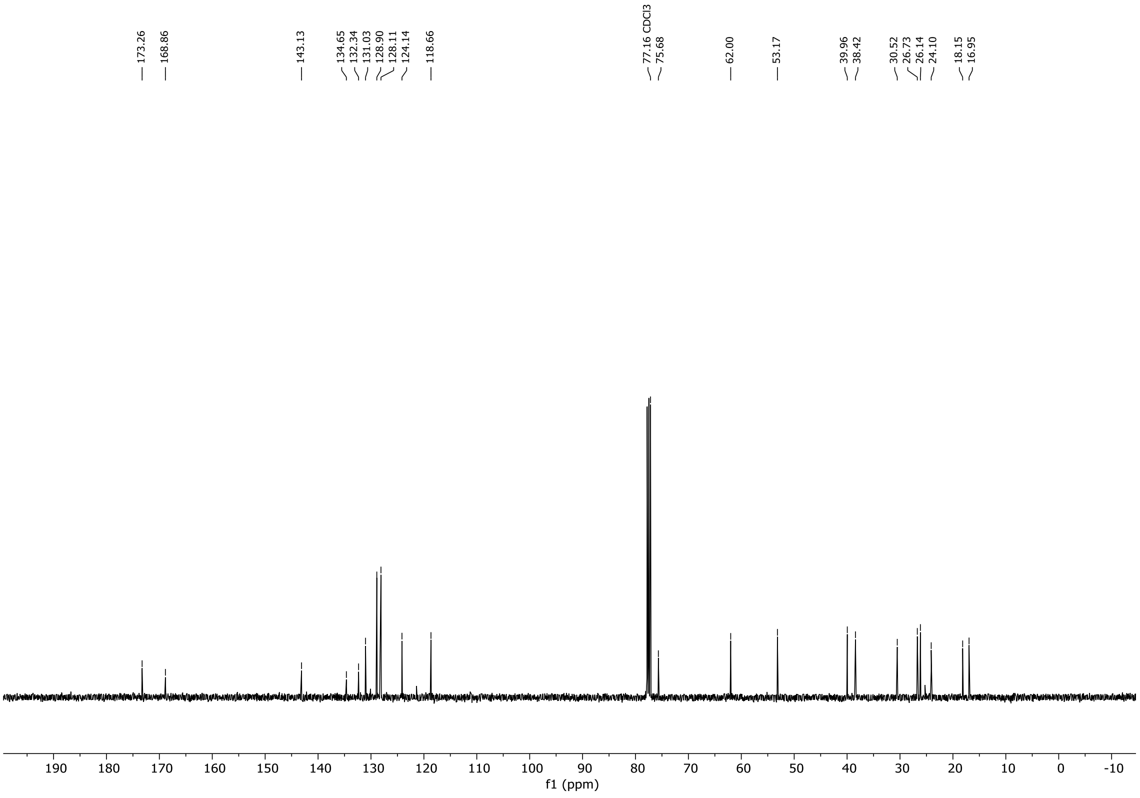

^{1}H NMR (599 MHz, $CDCl_3$) of **3ag** (*see procedure*)

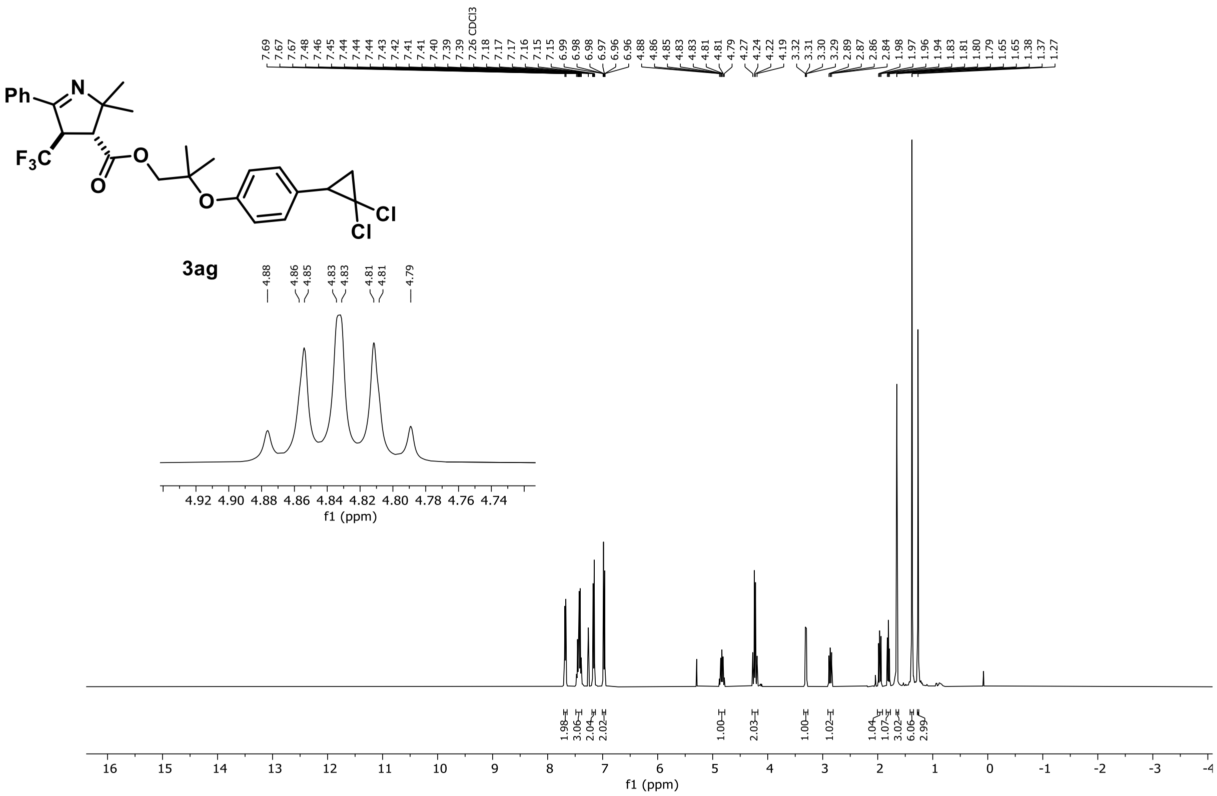

^{13}C NMR (126 MHz, $CDCl_3$) of **3ag**

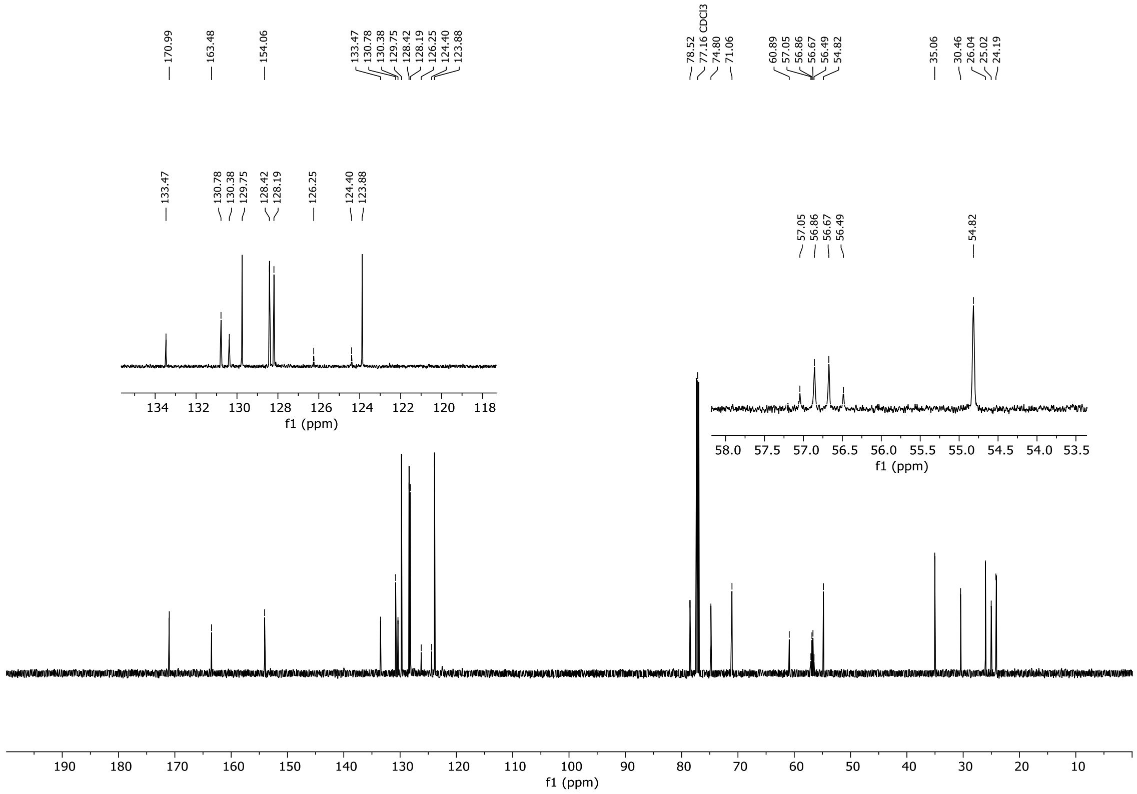

^{13}C NMR {^{1}H, ^{19}F} (126 MHz, $CDCl_3$) of **3ag**

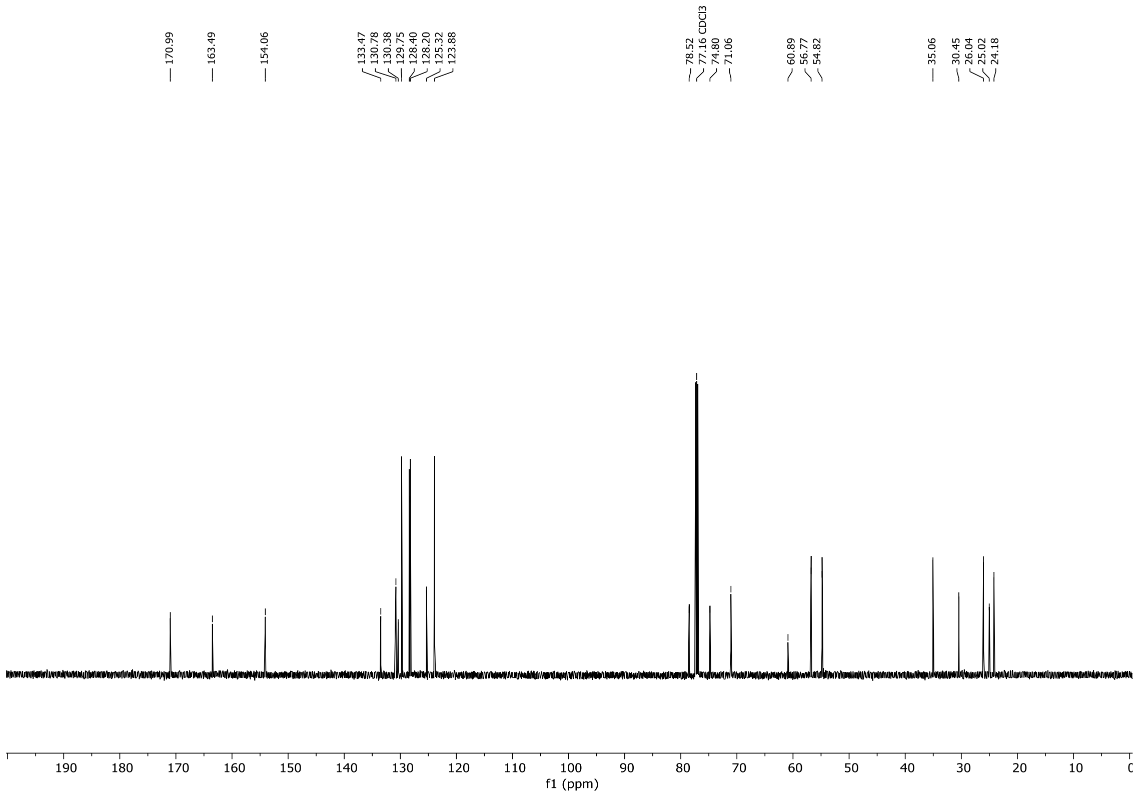

^{19}F NMR (377 MHz, $CDCl_3$) of **3ag**

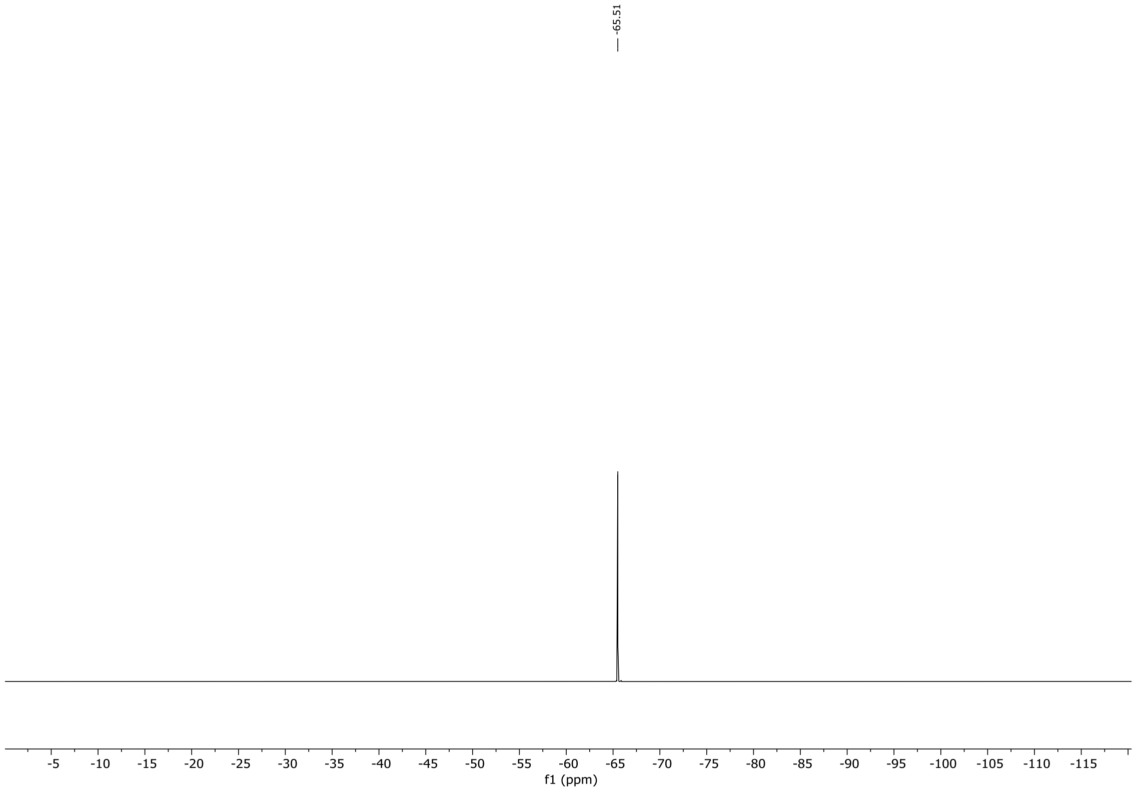

^{1}H NMR (400 MHz, $CDCl_3$) of **3ah** (*see procedure*)

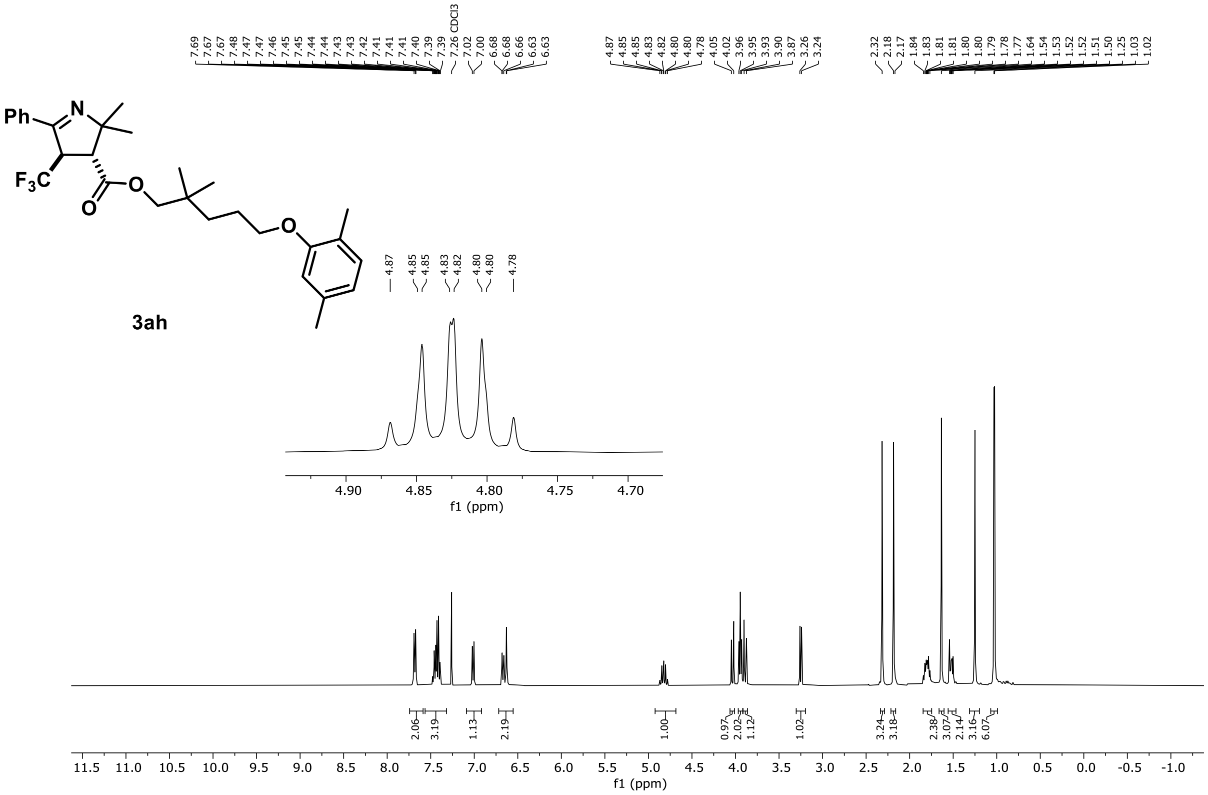

^{13}C NMR (126 MHz, $CDCl_3$) of **3ah**

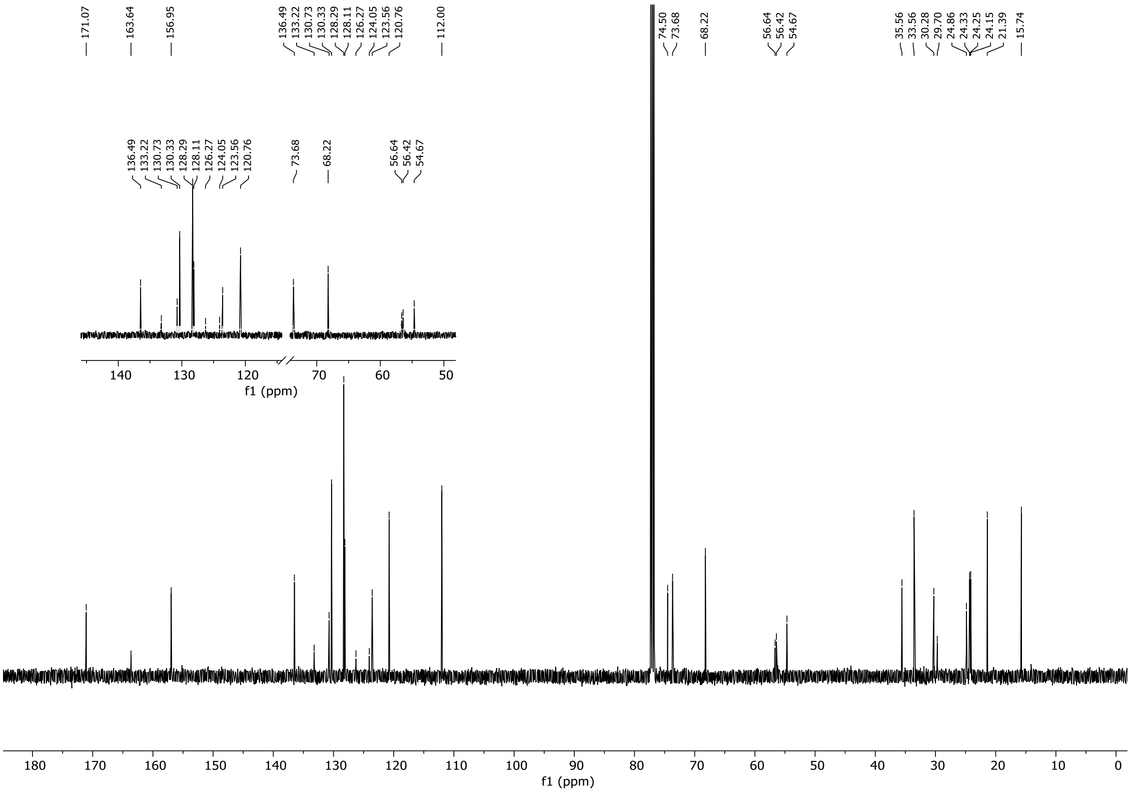

^{13}C NMR {^{1}H, ^{19}F} (126 MHz, $CDCl_3$) of **3ah**

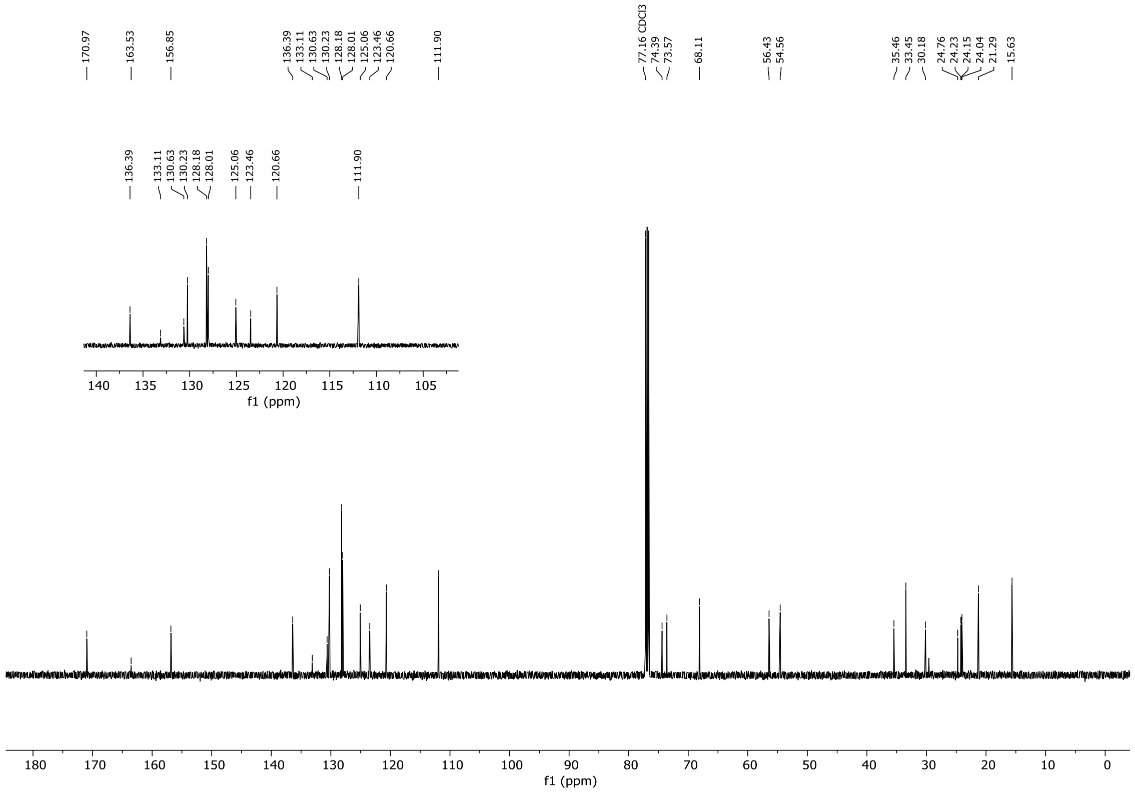

^{19}F NMR (376 MHz, $CDCl_3$) of **3ah**

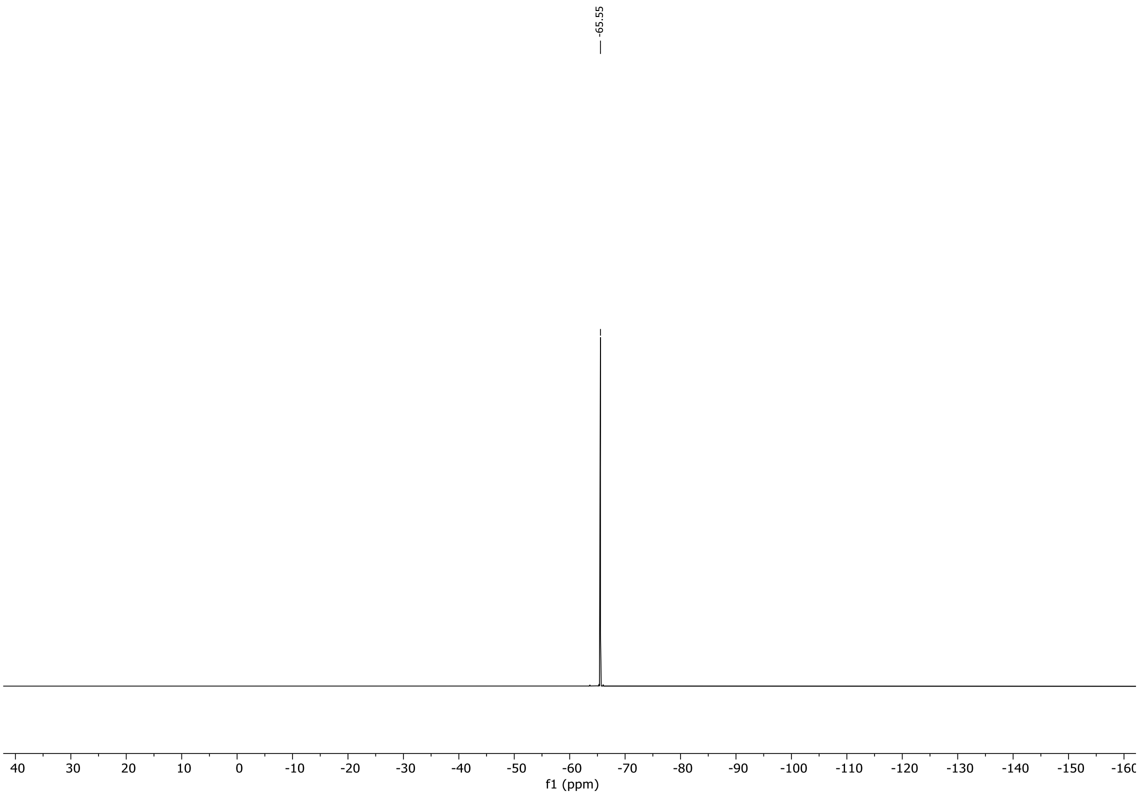

^{1}H NMR (400 MHz, $CDCl_3$) of **6a** (see procedure)

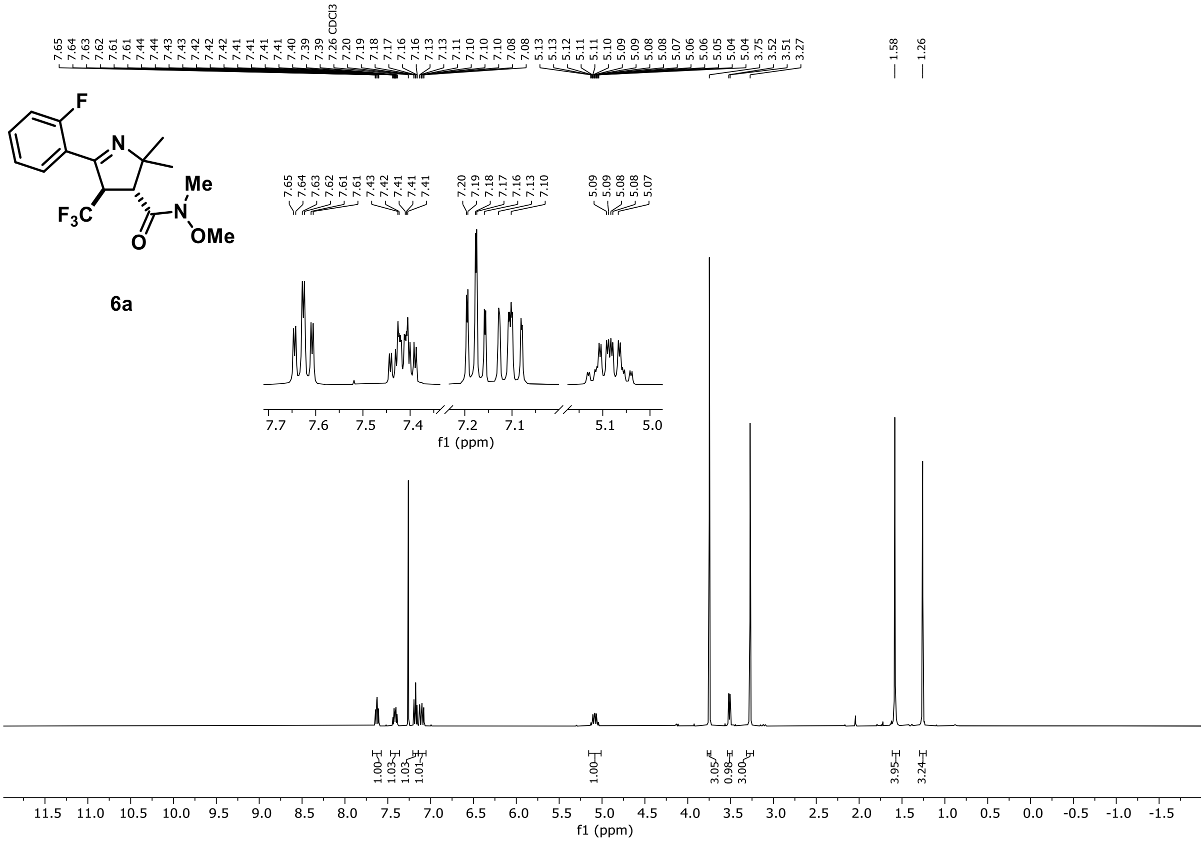

^{19}F NMR (470 MHz, $CDCl_3$) of **6a**

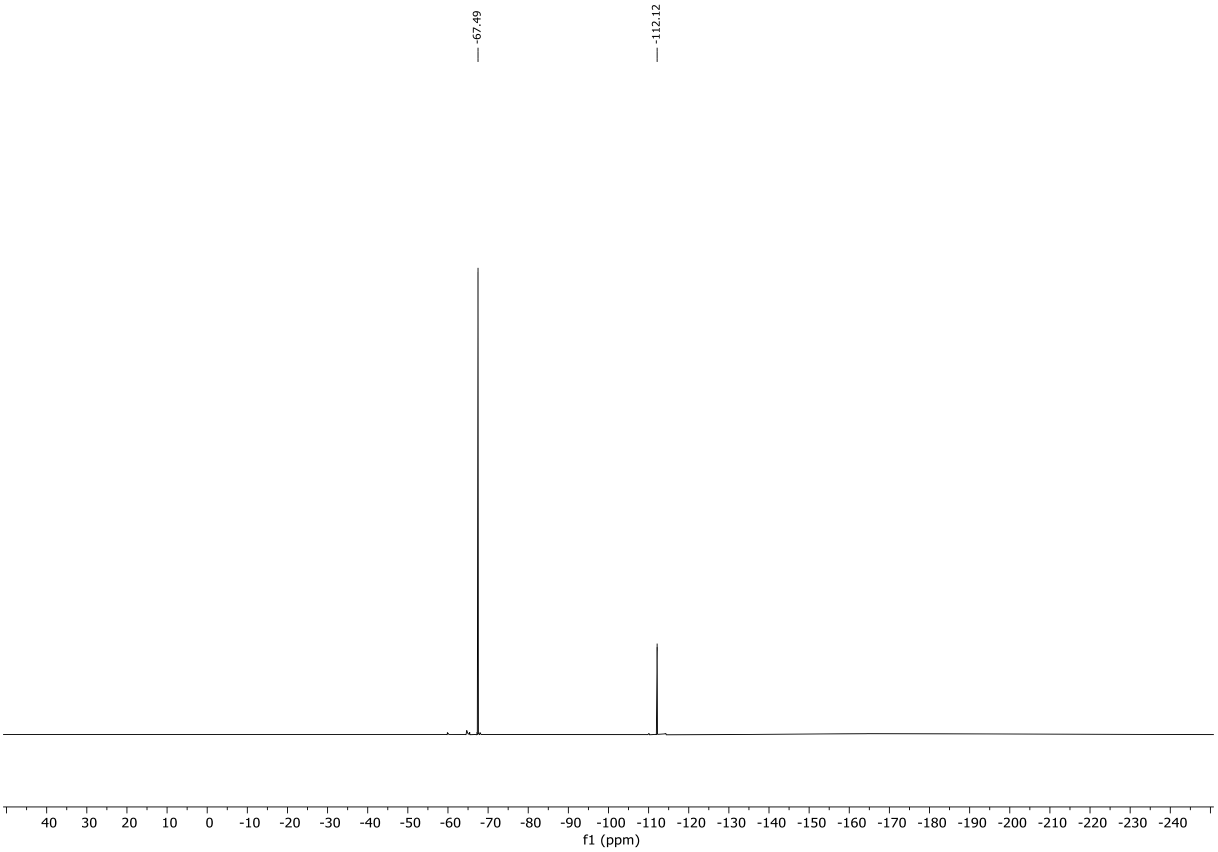

^{13}C NMR (151 MHz, $CDCl_3$) of **6a**

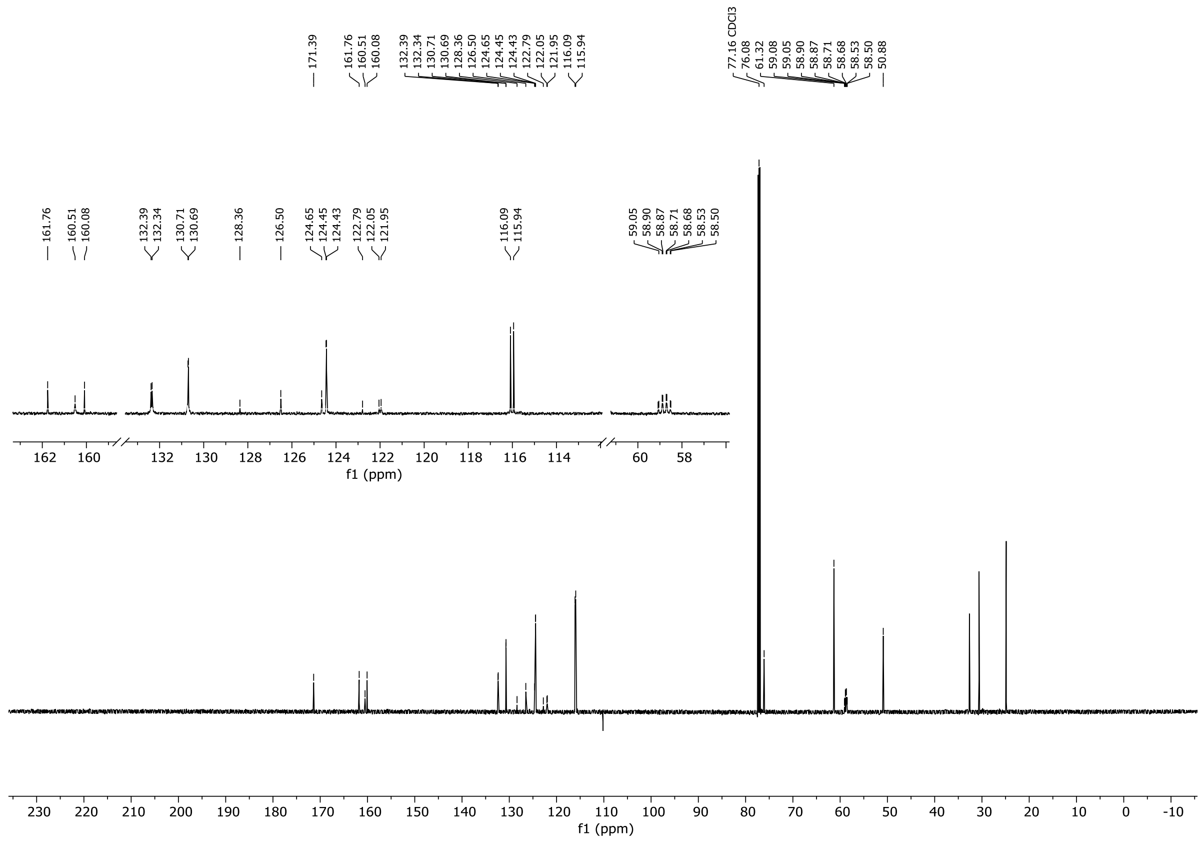

^{13}C NMR {^{1}H, ^{19}F} (126 MHz, $CDCl_3$) of **6a**

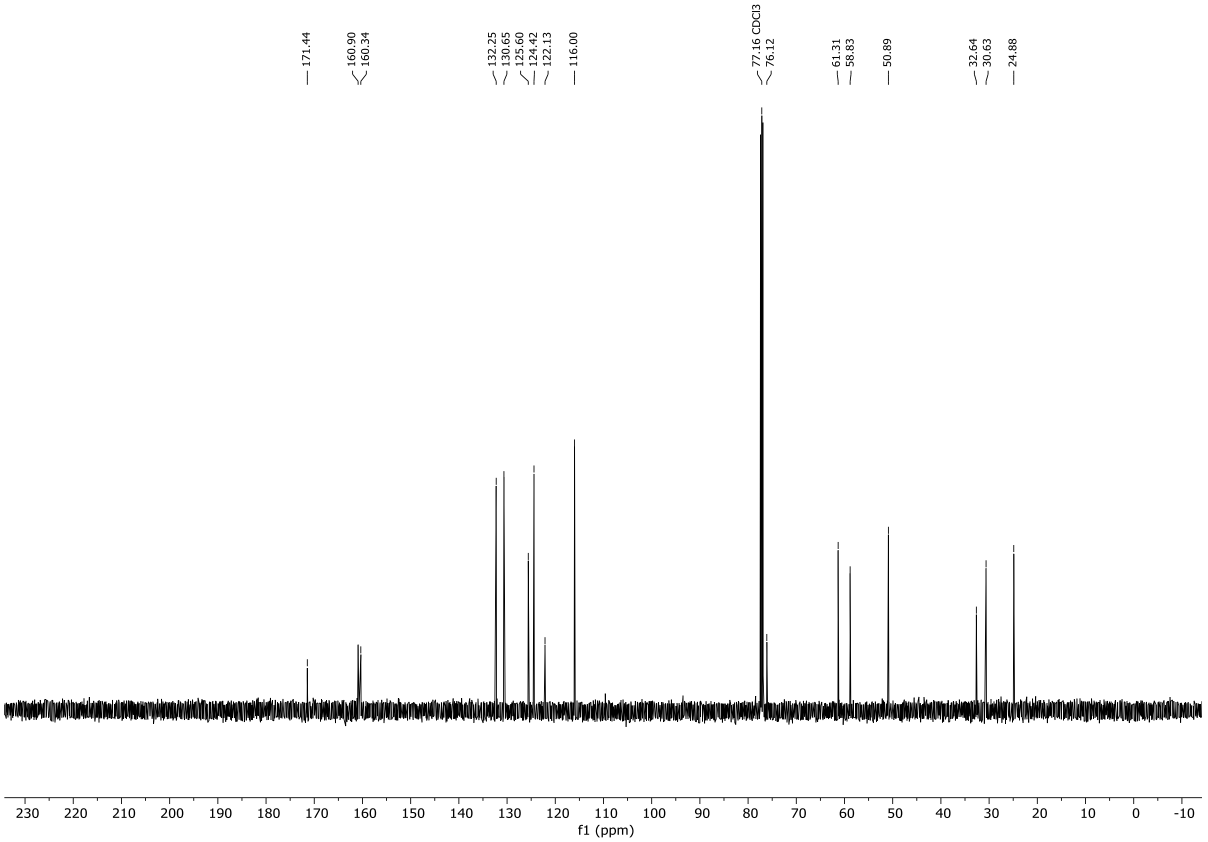

^{1}H NMR (599 MHz, $CDCl_3$) of **6b** (see procedure)

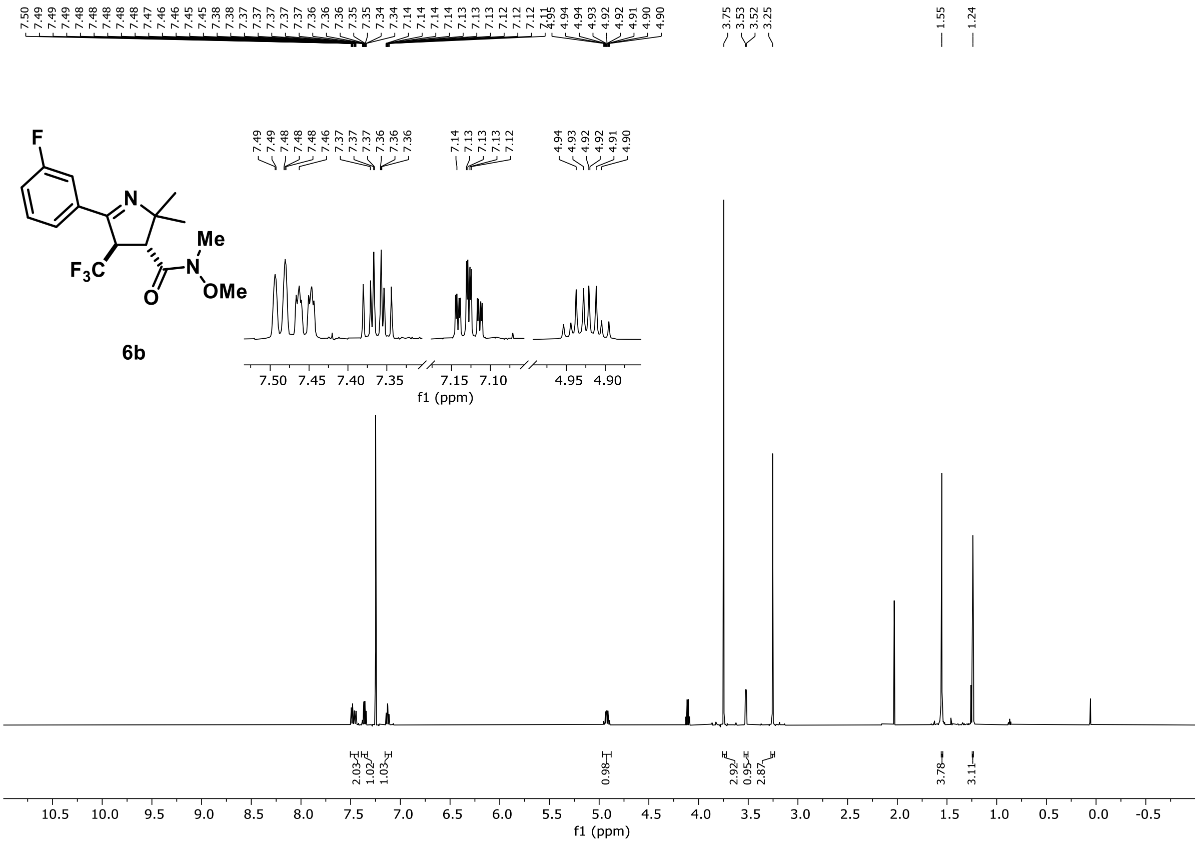

^{19}F NMR (564 MHz, $CDCl_3$) of **6b**

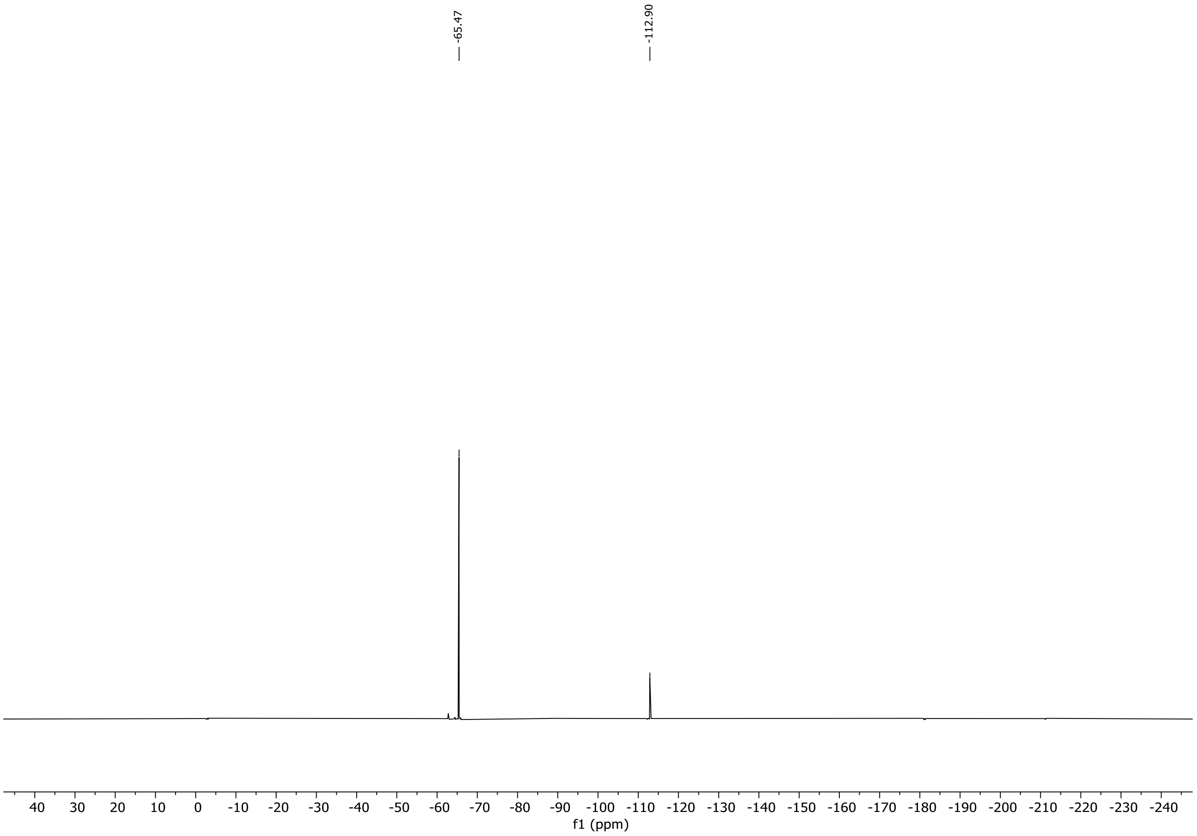

^{13}C NMR (151 MHz, $CDCl_3$) of **6b**

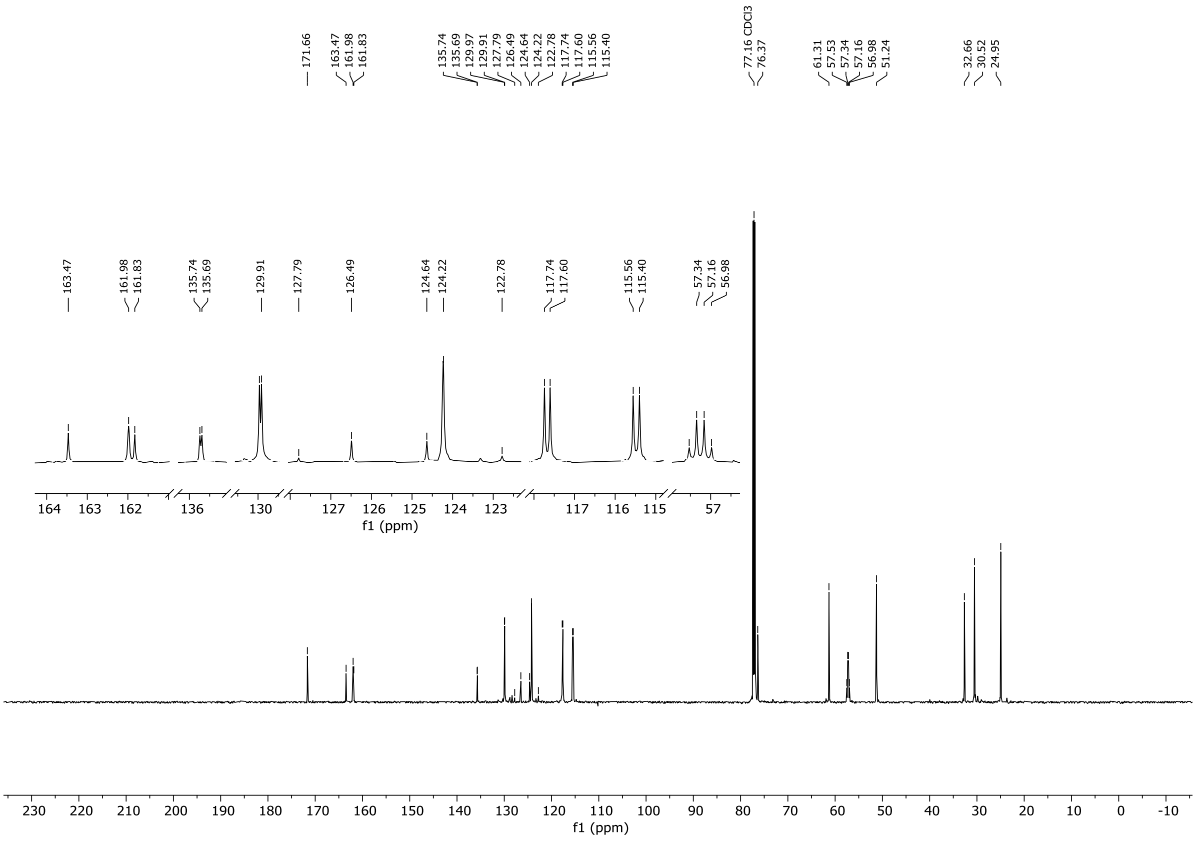

^{13}C NMR {^{1}H, ^{19}F} (126 MHz, $CDCl_3$) of **6b**

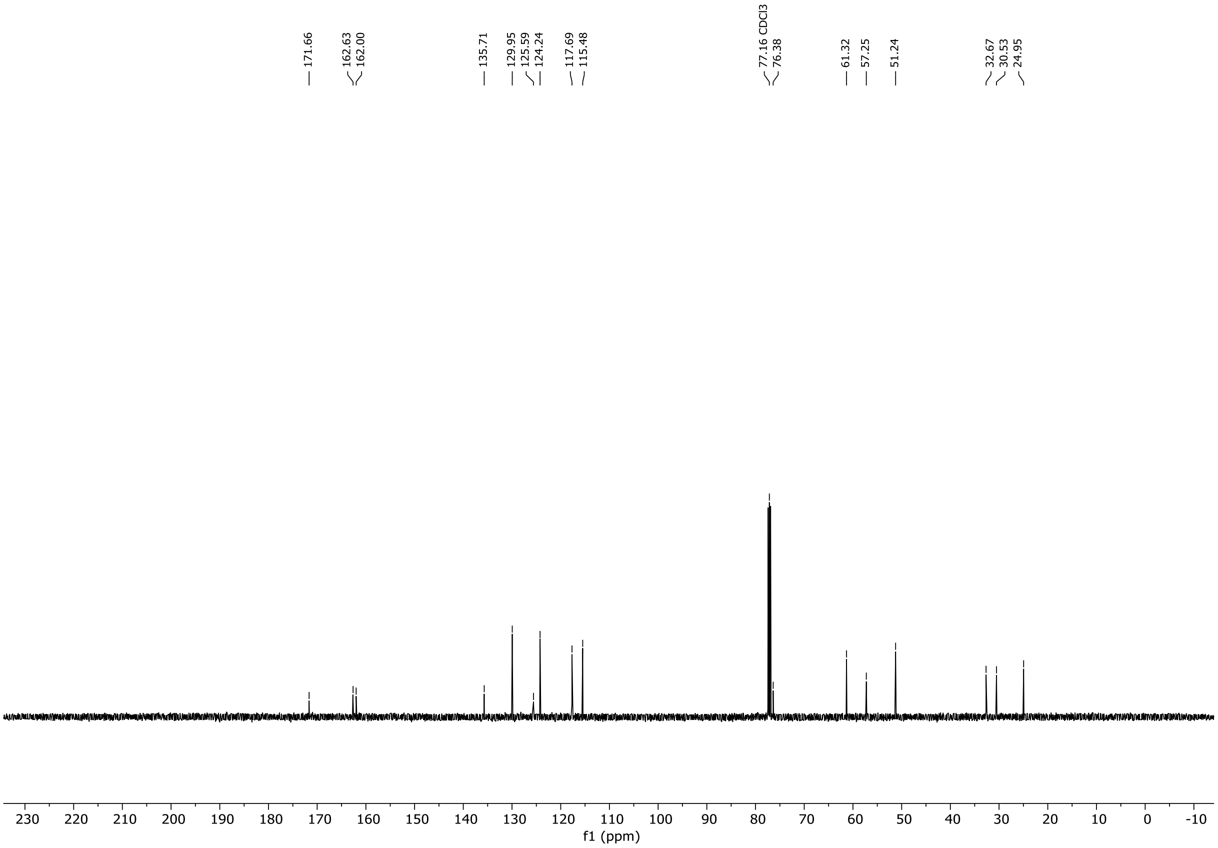

^{1}H NMR (599 MHz, $CDCl_3$) of **6c** (*see procedure*)

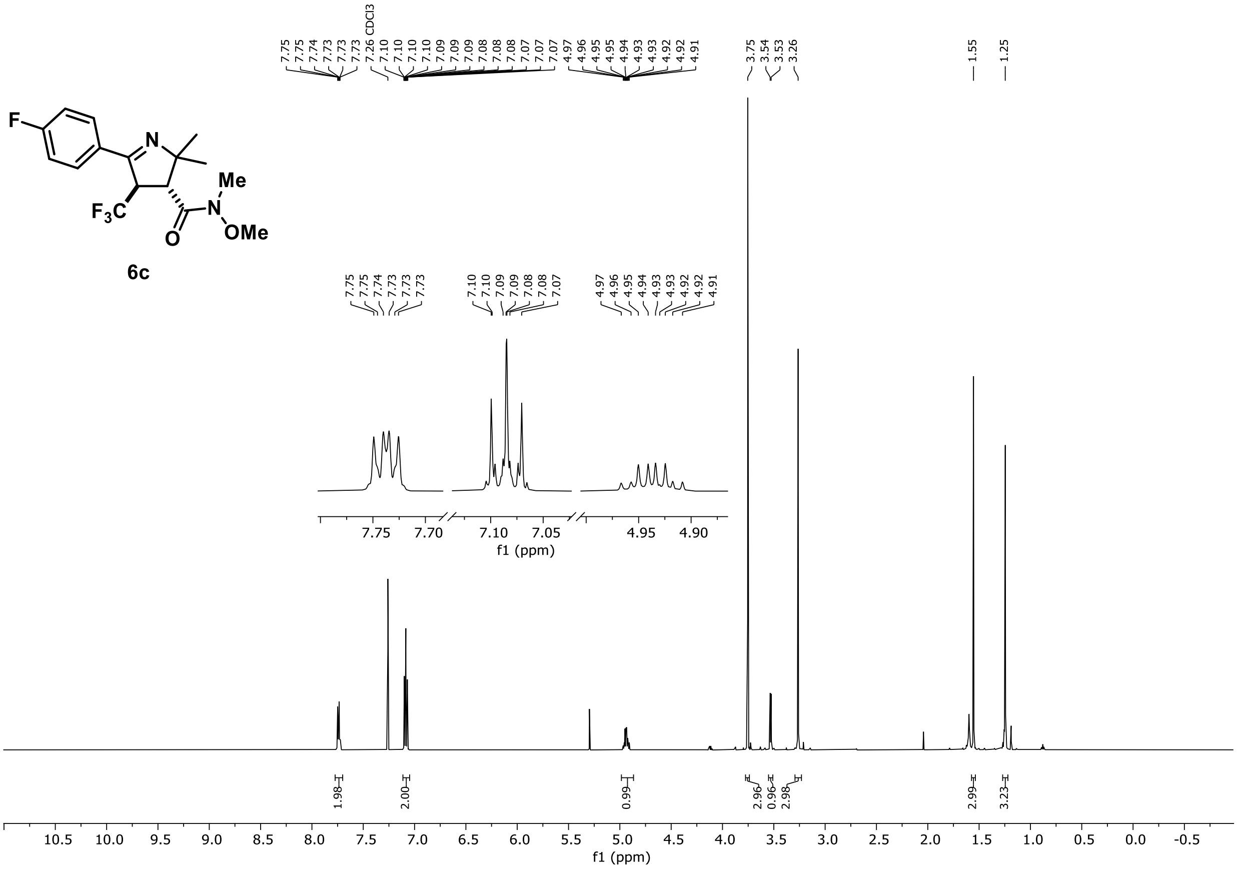

^{19}F NMR (564 MHz, $CDCl_3$) of **6c**

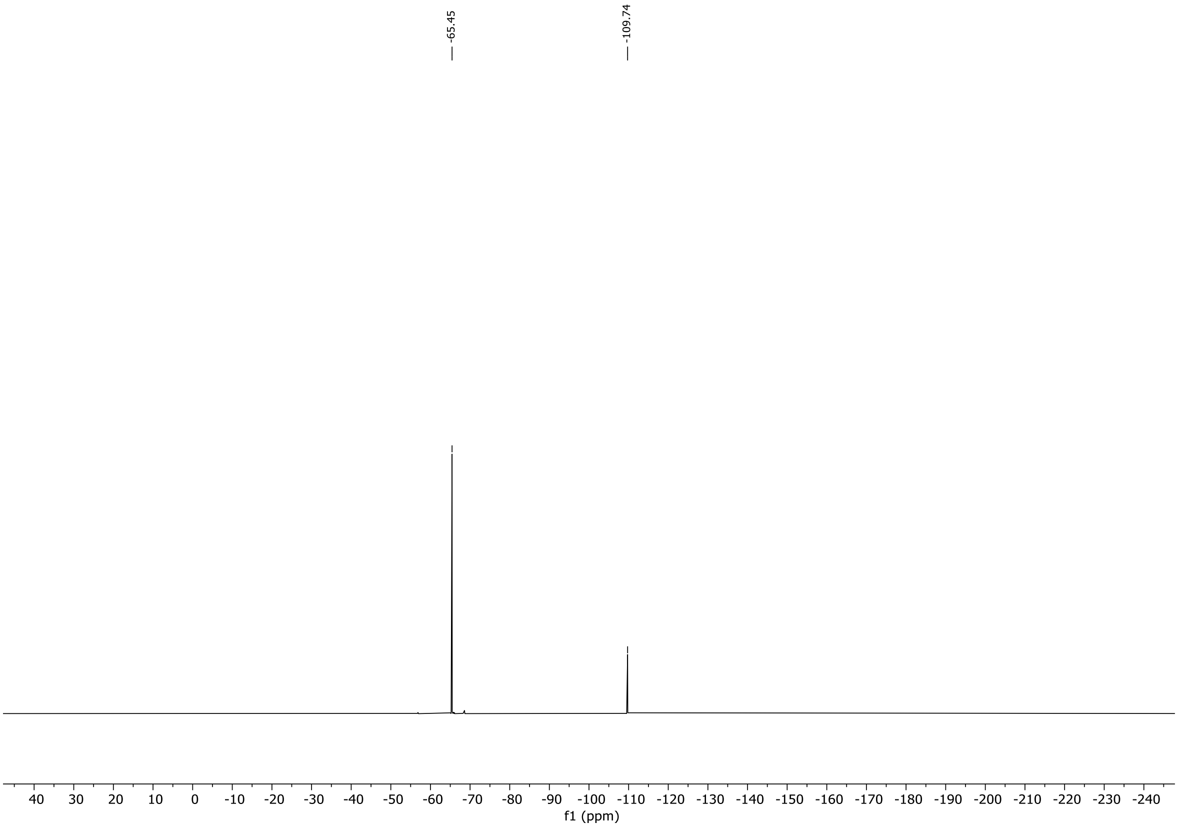

^{13}C NMR (151 MHz, $CDCl_3$) of **6c**

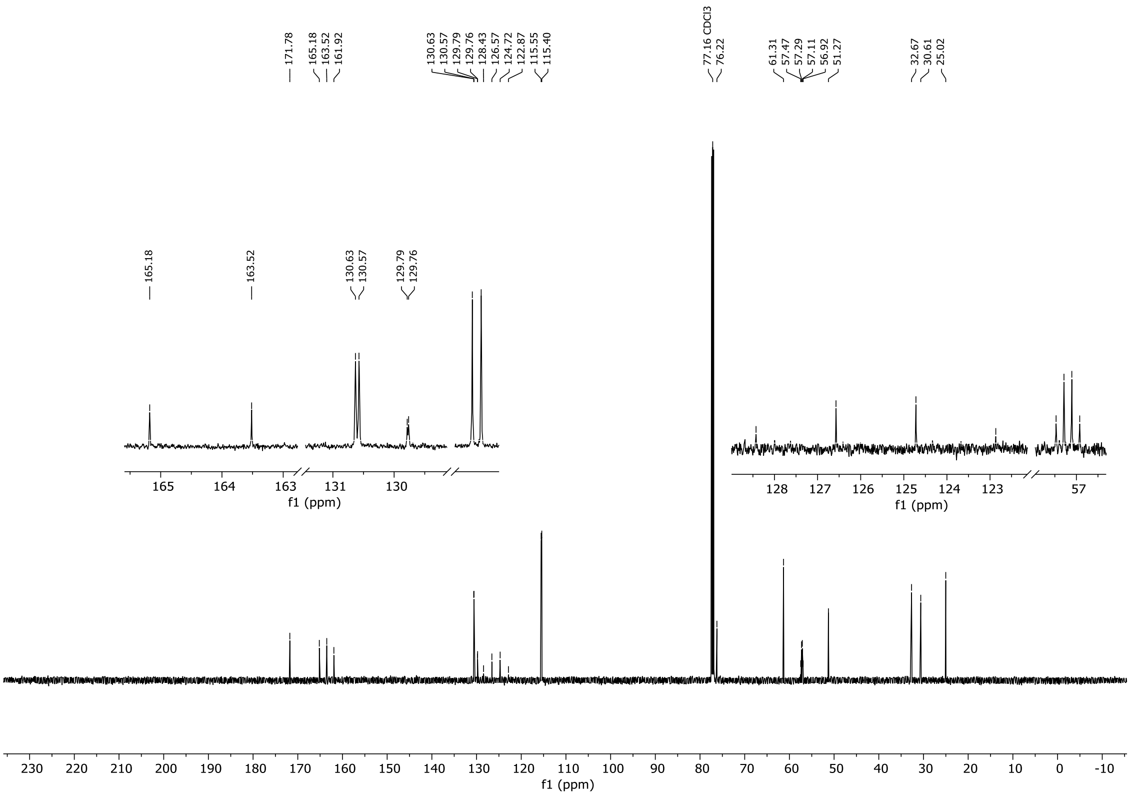

^{13}C NMR {^{1}H, ^{19}F} (151 MHz, $CDCl_3$) of **6c**

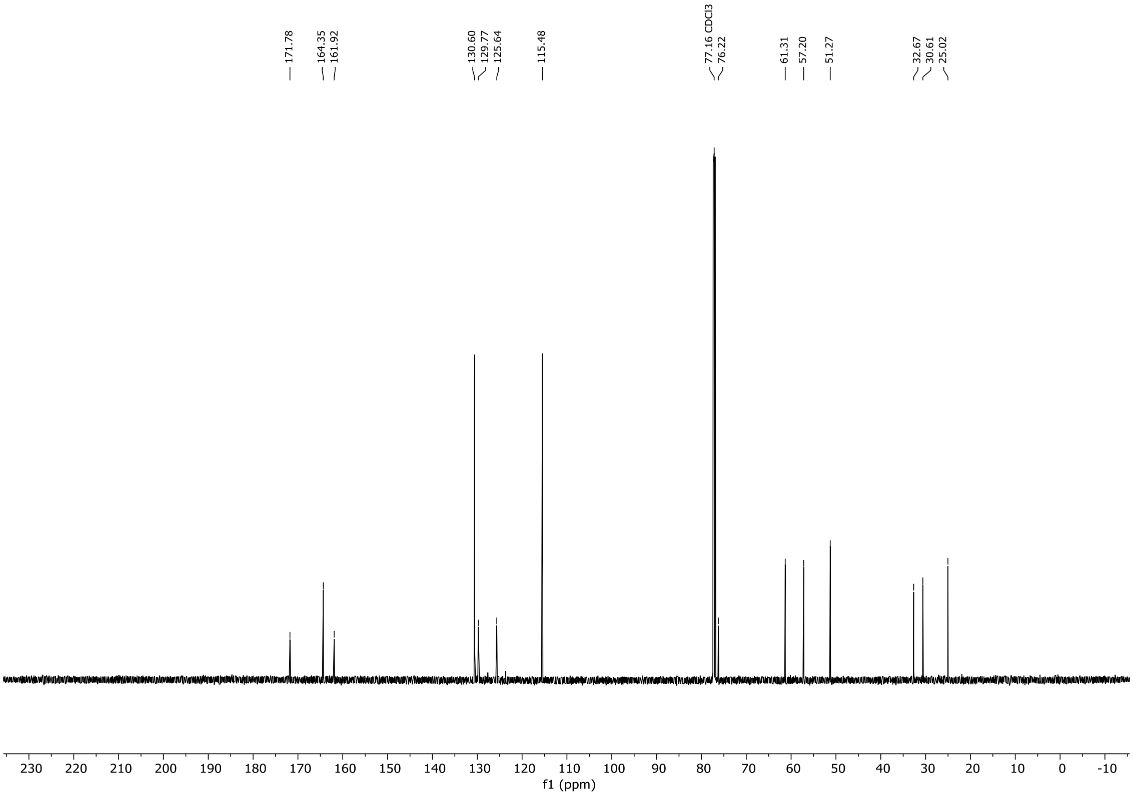

^{1}H NMR (500 MHz, $CDCl_3$) of **6d** (<u>*see procedure*</u>)

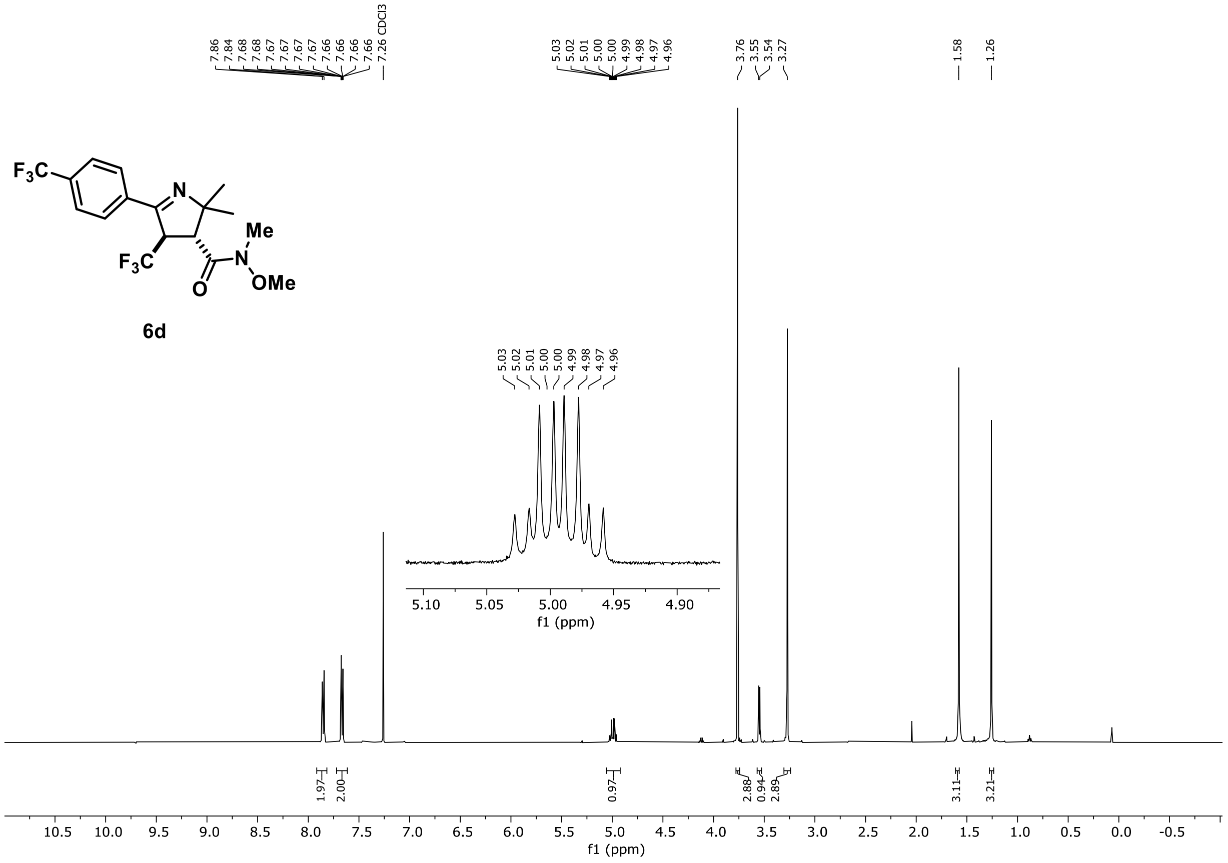

^{19}F NMR (470 MHz, $CDCl_3$) of **6d**

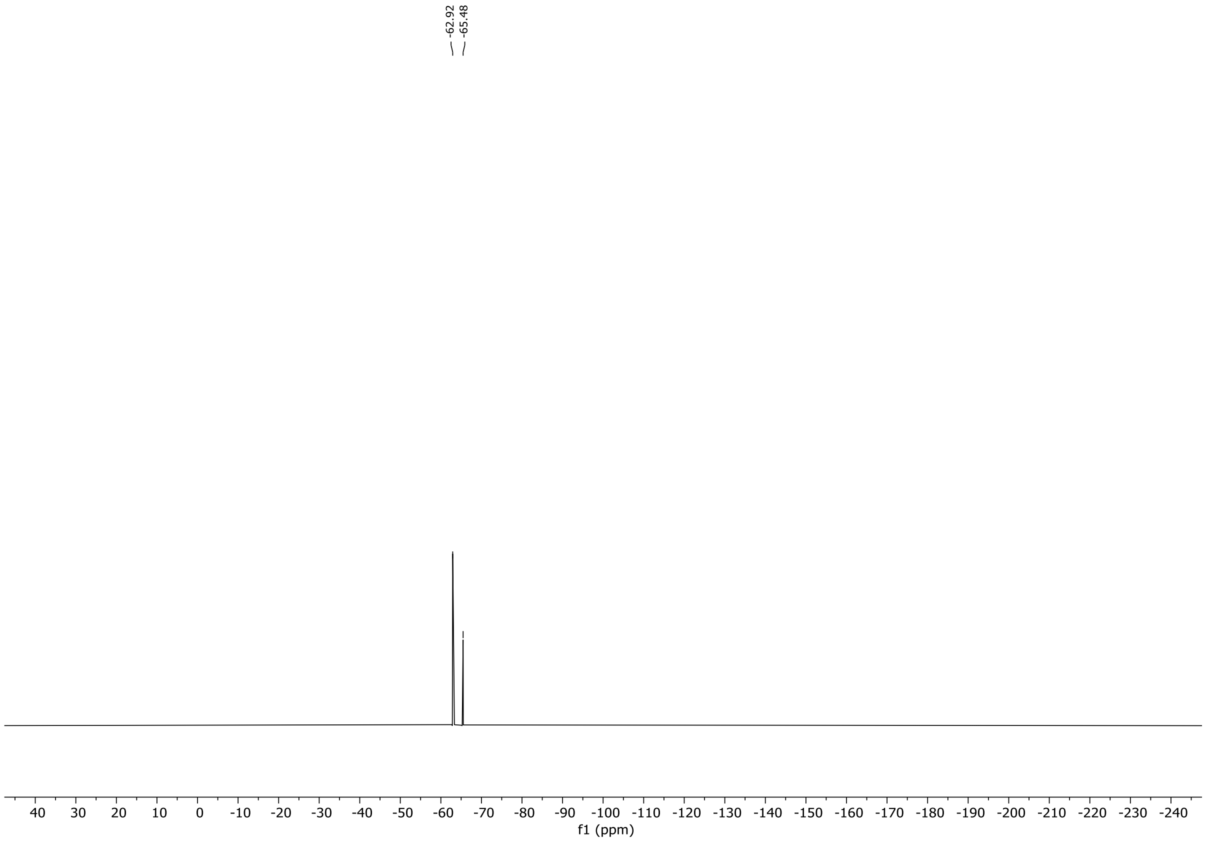

^{13}C NMR (151 MHz, $CDCl_3$) of **6d**

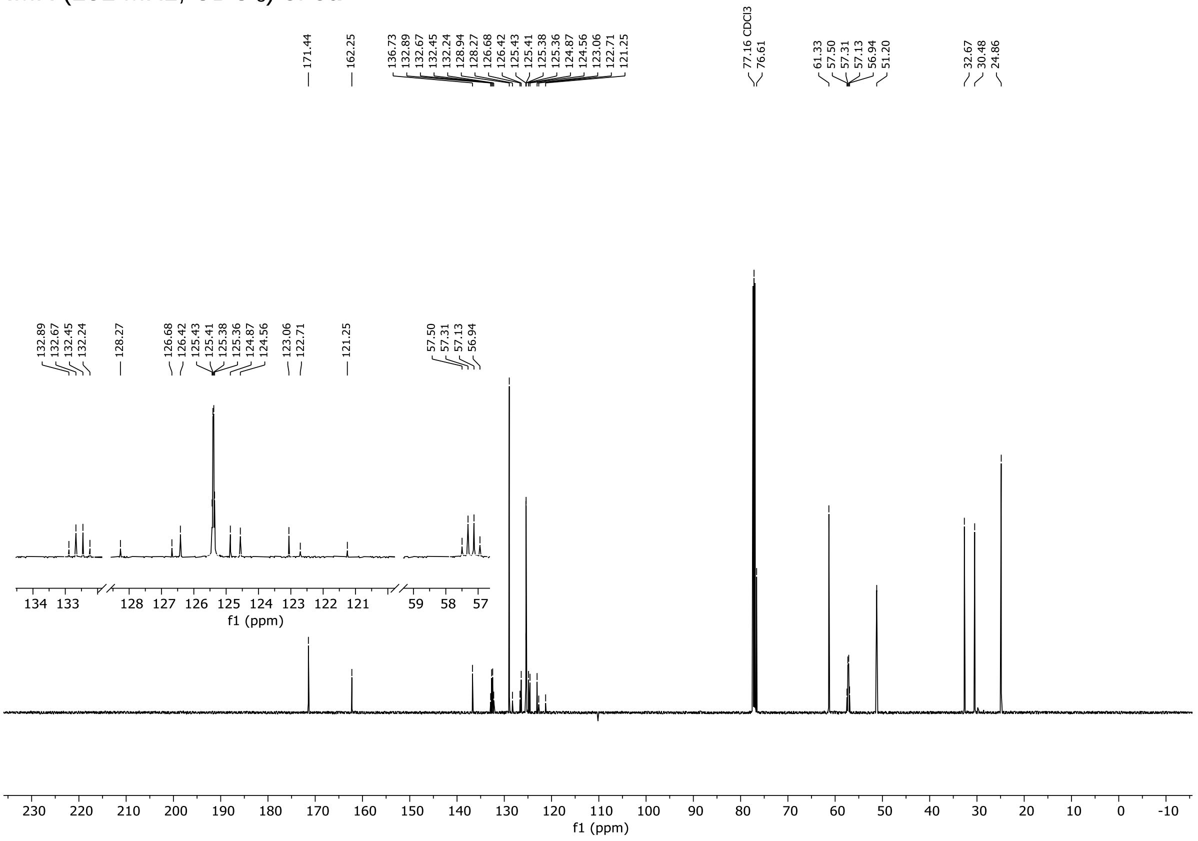

^{13}C NMR {^{1}H, ^{19}F} (126 MHz, $CDCl_3$) of **6d**

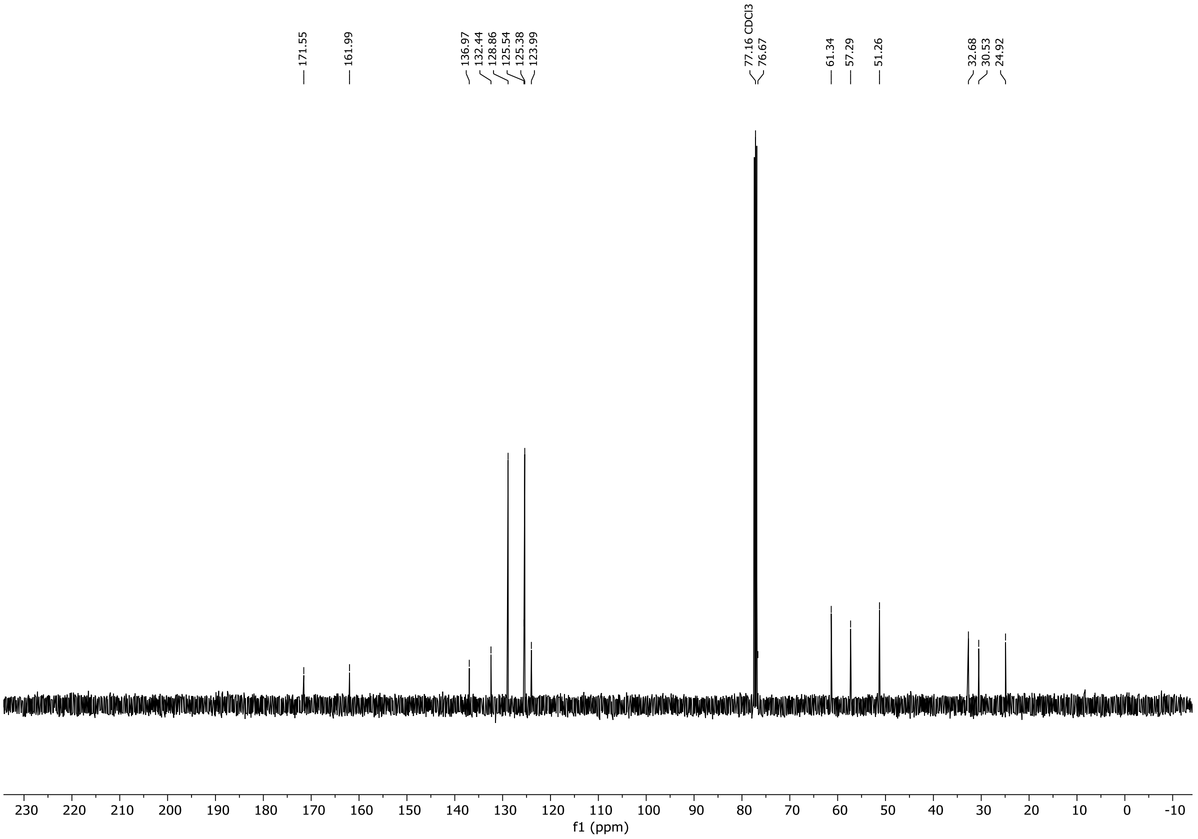

^{1}H NMR (500 MHz, $CDCl_3$) of **6f** (*see procedure*)

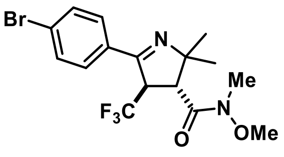

6f

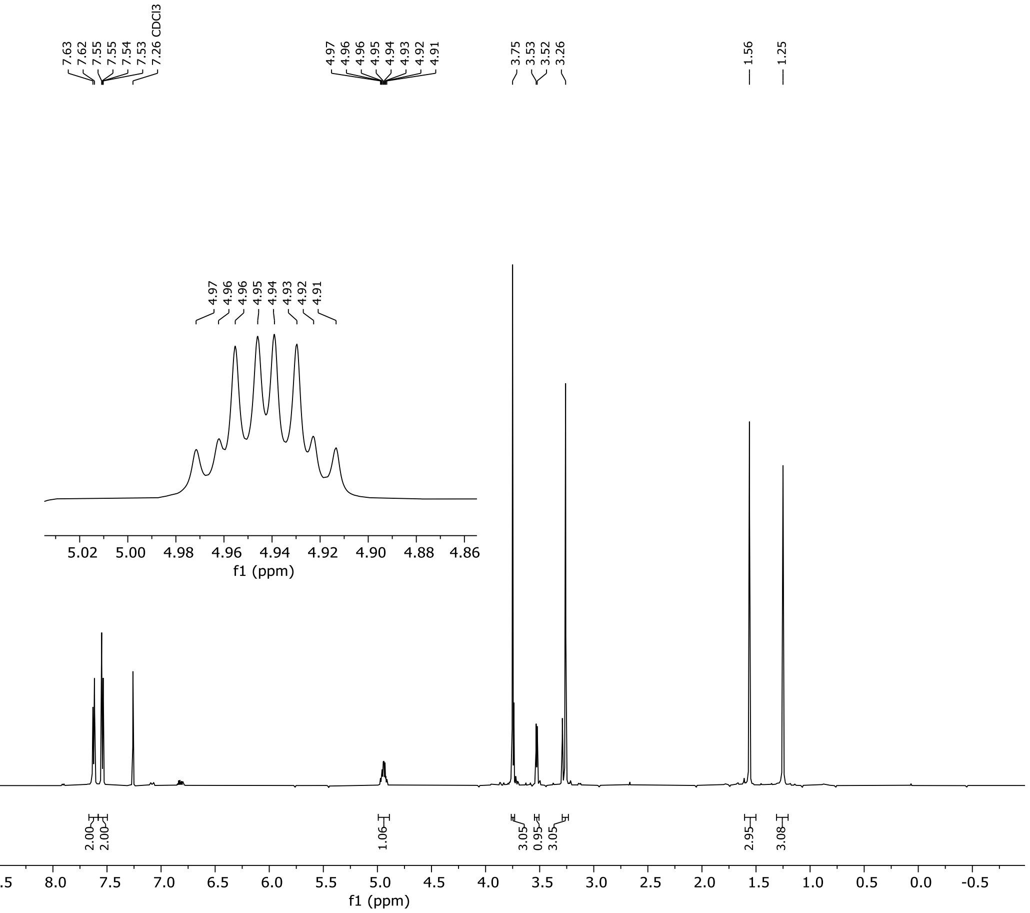

^{19}F NMR (470 MHz, $CDCl_3$) of **6f**

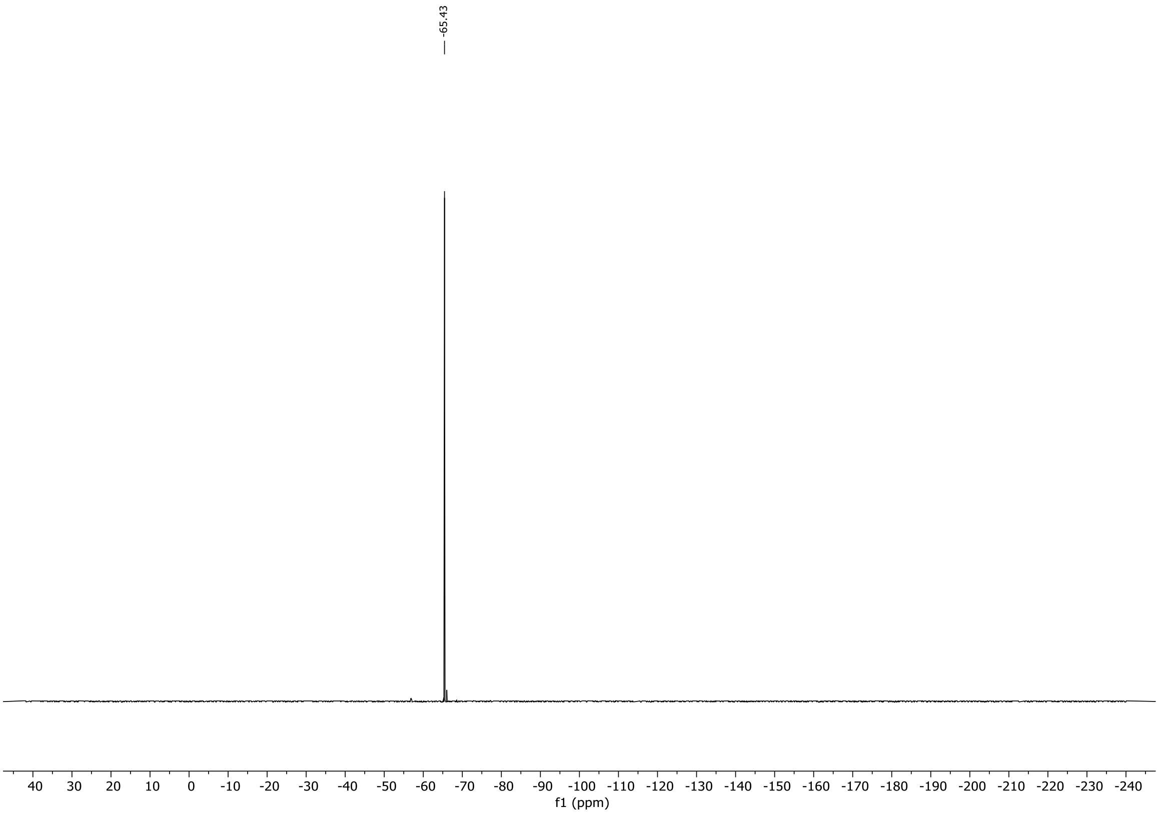

^{13}C NMR (151 MHz, $CDCl_3$) of **6f**

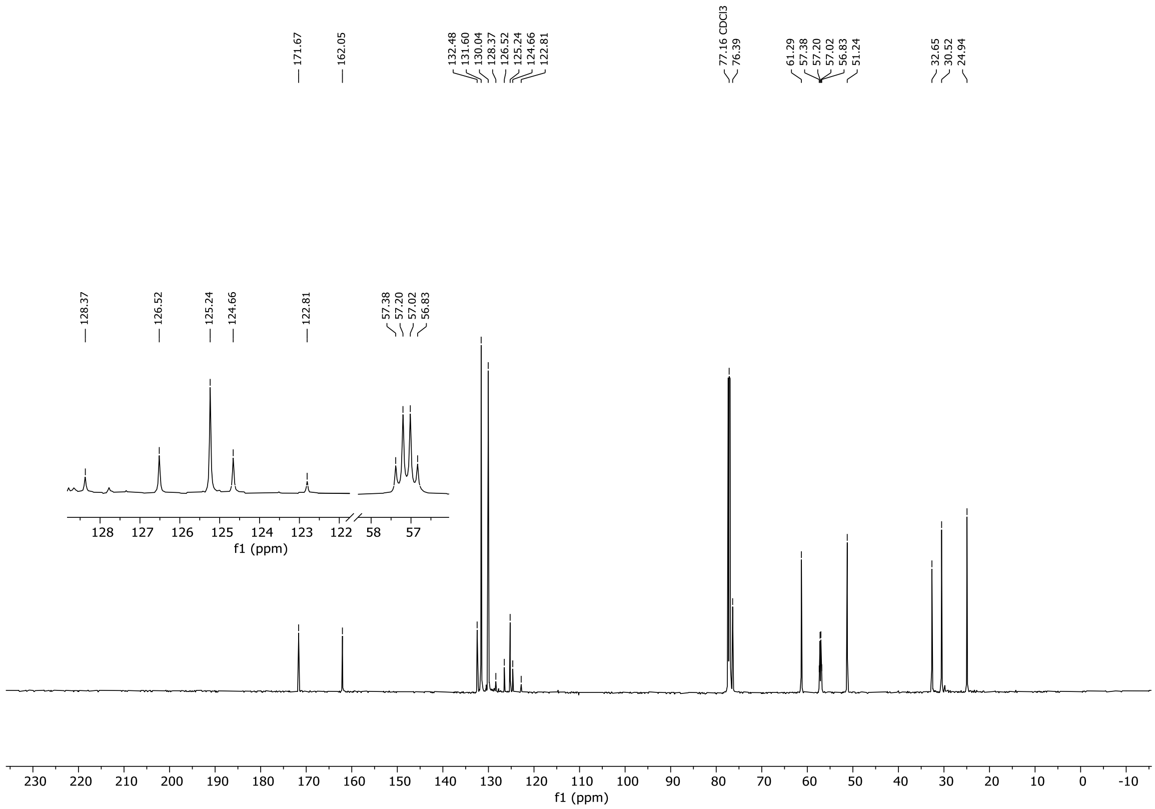

^{13}C NMR {^{1}H, ^{19}F} (126 MHz, $CDCl_3$) of **6f**

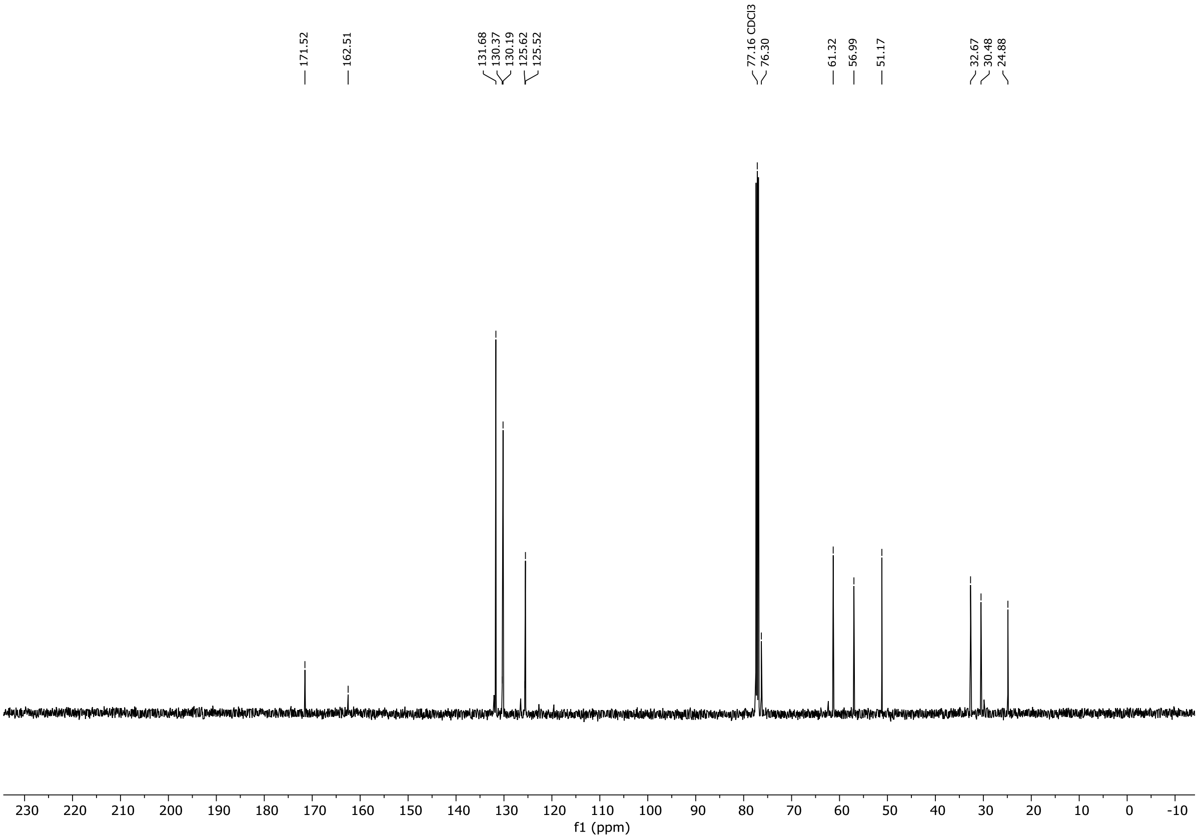

^{1}H NMR (500 MHz, $CDCl_3$) of **6e** (*see procedure*)

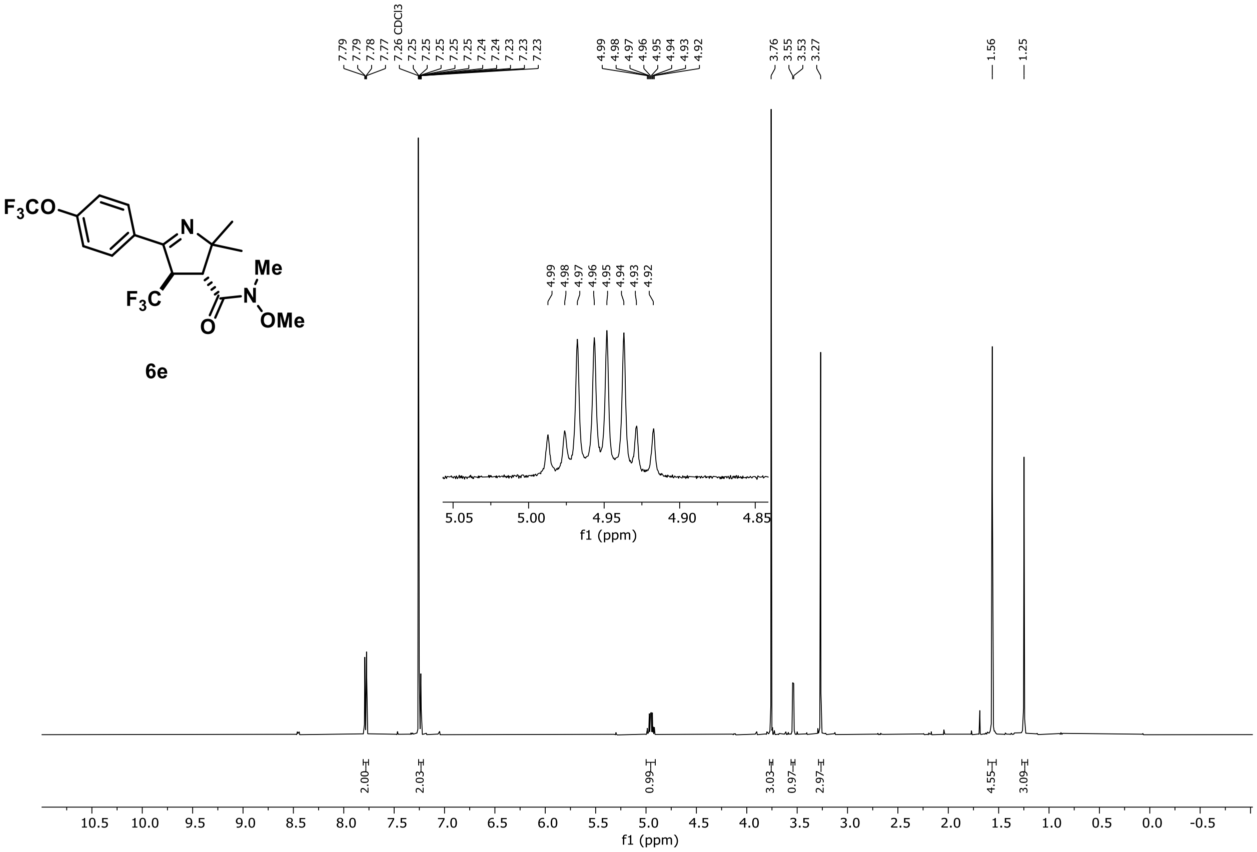

^{19}F NMR (470 MHz, $CDCl_3$) of **6e**

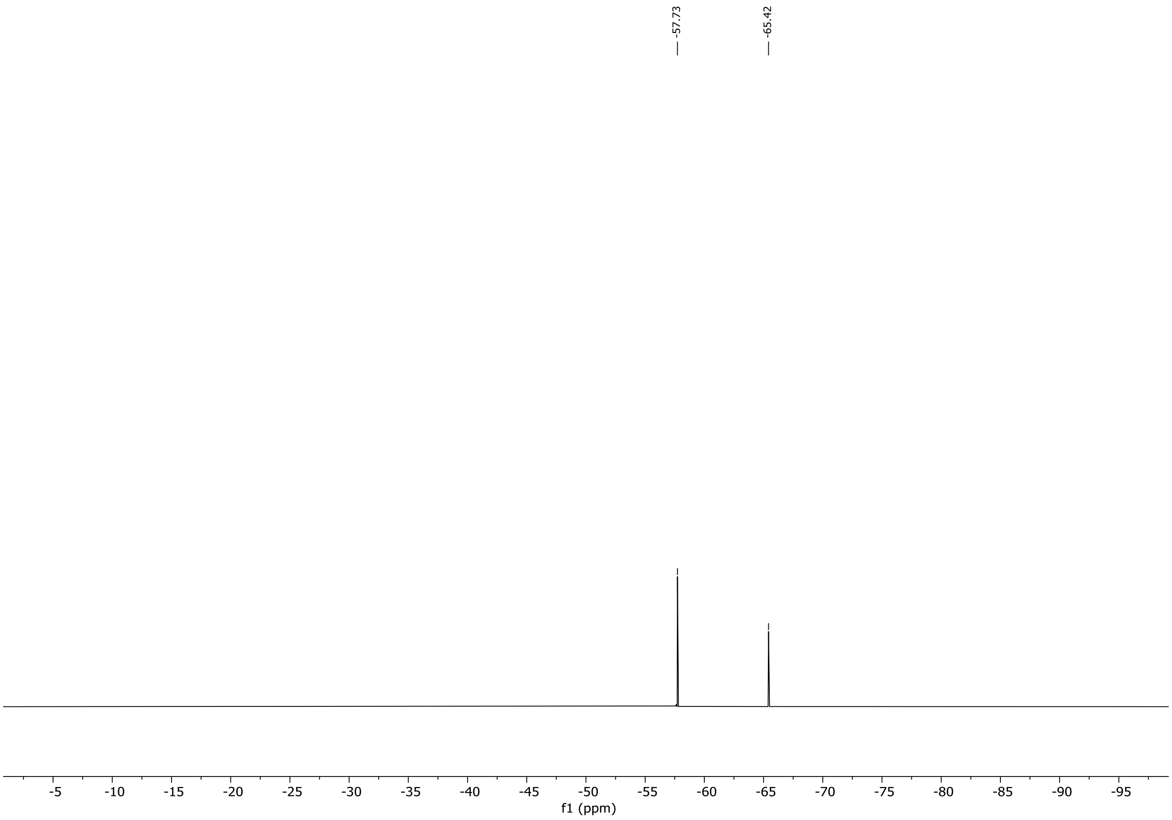

^{13}C NMR (151 MHz, $CDCl_3$) of **6e**

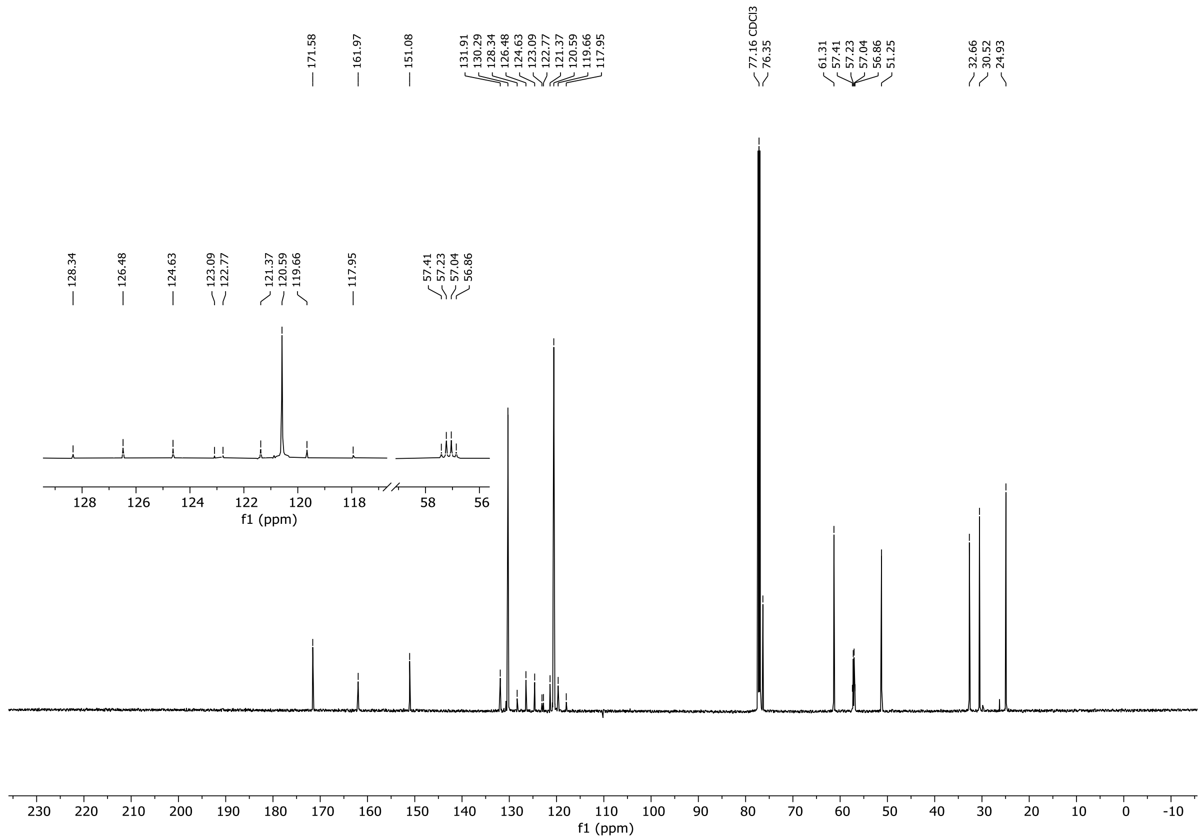

^{13}C NMR {^{1}H, ^{19}F} (126 MHz, $CDCl_3$) of **6e**

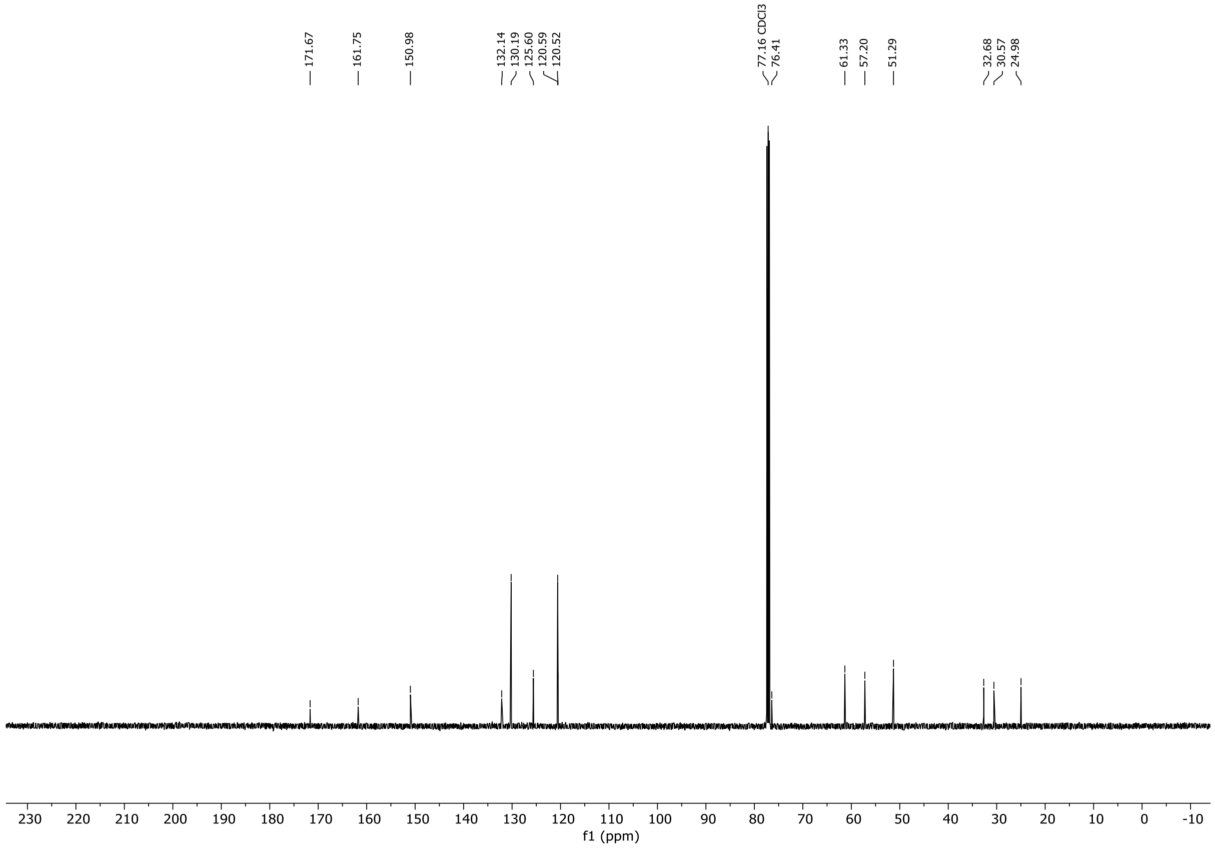

^{1}H NMR (500 MHz, $CDCl_3$) of **6g** (*see procedure*)

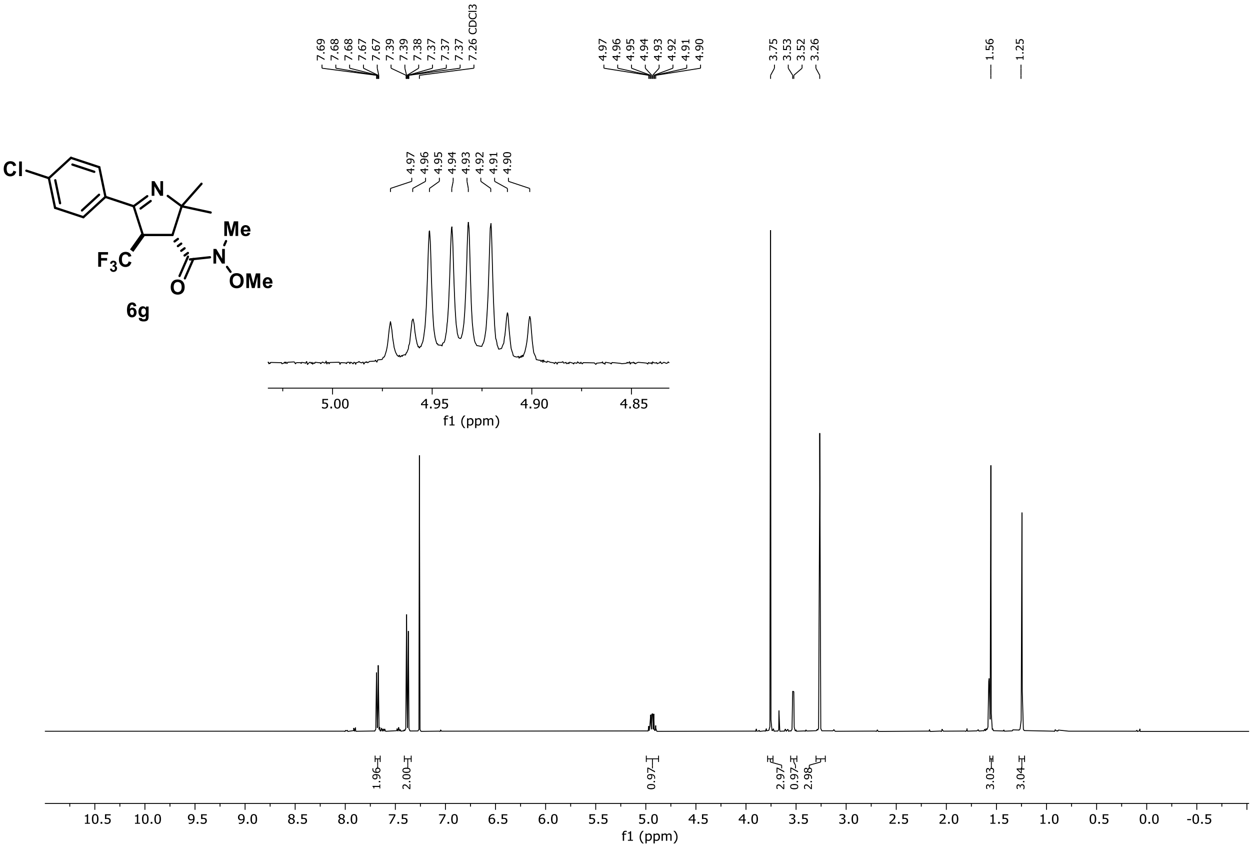

^{19}F NMR (470 MHz, $CDCl_3$) of **6g**

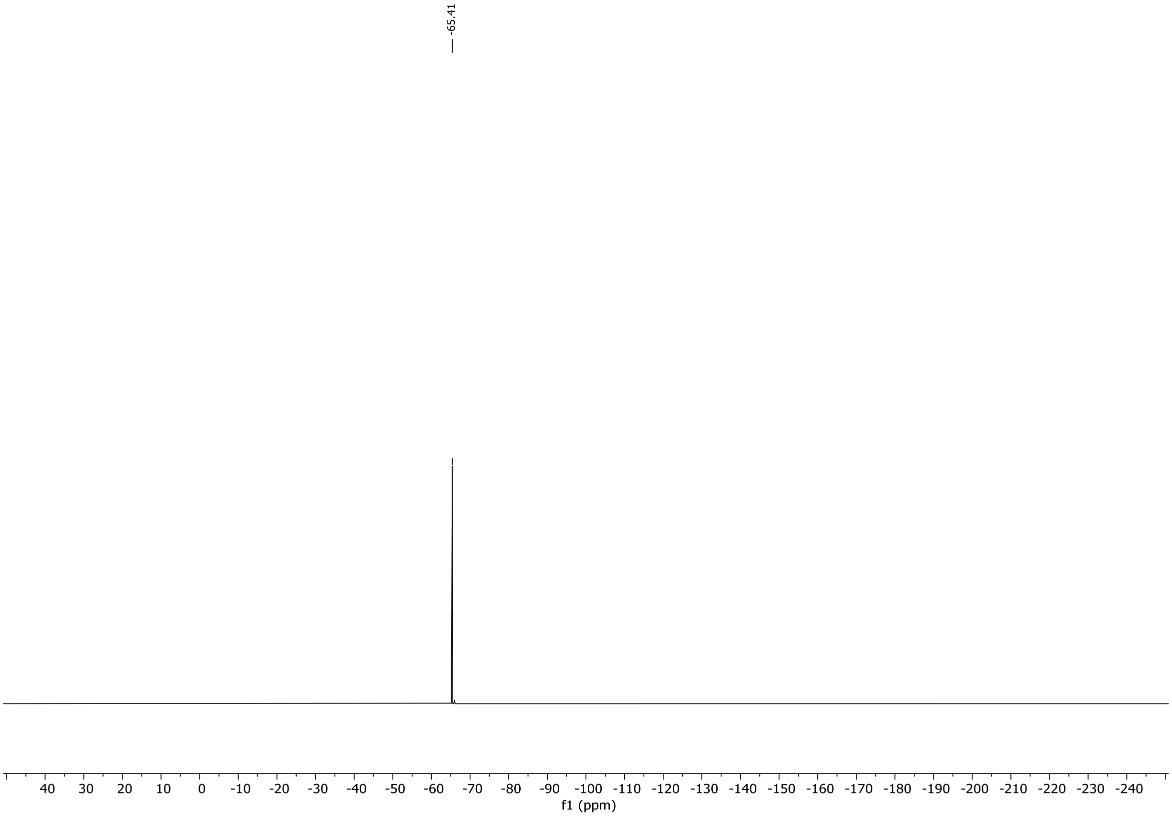

^{13}C NMR (151 MHz, $CDCl_3$) of **6g**

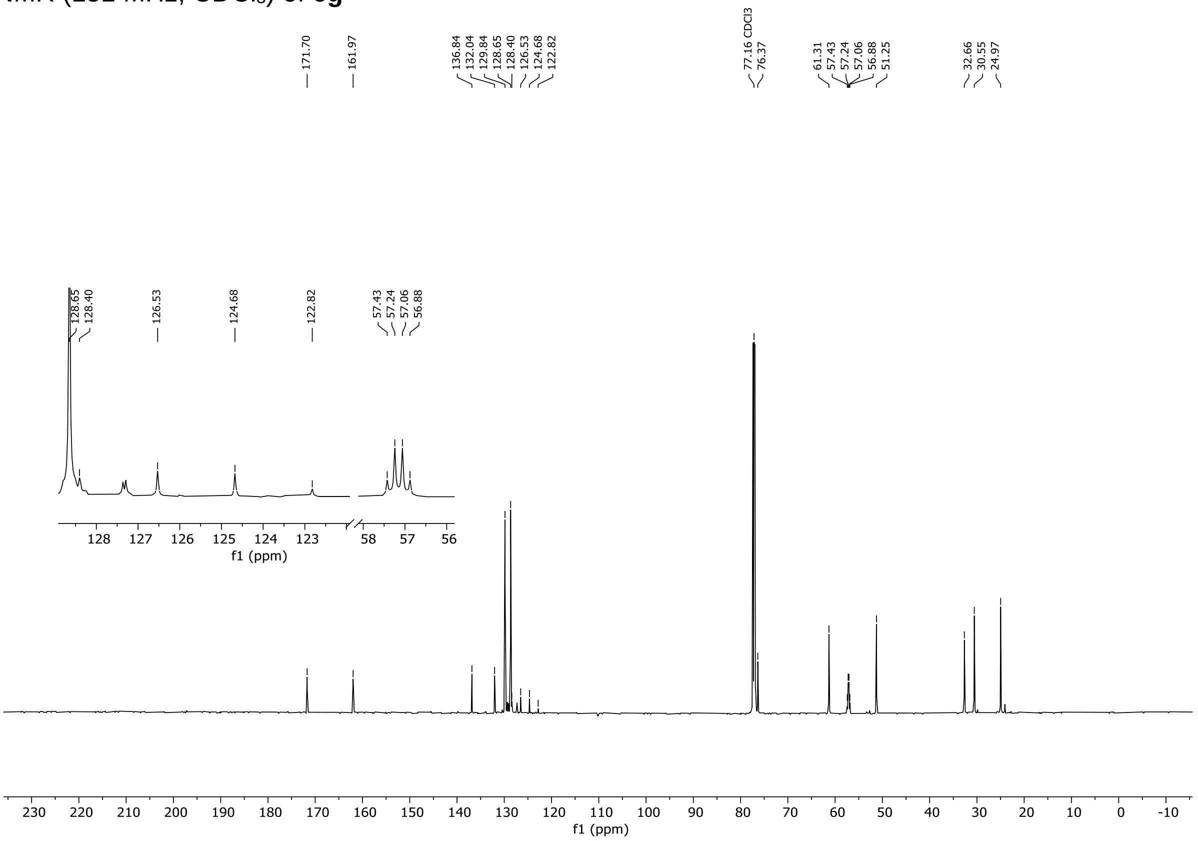

^{13}C NMR {^{1}H, ^{19}F} (126 MHz, $CDCl_3$) of **6g**

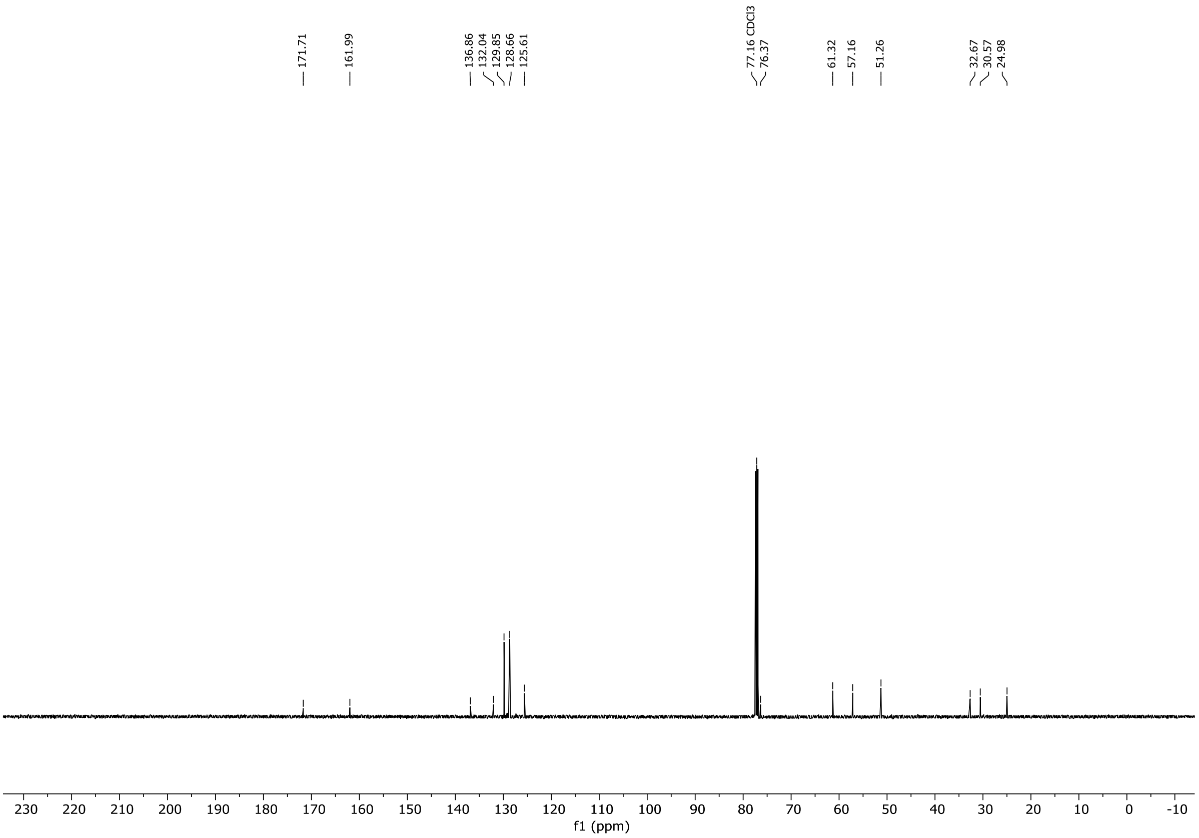

^{1}H NMR (500 MHz, $CDCl_3$) of **6h** (*see procedure*)

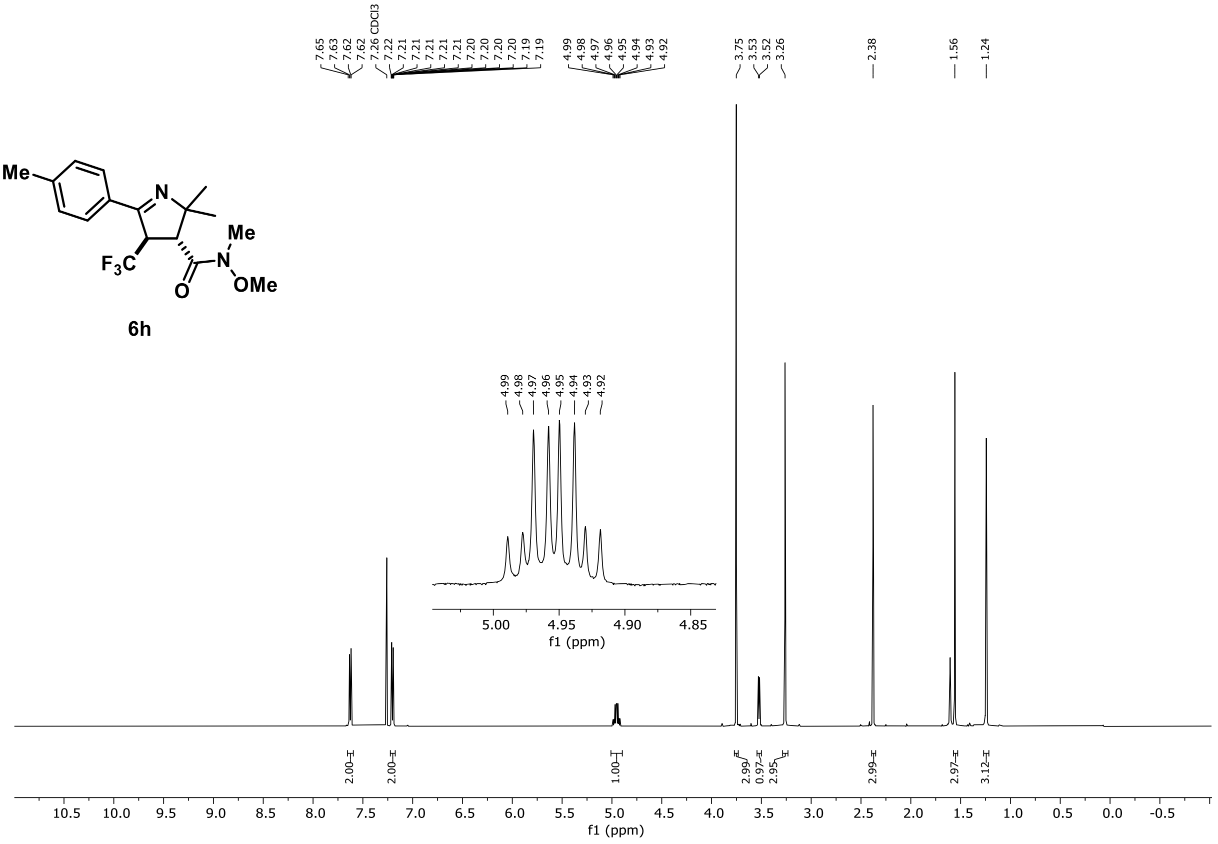

^{19}F NMR (470 MHz, $CDCl_3$) of **6h**

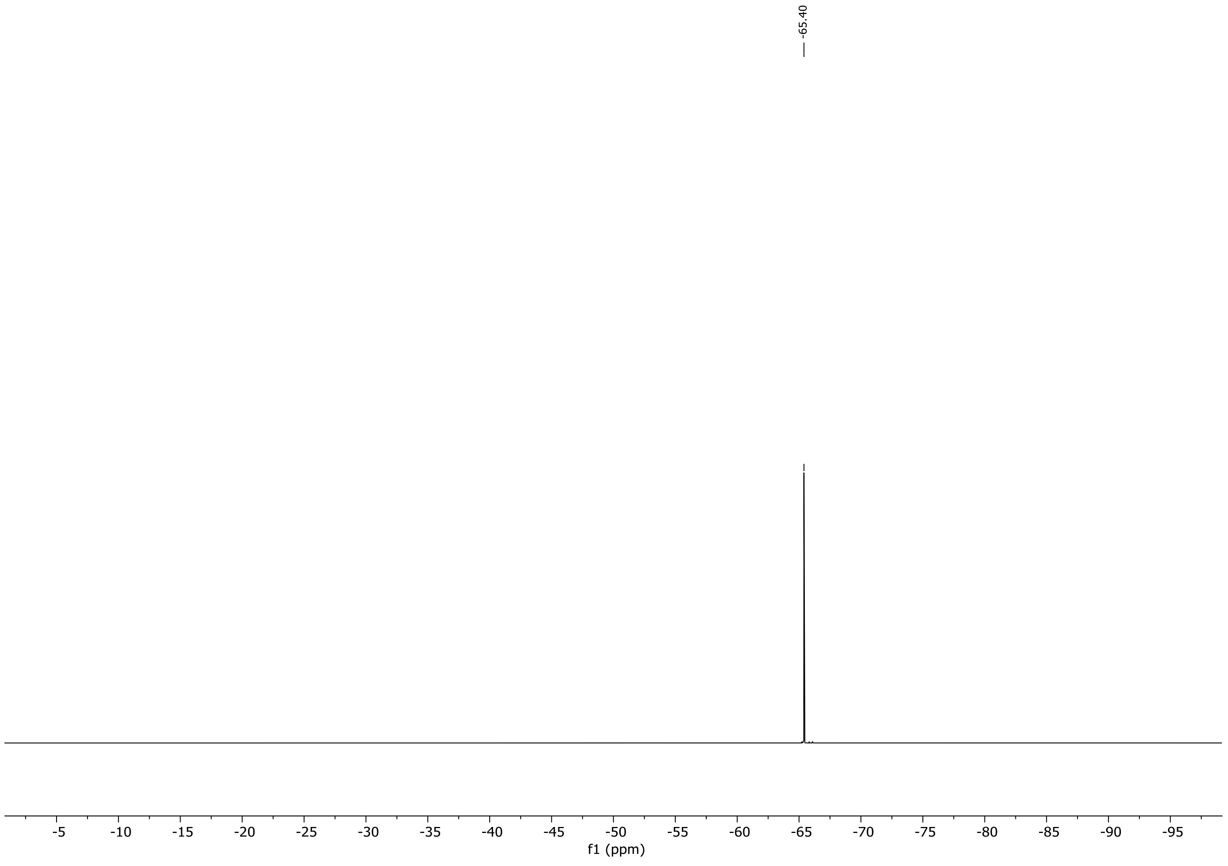

^{13}C NMR (151 MHz, $CDCl_3$) of **6h**

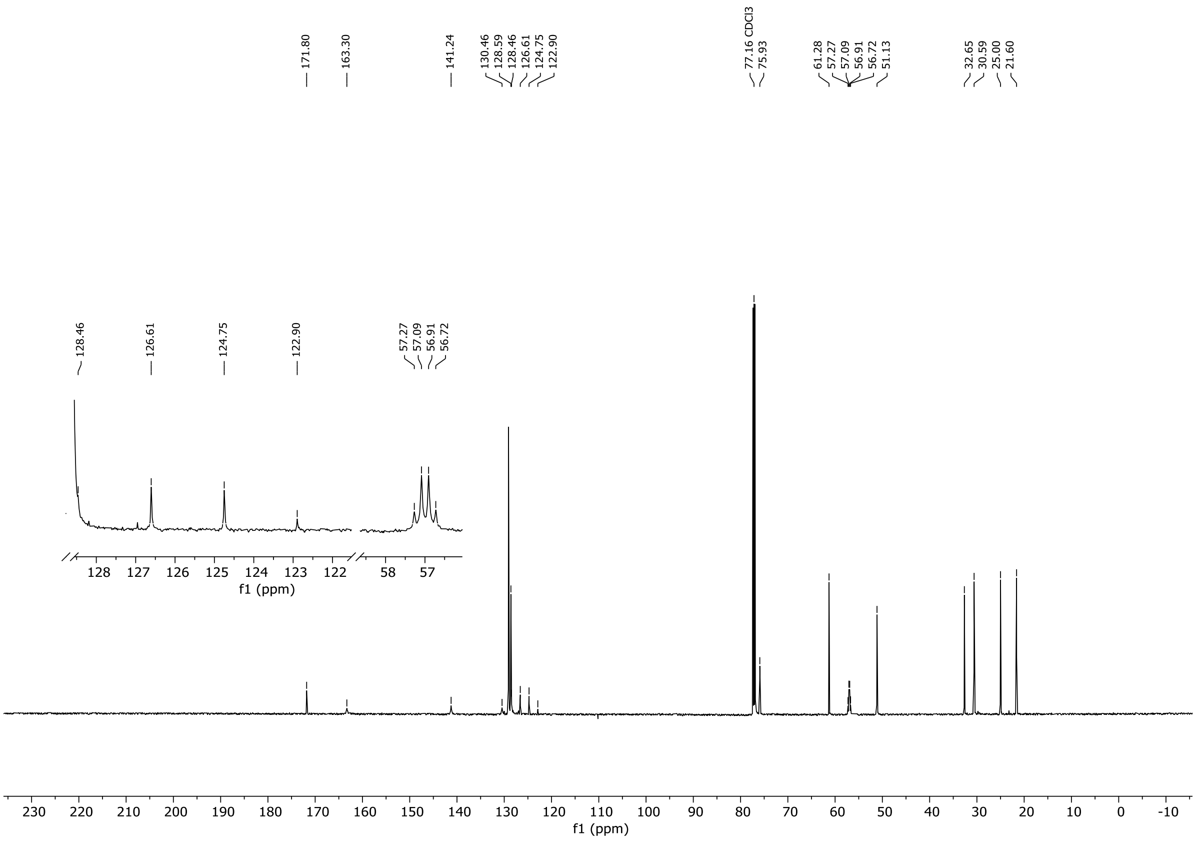

^{13}C NMR {^1H, ^{19}F} (126 MHz, $CDCl_3$) of **6h**

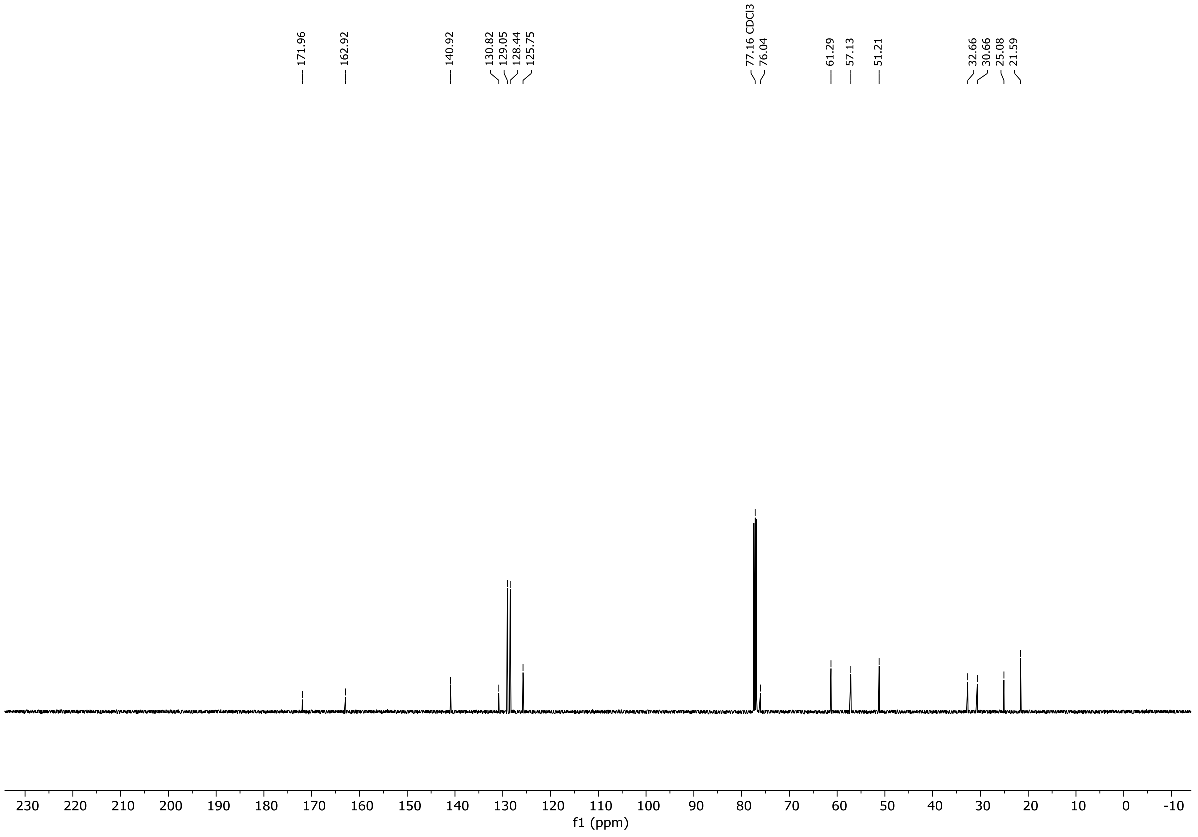

^{1}H NMR (500 MHz, $CDCl_3$) of **6i** (*see procedure*)

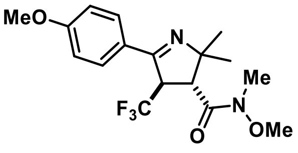

6i

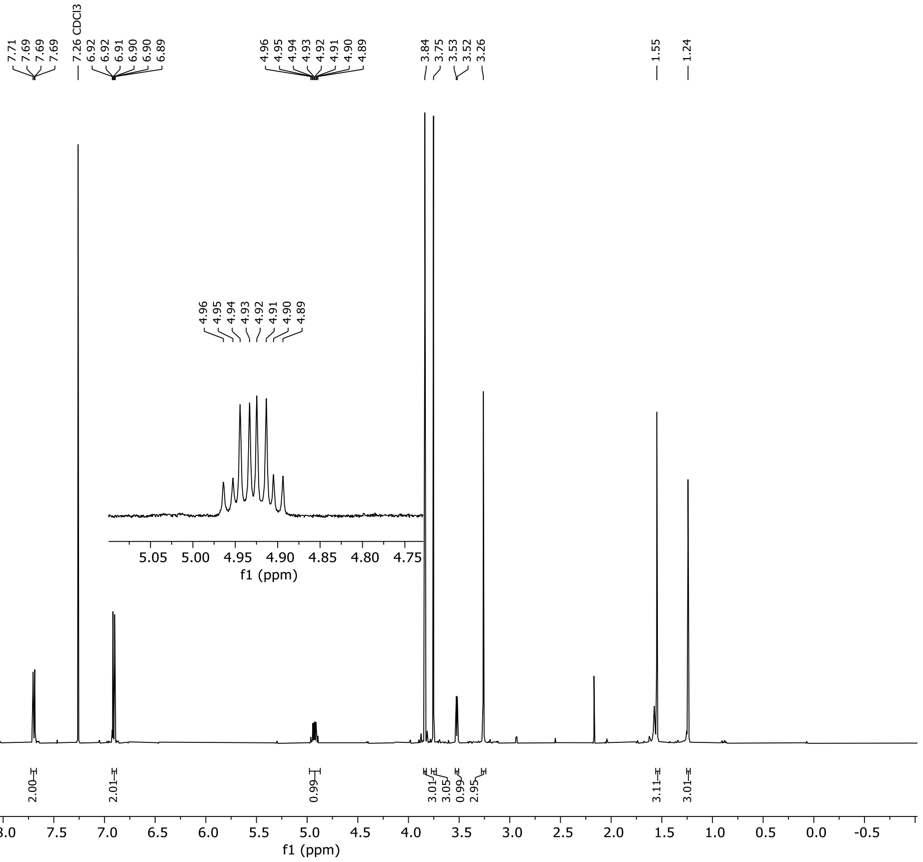

^{19}F NMR (377 MHz, $CDCl_3$) of **6i**

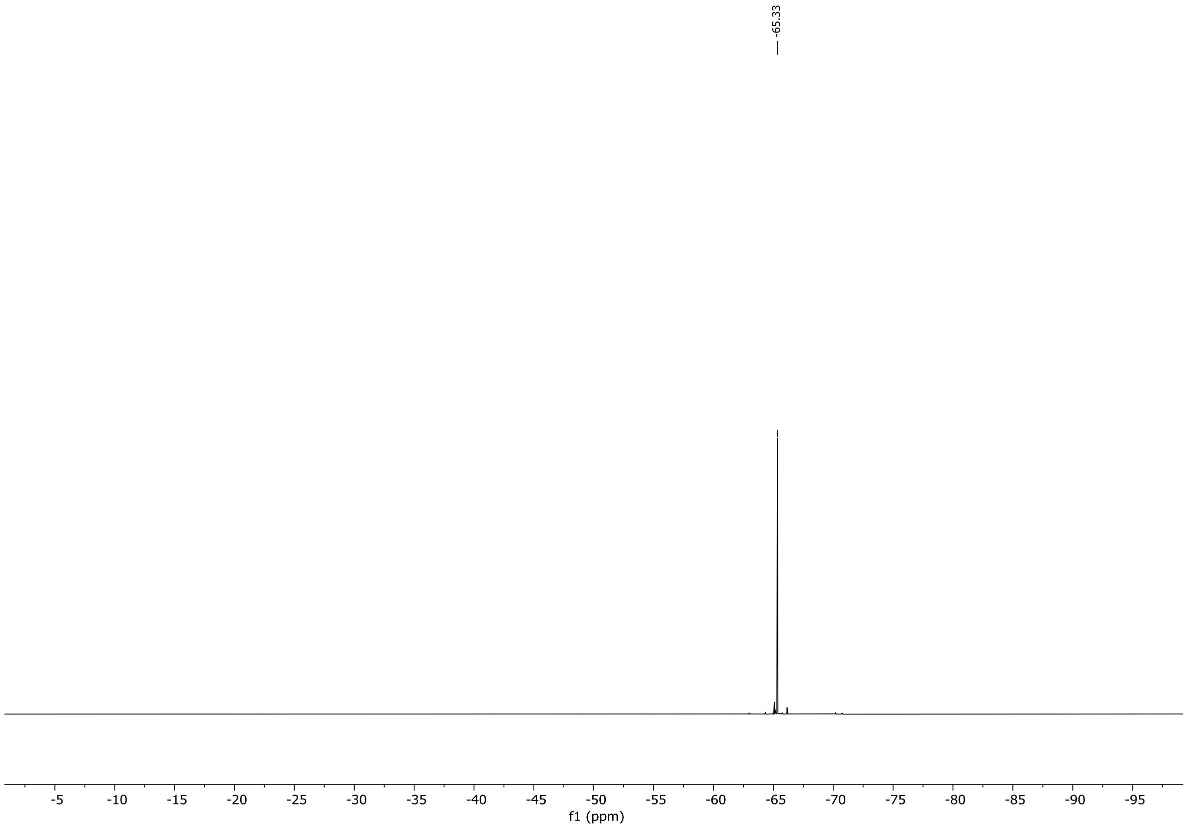

^{13}C NMR (151 MHz, $CDCl_3$) of **6i**

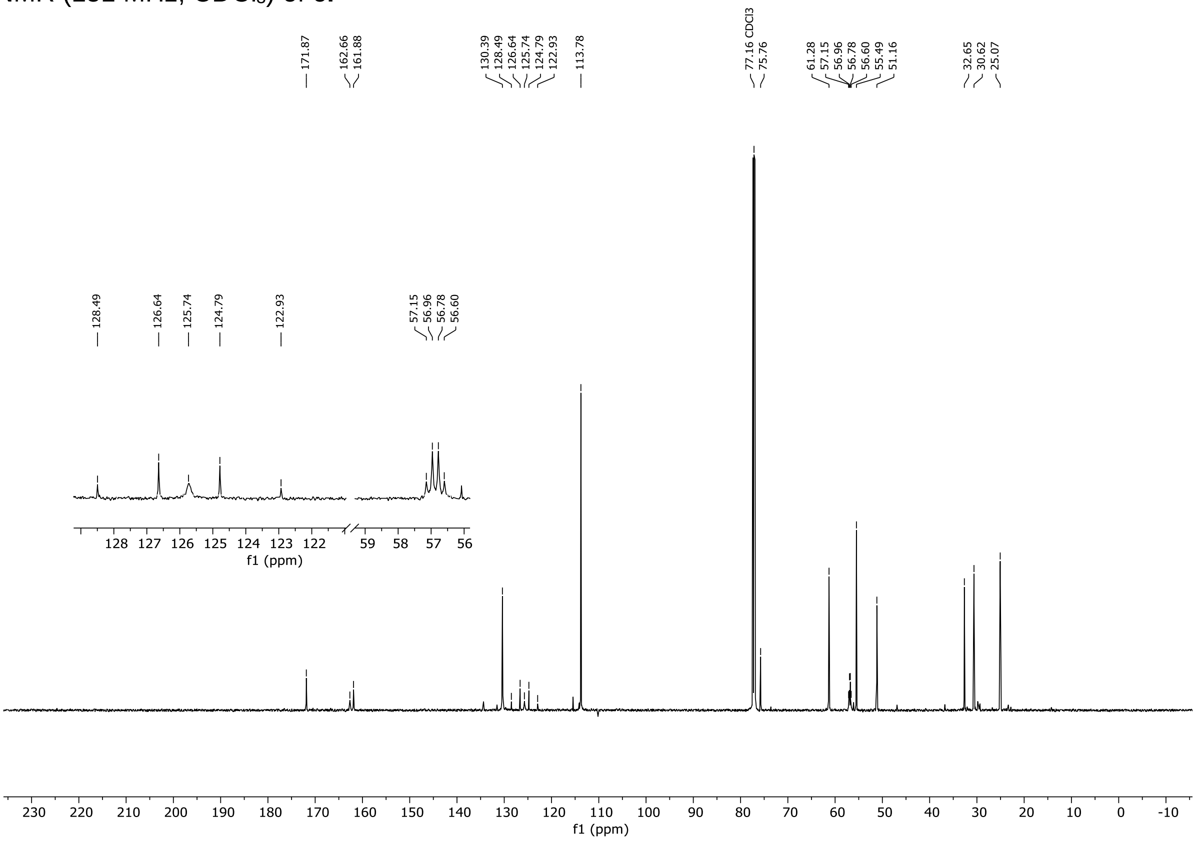

^{13}C NMR {^{1}H, ^{19}F} (126 MHz, $CDCl_3$) of **6i**

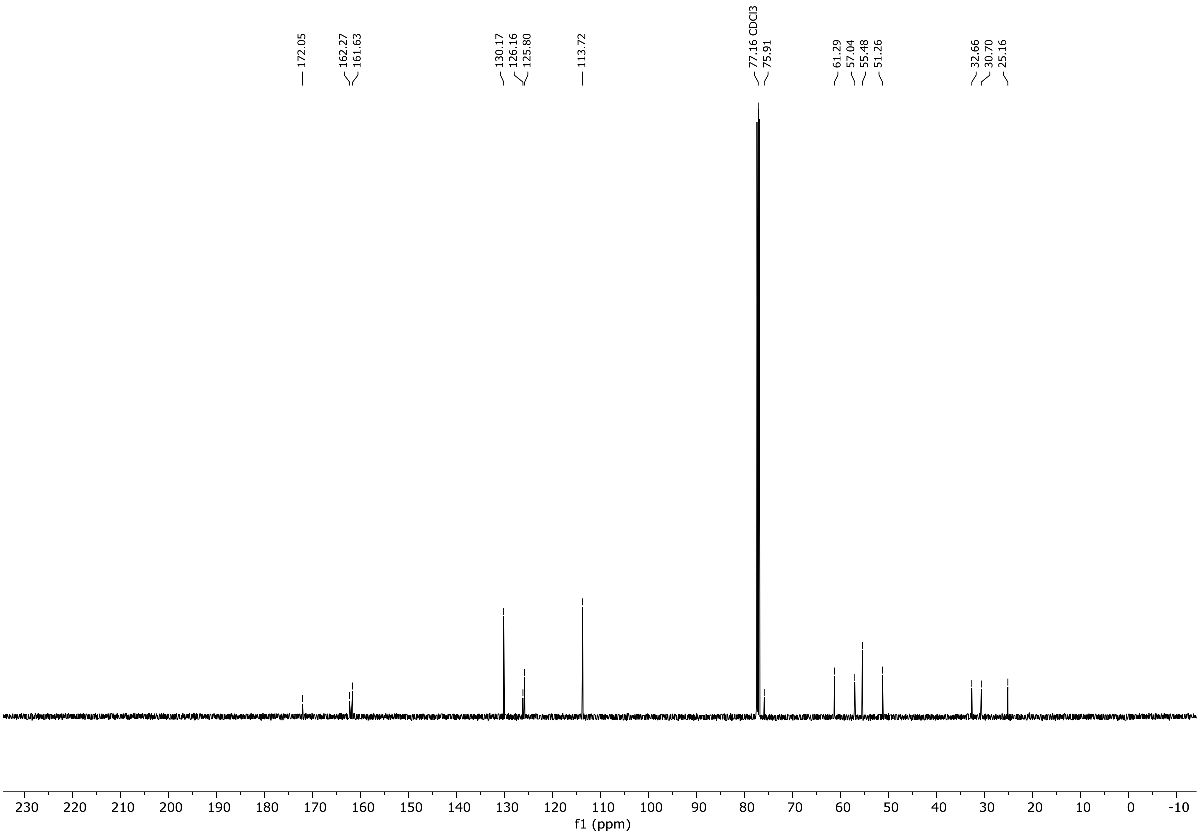

^{1}H NMR (400 MHz, $CDCl_3$) of **6l** (*see procedure*)

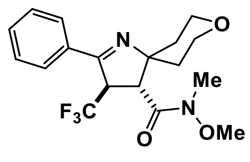

6l

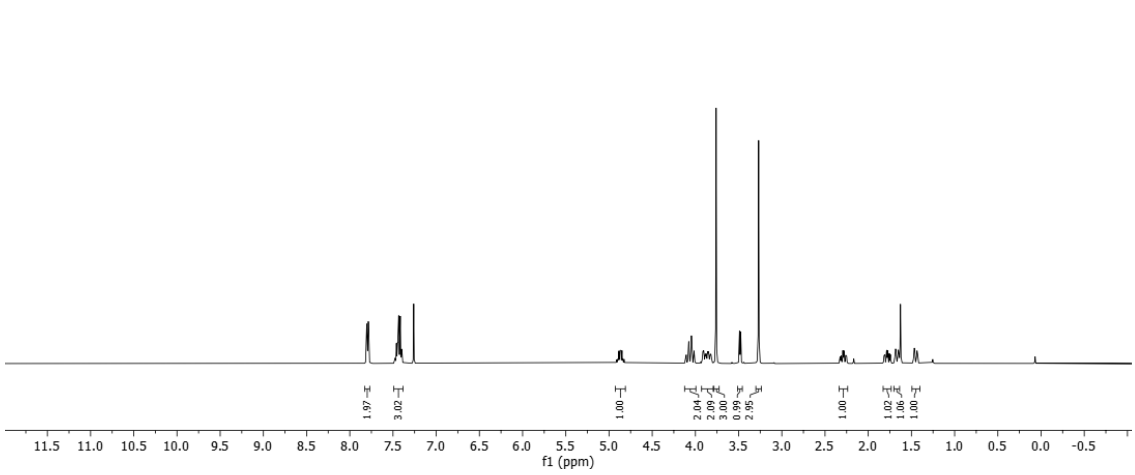

^{13}C NMR (126 MHz, $CDCl_3$) of **6l**

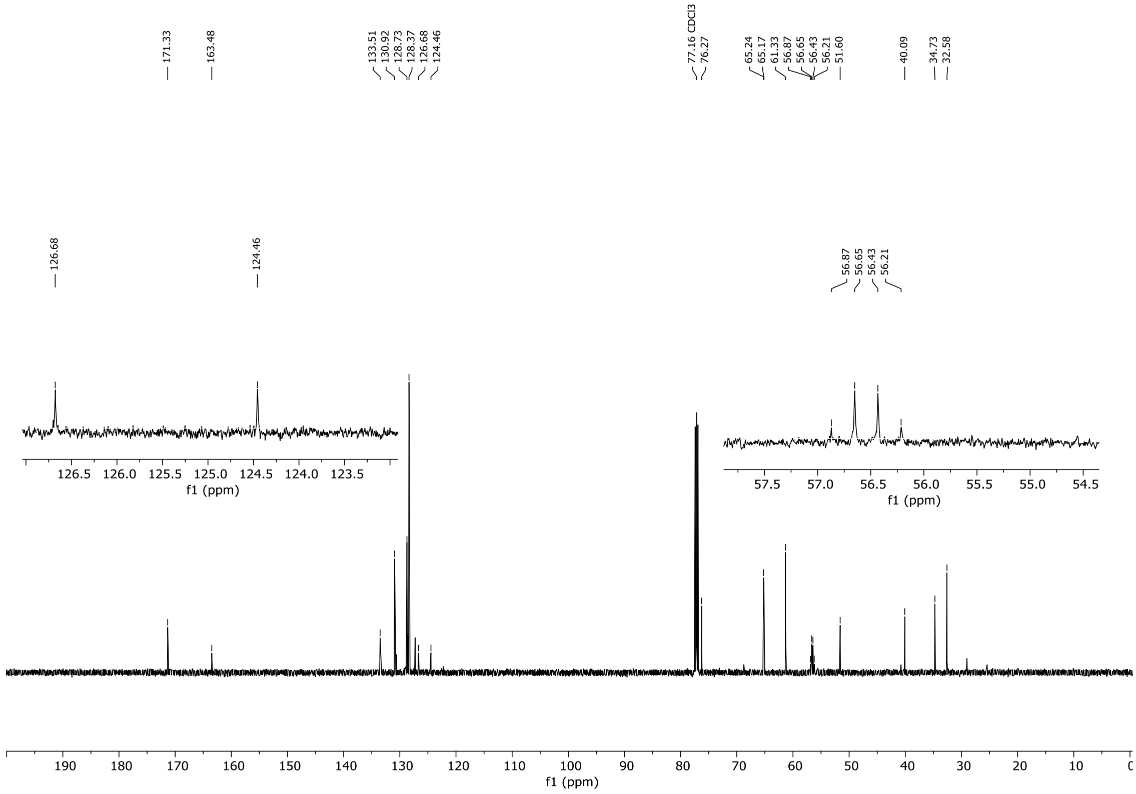

^{13}C NMR {^{1}H, ^{19}F} (126 MHz, $CDCl_3$) of **6l**

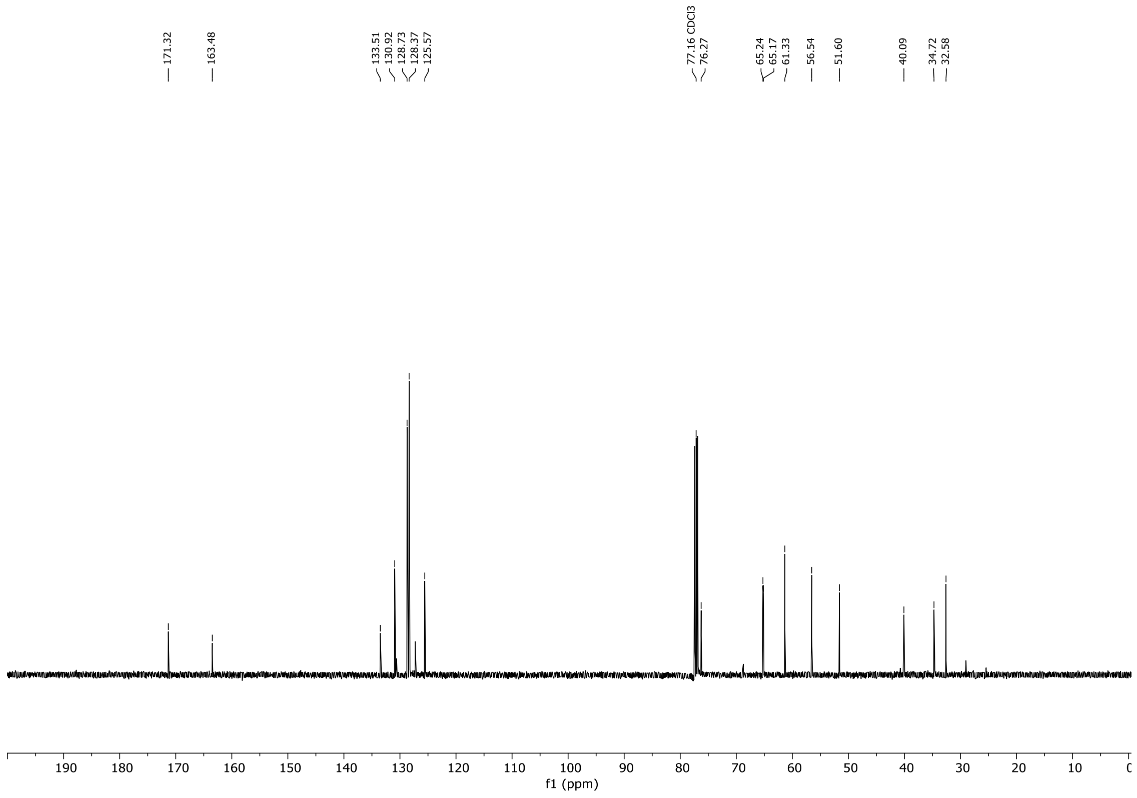

^{19}F NMR (377 MHz, $CDCl_3$) of **6l**

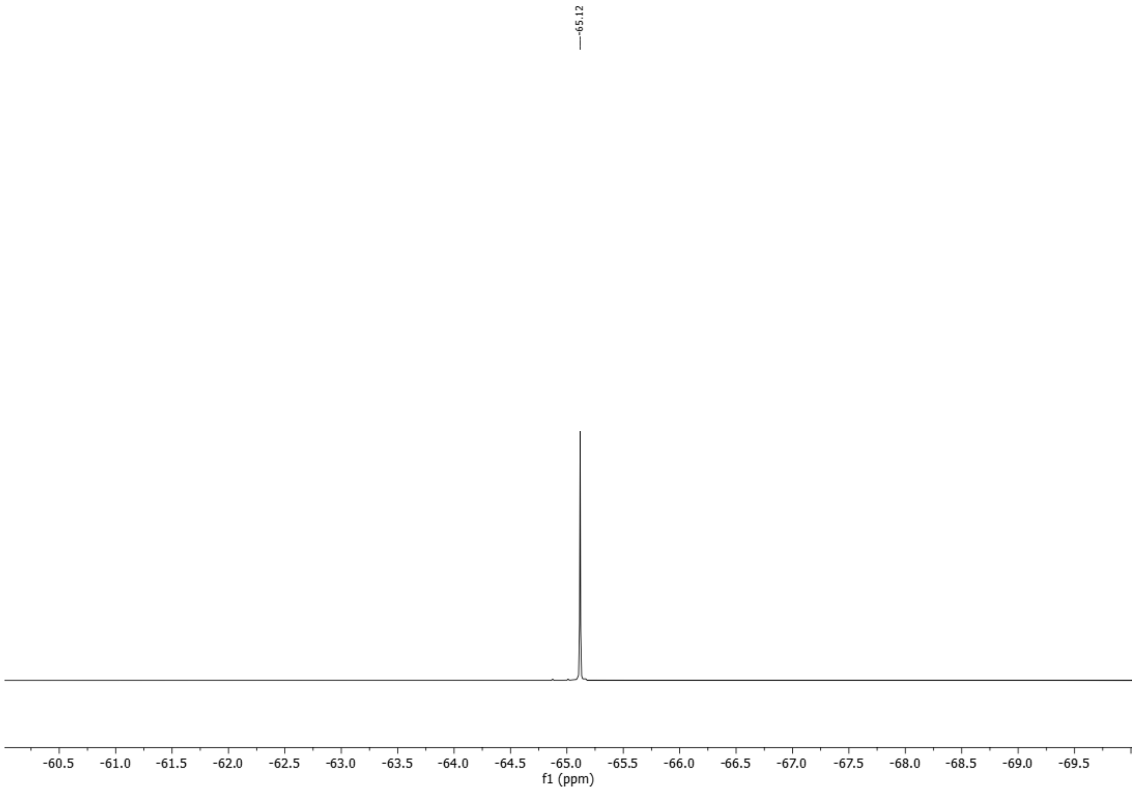

^{1}H NMR (400 MHz, $CDCl_3$) of **6m** (*see procedure*)

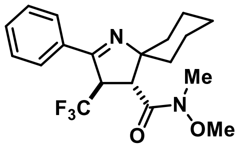

6m

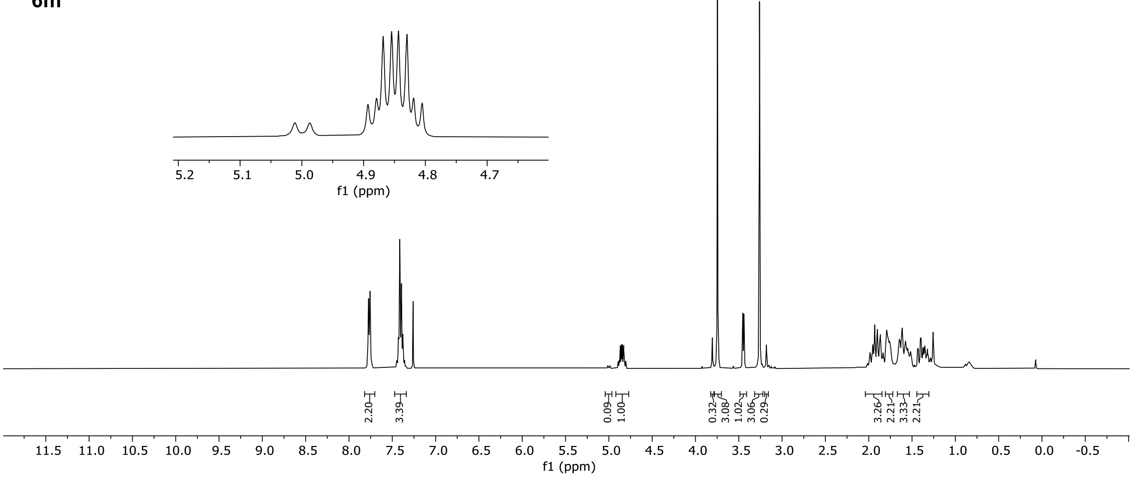

^{13}C NMR (151 MHz, $CDCl_3$) of **6m**

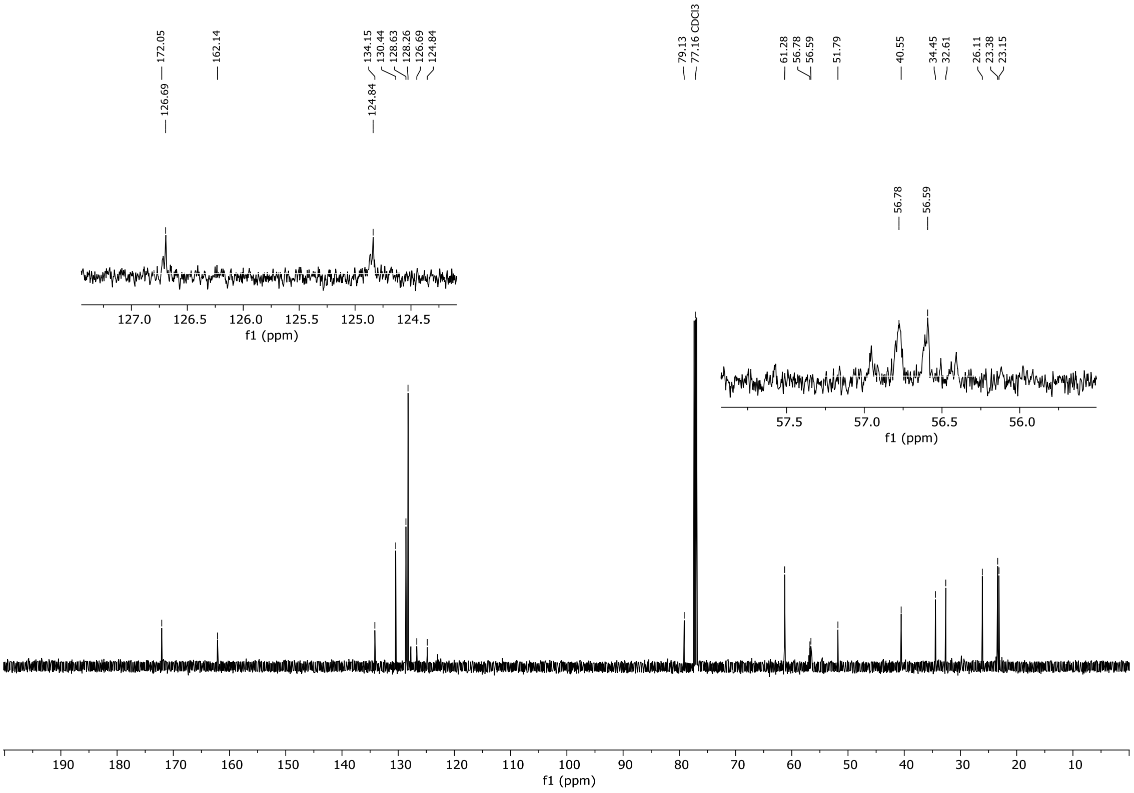

^{13}C NMR {^{1}H, ^{19}F} (151 MHz, $CDCl_3$) of **6m**

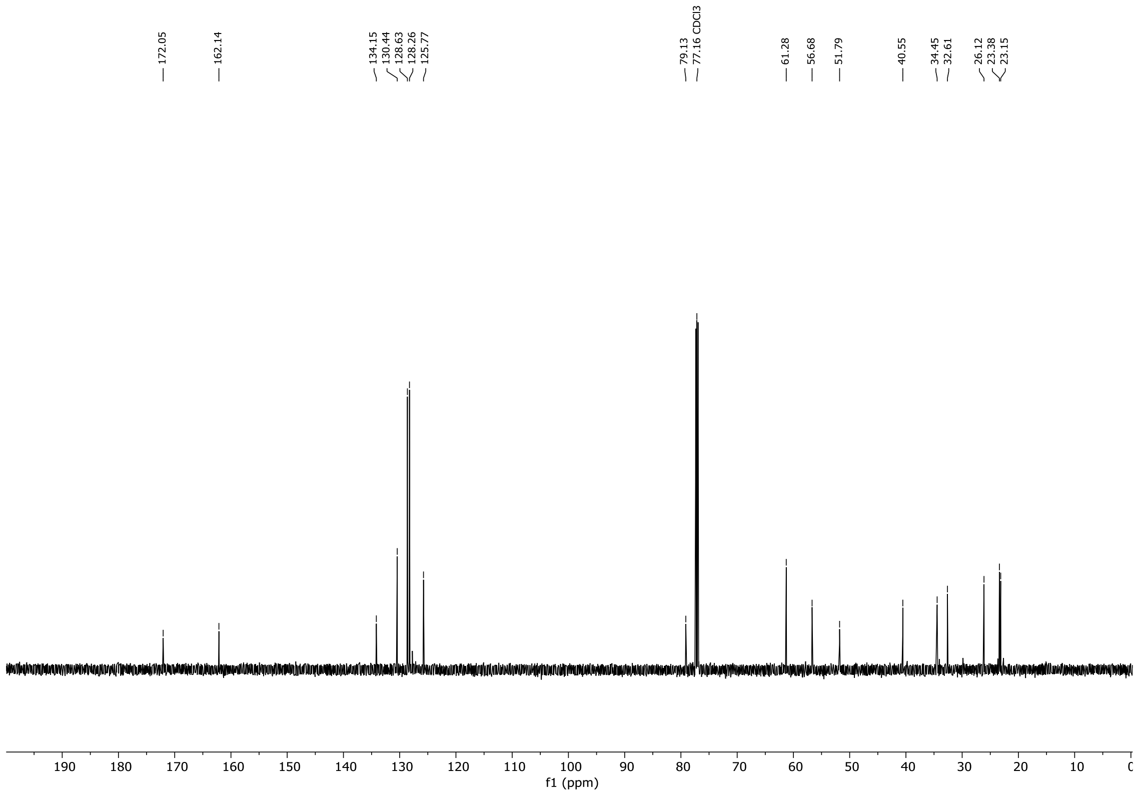

^{19}F NMR (377 MHz, $CDCl_3$) of **6m**

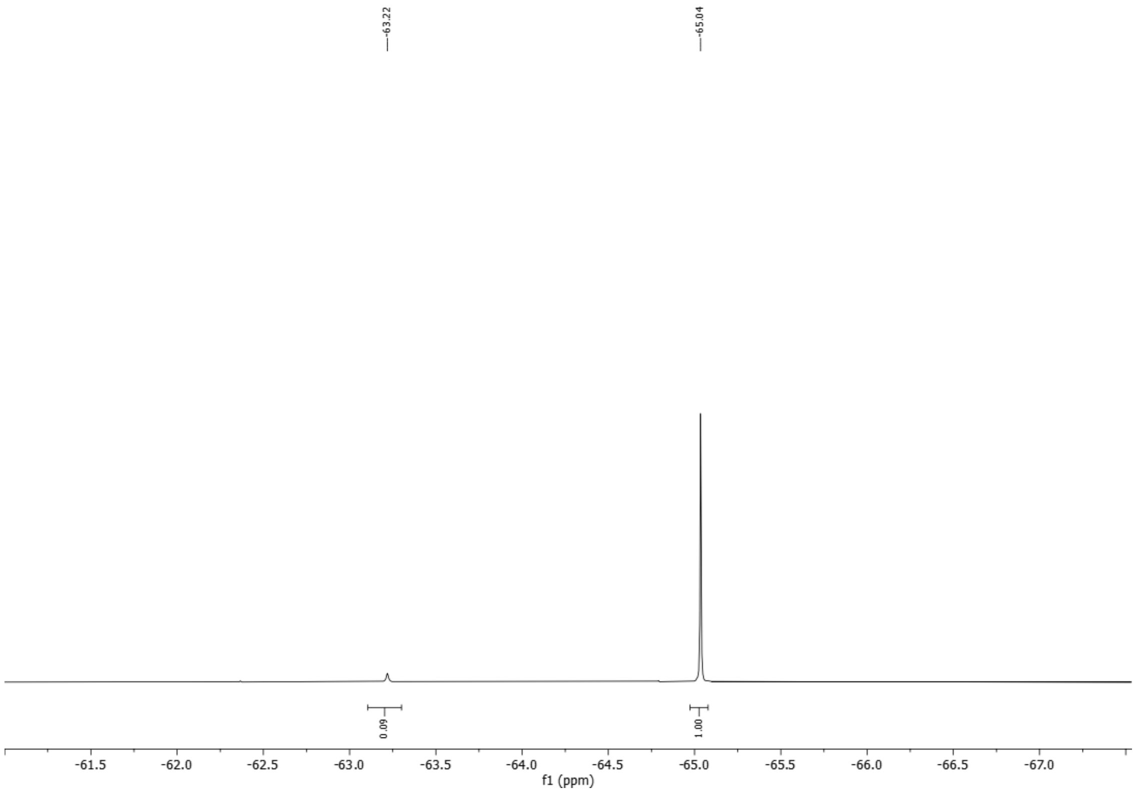

^{1}H NMR (400 MHz, MeOD) of **7a** (*see procedure*)

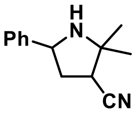

7a

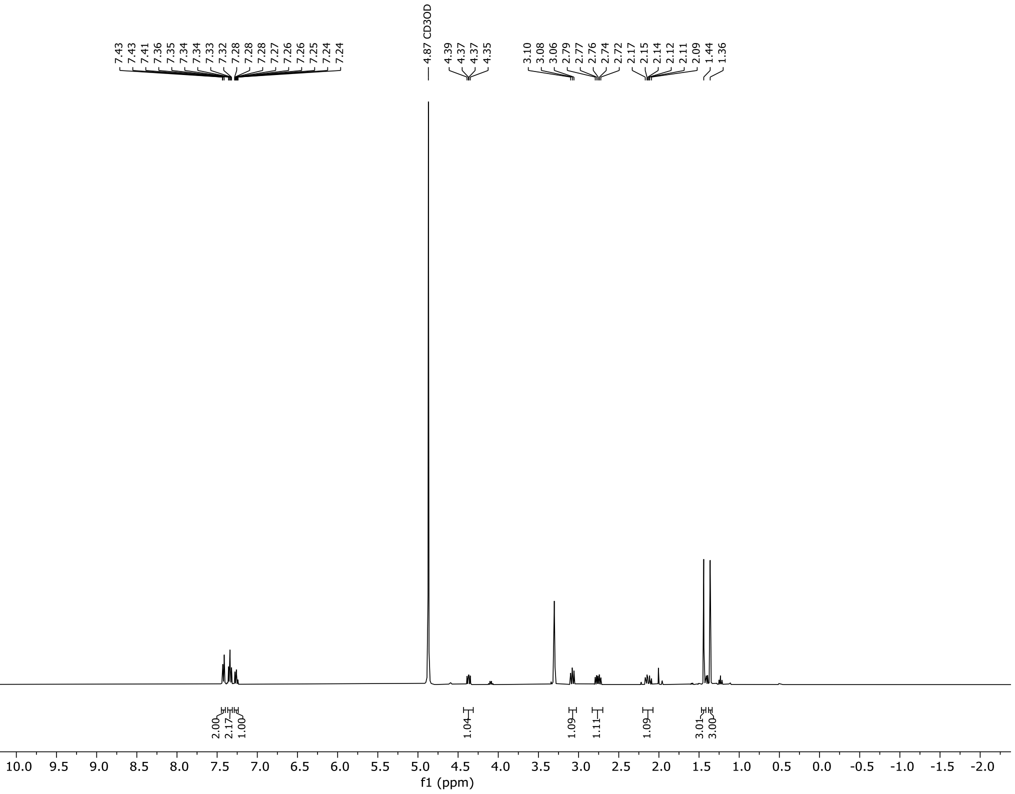

^{13}C NMR (126 MHz, MeOD) of **7a**

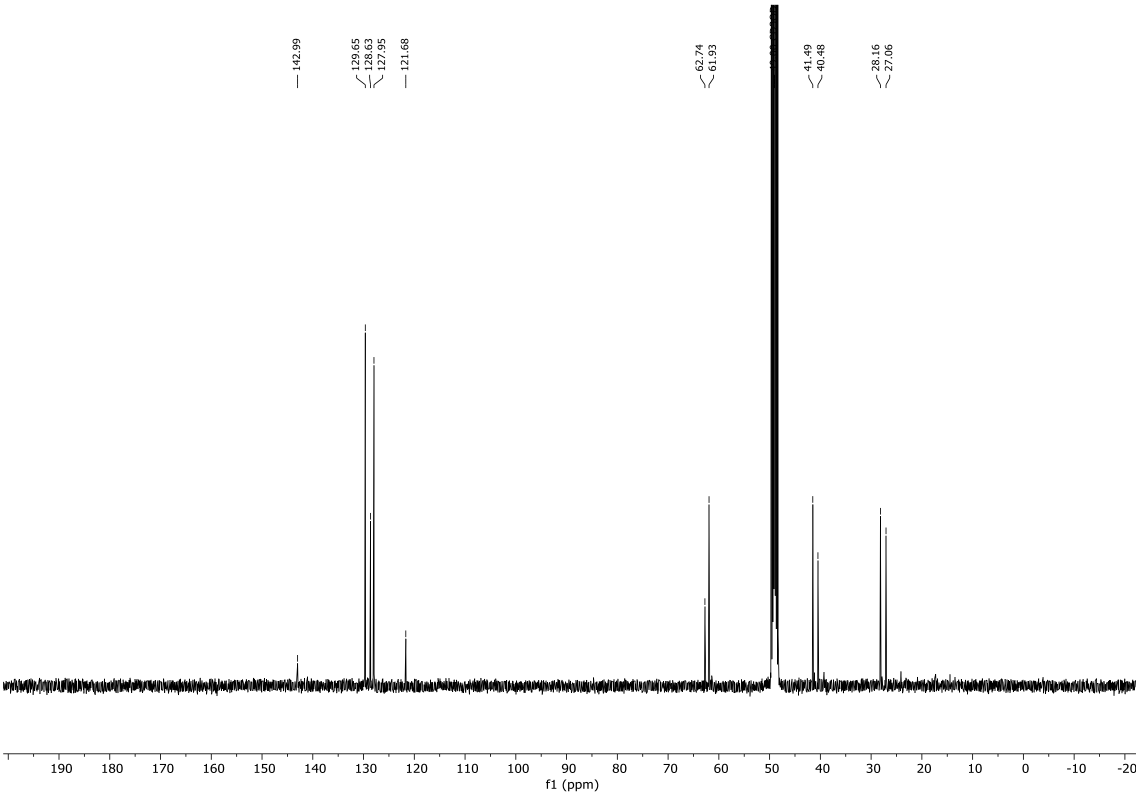

^{1}H NMR (599 MHz, $CDCl_3$) of **7b** (*see procedure*)

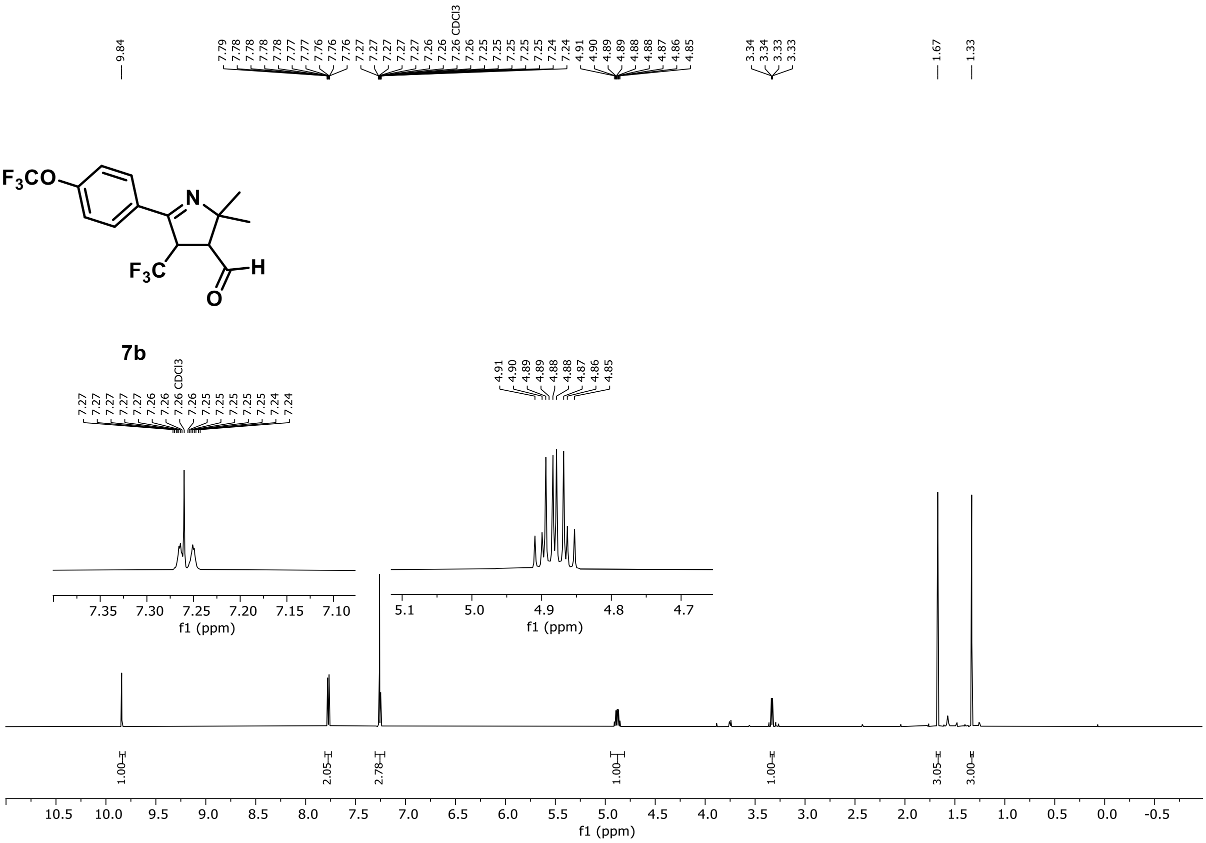

^{19}F NMR (377 MHz, $CDCl_3$) of **7b**

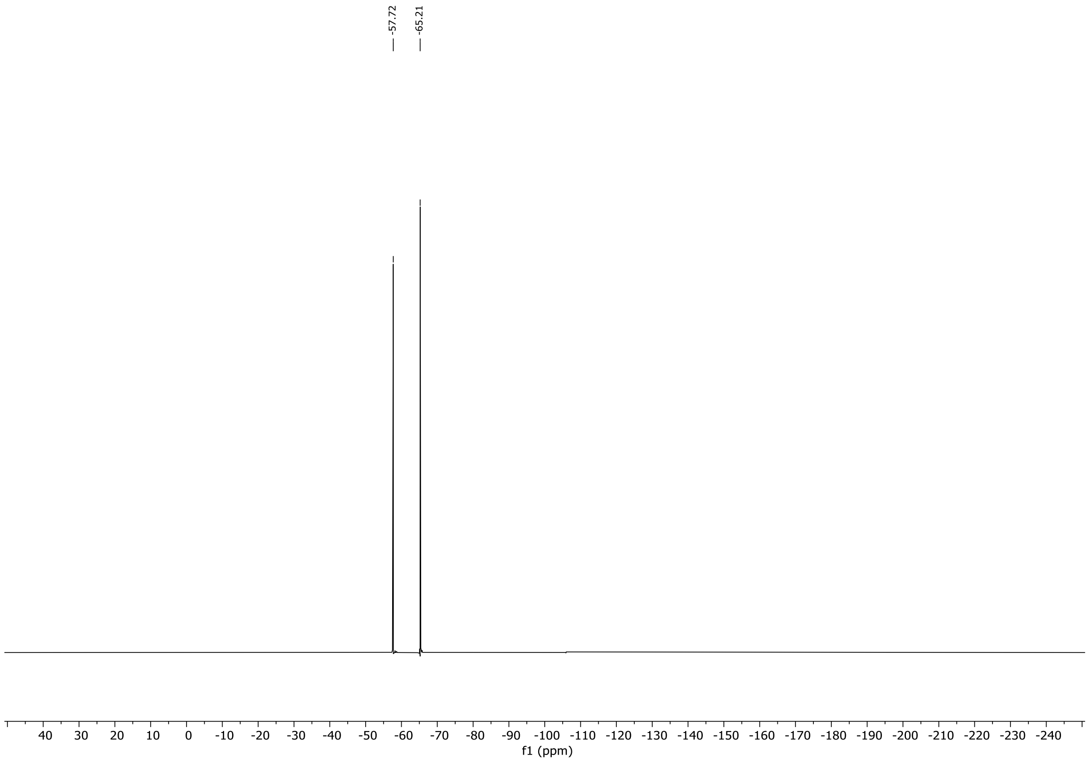

^{13}C NMR (151 MHz, $CDCl_3$) of **7b**

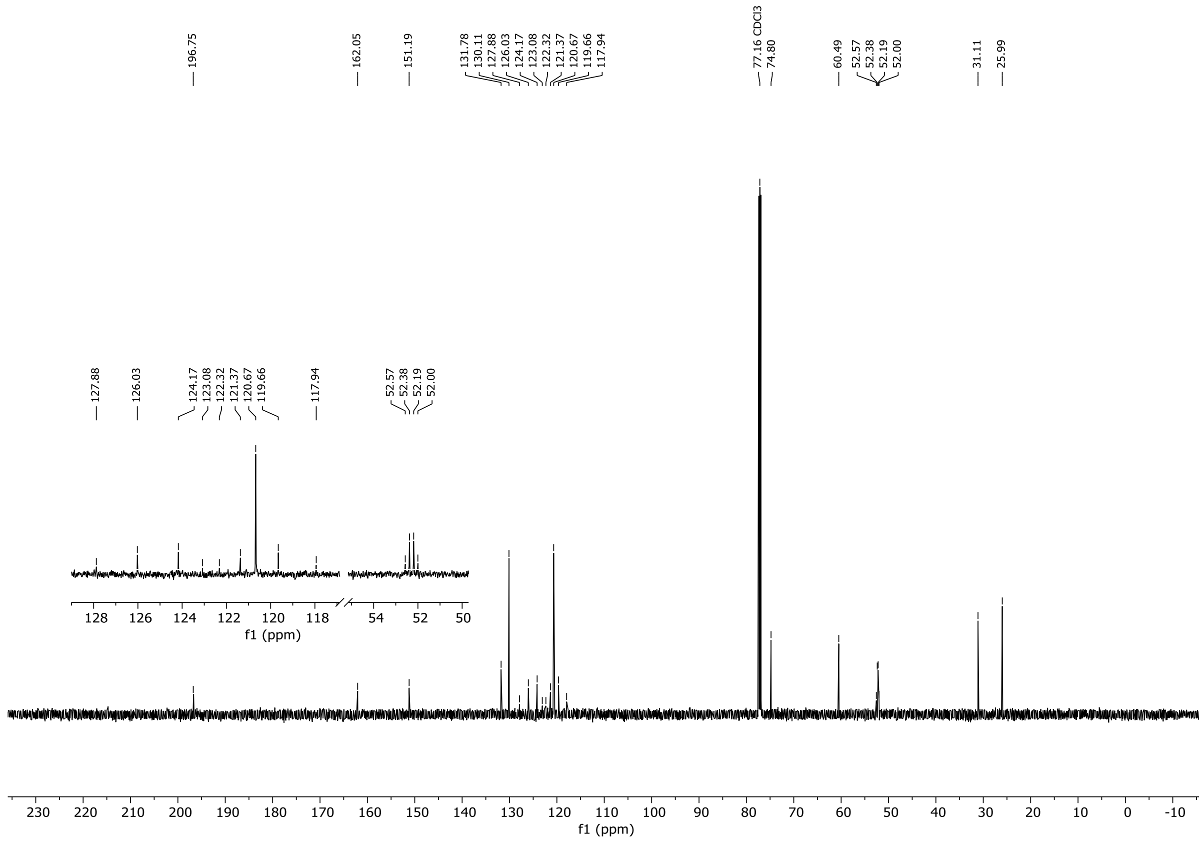

^{13}C NMR {^{1}H, ^{19}F} (151 MHz, $CDCl_3$) of **7b**

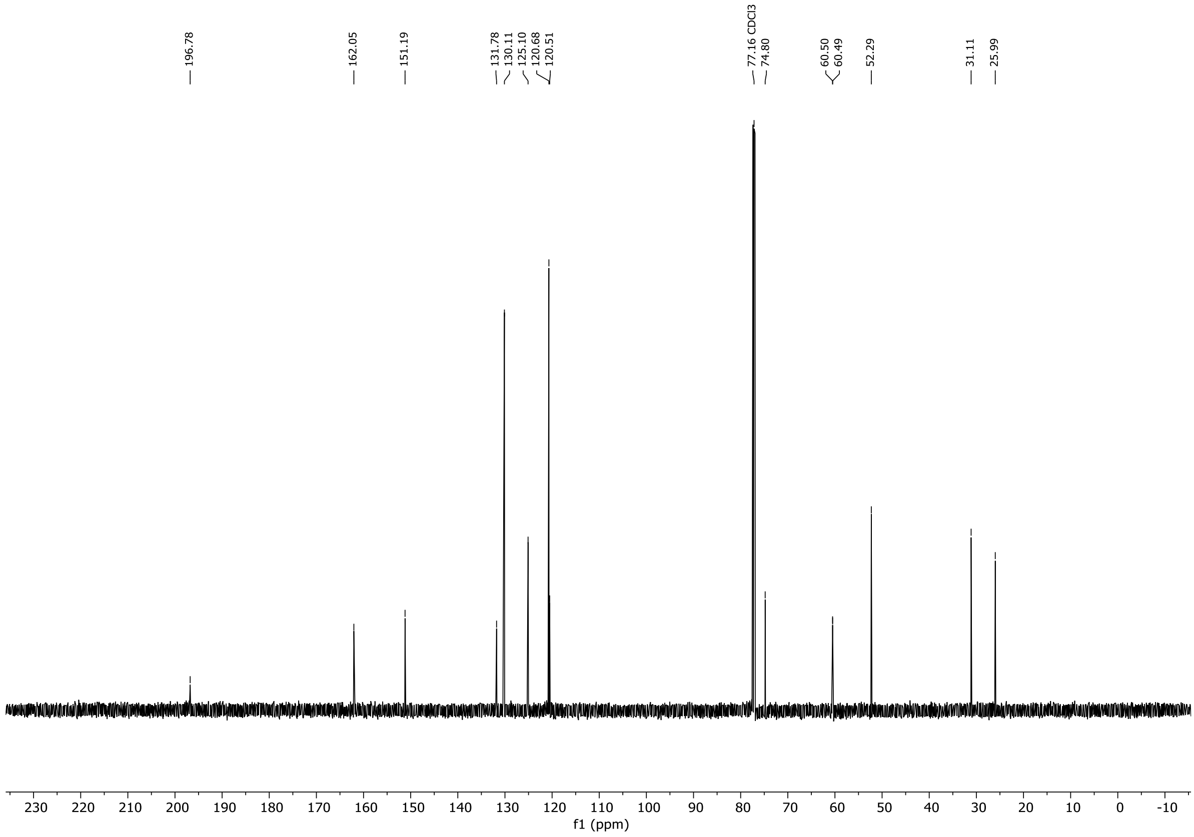

^{1}H NMR (400 MHz, MeOD) of **7c** (*see procedure*)

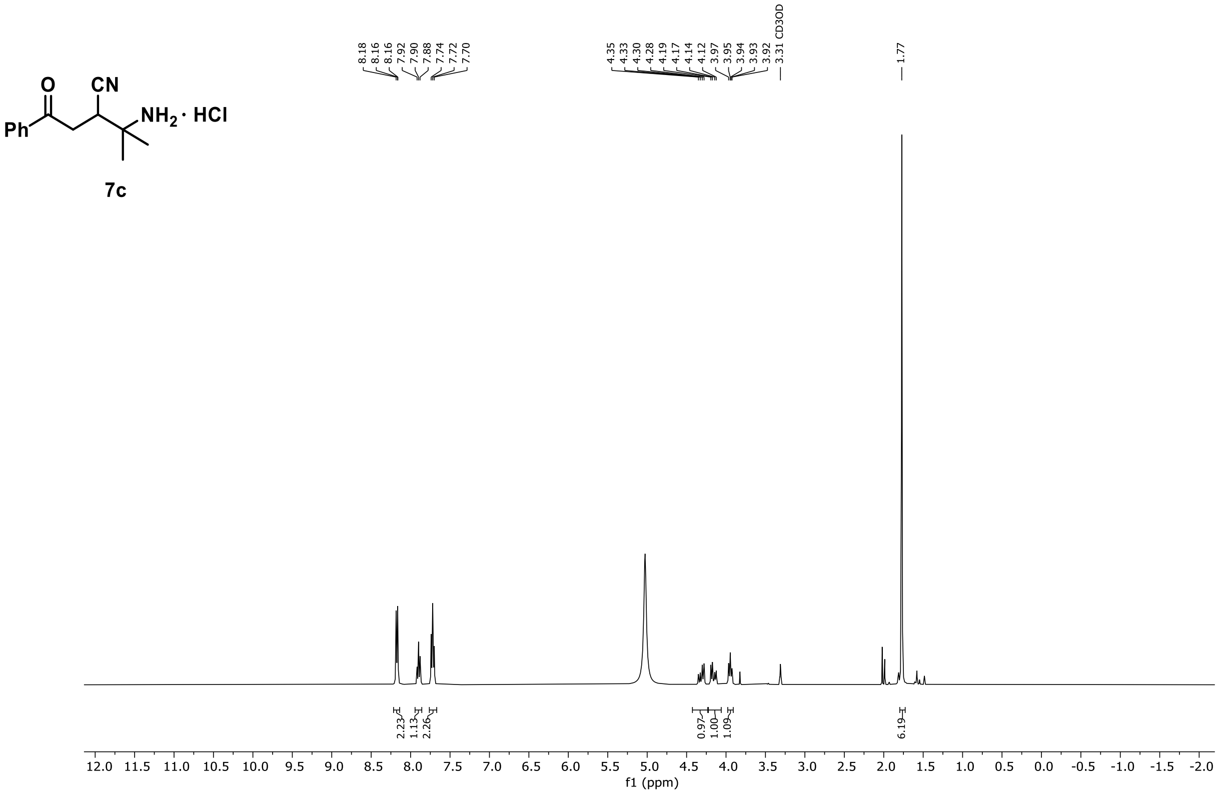

7c

^{13}C NMR (126 MHz, MeOD) of **7c**

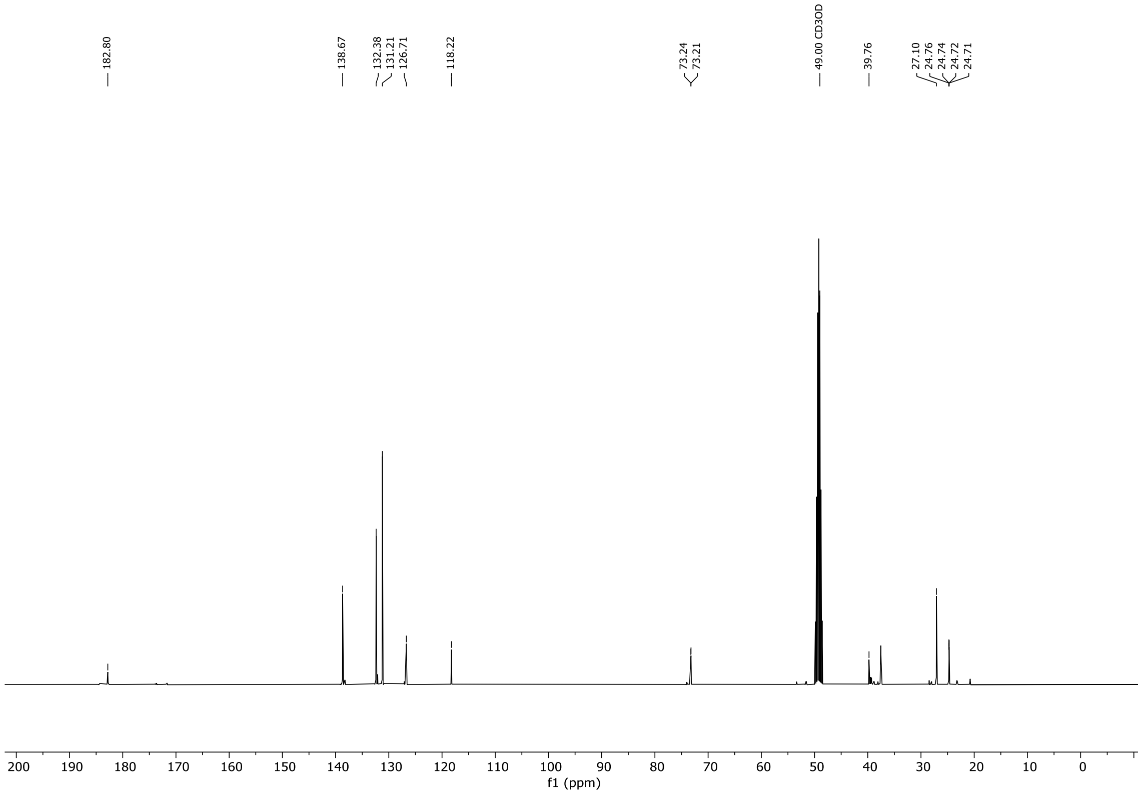

^{1}H NMR (400 MHz, $CDCl_3$) of **7d** (see procedure)

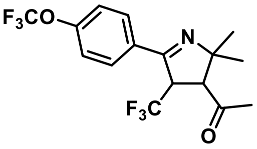
7d

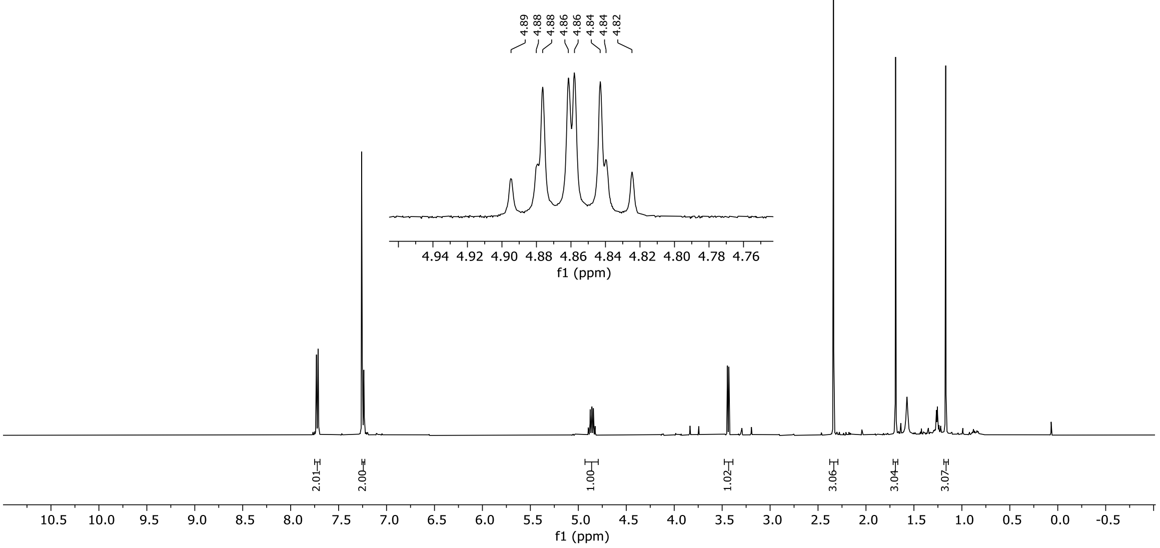

^{13}C NMR (126 MHz, $CDCl_3$) of **7d**

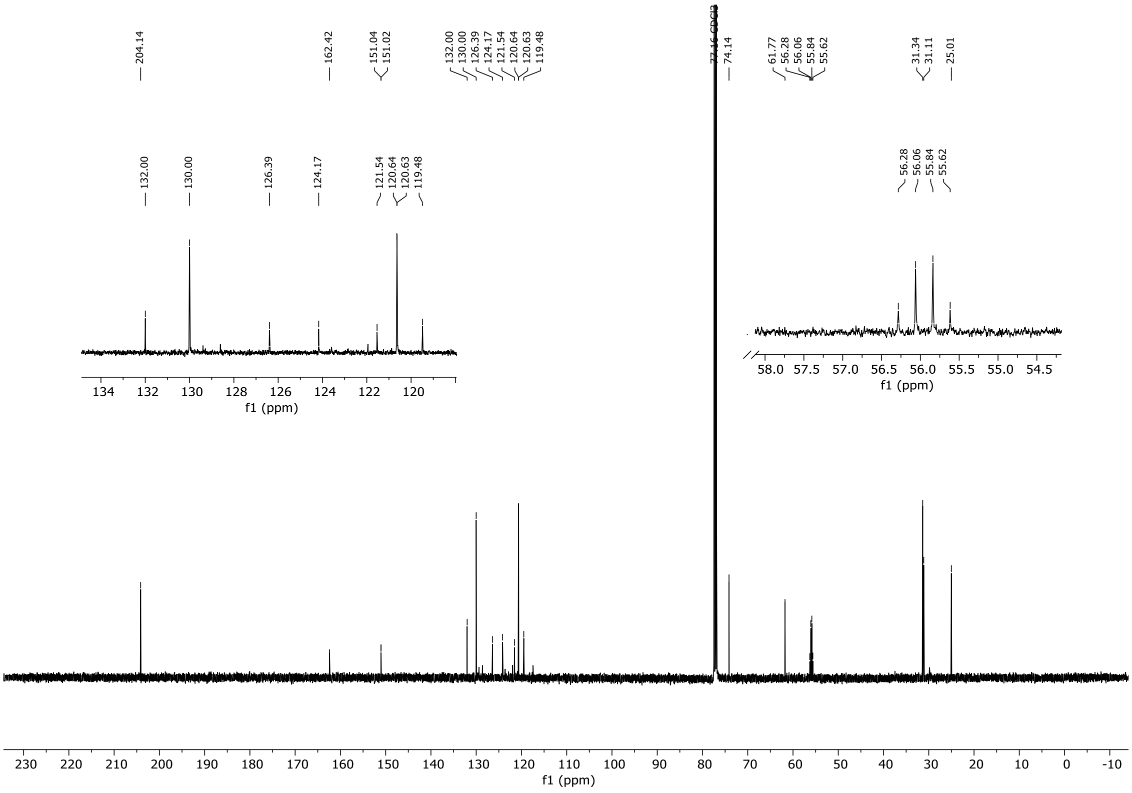

^{13}C NMR {^{1}H, ^{19}F} (126 MHz, $CDCl_3$) of **7d**

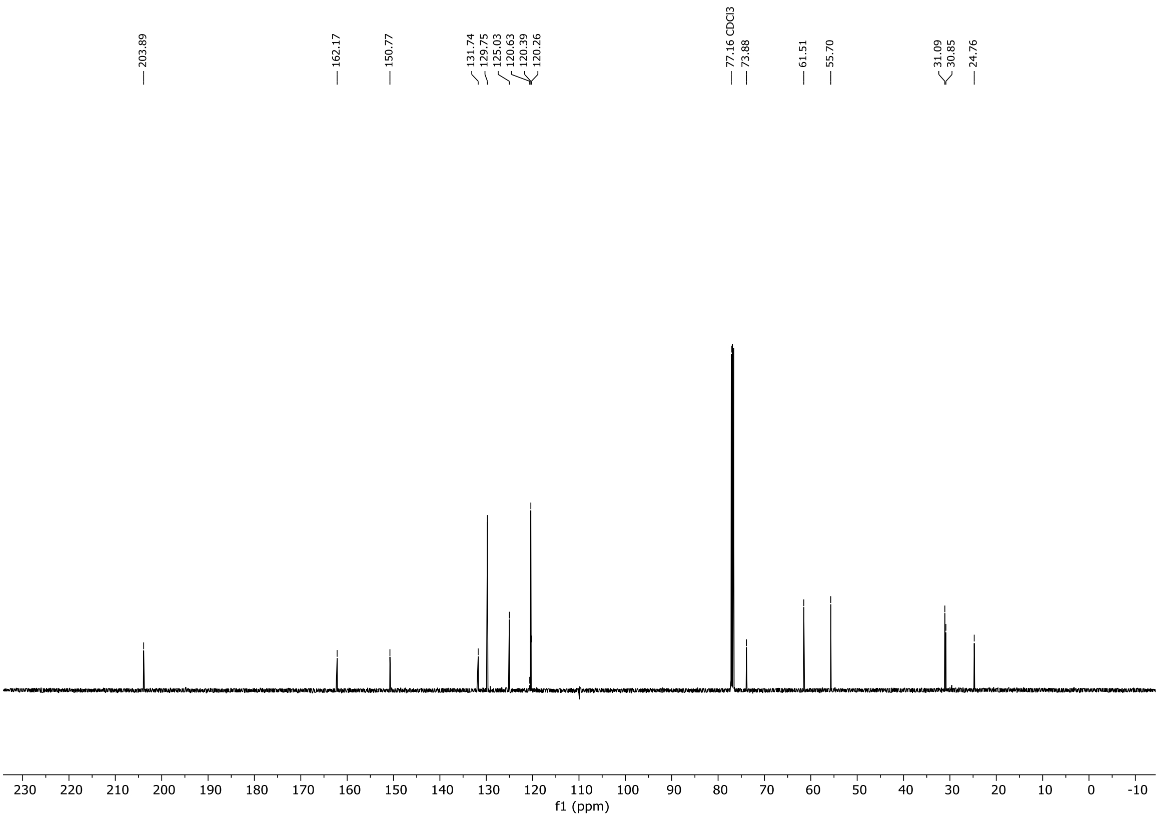

^{19}F NMR (376 MHz, $CDCl_3$) of **7d**

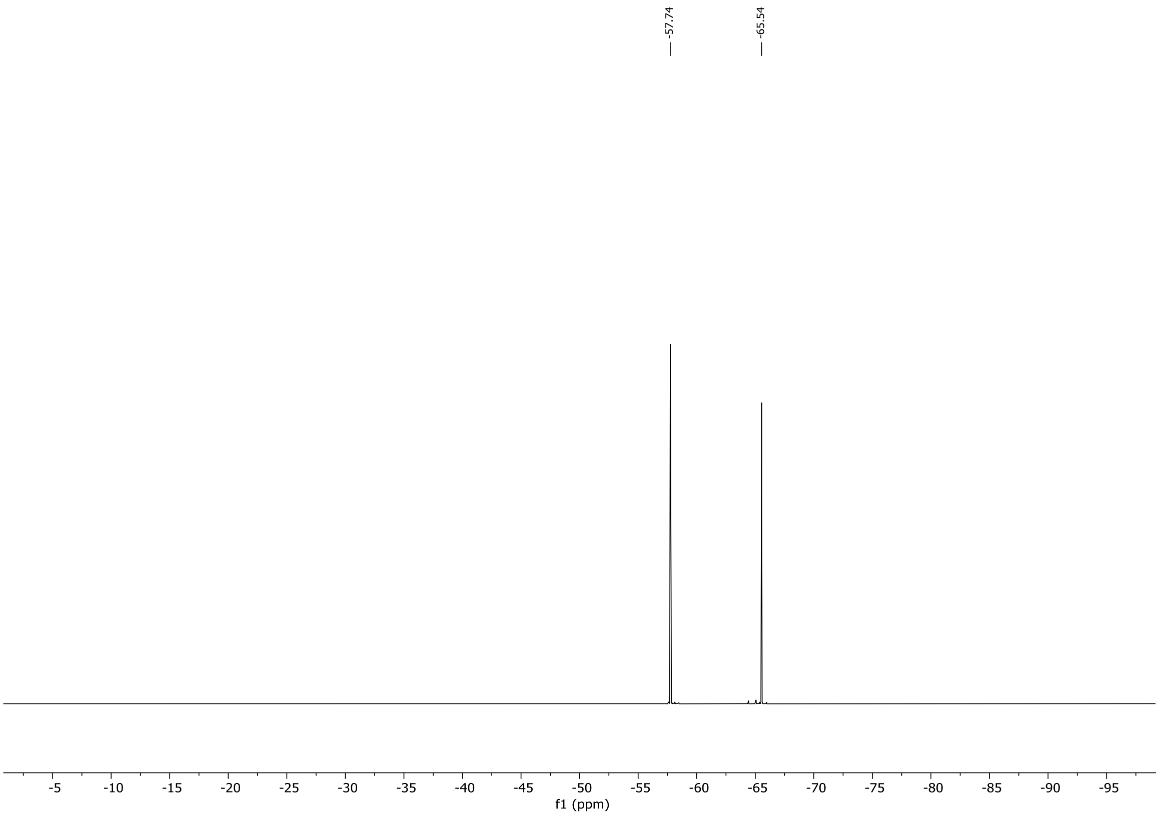

^{1}H NMR (400 MHz, $CDCl_3$) of **7e** (see procedure)

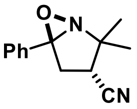

7e

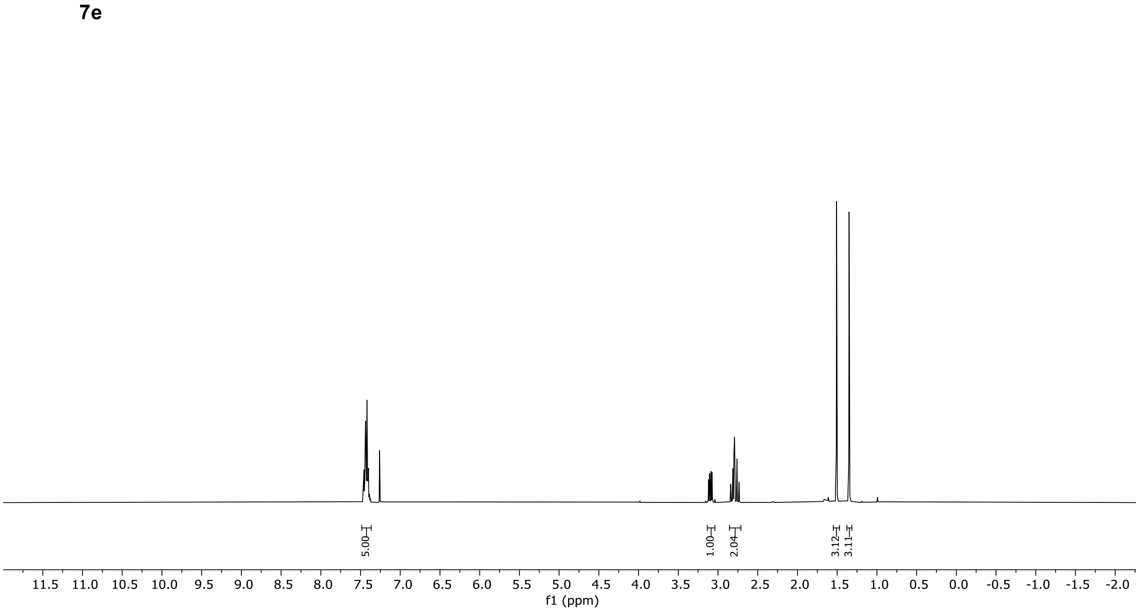

^{13}C NMR (126 MHz, $CDCl_3$) of **7e**

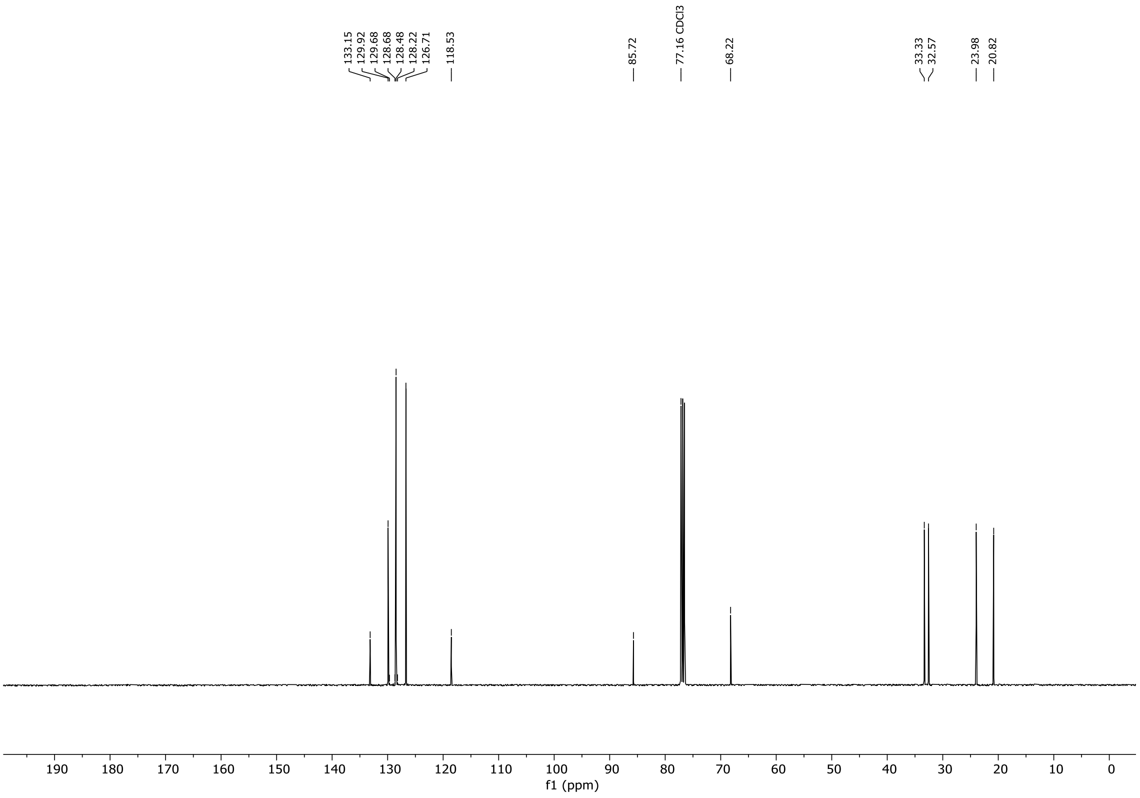

^{1}H NMR (500 MHz, $CDCl_3$) of **5a** (see procedure)

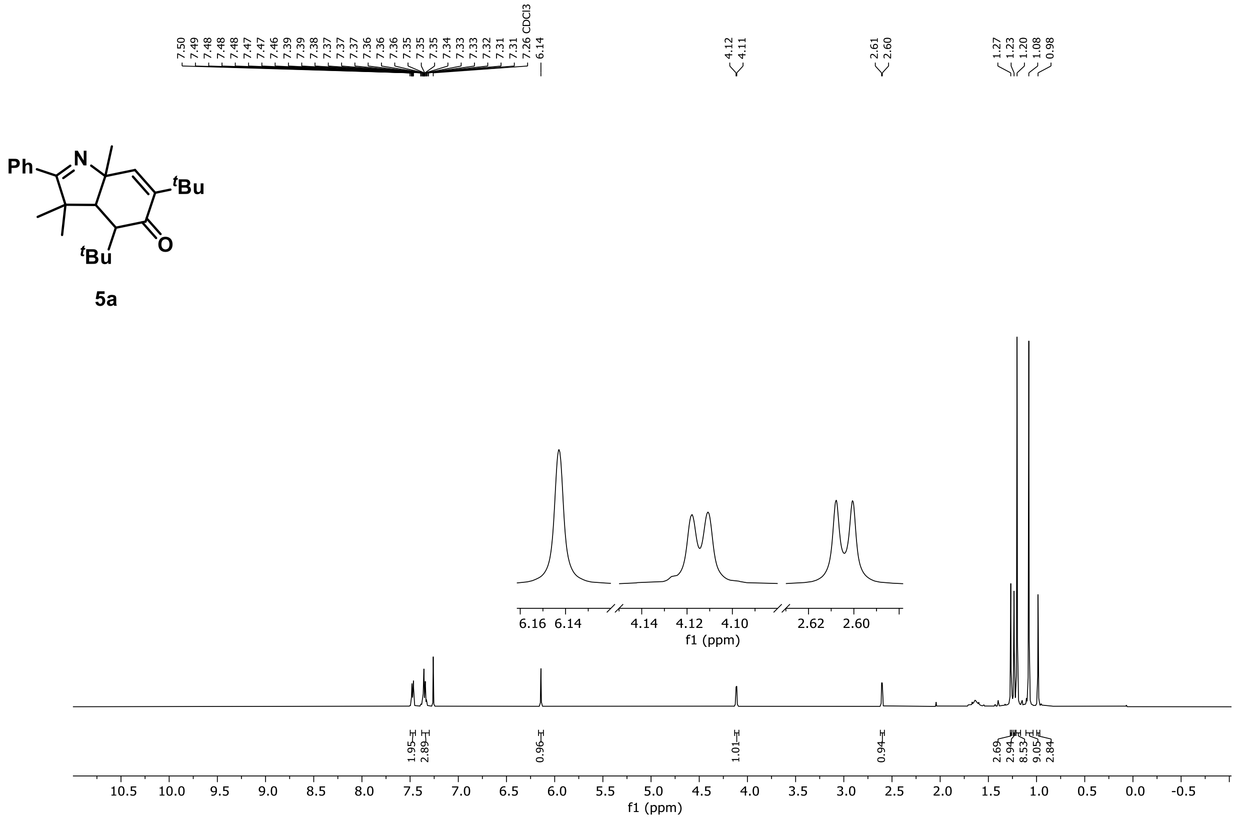

^{13}C NMR (126 MHz, $CDCl_3$) of **5a**

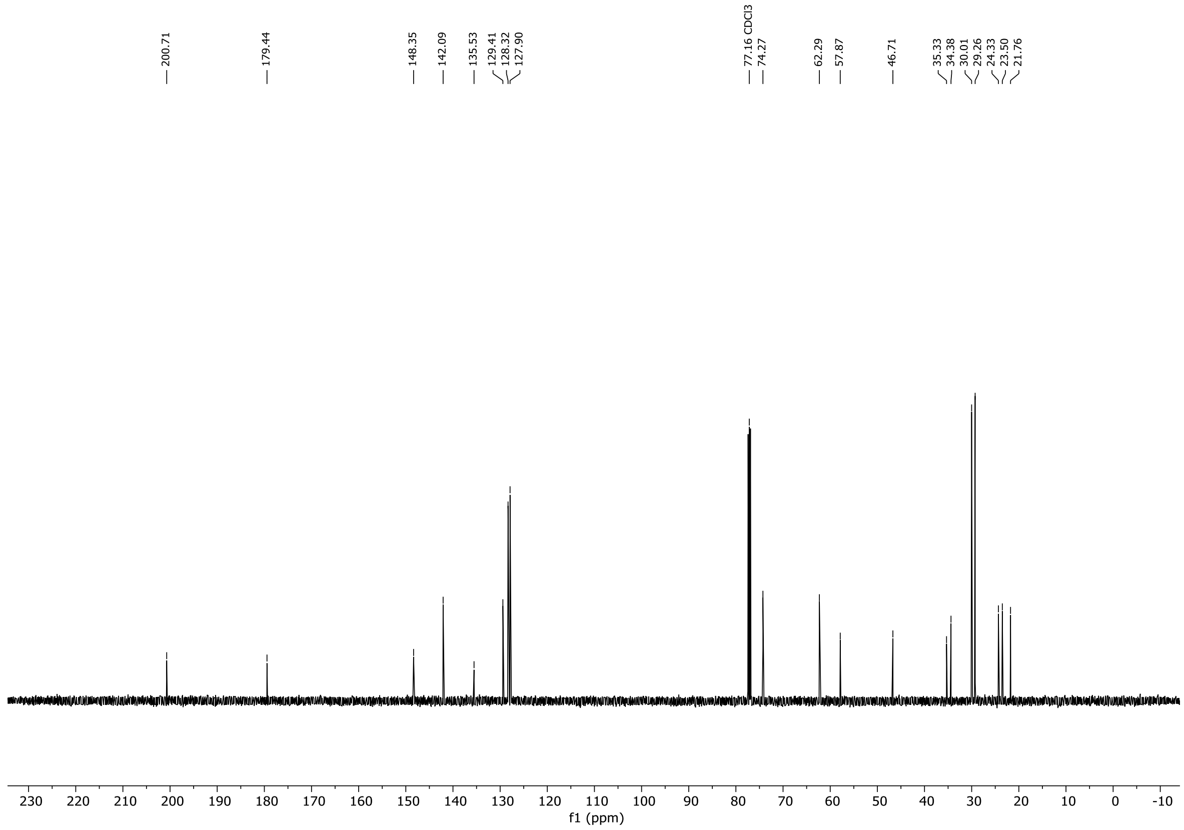

^{1}H NMR (400 MHz, $CDCl_3$) of **S6** (*see procedure*)

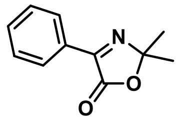

S6

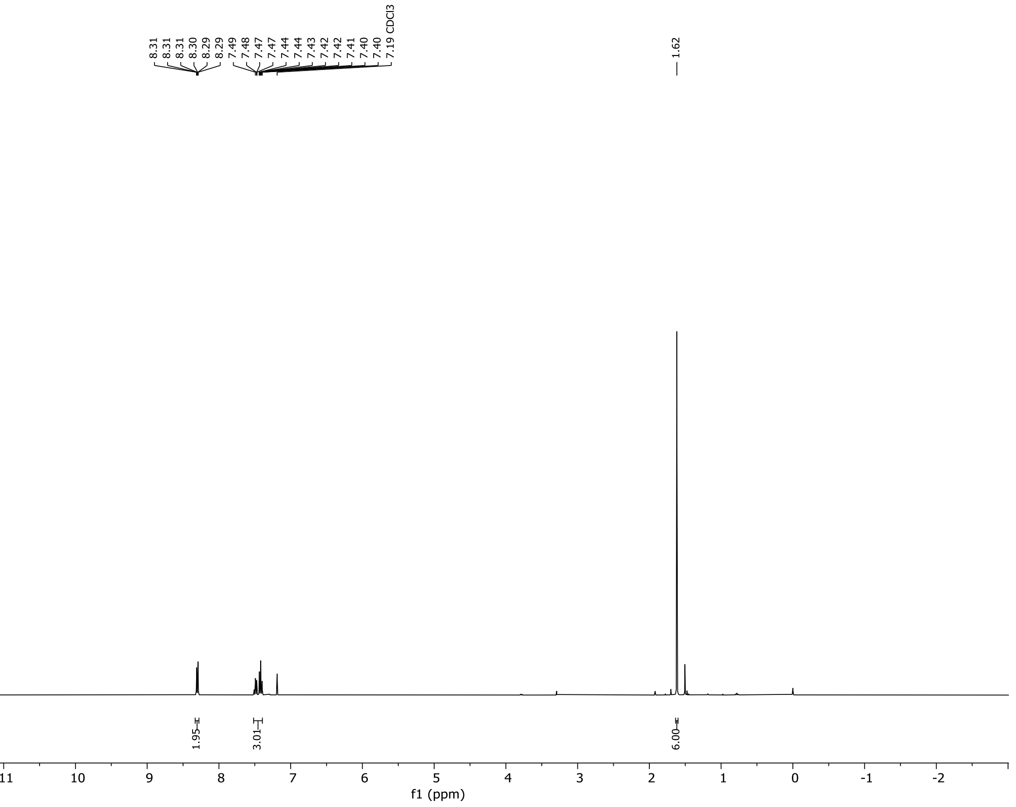

^{13}C NMR (126 MHz, $CDCl_3$) of **S6**

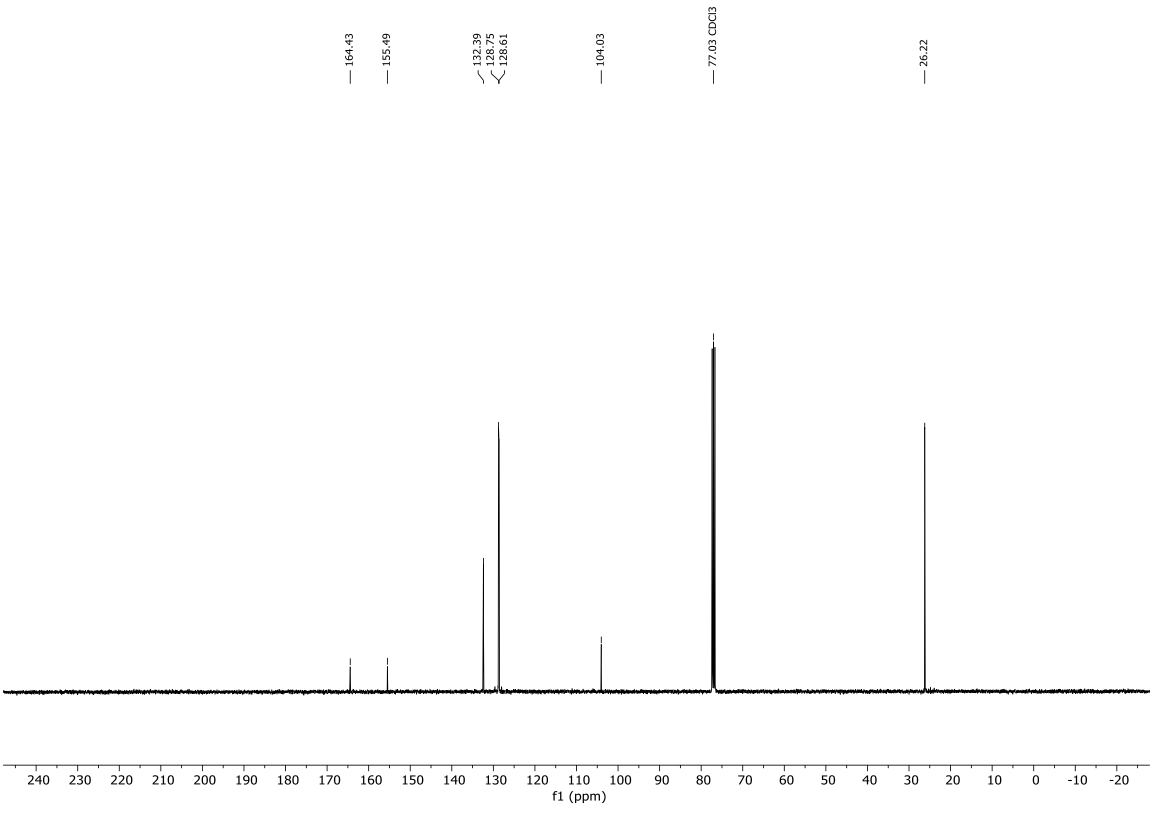

9. REFERENCES

(1) Too, P. C.; Wang, Y.-F.; Chiba, S. Rhodium(III)-catalyzed synthesis of isoquinolines from aryl ketone O-acyloxime derivatives and internal alkynes. *Org. Lett.* **2010**, *12*, 5688–5691.
(2) Jiang, Y.; Xi, S.; Wang, Q.; Fu, L.; He, L.; Wang, Z.; Zhang, M. Facile synthesis of δ-ketoesters via formal two-carbon insertion into β-ketoesters. *Tetrahedron Lett.* **2022**, *92*, 153656.
(3) Zhang, C.-C.; Chen, L.-J.; Shen, B.-C.; Xie, H.-D.; Li, W.; Sun, Z.-W. Enantioselective decarboxylative Mannich reaction of β-keto acids with C-alkynyl N-Boc N,O-acetals: access to chiral β-keto propargylamines. *Org. Biomol. Chem.* **2021**, *19*, 8607–8612.
(4) Wang, Z.-H.; Wang, D.-H. Cu-Catalyzed Synthesis of Benzoxazole with Phenol and Cyclic Oxime. *Org. Lett.* **2022**, *24*, 782–785.
(5) Okamoto, K.; Shimbayashi, T.; Tamura, E.; Ohe, K. Palladium-catalyzed aza-Wittig-type condensation of isoxazol-5(4H)-ones with aldehydes. *Chem* **2014**, *20*, 1490–1494.
(6) Okamoto, K.; Shimbayashi, T.; Yoshida, M.; Nanya, A.; Ohe, K. Synthesis of 2H-Azirines by Iridium-Catalyzed Decarboxylative Ring Contraction of Isoxazol-5(4H)-ones. *Angew. Chem. Int. Ed.* **2016**, *55*, 7199–7202.
(7) Feng, L.; Yang, C.; Xia, W. Visible-Light Promoted Selective Imination of Unactivated C-H Bonds via Copper-nitrene Intermediates for the Synthesis of 2H-Azirines. *Org. Lett.* **2019**, *21*, 8323–8327.
(8) Wang, R.; Wang, Y.; Ding, R.; Staub, P. B.; Zhao, C. Z.; Liu, P.; Wang, Y.-M. Designed Iron Catalysts for Allylic C-H Functionalization of Propylene and Simple Olefins. *Angew. Chem. Int. Ed.* **2023**, *62*, e202216309.
(9) Cornil, J.; Echeverria, P.-G.; Reymond, S.; Phansavath, P.; Ratovelomanana-Vidal, V.; Guérinot, A.; Cossy, J. Synthetic Studies toward the C14-C29 Fragment of Mirabalin. *Org. Lett.* **2016**, *18*, 4534–4537.
(10) Guo, F.; Fang, S.; He, J.; Su, Z.; Wang, T. Enantioselective organocatalytic synthesis of axially chiral aldehyde-containing styrenes via SNAr reaction-guided dynamic kinetic resolution. *Nat. Commun.* **2023**, *14*, 5050.
(11) Ociepa, M.; Wierzba, A. J.; Turkowska, J.; Gryko, D. Polarity-Reversal Strategy for the Functionalization of Electrophilic Strained Molecules via Light-Driven Cobalt Catalysis. *J. Am. Chem. Soc.* **2020**, *142*, 5355–5361.
(12) Li, B.; Zeng, W.; Wang, L.; Geng, Z.; Loh, T.-P.; Xie, P. Visible-Light-Induced Trifluoromethylation of Allylic Alcohols. *Org. Lett.* **2021**, *23*, 5235–5240.
(13) Zhao, J.-H.; Zheng, L.; Zou, J.-Y.; Zhang, S.-Y.; Shen, H.-C.; Wu, Y.; Wang, P. Construction of Si-Stereogenic Silanols by Palladium-Catalyzed Enantioselective C-H Alkenylation. *Angew. Chem. Int. Ed.* **2024**, *63*, e202402612.
(14) Akhmedov, A.; Gamirov, R.; Panina, Y.; Sokolova, E.; Leonteva, Y.; Tarasova, E.; Potekhina, R.; Fitsev, I.; Shurpik, D.; Stoikov, I. Towards potential antifungal agents: synthesis, supramolecular self-assembly and in vitro activity of azole mono-, sesqui- and diterpenoids. *Org. Biomol. Chem.* **2023**, *21*, 4863–4873.
(15) Duchemin, N.; Cattoen, M.; Gayraud, O.; Anselmi, S.; Siddiq, B.; Buccafusca, R.; Daumas, M.; Ferey, V.; Smietana, M.; Arseniyadis, S. Direct Access to Highly Enantioenriched α-Branched Acrylonitriles through a One-Pot Sequential Asymmetric Michael Addition/Retro-Dieckmann/Retro-Michael Fragmentation Cascade. *Org. Lett.* **2020**, *22*, 5995–6000.
(16) Fang, S.; Chen, L.; Yu, M.; Cheng, B.; Lin, Y.; Morris-Natschke, S. L.; Lee, K.-H.; Gu, Q.; Xu, J.

Synthesis, antitumor activity, and mechanism of action of 6-acrylic phenethyl ester-2-pyranone derivatives. *Org. Biomol. Chem.* **2015**, *13*, 4714–4726.

(17) Kranz, D. P.; Chiha, S.; Meier zu Greffen, A.; Neudörfl, J.-M.; Schmalz, H.-G. Synthesis of B-ring-modified steroids through BF3-promoted rearrangement/substitution of 6β-hydroxy-5,19-cyclosteroids. *Org. Lett.* **2012**, *14*, 3692–3695.

(18) Laskar, R.; Dutta, S.; Spies, J. C.; Mukherjee, P.; Rentería-Gómez, Á.; Thielemann, R. E.; Daniliuc, C. G.; Gutierrez, O.; Glorius, F. γ-Amino Alcohols via Energy Transfer Enabled Brook Rearrangement. *J. Am. Chem. Soc.* **2024**, *146*, 10899–10907.

(19) Stivanin, M. L.; Fernandes, A. A. G.; da Silva, A. F.; Okada, C. Y.; Jurberg, I. D. Blue Light‐Promoted N−H Insertion of Carbazoles, Pyrazoles and 1,2,3‐Triazoles into Aryldiazoacetates. *Adv. Synth. Catal.* **2020**, *362*, 1106–1111.

(20) Yang, K.; Li, R.; Gu, P. Electrocatalytic conversion of ω-azido carboxylic acids to 1-pyrrolines via a combined process of oxidative decarboxylation and intramolecular Schmidt rearrangement. *Org. Chem. Front.* **2024**, *11*, 3391–3396.

(21) Pitzer, L.; Schäfers, F.; Glorius, F. Rapid Assessment of the Reaction-Condition-Based Sensitivity of Chemical Transformations. *Angew. Chem. Int. Ed.* **2019**, *58*, 8572–8576.

(22) Collins, K. D.; Glorius, F. A robustness screen for the rapid assessment of chemical reactions. *Nat. Chem.* **2013**, *5*, 597–601.

(23) Xuan, J.; Xia, X.-D.; Zeng, T.-T.; Feng, Z.-J.; Chen, J.-R.; Lu, L.-Q.; Xiao, W.-J. Visible-light-induced formal 3+2 cycloaddition for pyrrole synthesis under metal-free conditions. *Angew. Chem. Int. Ed.* **2014**, *53*, 5653–5656.

(24) Devi, L.; Mishra, P.; Pokhriyal, A.; Rastogi, N. Organo-photocatalytic Synthesis of Functionalized Pyrroles from 2H-Azirines and α-Substituted Nitroalkenes. *SynOpen* **2022**, *06*, 198–207.

(25) Romero, N. A.; Nicewicz, D. A. Organic Photoredox Catalysis. *Chem. Rev.* **2016**, *116*, 10075–10166.

(26) Strieth-Kalthoff, F.; James, M. J.; Teders, M.; Pitzer, L.; Glorius, F. Energy transfer catalysis mediated by visible light: principles, applications, directions. *Chem. Soc. Rev.* **2018**, *47*, 7190–7202.

(27) Prier, C. K.; Rankic, D. A.; MacMillan, D. W. C. Visible light photoredox catalysis with transition metal complexes: applications in organic synthesis. *Chem. Rev.* **2013**, *113*, 5322–5363.

(28) Glorius, F.; Katzenburg, F.; Boser, F.; Schäfer, F.; Pflüger, P. Calibration-Free Quantification and Automated Data Analysis for High-Throughput Reaction Screening. *ChemRxiv* **2024**, doi: 10.26434/chemrxiv-2024-1ctkh.

(29) Dittwald, P.; Claesen, J.; Burzykowski, T.; Valkenborg, D.; Gambin, A. BRAIN: a universal tool for high-throughput calculations of the isotopic distribution for mass spectrometry. *Anal. Chem.* **2013**, *85*, 1991–1994.

(30) GitHub, "mobiusklein/brainpy: A Python implementation of Baffling Recursive Algorithm for Isotopic distribution calculations", can be found under https://github.com/mobiusklein/brainpy.

(31) Neese, F. Software update: The ORCA program system—Version 5.0. *WIREs Comput Mol Sci* **2022**, *12*.

(32) Hanwell, M. D.; Curtis, D. E.; Lonie, D. C.; Vandermeersch, T.; Zurek, E.; Hutchison, G. R. Avogadro: an advanced semantic chemical editor, visualization, and analysis platform. *Journal of cheminformatics* **2012**, *4*, 17.

(33) Schlosser, L.; Rana, D.; Pflüger, P.; Katzenburg, F.; Glorius, F. EnTdecker - A Machine Learning-Based

Platform for Guiding Substrate Discovery in Energy Transfer Catalysis. *Journal of the American Chemical Society* **2024**, *146*, 13266–13275.

(34) Bannwarth, C.; Ehlert, S.; Grimme, S. GFN2-xTB-An Accurate and Broadly Parametrized Self-Consistent Tight-Binding Quantum Chemical Method with Multipole Electrostatics and Density-Dependent Dispersion Contributions. *Journal of chemical theory and computation* **2019**, *15*, 1652–1671.

(35) Becke, A. D. Density-functional thermochemistry. III. The role of exact exchange. *The Journal of Chemical Physics* **1993**, *98*, 5648–5652.

(36) Weigend, F.; Ahlrichs, R. Balanced basis sets of split valence, triple zeta valence and quadruple zeta valence quality for H to Rn: Design and assessment of accuracy. *Physical chemistry chemical physics : PCCP* **2005**, *7*, 3297–3305.

(37) Chai, J.-D.; Head-Gordon, M. Long-range corrected hybrid density functionals with damped atom-atom dispersion corrections. *Physical chemistry chemical physics : PCCP* **2008**, *10*, 6615–6620.

(38) Grimme, S.; Antony, J.; Ehrlich, S.; Krieg, H. A consistent and accurate ab initio parametrization of density functional dispersion correction (DFT-D) for the 94 elements H-Pu. *The Journal of Chemical Physics* **2010**, *132*, 154104.

(39) Barone, V.; Cossi, M. Quantum Calculation of Molecular Energies and Energy Gradients in Solution by a Conductor Solvent Model. *J. Phys. Chem. A* **1998**, *102*, 1995–2001.

(40) Ogawa, A.; Curran, D. P. Benzotrifluoride: A Useful Alternative Solvent for Organic Reactions Currently Conducted in Dichloromethane and Related Solvents. *The Journal of organic chemistry* **1997**, *62*, 450–451.

(41) Helmich-Paris, B.; Souza, B. de; Neese, F.; Izsák, R. An improved chain of spheres for exchange algorithm. *The Journal of Chemical Physics* **2021**, *155*, 104109.

(42) Popescu, M. V.; Paton, R. S. Dynamic vertical triplet energies: Understanding and predicting triplet energy transfer. *Chem* **2024**, *10*, 3428–3443.

(43) Frisch, M.J., Trucks, G.W., Schlegel, H.B., Scuseria, G.E., Robb, M.A., Cheeseman, J.R.; Scalmani, G.; Barone, V.; Petersson, G. A.; Nakatsuji, H.; Li, X.; Caricato, M.; Marenich, A. V.; Bloino, J., Janesko, B.G., Gomperts, R., Mennucci, B., Hratchian, H.P., Ortiz, J.V., Izmaylov, A.F., Sonnenberg, J.L., Williams-Young, D., Ding, F., Lipparini, F., Egidi, F., Goings, J., Peng, B., Petrone, A., Henderson, T., Ranasinghe, D., Zakrzewski, V.G., Gao, J., Rega, N., Zheng, G., Liang, W., Hada, M., Ehara, M., Toyota, K., Fukuda, R., Hasegawa, J., Ishida, M., Nakajima, T., Honda, Y., Kitao, O., Nakai, H., Vreven, T., Throssell, K., Montgomery Jr., J.A., Peralta, J.E., Ogliaro, F., Bearpark, M.J., Heyd, J.J., Brothers, E.N., Kudin, K.N., Staroverov, V.N., Keith, T.A., Kobayashi, R., Normand, J., Raghavachari, K., Rendell, A.P., Burant, J.C., Iyengar, S.S., Tomasi, J., Cossi, M., Millam, J.M., Klene, M., Adamo, C., Cammi, R., Ochterski, J.W., Martin, R.L., Morokuma, K., Farkas, O., Foresman, J.B., Fox, D.J. *Gaussian 16*; Gaussian, Inc.: Wallingford CT, 2016.

(44) M. S. Teynor, N. Wohlgemuth, L. Carlson, J. Huang, S. L. Pugh, B. O. Grant, R. S. Hamilton, R. Carlsen, and D. H. Ess. *Milo*; Brigham Young University: Provo UT, 2021.

(45) Hunter, J. D. Matplotlib: A 2D Graphics Environment. *Comput. Sci. Eng.* **2007**, *9*, 90–95.

(46) *Scikit-learn: Machine learning in Python*, 2011.

(47) Zähringer, T. J. B.; Wienhold, M.; Gilmour, R.; Kerzig, C. Direct Observation of Triplet States in the Isomerization of Alkenylboronates by Energy Transfer Catalysis. *J. Am. Chem. Soc.* **2023**, *145*, 21576–21586.

(48) Bruker AXS. APEX4 Version 2021.4-0, SAINT Version 8.40B and SADABS Bruker AXS area detector

scaling and absorption correction Version 2016/2, Bruker AXS Inc., 2021.

(49) Sheldrick, G. M. SHELXT - integrated space-group and crystal-structure determination. *Actra Cryssstalogr. A, Found. Adv.* **2015**, *71*, 3–8.

(50) Sheldrick, G. M. Crystal structure refinement with SHELXL. *Acta Crystallogr. C: Struct. Chem.* **2015**, *71*, 3–8.

(51) Bruker AXS. XP – Interactive molecular graphics, Version 5.1, Bruker AXS Inc., 1998.
